# PROFET Predicts Continuous Gene Expression Dynamics from scRNA-seq Data to Elucidate Heterogeneity of Cancer Treatment Responses

**DOI:** 10.1101/2025.06.27.662030

**Published:** 2025-07-03

**Authors:** Yu-Chen Cheng, Hyemin Gu, Thomas O. McDonald, Wenbo Wu, Shubham Tripathi, Cristina Guarducci, Douglas Russo, Daniel L. Abravanel, Madeline Bailey, Yue Wang, Yun Zhang, Yannis Pantazis, Herbert Levine, Rinath Jeselsohn, Markos A. Katsoulakis, Franziska Michor

**Affiliations:** 1 Department of Data Science, Dana-Farber Cancer Institute, Boston, MA, USA; 2 Department of Biostatistics, Harvard T.H. Chan School of Public Health, Boston, MA, USA; 3 Center for Cancer Evolution, Dana-Farber Cancer Institute, Boston, MA, USA; 4 Department of Stem Cell and Regenerative Biology, Harvard University, Cambridge, MA, USA; 5 Department of Mathematics and Statistics, University of Massachusetts Amherst, Amherst, MA, USA; 6 Harvard-MIT Health Sciences and Technology, Cambridge, MA, USA; 7 Yale Center for Systems and Engineering Immunology and Department of Immunobiology , Yale School of Medicine, New Haven, CT, USA; 8 Department of Medical Oncology, Dana-Farber Cancer Institute, Boston, MA, USA; 9 Harvard Medical School, Boston, MA; 10 Harvard John A. Paulson School of Engineering and Applied Sciences, Harvard University, Cambridge, MA, USA; 11 Irving Institute for Cancer Dynamics and Department of Statistics, Columbia University, New York, NY, USA; 12 State Key Laboratory of Molecular Oncology, National Cancer Center/National Clinical Research Center for Cancer/Cancer Hospital, Chinese Academy of Medical Sciences and Peking Union Medical College, Beijing, China; 13 Institute of Applied and Computational Mathematics, Foundation for Research and Technology-Hellas, Greece; 14 Center for Theoretical Biological Physics, Northeastern University, Boston, MA, USA; 15 Department of Physics, Northeastern University, Boston, MA, USA; 16 Center for Functional Cancer Epigenetics, Dana-Farber Cancer Institute, Boston, MA, USA; 17 Breast Oncology Center, Dana-Farber Cancer Institute, Boston, MA, USA; 18 The Broad Institute of MIT and Harvard, Cambridge, MA, USA; 19 The Ludwig Center at Harvard, Boston, MA, USA

## Abstract

Single-cell RNA sequencing captures static snapshots of gene expression but lacks the ability to track continuous gene expression dynamics over time. To overcome this limitation, we developed PROFET (Particle-based Reconstruction Of generative Force-matched Expression Trajectories), a computational framework that reconstructs continuous, nonlinear single-cell gene expression trajectories from sparsely sampled scRNA-seq data. PROFET first generates particle flows between time-stamped samples using a novel Lipschitz-regularized gradient flow approach and then learns a global vector field for trajectory reconstruction using neural force-matching. The framework was developed using synthetic data simulating cell state transitions and subsequently validated on both mouse and human *in vitro* datasets. We then deployed PROFET to investigate heterogeneity in treatment responses to palbociclib, a CDK4/6 inhibitor, in hormone receptor positive breast cancer. By comparing newly generated scRNA-seq data from a palbociclib-resistant breast cancer cell line with published patient-derived datasets, we identified a subpopulation of patient cells exhibiting profound phenotypic shifts in response to treatment, along with surface markers uniquely enriched in those cells. By recovering temporal information from static snapshots, PROFET enables inference of continuous single-cell expression trajectories, providing a powerful tool for dissecting the heterogeneity of cell state transitions in treatment responses.

## Introduction

Modern live single-cell imaging technologies, such as those using fluorescently labeled reporter proteins, offer high temporal resolution for tracking gene expression dynamics at the single-cell level^1^. Despite this advantage, live-cell experimental approaches are limited by the small number of markers that can be tracked simultaneously, challenges in maintaining system stability, and the complexity of analyzing imaging data over prolonged periods^2^. In contrast, scRNA-seq technologies provide invaluable insights into cellular heterogeneity by capturing genome-wide expression patterns^3,4^, but face challenges as expression patterns can vary over time due to cell state changes, intrinsic gene expression noise and experimental variability. Critically, scRNA-seq data are inherently cross-sectional, as individual cells must be lysed for sequencing at each time point. These constraints limit the ability to reconstruct true single-cell trajectories from noisy time-course scRNA-seq datasets^5^.

As a result, neither live-cell imaging nor scRNA-seq alone can provide both the high temporal resolution and broad gene expression profiling required to capture dynamic changes in cellular states. To bridge this gap, recent data-driven, nonparametric approaches have leveraged optimal transport (OT) theory to infer cellular trajectories by linking distributions of cell states across discrete time points^6,7^. While OT-based methods are effective at estimating transition probabilities, they rely on the strong assumption that cells traverse gene expression space along the shortest paths, typically defined under the Wasserstein-2 (W2) metric^8^. This assumption corresponds to a linear interpolation between time points, which may fail to capture the nonlinear nature of many biological processes. When applied to datasets with more than two time points, OT methods produce multiple piecewise-linear interpolations, which introduce discontinuities in the reconstructed trajectories resulting in biologically implausible transitions.

To overcome these limitations, we developed a computational framework, termed PROFET (Particle-based Reconstruction Of generative Force-matched Expression Trajectories), which reconstructs continuous single-cell trajectories from discrete scRNA-seq time points. The framework consists of two key steps: first, it applies a Lipschitz-regularized gradient flow — distinct from OT methods—to interpolate between each pair of adjacent time points^9,10^; second, it trains a neural network on the piecewise interpolations to learn a globally smooth, time-dependent vector field that captures the entire cell state transition process. This two-step approach enables the reconstruction of continuous cell state trajectories across multiple time points, even under strong external perturbations—such as drug treatments—where trajectories become highly nonlinear and time-dependent, features that OT-based methods cannot capture.

PROFET was first developed using synthetic data simulating the epithelial-mesenchymal transition (EMT)^11^ in order to determine hyperparameters; the method was then validated using two *in vitro* time-series scRNA-seq datasets, one profiling mouse embryonic stem cell differentiation and the other capturing EMT induced by TGF-β^12,13^. We then applied the validated model to a newly generated scRNA-seq dataset from the MCF7 hormone receptor positive (HR+) breast cancer cell line as well as three datasets from HR+ metastatic breast cancer patients^14,15^ to reconstruct single-cell trajectories spanning pre- and post-palbociclib time points. Palbociclib, a CDK4/6 inhibitor, has demonstrated significant therapeutic benefit in HR+ breast cancer; however, acquired resistance to CDK4/6 inhibition has been widely reported in both clinical and preclinical studies^16,17^.

In this study of the MCF7 cell line, 16 weeks of palbociclib treatment led to experimentally confirmed drug resistance, accompanied by marked downregulation of cell cycle–related genes. To compare this response with that obtained from patient-derived data, we identified a subpopulation of cells in the patient samples that exhibited similarly pronounced phenotypic shifts, characterized by the same downregulation signature of cell cycle genes. Differential gene expression (DEG) analysis revealed that six surface markers— *UNC5B*, *TLR3*, *PCDH19*, *PROCR*, *SLITRK6*, and *SEMA6B* —were enriched in the pre-treatment states of these high-shift cells. These markers may serve as early predictors of phenotypic transitions in response to palbociclib treatment and potential targets for antibody-drug conjugates (ADCs)^18^. More broadly, PROFET provides a generalizable approach for reconstructing gene expression trajectories from time-series scRNA-seq data across phenotypically heterogeneous cell populations.

## Results

### Overview of Methods

**Figure 1.**
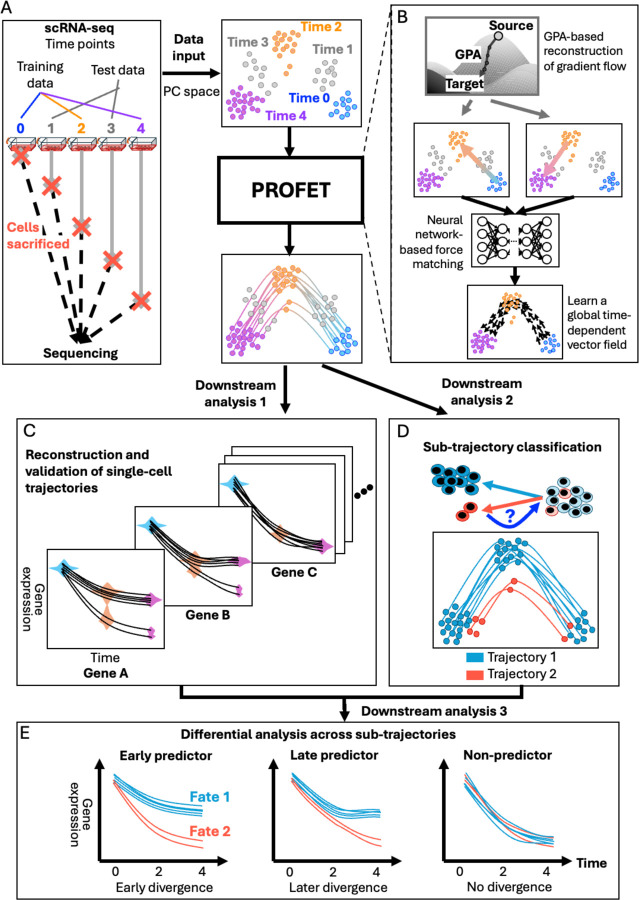
Reconstruction of continuous single-cell gene expression dynamics from discrete scRNA-seq data. (A) Study objective and approach. Left: Existing scRNA-seq technologies capture only static snapshots of cell states, limiting the ability to observe continuous trajectories. Right: To address this limitation, we developed PROFET to reconstruct continuous gene expression dynamics from sampled scRNA-seq time points (colored), while masking intermediate time points (gray) for validation. (B) Two-step architecture of PROFET. Step 1: Apply Generative Particle Algorithm (GPA) to infer gradient flows interpolating between observed distributions at adjacent time points. Step 2: Use a neural network to perform force-matching on these flows and learn a globally smooth, time-dependent vector field that models cell dynamics. (C–E) Three key downstream analyses enabled by our method. (C) Prediction of continuous single-cell gene expression dynamics. (D) Classification of predicted trajectories into terminal fates, enabling tracing fate-assigned cells to their origins. (E) Identification of diverse temporal gene expression patterns associated with distinct fates, illustrated by three representative genes: left panel shows early divergence, middle shows late divergence, and right shows no divergence.

To reconstruct complete trajectories from discrete scRNA-seq measurements, we employ a two-step framework to infer the underlying temporal dynamics using force-based generative models trained with a deep neural network (Figure 1A).

In the first step, we apply the Generative Particle Algorithm (GPA)^9,10^, which interpolates gene expression patterns by propagating initial cell states (source) along the gradient of a potential landscape (Figure 1B, Methods). Unlike traditional OT-based methods that assume W2-geodesics—i.e., shortest paths in probability space—GPA allows for nonlinear, data-driven flows by defining a potential based on a regularized Kullback-Leibler (KL) divergence with respect to the target distribution (target). This KL-based formulation has been widely used to model Waddington-like landscapes in developmental biology^21–23^. GPA offers key advantages: it constructs the potential landscape nonparametrically from data, avoids the geometric bias of continuous normalizing flows (CNFs)^19,24^ that require predefined source distributions (e.g., Gaussian), and enables flexible transport of empirical or irregular distributions—in a fully agnostic and mathematically principled manner^25^. To ensure biologically plausible transitions, GPA also incorporates a Lipschitz regularization on the gradient flow, which bounds instantaneous changes in cell states and enhances stability^10^ (Supplementary Notes 1–4).

In the second step, the outputs from GPA are used to train a neural network that learns a continuous, time-dependent vector field defined on an Eulerian grid using a force-matching scheme^26,27^ (Methods and Supplementary Note 5). This novel framework bridges the trajectory-based (Lagrangian) and field-based (Eulerian) representations of cell dynamics^28^, allowing us to reconstruct full gene expression space velocity fields from the time-varying trajectories simulated by GPA in Step 1. This design is biologically motivated: in many systems, particularly under perturbations (e.g., drug treatment or mutation), the underlying regulatory landscape is non-stationary and evolves over time. Our two-step approach accommodates this dynamic nature by lifting piecewise Lagrangian trajectories via force-matching into a global time-dependent vector field, enabling the reconstruction of smooth, nonlinear cellular trajectories that reflect time-inhomogeneous perturbation effects.

Building on this foundation, we performed three key downstream analyses to interpret PROFET outputs in biologically meaningful ways (Methods). First, to reconstruct single-gene dynamics, we applied an inverse transformation to map the inferred single-cell trajectories—originally reconstructed in a low-dimensional principal component (PC) space—back to the full gene expression space defined by all input genes. This step enables recovery of continuous gene expression dynamics for every gene in every individual cell (Figure 1C). Second, we implemented two strategies to classify predicted trajectories. The first assigns trajectories to predefined cell subpopulations based on clustering cells at a specific reference time point, allowing interpretation in terms of fate or origin (Figure 1D illustrates this classification applied to terminal fates). The second strategy quantifies the magnitude of cell state transitions across time by computing trajectory-wise Euclidean distances, which are then thresholded into distinct transition groups. Finally, by integrating the above classifications, we performed time-resolved differential gene expression analysis across sub-trajectories. This approach enabled us to identify not only which marker genes distinguish different trajectories, but also when these genes begin to diverge. These analyses uncover temporal transcriptional programs that underlie divergent treatment responses (Figure 1E).

### Model Validation Across Datasets: Hyperparameter Consistency and Dynamic Accuracy

**Figure 2.**
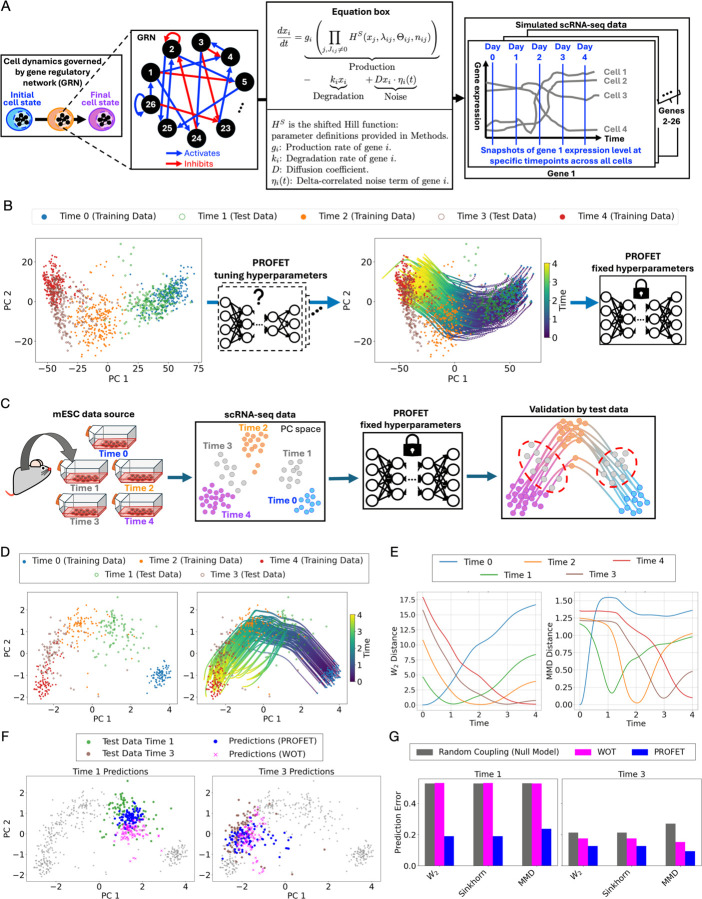
PROFET model development and validation process. (A) Overview of the synthetic data generation process simulating TGF-β–induced EMT using a gene regulatory network model. (B) PROFET development using synthetic EMT data. The model is trained using input from time points 0, 2, and 4 to reconstruct full single-cell trajectories. Hyperparameters were selected to ensure stable, non-divergent trajectories that align with the distributions at the withheld test time points. (C) Schematic of the validation process using mESC differentiation data. The model, with fixed hyperparameters, is applied to reconstruct trajectories from input data at time points 0, 2, and 4, and evaluated using held-out data from time points 1 and 3. (D) Principal component analysis (PCA) of the mESC dataset (left), and predicted single-cell trajectories by PROFET (right), with the trajectories colored by predicted time from day 0 to day 4. (E) Distribution distance between predicted trajectories and ground truth snapshots at each time point, computed using W2 (left) and MMD (right). (F) Comparison of predicted cell states at test time points (days 1 and 3) across three methods: PROFET (blue dots); WOT (magenta crosses), which uses an optimal transport plan and linear interpolation; and random coupling (black crosses), which applies a uniform transport plan with linear interpolation. (G) Prediction errors measured by W2, Sinkhorn, and MMD metrics across PROFET (blue), WOT (magenta), and random coupling (black), showing consistent outperformance of the other two methods by PROFET.

Synthetic data were used to determine the neural network hyperparameters required for learning the global Eulerian vector field from Lagrangian particle trajectories generated by GPA. In this synthetic dataset, we simulated single-cell trajectories based on a model of epithelial-mesenchymal transition (EMT) —a well-characterized cell state transition process that also serves as the focus of one of our *in vitro* validation datasets^14^. EMT has mechanistic parallels to stem cell differentiation^29,30^, which is represented by another *in vitro* validation dataset. Furthermore, given the well-established role of EMT in cancer metastasis^31^, the hyperparameters optimized using the EMT synthetic dataset serve as default parameters when applying PROFET to the metastatic breast cancer data analyzed below. The synthetic data were generated using a 26-gene regulatory network (GRN) specified by a system of stochastic differential equations (SDEs), which represent the gene–gene interactions underlying EMT dynamics^11^ (Figure 2A; Methods).

To capture the biological variability in EMT dynamics, we simulated GRN dynamics for an ensemble of random parameter sets sampled using a biologically-motivated scheme^32^; for each parameter set in the ensemble, the dynamics were simulated from random initial conditions (Methods and Supplementary Note 6). Using this approach, we generated 500 trajectories for each of the 26 genes and selected gene expression values at five discrete time points to emulate temporally sparse scRNA-seq measurements (Figure 2A right). To train our model, we used three of the five available time points (days 0, 2, and 4) as inputs into PROFET (Figure 2B). Hyperparameters were empirically adjusted to produce stable, non-divergent trajectories that aligned with the distributions observed at the withheld test time points (days 1 and 3) (Methods and Figure 2B). After selecting the hyperparameters, we fixed the model and validated its performance using *in vitro* time-series scRNA-seq datasets profiling mouse embryonic stem cell (mESC) differentiation^12^ and EMT induced by TGF-beta treatment in human cells^13^ (see next section).

We first applied PROFET to a five time-point (0, 12h, 24h, 36h, 48h) scRNA-seq dataset of mESC differentiation (Figure 2C). To ensure consistency, we used the same preprocessed dataset as in the original study^12,33^ (Methods). Specifically, the preprocessing step involved filtering for the top 100 most variable genes, which served as an initial dimensionality reduction before applying principal component analysis (PCA). The first two principal components explained 61.3% of the total variance. When visualizing the five time points in a two-dimensional PCA space (Figure 2D, upper panel), we confirmed a key observation in the original study^12^: cell state transitions do not follow a simple linear trajectory from time 0 to time 4. Instead, a distinct turning point occurs at time 2, indicating a nonlinear differentiation process.

We then used PROFET to reconstruct single-cell trajectories using only the data from time points 0, 2, and 4 (Figure 2D). The intermediate time points (1 and 3) were withheld and treated as test data. To evaluate prediction accuracy, we computed distributional distances between the predicted trajectories and the withheld data using three complementary metrics: W2 distance, Sinkhorn divergence, and maximum mean discrepancy (MMD) (Methods). Across all metrics, we observed that the reconstructed trajectories aligned closely with the test data, with predicted minima occurring within 3.5% of the total differentiation timeline around time point 1, and within 0.5% around time point 3 (Figure 2E). These results indicate that PROFET accurately captures the temporal structure of cellular transitions, even at unobserved intermediate stages.

Importantly, our model outperformed both WOT and random coupling (null model) approaches in predicting the withheld time points as demonstrated through both qualitative visualizations (Figure 2F) and quantitative evaluations (Figure 2G). We conducted a comprehensive comparison of all three methods across various distance metrics, and the results consistently showed that PROFET achieved the lowest prediction errors. For example, across W2, Sinkhorn, and MMD metrics, PROFET exhibited less than 50% of the prediction error of WOT when reconstructing cell states at time point 1 (Figure 2G and Supplementary Figure 1). Notably, while WOT is explicitly trained to minimize W2 distance, PROFET is not—yet PROFET outperformed WOT on this benchmark, highlighting the robustness and generalizability of PROFET’s learned dynamics. We also benchmarked against TrajectoryNET^19^, a CNF-based method; however, TrajectoryNET failed to converge and produced predicted trajectories with implausible detours from the withheld time points (Supplementary Note 7).

### Predicting Gene Expression and Differentiation Pathways from Single-Cell Trajectories

**Figure 3.**
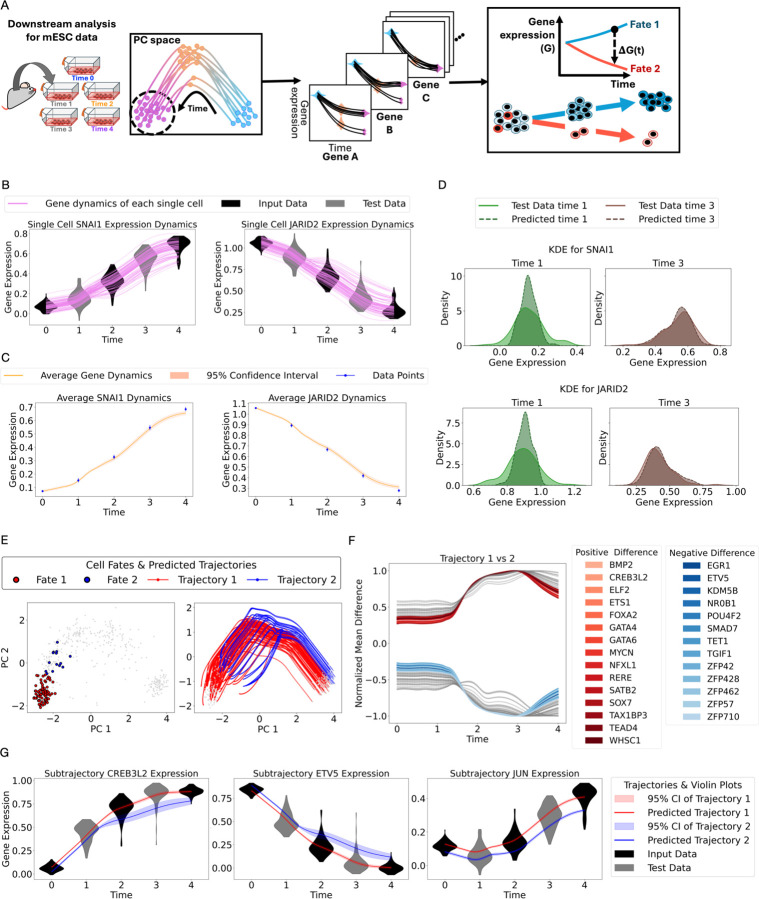
Downstream analysis of reconstructed single-cell trajectories for mESC data. (A) Overview of the downstream analysis pipeline for mESC differentiation. The process begins with PROFET-reconstructed single-cell trajectories in PCA space. These trajectories are then inverse-mapped back to the original gene expression space to recover dynamic gene expression profiles for each gene in each cell. Subsequently, we investigate gene expression differences across distinct sub-trajectories to identify the timing and magnitude of divergence associated with different terminal fates. ΔG(t) denotes the temporal difference in mean gene expression between sub-trajectories. (B) Single-cell, single-gene dynamics plotted as gene expression levels (y-axis) over time (x-axis), with each green curve representing an individual cell trajectory. The predicted trajectories are compared against real data distributions using violin plots (gray for test data, black for training data). (C) Predicted average gene expression trajectories (orange curves with shaded regions indicating 95% confidence intervals) compared to actual gene expression levels from real data (blue dots with error bars). (D) Comparison of predicted and actual gene expression distributions at day 1 (green) and day 3 (brown). Predicted distributions are shown as dashed lines, with real data distributions shown as solid lines. (E) Classification of differentiation fates and ancestral trajectory tracing. Left panel: Two distinct cell fate subgroups at day 4, identified through clustering analysis. Right panel: Ancestral trajectories of the two subgroups traced back to earlier time points. (F) Temporal quantification of gene expression divergence between fate groups. Normalized mean differences are plotted over time, with genes exceeding a divergence threshold of 0.5 highlighted. Positive values indicate higher expression in trajectory group 1, and negative values indicate higher expression in trajectory group 2. (G) Examples of gene expression divergence in the two subpopulations. Three representative genes are shown, with average gene expression trajectories plotted separately for each subgroup (shaded regions indicate 95% confidence intervals).

PROFET enables reconstruction of continuous single-cell gene expression trajectories for all genes in the mESC differentiation dataset. To this end, we applied an inverse PCA transformation to project the reconstructed trajectories from the low-dimensional PCA space back to the original high-dimensional gene expression space, defined by all genes in the input data (Methods). By aligning the trajectories with subgroup classifications, PROFET then enables analysis of gene expression differences across distinct subtrajectories (Figure 3A).

By performing this downstream analysis, we recovered the gene expression dynamics for each of the 100 genes across all individual cells in the mESC differentiation dataset. We highlight two representative genes, *JARID2* and *SNAI1*, both of which are known regulators of embryonic development^34,35^ (Figure 3B). Using three time points (time points 0, 2, and 4) for model input, PROFET successfully reconstructs full gene expression trajectories for individual cells, validated by accurate predictions at withheld time points (time points 1 and 3, treated as test data). To quantify prediction accuracy, we compared the mean gene expression along the reconstructed trajectories to the experimental data, finding that, for both test time points 1 and 3, the experimental means (with error bars) consistently fall within the 95% confidence intervals of our predicted means (Figure 3C). We further compared the full distributions of predicted and measured gene expression, observing no statistically significant differences for both genes (permutation test, *p* > 0.05 and Methods, Figure 3D). Complete results for all other genes are provided in Supplementary Figures 2–4 and Supplementary Table 1.

We next sought to investigate whether single-cell trajectories diverge into distinct cell fates during mESC differentiation. We performed cluster analysis on the final time point (time 4) of the scRNA-seq dataset (Methods), leading to the identification of two clusters representing different fate subpopulations (Figure 3E left). Leveraging the reconstructed single-cell trajectories, we assigned the final state of each trajectory to one of the two fate clusters by computing its distance to the cluster centroids and selecting the closer one (Methods). This approach enabled us to group trajectories into two fate-specific subpopulations (Figure 3E right).

To further examine which genes exhibit significant divergence between the two sub-trajectories, we computed the temporal difference of mean gene expression, normalized by the maximum difference between the groups from day 0 to day 4 (Methods). Genes with a normalized difference greater than 0.5 were considered substantially divergent (Figure 3F). The continuity of reconstructed trajectories also enabled us to pinpoint when these divergences emerged with high temporal resolution. Notably, we identified genes whose expression began to diverge between time points 1 and 2, shedding light on the timing of fate decisions (Figure 3F). Two representative genes, *CREB3L2* and *ETV5*, showed changes exceeding 0.5, in contrast to *JUN*, which exhibited a smaller change of 0.1. Their mean expression profiles across the two trajectory groups (Figure 3G) highlight distinct temporal patterns of gene expression divergence. Complete results for all other genes are provided in Supplementary Figure 5.

### Predicting Cell-State Convergence in EMT Dynamics

**Figure 4.**
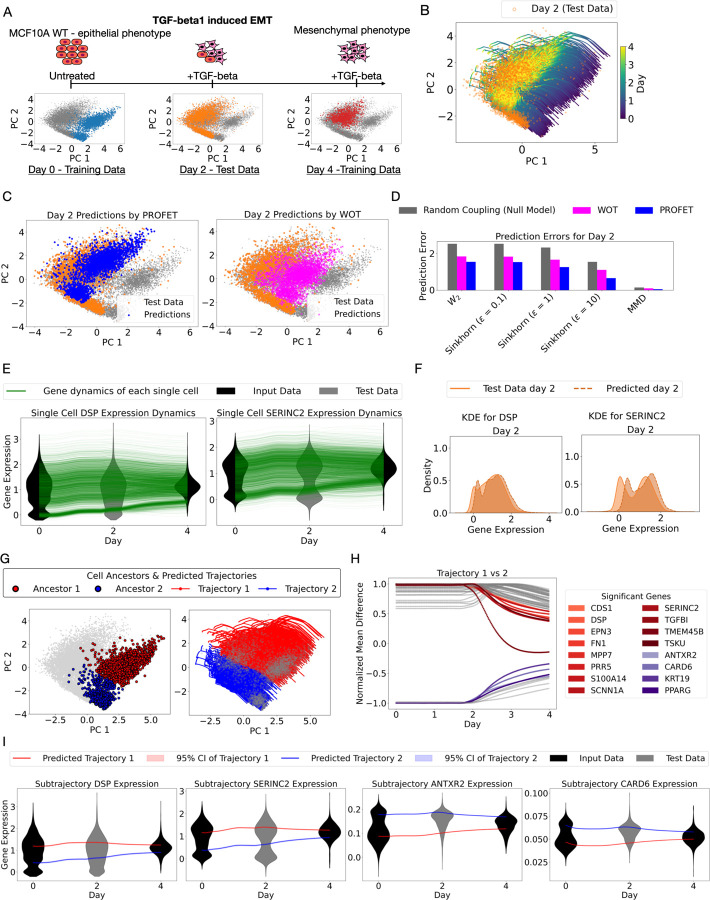
Downstream analysis of reconstructed single-cell trajectories for EMT data. (A) Illustration of EMT scRNA-seq data. (B) Application of PROFET to reconstruct continuous trajectories using only data from time points 0 and 4, with time point 2 held out for validation. The colormap represents the predicted time along the trajectory, ranging from time 0 to time 4, and the test data is highlighted by orange circles. (C) Comparison of test data predictions (time 2) between the PROFET (blue dots) and the WOT method (magenta crosses). (D) Quantitative comparison of prediction errors at time 2 across three methods—PROFET (blue), WOT (magenta), and random coupling (black)—measured using W2, Sinkhorn (entropy = 0.1, 1, 10), and MMD metrics. (E) Single-cell, single-gene dynamics, showing gene expression levels (y-axis) over time (x-axis). Each green curve represents an individual cell trajectory, with predicted trajectories compared against real data distributions using violin plots (gray for test data, black for training data). (F) Comparison of predicted and actual gene expression distributions at time 2. Predicted distributions are shown as dashed lines, while real data distributions are shown as solid lines. (G) Classification of ancestral cell subgroups and forward trajectory predictions. Left: Two distinct cell subpopulations at time 0, identified via clustering. Right: Forward-predicted trajectories of these two subpopulations. (H) Normalized mean differences in gene expression between the two trajectory groups are plotted over time. Positive values indicate higher expression in group 1, while negative values indicate higher expression in group 2. Genes exhibiting a drop greater than 0.5 in normalized mean difference at specific time points are highlighted, indicating pronounced convergence in expression levels between the two trajectories. (I) Representative examples of gene expression convergence. Four genes are shown, with average gene expression forward trajectories plotted separately for each ancestral subpopulation.

To further validate PROFET, we applied it to a time-series scRNA-seq dataset of MCF10A cells undergoing EMT induced by TGF-β^13^. This dataset visually exhibits cell-state convergence—transitioning from two distinct subpopulations at day 0 to a single population by day 4 (Figure 4A). However, with only discrete time points available in scRNA-seq data, the complete trajectory and timing of this convergence remain unclear. We thus used PROFET to reconstruct the full trajectories of cell states by interpolating between the initial (day 0) and final (day 4) distributions, while withholding the intermediate time point (day 2) for validation (Figure 4B).

To validate the accuracy of our predictions, we again benchmarked PROFET against WOT and random coupling. Notably, PROFET – unlike the other two – accurately recovered the bimodal structure observed in the day 2 test data (Figure 4C). To quantify this observation, we evaluated prediction errors across a range of latent dimensions and distance metrics. PROFET consistently produced lower errors than both baseline methods under all tested conditions (Figure 4D; Supplementary Figure 6).

We then again applied an inverse PCA transformation to reconstruct continuous single-cell, single-gene trajectories for all genes in the EMT dataset. Analysis of two example genes, *DSP*^36^ and *SERINC2*^37^, showed that their trajectories converged from two distinct modes at day 0 into a single mode by day 4 (Figure 4E). Notably, PROFET also captured the intermediate bimodal structure at day 2, which had been withheld as test data (Figure 4F). Permutation tests revealed no statistically significant differences between the predicted and observed distributions on day 2 (*p* > 0.05), supporting the accuracy of PROFET (Methods). Complete results for all other genes are provided in Supplementary Figures 7–8 and Supplementary Table 2.

Unlike the mESC dataset, where trajectory divergence was observed, the EMT dataset reveals evidence of convergence in cellular states. To further explore this observation, we performed clustering analysis on the day 0 data and identified two ancestral subpopulations (Figure 4G left). By classifying each reconstructed trajectory based on its proximity to these ancestral clusters, we traced their respective differentiation paths toward a common final state (Figure 4G right).

To identify genes associated with the convergence process, we calculated the normalized difference in mean gene expression between the two trajectory groups, as described in the previous section. Genes whose expression differences declined by more than 0.5 —representing a reduction of over 50% from their maximal divergence—between day 0 and day 4 were considered strongly associated with convergence (Figure 4H). A key advantage of PROFET, which reconstructs continuous trajectories using force-matching (Methods), is its ability to precisely localize the timing of convergence. We observed that a major reduction in inter-trajectory expression differences occurred after day 2, as illustrated by four representative genes. Each of these genes exhibited a decline of over 50% in mean expression differences between the two sub-trajectories from day 2 to day 4, highlighting their involvement in the convergence process in the later phase of EMT (Figure 4I).

### Tracing Phenotypic Shifts Associated with Palbociclib Therapy in HR+ Breast Cancer

**Figure 5.**
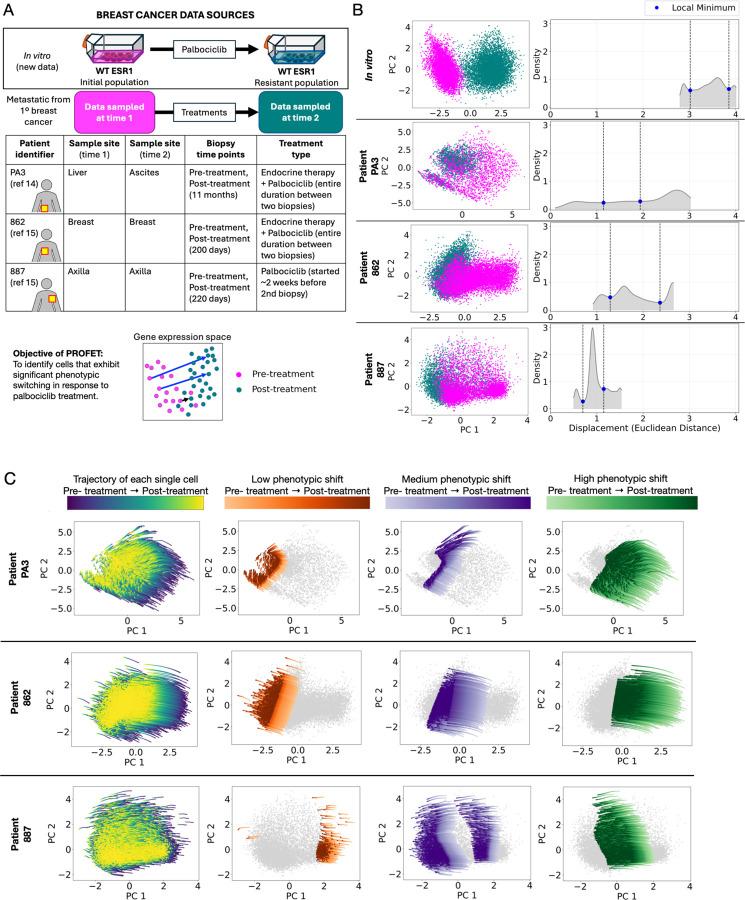
Reconstructed single-cell trajectories for palbociclib resistance. (A) Overview of the datasets analyzed (upper table): one in vitro cell line experiment and three patient-derived datasets—one from Luo et al. and two from Klughammer et al. The lower panel illustrates the objective of applying PROFET to these datasets: to identify single cells that undergo significant phenotypic switching in response to palbociclib treatment. (B) Each row represents one dataset. Left: PCA projection of scRNA-seq data, with pre-treatment cells in magenta and post-treatment cells in teal. Right: Kernel density estimates (KDE) of phenotypic shifts, defined as the Euclidean distances between initial and final cell states for each single-cell trajectory inferred by PROFET. C) Trajectories of resistance in HR+ breast cancer. Each row corresponds to one dataset. The first panel presents the full set of continuous single-cell trajectories reconstructed by PROFET. The subsequent three panels display subsets of trajectories grouped by their magnitudes of phenotypic shift, quantified as the Euclidean distances between initial and final cell states, as defined in (B).

We then sought to deploy PROFET to investigate heterogeneity in treatment response to palbociclib, a CDK4/6 inhibitor used in HR+ breast cancer that is known to induce variable treatment responses^16,38^. This analysis was performed across four datasets: one *in vitro* cell line model and three patient-derived samples. For the *in vitro* model, we generated a palbociclib-resistant line by treating MCF7 breast cancer cells with escalating doses of palbociclib over a 16-week period until stable resistance was established (Methods). Subsequently, scRNA-seq was performed on the parental and resistant cells (Figure 5A).

In parallel, we analyzed longitudinal scRNA-seq data from three HR+ metastatic breast cancer patients. The first patient (PA3) was part of the study by Luo et al^14^. Her initial biopsy was collected prior to palbociclib treatment, and two additional biopsies were both obtained 11 months after initiating palbociclib plus endocrine therapy. These latter samples were collected from ascitic fluid and confirmed disease progression; in our analysis, they were combined as a single post-treatment sample. The second (862) and third (887) patients were part of the study by Klughammer et al^15^. For patient 862, biopsies were collected before and after 200 days of treatment with endocrine therapy plus palbociclib; the second biopsy was performed for research purposes. Patient 887 initially responded to therapy following the first biopsy, but clinical progression was observed prior to the second biopsy, which was collected at day 220. The patient started receiving palbociclib approximately two weeks before the second biopsy (Figure 5A; Methods).

PROFET was developed and validated on datasets with lower dimensionality: a synthetic dataset with 26 genes, the mESC dataset with 100 genes, and the EMT dataset with 72 genes. In contrast, the four palbociclib-treated samples were assayed with genome-wide scRNA-seq, necessitating dimensionality reduction prior to analysis. However, due to high noise levels in these datasets, the first two principal components (PCs) captured only 5–10% of the total variance across samples, making direct PCA-based reduction insufficient.

To address this issue, we adopted a two-step dimensionality reduction strategy. First, we preselected 121 genes previously implicated in palbociclib response, including genes involved in cell cycle regulation, MYC and estrogen receptor (ER) signaling, growth factor pathways, the Hippo pathway, and inflammatory processes^39,40^ (Supplementary Table 3). In the second step, we applied PCA to the gene expression matrix restricted to this curated gene set. In the resulting PCA space, we observed that the *in vitro* dataset exhibited the most distinct separation between pre- and post-treatment samples while patient-derived datasets showed less clear separation, indicating heterogeneous responses to palbociclib (Figure 5B). To quantify these differences, we leveraged the continuous single-cell trajectories reconstructed by PROFET. For each cell, we computed the extent of phenotypic shift as the Euclidean distance between its inferred pre-treatment and post-treatment states in the gene expression space (Methods). As expected, the *in vitro* dataset exhibited the largest average shift with low variability, while the patient samples displayed broader, more heterogeneous distributions (Figure 5B): the coefficients of variation (CVs) were 0.10 for the *in vitro* data compared to 0.43, 0.30, and 0.26 for patients PA3, 862, and 887, respectively (Methods and Supplementary Table 4), confirming greater phenotypic variability in the clinical setting.

Based on the relatively homogeneous treatment response observed in the MCF7 *in vitro* dataset—specifically in genes relevant to palbociclib treatment—we used this dataset as a reference to dissect heterogeneity in resistant cell states in patient samples. We focused on identifying subpopulations of patient-derived cells that underwent substantial phenotypic shifts analogous to those observed *in vitro*. To achieve this, we examined the distributions of phenotypic shift magnitudes and identified three major modes using local minima (Methods and Figure 5B), allowing us to stratify cells in each patient sample into three subgroups: those marked by high, medium, and low phenotypic shifts. This classification, visualized in PCA space (Figure 5C), highlights the significance of PROFET not only in validating our hypothesis regarding heterogeneous treatment responses in patient data but also enabling the quantification of which specific cells exhibit substantial state transitions—an insight that is not readily apparent from unpaired scRNA-seq data alone. This single-cell level classification provides a framework for dissecting phenotypic heterogeneity in response to palbociclib and offers a powerful strategy for identifying candidate cell populations likely to undergo substantial phenotypic shifts in response to treatment.

### Trajectory-Based Dissection of Treatment Response Heterogeneity and Early Biomarker Discovery

**Figure 6.**
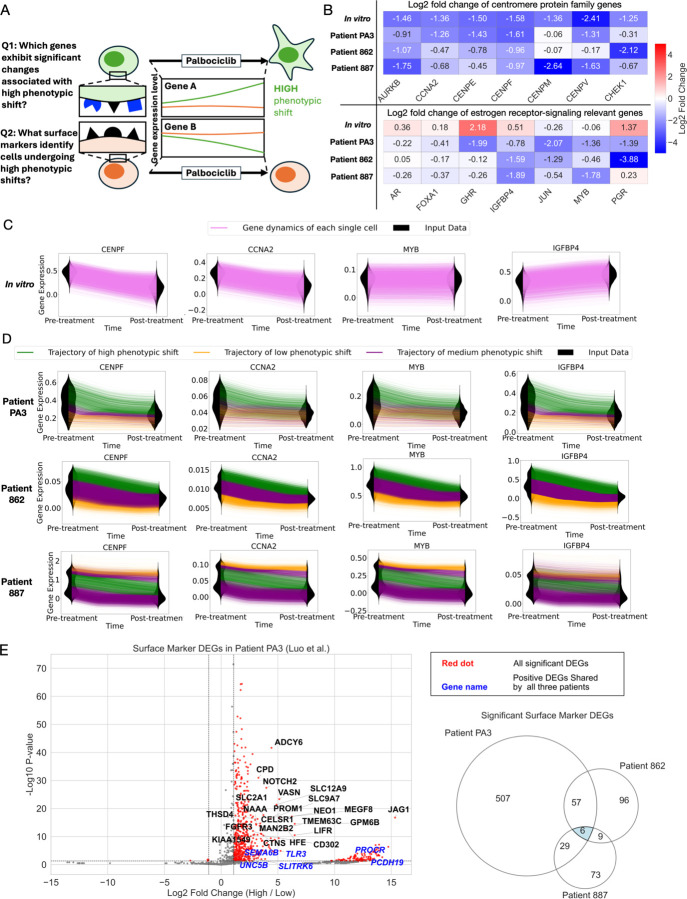
Downstream analysis of reconstructed single-cell trajectories in palbociclib resistance. (A) Schematic overview of the key biological questions addressed through downstream analyses of reconstructed single-cell trajectories that link pre-treatment and post-treatment states. (B) Differential gene expression analysis comparing post- versus pre-treatment time points for each dataset. Color indicates the direction and magnitude of log_2_ fold change (red: upregulated, blue: downregulated). (C) Representative examples of gene expression dynamics for individual cells in the *in vitro* dataset. Violin plots show observed gene expression levels used as input for trajectory inference; magenta lines represent predicted trajectories for each single cell. (D) Representative gene dynamics for individual cells across three patient datasets. Violin plots show real expression data; colored lines represent predicted trajectories from three subgroups of cells defined by low, medium, and high phenotypic shift levels, as described in Figure 5. (E) Differential expression analysis of surface marker genes in pre-treatment cells from patient PA3 (Luo et al.), comparing high versus low phenotypic shift subgroups. Red dots indicate significantly differentially expressed genes (p < 0.05, |log_2_ fold change| > 1.1). Blue-labeled genes represent significantly upregulated genes (positive log_2_ fold change) that are significantly upregulated in the other two patients (862 and 887) from Klughammer et al. The Venn diagram summarizes the overlap of differentially expressed surface markers across all three datasets.

Using these trajectory-based subgroups, we next aimed to identify genes undergoing the most significant changes within the high-shift subgroup, and to further pinpoint surface markers uniquely enriched in pre-treatment cells within this group (Figure 6A). To this end, we performed DEG analysis for all four datasets by comparing average gene expression levels between post-treatment samples to those from pre-treatment samples (Methods). This analysis revealed that most cell cycle-related genes were consistently downregulated following treatment across all datasets (Figure 6B). In particular, several members of the centromere protein family—*CENPE*, *CENPF*, *CENPM*, and *CENPV*—were significantly downregulated in the MCF7 cell line following palbociclib treatment (-2.41 < log2 fold change < −1.36; p < 5e–10), and showed similar downregulation trends in the patient samples (Figure 6B). Complete DEG results are provided in Supplementary Table 5.

In contrast, we observed divergent expression patterns of estrogen receptor (ER) signaling– relevant genes between the cell line and patient samples PA3 and 862, both of whom received combination therapy with palbociclib and endocrine treatment (Figure 5A). In the cell line data, *AR*, *FOXA1*, *GHR*, *IGFBP4*, and *PGR* were upregulated, whereas these same genes were downregulated in the patient samples. *JUN* and *MYB* were downregulated in both the cell line and patient datasets, with more pronounced downregulation observed in the patient samples (Figure 6B for the full fold change heatmap and tables). These results indicate that the profound downregulation of ER signaling–relevant genes is specific to the patient samples, while centromere genes were consistently downregulated in both the cell line and patient data. The well-established effects of these treatments—palbociclib primarily inhibiting cell cycle–related genes^16,41^, and endocrine therapy downregulating ER signaling–related genes^42,43^— validate our observed trajectories, aligned with the treatment regimens applied to each sample (Figure 5A).

Since this DEG analysis reflects only population-average changes, it does not resolve the specific subgroups within each tumor that exhibit these phenotypic changes induced by the combination therapy. To further dissect this treatment response heterogeneity, we used PROFET to reconstruct gene expression dynamics at the single-cell, single-gene level. Here, we present results for two representative cell cycle–related genes (*CENPF* and *CCNA2*) and two ER signaling–related genes (*MYB* and *IGFBP4*) (Figure 6C–D). Complete results for additional genes are provided in Supplementary Figures 9–12. In the *in vitro* dataset, *CENPF* and *CCNA2* showed uniform downregulation, *MYB* expression remained consistently steady, and *IGFBP4* was uniformly upregulated (Figure 6C). In contrast, the patient datasets displayed substantial variability, with the high phenotypic shift subgroup exhibiting more pronounced downregulation of all four genes compared to the low-shift subgroup (Figure 6D).

We then sought to identify early markers of the subpopulation of cells undergoing high phenotypic shifts in response to palbociclib resistance. To identify such markers, we performed DEG analysis of surface marker genes between the high and low phenotypic shift groups at the pre-treatment time point^37^. This analysis revealed six genes— *UNC5B*, *TLR3*, *PCDH19*, *PROCR*, *SLITRK6*, and *SEMA6B* —that were consistently enriched in the high-shift subgroup across all three patient datasets (Figure 6E).

Among these genes, *UNC5B* has been reported to be significantly upregulated in breast cancer and is correlated with poor overall survival in breast cancer patients^44^. Activation of *TLR3* has been shown to increase cancer stem cell (CSC) markers, which are associated with breast cancer progression^45^. *PROCR* is known to activate several signaling pathways in breast cancer cells, including ERK, PI3K–Akt–mTOR, and RhoA–ROCK–p38 pathways^46^. While *PCDH19* has not previously been implicated in metastatic breast cancer, it belongs to the protocadherin family; notably, another member of this family, *PCDHA1*, has been associated with poor prognosis and immune cell infiltration in breast cancer^47^. Although *SEMA6B* and *SLITRK6* have not yet been linked to breast cancer, *SLITRK6* has been identified as a novel biomarker in hepatocellular carcinoma^48^, while *SEMA6B* has been associated with poor prognosis and an immunosuppressive tumor microenvironment in colorectal cancer^49^—suggesting, based on our findings, that both genes may serve as candidate markers of disease progression in HR+ metastatic breast cancer.

Taken together, these studies demonstrate that overexpression of these genes is strongly associated with more aggressive breast cancer and poorer prognosis. Building on this evidence, our findings—based on reconstructed phenotypic trajectories before and after treatment—further suggest that these genes may serve novel roles as surface markers capable of predicting which pre-treatment cells are likely to undergo profound phenotypic changes in response to palbociclib and endocrine therapy. Notably, in both the *in vitro* dataset and patient samples PA3^14^ and 887^15^, where treatment resistance developed, these markers show promise for the early identification of treatment-resistant cells, paving the way for more targeted and proactive therapeutic strategies.

## Discussion

Here, we introduce PROFET, a computational framework for reconstructing continuous, nonlinear single-cell trajectories from limited, discrete scRNA-seq time points. PROFET addresses a key limitation of existing methods—their inability to model time-inhomogeneous (non-autonomous) cell dynamics driven by external perturbations such as drug treatment. Traditional OT methods compute only static couplings and cannot model intermediate nonlinear transitions, while CNF models require predefined source distributions that may bias the learned dynamics. In contrast, PROFET learns transport dynamics directly between observed cellular states without assuming prior geometries or distributions. It operates in two steps: first, it performs interpolation using a Lipschitz-regularized gradient flow; second, it trains a neural network to learn a global, continuous, time-dependent velocity field via a force-matching strategy. While methods like *Dynamo*^50^ also account for time-inhomogeneous dynamics through explicit dynamical equations, they rely on metabolically labeled or otherwise informative datasets to estimate parameters. PROFET offers a fully data-driven alternative, learning the vector field directly from conventional scRNA-seq data—providing broad applicability without the need for specialized experimental protocols.

Given PROFET’s strength in analyzing systems with phenotypic heterogeneity, we applied it to study variability in response to palbociclib, a CDK4/6 inhibitor used in HR+ breast cancer. In an *in vitro* breast cancer cell line treated with escalating doses of palbociclib, we observed a relatively uniform response characterized by downregulation of cell cycle genes and eventual drug resistance. In contrast, patient samples treated with combined palbociclib and endocrine therapy exhibited greater heterogeneity, with some cells showing strong downregulation of both cell cycle and ER signaling genes, while others remained largely unresponsive. Using PROFET to reconstruct single-cell trajectories, we identified a subset of pre-treatment cells that later underwent pronounced phenotypic shifts. Differential expression analysis revealed surface markers—including *UNC5B*, *TLR3*, *PCDH19*, *PROCR*, *SEMA6B*, and *SLITRK6*—that were enriched in this high-shift group, suggesting their potential as early predictors of treatment-associated transitions. Notably, both the *in vitro* model and two patient samples (PA3 and 887) showed signs of treatment resistance or disease progression, implicating these markers in the emergence of resistance. These findings point to the utility of these surface proteins not only for early response prediction in HR+ breast cancer but also as promising targets for ADCs, particularly *SLITRK6*, which is currently under clinical evaluation in ADC-based therapies^41^.

Despite outperforming existing trajectory analysis tools and demonstrating strong utility in biological applications, PROFET has limitations. First, PROFET does not model population dynamics, such as proliferation or apoptosis, and thus cannot distinguish phenotypic shifts from selective expansion or contraction of cell populations. Integrating lineage tracing and branching process models may enable joint inference of both trajectories and population changes. Second, PROFET currently relies on single-modality input (scRNA-seq), limiting its ability to capture the full spectrum of cellular identity shaped by multimodal factors like chromatin accessibility and spatial context. Extending the framework to integrate multi-omic and spatial data will enhance biological interpretability and support more mechanistic trajectory inference.

In summary, PROFET offers a biologically grounded framework for reconstructing continuous, time-inhomogeneous, and nonlinear single-cell trajectories from discrete time-series scRNA-seq data. This characteristic makes it particularly valuable for analyzing systems with phenotypic heterogeneity, especially in the context of cell state changes and treatment response. By enabling high-resolution inference of gene expression dynamics and fate transitions, PROFET offers a powerful approach for uncovering hidden heterogeneity, tracing phenotypic plasticity, and informing time-sensitive interventions in both developmental and disease contexts.

## Methods

### Generative Particles Algorithm

#### Gradient flow simulation with Generative Particles Algorithm (GPA)

We simulate data-driven trajectories using the Generative Particles Algorithm (GPA)^1^, a deterministic method that iteratively transports particles from a source distribution toward a target. In our setting, each particle represents a cell’s gene expression profile in reduced-dimensional space. Unlike normalizing flow-based approaches that require structural assumptions on the base or target distribution (e.g., Gaussianity or manifold support), GPA uses Lipschitz-regularized gradient flows to interpolate between arbitrary distributions—including empirical, discrete, or irregular ones—in a fully agnostic and mathematically justified manner. This provable flexibility^2^ is essential in single-cell analysis, where the underlying data structure is unknown.

At each time step, GPA estimates a potential function based on the current particle configuration and computes an update direction via its gradient. This induces an ODE-based dynamics:

(1) $$\begin{array}{cccc} \frac{dX_{t}}{dt}=v_{t}\left(X_{t}\right), & \text{with} & v_{t}\left(x\right)=-\nabla \phi_{t}^{L,*}\left(x\right), & X_{0}∼\rho_{0}, \end{array}$$

where $\phi_{t}^{L,*}$ is the time-dependent potential learned from the data. The underlying Wasserstein gradient flow formulation and the variational structure of the Lipschitz-regularized KL divergence are provided in detail in Supplementary Note 1.

To solve this ODE numerically, GPA uses a forward Euler scheme over $n_{T}$ steps of size Δt:

(2) $$\begin{array}{cc} Y_{n+1}^{i}=Y_{n}^{i}-\Delta t\nabla \phi_{n}^{L,}\left(Y_{n}^{i}\right), & \phi_{t}^{L,*}=\arg\underset{\phi \in {\text{Lip}}_{L}\left({\mathbb{R}}^{d}\right)}{\max}\left\{\frac{1}{N}\sum_{i}\phi \left(Y_{n}^{i}\right)-\frac{1}{M}\sum_{j}f^{*}\left(\phi \left(X^{j}\right)\right)\right\}, \end{array}$$

starting from samples ${\left\{Y_{0}^{i}\right\}}_{i=1}^{N}∼\rho_{0}$. The inner optimization approximates the KL divergence between the current particle distribution and the target distribution using the variational representation of f-divergence, where $f\left(x\right)=x\log x$ and $f^{*}$ denotes its Legendre transform.

At each iteration, GPA updates the particle positions and records the time-labeled velocities

(3) $$\begin{array}{ccc} \left(x,t,v\right)=\left(Y_{n}^{i},n\Delta t,-\nabla \phi_{n}^{L,*}\left(Y_{n}^{i}\right)\right), & i=1,\ldots ,N; & n=0,\ldots ,n_{T}-1. \end{array}$$

The evolution naturally slows as the particle distribution approaches the target, which is quantified by the decay of the expected kinetic energy $E\left[{\left\|\nabla \phi_{n}^{L,*}\left(Y_{n}^{i}\right)\right\|}^{2}\right]\to 0$.

#### Role of Lipschitz regularization in empirical gradient flow modeling

Lipschitz regularization on the KL divergence serves *three critical purposes* in modeling empirical gradient flows:

1. **Theoretical well-posedness:** Empirical distributions—being finite samples from unknown distributions on ${\mathbb{R}}^{d}$ —are generally not absolutely continuous with respect to each other. Consequently, the KL divergence between them may become ill-defined or divergent. The Lipschitz-regularized KL divergence resolves this issue by defining a well-posed variational objective even when absolute continuity fails. This ensures that the first variation and corresponding flow velocity remain well-defined. See Supplementary Note 2 for a detailed discussion of this issue.
2. **Velocity control:** Lipschitz continuity ensures that the velocity field induced by the flow has a finite speed bound, specifically bounded by the Lipschitz constant L. In the limit as L→∞, the velocity field recovers the unregularized KL gradient flow (e.g., the Ornstein–Uhlenbeck process). In practice, when simulating empirical distributions with compact support, a reasonably large but finite L suffices to approximate the KL-induced dynamics well. For further illustrations and examples, see Supplementary Note 3.
3. **Numerical stability and stiffness control:** Bounding the velocity field by a finite Lipschitz constant L ensures that the transport speed is controlled, enabling stable time integration under the Courant–Friedrichs–Lewy (CFL) condition for Euler-type methods (2). However, larger L values allow the velocity to vary more rapidly across space, which can lead to stiff dynamics—requiring extremely small Δt to avoid instability. This stiffness–stability trade-off motivates the use of moderate values of L that are large enough to capture meaningful dynamical behavior (e.g., convergence rate) but small enough to maintain stable, efficient simulations at practical step sizes. In effect, L acts as a dial that balances dynamical accuracy and numerical feasibility in GPA.

In our method, we set L=1 to ensure numerical stability across diverse datasets, providing a universally robust baseline. The step size Δt is then adjusted per dataset to accommodate variations in scale and complexity.

#### Handling high-dimensionality via latent-space modeling

Gene expression datasets are intrinsically high-dimensional, with each cell represented by expression levels across thousands of genes. To make learning tractable while preserving meaningful structure, we apply Principal Component Analysis (PCA) to project the data into a lower-dimensional latent space, where GPA is used to model the dynamics. For downstream analysis, we reconstruct the trajectories in the original space using the inverse PCA transform.

Across our 5 datasets — with ambient dimensions D=26,100,72,117,116,115,115 corresponding to the number of genes in synthetic data, mESC data, EMT data, breast cancer cell line, patient PA3, patient 862, and patient 887, respectively — we reduce the dimension of each of them to d=2,8,32,…,D and observe that GPA consistently performs well up to moderate latent dimensions (e.g., d≤32), capturing temporal structure without overfitting or numerical instability.

To justify latent modeling of high-dimensional biological applications, we provide a theoretical guarantee based on the Lipschitz-regularized divergence in Supplementary Note 4.

#### Stopping time for GPA

To determine an appropriate simulation length for the Generative Particles Algorithm (GPA), we empirically identify the stopping time $n_{T}$ based on validation against intermediate snapshots not used during training. Specifically, we evaluate how well the simulated trajectories match these held-out snapshots by computing the Sinkhorn divergence^3^ between the generated particle distribution at intermediate timepoints and the true empirical distribution. This provides an objective criterion for selecting $n_{T}$ that best captures the temporal structure of the data. During training, we monitor the Lipschitz-regularized KL divergence $D_{f}^{{\text{Lip}}_{L}}$ (with f=KL and L=1), and find that a threshold value of $D_{f}^{{\text{Lip}}_{L}}<0.05$ reliably corresponds to optimal alignment with the held-out distributions. This threshold provides a practical and computationally efficient criterion for early stopping that balances convergence and overfitting to the endpoint distributions.

#### Extending GPA to capture nonlinear temporal dynamics

While GPA accurately simulates local transport between pairs of gene expression snapshots, many biological processes—such as drug response or lineage bifurcation—exhibit temporally nonlinear or branched dynamics that cannot be represented by a single global trajectory. To address this, we partition the total time horizon $\left[0,T\right]$ into subintervals and apply GPA independently within each, using the corresponding pair of snapshots.

This strategy yields a sequence of locally consistent gradient flows that adapt to complex temporal structures while preserving interpretability through KL-consistent dynamics. These local simulations serve as the foundation for constructing a unified, continuous-time model in the next stage of our framework.

### Particle-based Reconstruction Of generative Force-matched Expression Trajectories

#### Force-matching for learning a unified velocity field

To aggregate the locally simulated gradient flows from multiple GPA subintervals into a global dynamic model, we adopt a force-matching approach^4^. Classically used to learn coarse-grained force fields from trajectory data in molecular simulations^5–9^, force-matching is here extended to the data-driven regime: instead of relying on predefined physical models, we use GPA-inferred particle trajectories—obtained separately on each subinterval—to supervise the learning of a continuous-time Eulerian velocity field. Importantly, we require a globally defined velocity field over the entire time horizon to support downstream analyses—such as tracing fate bifurcations (Figure 3E), identifying genes with temporally localized divergence or convergence (Figures 3F–G, 4H–I), and quantifying phenotypic shifts in patient-derived cells (Figure 5C)—all of which rely on continuous integration from arbitrary time points.

We define a neural velocity field $v_{\theta }\left(x,s\right)$ over space and time and train it by minimizing the deviation between the learned velocity and the GPA-estimated transport velocity at randomly sampled time points across all particles:

(4) $$\underset{\theta }{\min}\;E_{\left(x,s\right)}\left[{\left|v_{\theta }\left(x,s\right)+\nabla \phi_{s}^{L,*}\left(x\right)\right|}^{2}\right].$$

Here, $\phi_{s}^{L,*}$ is the potential estimated from GPA on the subinterval that covers time s. To ensure temporal consistency across subintervals, the physical time variable $s\in \left[0,T\right]$ is defined by proportionally aligning each subinterval’s local GPA simulation time $t\in \left[0,n_{T}^{\left(i\right)}\Delta t^{\left(i\right)}\right]$ to a shared physical time scale. All subintervals are reparameterized relative to the longest GPA simulation horizon $\max_{i}n_{T}^{\left(i\right)}\Delta t^{\left(i\right)}$, allowing the learned velocity field $v_{\theta }\left(x,s\right)$ to be defined on a unified time domain.

The resulting training process unifies the velocity information from all subintervals and fits a smooth vector field that generalizes beyond the discrete particle paths. This formulation distills the localized Lagrangian dynamics simulated by GPA into a global Eulerian flow model, enabling continuous-time interpolation and extrapolation across space and time.

Unlike conventional flow-matching methods^10^, our formulation uses empirical velocity targets derived from simulated particle dynamics. This approach enables the learned field to capture nonlinear biological transitions such as convergence, divergence, or bifurcations in cell states. A detailed comparison between this approach and score-based generative models using flow-matching is provided in Supplementary Note 5.

#### Overview of the two-step framework

Our method infers continuous-time gene expression dynamics from a small number of time-stamped single-cell snapshots by combining particle-based simulation and neural flow modeling in two successive stages.

In the first stage, we apply GPA independently to each consecutive pair of gene expression distributions $\left\{P_{t_{i}},P_{t_{i+1}}\right\}$. GPA simulates discrete-time particle trajectories that approximate the Wasserstein gradient flow minimizing the Lipschitz-regularized KL divergence. At each iteration, GPA solves the optimization problem to estimate the dual potential $\phi_{n}^{L,*}$ and updates particle positions via forward Euler integration as in Eq. (2). This process yields a time series of particle states and their corresponding velocity vectors.

In the second stage, we train a time-dependent velocity field $v_{\theta }\left(x,s\right)$ parameterized by a neural network to match the simulated velocities from GPA. This is done by minimizing the squared loss between predicted and simulated velocities over all time-labeled particle states. The result is a continuous vector field that generalizes beyond particle locations and interpolates temporal dynamics across the full time horizon.

#### Model architecture and training algorithm

To estimate the potential $\phi_{n}^{L,*}$, we use a fully connected feedforward network (MLP) with 3 hidden layers of 128 units each and ReLU activations, followed by a projection layer to enforce the 1-Lipschitz constraint via spectral normalization. The velocity field $v_{\theta }\left(x,s\right)$ is modeled as another MLP with 4 hidden layers of 64 units and Tanh activations, taking both position 𝑥 and time 𝑠 as inputs. Spectral normalization is applied to all layers to ensure Lipschitz continuity, which improves numerical stability and supports temporally smooth interpolation across time points. These architectures were selected based on preliminary experiments indicating reliable scalability to high-dimensional settings—up to ∼100 dimensions in the GPA step and ∼32 dimensions in the force-matching step. We further explored various hyperparameters including learning rates, iteration steps, network size, and regularization thresholds (Supplementary Figure 13A–D). For the velocity field network, the chosen width and depth provided sufficient expressivity for modeling nonlinear dynamics while avoiding underfitting issues observed in larger architectures, which were prone to optimization instability and diminished learning efficacy.

The overall procedure is summarized in Algorithm 1 and is illustrated in Figure 1.


$$\bar{\underline{\begin{array}{l} \underline{Algorithm\;1\;\text{Particle-based}\;\text{Reconstruction}\;\text{Of}\;\text{generative}\;\text{Force-matched}\;\text{Expression}\;\text{Trajectories}\;\left(\text{PROFET}\right)}\\ Require:\;\text{Time-stamped}\;\text{gene}\;\text{expression}\;\text{snapshots}\;{\left\{P_{t}\right\}}_{t=0}^{T}\\ Ensure:\;\text{Learned}\;\text{continuous-time}\;\text{velocity}\;\text{field}\;v_{\theta }\left(x,s\right)\\ \;\;\;\;\;\;Step\;1:\;Trajectory\;Inference\;via\;GPA\\ \;\;1:\;for\;\text{each}\;\text{consecutive}\;\text{pair}\;\left\{P_{t_{i}},P_{t_{i+1}}\right\}\;do\\ \;\;2:\;\;\;\;\;\text{Initialize}\;\text{particles}\;{\left\{Y_{0}^{i}\right\}}_{i=1}^{N}∼P_{t_{i}},{\left\{X^{j}\right\}}_{j=1}^{M}∼P_{t_{i+1}},\\ \;\;3:\;\;\;\;\;for\;n=0\;\text{to}\;n_{T}-1\;do\\ \;\;4:\;\;\;\;\;\;\;\;\;\text{Solve}\;\text{dual}\;\text{potential}\;\phi_{t}^{L,*}=\arg\;\max_{\phi \in Lip_{L}}\left\{\frac{1}{N}\sum_{i}\phi \left(Y_{n}^{i}\right)-\frac{1}{M}\sum_{j}f^{*}\left(\phi \left(X^{j}\right)\right)\right\}\\ \;\;5:\;\;\;\;\;\;\;\;\;\text{Update}\;\text{particles}:Y_{n+1}^{i}\leftarrow Y_{n}^{i}-\Delta t\nabla \phi_{n}^{L,*}\left(Y_{n}^{i}\right)\\ \;\;6:\;\;\;\;\;end\;for\\ \;\;7:\;\;\;\;\;\text{Collect}\;\text{supervised}\;\text{samples:}\;\left(x,s,v\right)\leftarrow \left(Y_{n}^{i},n\Delta t,-\nabla \phi_{n}^{L,*}\left(Y_{n}^{i}\right)\right)\\ \;\;8:\;end\;for\\ \;\;\;\;\;\;Step\;2:\;Flow\;Model\;Training\\ \;\;9:\;\text{Initialize}\;\text{neural}\;\text{network}\;v_{\theta }\left(x,s\right)\\ \;10:\;while\;\text{not}\;\text{converged}\;do\\ \;11:\;\;\;\;\;\text{Sample}\;\text{batch}\left\{\left(x_{k},s_{k},v_{k}\right)\right\}\;\text{from}\;\text{all}\;\text{collected}\;\text{trajectories}\\ \;12:\;\;\;\;\;\text{Compute}\;\text{flow}\;\text{matching}\;\text{loss:}\;𝓛\left(\theta \right)=\frac{1}{K}\sum_{k}{\left\|v_{\theta }\left(x_{k},s_{k}\right)-v_{k}\right\|}^{2}\\ \;13:\;\;\;\;\;\text{Update}\;\theta \;\text{via}\;\text{gradient}\;\text{descent}\\ \;14:\;end\;while\\ \;15:\;return\;v_{\theta }\left(x,s\right) \end{array}}}$$

### Downstream Analysis Using Neural GPA

To extract biologically meaningful insights from the PROFET-inferred trajectories, we performed three main downstream analyses. First, we reconstructed the dynamic expression profiles of all genes provided in the input data for each individual cell. This step enables gene-level resolution of temporal behavior along each cell’s trajectory. Second, although PROFET outputs trajectories at the single-cell level, we further enhanced interpretability by grouping these trajectories into subpopulations corresponding to distinct cellular behaviors or phenotypic states.

Finally, by integrating trajectory classification with single-gene dynamics, we extended conventional differential gene expression analysis—typically performed at discrete time points— to a trajectory-aware framework. This approach allows us to identify key gene markers associated with divergent fate transitions, thereby uncovering regulators that may drive phenotypic bifurcations over time.

#### Reconstruction and evaluation of single-gene dynamics

To ensure stability and computational efficiency, PROFET was applied to reconstruct cell state trajectories in a reduced-dimensional space. Benchmarking against WOT and a null model demonstrated reliable performance with dimensions up to 30 (Supplementary Figures 1 and 6). Accordingly, we first performed PCA on the gene expression matrix compiled across all input time points to reduce dimensionality. The resulting principal components were then used to represent the data in a lower-dimensional space for trajectory reconstruction. To recover gene-level information, the reconstructed trajectories were mapped back to the original gene expression space using the inverse PCA transformation^11^, thereby yielding predicted gene expression profiles for each individual cell.

To evaluate the accuracy of these reconstructions, we compared the predicted gene expression levels at held-out test time points with the actual expression data. We assessed whether the overall distribution of predicted cell states was statistically indistinguishable from that of the observed test data. We conducted permutation tests using three distributional distance metrics: Total Variation (TV) distance, Kullback-Leibler (KL) divergence, and Sinkhorn divergence^12^. For each metric, we first computed the observed distance between the predicted and observed distributions. Next, we pooled the two datasets and randomly permuted the group labels, recomputing the distance between two groups of equal size for each permutation. This process was repeated 1,000 times to generate a null distribution of distances under the hypothesis that both datasets originate from the same underlying distribution. A one-sided p-value was calculated as the fraction of permutations in which the permuted distance exceeded the observed distance. This approach enabled us to quantitatively assess the alignment between predicted and actual cell state distributions over time. Results for the mESC and EMT datasets, including all p-values, are provided in Supplementary Table 1 and Table 2, respectively. Finally, we summarized the number of genes for which predicted and observed distributions were statistically indistinguishable $\left(p<0.05\right)$, based on one, two, or all three metrics (Supplementary Figure 14).

#### Classification of sub-trajectories

In this study, we implemented two complementary strategies to classify predicted single-cell trajectories, providing distinct biological interpretations of cell fate dynamics. The first strategy involves classification based on cell subpopulations defined at a specific time point. These subpopulations can be identified using unsupervised clustering alone or in combination with supervised annotations such as known cell types or marker-based labels.

In our analysis, we adopted an unsupervised approach using k-means clustering^13^ to define subpopulations from the observed data. Specifically, we first reduced the dimensionality of gene expression matrices using PCA and then applied the k-means algorithm to the cell states at a chosen reference time point (e.g., the final day for mESC or the initial day for EMT). We used the kmeans.fit function from the scikit-learn Python package^14^ to train the k-means model on the PCA-transformed gene expression profiles, which partitions the data into k clusters by minimizing within-cluster variance and learns the associated cluster centroids in the reduced feature space. The optimal number of clusters $\left(k\right)$ was determined using the silhouette score (Supplementary Figure 15A).

Once the k-means model was trained on the observed cells, we then used it to assign predicted cell states from PROFET to the learned subpopulations. This was done using the kmeans.predict function, which computes the nearest centroid in Euclidean space for each predicted cell state at the reference time point and assigns it the corresponding cluster label. This strategy enables trajectory classification by linking each predicted cell to a biologically meaningful subpopulation derived from the data. For instance, in the mESC dataset, this allowed us to identify sub-trajectories that diverge into distinct terminal fates; in the EMT dataset, it enabled classification of sub-trajectories based on their inferred origins.

The second strategy classifies trajectories based on the extent of cell state transitions between two selected time points. For each trajectory, we computed the Euclidean distance between its predicted cell states at the early and late stages, using this distance as a quantitative proxy for the magnitude of phenotypic change or differentiation. Applying this procedure across all trajectories yielded a distribution of transition distances, which reflects the heterogeneity in dynamic behavior among cells. To stratify trajectories, we categorized them into low, intermediate, or high transition groups based on thresholds identified from local minima in the histogram of transition distances. This data-driven thresholding approach enables separation of distinct transition regimes. Unlike the first strategy, which aligns trajectories with known subpopulations at a fixed reference time point, this approach captures cell state transitions and highlights trajectories undergoing varying degrees of phenotypic change over time.

#### Differential analysis across sub-trajectories

By integrating the reconstructed gene expression dynamics along each predicted trajectory with the sub-trajectory classifications, we conducted two complementary types of differential analysis to identify gene expression differences associated with divergent cell fates. The first approach focused on quantifying temporal divergence in mean gene expression between subpopulations. For each gene, we computed the mean expression within each sub-trajectory group at every time point, then calculated the difference in means between the two groups. These differences were subsequently normalized by the maximum observed value across all time points, producing a relative divergence profile. This method allowed us to pinpoint when specific genes began to diverge between subpopulations, thereby highlighting key temporal features of gene regulation along distinct cell fates.

The second approach involved standard differential gene expression (DGE) analysis^15^ at selected time points to identify gene markers that distinguish sub-trajectory groups. For each gene, we calculated the $\log_{2}$ fold change in expression between groups and assessed statistical significance using either a two-sample *t*-test. These comparisons were performed at individual time points using sets of cells assigned to different sub-trajectories. By interpreting the results within the framework of sub-trajectory definitions—such as the degree of phenotypic transition or final cell identity—we were able to identify gene expression features associated with distinct developmental paths or treatment responses.

### Synthetic Data for EMT Modeling

We generated a synthetic dataset mimicking an experimental setup wherein a population of epithelial cells is treated with an inducer of EMT such as TGF-β for a fixed duration of time. For this purpose, we started with a previously characterized 26-node gene regulatory network that is known to establish gene expression patterns characteristic of epithelial and mesenchymal cells and to govern the transition between these phenotypic states^16,17^. The gene regulatory network is described by a connection matrix J whose elements represent the regulatory interactions between genes:

(5) $$J_{ij}=\left\{\begin{array}{c} +1\\ \begin{array}{cc} -1 & \text{if} \end{array}\\ 0 \end{array}\right.\begin{array}{l} \text{gene}\;j\;\text{activates}\;\text{gene}\;i\\ \text{gene}\;j\;\text{inhibits}\;\text{gene}\;i\\ \text{gene}\;j\;\text{does}\;\text{not}\;\text{regulate}\;\text{gene}\;i \end{array}$$

#### SDE simulation of EMT gene regulatory network dynamics

Given the connection matrix J, we can write a system of stochastic differential equations to describe the dynamics of the EMT gene expression vector $x\equiv \left(x_{i}\right),1\leq i\leq 26$:

(6) $$\frac{dx_{i}}{dt}=g_{i}\left(\prod_{j,J_{ij}\neq 0}H^{S}\left(x_{j},\lambda_{ij},\Theta_{ij},n_{ij}\right)\right)-k_{i}x_{i}+Dx_{i}\eta_{i}\left(t\right)$$

Here, $g_{i}$ and $k_{i}$ are the production and degradation rates for gene i, respectively. The term $Dx_{i}\eta_{i}\left(t\right)$ corresponds to multiplicative noise where D=1 is the noise magnitude and $\eta_{i}\left(t\right)$ is a delta correlated noise term with zero mean and unit variance. $H^{S}$ is the shifted Hill function:

(7) $$H^{S}\left(x_{j},\lambda_{ij},\Theta_{ij},n_{ij}\right)=\lambda_{ij}+\left(1-\lambda_{ij}\right)\left(\frac{1}{1+{\left(\frac{x_{j}}{\Theta_{ij}}\right)}^{n_{ij}}}\right)$$

The parameters $\lambda_{ij}$, $\Theta_{ij}$, and $n_{ij}$ together characterize the nature of the regulation of gene i by gene j:

1. $\lambda_{ij}$ is the maximum fold change in the production rate of gene i that gene j can cause. Thus, $\lambda_{ij}>1$ if $J_{ij}=+1$ and $0<\lambda_{ij}<1$ if $J_{ij}=-1$.
2. $\Theta_{ij}$ is the threshold parameter: the regulatory edge form j to i is active if $x_{j}>\Theta_{ij}$. The regulatory interaction is very weak otherwise.
3. $n_{ij}$ is the Hill coefficient: $H^{S}$ varies more steeply with $x_{j}$ for higher values of $n_{ij}$.

Given a parameter set $\left(\left\{g_{i}\right\},\left\{k_{i}\right\},\left\{\lambda_{ij}\right\},\left\{\Theta_{ij}\right\},\left\{n_{ij}\right\}\right)$ and the initial condition $x_{0}$, Eq. (6) can be solved using the Euler-Maruyama method^18^.

### Dataset

#### Preprocessing of mESC data

The dataset analyzed consists of single-cell RNA-sequencing data from mouse embryonic stem cells collected at five time points: 0, 12, 24, 48, and 72 hours, comprising a total of 456 cells^19,20^. To preprocess the data, we applied a log-transformation to the raw read counts, replacing each value x with log(1+x). Following the same gene selection strategy as in the original study^19^, we calculated the variance of each gene across all cells and selected the top 100 genes with the highest variance for downstream temporal analysis.

#### Preprocessing of EMT data induced by TGF-β

The single-cell RNA-sequencing dataset analyzed in this study was obtained from a previous publication on TGF-β-induced epithelial-to-mesenchymal transition (EMT)^21^. We utilized the processed gene expression matrix provided by the authors; raw sequencing reads are available through the NCBI Sequence Read Archive (BioProject accession PRJNA698642)^22^. For dimensionality reduction, we selected a subset of 72 genes that overlap with a previously established list of 76 genes known to be strongly associated with the EMT gene expression signature^23,24^.

#### Breast cancer cell line generation and scRNA-seq library preparation

MCF7 cells engineered to express a doxycycline (DOX)-inducible-hemagglutinin (HA)-tagged Y537S mutant estrogen receptor (ER) ^25^ were cultured in DMEM containing 10% fetal bovine serum (FBS), 10 µg/ml penicillin-streptomycin-glutamine (PSG), and 500 µg/ml of geneticin (full medium). Cells were maintained either in the absence of DOX to allow expression of wild-type ER (ER-WT), or in the presence of 1 µg/ml DOX to induce expression of the Y537S mutant ER (ER-mutant). To establish ER-WT and ER-mutant cell lines resistant to palbociclib (PalboR), MCF7 cells treated with or without DOX were exposed to increasing concentrations of the drugs (from 50 nM to 1 µM). Cells were considered resistant once they restored the ability to proliferate in the presence of 1 µM palbociclib. Resistant cells were subsequently maintained in full medium supplemented with 1 µM palbociclib, with or without DOX. As a control, ER-WT and ER-mutant MCF7 cells were long-term cultured with the vehicle of the drugs (0.01% dimethyl sulfoxide, DMSO). Cells were authenticated by short tandem repeat (STR) profiling (Bio-Synthesis, USA) and routinely tested for mycoplasma contamination using the MycoAlert^®^ Mycoplasma Detection Kit (Lonza), following the manufacturer’s instructions. Parental ER-WT MCF7 cells, along with ER-WT and ER-mutant MCF7 cells resistant to palbociclib, or long-term cultured with the drugs’ vehicle, were analyzed by scRNA-seq. For each condition, we processed cell suspensions with a cell concentration optimized to recover approximately 8,000 transcriptomes. scRNA-seq libraries were then generated at the Translational Immunogenomics Lab, Dana-Farber Cancer Institute, following the 10x Genomics Chromium Next GEM Single Cell 3’ v3.1 protocol. The quality of the libraries was evaluated by Bioanalyzer using the High Sensitivity DNA kit (Agilent). Samples were sequenced by Illumina HiSeq or NovaSeq6000 (Paired-End 150 bp sequencing) at Novogene Corporation Inc.

#### Preprocessing of breast cancer cell line data

Samples were aligned with 10x Genomics Cell Ranger (v3.1.0) pipeline^26^ with the cellranger-count command with parameter ‘expect-cells=8000’ to the reference genome refdata-cellranger-GRCh38–3.0.0 provided by 10x Genomics. Filtered feature barcode matrices were imported into R using the Read10X function from Seurat (v5.2.1)^27^ and empty droplets and dead cells were filtered out by exploratory data analysis of QC metrics and manually choosing thresholds to keep cells with percent mitochondrial reads <= 25%, minimum number of genes with nonzero reads per cell >= 100, and minimum total number reads per cell >= 10000. Normalized expression values were calculated with Seurat’s NormalizeData function with default ‘LogNormalize’ options.

#### Preprocessing of breast cancer patient data

For the Klughammer dataset, we used the original cell annotations to select malignant epithelial cells for downstream analysis^28^. For the Luo dataset, we followed the published inferCNV pipeline^29^ to identify malignant cells. Prior to running inferCNV, we performed unsupervised clustering using Seurat^30^, which identified 15 distinct cell clusters (Supplementary Figure 16A). Based on the expression of known immune cell marker genes (Supplementary Table 6), a subset of clusters —specifically clusters 2, 3, 4, 5, 7, 9, 11, 13, and 14 (Supplementary Figure 16A)— was identified as immune cell populations and designated as reference normal cells for the inferCNV analysis. We then applied inferCNV to infer copy number variations (CNVs) across the remaining cells. The analysis revealed consistently abnormal CNV burden across all clusters except one (Supplementary Figure 16B).

Further inspection of this exception group (cluster 1) revealed elevated expression of multiple collagen family genes (Supplementary Figure 16C), suggesting these cells were fibroblasts. As a result, this fibroblast cluster was excluded, and the remaining clusters were retained as malignant epithelial cells. To validate this classification, we examined the expression of EPCAM, a well-established marker of malignant epithelial cells^31^, and confirmed its significant overexpression in the inferred malignant population (Supplementary Figure 16D). This confirmed both the malignant nature and epithelial identity of the selected cells.

### Evaluating Prediction Accuracy Using Distributional Metrics

#### Computing distributional distances between predicted and test-time distributions

To quantify how closely the predicted cell states aligned with the experimentally observed distributions at withheld time points, we computed distributional distances using three metrics: W2 distance, Sinkhorn divergence, and maximum mean discrepancy (MMD). We approximated the W2 distance using the entropic optimal transport cost computed via the SamplesLoss function from the GeomLoss library ^32^. This regularized OT cost is defined as:

(8) $${𝓛}_{\varepsilon }\left(\mu ,v\right)=\underset{\gamma \in \prod \left(a,b\right)}{\min}\sum_{i,j}\gamma_{ij}{\left\|x_{i}-y_{i}\right\|}^{2}+\varepsilon \sum_{i,j}\gamma_{ij}\left(\log\gamma_{ij}-1\right),$$

where 𝛾 is a transport plan between discrete empirical distributions $\mu =\sum_{i}a_{i}\delta_{x_{i}}$ and $v=\sum_{j}b_{j}\delta_{y_{j}}$ with marginals a and b, and ε>0 controls the strength of entropy regularization. This formulation provides a smooth approximation of the W2 distance that is computationally stable and scalable ^33^. To obtain a proper divergence, we computed the Sinkhorn divergence ^32,34^ by correcting for the bias introduced by the entropy term:

(9) $${\text{Sinkhorn}}_{\varepsilon }\left(\mu ,v\right)={𝓛}_{\varepsilon }\left(\mu ,v\right)-\frac{1}{2}\left[{𝓛}_{\varepsilon }\left(\mu ,\mu \right)+{𝓛}_{\varepsilon }\left(v,v\right)\right].$$

We tested a range of entropy regularization parameters ε∈{0.01,0.1,1,10,100} to assess the sensitivity of this metric. Additionally, we computed MMD between predicted and observed distributions using a radial basis function (RBF) kernel:

(10) $$\text{MMD}\left(X,Y\right)=\frac{1}{n^{2}}\sum_{i,j}K\left(x_{i},x_{j}\right)+\frac{1}{m^{2}}\sum_{i,j}K\left(y_{i},y_{j}\right)-\frac{2}{nm}\sum_{i,j}K\left(x_{i},y_{j}\right),$$

where $K\left(x,y\right)=\exp\left(-\gamma {\left\|x-y\right\|}^{2}\right)$ is the RBF kernel with bandwidth γ=1.0 ^35,36^. All computations were performed in Python using the GeomLoss ^32^ and POT (Python Optimal Transport) ^37^ libraries.

#### Permutation testing of predicted versus observed distributions

To assess the agreement between predicted gene expression distributions and experimentally measured distributions at held-out time points, we conducted permutation-based hypothesis testing for each gene. Specifically, we evaluated whether the predicted and observed distributions could be considered samples from the same underlying distribution (null hypothesis), using three complementary distance metrics: total variation distance (TV), Kullback–Leibler divergence (KL), and Sinkhorn divergence. For each gene and each time point, we computed the observed value of the respective distance metric between the predicted and test distributions. We then permuted the pooled samples 1,000 times to construct a null distribution and estimated the empirical *p*-value as the proportion of permuted statistics greater than or equal to the observed one.

To evaluate the robustness of our predictions, we quantified the number of genes for which the null hypothesis could not be rejected (*p* > 0.05) under at least one, at least two, or all three distance metrics. In the mESC dataset (100 genes), at the held-out time point 1, 78 genes passed the test with at least one metric, 50 genes with at least two, and 37 genes with all three. At time point 3, these numbers increased to 100, 99, and 91, respectively. In the EMT dataset (72 genes), when predicting time point 2, 62 genes passed with at least one metric, 8 with at least two, and only 1 gene with all three. These results—summarized in Supplementary Figure 14, with all *p*-values reported in Supplementary Tables 1 and 2—highlight the consistency of PROFET’s predictions and underscore the value of using multiple statistical metrics to validate distributional similarity across time points.

### Quantifying and Classifying Phenotypic Heterogeneity from Predicted Trajectories

#### Displacement-based quantification of phenotypic shift

To quantify the extent of phenotypic shifts inferred by PROFET, we computed the Euclidean displacement for each cell between its predicted pre-treatment and post-treatment states. Summary statistics—including the mean, standard deviation, interquartile range (IQR), coefficient of variation (CV), and entropy of the displacement distribution—were recorded for each dataset (Supplementary Table 4). The full implementation can be found in the plot_X1_hat_displacement_distribution function, which loads the trained model and PCA parameters, computes displacements, saves statistical outputs as CSV files, and generates visualizations.

#### KDE-based stratification of cellular subpopulations

To identify subpopulations with distinct phenotypic shifts, we used kernel density estimation (KDE) to smooth the histogram of displacement values^38^. The density curves were computed using the Scott bandwidth method, and local minima were detected via peak-finding on the inverse KDE curve. The two smallest local minima (based on the 𝑥-axis values) were selected as cutoffs to stratify cells into three groups: low, medium, and high phenotypic shift. This analysis was performed independently for each patient dataset using the corresponding histogram of cell displacements. The KDE and local minima detection steps are implemented in a Python pipeline that reads per-sample histograms, applies KDE smoothing via scipy.stats.gaussian_kde, and uses scipy.signal.find_peaks to locate valleys in the density curve^39^.

## Supplementary Material

Supplement 1

Supplement 2

Supplement 3

Supplement 4

Supplement 5

Supplement 6

Supplement 7

Supplement 8

Supplement 9

Supplement 10

Supplement 11

Supplement 12

Supplement 13

Supplement 14

Supplement 15

## Data Availability

The original datasets used in this study are publicly available from their respective publications: the mESC dataset from Hayashi et al.^12^, the EMT dataset from Deshmukh et al.^13^, patient PA3 data from Luo et al.^14^, and patient 862 and 887 data from Klughammer et al.^15^ The corresponding preprocessed datasets, including the synthetic EMT data used in this study, are available on the Michor Lab GitHub repository: https://github.com/Michorlab/PROFET. The MCF7A cell line dataset generated in this study will be made available via the Gene Expression Omnibus (GEO).
