# Supplementary material for: PROFET Predicts Continuous Gene Expression Dynamics from scRNA-seq Data to Elucidate Heterogeneity of Cancer Treatment Responses
Source: bioRxiv. 2025 Jul 3:2025.06.27.662030. Preprint. [Version 1] doi: 10.1101/2025.06.27.662030 (PMC12236938; doi:10.1101/2025.06.27.662030)
Supplement: Supplement 1 [file media-1.pdf]

Supplementary Notes: PROFET Predicts Continuous Gene Expression Dynamics from scRNA-seq Data to Elucidate Resistance to Cancer Therapy

Yu-Chen Cheng*, Hyemin Gu*, Thomas O. McDonald, Wenbo Wu, Shubham Tripathi, Cristina Guarducci, Douglas Russo, Daniel L. Abravanel, Madeline Bailey, Yue Wang, Yun Zhang, Yannis Pantazis, Herbert Levine, Rinath Jeselsohn, Markos A. Katsoulakis†, Franziska Michor†

*These authors contributed equally to this work.
†Corresponding authors.

Supplementary Note 1: Wasserstein Gradient Flow and Lipschitz-Regularized KL Divergence

We briefly review the mathematical foundation behind the Generative Particles Algorithm (GPA), which simulates Wasserstein gradient flows driven by divergences between probability measures.

Wasserstein gradient flow of probability measures. Let ρ_t be a time-indexed family of probability distributions evolving over $\mathbb{R}^d$. The Wasserstein gradient flow describes their evolution as a solution to the continuity equation

$$\partial_t \rho_t + \nabla \cdot (\rho_t v_t) = 0, \tag{1}$$

where the velocity field v_t is given by the Wasserstein gradient of an energy functional $\mathcal{F} : \mathcal{P}(\mathbb{R}^d) \to \mathbb{R}$, namely:

$$v_t = -\nabla \frac{\delta \mathcal{F}}{\delta \rho}(\rho_t), \tag{2}$$

with $\frac{\delta \mathcal{F}}{\delta \rho}(\rho_t)$ denoting the first variation of $\mathcal{F}$ with respect to ρ_t. This framework provides a natural interpretation of dynamics over probability measures as steepest descent flows in the space of distributions.

KL divergence and its regularization. A common choice for the energy functional is the Kullback–Leibler (KL) divergence, defined as $\mathrm{KL}(\rho_t \| \pi) = \mathbb{E}_{\rho_t}\left[\log\left(\frac{d\rho_t}{d\pi}\right)\right]$, where π is a fixed target distribution. This formulation requires that ρ_t is absolutely continuous with respect to π (i.e., $\rho_t \ll \pi$), so that the Radon–Nikodym derivative $\frac{d\rho_t}{d\pi}$ exists. In practice, both ρ_t and π may be represented only by samples, and neither density may be available in closed form.

In such cases, we instead consider the variational formulation of f-divergences, which allows estimation directly from samples:

$$D_f(\rho_t \| \pi) = \sup_{\phi \in C_b(\mathbb{R}^d)} \left\{ \mathbb{E}_{\rho_t}[\phi] - \inf_{\nu \in \mathbb{R}} \mathbb{E}_\pi [f^\star(\phi - \nu) + \nu] \right\}, \tag{3}$$

where $f(x) = x \log x$ for KL divergence and $f^\star$ is its Legendre transform. This formulation does not require access to densities or absolute continuity with respect to Lebesgue measure, making it particularly suitable for sample-based approximation.

However, for empirical distributions, KL divergence may diverge or become non-differentiable due to lack of absolute continuity. To address this, we use a Lipschitz-regularized f-divergence with $f(x) = x \log x$, which remains well-defined even for discrete measures. It admits a dual variational representation:

$$D_f^{\mathrm{Lip}_L(\mathbb{R}^d)}(\rho_t \| \pi) = \sup_{\phi \in \mathrm{Lip}_L(\mathbb{R}^d)} \left\{ \mathbb{E}_{\rho_t}[\phi] - \inf_{\nu \in \mathbb{R}} \mathbb{E}_\pi [f^\star(\phi - \nu) + \nu] \right\}, \tag{4}$$

where $f^\star$ is the Legendre transform of f, and the test function ϕ is constrained to be L-Lipschitz. The optimal potential $\phi_t^{L,*}$ obtained from this formulation approximates the first variation $\frac{\delta \mathcal{F}}{\delta \rho}(\rho_t)$, ensuring that the induced velocity field $v_t = -\nabla \phi_t^{L,*}$ is both numerically stable and biologically plausible.

This formulation avoids the need for explicit densities or absolute continuity with respect to Lebesgue measure, and its robustness under minimal moment assumptions is further strengthened when combined with Lipschitz constraints, as discussed in Supplementary Information 2.

Supplementary Note 2: Robustness through Lipschitz regularization

In Wasserstein gradient flows driven by KL divergence, the velocity field is given by the gradient of the first variation of the divergence functional. For the unregularized KL divergence, this first variation is the log-density ratio:

$$\frac{\delta}{\delta \rho} \mathrm{KL}(\rho \| \pi) = \log\left(\frac{d\rho}{d\pi}\right), \tag{5}$$

which defines the velocity field as:

$$v_t(x) = -\nabla \log\left(\frac{d\rho_t}{d\pi}\right). \tag{6}$$

However, this expression is only well-defined when the source distribution ρ_t is absolutely continuous with respect to the target distribution π. In practical applications—especially when ρ_t is an empirical distribution represented by discrete particles—this condition typically fails, resulting in an infinite KL divergence and an undefined first variation. As a result, the velocity field breaks down and cannot be used for gradient flow simulation.

Lipschitz regularization overcomes this limitation by defining a relaxed divergence:

$$D_f^{\mathrm{Lip}_L}(\rho_t\|\pi) = \sup_{\phi\in\mathrm{Lip}_L}\left\{\mathbb{E}_{\rho_t}[\phi] - \inf_{\nu\in\mathbb{R}}\mathbb{E}_\pi[f^\star(\phi-\nu)+\nu]\right\}, \tag{7}$$

which remains finite and admits a well-defined first variation as long as ρ_t has finite first moment [1]—even when ρ_t is not absolutely continuous w.r.t. π, and even when π is heavy-tailed, supported on low-dimensional manifolds, or discrete, as in our case. This allows our method, which learns dynamics through Lipschitz-regularized gradient flows, to remain agnostic to the parametric form or regularity of the target distribution, enabling a fully data-driven modeling of transitions. The optimal potential $\phi_t^{L,*}$ serves as a proxy for $\log\left(\frac{d\rho_t}{d\pi}\right)$; more precisely, $\phi_t^{L,*}$ approximates $\log\left(\frac{d\rho_t}{d\pi}\right)$ using $\log\left(\frac{d\sigma_t}{d\pi}\right)$, where σ_t is an intermediate measure that minimizes the KL divergence to π while remaining close to ρ_t in Wasserstein-1 distance. This formulation effectively estimates the gradient of the log-likelihood while maintaining robustness and boundedness. Consequently, the velocity field

$$v_t(x) = -\nabla\phi_t^{L,*}(x) \tag{8}$$

remains numerically stable throughout training and enables particle evolution under ill-posed conditions that would render the standard KL-based gradient flow inapplicable.

Supplementary Note 3: Velocity growth in KL gradient flow and the role of Lipschitz regularization

While the KL divergence is well-defined for many continuous distributions, its associated gradient flow can yield unbounded velocity fields, making it challenging to simulate numerically. To illustrate, consider two distributions from the exponential family:

$$p(x) = h(x)\exp\left(\langle\theta_p, T(x)\rangle - A(\theta_p)\right), \quad q(x) = h(x)\exp\left(\langle\theta_q, T(x)\rangle - A(\theta_q)\right). \tag{9}$$

with log-density ratio and gradient:

$$\log\frac{p(x)}{q(x)} = \langle\theta_p-\theta_q, T(x)\rangle - \left(A(\theta_p)-A(\theta_q)\right), \quad \nabla_x\log\frac{p(x)}{q(x)} = J_T(x)^\top(\theta_p-\theta_q). \tag{10}$$

When the sufficient statistics $T(x)$ contain polynomial components, the gradient can grow without bound as $|x|\to\infty$. This leads to a velocity field that diverges in magnitude, rendering the corresponding gradient flow ill-posed or numerically unstable.

However, empirical distributions are inherently supported on compact subsets of $\mathbb{R}^d$. Within this bounded support, the gradient field can be well-approximated by a Lipschitz-constrained potential $-\nabla\phi_t^{L,*}$, where the Lipschitz constant L sets an upper bound on velocity magnitude. When L is chosen sufficiently large, the resulting flow closely mimics the unregularized KL-induced dynamics — such as the Ornstein–Uhlenbeck (OU) process — while avoiding the instability of unbounded acceleration.

Figure 1 illustrates this behavior, showing that GPA with a large L (e.g., $L = 10$) can recover the convergence dynamics of the OU process, whereas smaller L values lead to slower convergence due to more limited transport velocity.

Supplementary Note 4: Theoretical guarantee for latent-space modeling

Let $\mathcal{E}:\mathbb{R}^D\to\mathbb{R}^d$ and $\mathcal{D}:\mathbb{R}^d\to\mathbb{R}^D$be Lipschitz encoder and decoder maps, and let P and Q be probability measures in the latent and ambient spaces respectively. The Lipschitz-regularized f-divergence satisfies the inequality [2]:

$$D_f^{\mathrm{Lip}_L}(\mathcal{D}_\#P|Q) \le D_f^{a_\mathcal{D}\mathrm{Lip}_L}(P|\mathcal{E}_\#Q), \tag{11}$$

where $a_\mathcal{D}$ is the Lipschitz constant of the decoder.

This inequality implies that the modeling error incurred by transporting distributions in latent space and decoding back to ambient space is controlled. In particular, if the decoder has bounded distortion, the divergence in the ambient space remains upper-bounded by a Lipschitz-scaled divergence in the latent space. This justifies the use of GPA in reduced dimensions, especially in biological applications where the intrinsic data manifold may be significantly lower-dimensional.

Supplementary Note 5: Comparison to flow matching in score-based generative modeling

After GPA simulation, we learn a continuous-time Eulerian velocity field $v_\theta(x,s)$ using force-matching. This distills a sequence of local, time-indexed potentials $\{\phi_n^{L,*}\}$ into a single neural vector field that governs global dynamics.

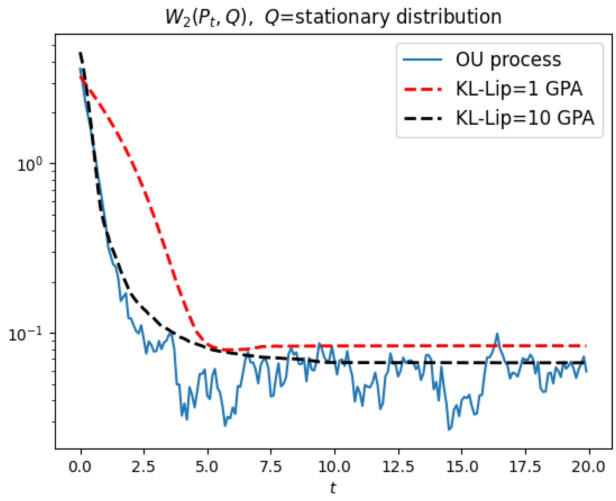

Figure 1: Convergence speed of Lipschitz-regularized KL GPA with different Lipschitz constants $L = 1, 10$ and OU process. $L = 10$ approximately recovers the convergence speed from the OU process. Stationary distribution is a Gaussian distribution.

Structurally, our force-matching loss closely resembles the denoising score matching (DSM) loss used in score-based generative models. The DSM objective takes the form:

$$\mathcal{L}_{\text{DSM}}(\theta) = \mathbb{E}_{t\sim\mathcal{U}(0,T)}\mathbb{E}_{x_0\sim\text{pdata},\, x_t\sim\mathcal{N}(x_0,\sigma_t^2 I)}\left[\left|s_\theta(x_t, t) - \frac{1}{\sigma_t^2}(x_0 - x_t)\right|^2\right], \tag{12}$$

where a score network s_θ is trained to recover the gradient of the log-density at perturbed inputs, assuming an analytically defined forward process (e.g., Gaussian noise).

In contrast, our force-matching objective:

$$\mathbb{E}_{(x,s)}\left[\left|v_\theta(x, s) + \nabla\phi_s^{L,*}(x)\right|^2\right], \tag{13}$$

supervises the neural velocity field using gradients derived from GPA-based simulations, without relying on a parametric form for the forward dynamics.

This key distinction enables our approach to flexibly model complex systems in which analytic transition distributions (e.g., Gaussian noise or OT interpolation) are inadequate or biologically unrealistic. Whereas traditional flow-matching methods [3] and score-based models [4] typically impose Gaussian smoothing or regular OT priors, our method infers transitions directly from empirical distributions. This allows it to capture biologically relevant phenomena such as bifurcations, convergence to terminal states, or heterogeneous treatment responses—dynamics that arise naturally in developmental and disease progression settings.

Supplementary Note 6: Simulation setup to model epithelial-mesenchymal transition

Capturing biological variability in EMT dynamics Various studies profiling EMT at single-cell resolution have reported heterogeneity in gene expression dynamics even in a genetically homogeneous population [5, 6, 7]. To capture this behavior, we simulated Eq. (6) (Methods) for an ensemble of parameter sets and for multiple random initial conditions for each parameter set in the ensemble. Parameter sets in the ensemble were sampled in a manner that captures the range of biologically plausible behaviors (see [8] for a detailed description of the sampling scheme). In the absence of noise (*i.e.*, for $D = 0$), the EMT gene regulatory network in our setup exhibits multi-stability, exhibiting stable states that span the spectrum from epithelial to mesenchymal phenotypes [9]. To simplify the analysis, we consider only those parameter sets that exhibit tri-stability for $D = 0$; the three steady states for each parameter set can correspond to epithelial, hybrid epithelial / mesenchymal, and mesenchymal phenotypes which are the key categories of phenotypic states studied in EMT. Parameter sets with fewer or more stable states when no noise is present were discarded from the ensemble. For each such parameter set, we simulated the dynamics starting from 10 random initial conditions from $t = 0$ to $t = 1000$ (shown in Fig. 2AB). Each simulated trajectory in our setup can then be interpreted as corresponding to the dynamical behavior of a single cell. Note that the time here is simulation time and its mapping to real time will depend on the timescale associated with different regulatory interactions in the gene network.

Simulating perturbation-induced transition from epithelial to mesenchymal state Simulating dynamics for a given parameter set starting from a random initial condition can result in a trajectory that leads to an epithelial, hybrid, or mesenchymal state. Since we want to focus on transition from epithelial to mesenchymal states, we discarded trajectories that failed to remain in an epithelial state (characterized by high expression of CDH1 and low expression of VIM) between $t = 200$ and $t = 500$. At $t = 500$, we introduced a perturbation that can trigger a transition from an epithelial state to

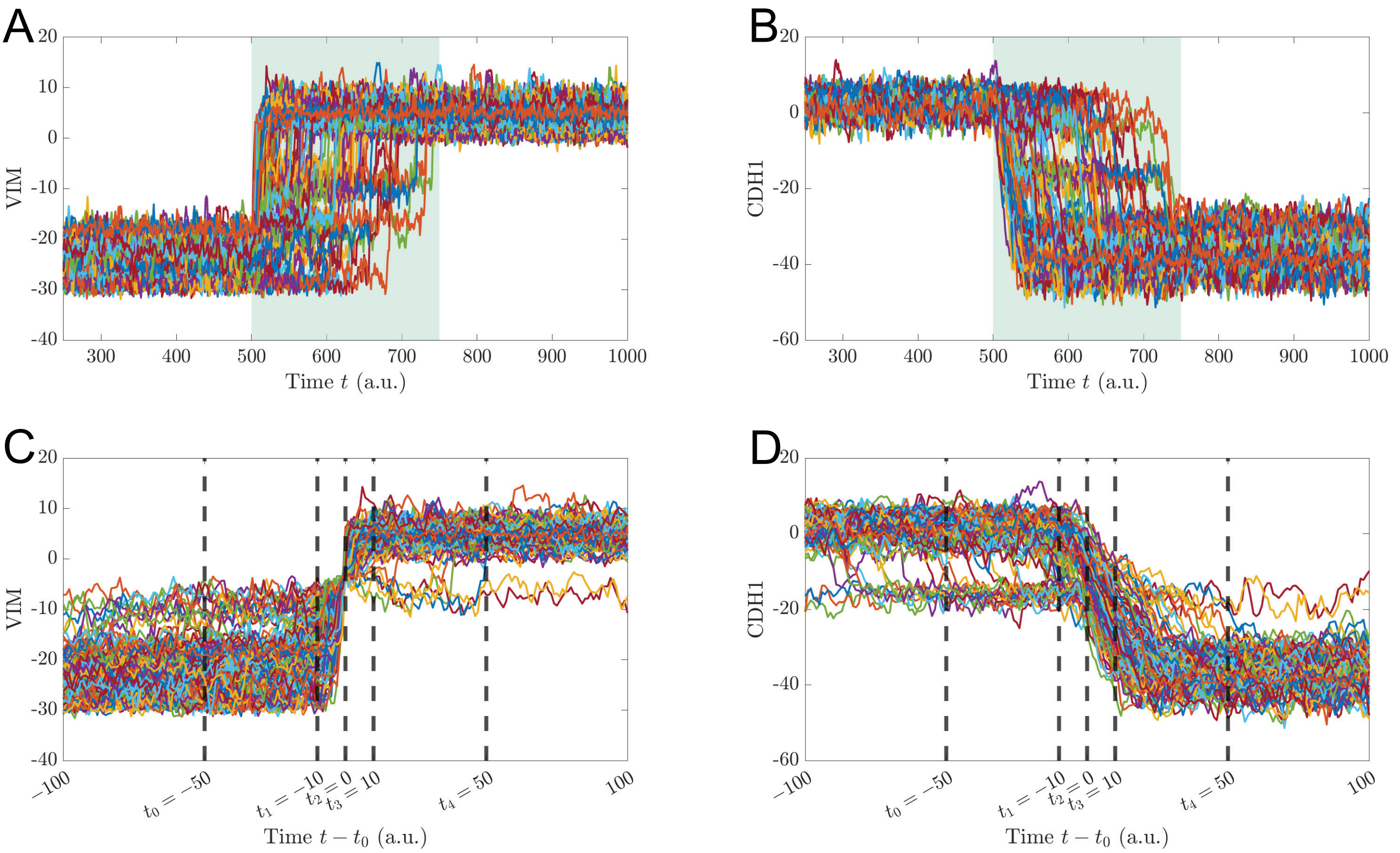

Figure 2: Simulation setup to model epithelial-mesenchymal transition in a population of epithelial cells. (A) Simulated expression levels (log2-transformed) of VIM, a mesenchymal marker, and (B) of CDH1, an epithelial marker, along the simulated trajectories. Each trajectory shown can be interpreted as representing the behavior of a single cell in the population. The shaded region indicates the time period for which the EMT-inducing perturbation (SNAIL up-regulation, miR-34 down-regulation) was active. (C) Same trajectories as in A and (D) as in B shown with shifted time. Each trajectory is shifted by t_0, the time point at which the VIM expression along the trajectory (log2-transformed) becomes positive for the first time during the perturbation period.

a mesenchymal state: 100-fold increase in the production rate of SNAIL and 100-fold decrease in the production rate of miR-34. This perturbation lasted till $t = 750$ at which point it was withdrawn and SNAIL and miR-34 production rates restored to their original values (see shaded region in Fig. 2AB). The perturbation causes cells (represented by trajectories) to start transitioning to mesenchymal states characterized by low expression of CDH1 and high expression of VIM. All cells in our setup do not transition at the same time within the perturbation window, consistent with experimental data [7], and cells can remain in the mesenchymal state even after the EMT-inducing perturbation has been withdrawn (*i.e.*, for $t > 750$). Note that all cells in our simulation setup do not transition to a mesenchymal state in response to the perturbation and among those that do transition, not all remain in the mesenchymal state after the perturbation is withdrawn; trajectories corresponding to such behavior were also discarded. Fig. 2AB show numerous simulated trajectories retained in the ensemble and their response to the perturbation.

To generate a synthetic dataset for PROFET training, we aligned the simulated trajectories by the time point at which the log2-transformed VIM expression level first becomes positive (representing the transition to a more mesenchymal state). Fig. 2 C-D show the trajectories shifted by their transition time points. This step is equivalent to the pseudotime analysis done in various single-cell studies to group together cells that are at different stages of transition from one phenotype to another [6, 7], and is needed since all cells in a population do not undergo phenotypic transition at the same rate (a behavior that is recapitulated in our simulation setup). Thereafter, we sampled the simulated cell population at five points in shifted time (indicated by dashed vertical lines in Fig. 2 C-D). Sampled cell states at these time points (before, during, and after EMT) constituted the synthetic longitudinal dataset that was then used to train the PROFET model and to test the performance of the overall approach.

Supplementary Note 7: Full description of novelty and comparison to existing methods

Gradient flow-based modeling from empirical data. We propose an agnostic and data-driven framework for modeling temporal dynamics via Wasserstein gradient flows. At the core of our approach is GPA, which simulates particle trajectories that follow the gradient flow of an energy functional—specifically, a Lipschitz-regularized KL divergence. Unlike OT-based methods that optimize static couplings or CNFs that rely on a predefined base distribution, our method directly learns smooth dynamics from solely empirical source and target distributions, without assuming specific distributional forms or

mechanistic priors. The learned trajectories interpolate between observed single-cell snapshots in a fully nonparametric framework that remains robust to complex, irregular, or discrete data supports, enabling flexible and interpretable modeling of biological processes.

Robustness and stability via Lipschitz-regularzied divergence. A key strength of our method lies in the robustness and stability of the Lipschitz-regularized KL divergence used to define the gradient flow. This regularization ensures that the induced velocity field is bounded by a constant L, which makes the simulation numerically stable and imposes a biologically sensible constraint on transport speeds—unlike unregularized OT flows with unbounded gradients or diffusion models with predefined stochasticity. As shown in [1], the resulting divergence $D_f^{\Gamma_L}(\rho\|\pi)$ is well-defined and admits stable variational gradients for arbitrary target distributions π, as long as the current distribution ρ—which evolves step-by-step during the GPA simulation—has finite first moment. This makes the method particularly robust in challenging settings where the distributions are heavy-tailed, lie on low-dimensional or fractal manifolds, or are purely discrete—as is often the case in single-cell data.

Learning generalizable velocity fields via force matching. After GPA simulation, we learn a continuous-time Eulerian velocity field $v_\theta(x, s)$ using force-matching, distilling local potentials $\{\phi_n^{L,*}\}$ into a unified neural vector field. This process enables generalization across time and space, without relying on an analytical form of the dynamics. While the structure of our objective is reminiscent of denoising score matching or flow-matching approaches, a detailed comparison—including its connection and distinction from score-based generative models—is provided in Supplementary Note 4.

Comparison to static Optimal Transport methohs. Compared to Optimal Transport (OT) methods[10] that only couple marginal distributions, our framework recovers full temporal trajectories, ensuring dynamic consistency across time. OT-based approaches compute static couplings that optimize transport cost between endpoints, but do not model intermediate states explicitly. As a result, they naturally lead to straight-line transport paths that do not capture the curvature or temporal structure of biological dynamics. This limitation makes OT-based models ill-suited for applications where understanding the continuous evolution of cell states is essential.

Comparison to Continuous Normalizing Flows Continuous Normalizing Flows (CNFs) [11] extend transport modeling to the trajectory level by learning time-dependent velocity fields that map a base distribution to data. However, vanilla CNFs match only the endpoint distributions and place no constraint on the intermediate paths. This under-determined nature leads to instability during training, as many possible flows can yield the same marginal match. To mitigate this, CNFs commonly introduce an additional regularization term that penalizes the kinetic energy of the trajectory, thereby encouraging smoother paths. While this improves training stability, it induces a strong inductive bias: minimizing kinetic energy leads to straight-line trajectories, regardless of the endpoint matching metric. As rigorously shown in [12] using mean-field game and Hamilton–Jacobi arguments, this bias persists even when the training objective includes non-OT terms such as KL divergence.

In addition, CNFs typically require a predefined source distribution—often a standard Gaussian—which is pushed forward to match the target. This choice can significantly bias the learned trajectories when the true source distribution differs from this prior. In our experiments with TrajectoryNet [13] in Figure 3, we observed that this mismatch distorts the inferred dynamics: although the endpoints are matched, the interpolated paths veer away from the biologically meaningful intermediate distributions and fail to recover the true temporal progression. In contrast, our method does not assume any parametric source distribution and instead simulates dynamics directly between empirical snapshots, enabling robust and faithful recovery of nonlinear and branching behaviors observed in real biological processes.

References

[1] Ziyu Chen, Hyemin Gu, Markos A. Katsoulakis, Luc Rey-Bellet, and Wei Zhu. Robust generative learning with lipschitz-regularized α-divergences allows minimal assumptions on target distributions, 2024.

[2] Hyemin Gu, Panagiota Birmpa, Yannis Pantazis, Luc Rey-Bellet, and Markos A. Katsoulakis. Lipschitz-regularized gradient flows and generative particle algorithms for high-dimensional scarce data. *SIAM Journal on Data Science*, 2024. To appear.

[3] Yaron Lipman, Ricky T. Q. Chen, Heli Ben-Hamu, Maximilian Nickel, and Matthew Le. Flow matching for generative modeling. In *The Eleventh International Conference on Learning Representations, ICLR 2023, Kigali, Rwanda, May 1-5, 2023*. OpenReview.net, 2023.

[4] Yang Song, Jascha Sohl-Dickstein, Diederik P. Kingma, Abhishek Kumar, Stefano Ermon, and Ben Poole. Score-based generative modeling through stochastic differential equations. In *9th International Conference on Learning Representations, ICLR 2021, Virtual Event, Austria, May 3-7, 2021*. OpenReview.net, 2021.

[5] Toni Celià-Terrassa, Caleb Bastian, Daniel D. Liu, Brian Ell, Nicole M. Aiello, Yong Wei, Jose Zamalloa, Andres M. Blanco, Xiang Hang, Dmitriy Kunisky, Wenyang Li, Elizabeth D. Williams, Herschel Rabitz, and Yibin Kang. Hysteresis control of epithelial-mesenchymal transition dynamics conveys a distinct program with enhanced metastatic ability. *Nat. Commun.*, 9:5005, 2018.

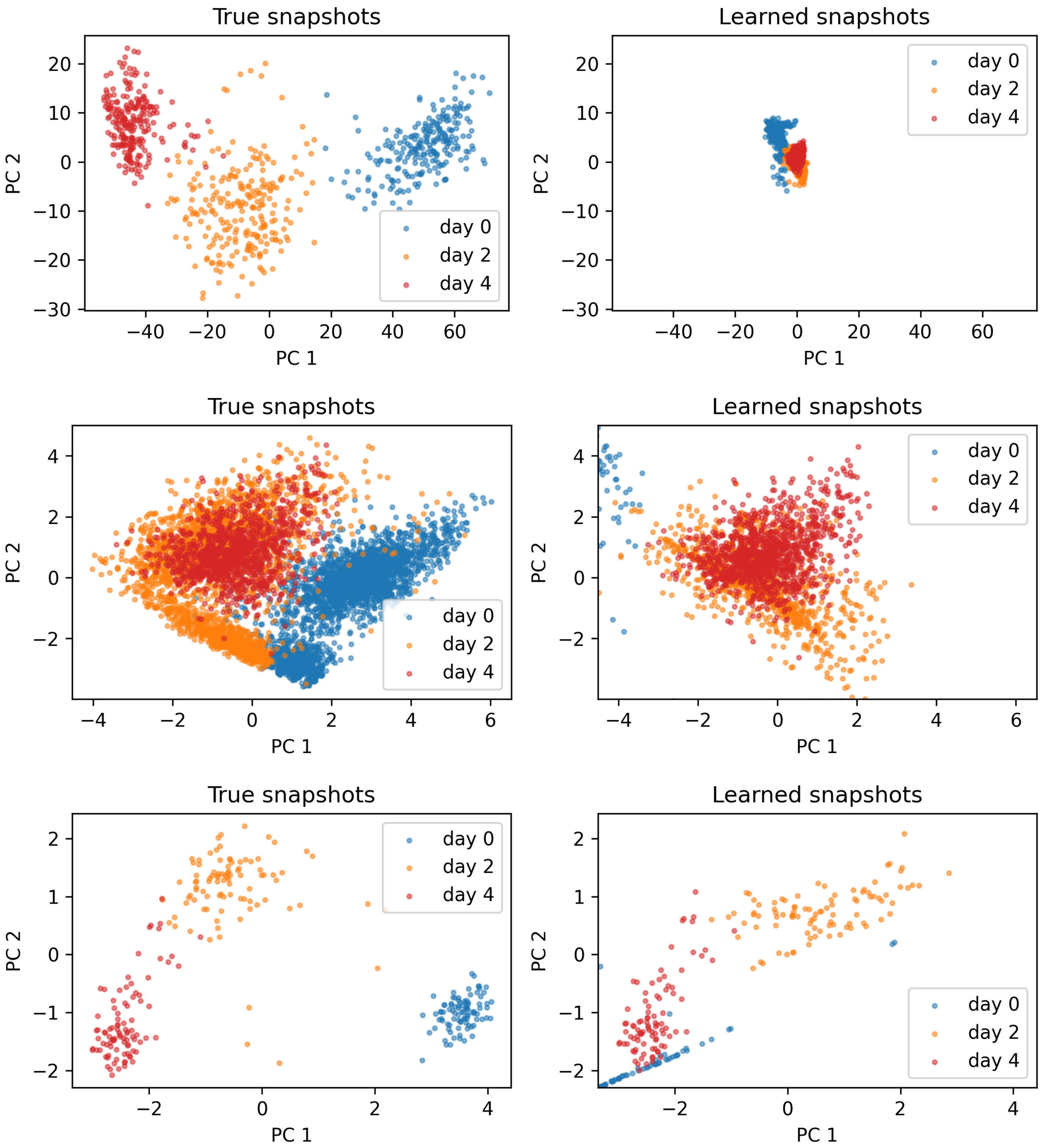

Figure 3: The learned trajectories from TrajectoryNet [13] on synthetic data (top), EMT data (center), and stem cell differentiation data (bottom) are completely off the trajectories. This trajectory bias worsens as the data support becomes broader, since CNF-based methods tend to move particles toward a Gaussian prior, inducing stiffness in the dynamics.

[6] David P. Cook and Barbara C. Vanderhyden. Context specificity of the emt transcriptional response. *Nat. Commun.*, 11:2142, 2020.

[7] Abhijeet P. Deshmukh, Suhas V. Vasaikar, Katarzyna Tomczak, Shubham Tripathi, Petra den Hollander, Emre Arslan, Priyanka Chakraborty, Rama Soundararajan, Mohit Kumar Jolly, Kunal Rai, Herbert Levine, and Sendurai A. Mani. Identification of EMT signaling cross-talk and gene regulatory networks by single-cell RNA sequencing. *Proc. Natl. Acad. Sci. U.S.A.*, 118(19):e2102050118, 2021.

[8] Bin Huang, Mingyang Lu, Dongya Jia, Eshel Ben-Jacob, Herbert Levine, and Jose N. Onuchic. Interrogating the topological robustness of gene regulatory circuits by randomization. *PLOS Comput. Biol.*, 13(3):1–21, 2017.

[9] Dongya Jia, Jason T George, Satyendra C Tripathi, Deepali L Kundnani, Mingyang Lu, Samir M Hanash, José N Onuchic, Mohit Kumar Jolly, and Herbert Levine. Testing the gene expression classification of the EMT spectrum. *Phys. Biol.*, 16(2):025002, 2019.

[10] Geoffrey Schiebinger, Jian Shu, Marcin Tabaka, Brian Cleary, Vidya Subramanian, Aryeh Solomon, Joshua Gould, Siyan Liu, Stacie Lin, Peter Berube, et al. Optimal-transport analysis of single-cell gene expression identifies developmental trajectories in reprogramming. *Cell*, 176(4):928–943, 2019.

[11] Ricky TQ Chen, Yulia Rubanova, Jesse Bettencourt, and David K Duvenaud. Neural ordinary differential equations. *Advances in neural information processing systems*, 31, 2018.

[12] Hyemin Gu, Markos A. Katsoulakis, Luc Rey-Bellet, and Benjamin J. Zhang. Combining wasserstein-1 and wasserstein-2 proximals: robust manifold learning via well-posed generative flows, 2024.

[13] Alexander Tong, Jessie Huang, Guy Wolf, David van Dijk, and Smita Krishnaswamy. Trajectorynet: A dynamic optimal transport network for modeling cellular dynamics. In *Proceedings of the 37th International Conference on Machine Learning, ICML 2020, 13-18 July 2020, Virtual Event*, volume 119 of *Proceedings of Machine Learning Research*, pages 9526–9536. PMLR, 2020.
