# Supplementary material for: PROFET Predicts Continuous Gene Expression Dynamics from scRNA-seq Data to Elucidate Heterogeneity of Cancer Treatment Responses
Source: bioRxiv. 2025 Jul 3:2025.06.27.662030. Preprint. [Version 1] doi: 10.1101/2025.06.27.662030 (PMC12236938; doi:10.1101/2025.06.27.662030)
Supplement: Supplement 2 [file media-2.pdf]

Supplementary Figure 1

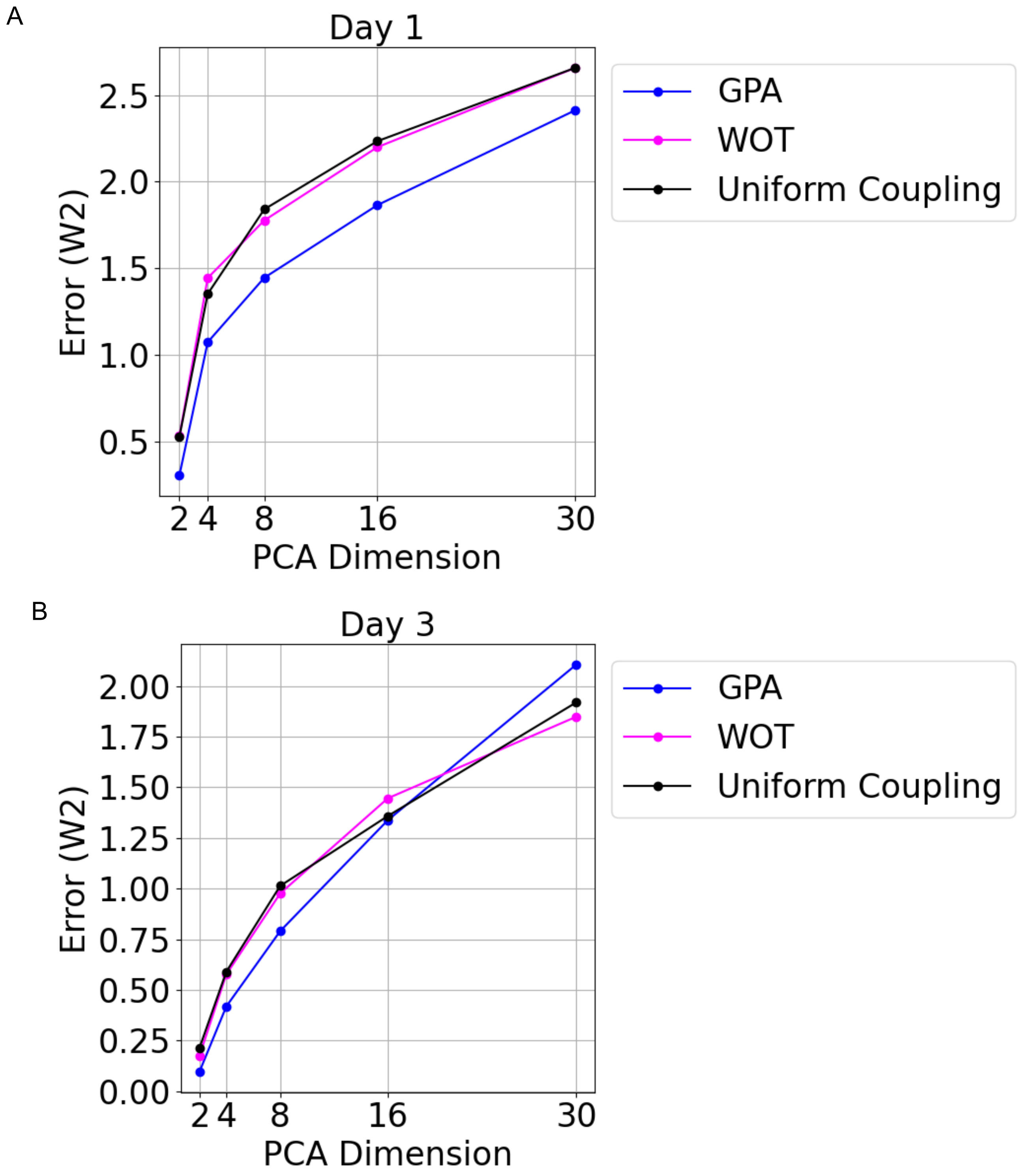

C

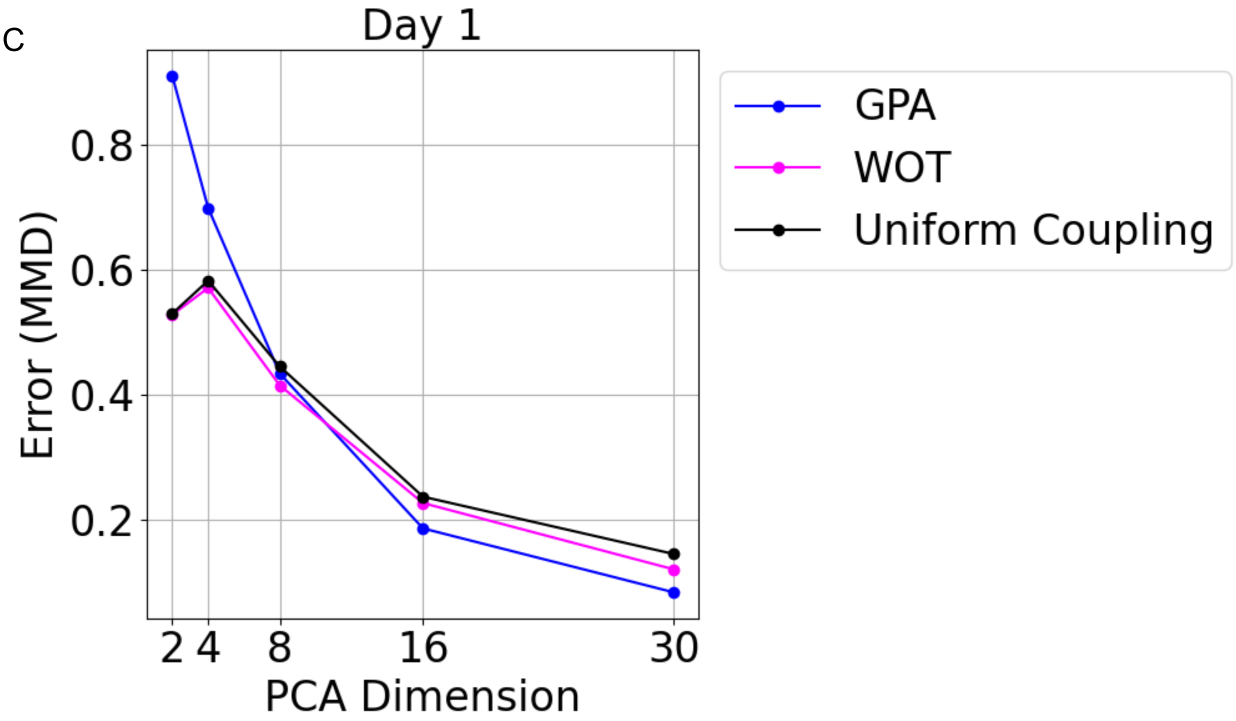

Day 1
0.8
0.6
0.4
0.2
Error (MMD)
2 4 8 16 30
PCA Dimension
GPA
WOT
Uniform Coupling

D

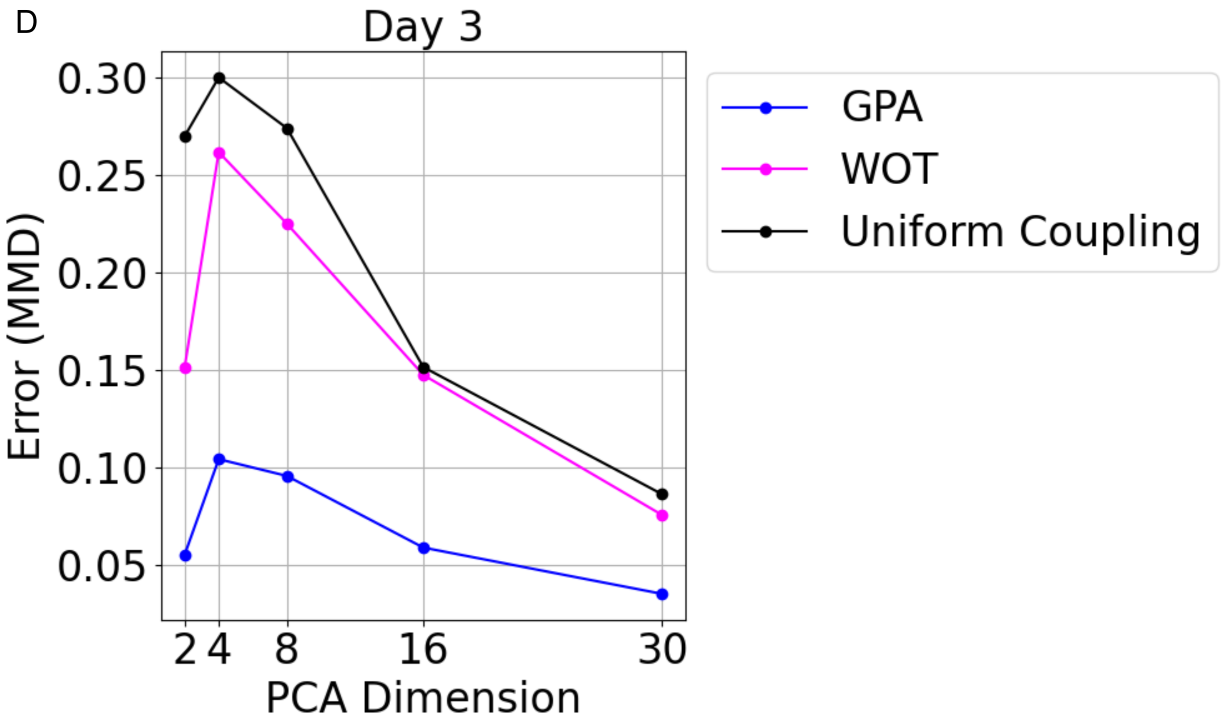

Day 3
0.30
0.25
0.20
0.15
0.10
0.05
Error (MMD)
2 4 8 16 30
PCA Dimension
GPA
WOT
Uniform Coupling

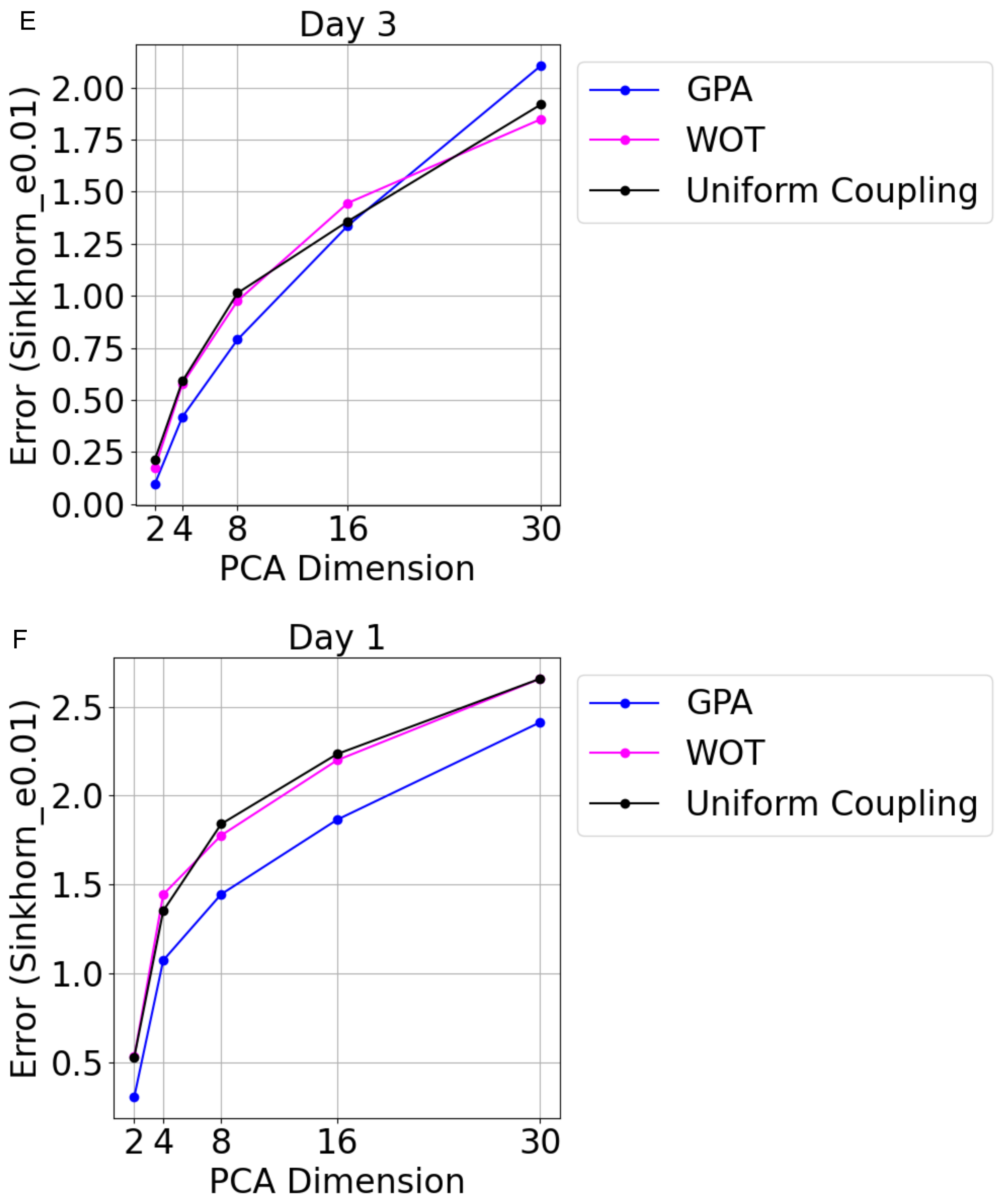
E
Day 3
Error (Sinkhorn_e0.01)
0.00
0.25
0.50
0.75
1.00
1.25
1.50
1.75
2.00
2 4 8 16 30
PCA Dimension
GPA
WOT
Uniform Coupling
F
Day 1
Error (Sinkhorn_e0.01)
0.5
1.0
1.5
2.0
2.5
2 4 8 16 30
PCA Dimension
GPA
WOT
Uniform Coupling

G

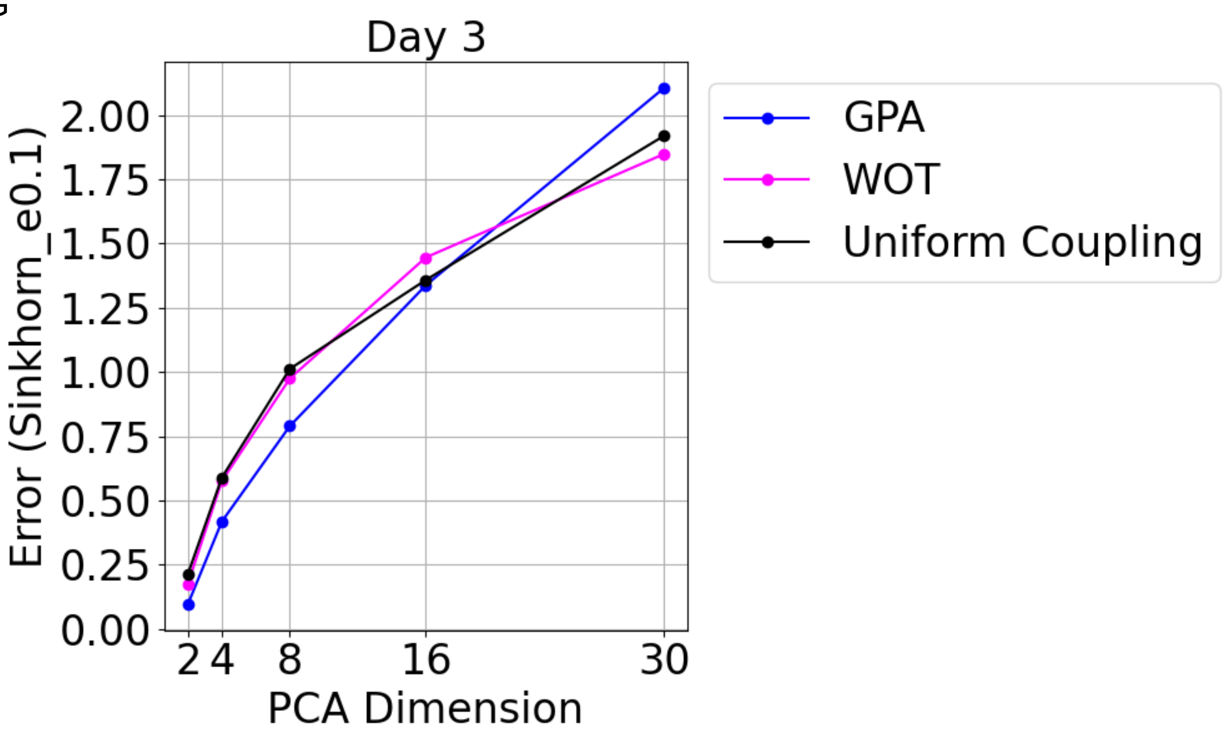

Day 3
Error (Sinkhorn_e0.1)
0.00
0.25
0.50
0.75
1.00
1.25
1.50
1.75
2.00
2
4
8
16
30
PCA Dimension
GPA
WOT
Uniform Coupling

H

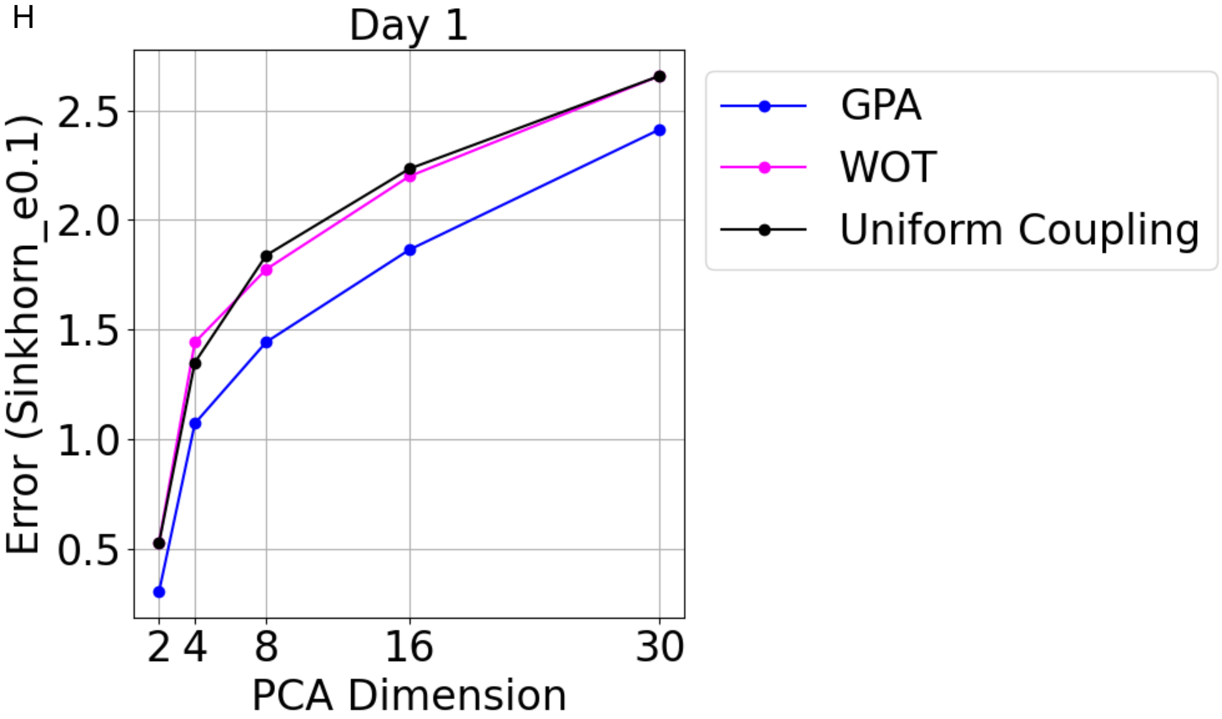

Day 1
Error (Sinkhorn_e0.1)
0.5
1.0
1.5
2.0
2.5
2
4
8
16
30
PCA Dimension
GPA
WOT
Uniform Coupling

I

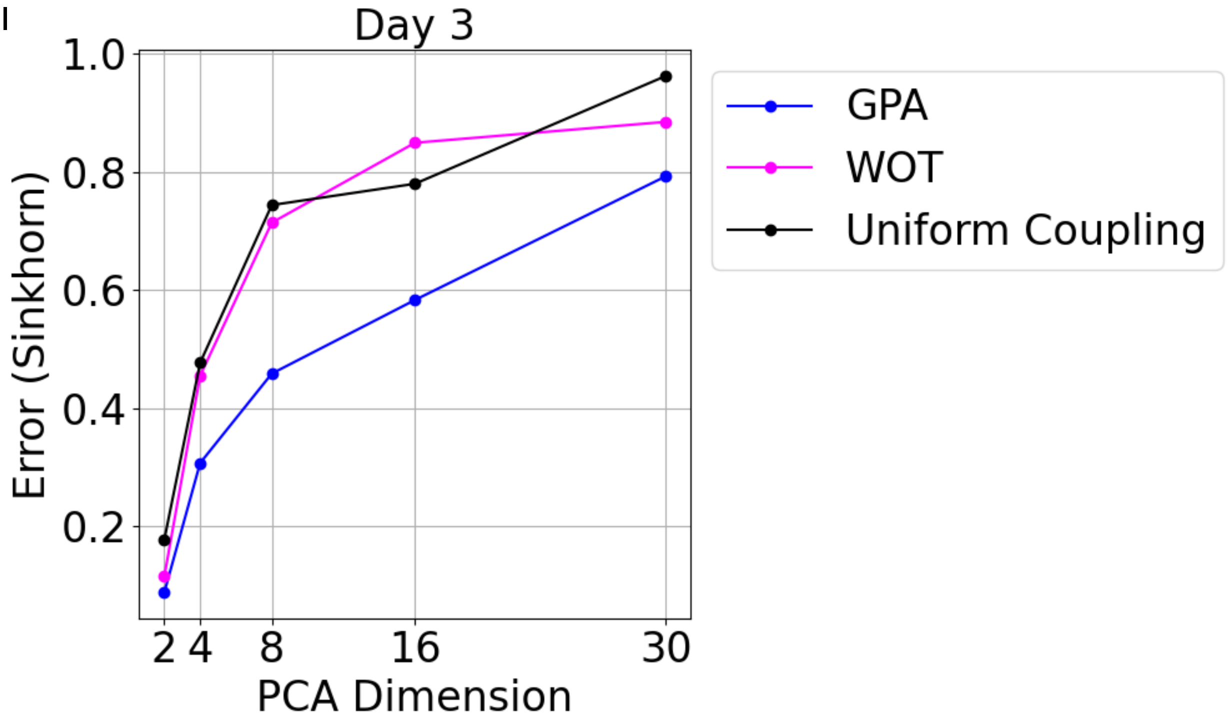
Day 3
Error (Sinkhorn)
1.0
0.8
0.6
0.4
0.2
2 4 8 16 30
PCA Dimension
GPA
WOT
Uniform Coupling

J

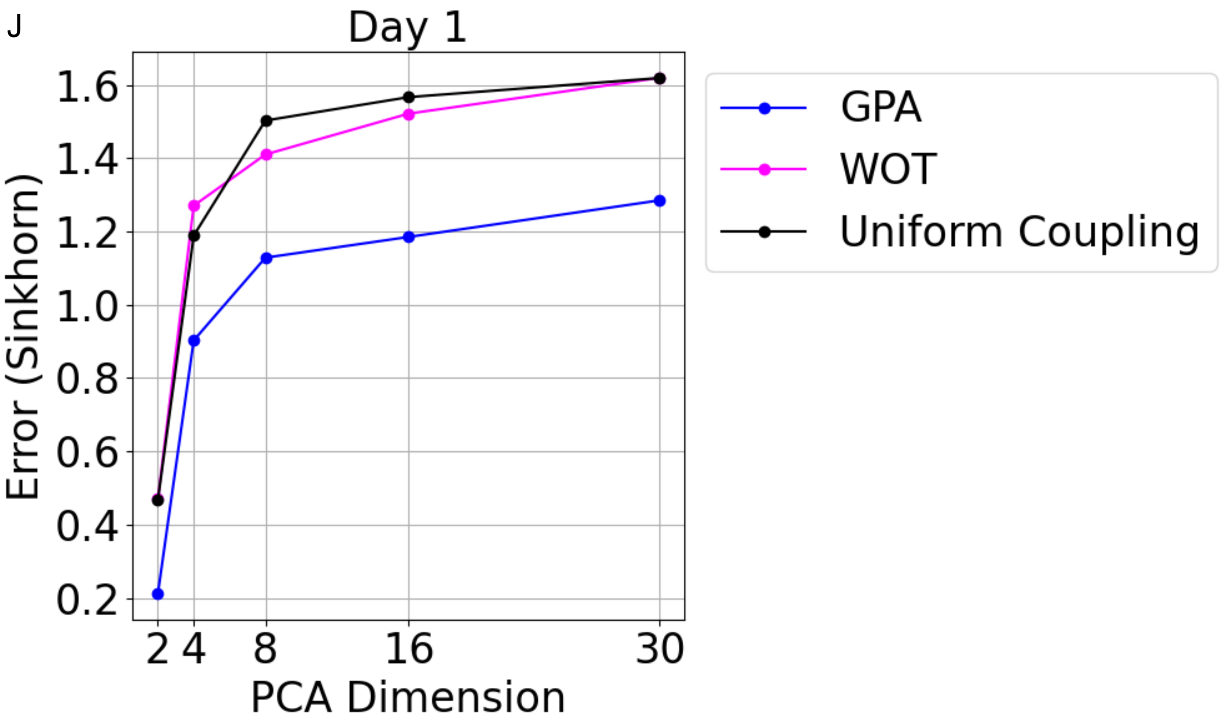
Day 1
Error (Sinkhorn)
1.6
1.4
1.2
1.0
0.8
0.6
0.4
0.2
2 4 8 16 30
PCA Dimension
GPA
WOT
Uniform Coupling

K

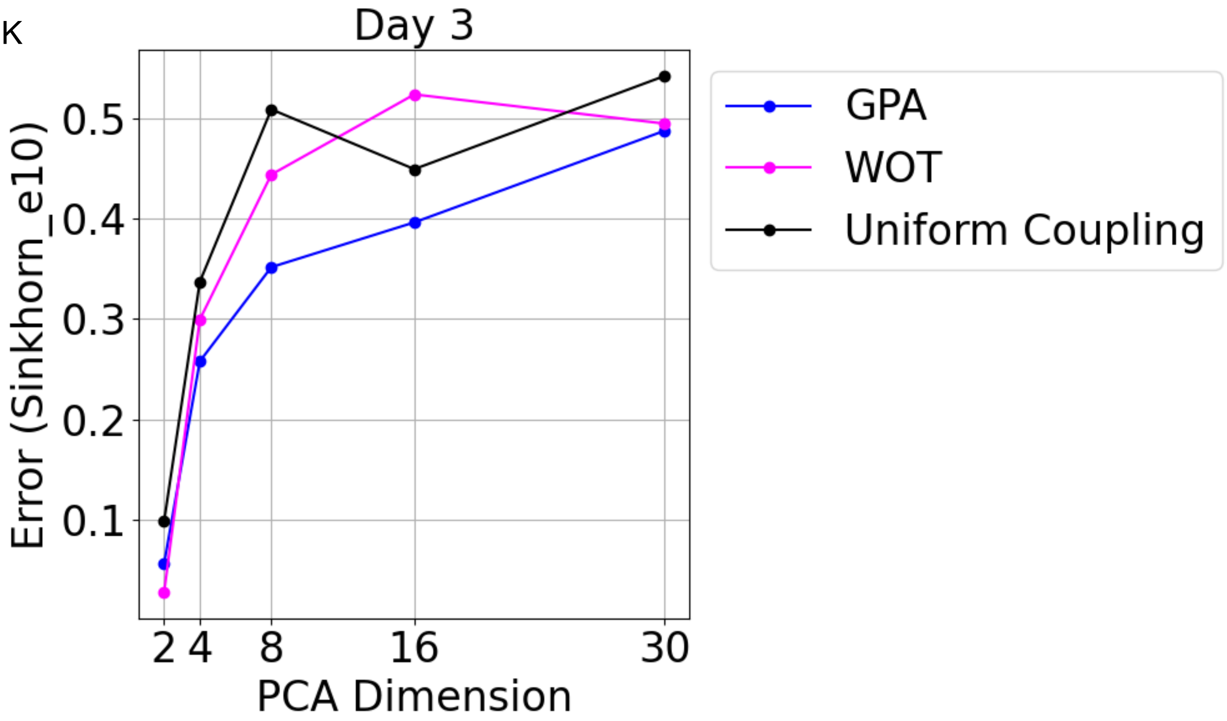
Day 3
Error (Sinkhorn_e10)
0.1
0.2
0.3
0.4
0.5
2 4 8 16 30
PCA Dimension
GPA
WOT
Uniform Coupling

L

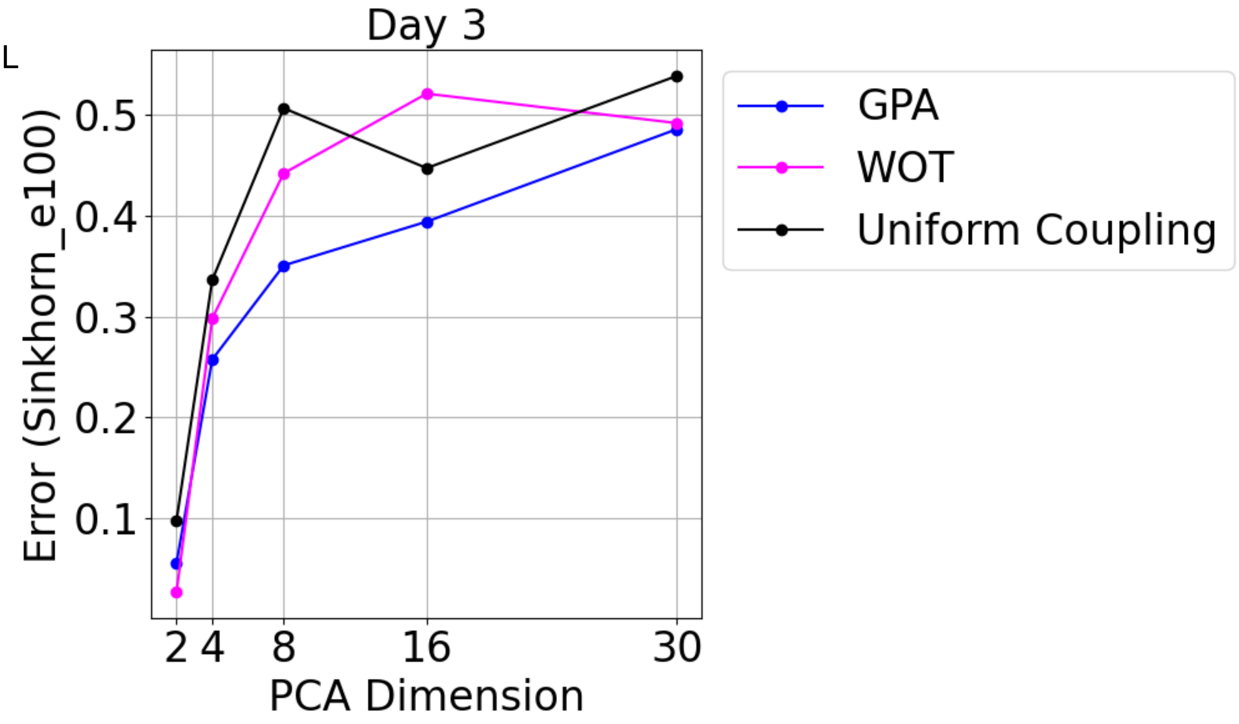
Day 3
Error (Sinkhorn_e100)
0.1
0.2
0.3
0.4
0.5
2 4 8 16 30
PCA Dimension
GPA
WOT
Uniform Coupling

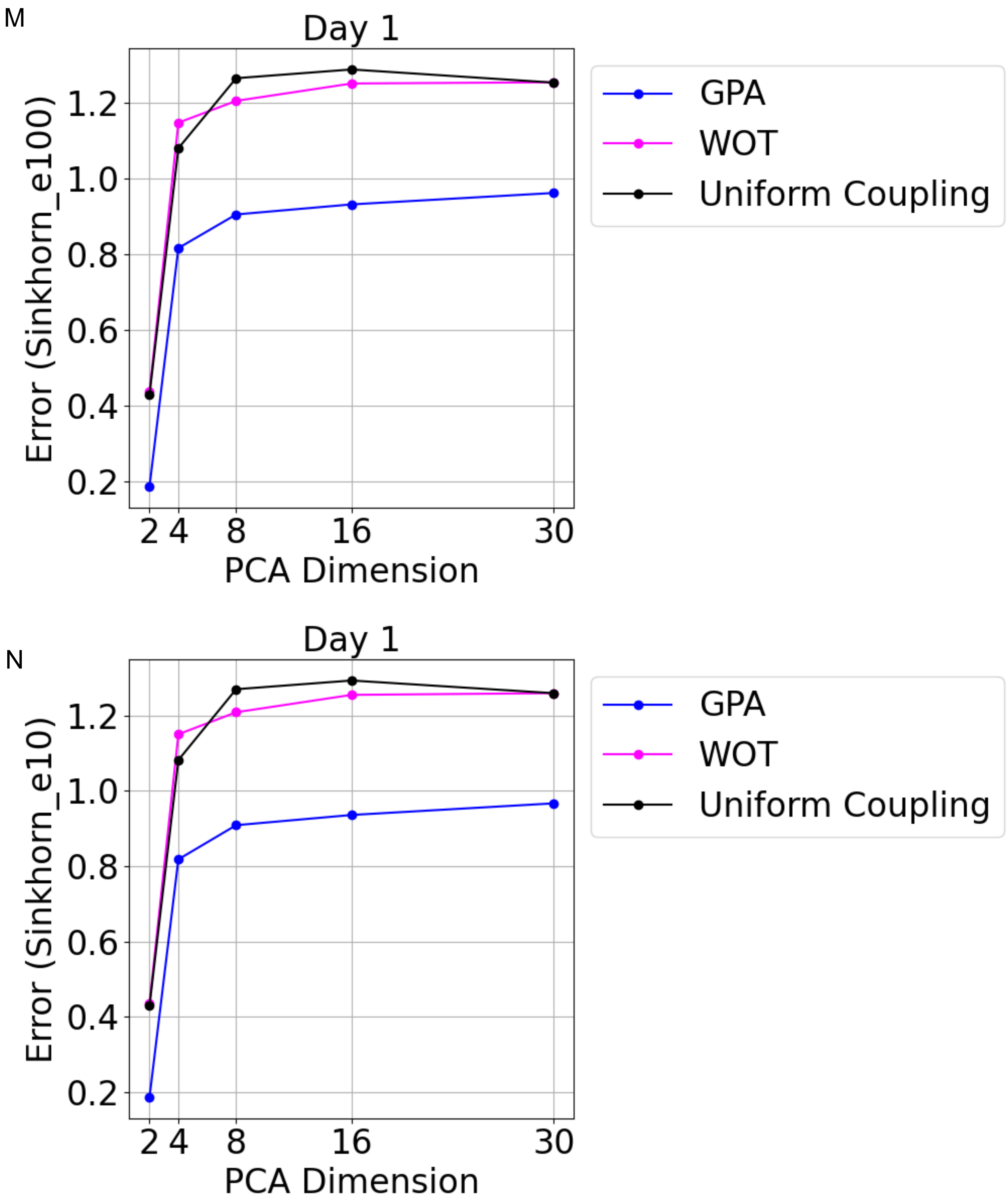

Supplementary Figure 1: Error distances between predicted and test data (Day 1 and Day 3) across different metrics.
