# Supplementary material for: PROFET Predicts Continuous Gene Expression Dynamics from scRNA-seq Data to Elucidate Heterogeneity of Cancer Treatment Responses
Source: bioRxiv. 2025 Jul 3:2025.06.27.662030. Preprint. [Version 1] doi: 10.1101/2025.06.27.662030 (PMC12236938; doi:10.1101/2025.06.27.662030)
Supplement: Supplement 3 [file media-3.pdf]

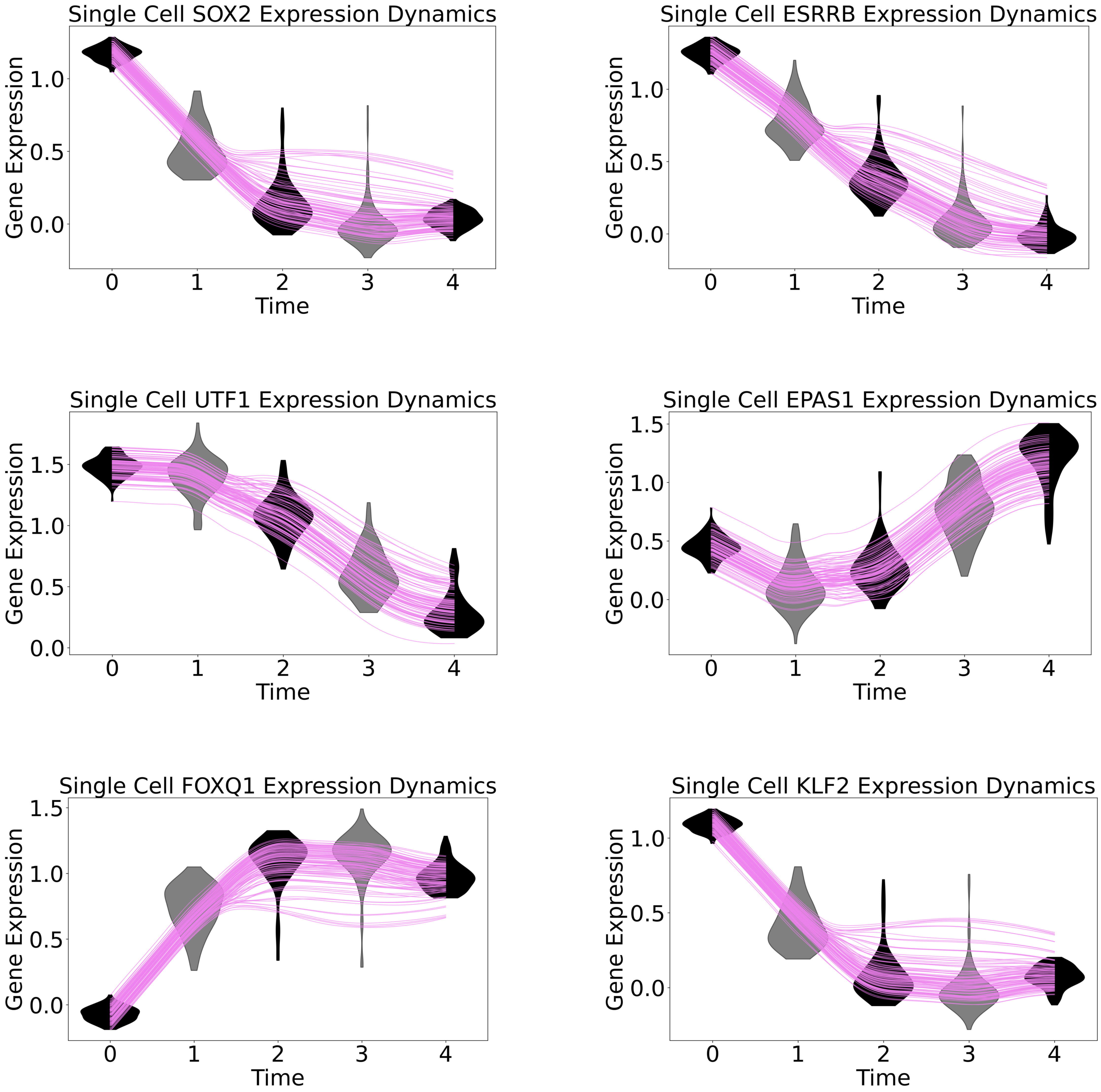
Single Cell SOX2 Expression Dynamics
Single Cell ESRRB Expression Dynamics
Single Cell UTF1 Expression Dynamics
Single Cell EPAS1 Expression Dynamics
Single Cell FOXQ1 Expression Dynamics
Single Cell KLF2 Expression Dynamics
Gene Expression
Time
0
1
2
3
4
0.0
0.5
1.0
1.5

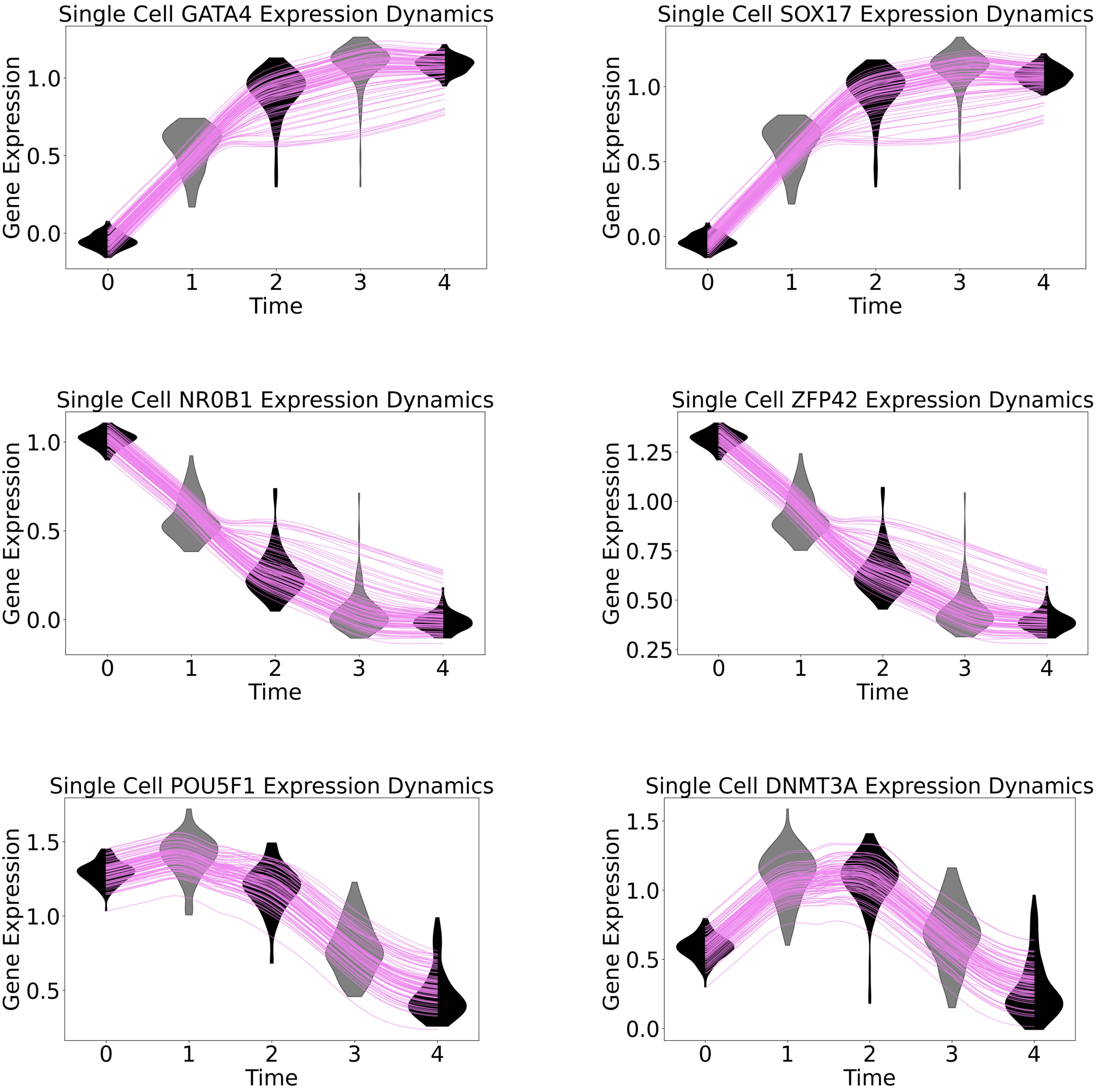
Single Cell GATA4 Expression Dynamics
Gene Expression
0.0
0.5
1.0
Time
0
1
2
3
4
Single Cell SOX17 Expression Dynamics
Gene Expression
0.0
0.5
1.0
Time
0
1
2
3
4
Single Cell NR0B1 Expression Dynamics
Gene Expression
0.0
0.5
1.0
Time
0
1
2
3
4
Single Cell ZFP42 Expression Dynamics
Gene Expression
0.25
0.50
0.75
1.00
1.25
Time
0
1
2
3
4
Single Cell POU5F1 Expression Dynamics
Gene Expression
0.5
1.0
1.5
Time
0
1
2
3
4
Single Cell DNMT3A Expression Dynamics
Gene Expression
0.0
0.5
1.0
1.5
Time
0
1
2
3
4

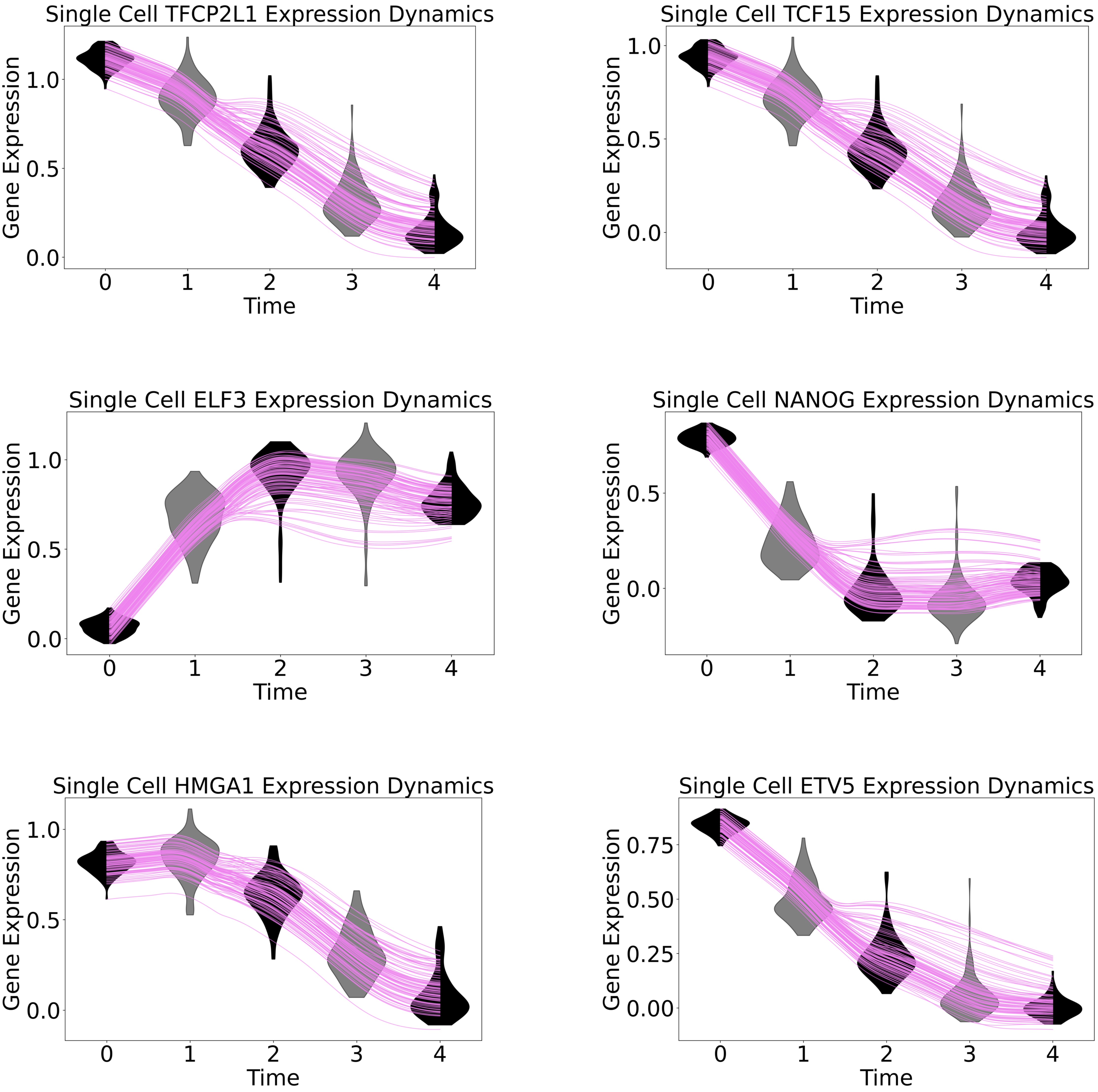
Single Cell TFCP2L1 Expression Dynamics
Single Cell TCF15 Expression Dynamics
Single Cell ELF3 Expression Dynamics
Single Cell NANOG Expression Dynamics
Single Cell HMGA1 Expression Dynamics
Single Cell ETV5 Expression Dynamics
Gene Expression
Time
0.0
0.5
1.0
0.25
0.50
0.75
0.00
0
1
2
3
4

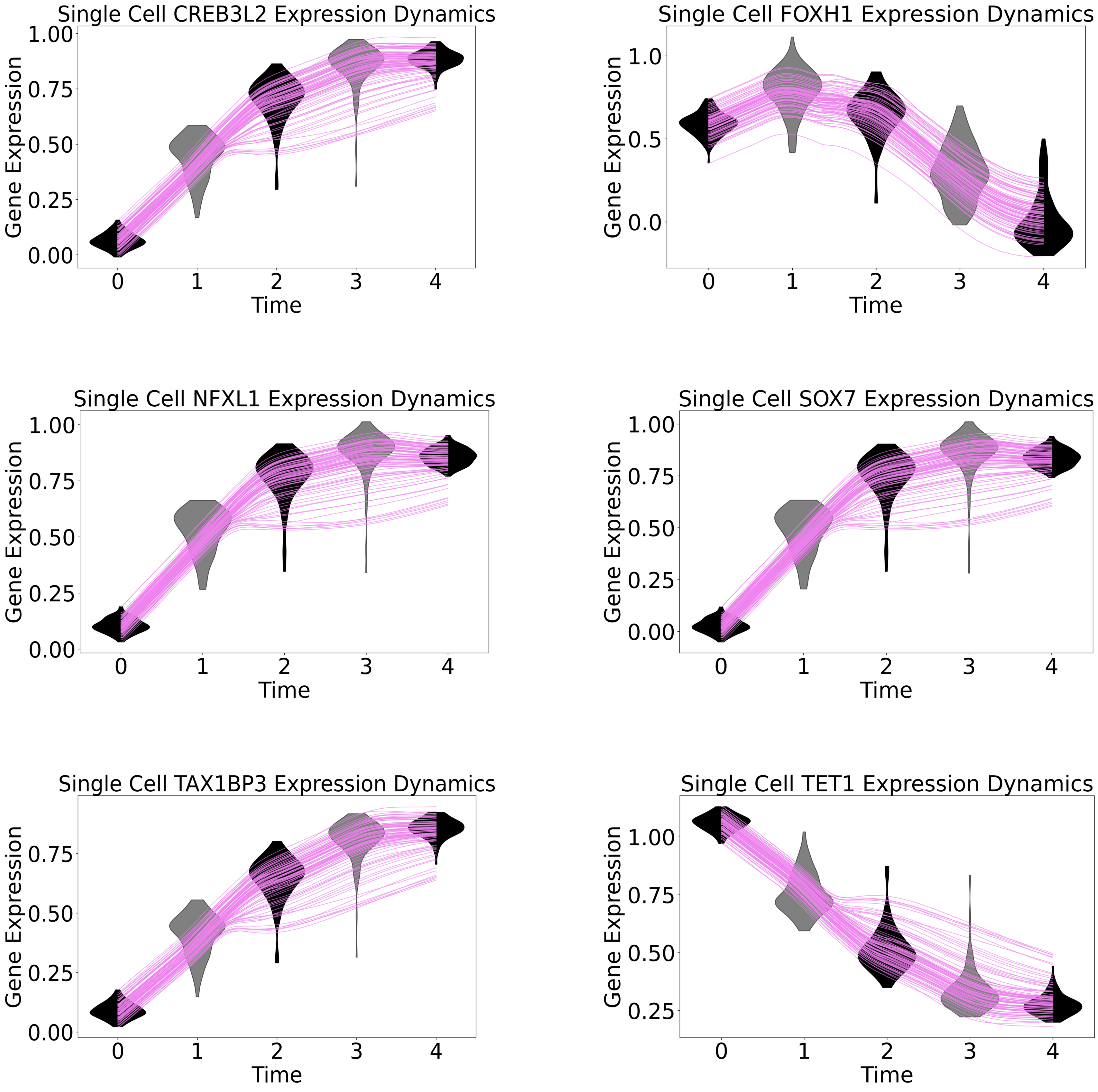

Single Cell CREB3L2 Expression Dynamics
Single Cell FOXH1 Expression Dynamics
Single Cell NFXL1 Expression Dynamics
Single Cell SOX7 Expression Dynamics
Single Cell TAX1BP3 Expression Dynamics
Single Cell TET1 Expression Dynamics
Gene Expression
Time
0
1
2
3
4
0.00
0.25
0.50
0.75
1.00
0.0
0.5
1.0

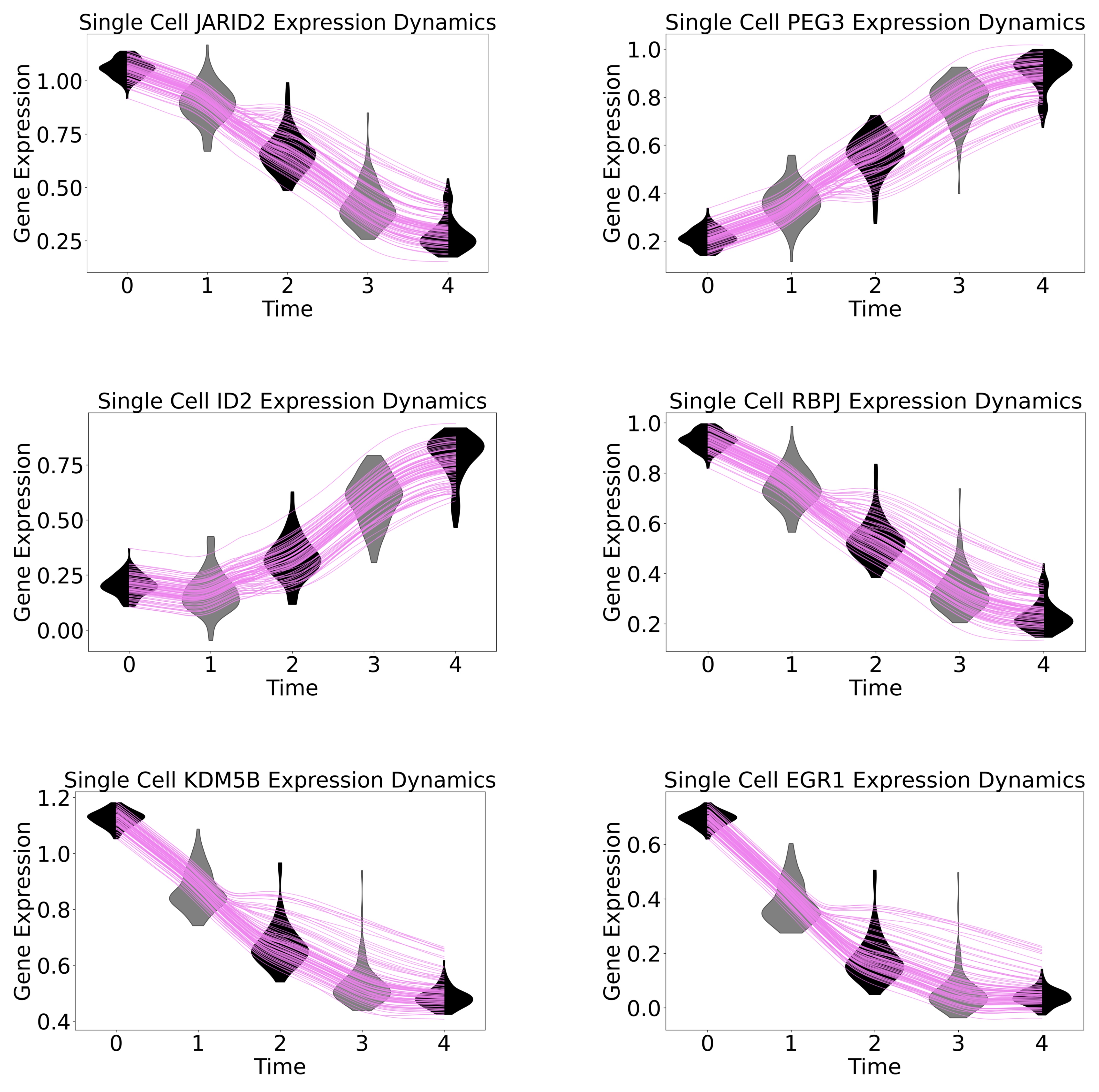

Single Cell JARID2 Expression Dynamics
Gene Expression
1.00
0.75
0.50
0.25
0
1
2
3
4
Time
Single Cell PEG3 Expression Dynamics
1.0
0.8
0.6
0.4
0.2
Single Cell ID2 Expression Dynamics
0.75
0.50
0.25
0.00
Single Cell RBPJ Expression Dynamics
Single Cell KDM5B Expression Dynamics
1.2
1.0
0.8
0.6
0.4
Single Cell EGR1 Expression Dynamics
0.6
0.4
0.2
0.0

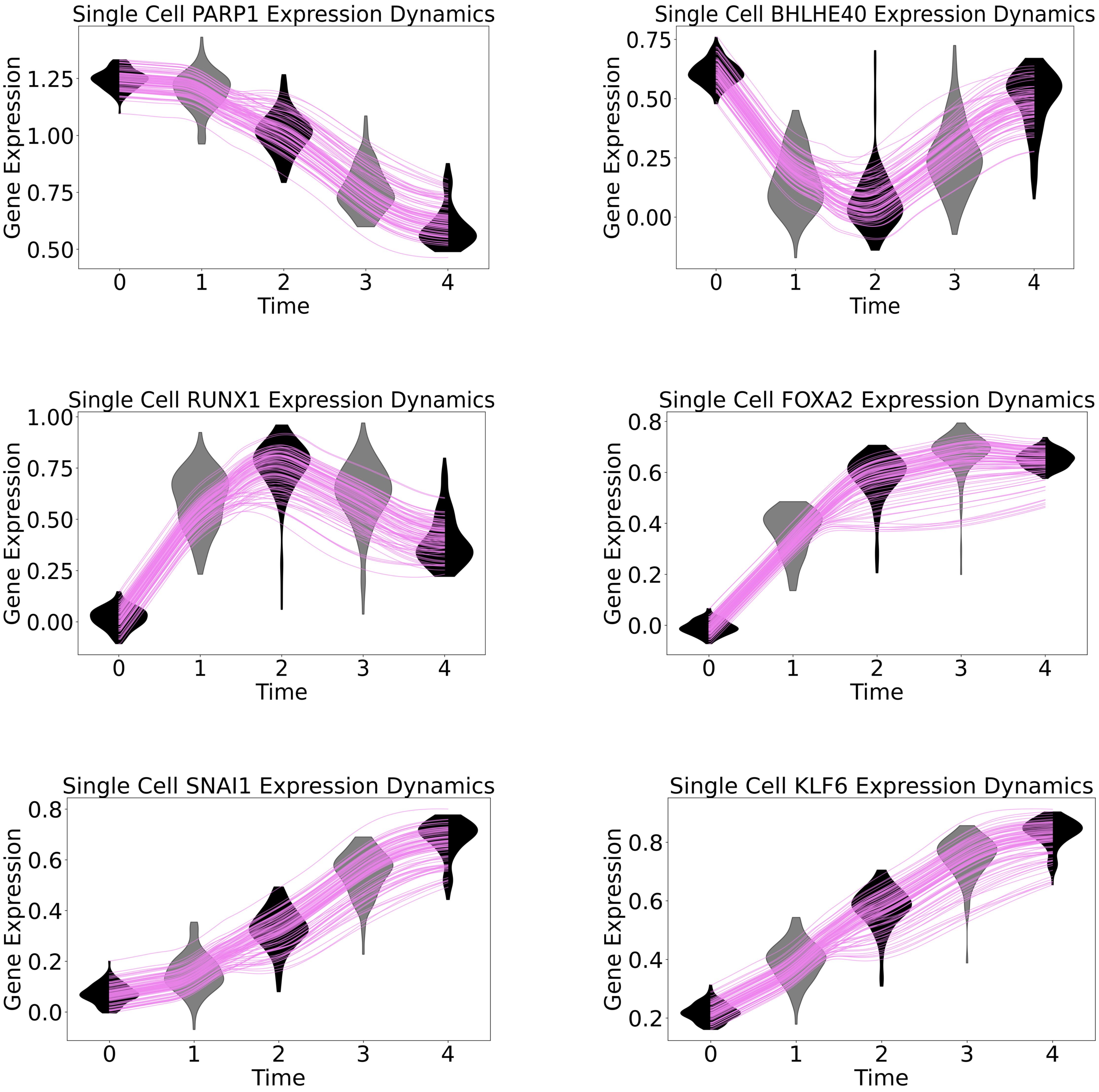
Single Cell PARP1 Expression Dynamics
Gene Expression
1.25
1.00
0.75
0.50
0
1
2
3
4
Time
Single Cell BHLHE40 Expression Dynamics
Gene Expression
0.75
0.50
0.25
0.00
0
1
2
3
4
Time
Single Cell RUNX1 Expression Dynamics
Gene Expression
1.00
0.75
0.50
0.25
0.00
0
1
2
3
4
Time
Single Cell FOXA2 Expression Dynamics
Gene Expression
0.8
0.6
0.4
0.2
0.0
0
1
2
3
4
Time
Single Cell SNAI1 Expression Dynamics
Gene Expression
0.8
0.6
0.4
0.2
0.0
0
1
2
3
4
Time
Single Cell KLF6 Expression Dynamics
Gene Expression
0.8
0.6
0.4
0.2
0
1
2
3
4
Time

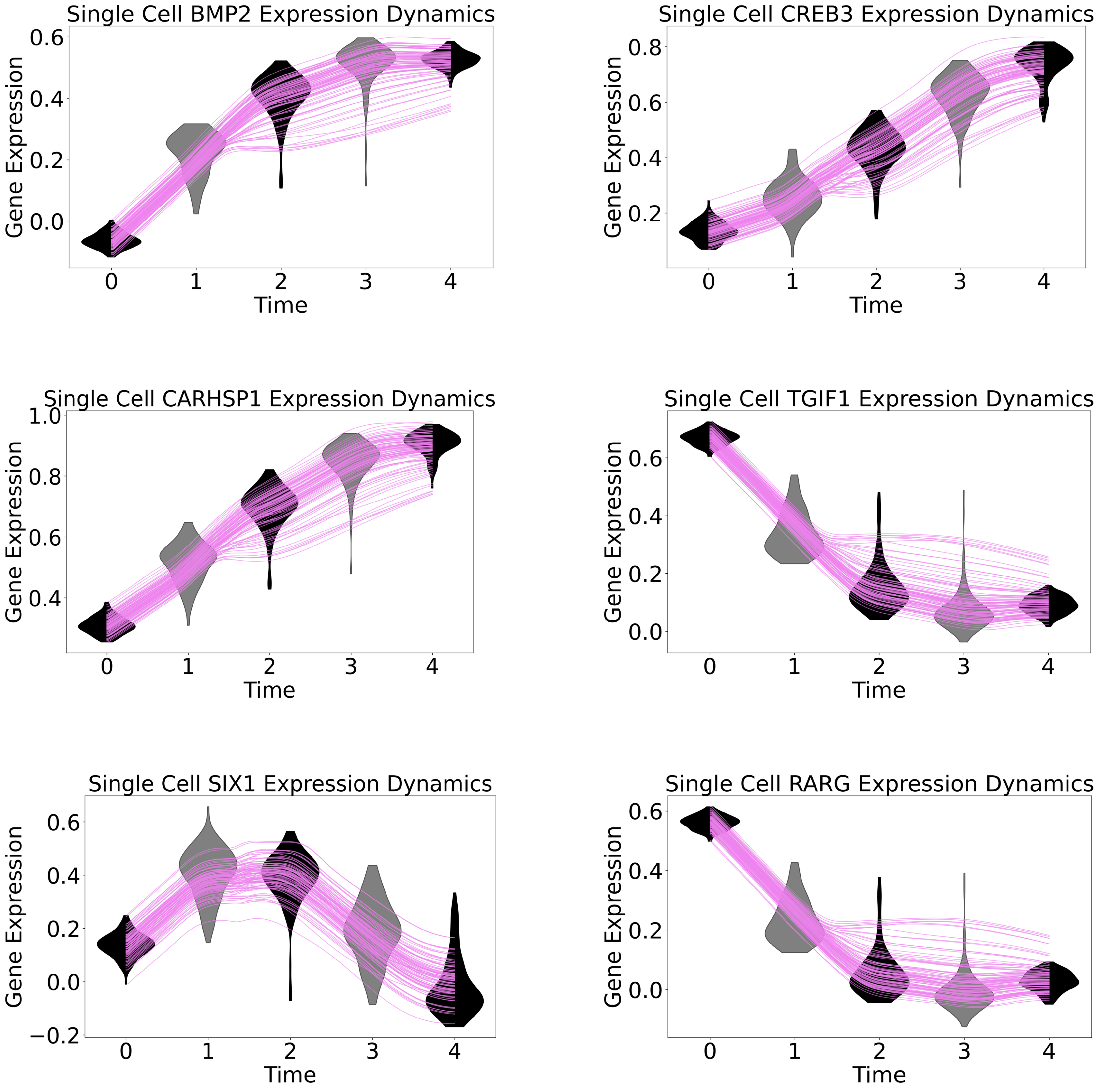
Single Cell BMP2 Expression Dynamics
Single Cell CREB3 Expression Dynamics
Single Cell CARHSP1 Expression Dynamics
Single Cell TGIF1 Expression Dynamics
Single Cell SIX1 Expression Dynamics
Single Cell RARG Expression Dynamics
Gene Expression
Time
0
1
2
3
4

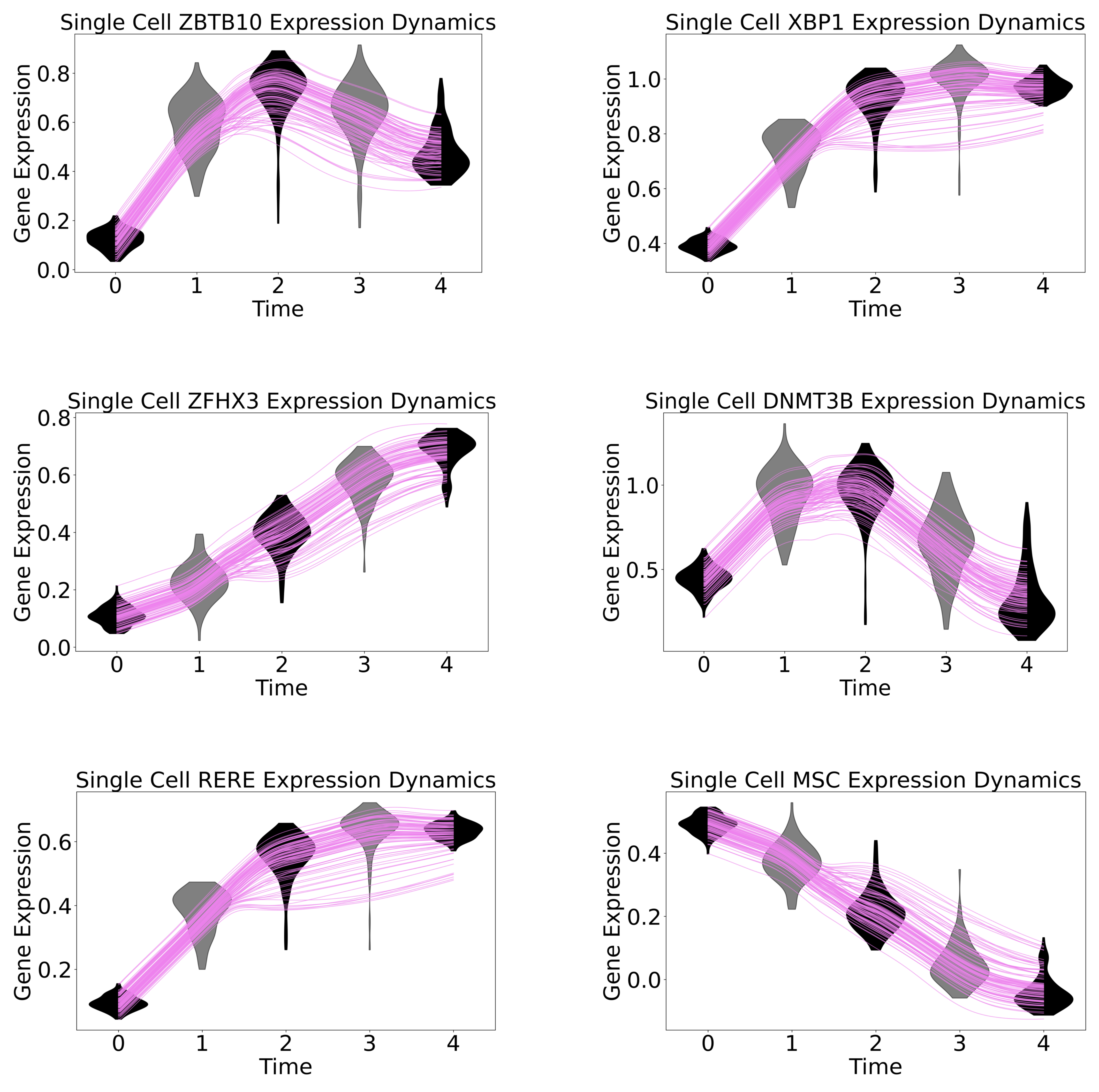

Single Cell ZBTB10 Expression Dynamics
Single Cell XBP1 Expression Dynamics
Single Cell ZFHX3 Expression Dynamics
Single Cell DNMT3B Expression Dynamics
Single Cell RERE Expression Dynamics
Single Cell MSC Expression Dynamics
Gene Expression
Time

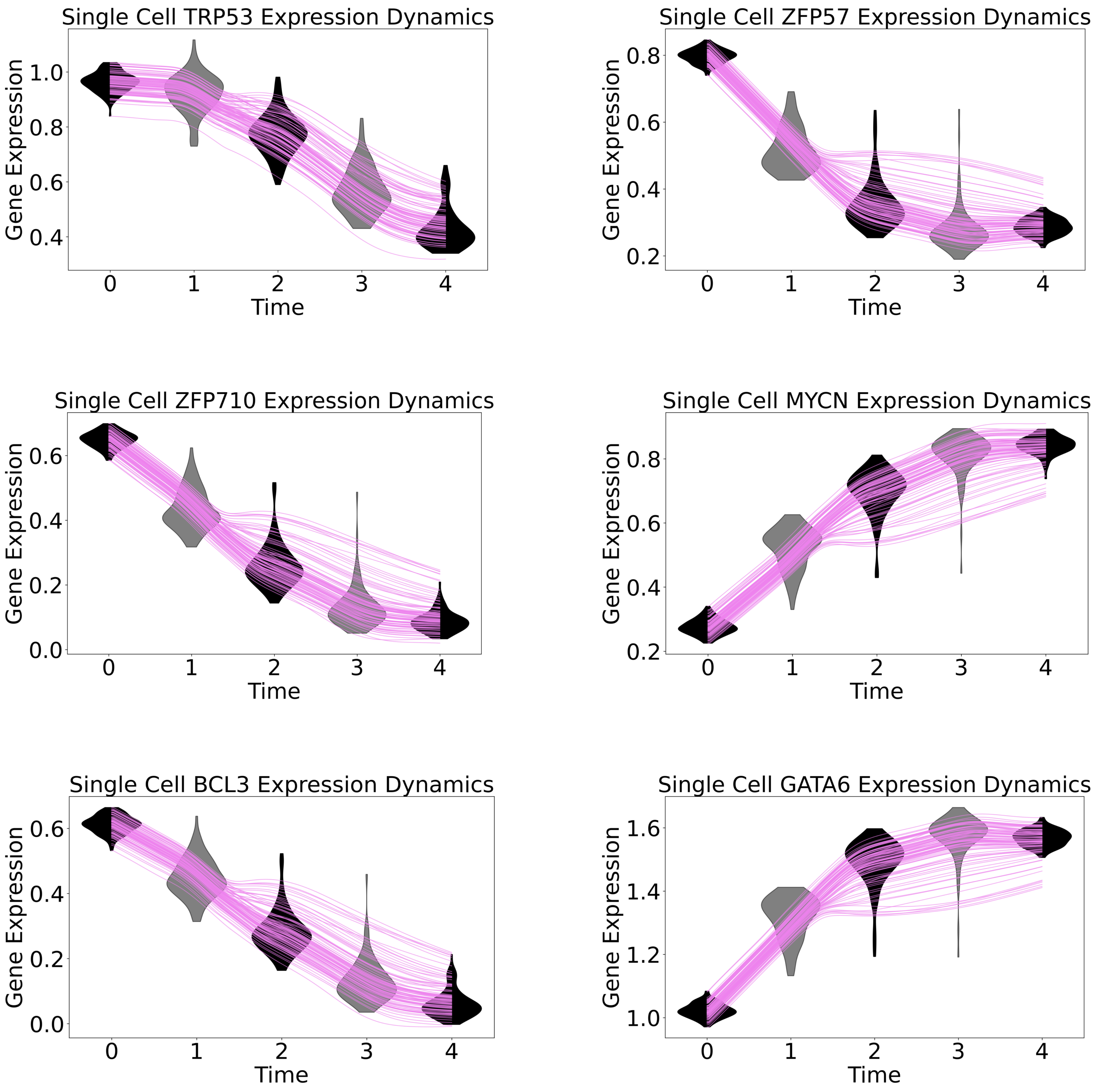
Single Cell TRP53 Expression Dynamics
Gene Expression
1.0
0.8
0.6
0.4
0
1
2
3
4
Time
Single Cell ZFP57 Expression Dynamics
Gene Expression
0.8
0.6
0.4
0.2
0
1
2
3
4
Time
Single Cell ZFP710 Expression Dynamics
Gene Expression
0.6
0.4
0.2
0.0
0
1
2
3
4
Time
Single Cell MYCN Expression Dynamics
Gene Expression
0.8
0.6
0.4
0.2
0
1
2
3
4
Time
Single Cell BCL3 Expression Dynamics
Gene Expression
0.6
0.4
0.2
0.0
0
1
2
3
4
Time
Single Cell GATA6 Expression Dynamics
Gene Expression
1.6
1.4
1.2
1.0
0
1
2
3
4
Time

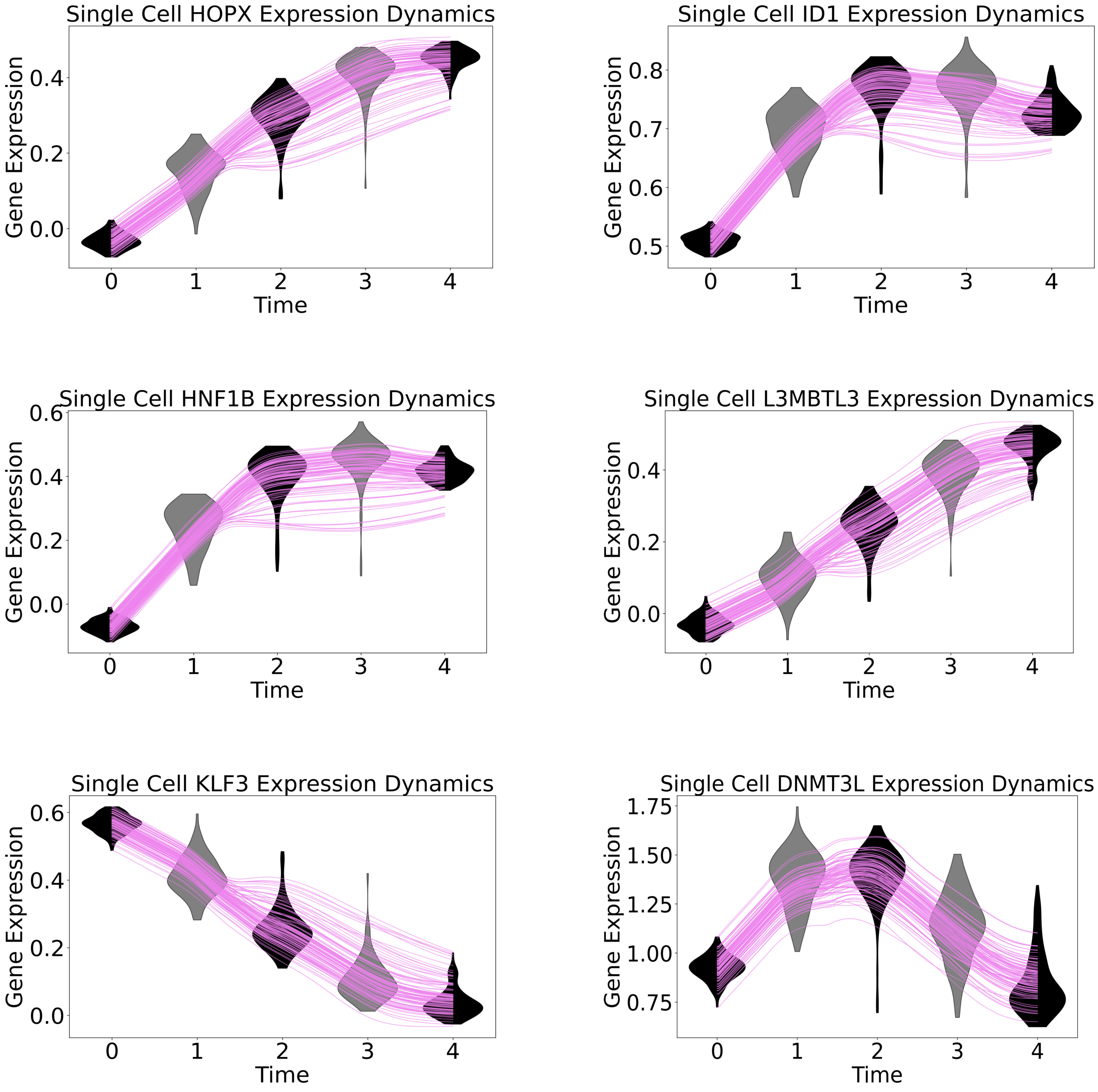
Single Cell HOPX Expression Dynamics
Single Cell ID1 Expression Dynamics
Single Cell HNF1B Expression Dynamics
Single Cell L3MBTL3 Expression Dynamics
Single Cell KLF3 Expression Dynamics
Single Cell DNMT3L Expression Dynamics
Gene Expression
Time
0.0
0.2
0.4
0.5
0.6
0.7
0.8
0.75
1.00
1.25
1.50
1.75
0
1
2
3
4

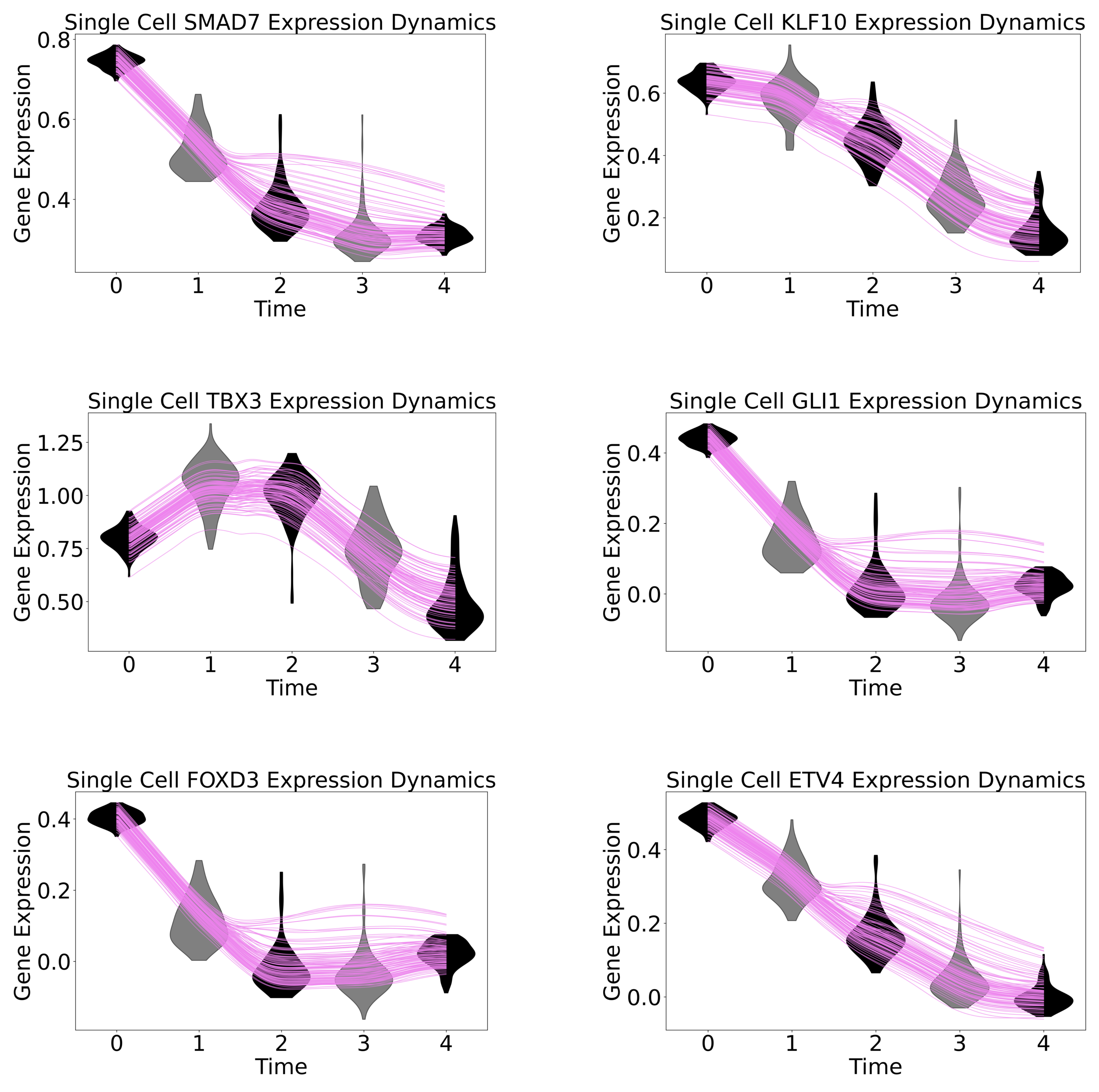

Single Cell SMAD7 Expression Dynamics
Gene Expression
0.8
0.6
0.4
Time
0 1 2 3 4
Single Cell KLF10 Expression Dynamics
Gene Expression
0.6
0.4
0.2
Time
0 1 2 3 4
Single Cell TBX3 Expression Dynamics
Gene Expression
1.25
1.00
0.75
0.50
Time
0 1 2 3 4
Single Cell GLI1 Expression Dynamics
Gene Expression
0.4
0.2
0.0
Time
0 1 2 3 4
Single Cell FOXD3 Expression Dynamics
Gene Expression
0.4
0.2
0.0
Time
0 1 2 3 4
Single Cell ETV4 Expression Dynamics
Gene Expression
0.4
0.2
0.0
Time
0 1 2 3 4

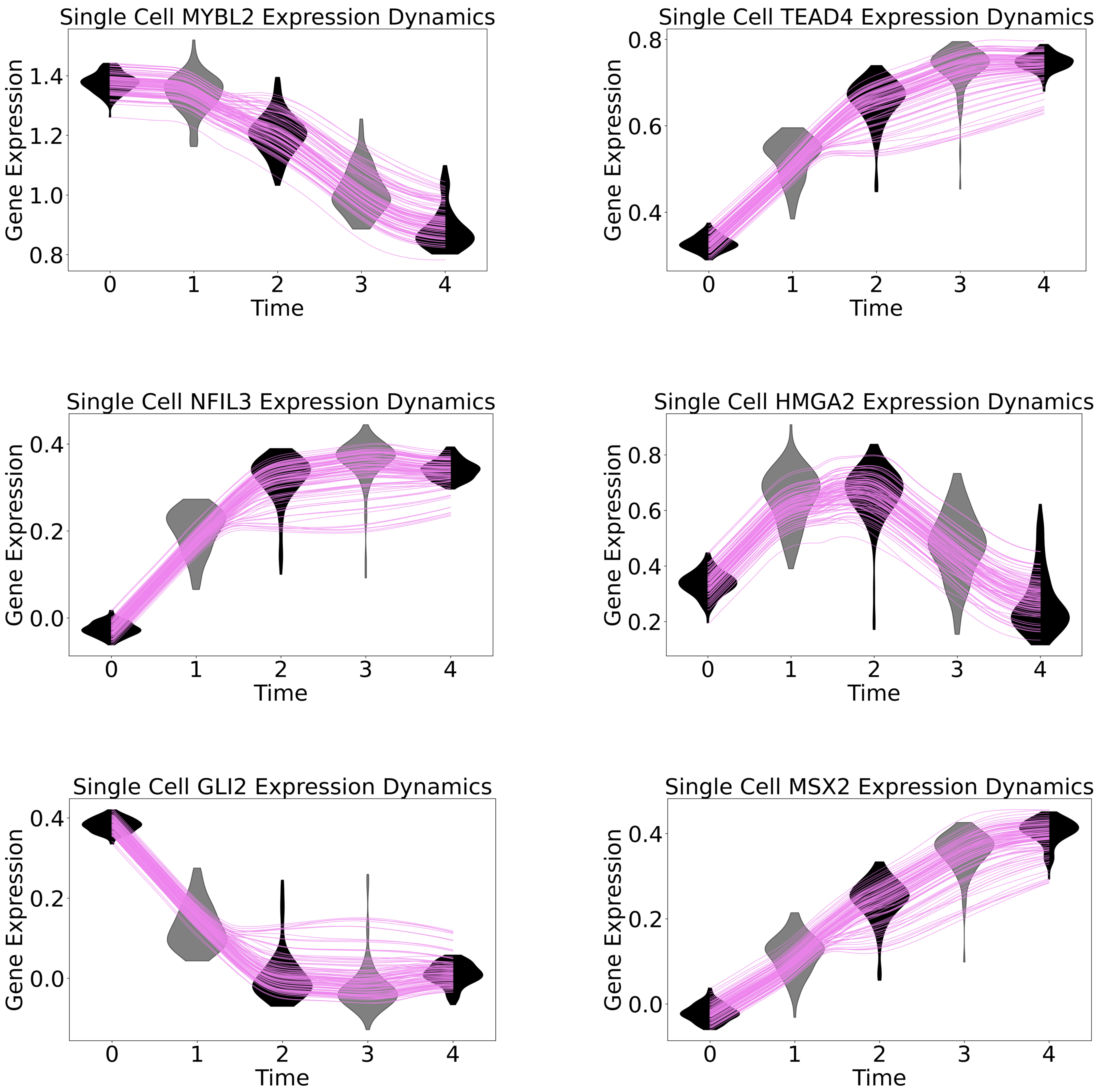

Single Cell MYBL2 Expression Dynamics
Single Cell TEAD4 Expression Dynamics
Single Cell NFIL3 Expression Dynamics
Single Cell HMGA2 Expression Dynamics
Single Cell GLI2 Expression Dynamics
Single Cell MSX2 Expression Dynamics
Gene Expression
Time
0
1
2
3
4

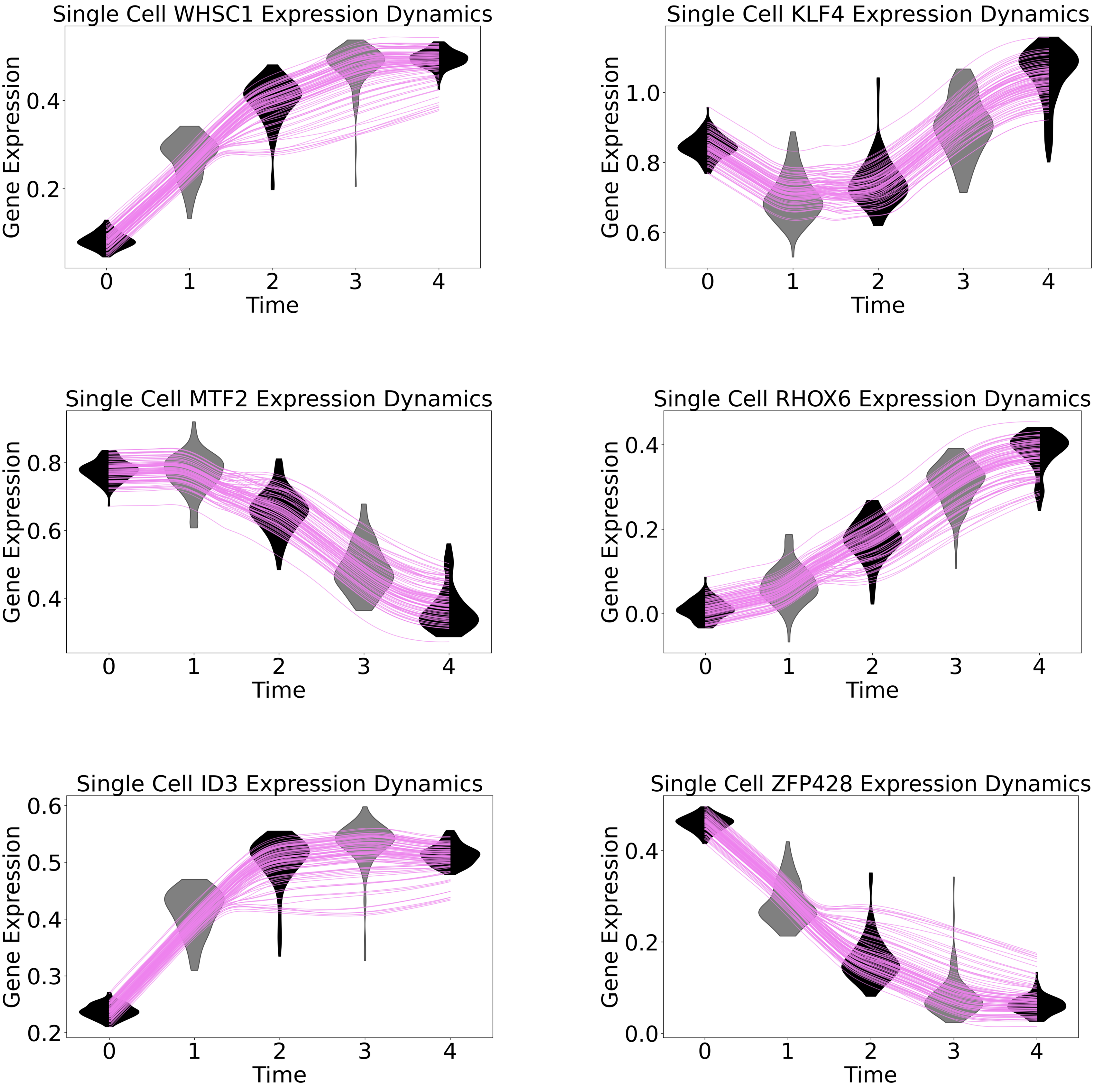

Single Cell WHSC1 Expression Dynamics
Gene Expression
0.2
0.4
Time
0
1
2
3
4
Single Cell KLF4 Expression Dynamics
Gene Expression
0.6
0.8
1.0
Time
0
1
2
3
4
Single Cell MTF2 Expression Dynamics
Gene Expression
0.4
0.6
0.8
Time
0
1
2
3
4
Single Cell RHOX6 Expression Dynamics
Gene Expression
0.0
0.2
0.4
Time
0
1
2
3
4
Single Cell ID3 Expression Dynamics
Gene Expression
0.2
0.3
0.4
0.5
0.6
Time
0
1
2
3
4
Single Cell ZFP428 Expression Dynamics
Gene Expression
0.0
0.2
0.4
Time
0
1
2
3
4

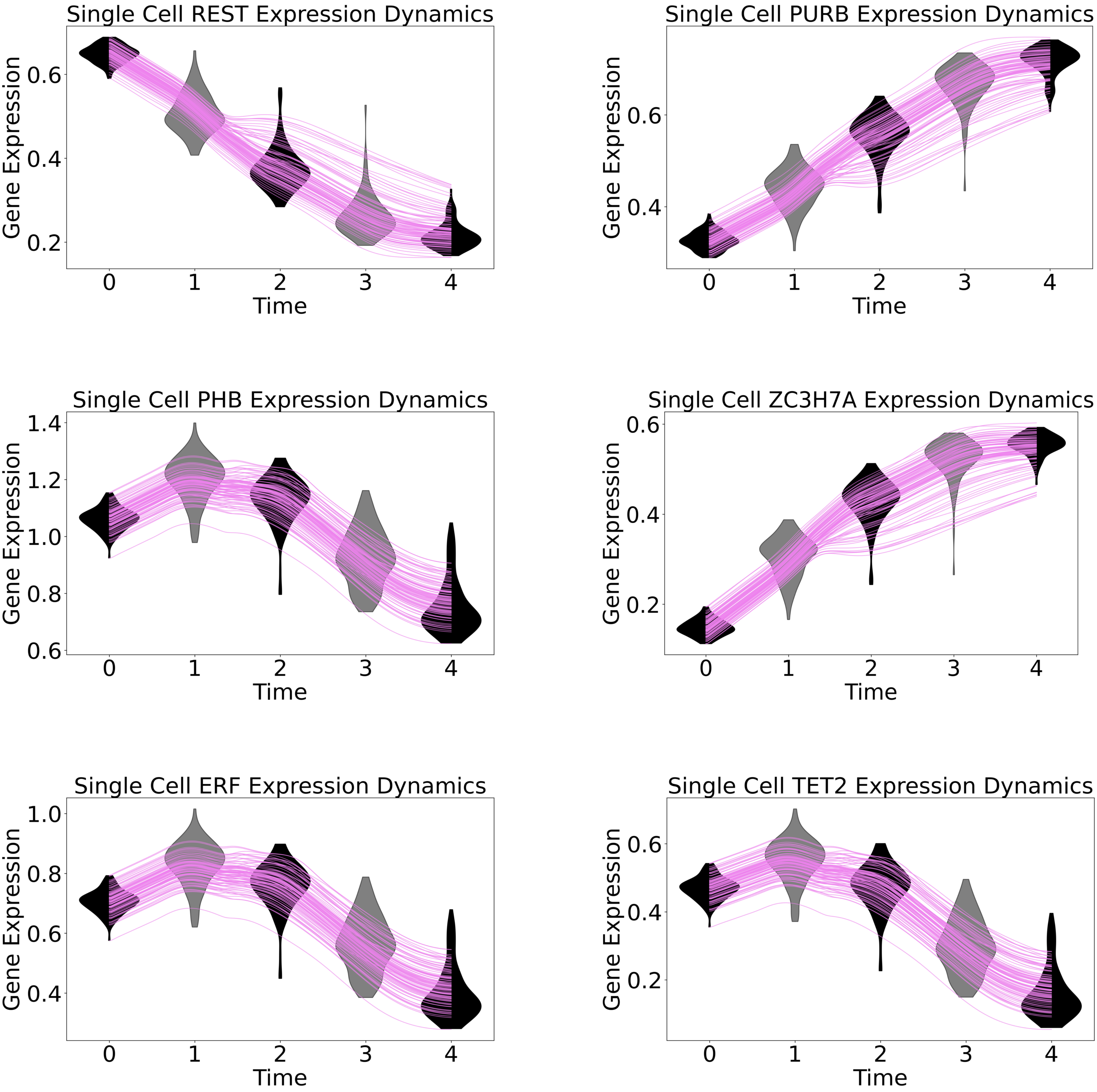
Single Cell REST Expression Dynamics
Single Cell PURB Expression Dynamics
Single Cell PHB Expression Dynamics
Single Cell ZC3H7A Expression Dynamics
Single Cell ERF Expression Dynamics
Single Cell TET2 Expression Dynamics
Gene Expression
Time

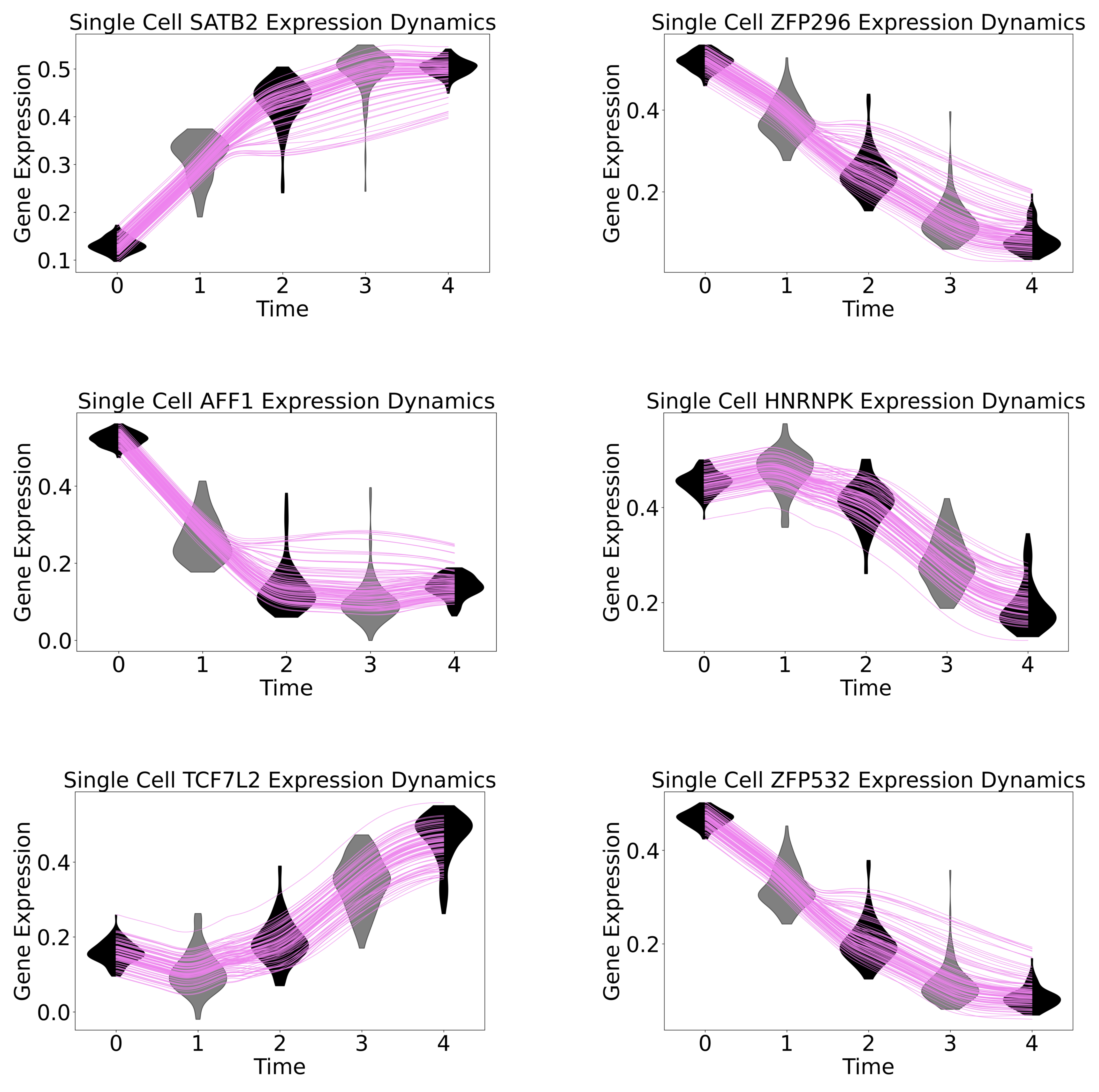

Single Cell SATB2 Expression Dynamics
Gene Expression
0.1
0.2
0.3
0.4
0.5
Time
0 1 2 3 4
Single Cell ZFP296 Expression Dynamics
Gene Expression
0.2
0.4
Time
0 1 2 3 4
Single Cell AFF1 Expression Dynamics
Gene Expression
0.0
0.2
0.4
Time
0 1 2 3 4
Single Cell HNRNPK Expression Dynamics
Gene Expression
0.2
0.4
Time
0 1 2 3 4
Single Cell TCF7L2 Expression Dynamics
Gene Expression
0.0
0.2
0.4
Time
0 1 2 3 4
Single Cell ZFP532 Expression Dynamics
Gene Expression
0.2
0.4
Time
0 1 2 3 4

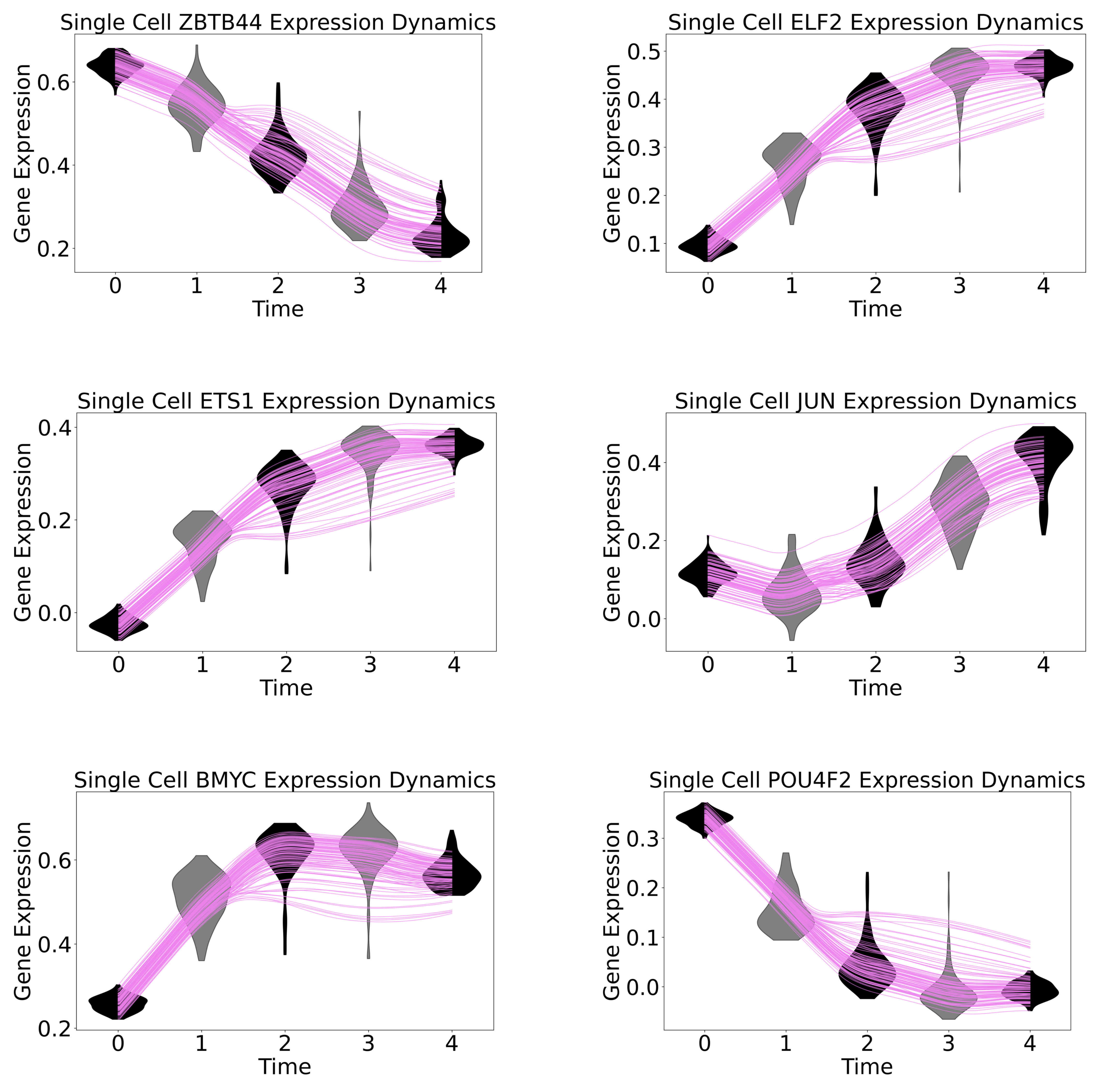
Single Cell ZBTB44 Expression Dynamics
Single Cell ELF2 Expression Dynamics
Single Cell ETS1 Expression Dynamics
Single Cell JUN Expression Dynamics
Single Cell BMYC Expression Dynamics
Single Cell POU4F2 Expression Dynamics
Gene Expression
Time

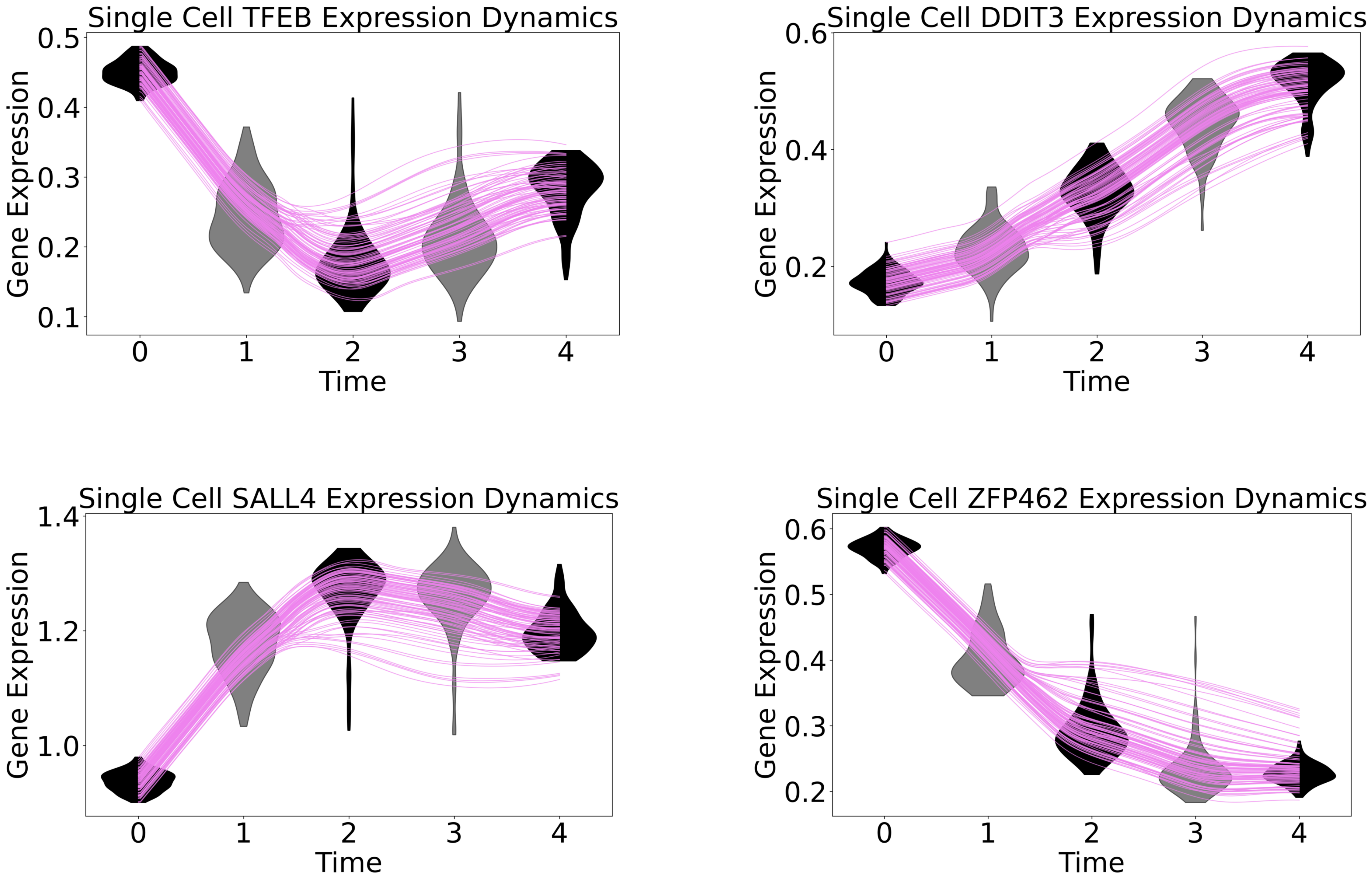

Supplementary Figure 2: Single-cell, single-gene dynamics plotted as gene expression levels (y-axis) over time (x-axis), with each green curve representing an individual cell trajectory. The predicted trajectories are compared against real data distributions using violin plots (gray for test data, black for training data).
