# Supplementary material for: PROFET Predicts Continuous Gene Expression Dynamics from scRNA-seq Data to Elucidate Heterogeneity of Cancer Treatment Responses
Source: bioRxiv. 2025 Jul 3:2025.06.27.662030. Preprint. [Version 1] doi: 10.1101/2025.06.27.662030 (PMC12236938; doi:10.1101/2025.06.27.662030)
Supplement: Supplement 4 [file media-5.pdf]

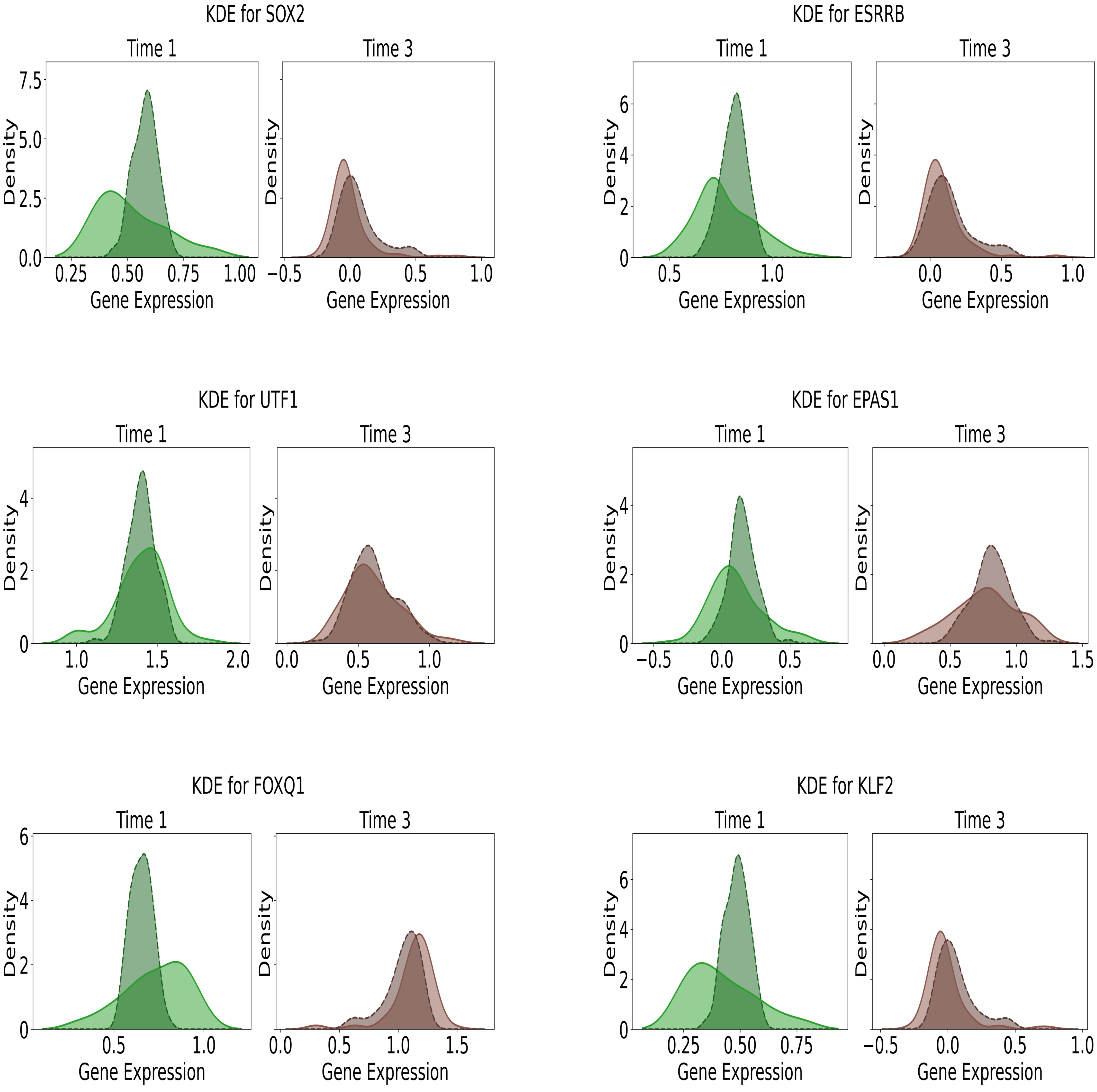
KDE for SOX2
Time 1
Time 3
KDE for ESRRB
Time 1
Time 3
KDE for UTF1
Time 1
Time 3
KDE for EPAS1
Time 1
Time 3
KDE for FOXQ1
Time 1
Time 3
KDE for KLF2
Time 1
Time 3
Density
Gene Expression

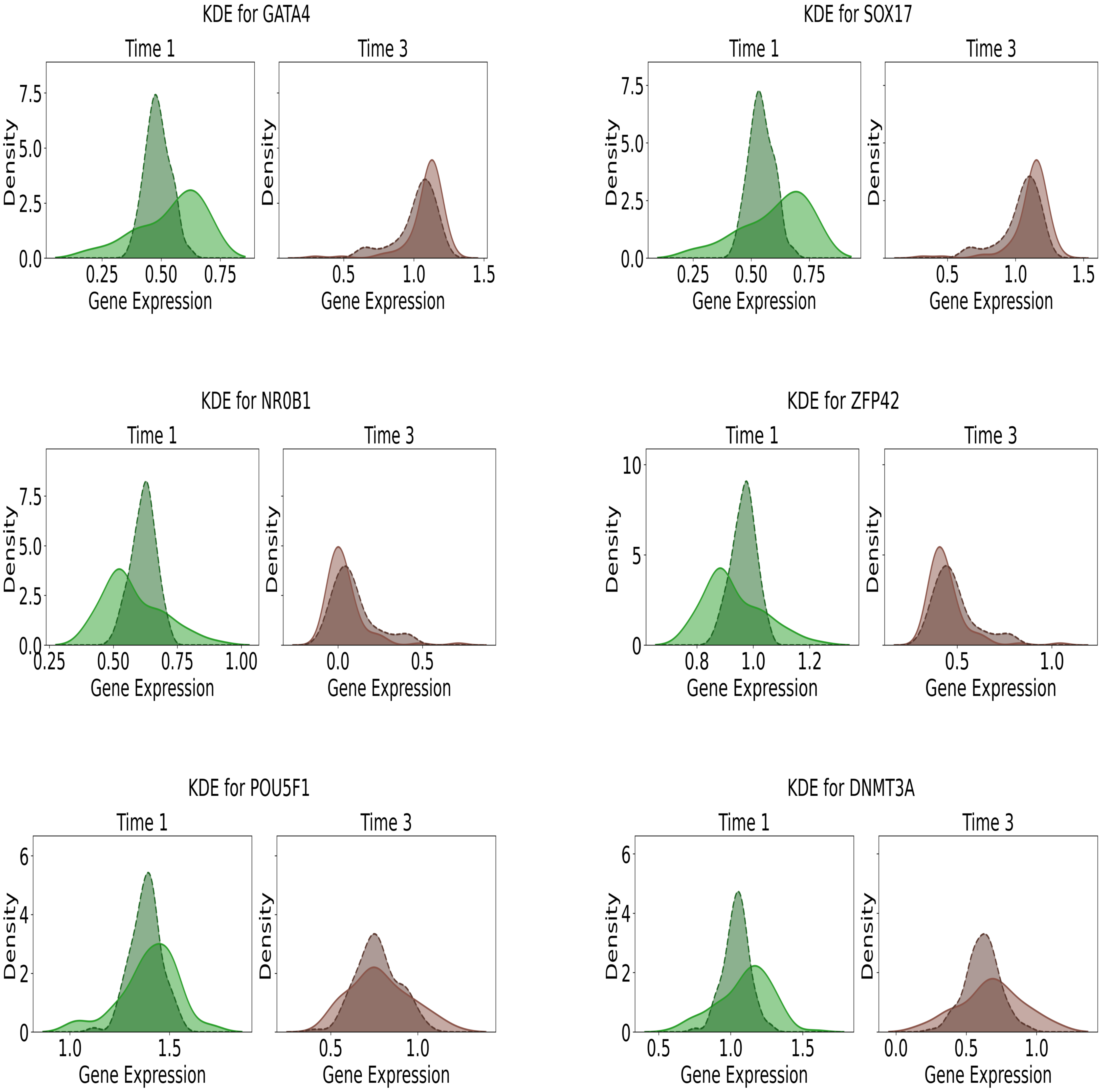

KDE for GATA4
Time 1
Time 3
KDE for SOX17
Time 1
Time 3
KDE for NR0B1
Time 1
Time 3
KDE for ZFP42
Time 1
Time 3
KDE for POU5F1
Time 1
Time 3
KDE for DNMT3A
Time 1
Time 3
Density
Gene Expression

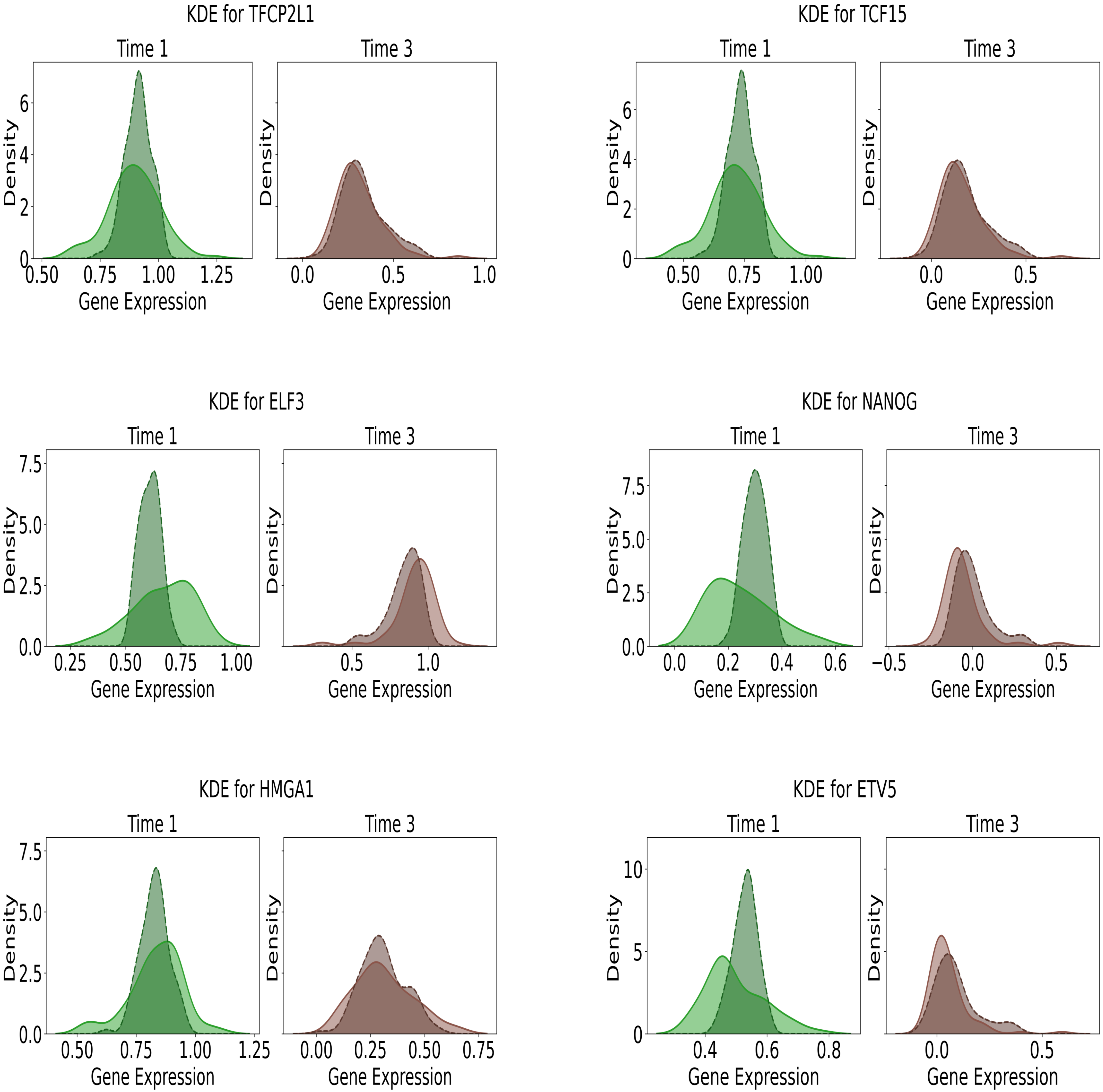
KDE for TFCP2L1
Time 1
Time 3
Density
Gene Expression
KDE for TCF15
Time 1
Time 3
Density
Gene Expression
KDE for ELF3
Time 1
Time 3
Density
Gene Expression
KDE for NANOG
Time 1
Time 3
Density
Gene Expression
KDE for HMGA1
Time 1
Time 3
Density
Gene Expression
KDE for ETV5
Time 1
Time 3
Density
Gene Expression

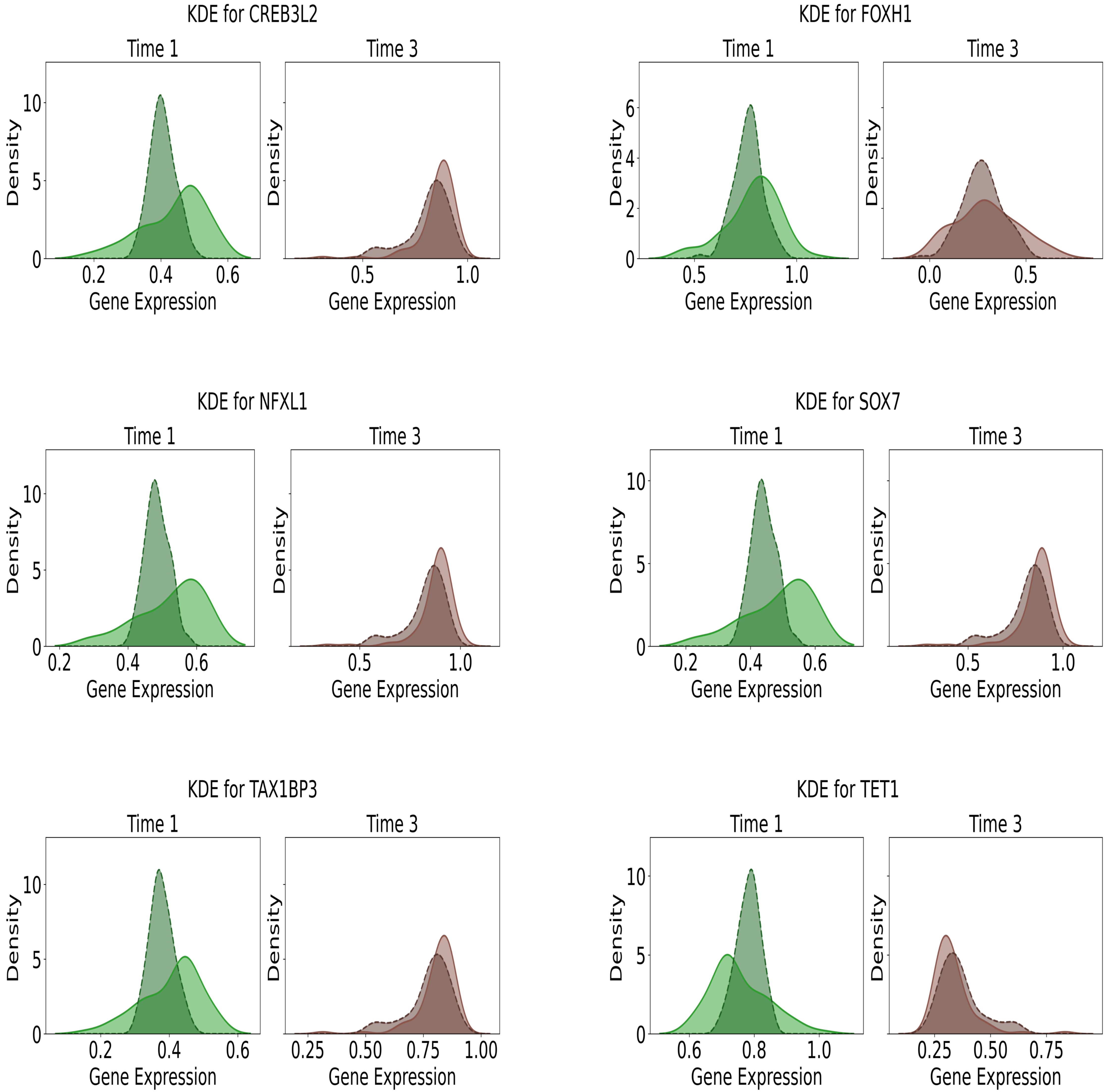

KDE for CREB3L2
KDE for FOXH1
KDE for NFXL1
KDE for SOX7
KDE for TAX1BP3
KDE for TET1
Time 1
Time 3
Density
Gene Expression

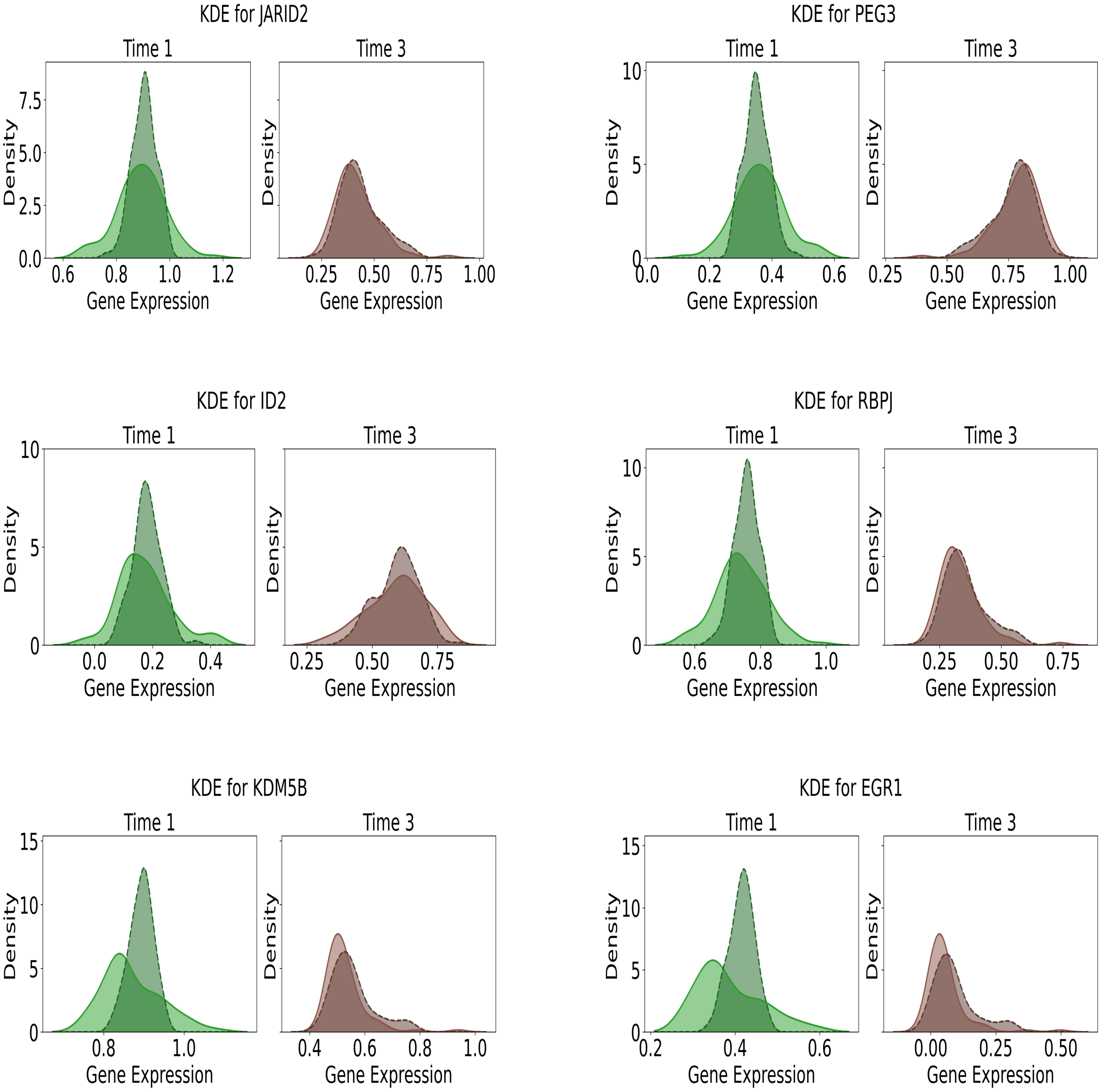

KDE for JARID2
Time 1
Time 3
KDE for PEG3
Time 1
Time 3
KDE for ID2
Time 1
Time 3
KDE for RBPJ
Time 1
Time 3
KDE for KDM5B
Time 1
Time 3
KDE for EGR1
Time 1
Time 3
Density
Gene Expression

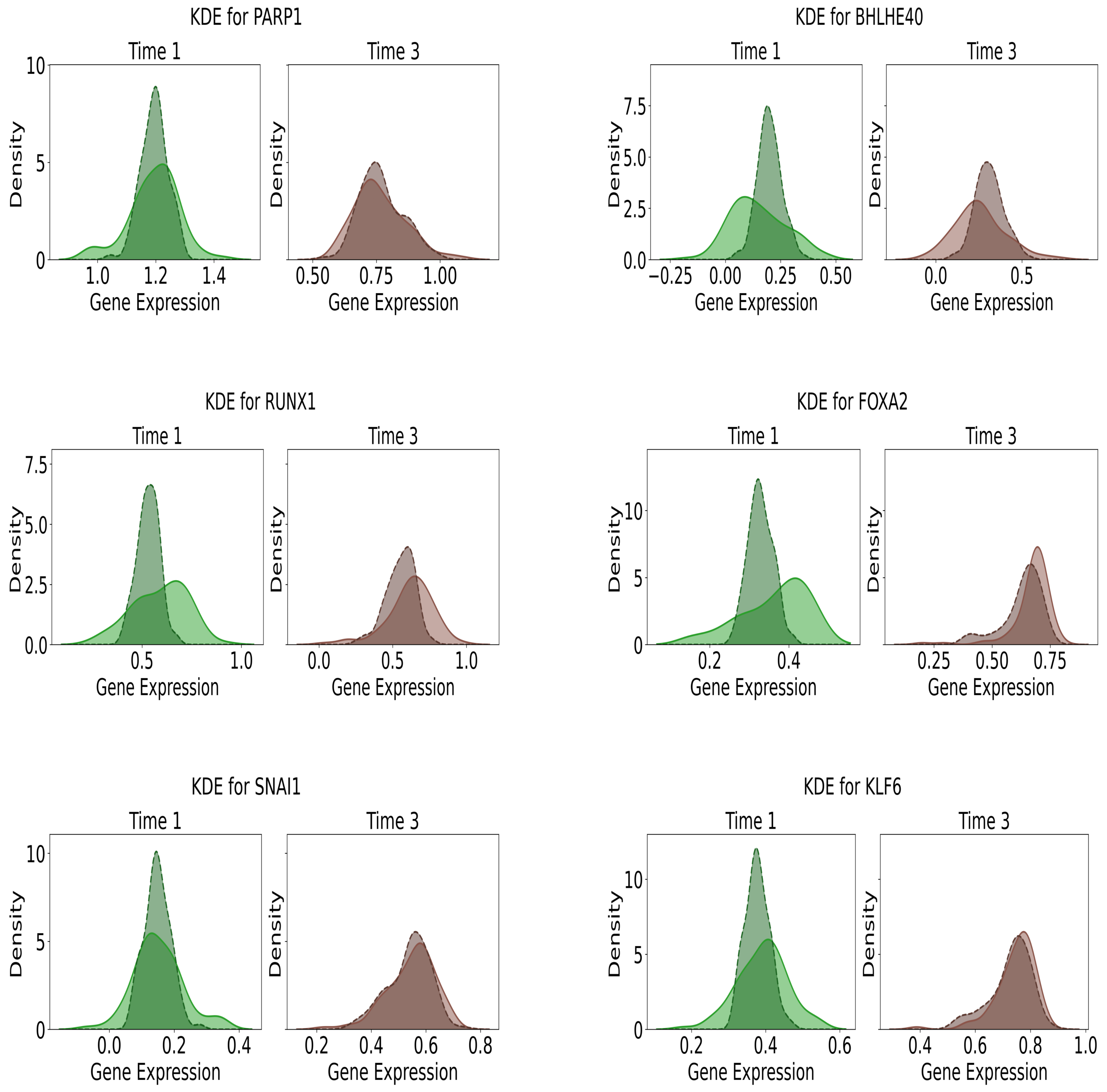
KDE for PARP1
Time 1
Time 3
KDE for BHLHE40
Time 1
Time 3
KDE for RUNX1
Time 1
Time 3
KDE for FOXA2
Time 1
Time 3
KDE for SNAI1
Time 1
Time 3
KDE for KLF6
Time 1
Time 3
Density
Gene Expression
0
5
10
1.0
1.2
1.4
0.50
0.75
1.00
0.0
2.5
5.0
7.5
−0.25
0.00
0.25
0.50
0.0
0.5
1.0
0.2
0.4
0.25
0.50
0.75
0.6
0.8

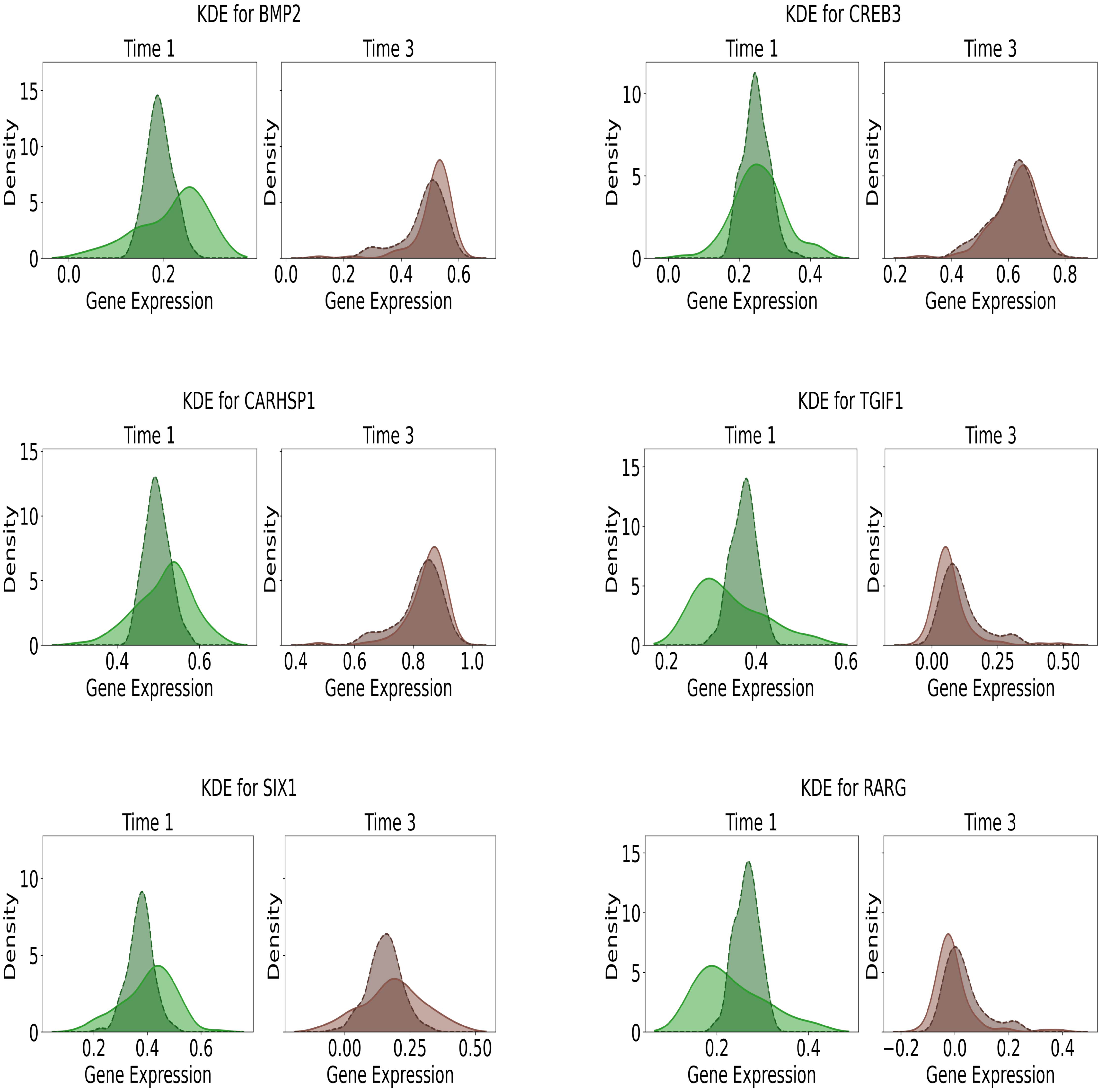
KDE for BMP2
Time 1
Time 3
Density
Gene Expression
0
5
10
15
0.0
0.2
0.4
0.6
KDE for CREB3
0.8
KDE for CARHSP1
1.0
KDE for TGIF1
0.00
0.25
0.50
KDE for SIX1
KDE for RARG
−0.2

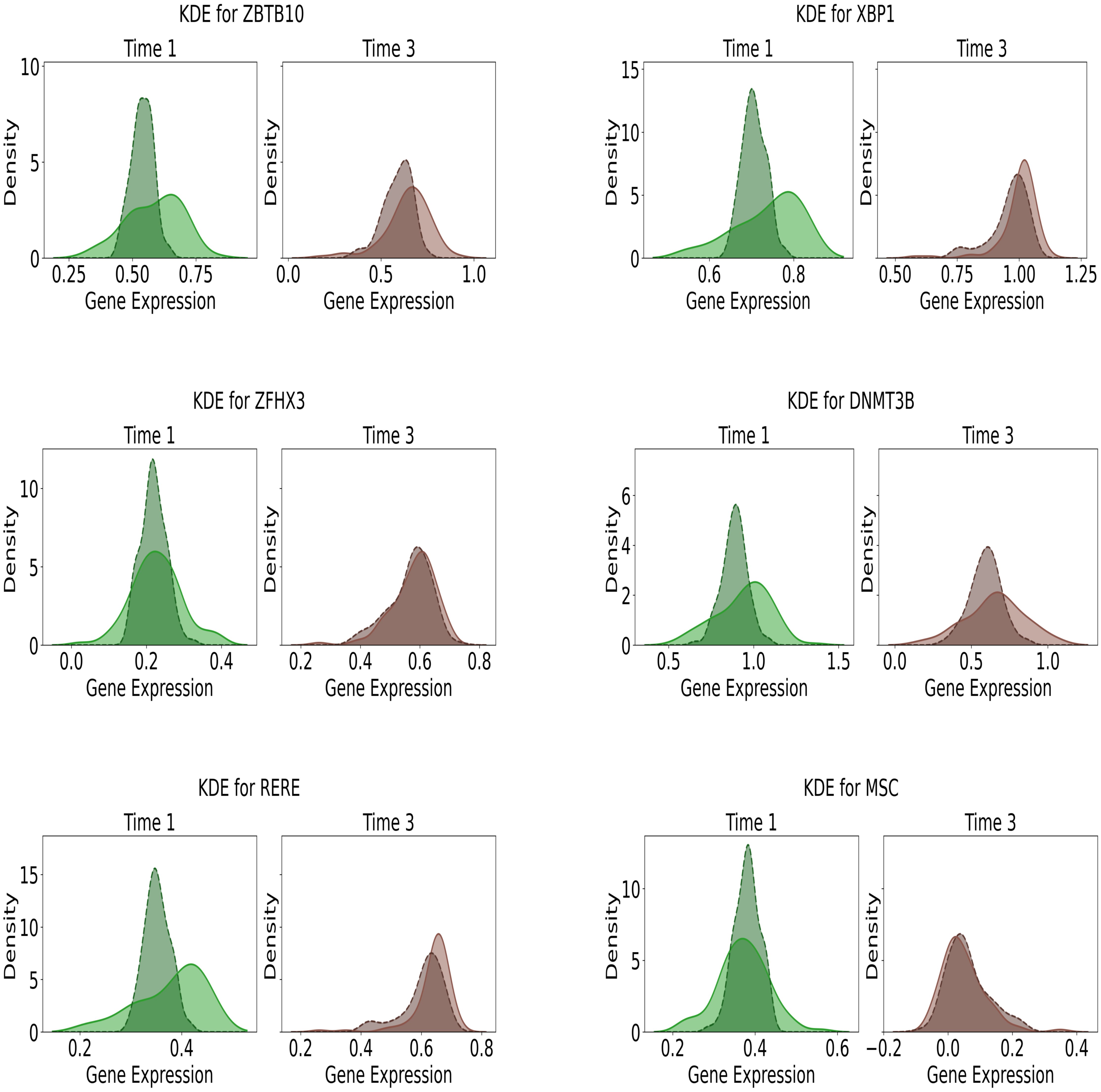
KDE for ZBTB10
Time 1
Time 3
Density
Gene Expression
10
5
0
0.25
0.50
0.75
0.0
0.5
1.0
KDE for XBP1
Time 1
Time 3
Density
Gene Expression
15
10
5
0
0.6
0.8
0.50
0.75
1.00
1.25
KDE for ZFHX3
Time 1
Time 3
Density
Gene Expression
10
5
0
0.0
0.2
0.4
0.2
0.4
0.6
0.8
KDE for DNMT3B
Time 1
Time 3
Density
Gene Expression
6
4
2
0
0.5
1.0
1.5
0.0
0.5
1.0
KDE for RERE
Time 1
Time 3
Density
Gene Expression
15
10
5
0
0.2
0.4
0.2
0.4
0.6
0.8
KDE for MSC
Time 1
Time 3
Density
Gene Expression
10
5
0
0.2
0.4
0.6
−0.2
0.0
0.2
0.4

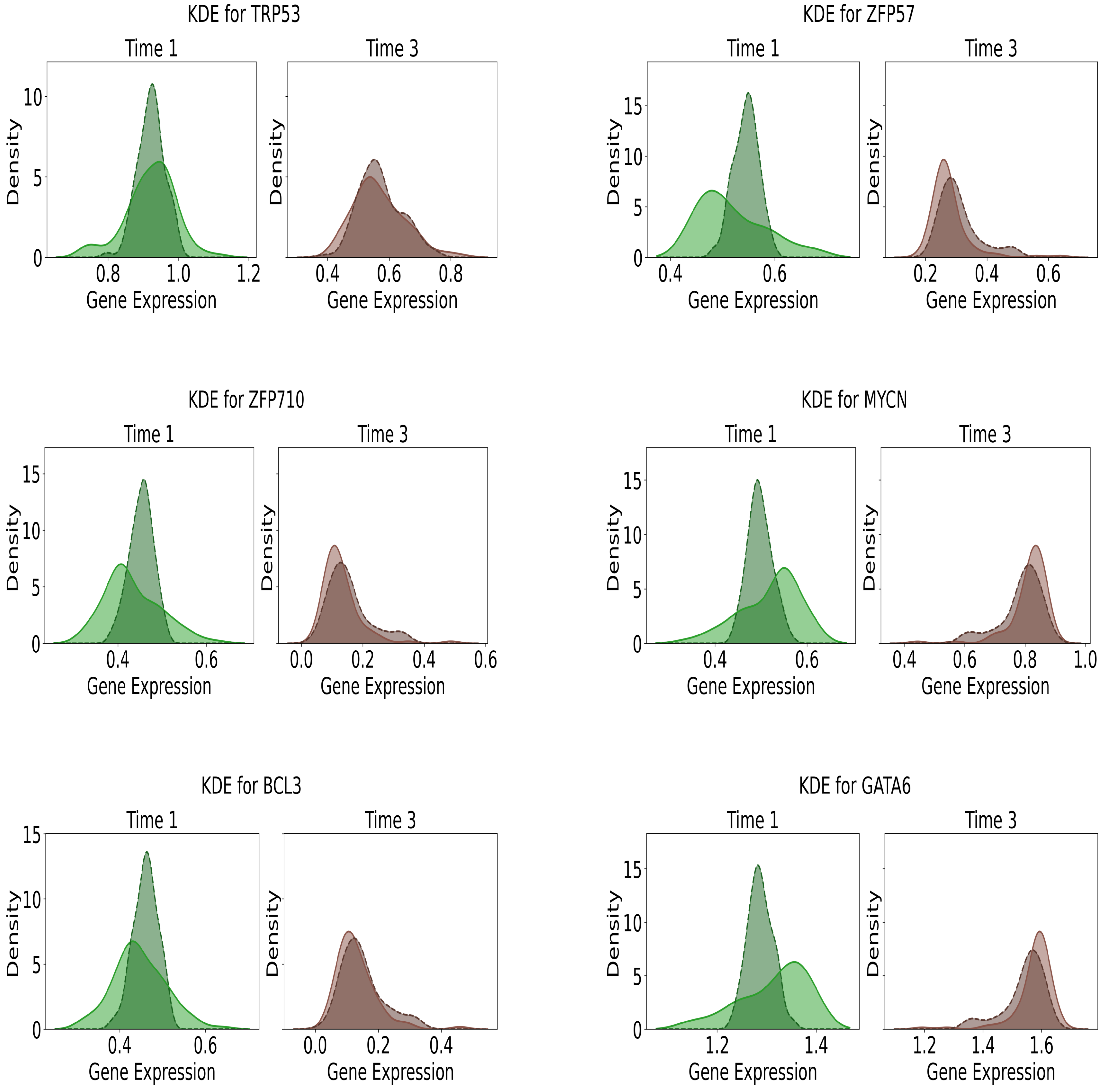
KDE for TRP53
Time 1
Time 3
Density
Gene Expression
KDE for ZFP57
Time 1
Time 3
Density
Gene Expression
KDE for ZFP710
Time 1
Time 3
Density
Gene Expression
KDE for MYCN
Time 1
Time 3
Density
Gene Expression
KDE for BCL3
Time 1
Time 3
Density
Gene Expression
KDE for GATA6
Time 1
Time 3
Density
Gene Expression

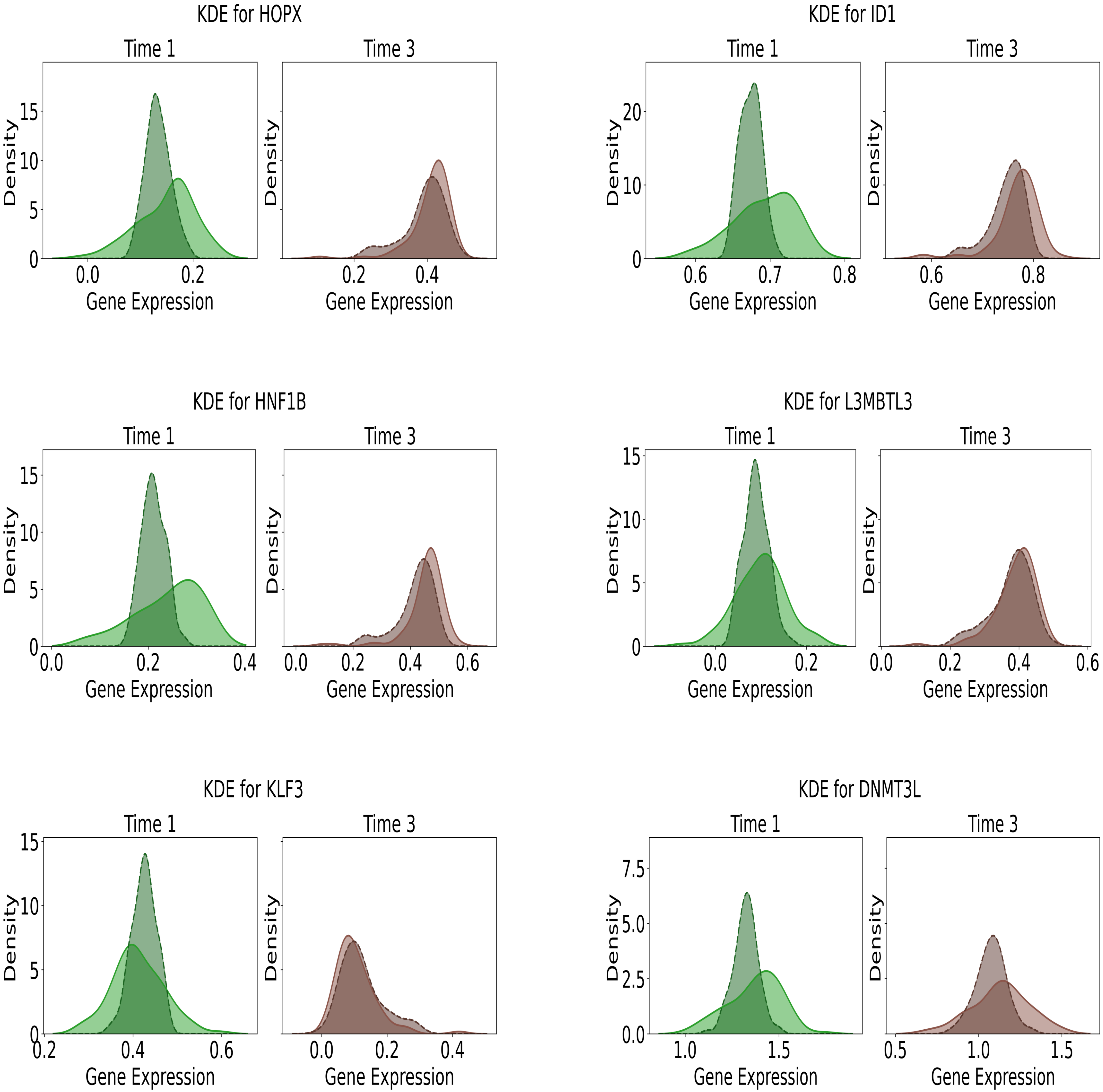

KDE for HOPX
KDE for ID1
KDE for HNF1B
KDE for L3MBTL3
KDE for KLF3
KDE for DNMT3L
Time 1
Time 3
Density
Gene Expression

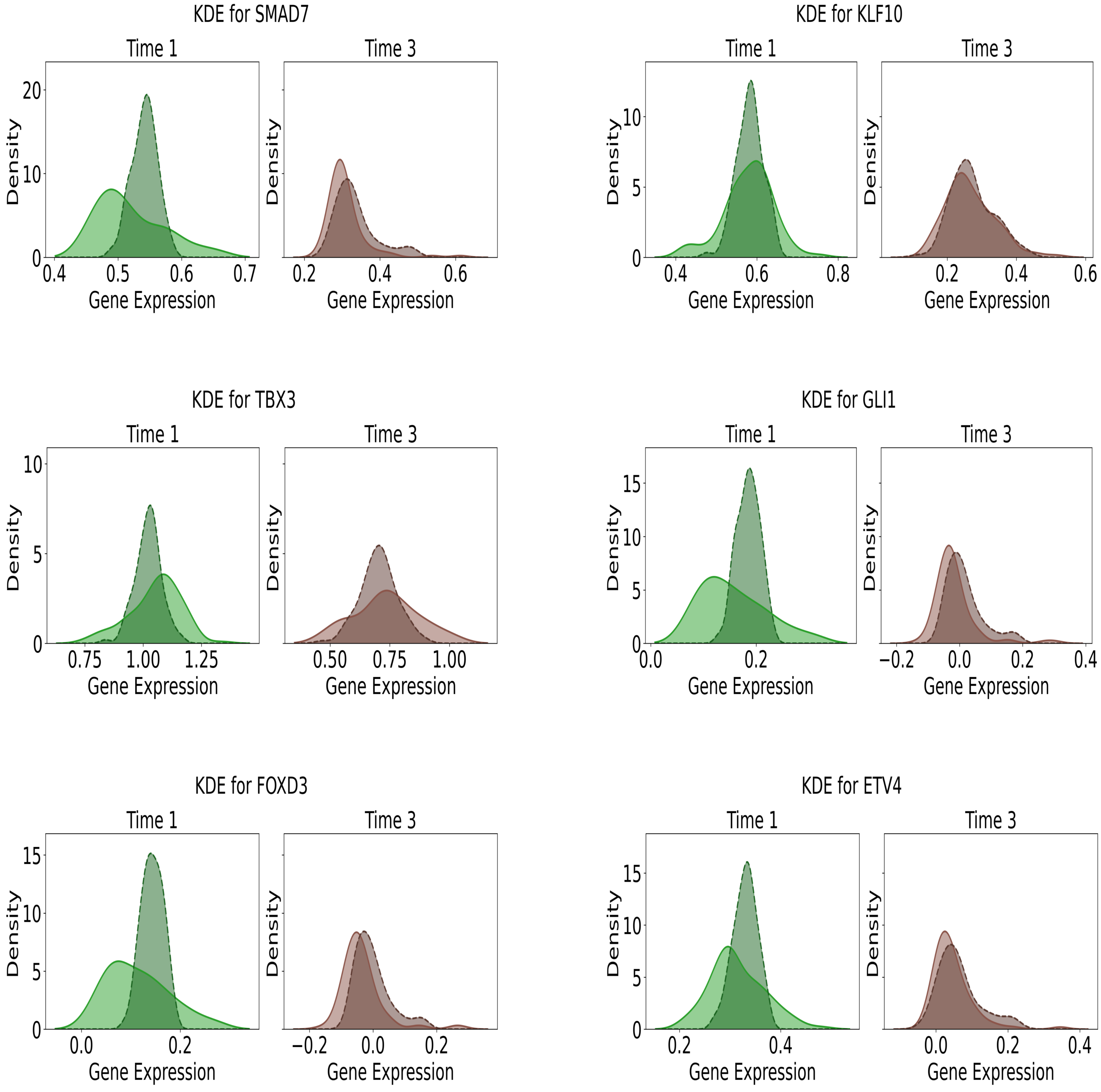

KDE for SMAD7
Time 1
Time 3
KDE for KLF10
Time 1
Time 3
KDE for TBX3
Time 1
Time 3
KDE for GLI1
Time 1
Time 3
KDE for FOXD3
Time 1
Time 3
KDE for ETV4
Time 1
Time 3
Density
Gene Expression

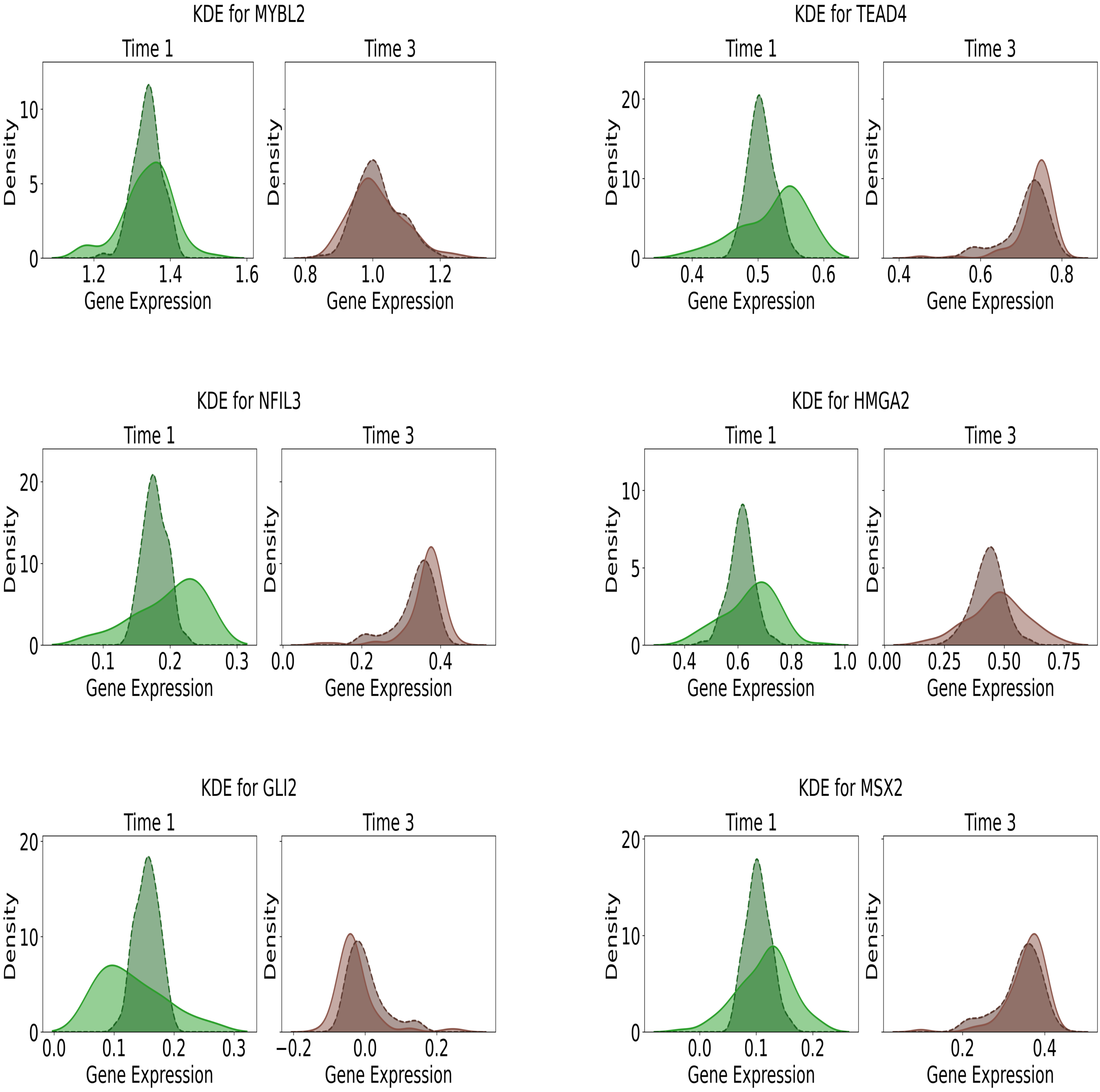
KDE for MYBL2
Time 1
Time 3
Density
Gene Expression
0
5
10
1.2
1.4
1.6
0.8
1.0
1.2
KDE for TEAD4
Time 1
Time 3
Density
Gene Expression
0
10
20
0.4
0.5
0.6
0.4
0.6
0.8
KDE for NFIL3
Time 1
Time 3
Density
Gene Expression
0
10
20
0.1
0.2
0.3
0.0
0.2
0.4
KDE for HMGA2
Time 1
Time 3
Density
Gene Expression
0
5
10
0.4
0.6
0.8
1.0
0.00
0.25
0.50
0.75
KDE for GLI2
Time 1
Time 3
Density
Gene Expression
0
10
20
0.0
0.1
0.2
0.3
−0.2
0.0
0.2
KDE for MSX2
Time 1
Time 3
Density
Gene Expression
0
10
20
0.0
0.1
0.2
0.2
0.4

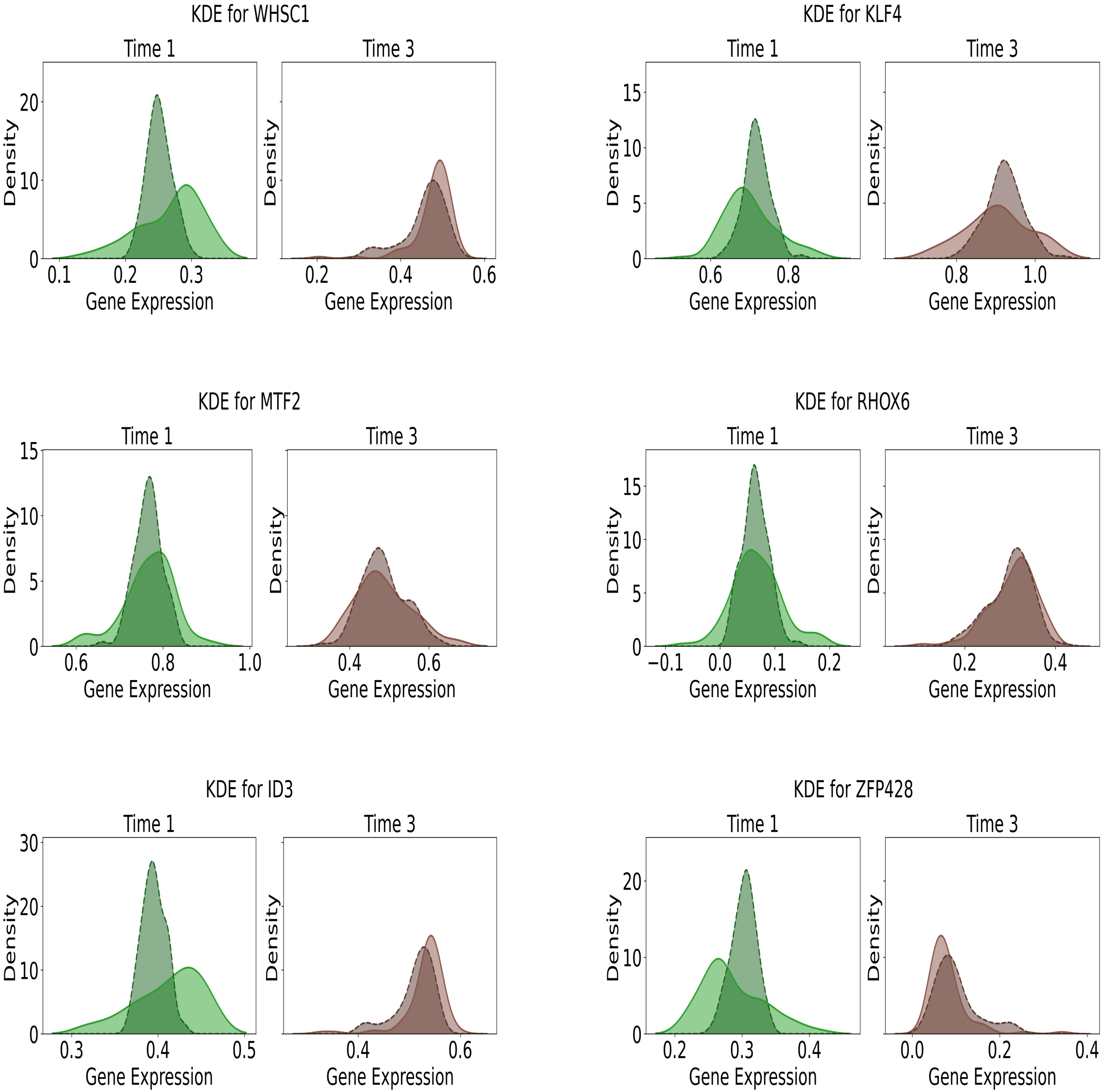

KDE for WHSC1
Time 1
Time 3
Density
Gene Expression
0
10
20
0.1
0.2
0.3
0.4
0.6
KDE for KLF4
Time 1
Time 3
Density
Gene Expression
0
5
10
15
0.6
0.8
1.0
KDE for MTF2
Time 1
Time 3
Density
Gene Expression
0
5
10
15
0.6
0.8
1.0
0.4
0.6
KDE for RHOX6
Time 1
Time 3
Density
Gene Expression
0
5
10
15
−0.1
0.0
0.1
0.2
0.4
KDE for ID3
Time 1
Time 3
Density
Gene Expression
0
10
20
30
0.3
0.4
0.5
0.6
KDE for ZFP428
Time 1
Time 3
Density
Gene Expression
0
10
20
0.2
0.3
0.4
0.0

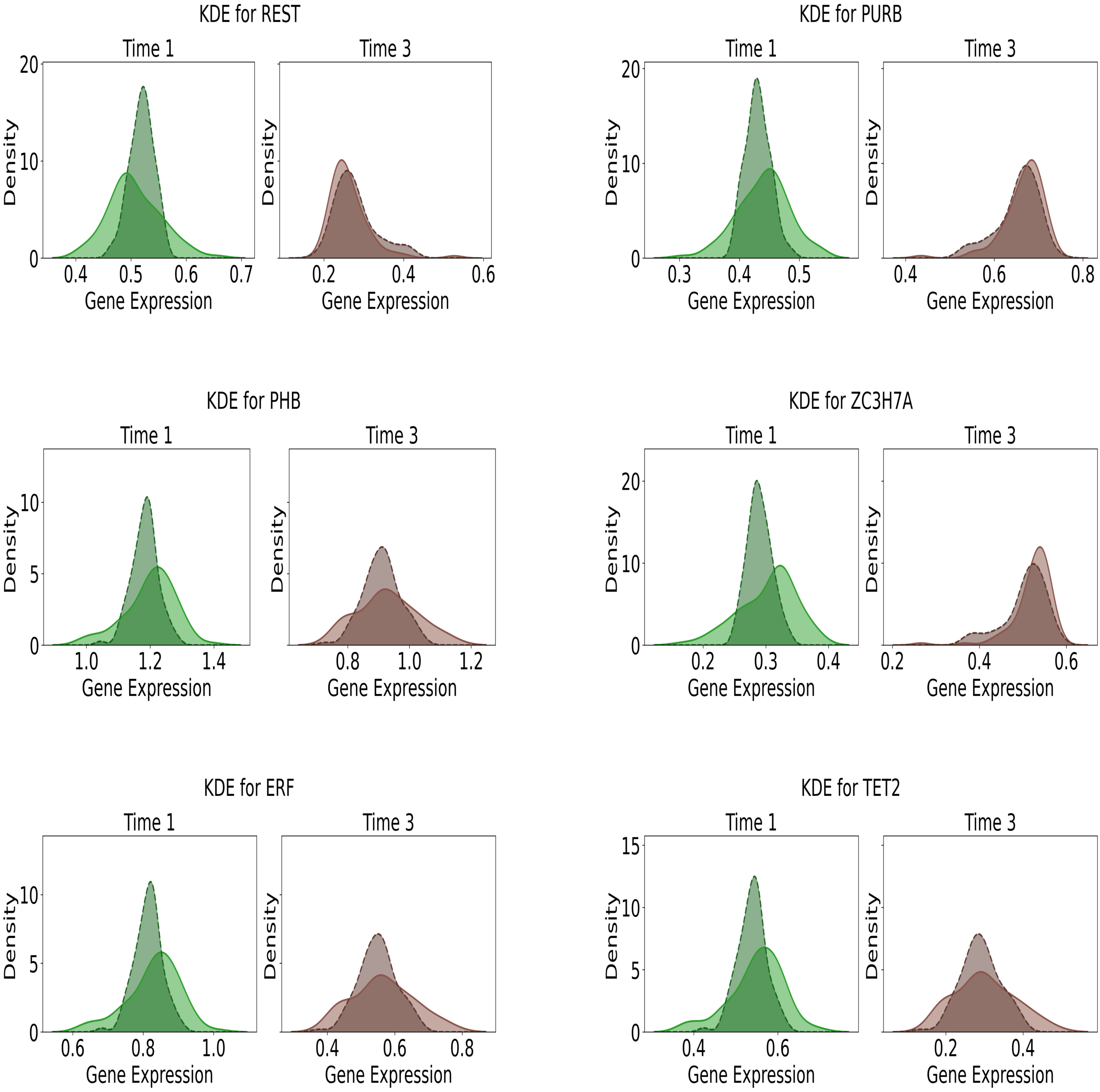

KDE for REST
Time 1
Time 3
Density
Gene Expression
0
10
20
0.4
0.5
0.6
0.7
0.2
0.4
0.6
KDE for PURB
Time 1
Time 3
Density
Gene Expression
0
10
20
0.3
0.4
0.5
0.4
0.6
0.8
KDE for PHB
Time 1
Time 3
Density
Gene Expression
0
5
10
1.0
1.2
1.4
0.8
1.0
1.2
KDE for ZC3H7A
Time 1
Time 3
Density
Gene Expression
0
10
20
0.2
0.3
0.4
0.2
0.4
0.6
KDE for ERF
Time 1
Time 3
Density
Gene Expression
0
5
10
0.6
0.8
1.0
0.4
0.6
0.8
KDE for TET2
Time 1
Time 3
Density
Gene Expression
0
5
10
15
0.4
0.6
0.2
0.4

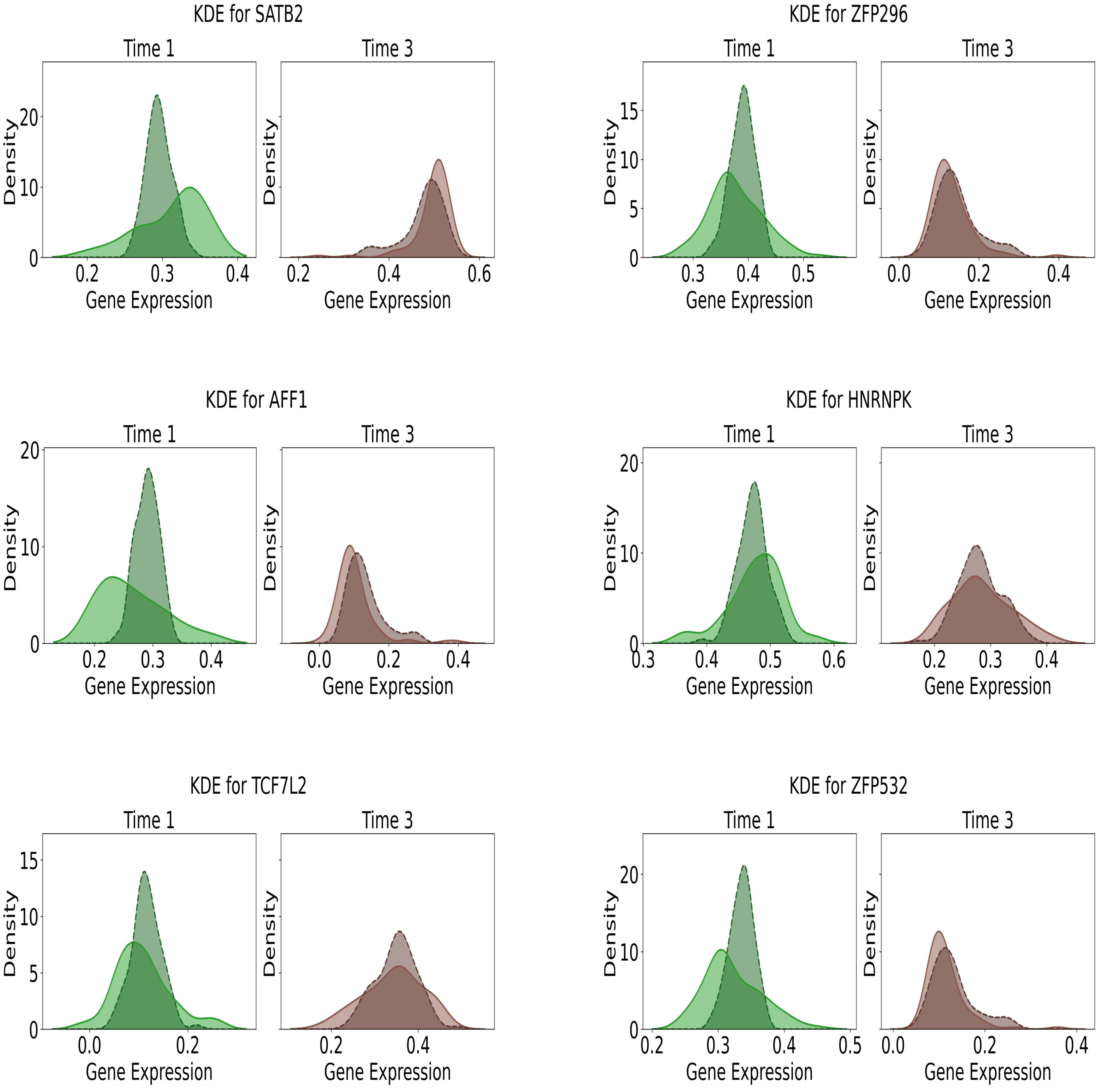

KDE for SATB2
Time 1
Time 3
Density
Gene Expression
KDE for ZFP296
Time 1
Time 3
Density
Gene Expression
KDE for AFF1
Time 1
Time 3
Density
Gene Expression
KDE for HNRNPK
Time 1
Time 3
Density
Gene Expression
KDE for TCF7L2
Time 1
Time 3
Density
Gene Expression
KDE for ZFP532
Time 1
Time 3
Density
Gene Expression

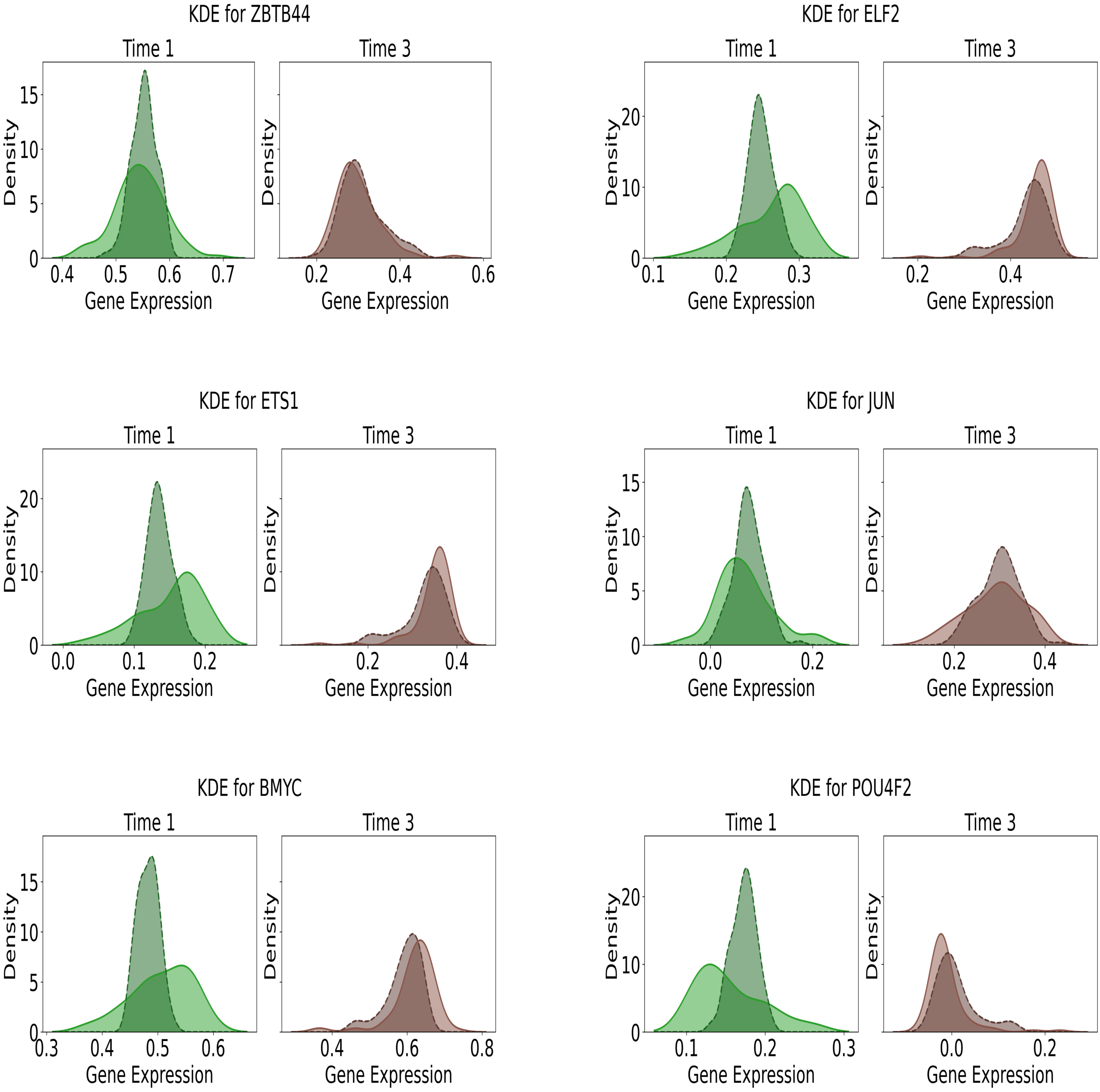

KDE for ZBTB44
Time 1
Time 3
Density
Gene Expression
0
5
10
15
0.4
0.5
0.6
0.7
0.2
0.4
0.6
KDE for ELF2
Time 1
Time 3
Density
Gene Expression
0
10
20
0.1
0.2
0.3
0.2
0.4
KDE for ETS1
Time 1
Time 3
Density
Gene Expression
0
10
20
0.0
0.1
0.2
0.2
0.4
KDE for JUN
Time 1
Time 3
Density
Gene Expression
0
5
10
15
0.0
0.2
0.2
0.4
KDE for BMYC
Time 1
Time 3
Density
Gene Expression
0
5
10
15
0.3
0.4
0.5
0.6
0.4
0.6
0.8
KDE for POU4F2
Time 1
Time 3
Density
Gene Expression
0
10
20
0.1
0.2
0.3
0.0
0.2

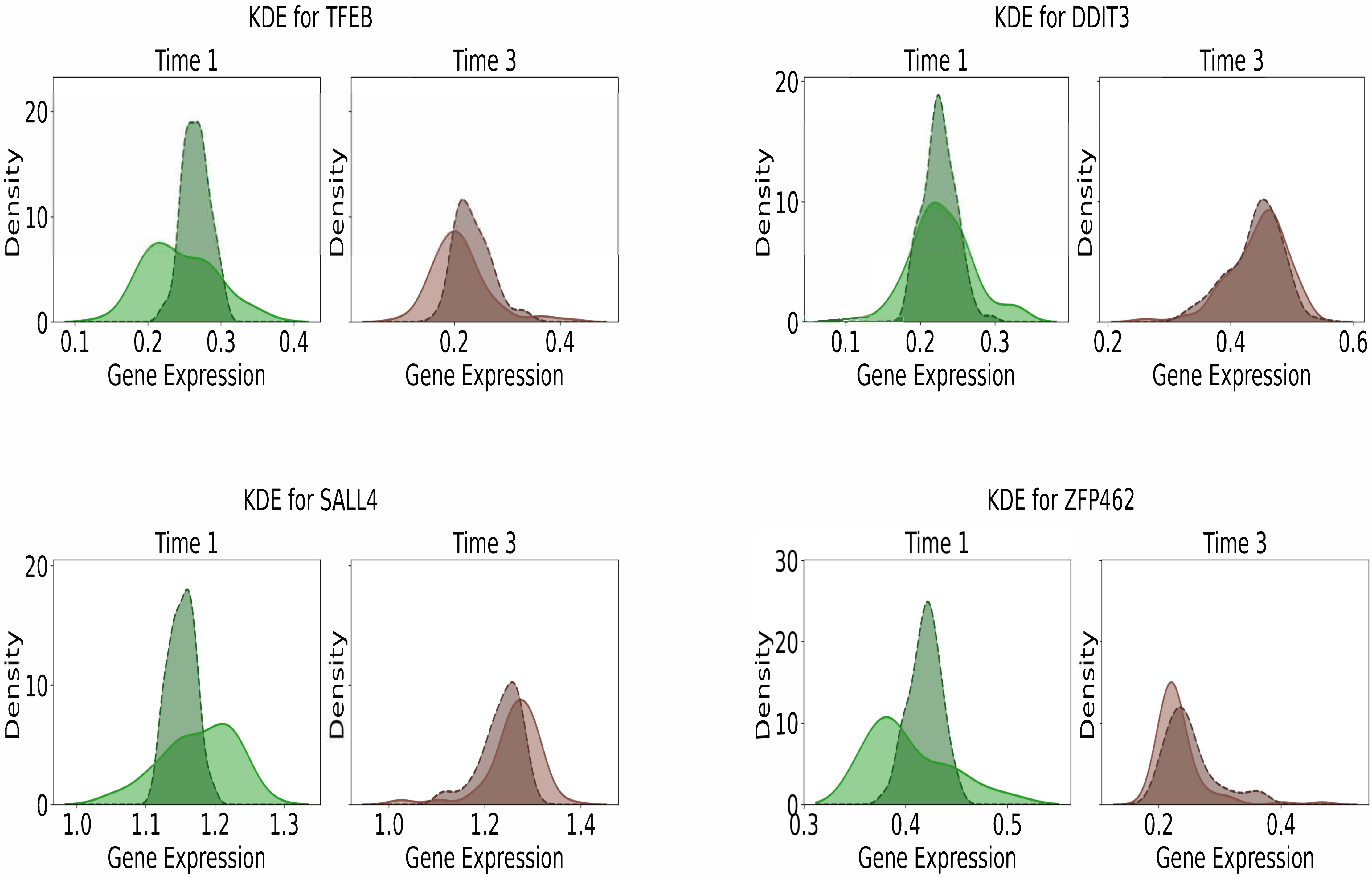

Supplementary Figure 4:Comparison of predicted and actual gene expression distributions at day 1 (green) and day 3 (brown). Predicted distributions are shown as dashed lines, with real data distributions shown as solid lines
