# Supplementary material for: PROFET Predicts Continuous Gene Expression Dynamics from scRNA-seq Data to Elucidate Heterogeneity of Cancer Treatment Responses
Source: bioRxiv. 2025 Jul 3:2025.06.27.662030. Preprint. [Version 1] doi: 10.1101/2025.06.27.662030 (PMC12236938; doi:10.1101/2025.06.27.662030)
Supplement: Supplement 5 [file media-6.pdf]

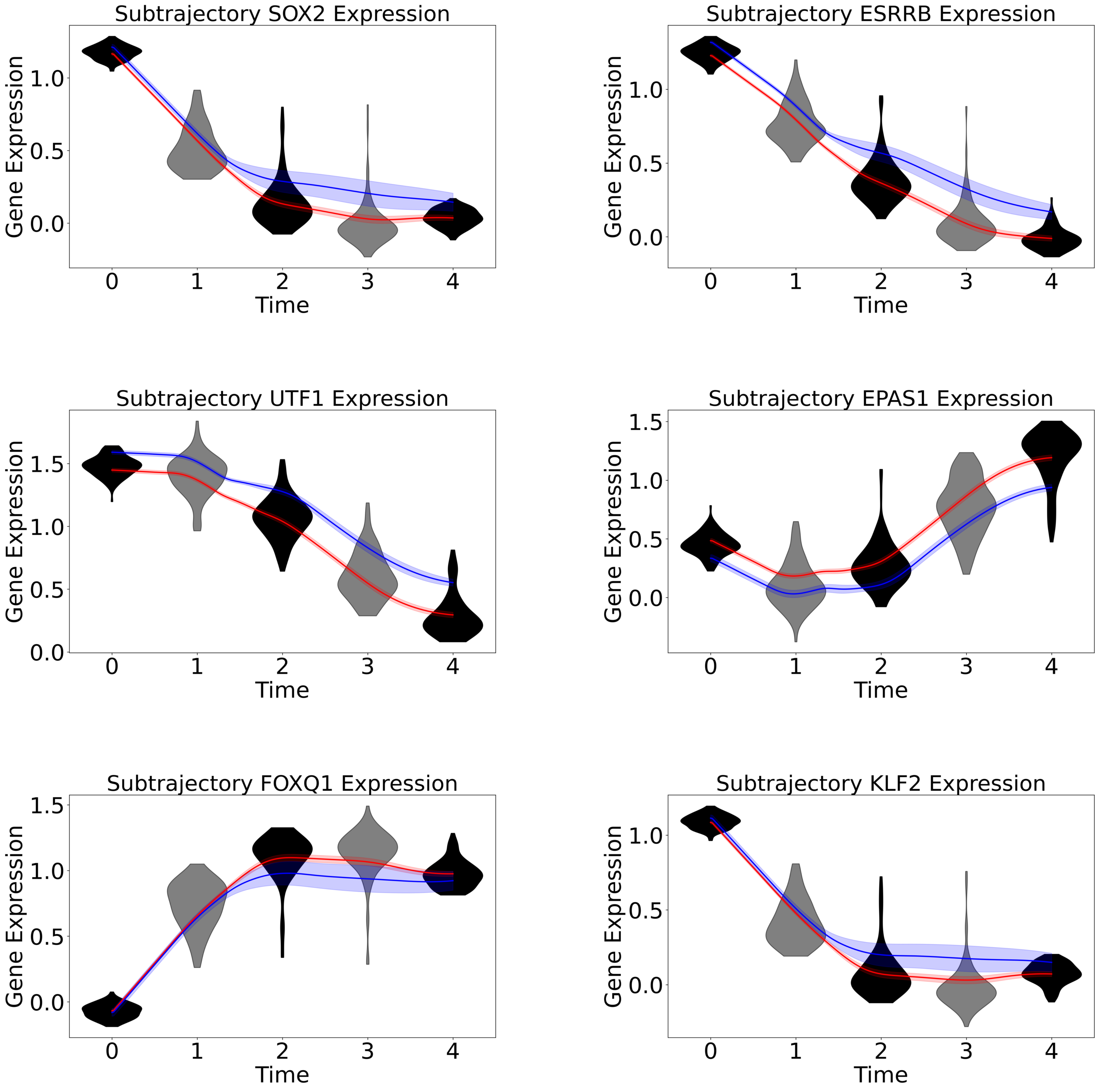

Subtrajectory SOX2 Expression
Subtrajectory ESRRB Expression
Subtrajectory UTF1 Expression
Subtrajectory EPAS1 Expression
Subtrajectory FOXQ1 Expression
Subtrajectory KLF2 Expression
Gene Expression
Time

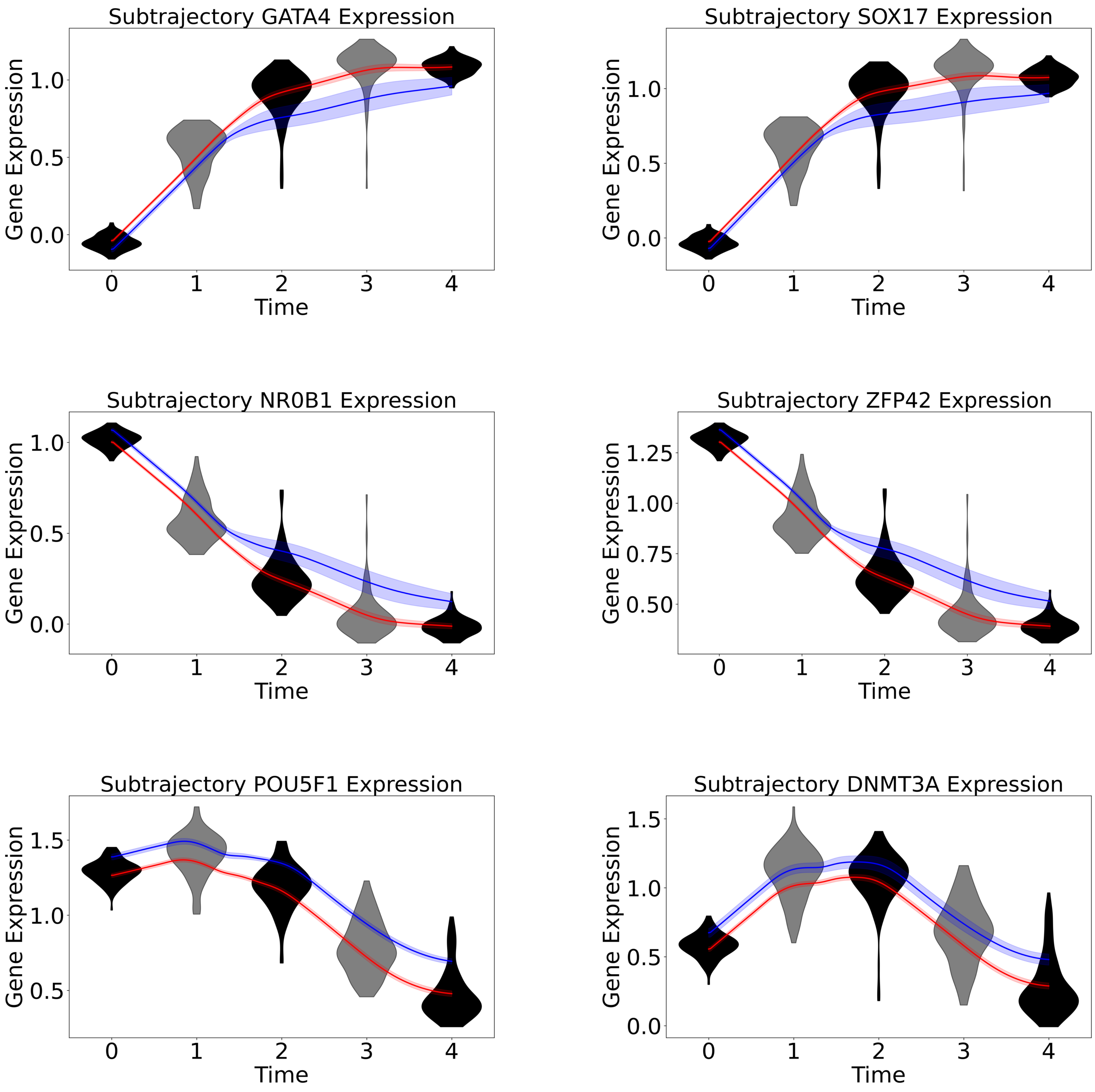
Subtrajectory GATA4 Expression
Subtrajectory SOX17 Expression
Subtrajectory NR0B1 Expression
Subtrajectory ZFP42 Expression
Subtrajectory POU5F1 Expression
Subtrajectory DNMT3A Expression
Gene Expression
Time
0
1
2
3
4
0.0
0.5
1.0
1.5
0.50
0.75
1.00
1.25

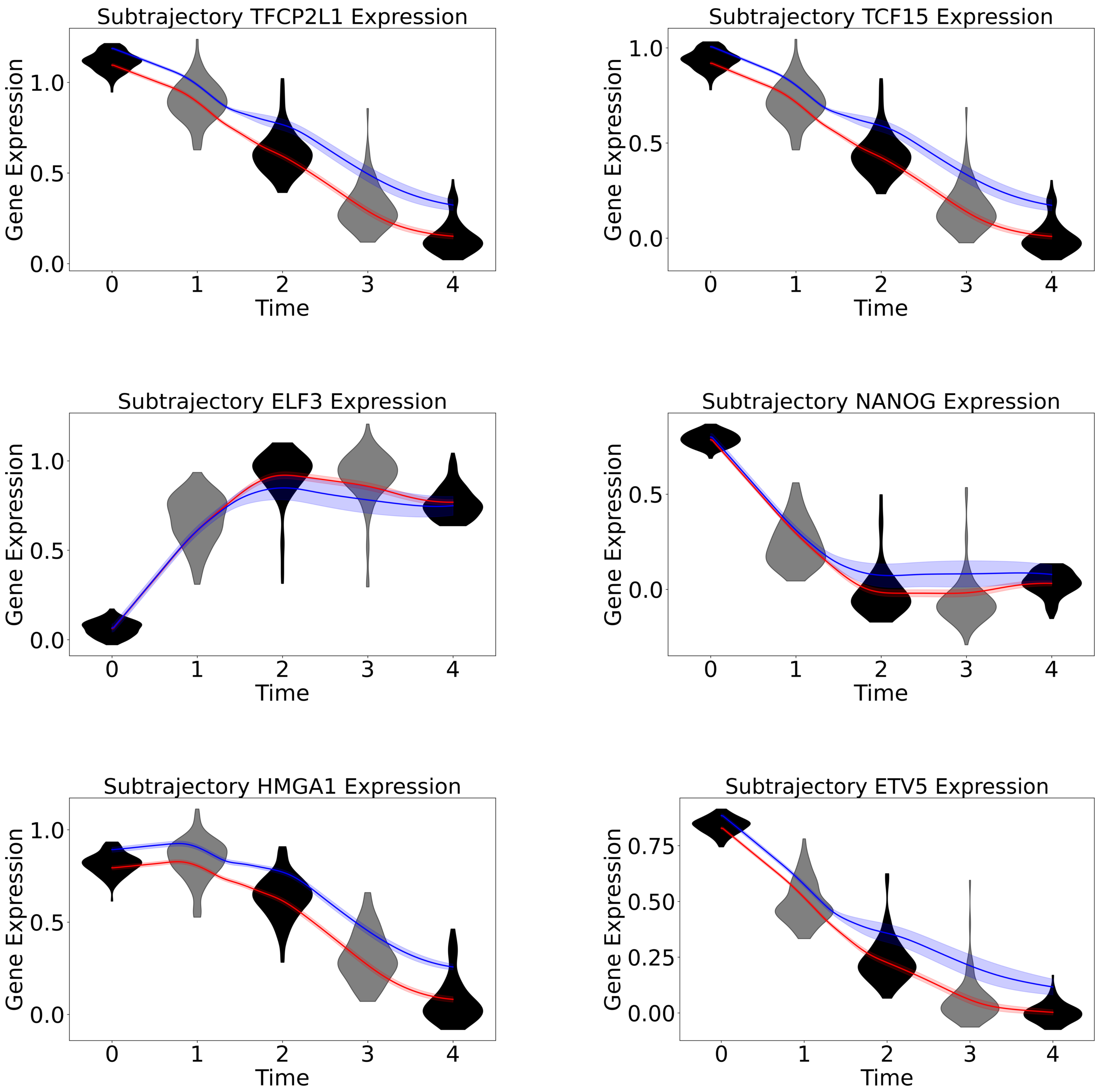
Subtrajectory TFCP2L1 Expression
Subtrajectory TCF15 Expression
Subtrajectory ELF3 Expression
Subtrajectory NANOG Expression
Subtrajectory HMGA1 Expression
Subtrajectory ETV5 Expression
Gene Expression
Time
0
1
2
3
4
0.0
0.5
1.0
0.00
0.25
0.50
0.75

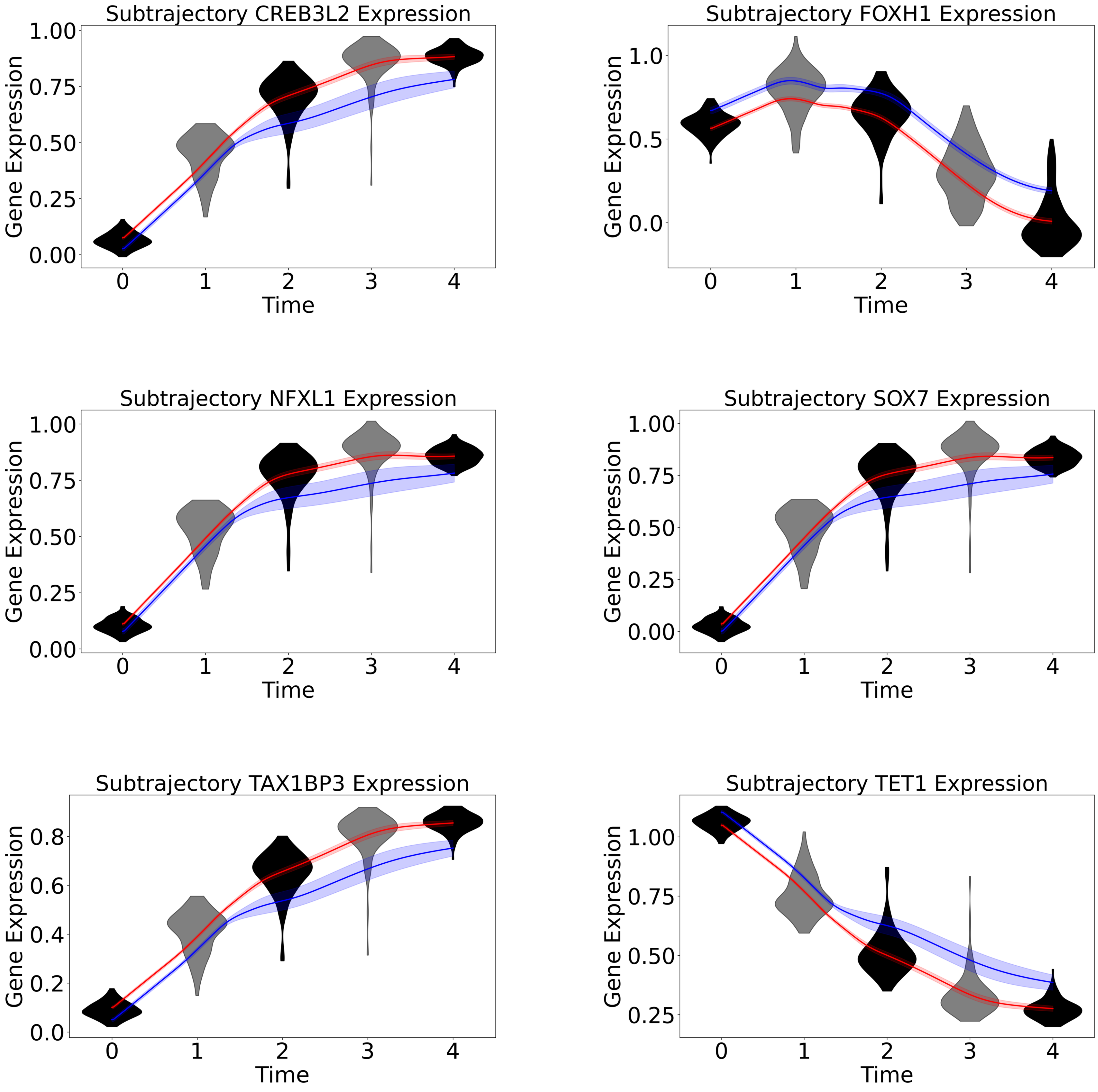

Subtrajectory CREB3L2 Expression
Subtrajectory FOXH1 Expression
Subtrajectory NFXL1 Expression
Subtrajectory SOX7 Expression
Subtrajectory TAX1BP3 Expression
Subtrajectory TET1 Expression
Gene Expression
Time
0.00
0.25
0.50
0.75
1.00
0.0
0.5
1.0
0.2
0.4
0.6
0.8
0
1
2
3
4

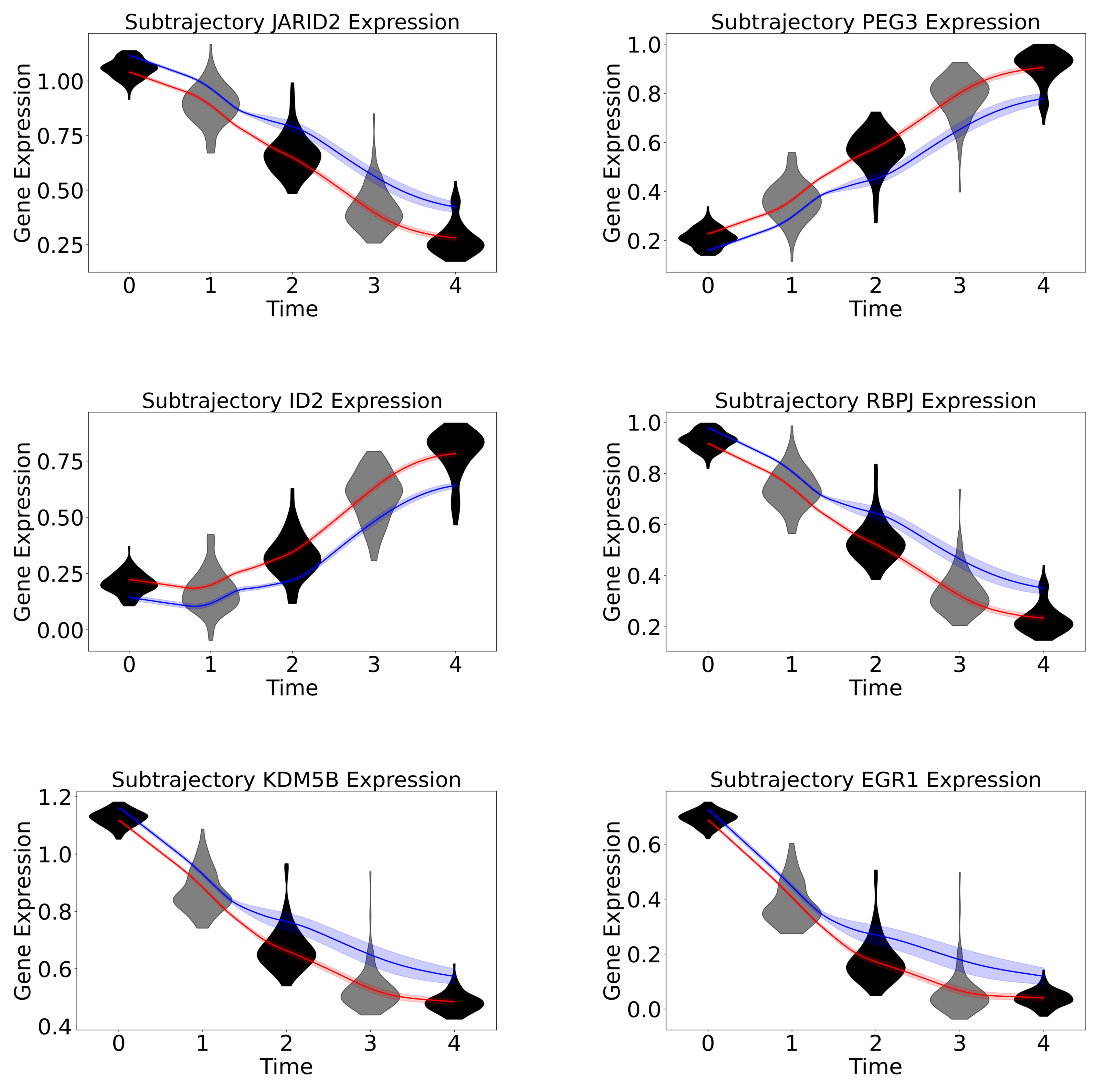
Subtrajectory JARID2 Expression
Gene Expression
1.00
0.75
0.50
0.25
0
1
2
3
4
Time
Subtrajectory PEG3 Expression
Gene Expression
1.0
0.8
0.6
0.4
0.2
0
1
2
3
4
Time
Subtrajectory ID2 Expression
Gene Expression
0.75
0.50
0.25
0.00
0
1
2
3
4
Time
Subtrajectory RBPJ Expression
Gene Expression
1.0
0.8
0.6
0.4
0.2
0
1
2
3
4
Time
Subtrajectory KDM5B Expression
Gene Expression
1.2
1.0
0.8
0.6
0.4
0
1
2
3
4
Time
Subtrajectory EGR1 Expression
Gene Expression
0.6
0.4
0.2
0.0
0
1
2
3
4
Time

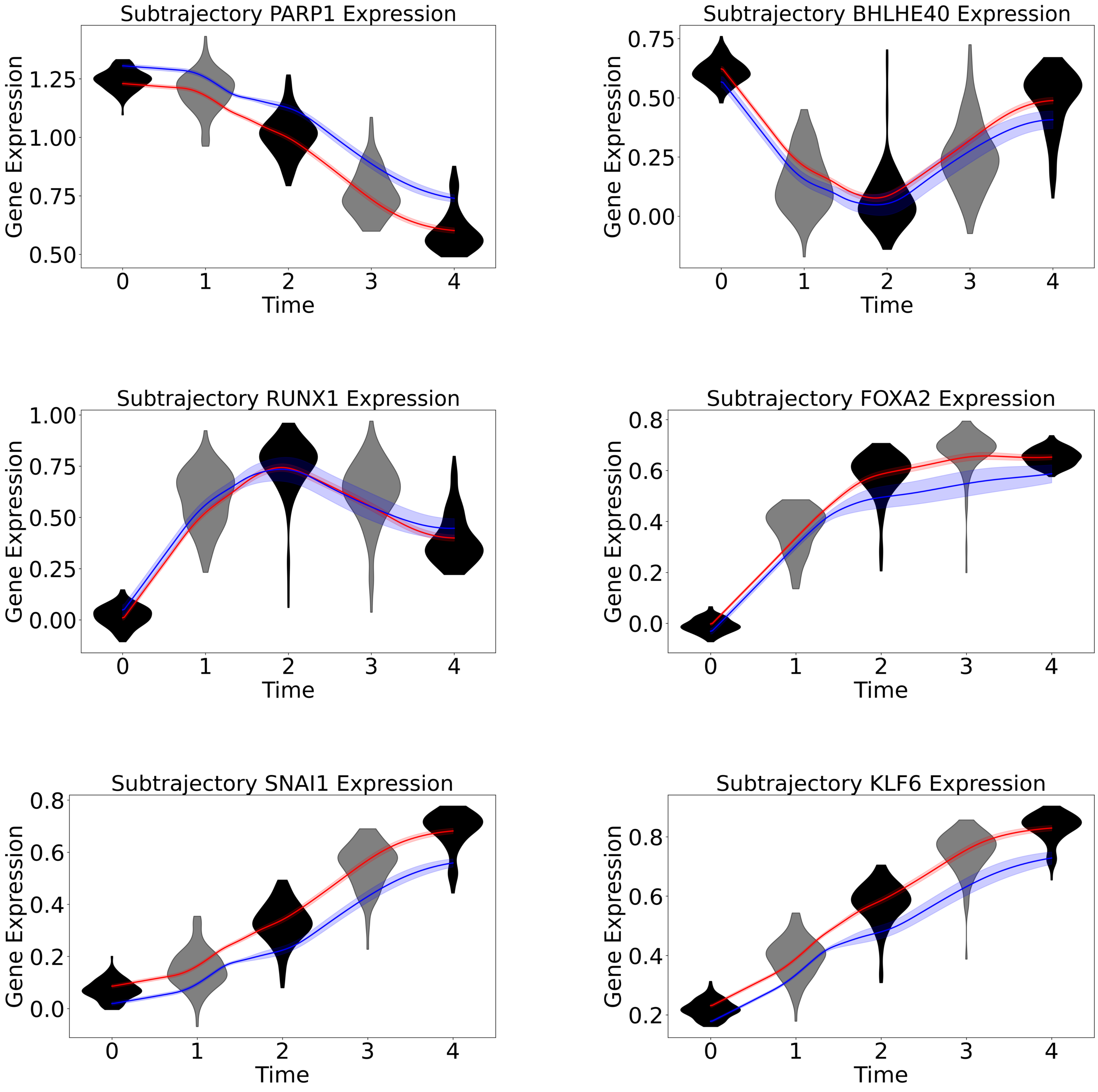
Subtrajectory PARP1 Expression
Gene Expression
1.25
1.00
0.75
0.50
0
1
2
3
4
Time
Subtrajectory BHLHE40 Expression
Gene Expression
0.75
0.50
0.25
0.00
0
1
2
3
4
Time
Subtrajectory RUNX1 Expression
Gene Expression
1.00
0.75
0.50
0.25
0.00
0
1
2
3
4
Time
Subtrajectory FOXA2 Expression
Gene Expression
0.8
0.6
0.4
0.2
0.0
0
1
2
3
4
Time
Subtrajectory SNAI1 Expression
Gene Expression
0.8
0.6
0.4
0.2
0.0
0
1
2
3
4
Time
Subtrajectory KLF6 Expression
Gene Expression
0.8
0.6
0.4
0.2
0
1
2
3
4
Time

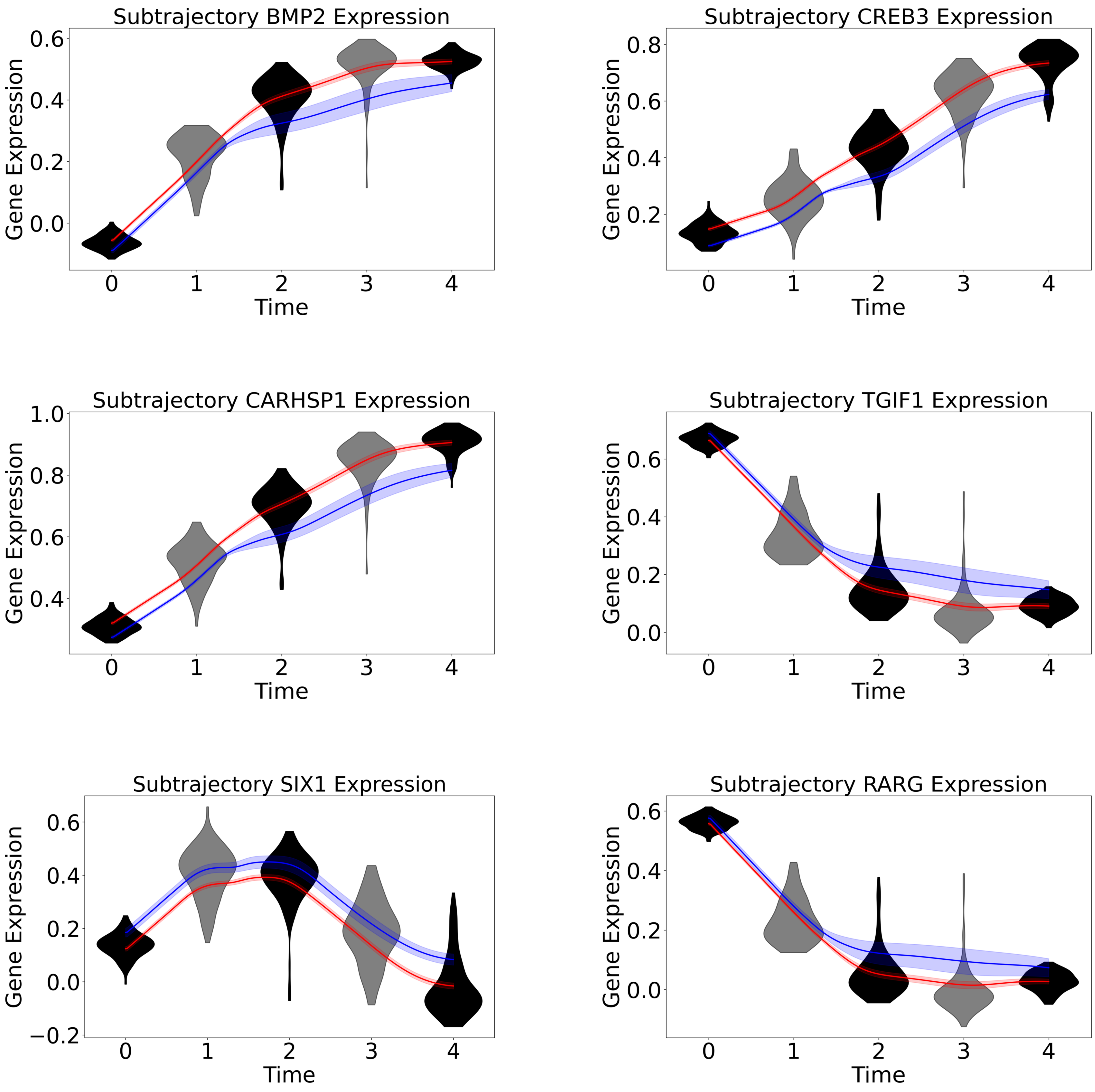
Subtrajectory BMP2 Expression
Subtrajectory CREB3 Expression
Subtrajectory CARHSP1 Expression
Subtrajectory TGIF1 Expression
Subtrajectory SIX1 Expression
Subtrajectory RARG Expression
Gene Expression
Time

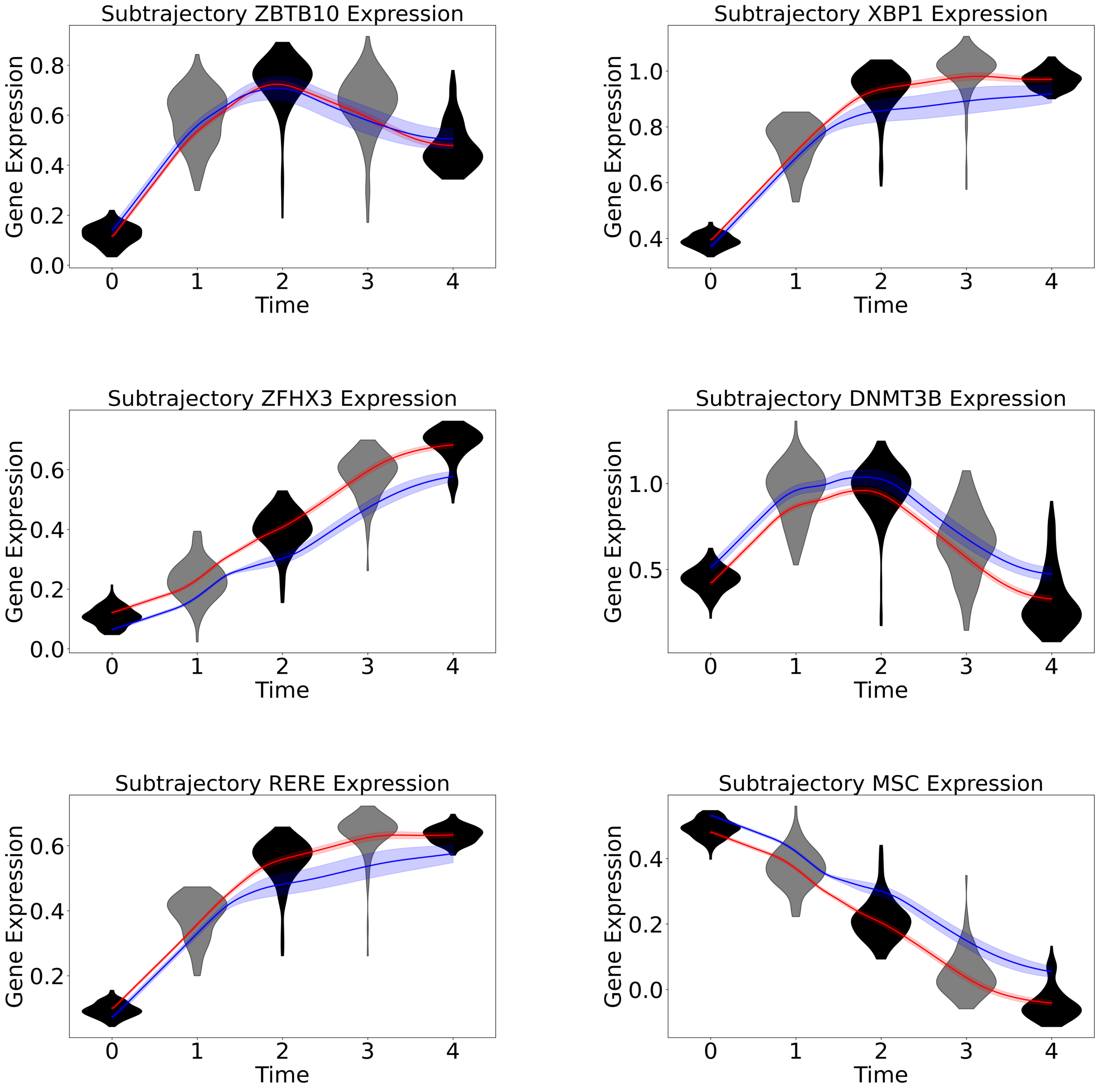

Subtrajectory ZBTB10 Expression
Subtrajectory XBP1 Expression
Subtrajectory ZFHX3 Expression
Subtrajectory DNMT3B Expression
Subtrajectory RERE Expression
Subtrajectory MSC Expression
Gene Expression
Time
0
1
2
3
4
0.0
0.2
0.4
0.6
0.8
1.0
0.5

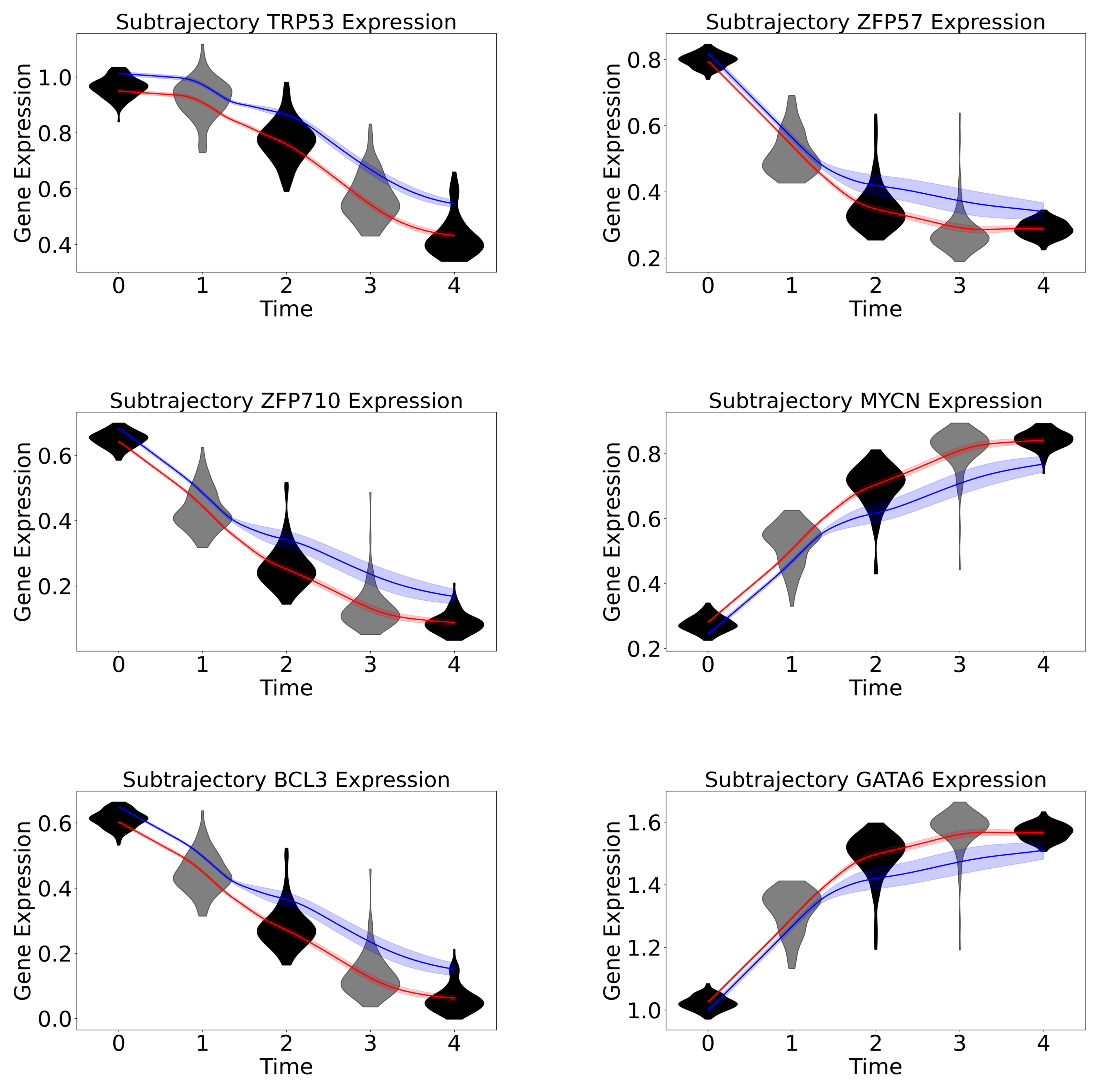
Subtrajectory TRP53 Expression
Subtrajectory ZFP57 Expression
Subtrajectory ZFP710 Expression
Subtrajectory MYCN Expression
Subtrajectory BCL3 Expression
Subtrajectory GATA6 Expression
Gene Expression
Time

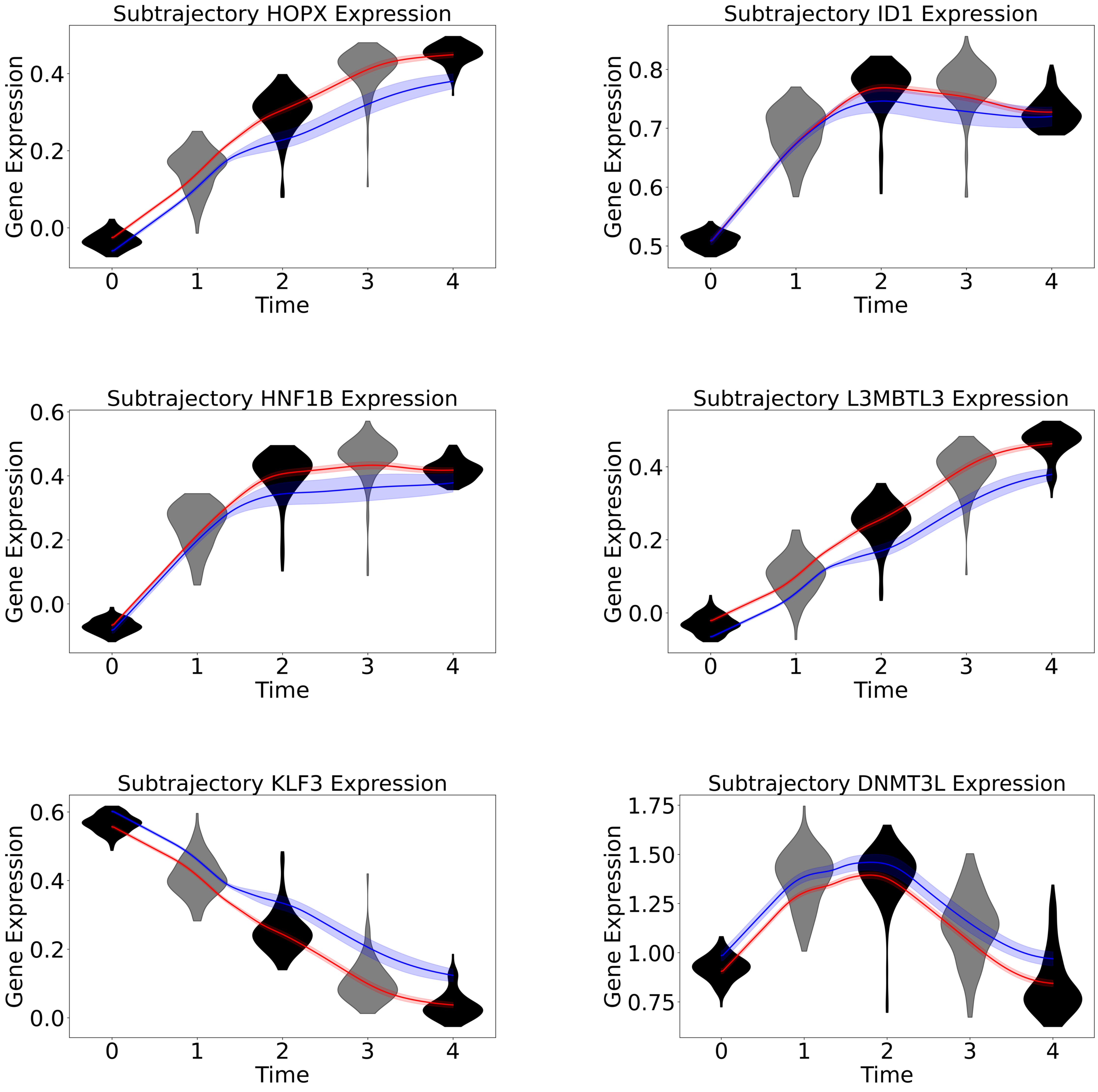

Subtrajectory HOPX Expression
Gene Expression
Time
Subtrajectory ID1 Expression
Gene Expression
Time
Subtrajectory HNF1B Expression
Gene Expression
Time
Subtrajectory L3MBTL3 Expression
Gene Expression
Time
Subtrajectory KLF3 Expression
Gene Expression
Time
Subtrajectory DNMT3L Expression
Gene Expression
Time

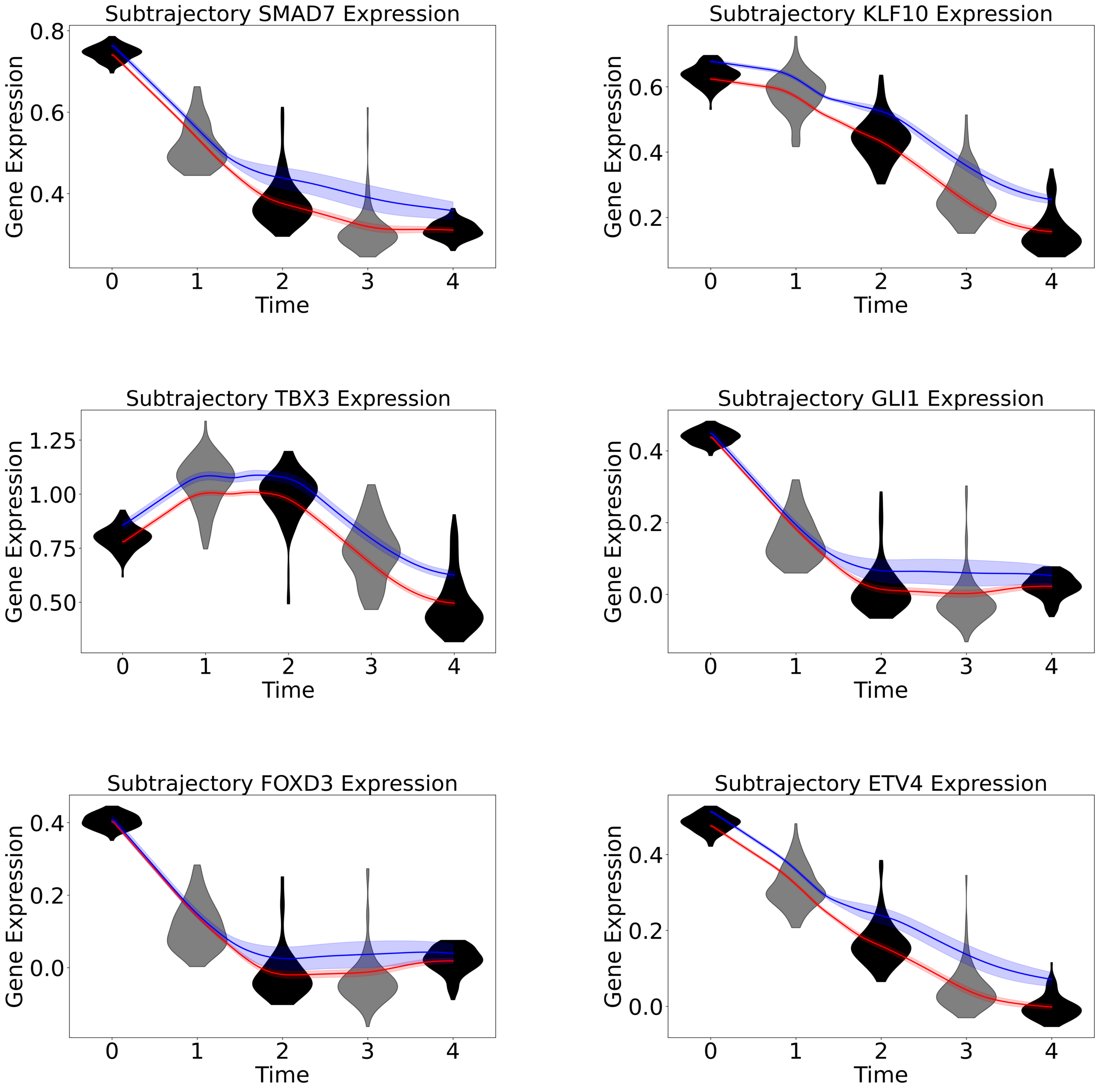

Subtrajectory SMAD7 Expression
Gene Expression
0.8
0.6
0.4
0
1
2
3
4
Time
Subtrajectory KLF10 Expression
Gene Expression
0.6
0.4
0.2
0
1
2
3
4
Time
Subtrajectory TBX3 Expression
Gene Expression
1.25
1.00
0.75
0.50
0
1
2
3
4
Time
Subtrajectory GLI1 Expression
Gene Expression
0.4
0.2
0.0
0
1
2
3
4
Time
Subtrajectory FOXD3 Expression
Gene Expression
0.4
0.2
0.0
0
1
2
3
4
Time
Subtrajectory ETV4 Expression
Gene Expression
0.4
0.2
0.0
0
1
2
3
4
Time

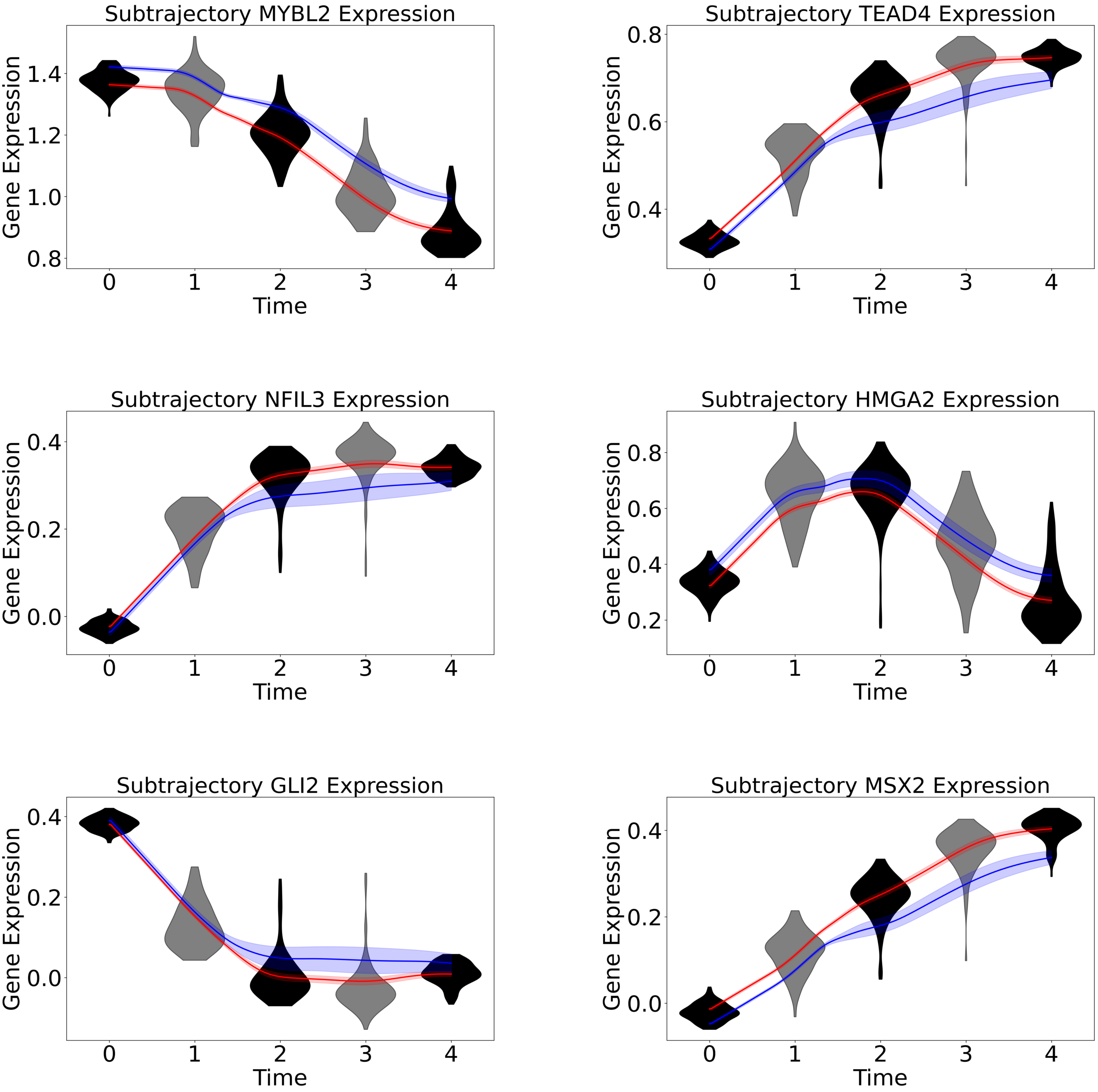
Subtrajectory MYBL2 Expression
Gene Expression
1.4
1.2
1.0
0.8
0
1
2
3
4
Time
Subtrajectory TEAD4 Expression
Gene Expression
0.8
0.6
0.4
0
1
2
3
4
Time
Subtrajectory NFIL3 Expression
Gene Expression
0.4
0.2
0.0
0
1
2
3
4
Time
Subtrajectory HMGA2 Expression
Gene Expression
0.8
0.6
0.4
0.2
0
1
2
3
4
Time
Subtrajectory GLI2 Expression
Gene Expression
0.4
0.2
0.0
0
1
2
3
4
Time
Subtrajectory MSX2 Expression
Gene Expression
0.4
0.2
0.0
0
1
2
3
4
Time

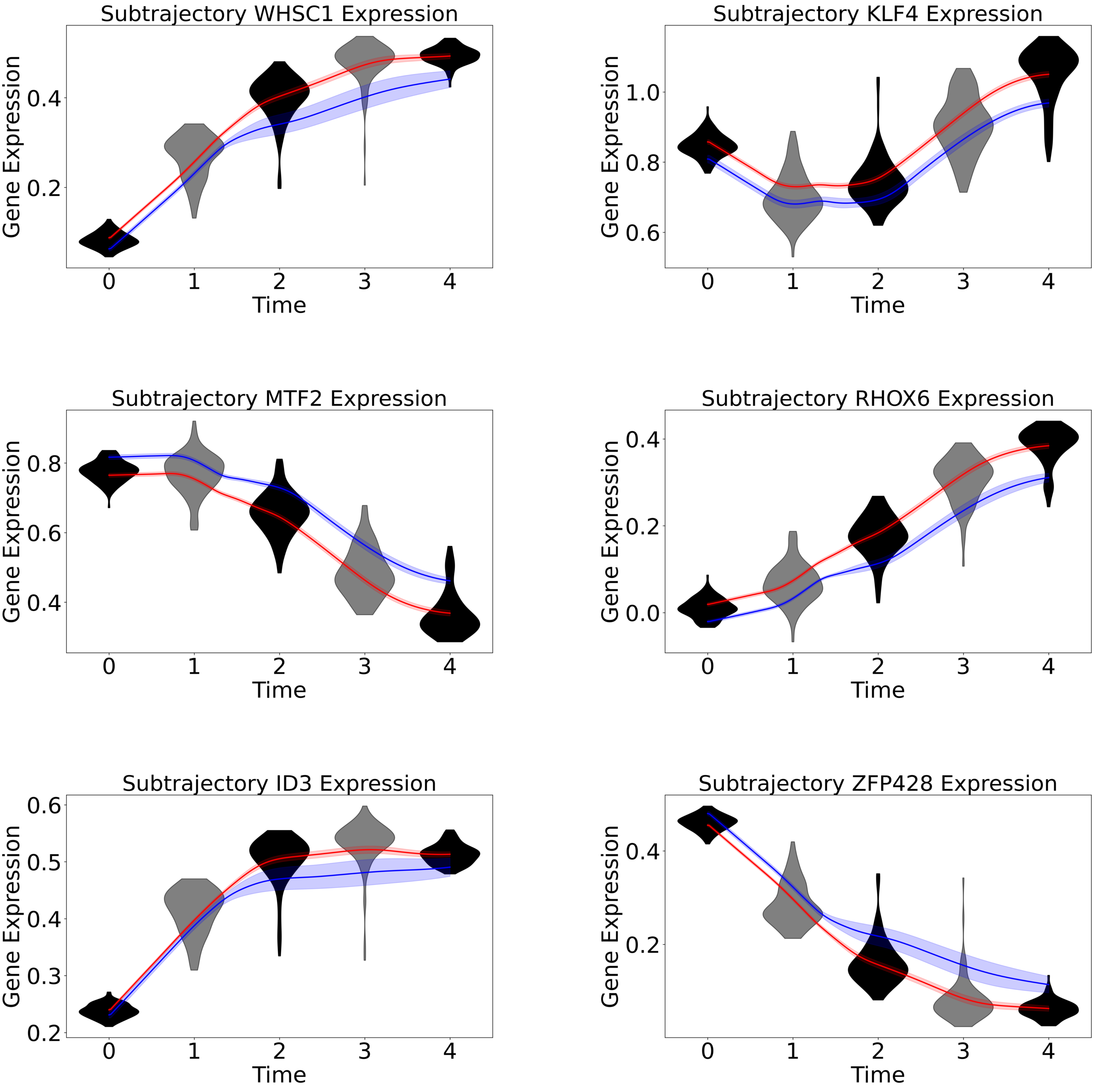
Subtrajectory WHSC1 Expression
Subtrajectory KLF4 Expression
Subtrajectory MTF2 Expression
Subtrajectory RHOX6 Expression
Subtrajectory ID3 Expression
Subtrajectory ZFP428 Expression
Gene Expression
Time
0
1
2
3
4

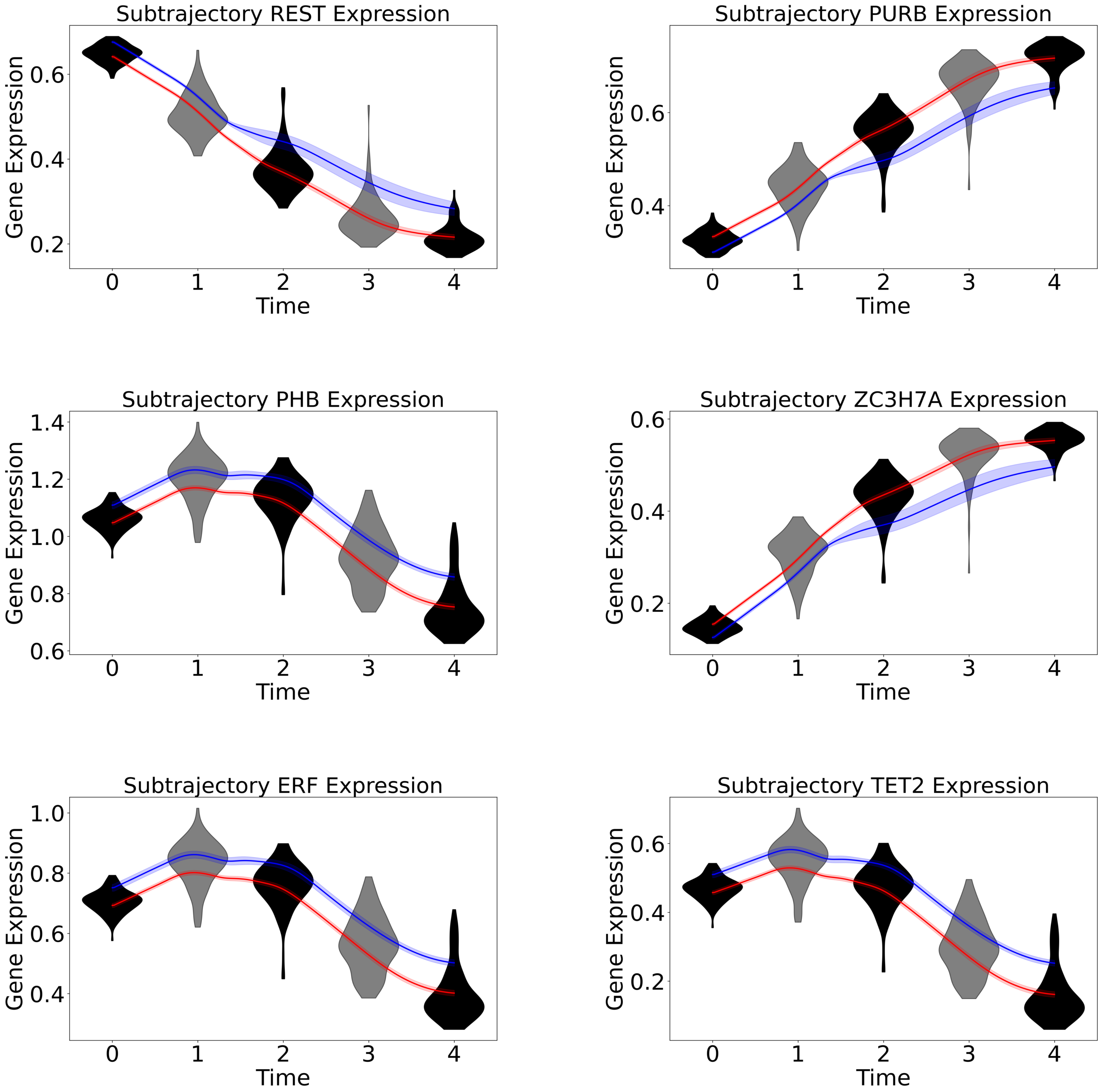
Subtrajectory REST Expression
Subtrajectory PURB Expression
Subtrajectory PHB Expression
Subtrajectory ZC3H7A Expression
Subtrajectory ERF Expression
Subtrajectory TET2 Expression
Gene Expression
Time
0
1
2
3
4
0.2
0.4
0.6
0.8
1.0
1.2
1.4

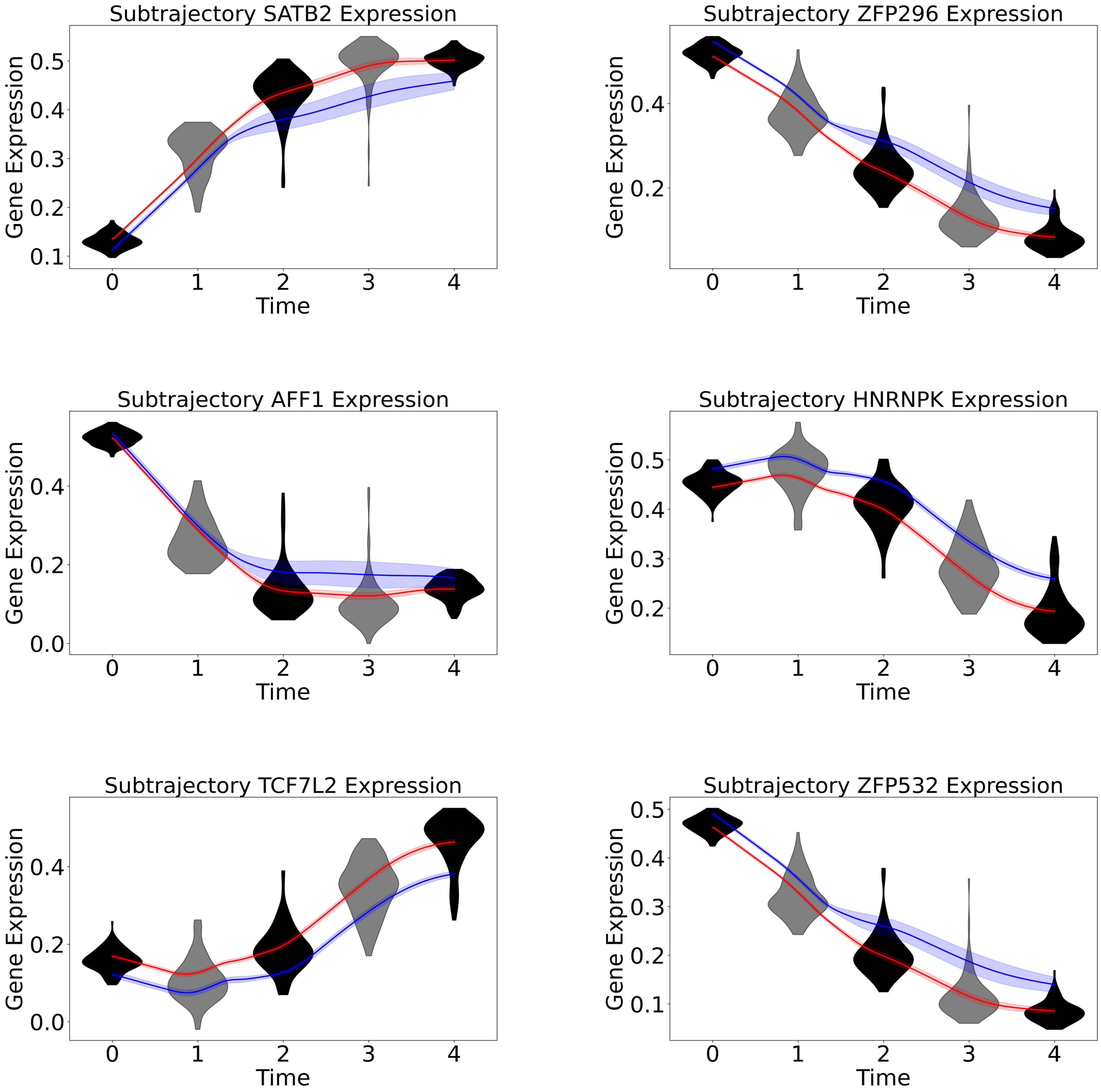
Subtrajectory SATB2 Expression
Gene Expression
0.1
0.2
0.3
0.4
0.5
Time
0
1
2
3
4
Subtrajectory ZFP296 Expression
Gene Expression
0.2
0.4
Time
0
1
2
3
4
Subtrajectory AFF1 Expression
Gene Expression
0.0
0.2
0.4
Time
0
1
2
3
4
Subtrajectory HNRNPK Expression
Gene Expression
0.2
0.3
0.4
0.5
Time
0
1
2
3
4
Subtrajectory TCF7L2 Expression
Gene Expression
0.0
0.2
0.4
Time
0
1
2
3
4
Subtrajectory ZFP532 Expression
Gene Expression
0.1
0.2
0.3
0.4
0.5
Time
0
1
2
3
4

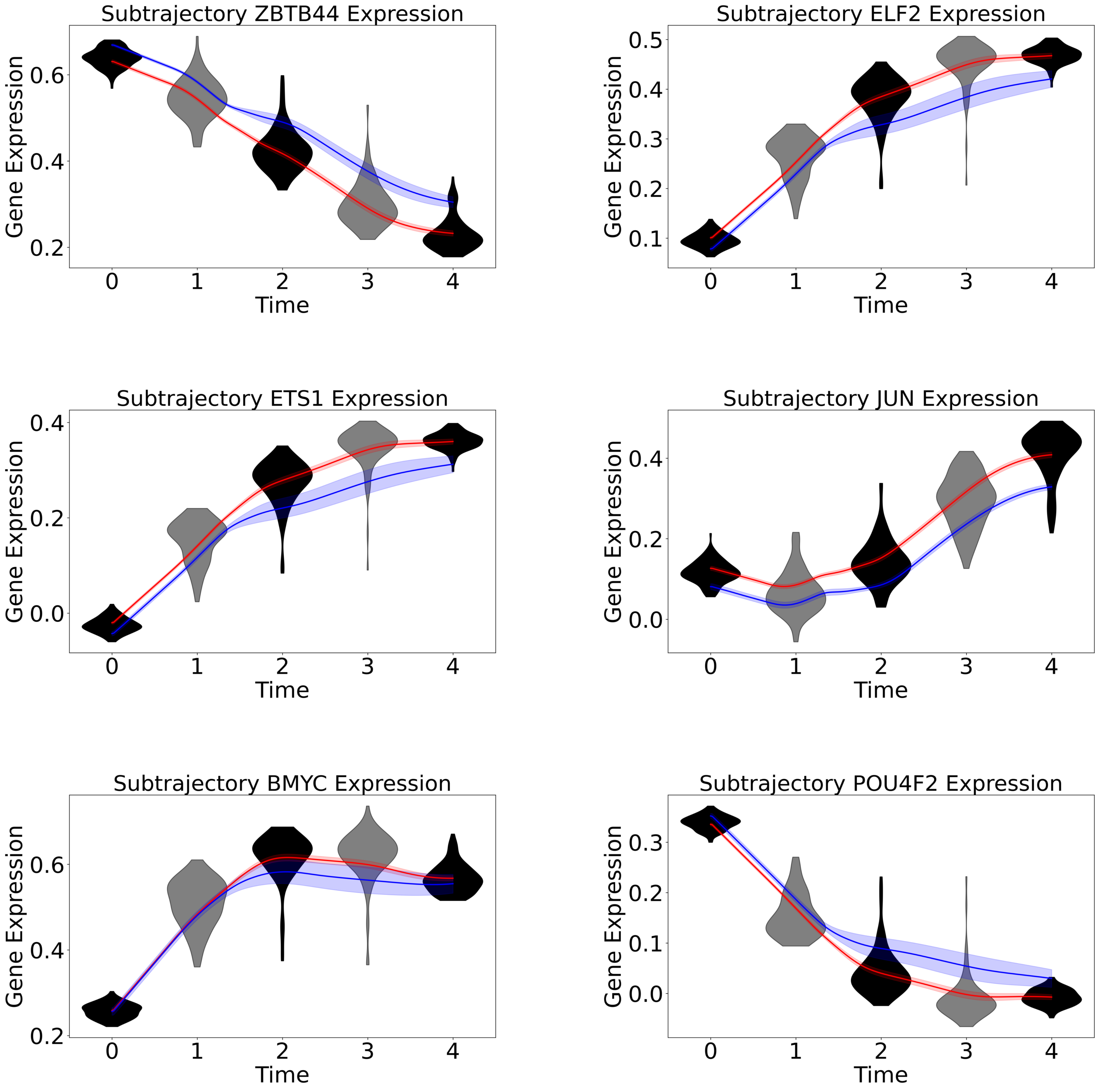

Subtrajectory ZBTB44 Expression
Gene Expression
0.2
0.4
0.6
0 1 2 3 4
Time
Subtrajectory ELF2 Expression
Gene Expression
0.1
0.2
0.3
0.4
0.5
0 1 2 3 4
Time
Subtrajectory ETS1 Expression
Gene Expression
0.0
0.2
0.4
0 1 2 3 4
Time
Subtrajectory JUN Expression
Gene Expression
0.0
0.2
0.4
0 1 2 3 4
Time
Subtrajectory BMYC Expression
Gene Expression
0.2
0.4
0.6
0 1 2 3 4
Time
Subtrajectory POU4F2 Expression
Gene Expression
0.0
0.1
0.2
0.3
0 1 2 3 4
Time

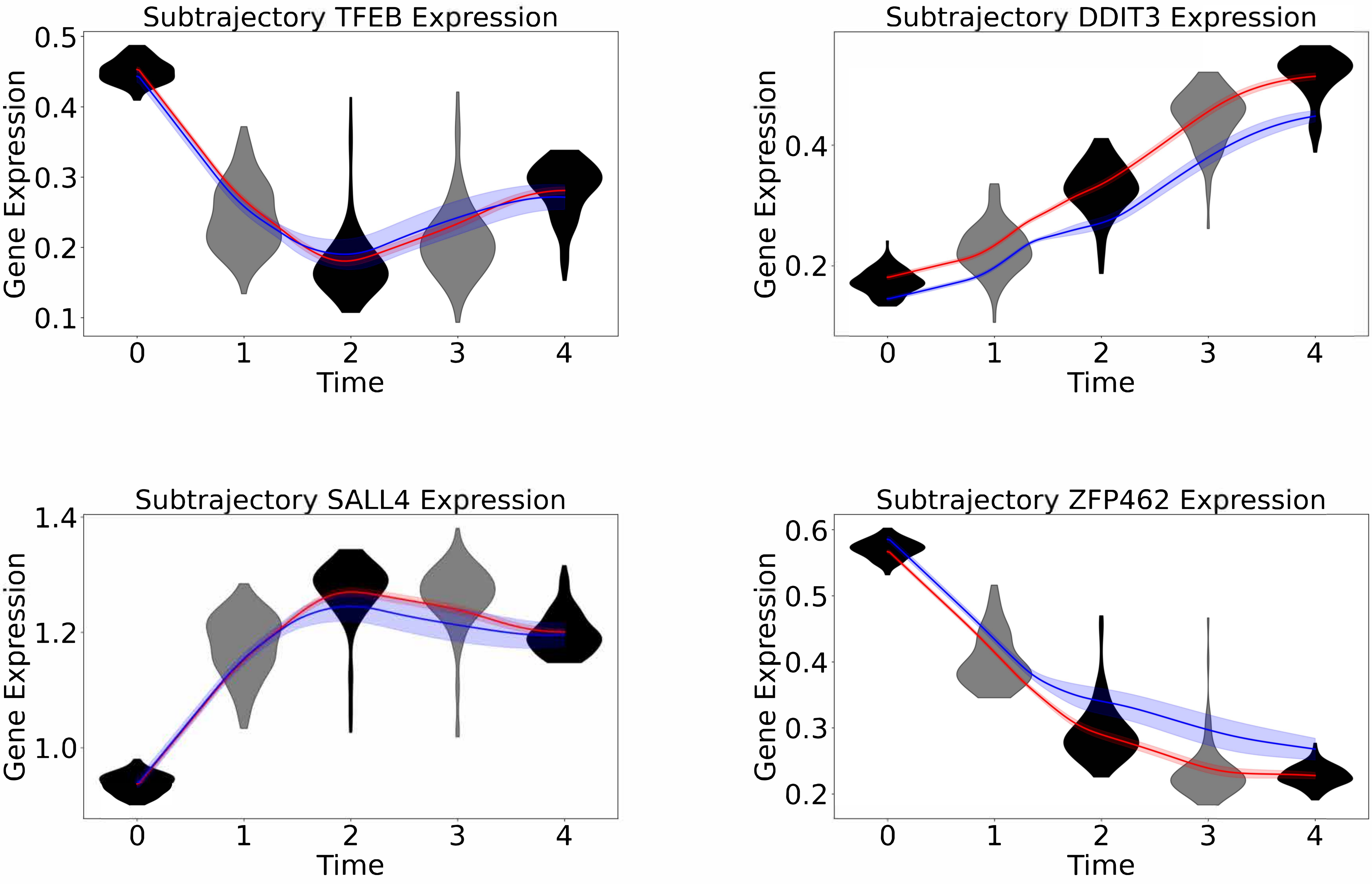

Supplementary Figure 5: Average gene expression trajectories plotted separately for each subgroup (shaded regions indicate 95% confidence intervals).
