# Supplementary material for: PROFET Predicts Continuous Gene Expression Dynamics from scRNA-seq Data to Elucidate Heterogeneity of Cancer Treatment Responses
Source: bioRxiv. 2025 Jul 3:2025.06.27.662030. Preprint. [Version 1] doi: 10.1101/2025.06.27.662030 (PMC12236938; doi:10.1101/2025.06.27.662030)
Supplement: Supplement 6 [file media-7.pdf]

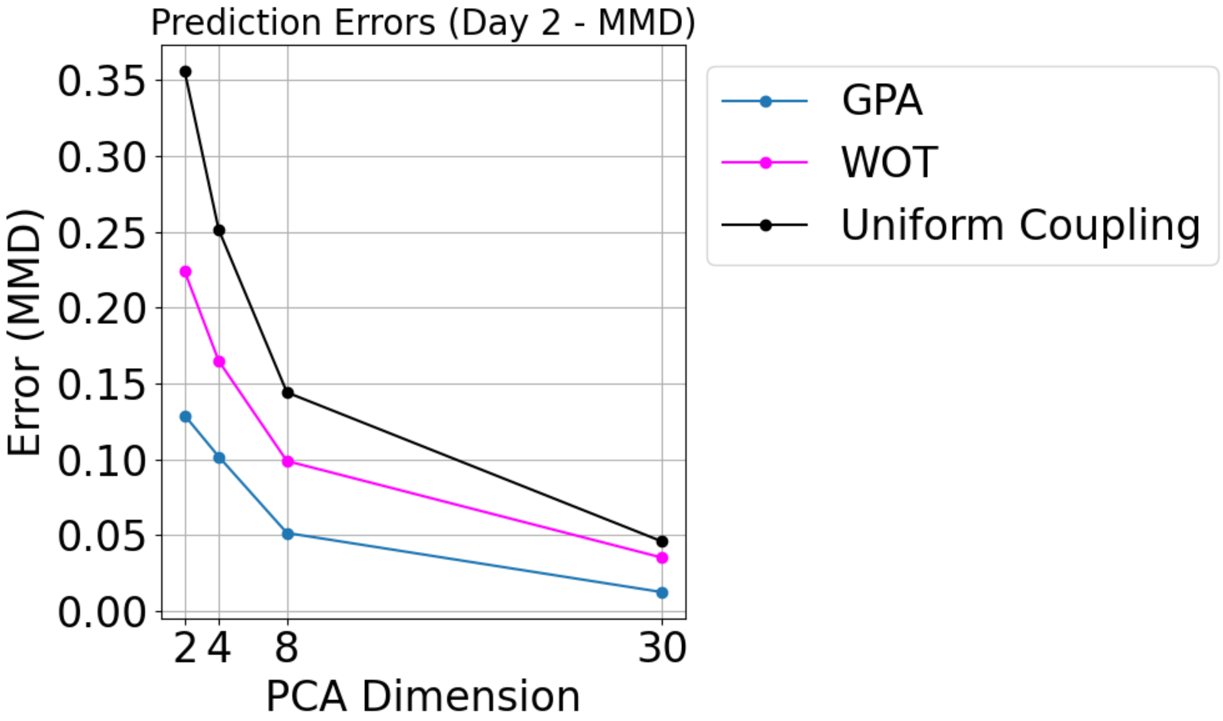

Prediction Errors (Day 2 - MMD)
Error (MMD)
0.00
0.05
0.10
0.15
0.20
0.25
0.30
0.35
2
4
8
30
PCA Dimension
GPA
WOT
Uniform Coupling

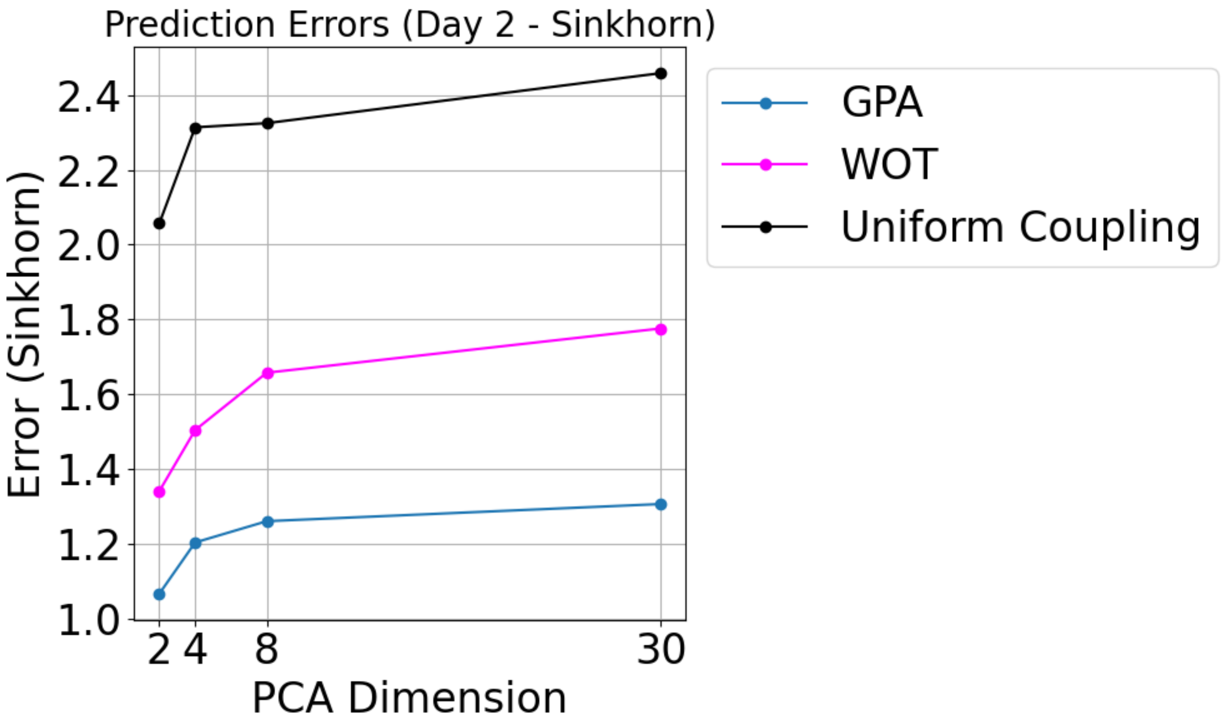

Prediction Errors (Day 2 - Sinkhorn)
Error (Sinkhorn)
1.0
1.2
1.4
1.6
1.8
2.0
2.2
2.4
2
4
8
30
PCA Dimension
GPA
WOT
Uniform Coupling

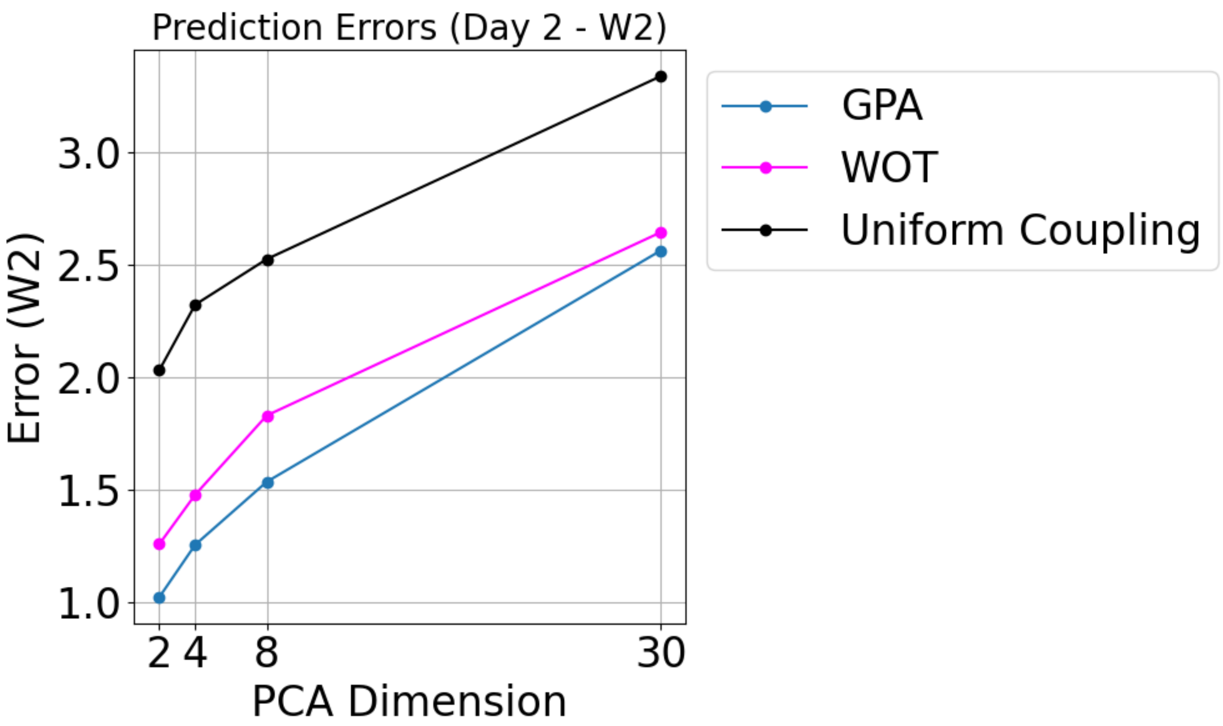

Supplementary Figure 6: Error distances between predicted and test data (Day 2) across different metrics.
