# Supplementary material for: PROFET Predicts Continuous Gene Expression Dynamics from scRNA-seq Data to Elucidate Heterogeneity of Cancer Treatment Responses
Source: bioRxiv. 2025 Jul 3:2025.06.27.662030. Preprint. [Version 1] doi: 10.1101/2025.06.27.662030 (PMC12236938; doi:10.1101/2025.06.27.662030)
Supplement: Supplement 7 [file media-8.pdf]

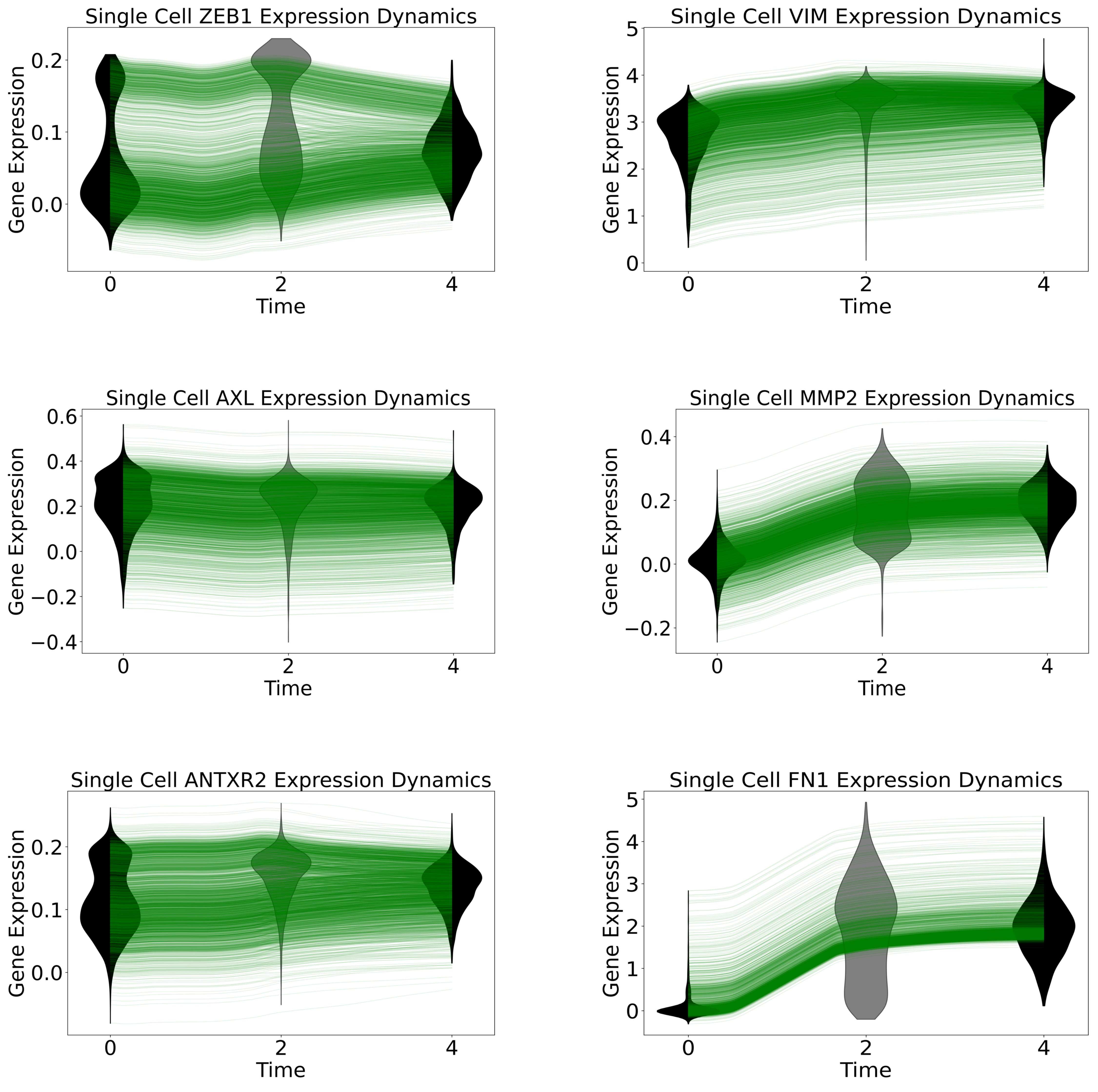
Single Cell ZEB1 Expression Dynamics
Single Cell VIM Expression Dynamics
Single Cell AXL Expression Dynamics
Single Cell MMP2 Expression Dynamics
Single Cell ANTXR2 Expression Dynamics
Single Cell FN1 Expression Dynamics
Gene Expression
Time

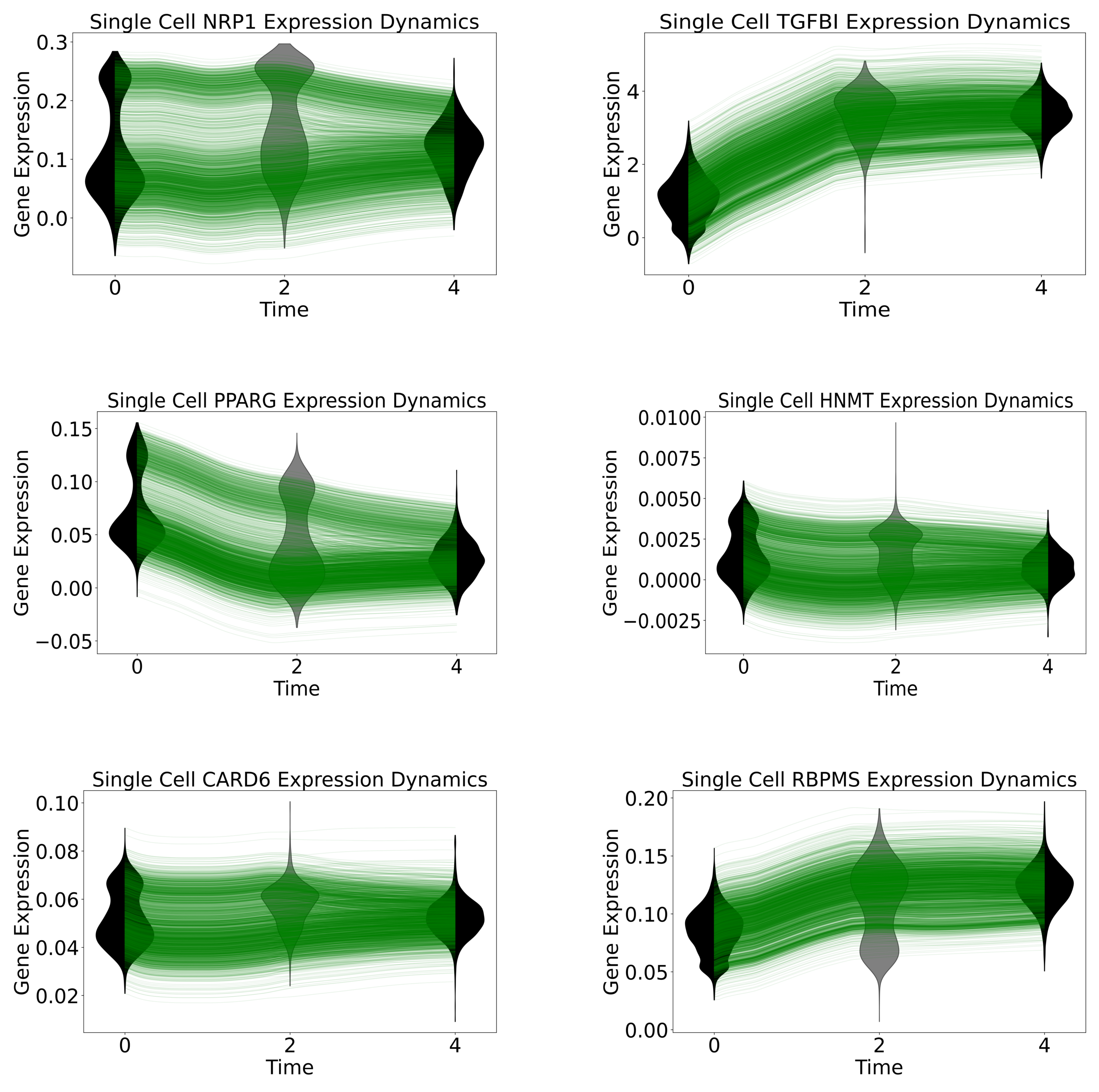
Single Cell NRP1 Expression Dynamics
Single Cell TGFBI Expression Dynamics
Single Cell PPARG Expression Dynamics
Single Cell HNMT Expression Dynamics
Single Cell CARD6 Expression Dynamics
Single Cell RBPMS Expression Dynamics
Gene Expression
Time

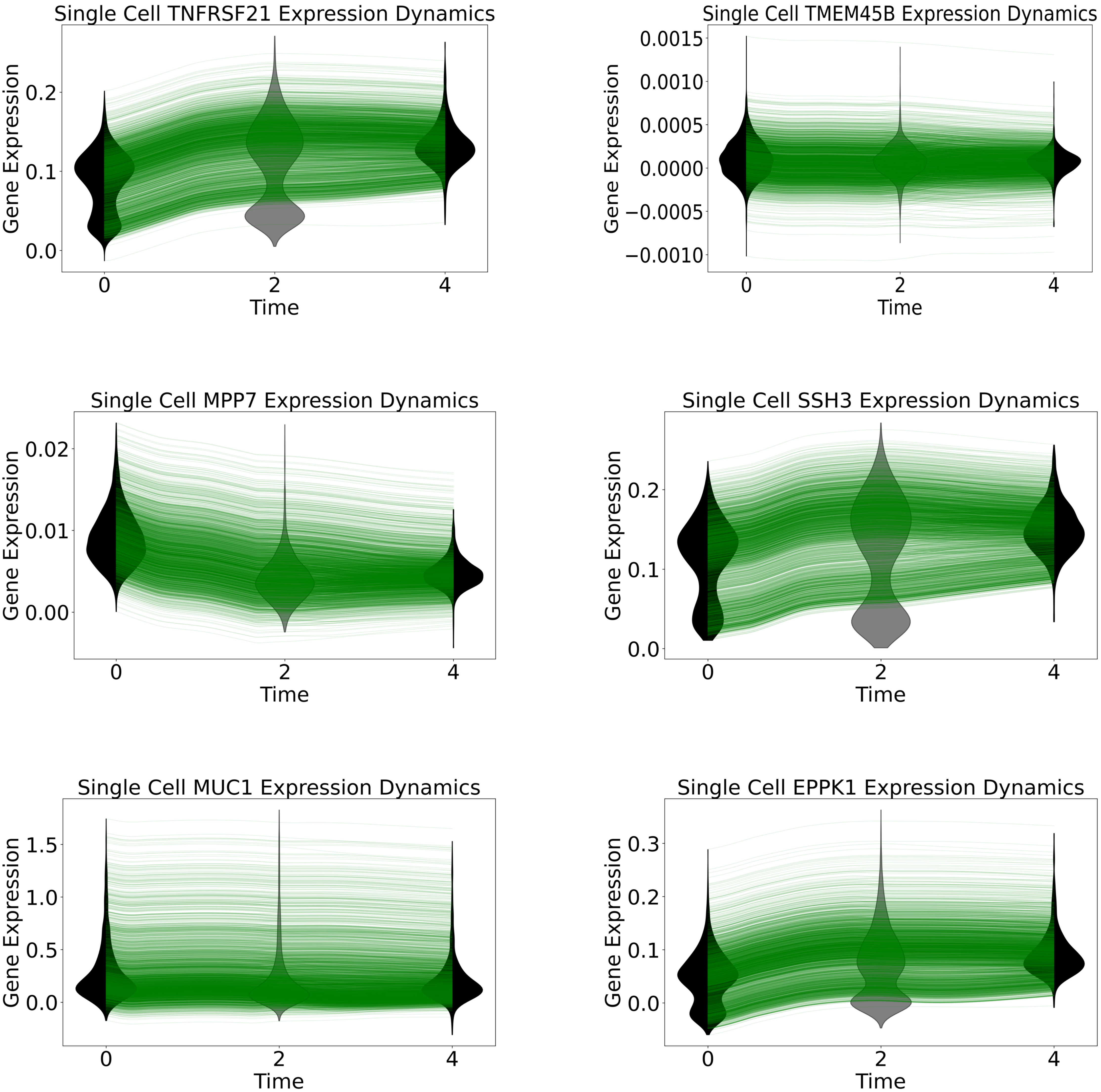
Single Cell TNFRSF21 Expression Dynamics
Gene Expression
0.0
0.1
0.2
0
2
4
Time
Single Cell TMEM45B Expression Dynamics
Gene Expression
0.0015
0.0010
0.0005
0.0000
−0.0005
−0.0010
0
2
4
Time
Single Cell MPP7 Expression Dynamics
Gene Expression
0.02
0.01
0.00
0
2
4
Time
Single Cell SSH3 Expression Dynamics
Gene Expression
0.2
0.1
0.0
0
2
4
Time
Single Cell MUC1 Expression Dynamics
Gene Expression
1.5
1.0
0.5
0.0
0
2
4
Time
Single Cell EPPK1 Expression Dynamics
Gene Expression
0.3
0.2
0.1
0.0
0
2
4
Time

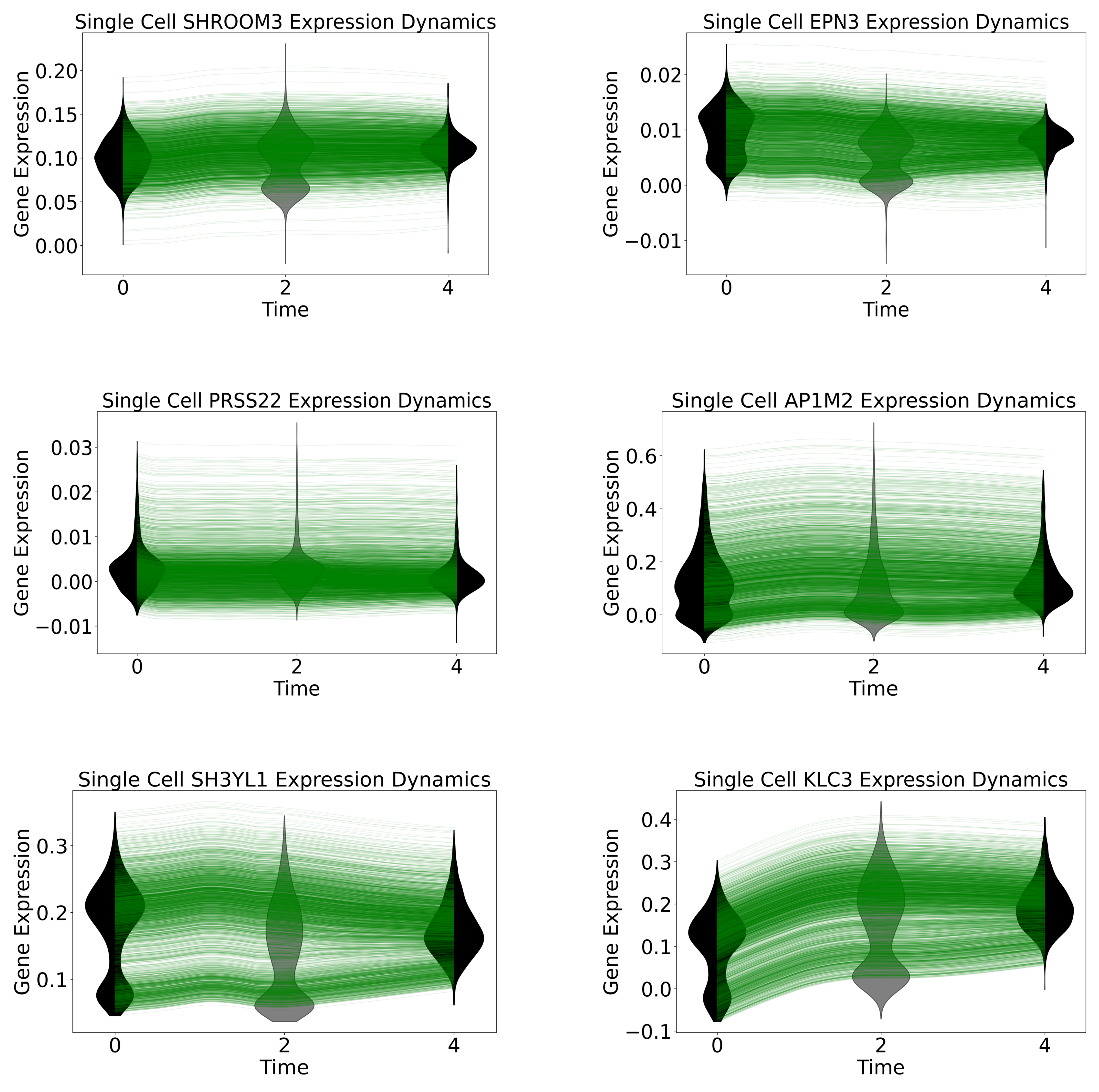
Single Cell SHROOM3 Expression Dynamics
Single Cell EPN3 Expression Dynamics
Single Cell PRSS22 Expression Dynamics
Single Cell AP1M2 Expression Dynamics
Single Cell SH3YL1 Expression Dynamics
Single Cell KLC3 Expression Dynamics
Gene Expression
Time
0
2
4

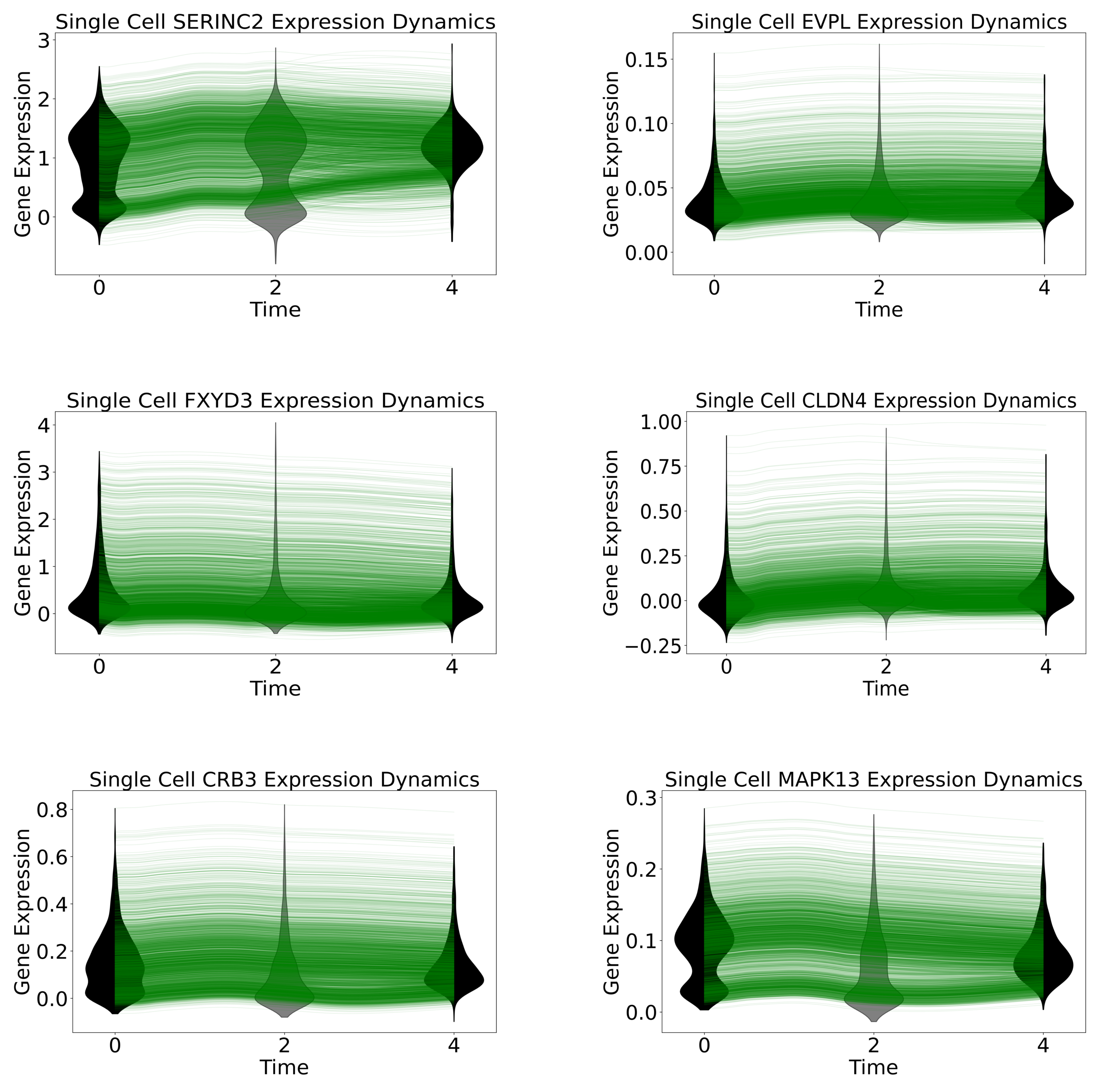
Single Cell SERINC2 Expression Dynamics
Single Cell EVPL Expression Dynamics
Single Cell FXYD3 Expression Dynamics
Single Cell CLDN4 Expression Dynamics
Single Cell CRB3 Expression Dynamics
Single Cell MAPK13 Expression Dynamics
Gene Expression
Time

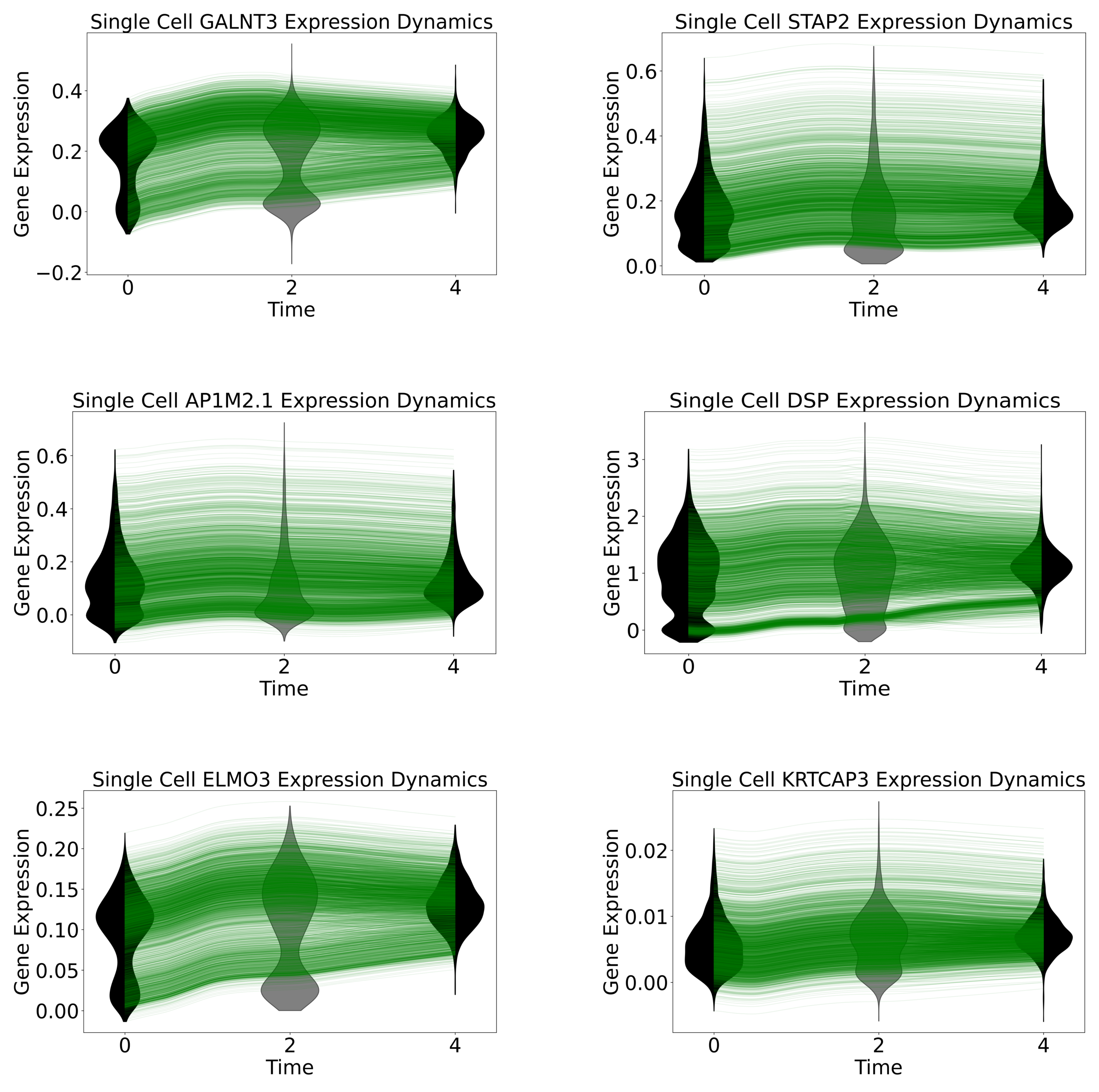

Single Cell GALNT3 Expression Dynamics
Gene Expression
Time
Single Cell STAP2 Expression Dynamics
Gene Expression
Time
Single Cell AP1M2.1 Expression Dynamics
Gene Expression
Time
Single Cell DSP Expression Dynamics
Gene Expression
Time
Single Cell ELMO3 Expression Dynamics
Gene Expression
Time
Single Cell KRTCAP3 Expression Dynamics
Gene Expression
Time

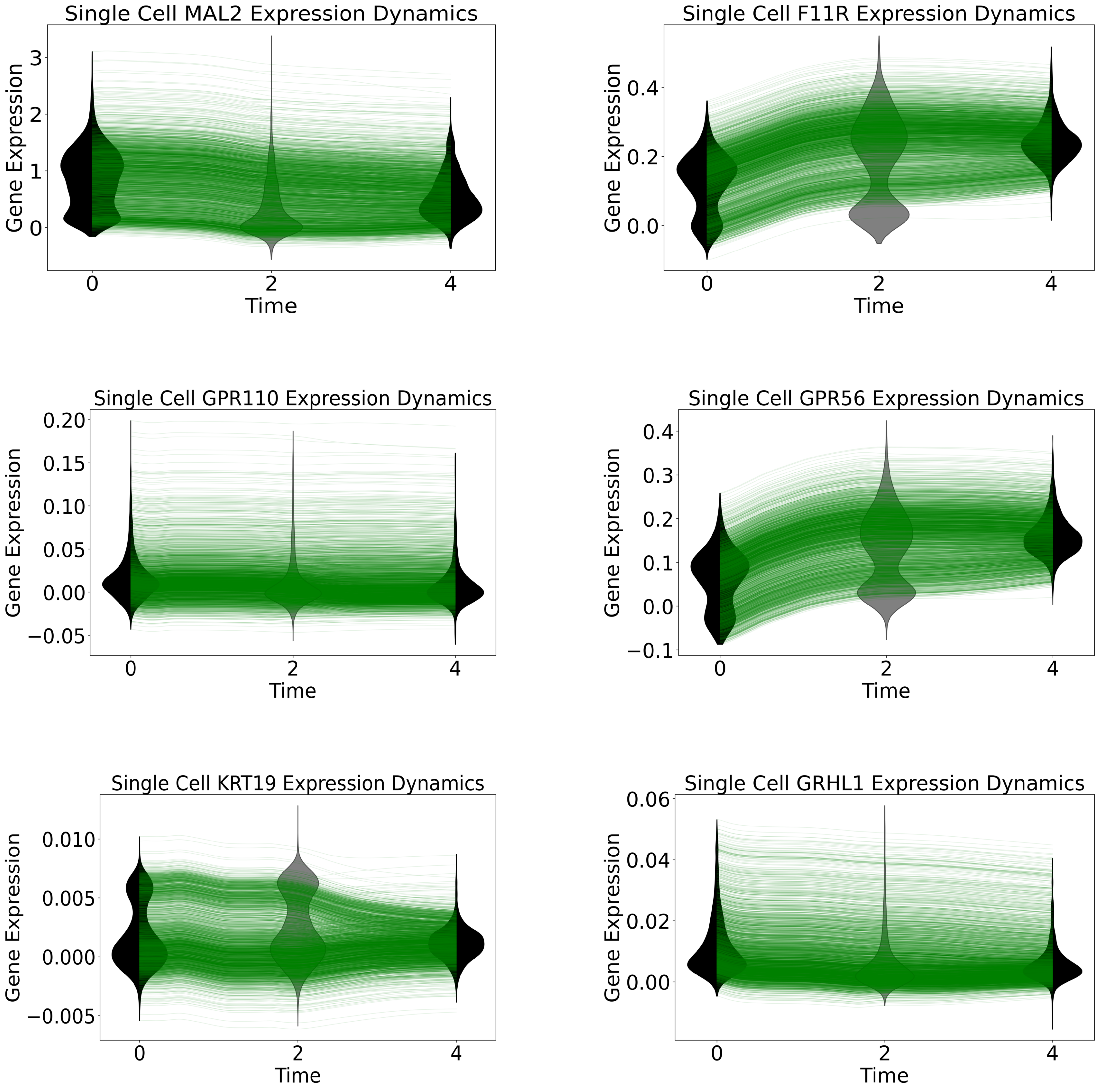
Single Cell MAL2 Expression Dynamics
Gene Expression
Time
Single Cell F11R Expression Dynamics
Gene Expression
Time
Single Cell GPR110 Expression Dynamics
Gene Expression
Time
Single Cell GPR56 Expression Dynamics
Gene Expression
Time
Single Cell KRT19 Expression Dynamics
Gene Expression
Time
Single Cell GRHL1 Expression Dynamics
Gene Expression
Time

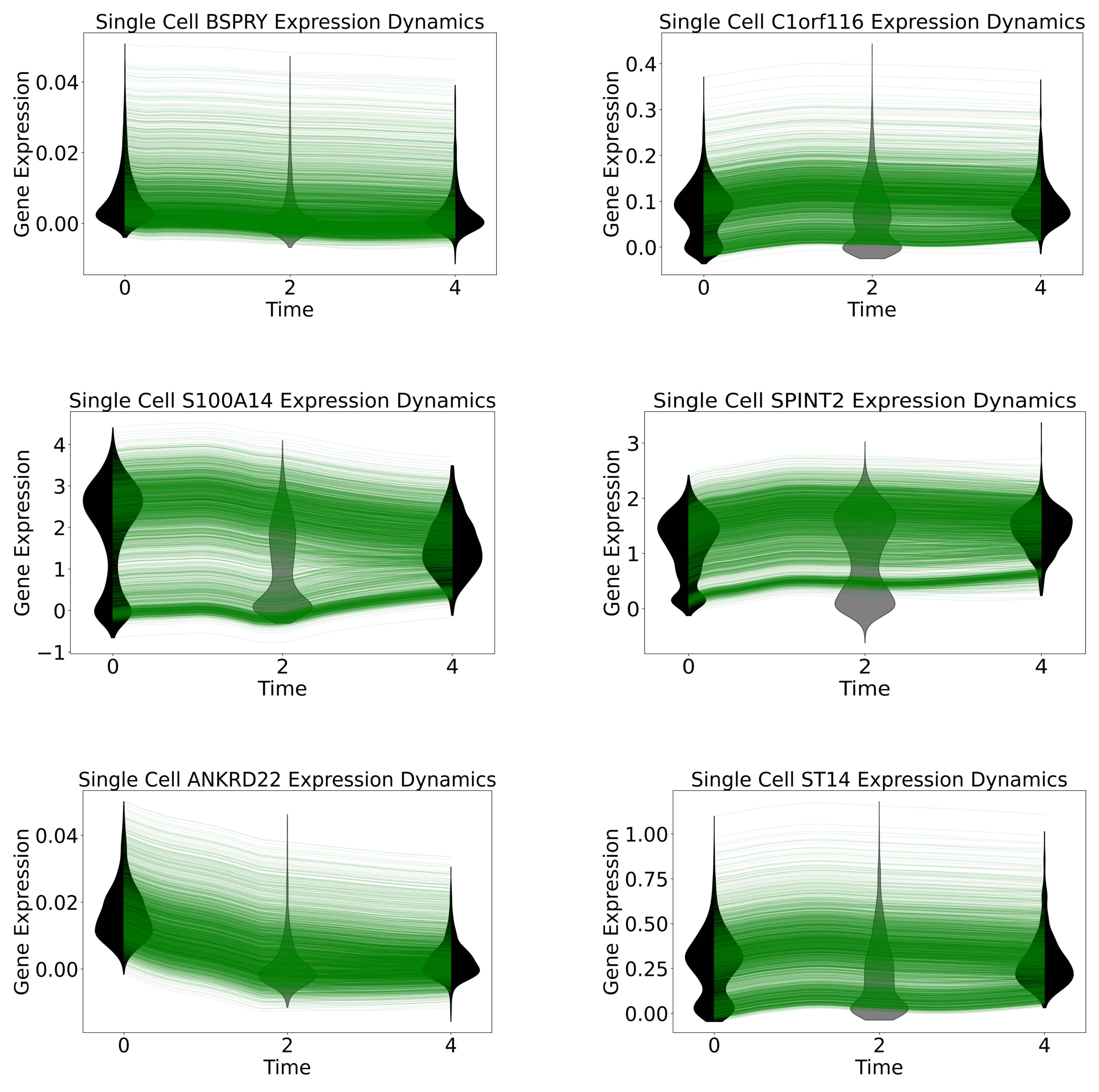

Single Cell BSPRY Expression Dynamics
Single Cell C1orf116 Expression Dynamics
Single Cell S100A14 Expression Dynamics
Single Cell SPINT2 Expression Dynamics
Single Cell ANKRD22 Expression Dynamics
Single Cell ST14 Expression Dynamics
Gene Expression
Time

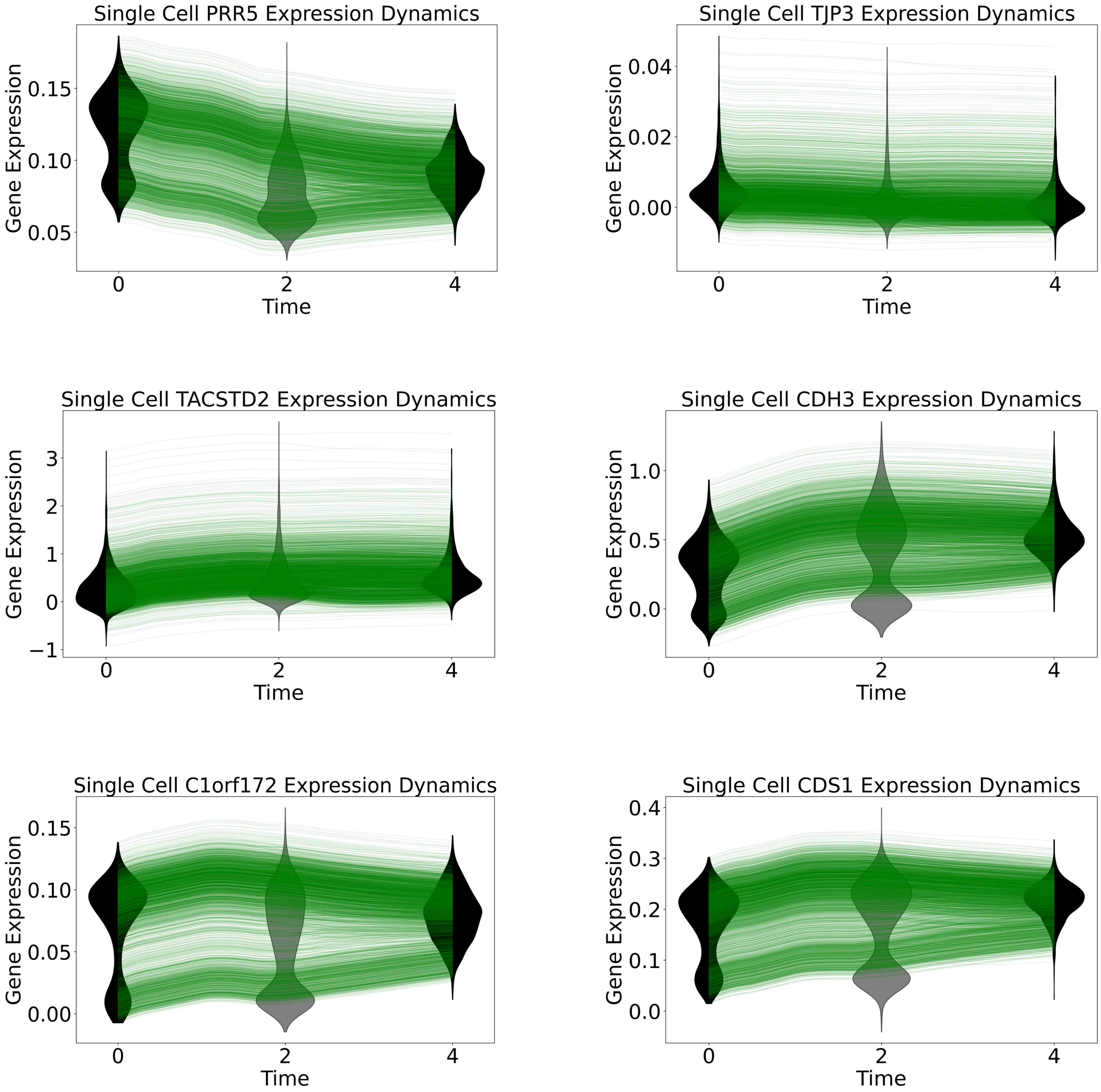
Single Cell PRR5 Expression Dynamics
Gene Expression
0.05
0.10
0.15
Time
0
2
4
Single Cell TJP3 Expression Dynamics
0.00
0.02
0.04
Single Cell TACSTD2 Expression Dynamics
−1
0
1
2
3
Single Cell CDH3 Expression Dynamics
0.0
0.5
1.0
Single Cell C1orf172 Expression Dynamics
0.00
0.05
0.10
0.15
Single Cell CDS1 Expression Dynamics
0.0
0.1
0.2
0.3
0.4

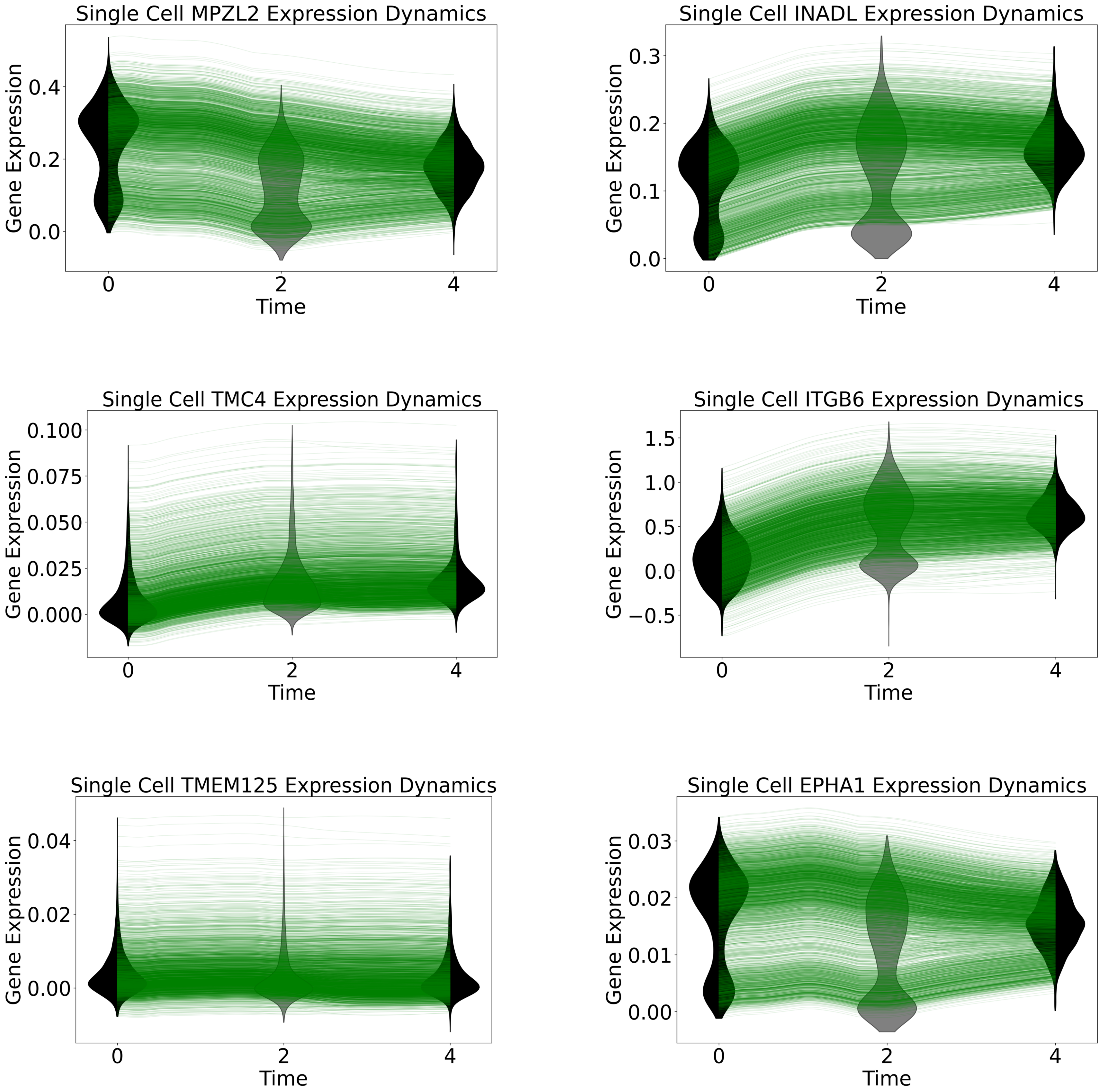
Single Cell MPZL2 Expression Dynamics
Single Cell INADL Expression Dynamics
Single Cell TMC4 Expression Dynamics
Single Cell ITGB6 Expression Dynamics
Single Cell TMEM125 Expression Dynamics
Single Cell EPHA1 Expression Dynamics
Gene Expression
Time
0
2
4

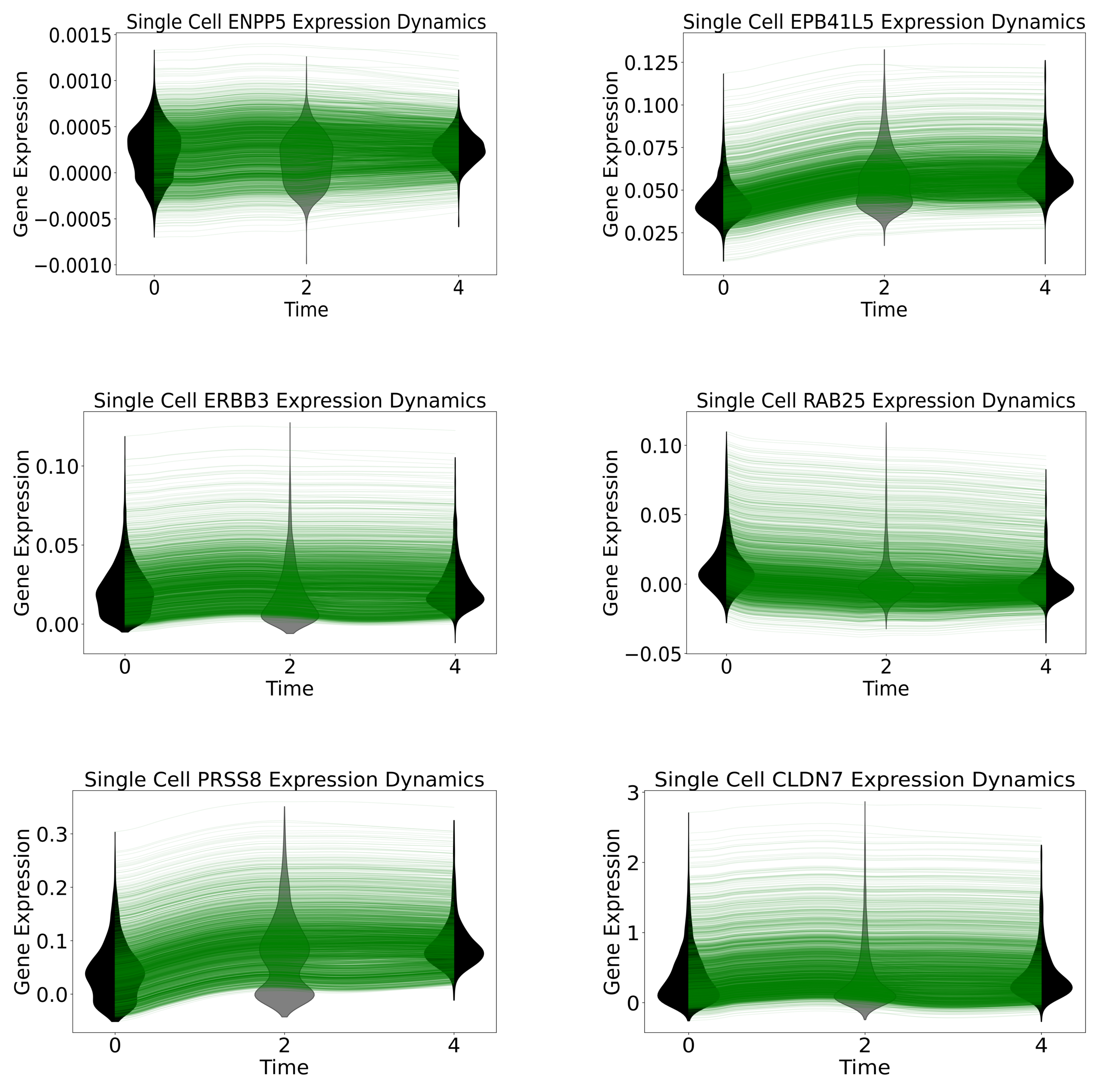
Single Cell ENPP5 Expression Dynamics
Gene Expression
Time
Single Cell EPB41L5 Expression Dynamics
Gene Expression
Time
Single Cell ERBB3 Expression Dynamics
Gene Expression
Time
Single Cell RAB25 Expression Dynamics
Gene Expression
Time
Single Cell PRSS8 Expression Dynamics
Gene Expression
Time
Single Cell CLDN7 Expression Dynamics
Gene Expression
Time

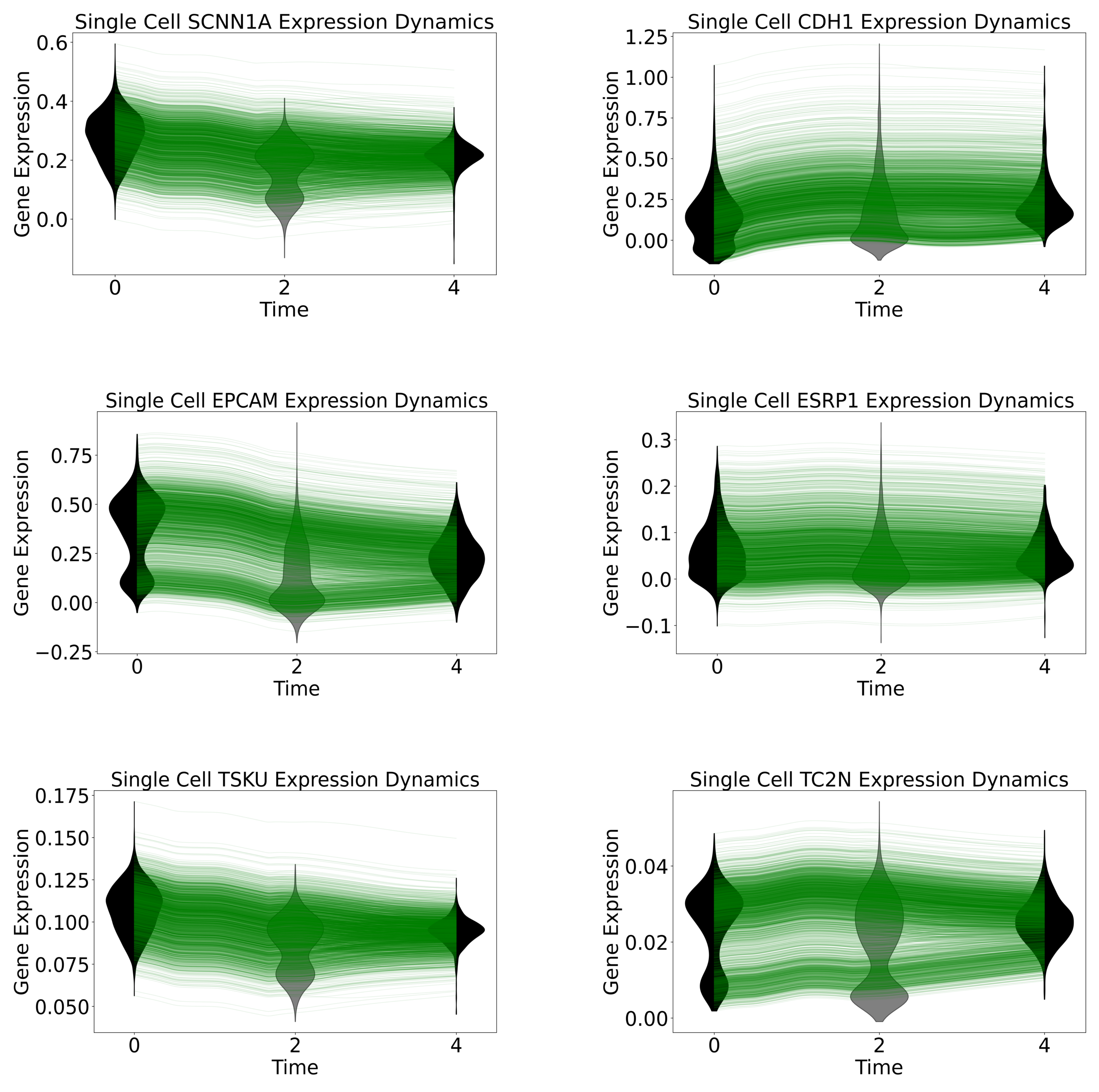
Single Cell SCNN1A Expression Dynamics
Gene Expression
0.6
0.4
0.2
0.0
Time
0
2
4
Single Cell CDH1 Expression Dynamics
Gene Expression
1.25
1.00
0.75
0.50
0.25
0.00
Time
0
2
4
Single Cell EPCAM Expression Dynamics
Gene Expression
0.75
0.50
0.25
0.00
−0.25
Time
0
2
4
Single Cell ESRP1 Expression Dynamics
Gene Expression
0.3
0.2
0.1
0.0
−0.1
Time
0
2
4
Single Cell TSKU Expression Dynamics
Gene Expression
0.175
0.150
0.125
0.100
0.075
0.050
Time
0
2
4
Single Cell TC2N Expression Dynamics
Gene Expression
0.04
0.02
0.00
Time
0
2
4

Supplementary Figure 7: Single-cell, single-gene dynamics plotted as gene expression levels (y-axis) over time (x-axis), with each green curve representing an individual cell trajectory. The predicted trajectories are compared against real data distributions using violin plots (gray for test data, black for training data).
