# Supplementary material for: PROFET Predicts Continuous Gene Expression Dynamics from scRNA-seq Data to Elucidate Heterogeneity of Cancer Treatment Responses
Source: bioRxiv. 2025 Jul 3:2025.06.27.662030. Preprint. [Version 1] doi: 10.1101/2025.06.27.662030 (PMC12236938; doi:10.1101/2025.06.27.662030)
Supplement: Supplement 8 [file media-9.pdf]

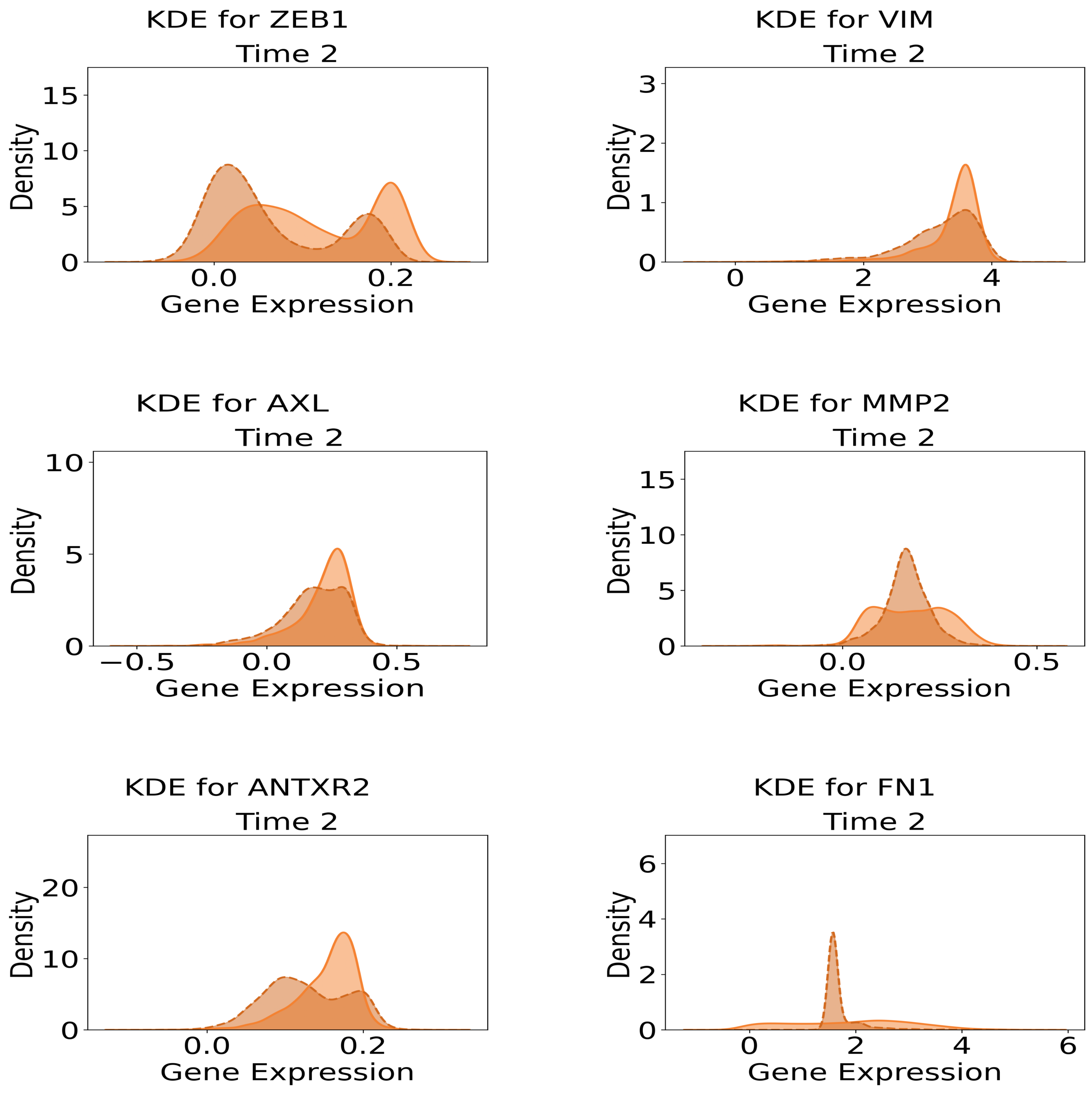

KDE for ZEB1
Time 2
KDE for VIM
Time 2
KDE for AXL
Time 2
KDE for MMP2
Time 2
KDE for ANTXR2
Time 2
KDE for FN1
Time 2
Density
Gene Expression

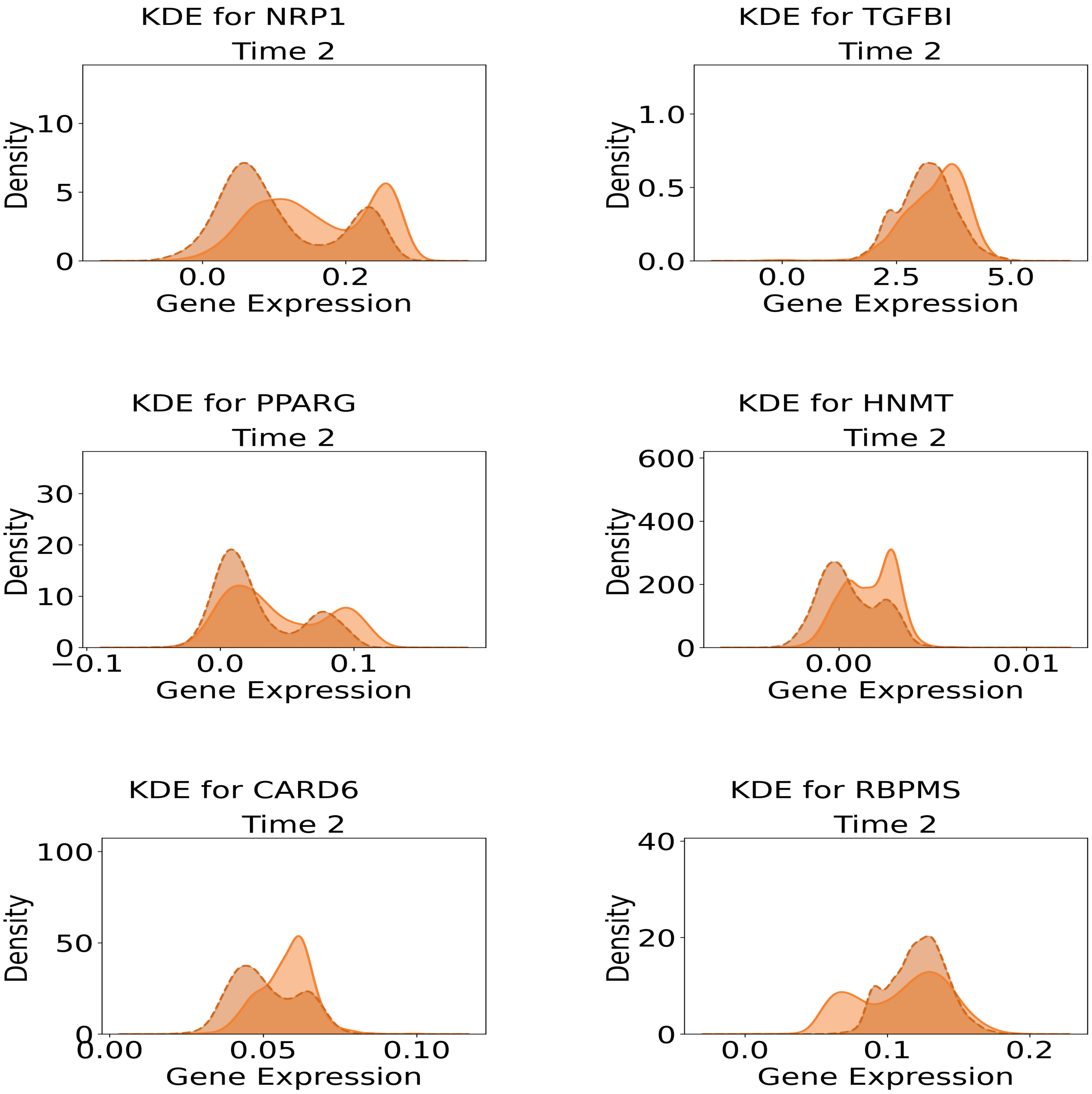

KDE for NRP1
Time 2
Density
Gene Expression
0
5
10
0.0
0.2
KDE for TGFBI
Time 2
Density
Gene Expression
0.0
0.5
1.0
0.0
2.5
5.0
KDE for PPARG
Time 2
Density
Gene Expression
0
10
20
30
−0.1
0.0
0.1
KDE for HNMT
Time 2
Density
Gene Expression
0
200
400
600
0.00
0.01
KDE for CARD6
Time 2
Density
Gene Expression
0
50
100
0.00
0.05
0.10
KDE for RBPMS
Time 2
Density
Gene Expression
0
20
40
0.0
0.1
0.2

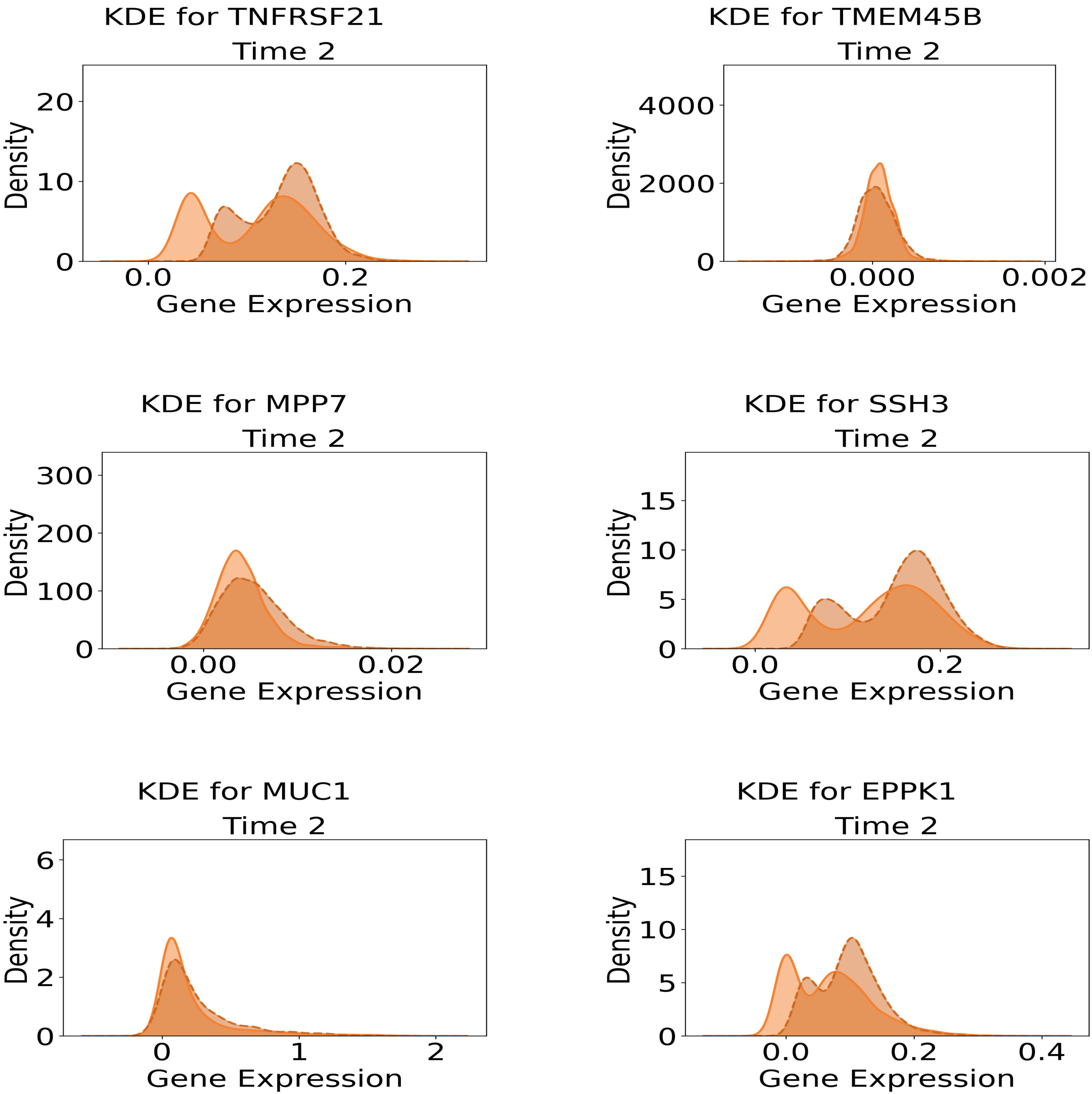
KDE for TNFRSF21
Time 2
Density
Gene Expression
0
10
20
0.0
0.2
KDE for TMEM45B
Time 2
Density
Gene Expression
0
2000
4000
0.000
0.002
KDE for MPP7
Time 2
Density
Gene Expression
0
100
200
300
0.00
0.02
KDE for SSH3
Time 2
Density
Gene Expression
0
5
10
15
0.0
0.2
KDE for MUC1
Time 2
Density
Gene Expression
0
2
4
6
0
1
2
KDE for EPPK1
Time 2
Density
Gene Expression
0
5
10
15
0.0
0.2
0.4

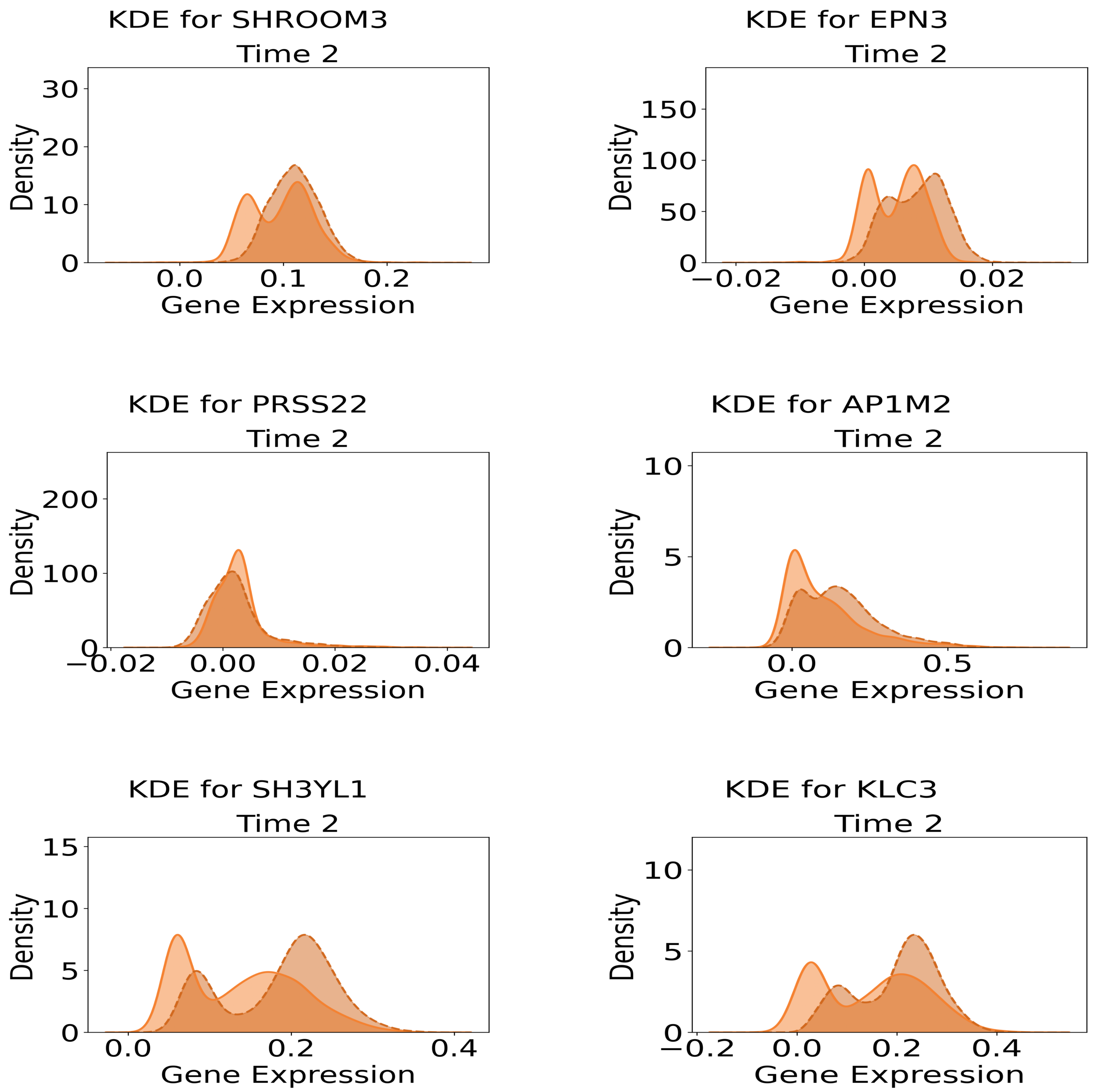
KDE for SHROOM3
Time 2
Density
Gene Expression
0
10
20
30
0.0
0.1
0.2
KDE for EPN3
Time 2
Density
Gene Expression
0
50
100
150
−0.02
0.00
0.02
KDE for PRSS22
Time 2
Density
Gene Expression
0
100
200
−0.02
0.00
0.02
0.04
KDE for AP1M2
Time 2
Density
Gene Expression
0
5
10
0.0
0.5
KDE for SH3YL1
Time 2
Density
Gene Expression
0
5
10
15
0.0
0.2
0.4
KDE for KLC3
Time 2
Density
Gene Expression
0
5
10
−0.2
0.0
0.2
0.4

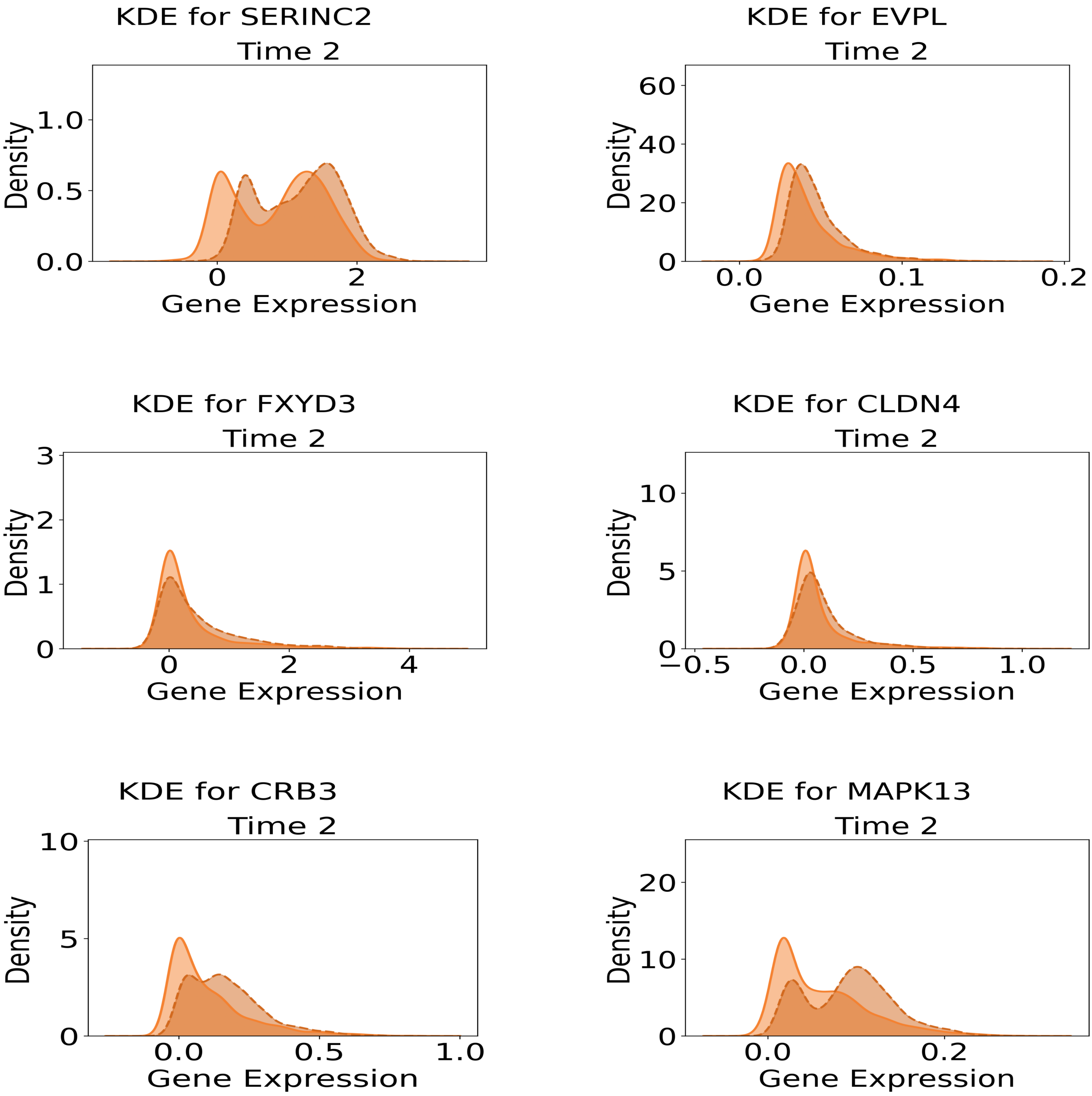

KDE for SERINC2
Time 2
Density
1.0
0.5
0.0
0
2
Gene Expression
KDE for EVPL
Time 2
Density
60
40
20
0
0.0
0.1
0.2
Gene Expression
KDE for FXYD3
Time 2
Density
3
2
1
0
0
2
4
Gene Expression
KDE for CLDN4
Time 2
Density
10
5
0
−0.5
0.0
0.5
1.0
Gene Expression
KDE for CRB3
Time 2
Density
10
5
0
0.0
0.5
1.0
Gene Expression
KDE for MAPK13
Time 2
Density
20
10
0
0.0
0.2
Gene Expression

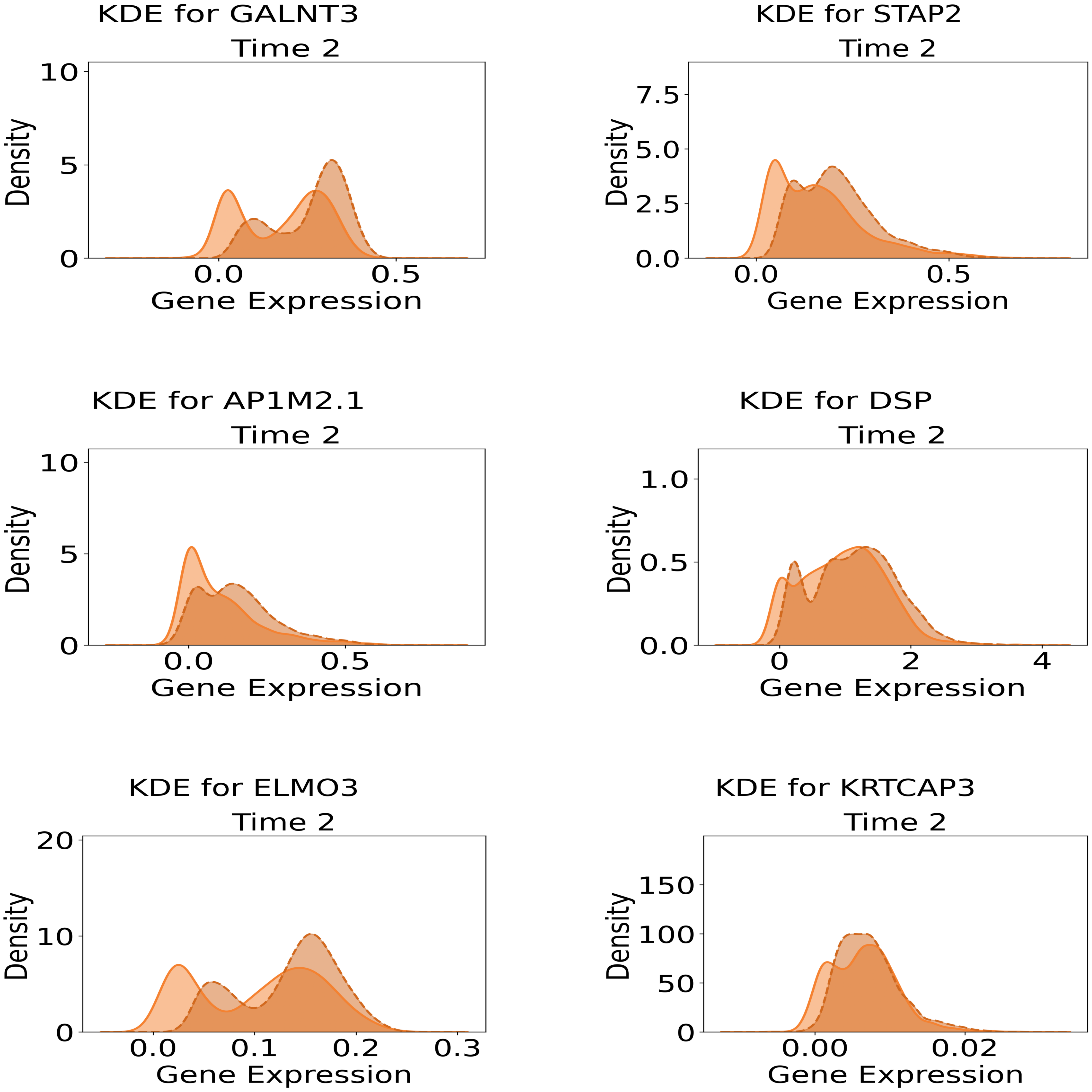
KDE for GALNT3
Time 2
Density
Gene Expression
KDE for STAP2
Time 2
Density
Gene Expression
KDE for AP1M2.1
Time 2
Density
Gene Expression
KDE for DSP
Time 2
Density
Gene Expression
KDE for ELMO3
Time 2
Density
Gene Expression
KDE for KRTCAP3
Time 2
Density
Gene Expression

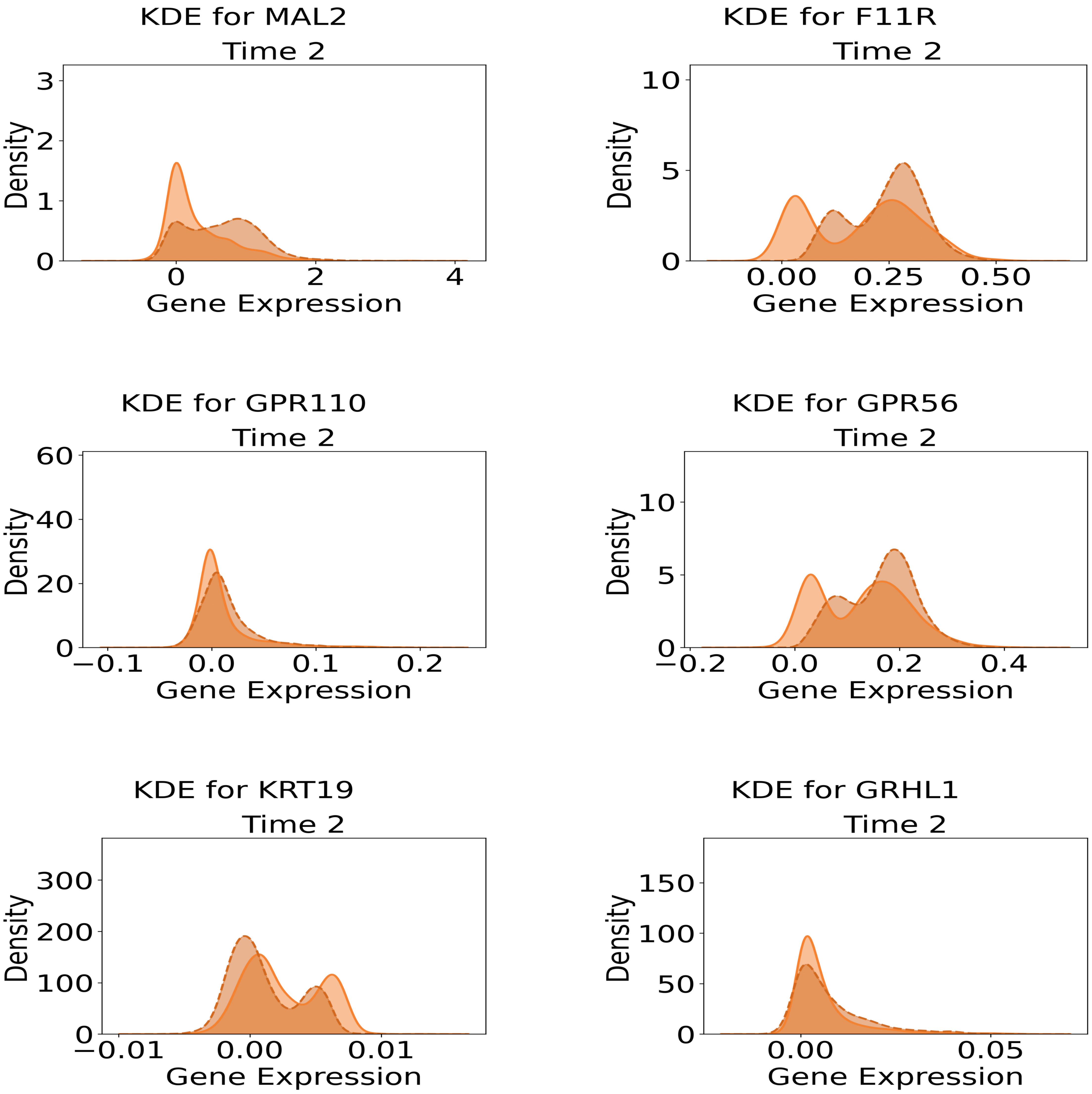
KDE for MAL2
Time 2
Density
Gene Expression
0
1
2
3
0
2
4
KDE for F11R
Time 2
Density
Gene Expression
0
5
10
0.00
0.25
0.50
KDE for GPR110
Time 2
Density
Gene Expression
0
20
40
60
−0.1
0.0
0.1
0.2
KDE for GPR56
Time 2
Density
Gene Expression
0
5
10
−0.2
0.0
0.2
0.4
KDE for KRT19
Time 2
Density
Gene Expression
0
100
200
300
−0.01
0.00
0.01
KDE for GRHL1
Time 2
Density
Gene Expression
0
50
100
150
0.00
0.05

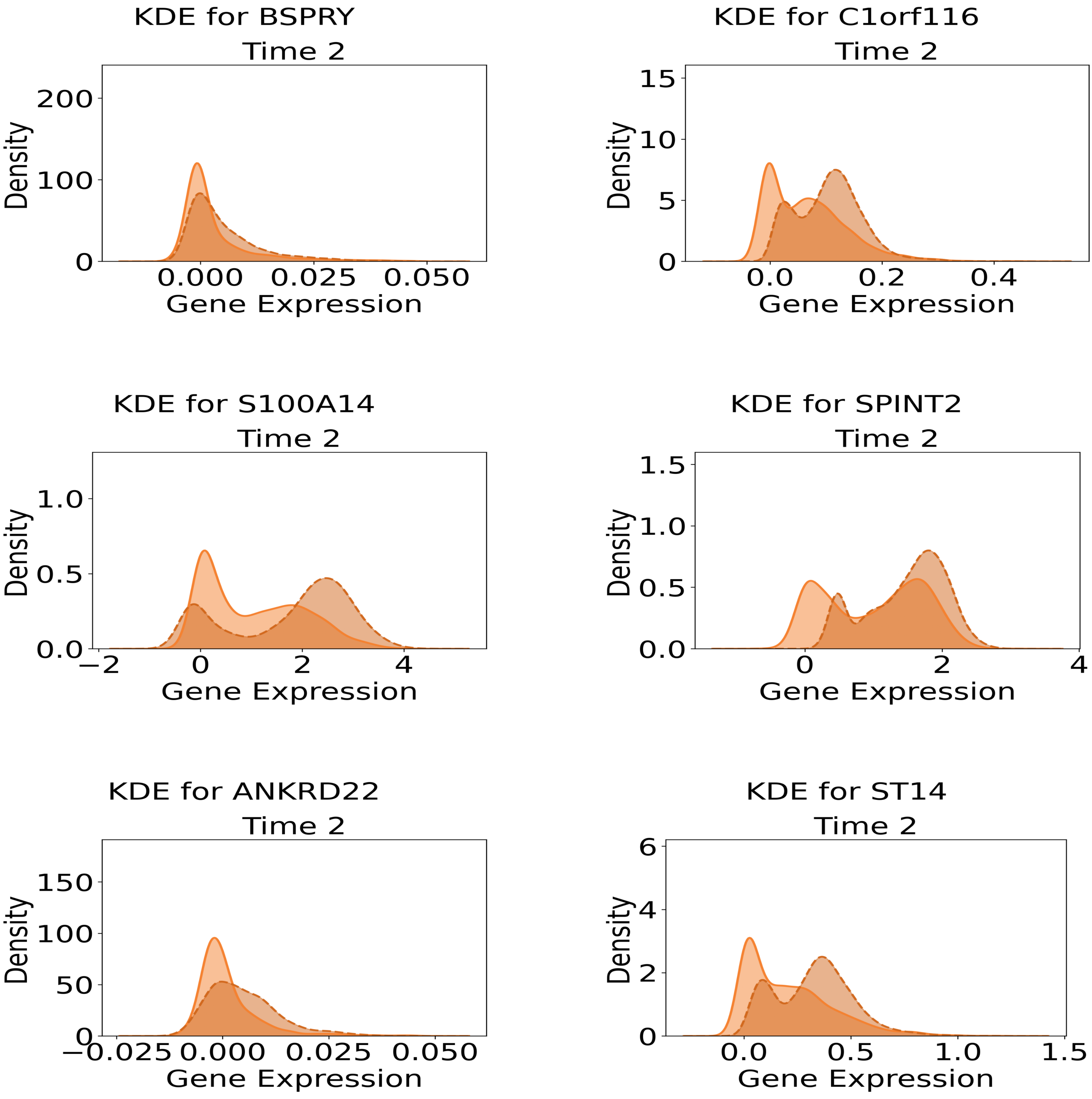
KDE for BSPRY
Time 2
Density
Gene Expression
0
100
200
0.000
0.025
0.050
KDE for C1orf116
Time 2
Density
Gene Expression
0
5
10
15
0.0
0.2
0.4
KDE for S100A14
Time 2
Density
Gene Expression
0.0
0.5
1.0
−2
0
2
4
KDE for SPINT2
Time 2
Density
Gene Expression
0.0
0.5
1.0
1.5
0
2
4
KDE for ANKRD22
Time 2
Density
Gene Expression
0
50
100
150
−0.025
0.000
0.025
0.050
KDE for ST14
Time 2
Density
Gene Expression
0
2
4
6
0.0
0.5
1.0
1.5

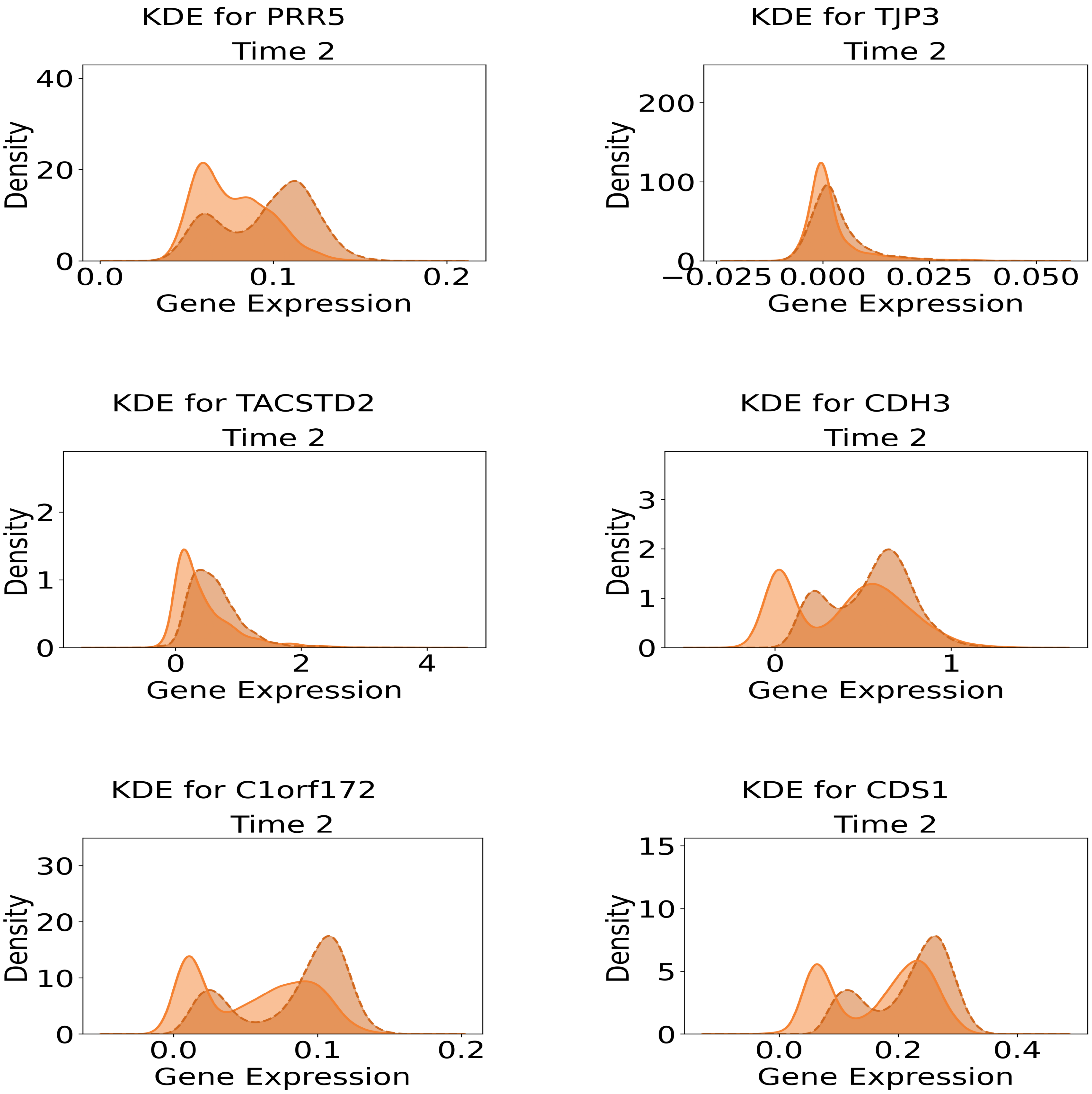
KDE for PRR5
Time 2
Density
Gene Expression
0
20
40
0.0
0.1
0.2
KDE for TJP3
Time 2
Density
Gene Expression
0
100
200
−0.025
0.000
0.025
0.050
KDE for TACSTD2
Time 2
Density
Gene Expression
0
1
2
0
2
4
KDE for CDH3
Time 2
Density
Gene Expression
0
1
2
3
0
1
KDE for C1orf172
Time 2
Density
Gene Expression
0
10
20
30
0.0
0.1
0.2
KDE for CDS1
Time 2
Density
Gene Expression
0
5
10
15
0.0
0.2
0.4

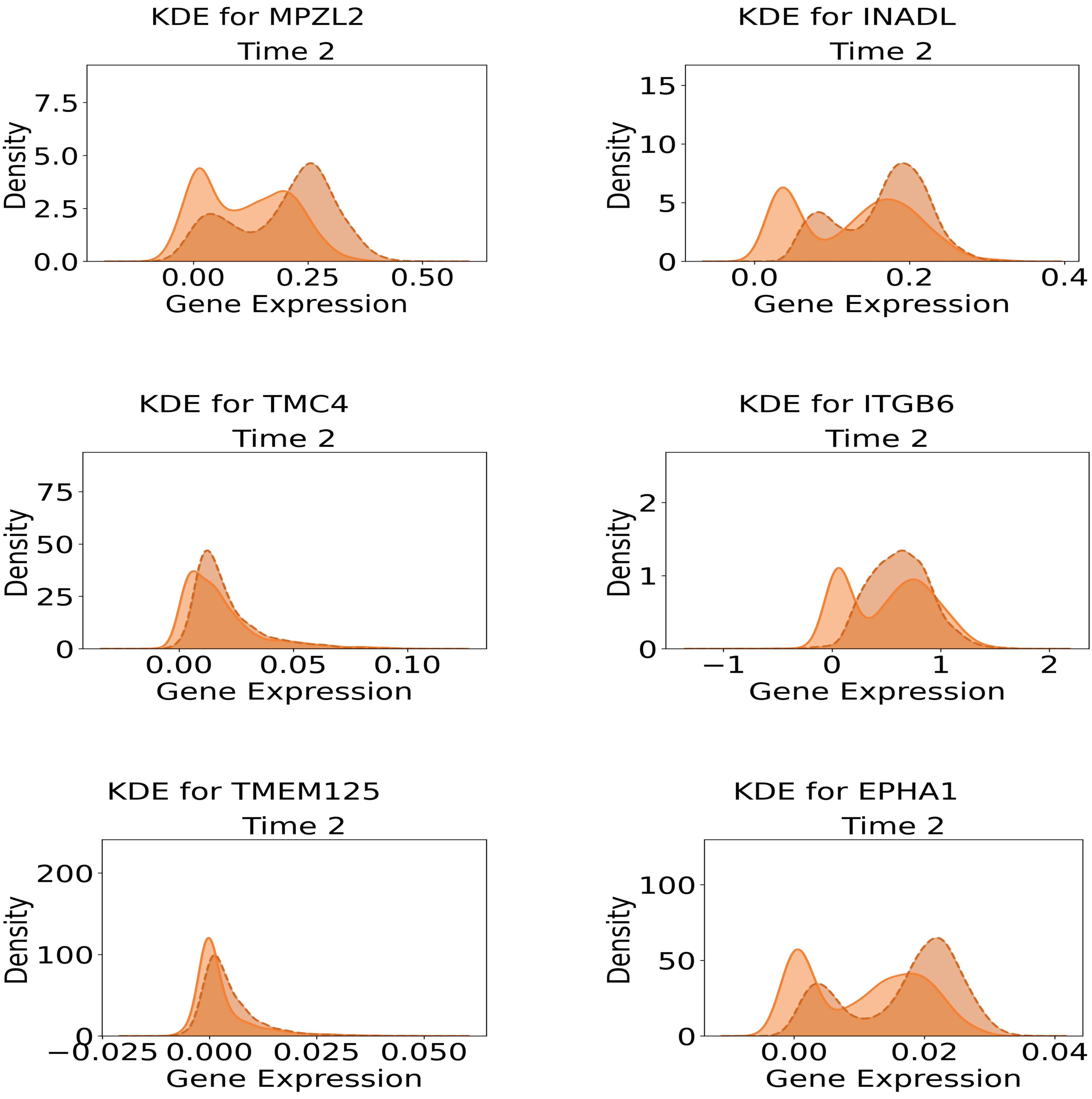
KDE for MPZL2
Time 2
Density
Gene Expression
0.0
2.5
5.0
7.5
0.00
0.25
0.50
KDE for INADL
Time 2
Density
Gene Expression
0
5
10
15
0.0
0.2
0.4
KDE for TMC4
Time 2
Density
Gene Expression
0
25
50
75
0.00
0.05
0.10
KDE for ITGB6
Time 2
Density
Gene Expression
0
1
2
−1
0
1
2
KDE for TMEM125
Time 2
Density
Gene Expression
0
100
200
−0.025
0.000
0.025
0.050
KDE for EPHA1
Time 2
Density
Gene Expression
0
50
100
0.00
0.02
0.04

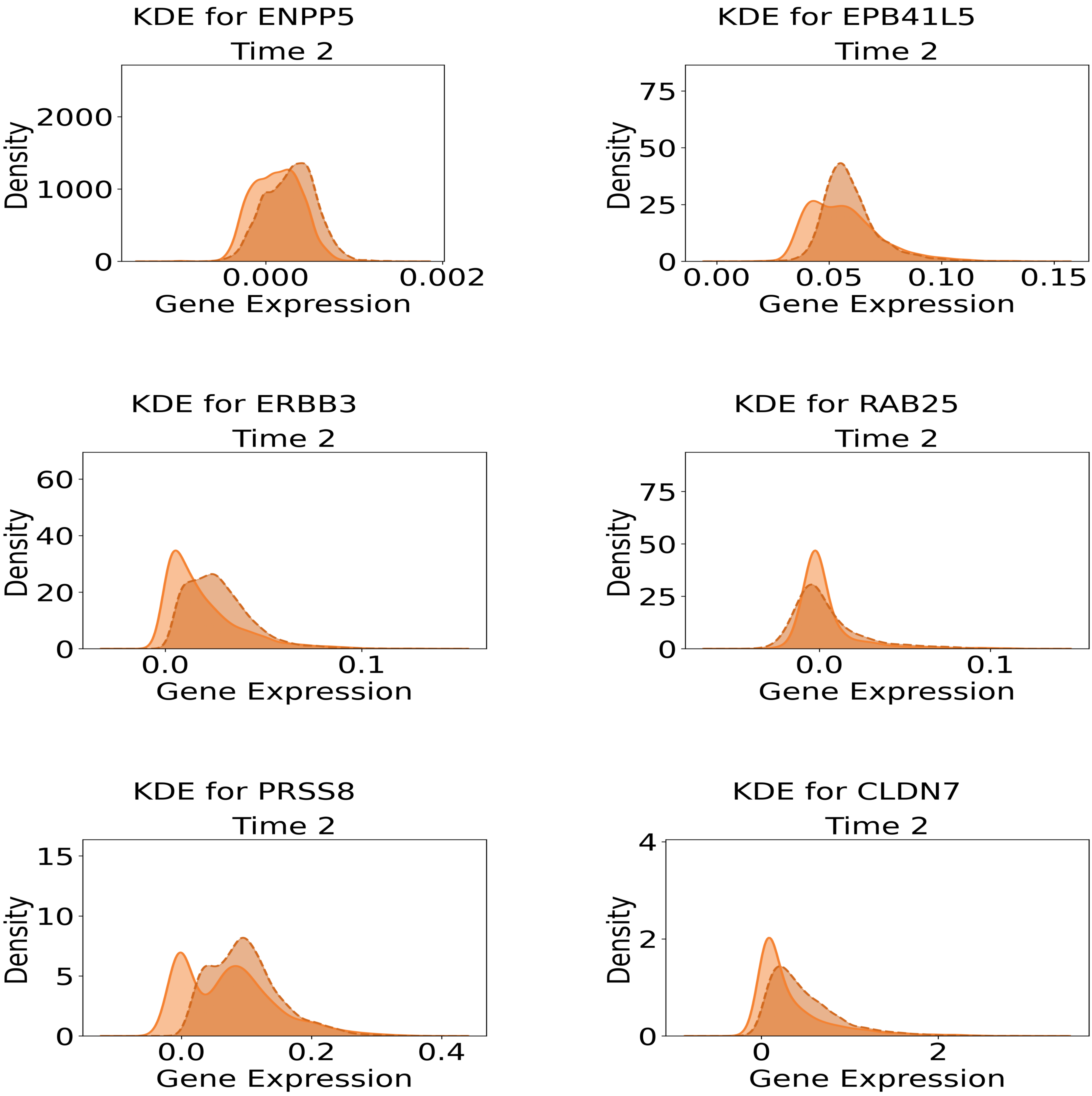

KDE for ENPP5
Time 2
Density
2000
1000
0
0.000
0.002
Gene Expression
KDE for EPB41L5
Time 2
Density
75
50
25
0
0.00
0.05
0.10
0.15
Gene Expression
KDE for ERBB3
Time 2
Density
60
40
20
0
0.0
0.1
Gene Expression
KDE for RAB25
Time 2
Density
75
50
25
0
0.0
0.1
Gene Expression
KDE for PRSS8
Time 2
Density
15
10
5
0
0.0
0.2
0.4
Gene Expression
KDE for CLDN7
Time 2
Density
4
2
0
0
2
Gene Expression

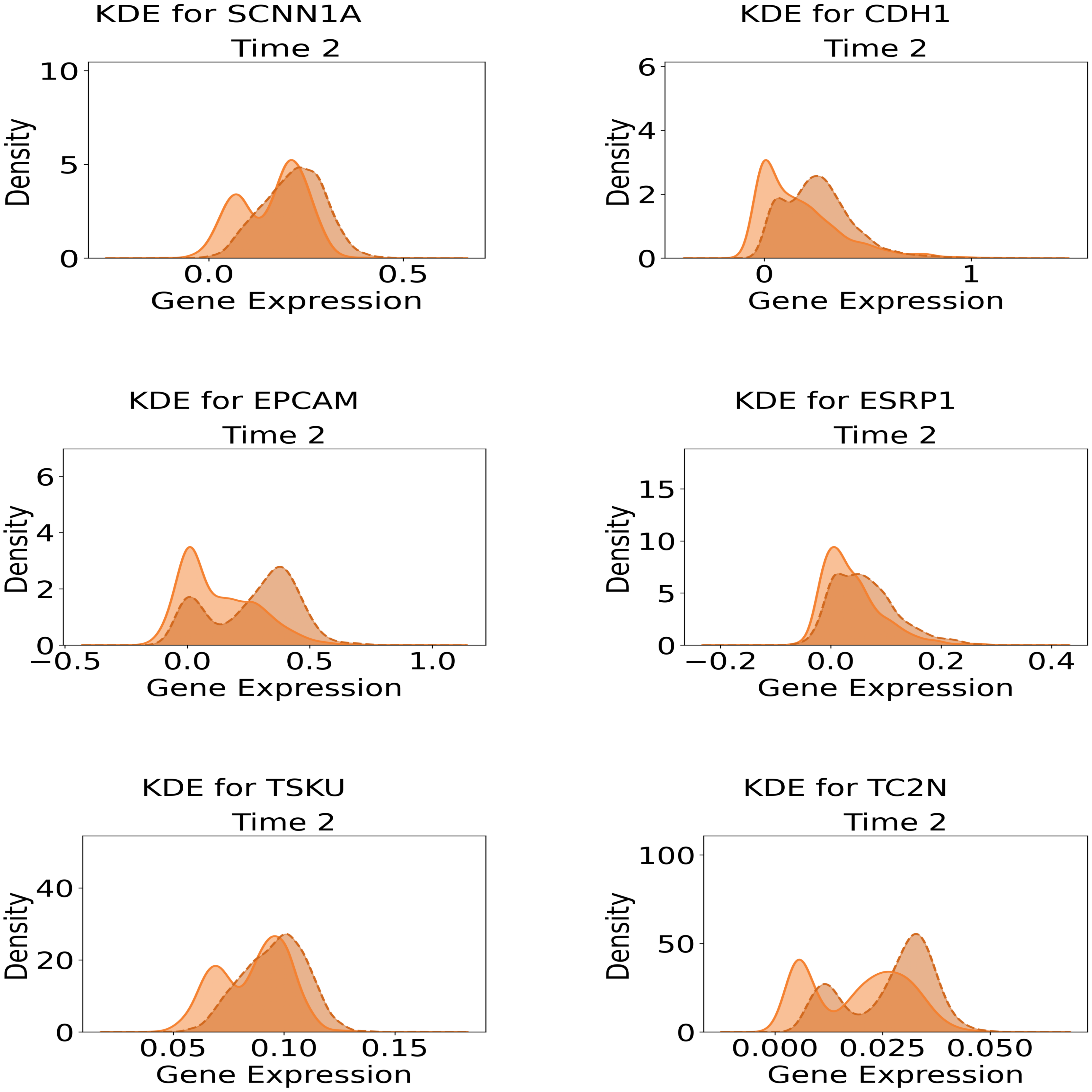

KDE for SCNN1A
Time 2
KDE for CDH1
Time 2
KDE for EPCAM
Time 2
KDE for ESRP1
Time 2
KDE for TSKU
Time 2
KDE for TC2N
Time 2
Density
Gene Expression

Supplementary Figure 8: Comparison of predicted and actual gene expression distributions at time 2. Predicted distributions are shown as dashed lines, while real data distributions are shown as solid lines.
