# Supplementary material for: PROFET Predicts Continuous Gene Expression Dynamics from scRNA-seq Data to Elucidate Heterogeneity of Cancer Treatment Responses
Source: bioRxiv. 2025 Jul 3:2025.06.27.662030. Preprint. [Version 1] doi: 10.1101/2025.06.27.662030 (PMC12236938; doi:10.1101/2025.06.27.662030)
Supplement: Supplement 9 [file media-10.pdf]

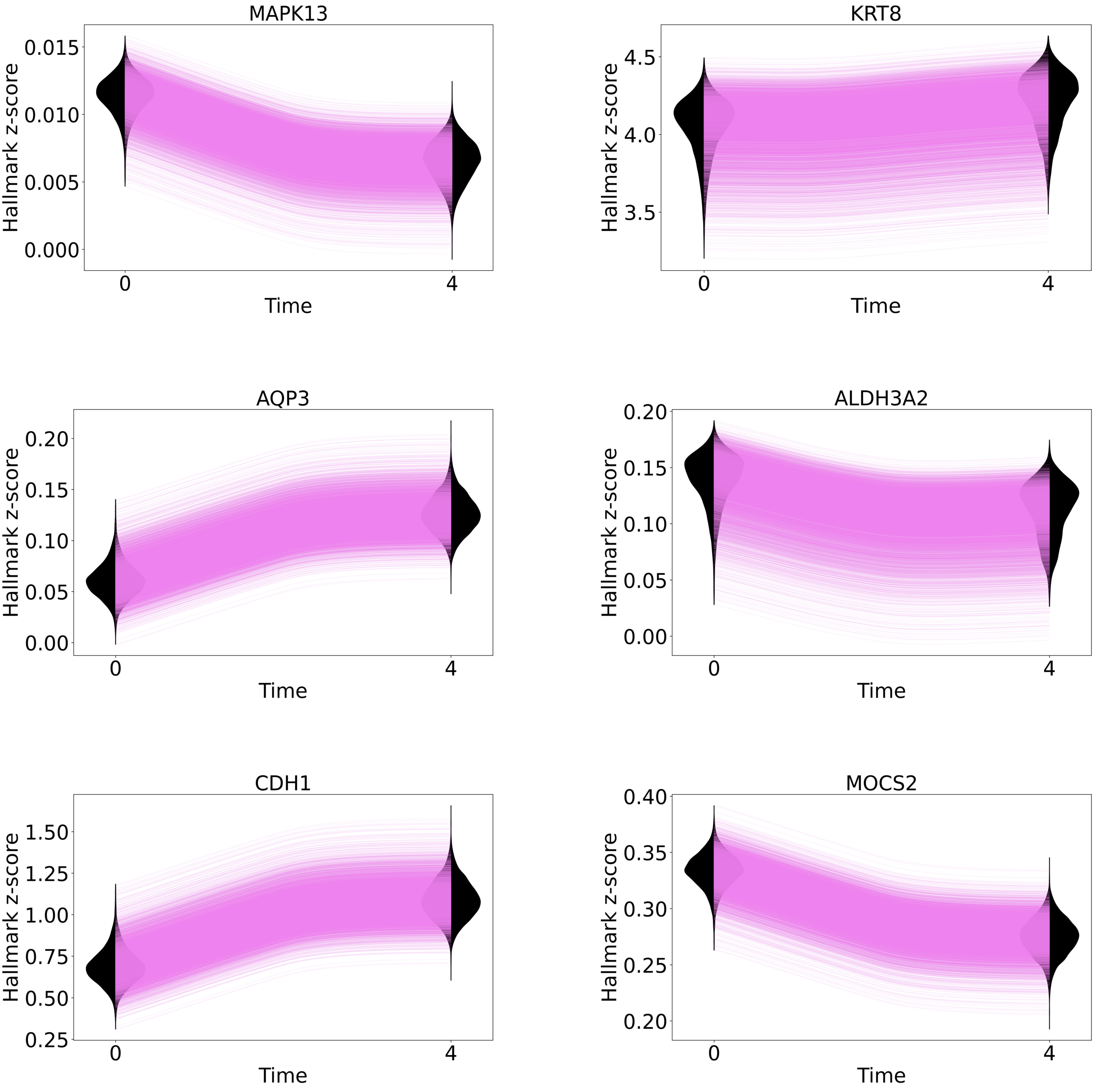
MAPK13
Hallmark z-score
0.015
0.010
0.005
0.000
0
4
Time
KRT8
Hallmark z-score
4.5
4.0
3.5
0
4
Time
AQP3
Hallmark z-score
0.20
0.15
0.10
0.05
0.00
0
4
Time
ALDH3A2
Hallmark z-score
0.20
0.15
0.10
0.05
0.00
0
4
Time
CDH1
Hallmark z-score
1.50
1.25
1.00
0.75
0.50
0.25
0
4
Time
MOCS2
Hallmark z-score
0.40
0.35
0.30
0.25
0.20
0
4
Time

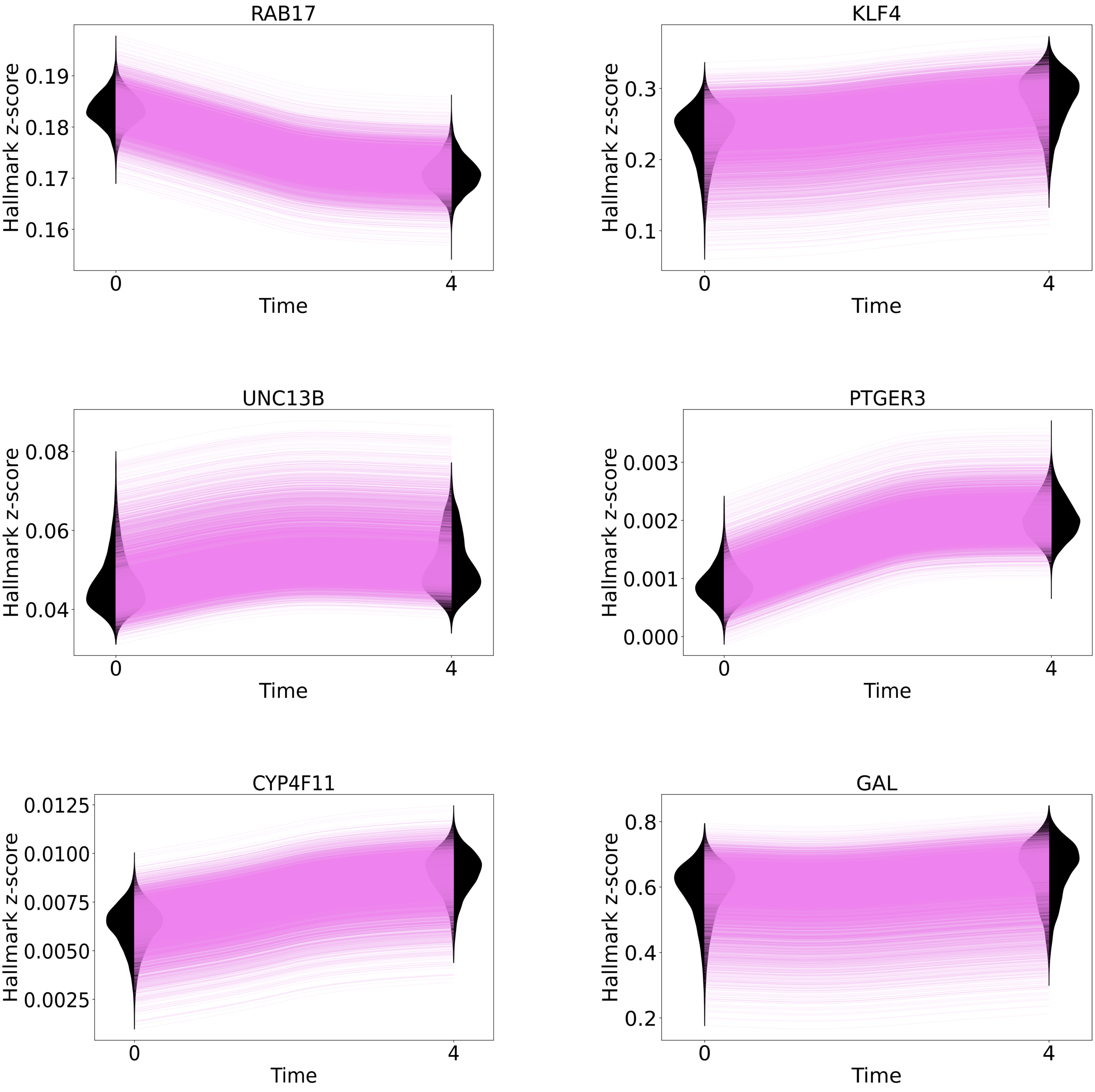
RAB17
Hallmark z-score
0.19
0.18
0.17
0.16
0
4
Time
KLF4
Hallmark z-score
0.3
0.2
0.1
0
4
Time
UNC13B
Hallmark z-score
0.08
0.06
0.04
0
4
Time
PTGER3
Hallmark z-score
0.003
0.002
0.001
0.000
0
4
Time
CYP4F11
Hallmark z-score
0.0125
0.0100
0.0075
0.0050
0.0025
0
4
Time
GAL
Hallmark z-score
0.8
0.6
0.4
0.2
0
4
Time

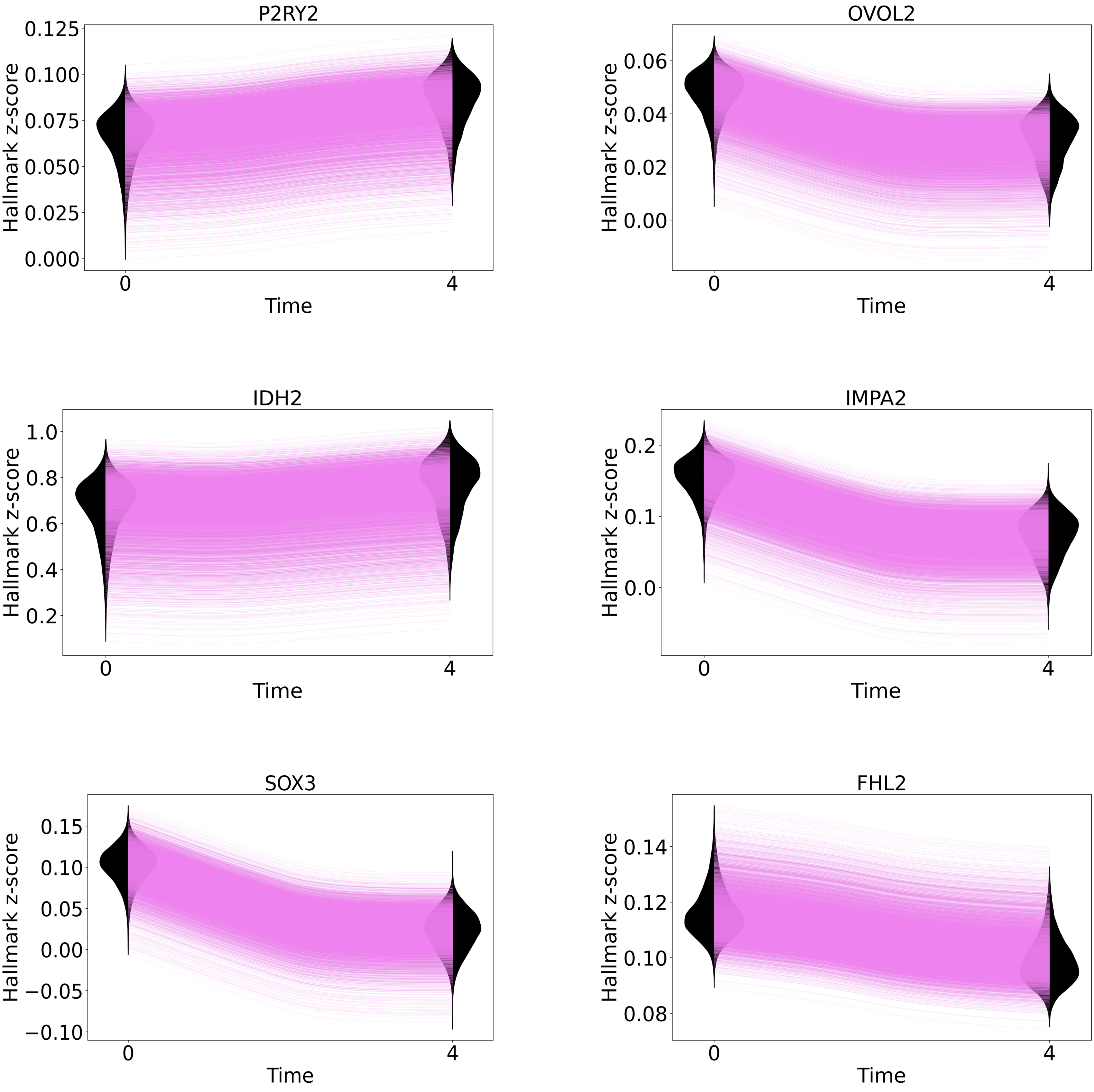
P2RY2
Hallmark z-score
0.000
0.025
0.050
0.075
0.100
0.125
0
4
Time
OVOL2
Hallmark z-score
0.00
0.02
0.04
0.06
0
4
Time
IDH2
Hallmark z-score
0.2
0.4
0.6
0.8
1.0
0
4
Time
IMPA2
Hallmark z-score
0.0
0.1
0.2
0
4
Time
SOX3
Hallmark z-score
−0.10
−0.05
0.00
0.05
0.10
0.15
0
4
Time
FHL2
Hallmark z-score
0.08
0.10
0.12
0.14
0
4
Time

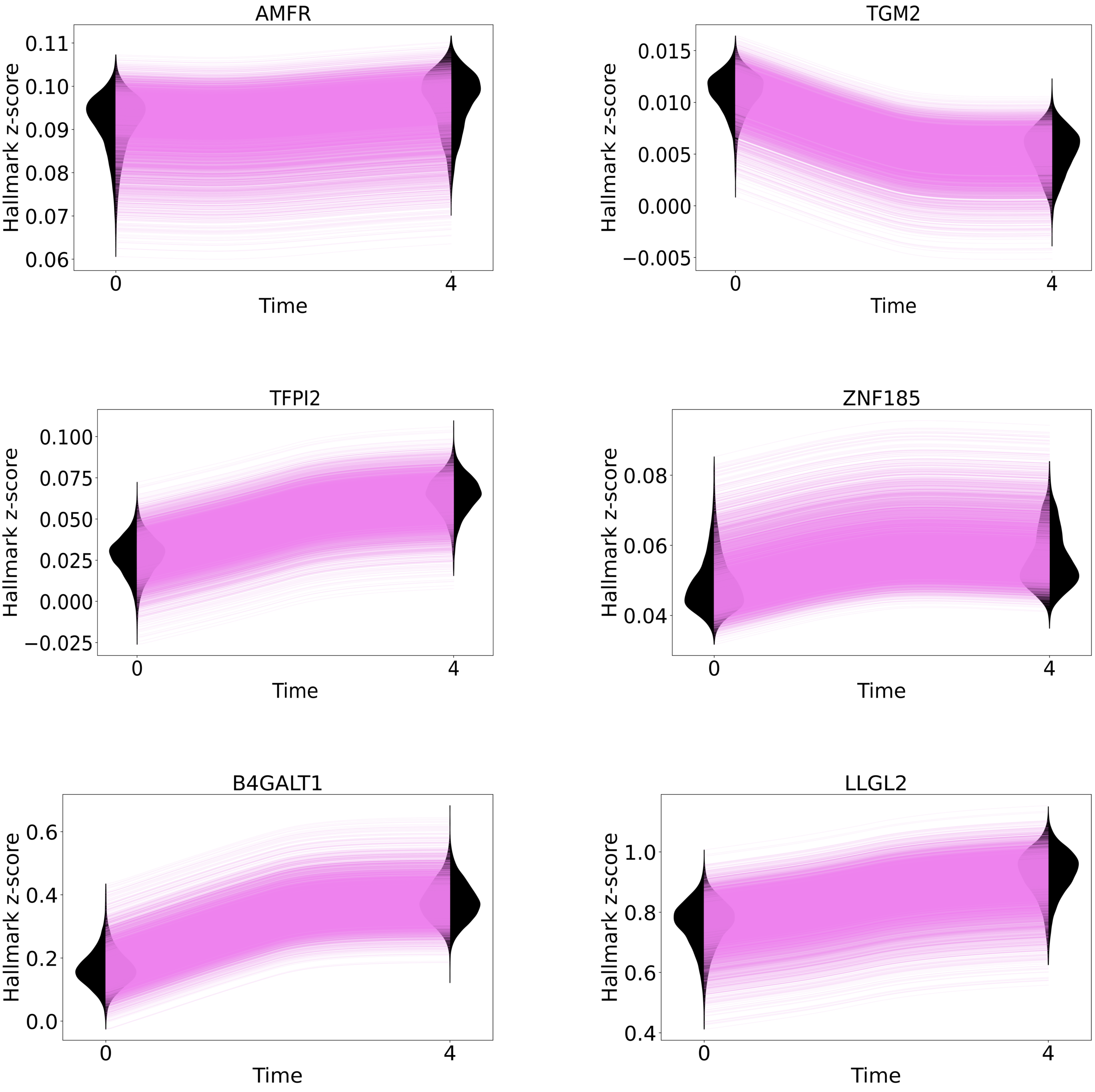
AMFR
TGM2
TFPI2
ZNF185
B4GALT1
LLGL2
Hallmark z-score
Time
0
4
0.11
0.10
0.09
0.08
0.07
0.06
0.015
0.010
0.005
0.000
−0.005
0.100
0.075
0.050
0.025
0.000
−0.025
0.08
0.06
0.04
0.6
0.4
0.2
0.0
1.0
0.8
0.6
0.4

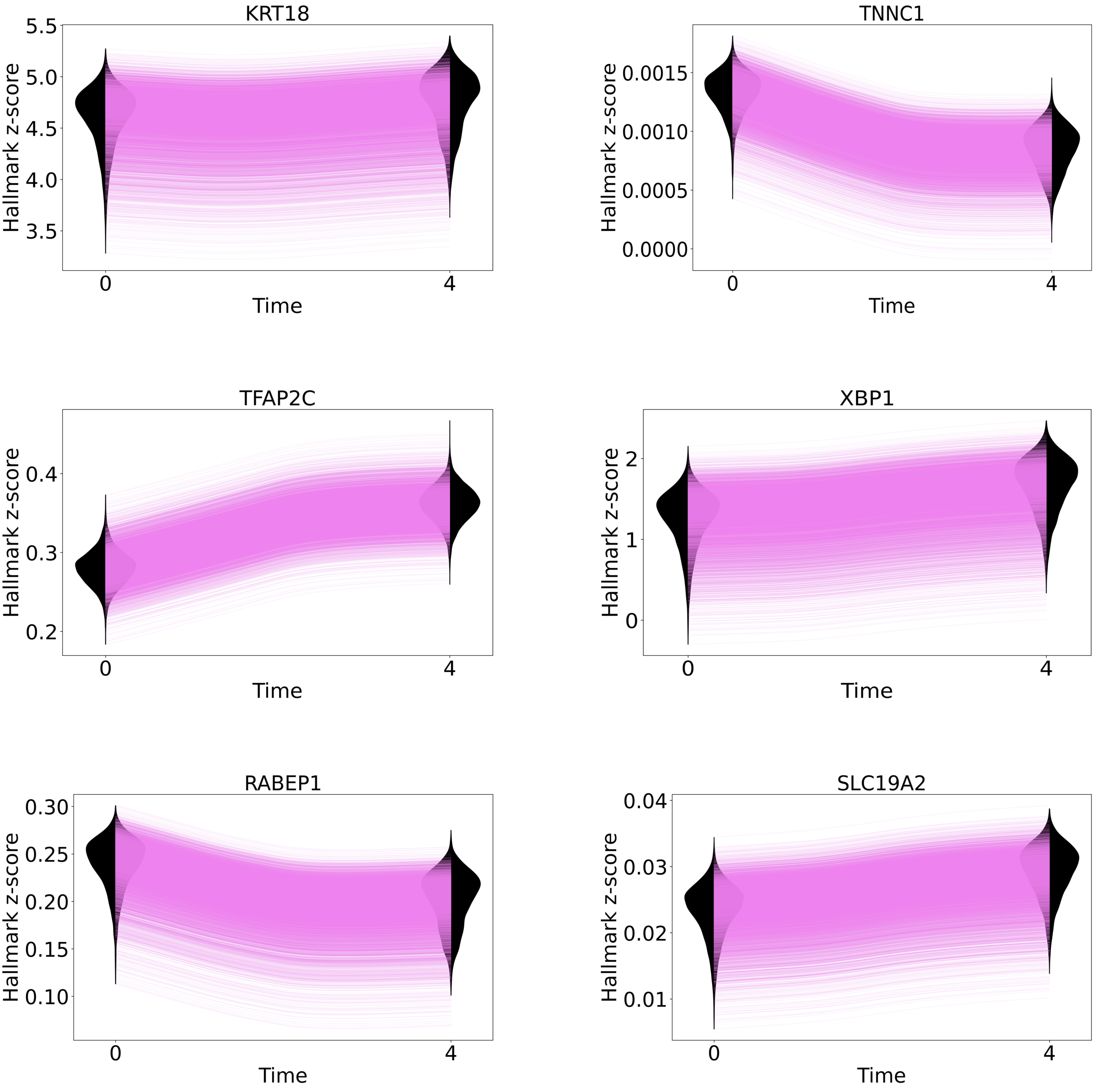
KRT18
Hallmark z-score
5.5
5.0
4.5
4.0
3.5
0
4
Time
TNNC1
Hallmark z-score
0.0015
0.0010
0.0005
0.0000
0
4
Time
TFAP2C
Hallmark z-score
0.4
0.3
0.2
0
4
Time
XBP1
Hallmark z-score
2
1
0
0
4
Time
RABEP1
Hallmark z-score
0.30
0.25
0.20
0.15
0.10
0
4
Time
SLC19A2
Hallmark z-score
0.04
0.03
0.02
0.01
0
4
Time

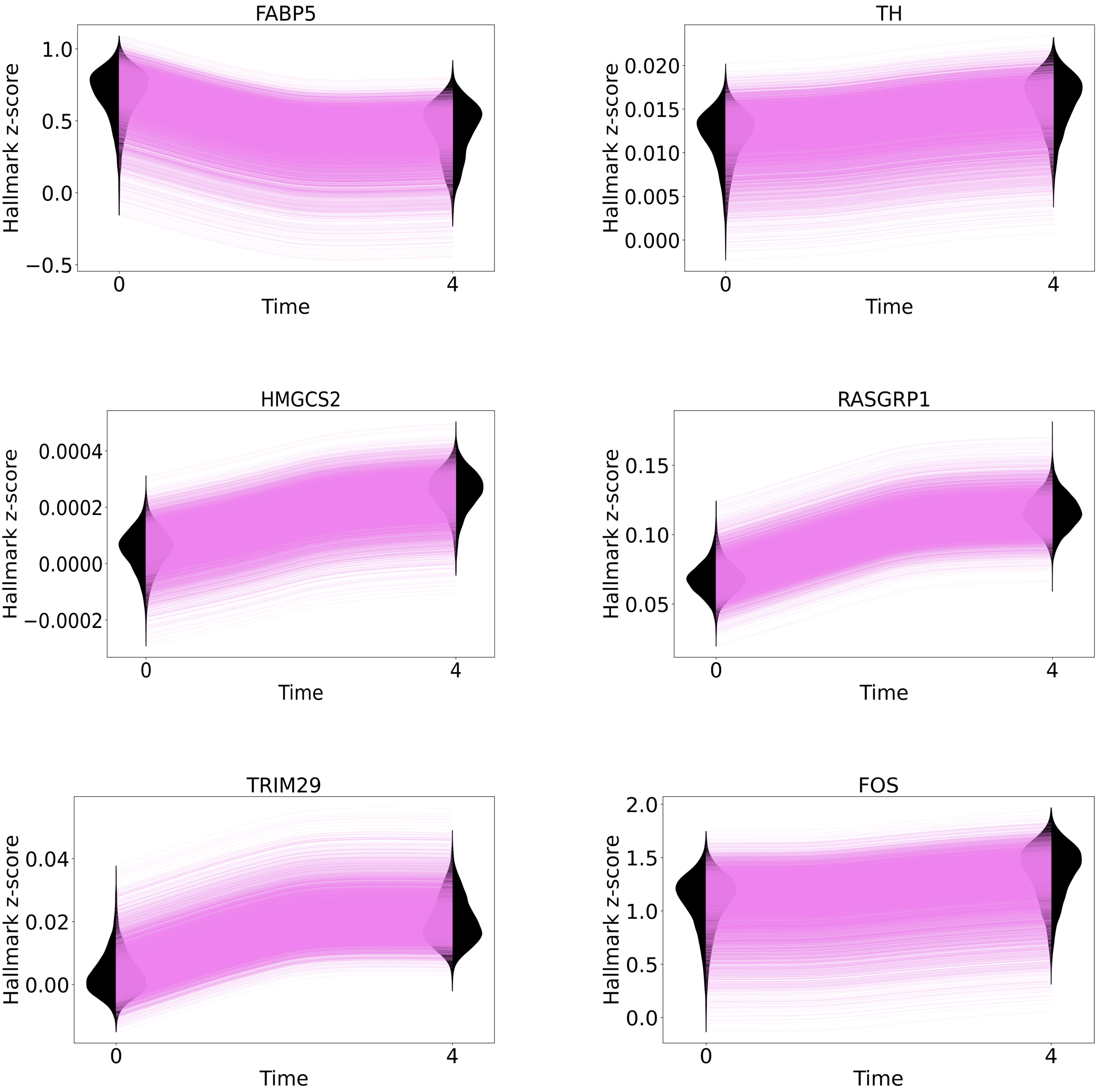

FABP5
TH
HMGCS2
RASGRP1
TRIM29
FOS
Hallmark z-score
Time
0
4
1.0
0.5
0.0
−0.5
0.020
0.015
0.010
0.005
0.000
0.0004
0.0002
0.0000
−0.0002
0.15
0.10
0.05
0.04
0.02
0.00
2.0
1.5
1.0
0.5
0.0

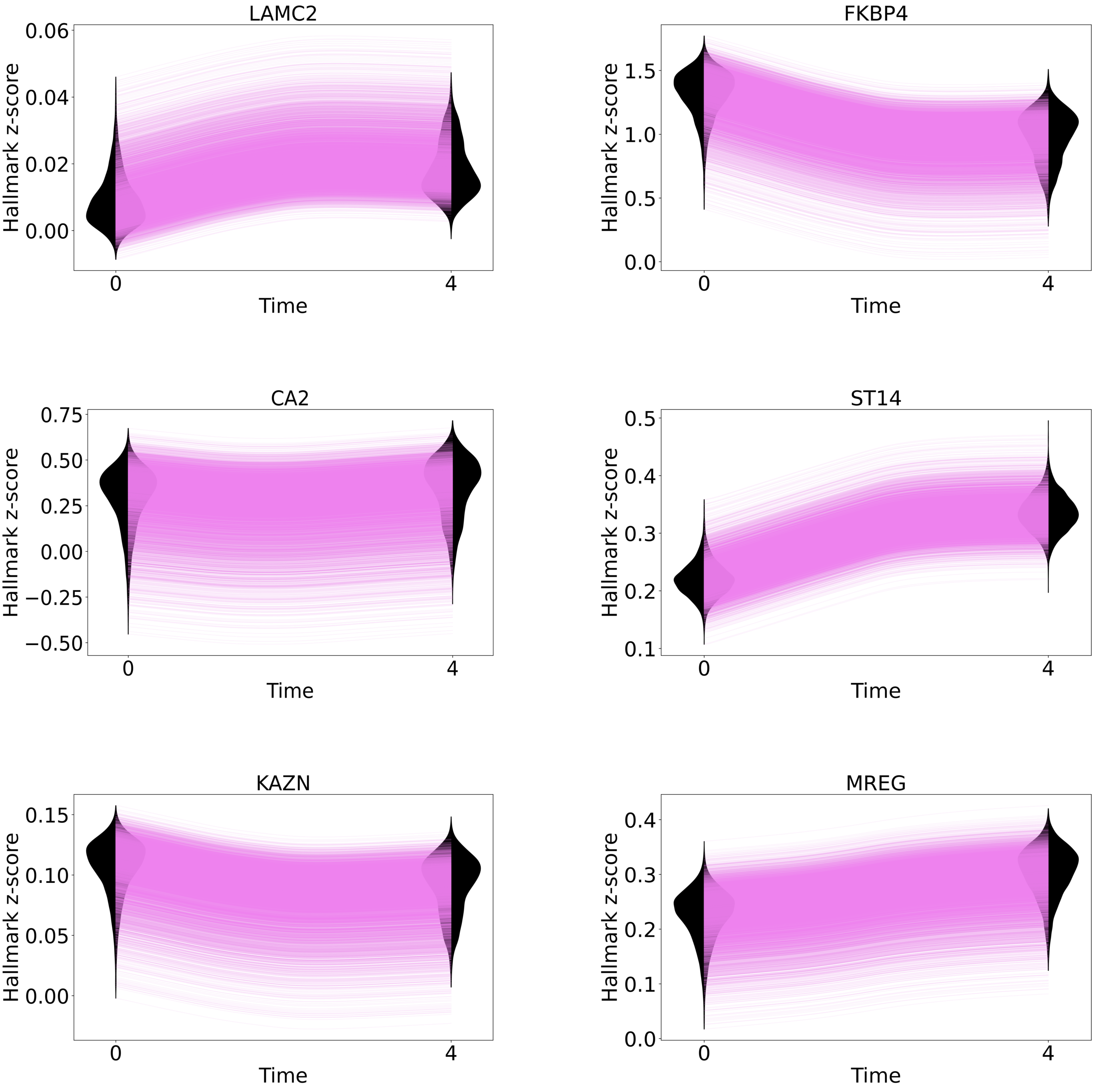

LAMC2
FKBP4
CA2
ST14
KAZN
MREG
Hallmark z-score
Time
0
4

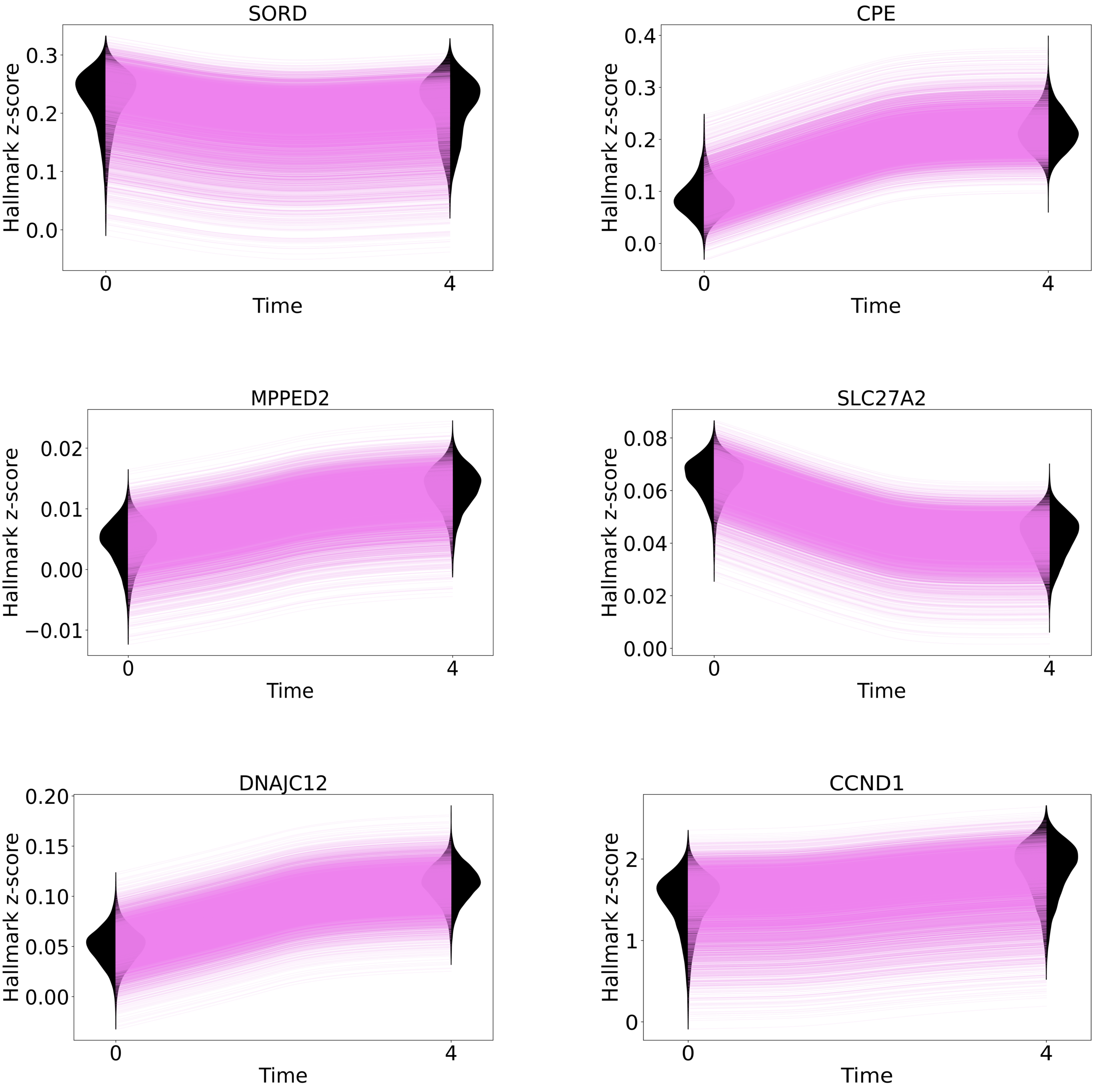

SORD
CPE
MPPED2
SLC27A2
DNAJC12
CCND1
Hallmark z-score
Time
0
4
0.0
0.1
0.2
0.3
0.4
0.00
0.01
0.02
−0.01
0.02
0.04
0.06
0.08
0.05
0.10
0.15
0.20
1
2

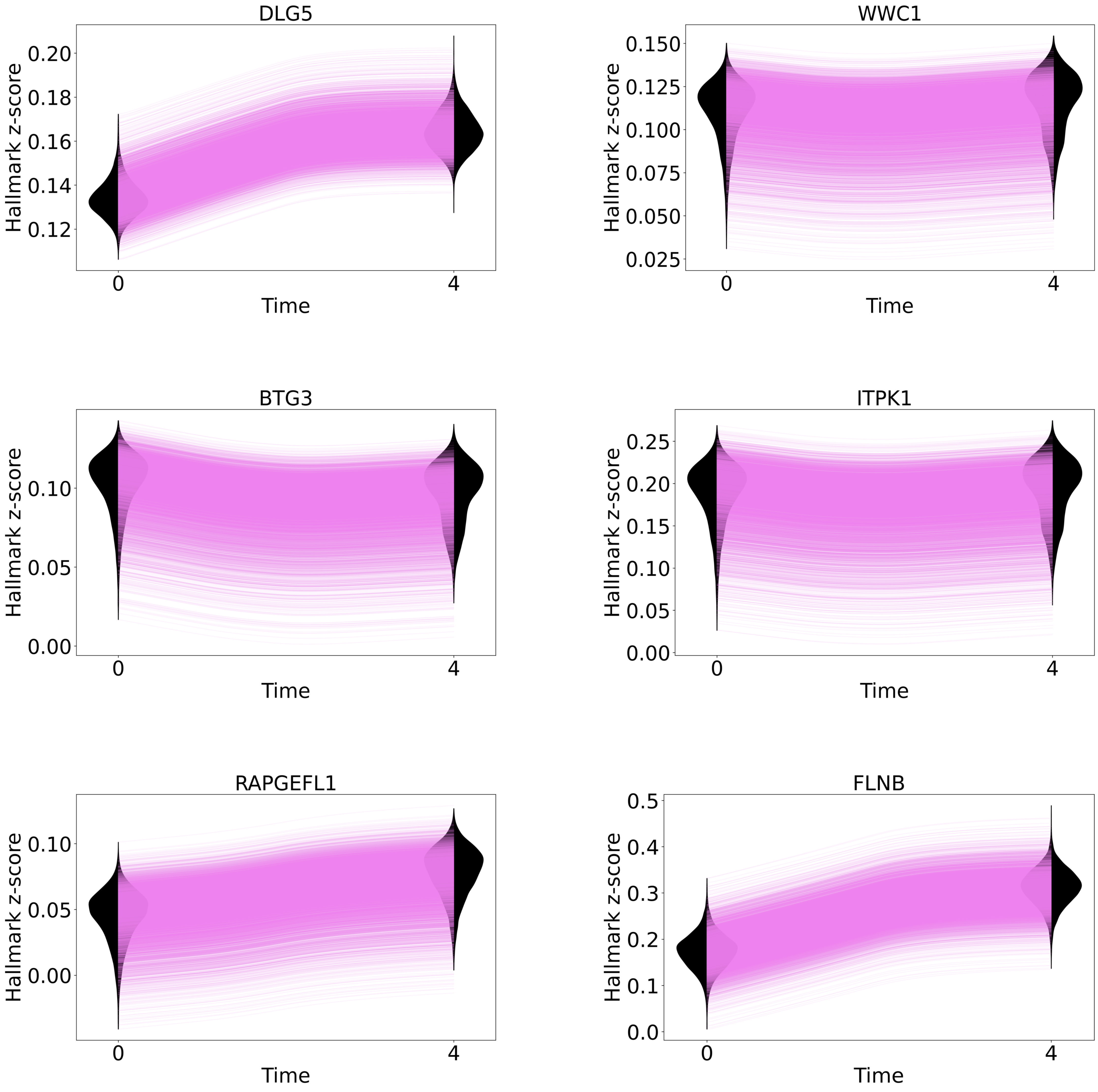

DLG5
Hallmark z-score
0.20
0.18
0.16
0.14
0.12
0
4
Time
WWC1
Hallmark z-score
0.150
0.125
0.100
0.075
0.050
0.025
0
4
Time
BTG3
Hallmark z-score
0.10
0.05
0.00
0
4
Time
ITPK1
Hallmark z-score
0.25
0.20
0.15
0.10
0.05
0.00
0
4
Time
RAPGEFL1
Hallmark z-score
0.10
0.05
0.00
0
4
Time
FLNB
Hallmark z-score
0.5
0.4
0.3
0.2
0.1
0.0
0
4
Time

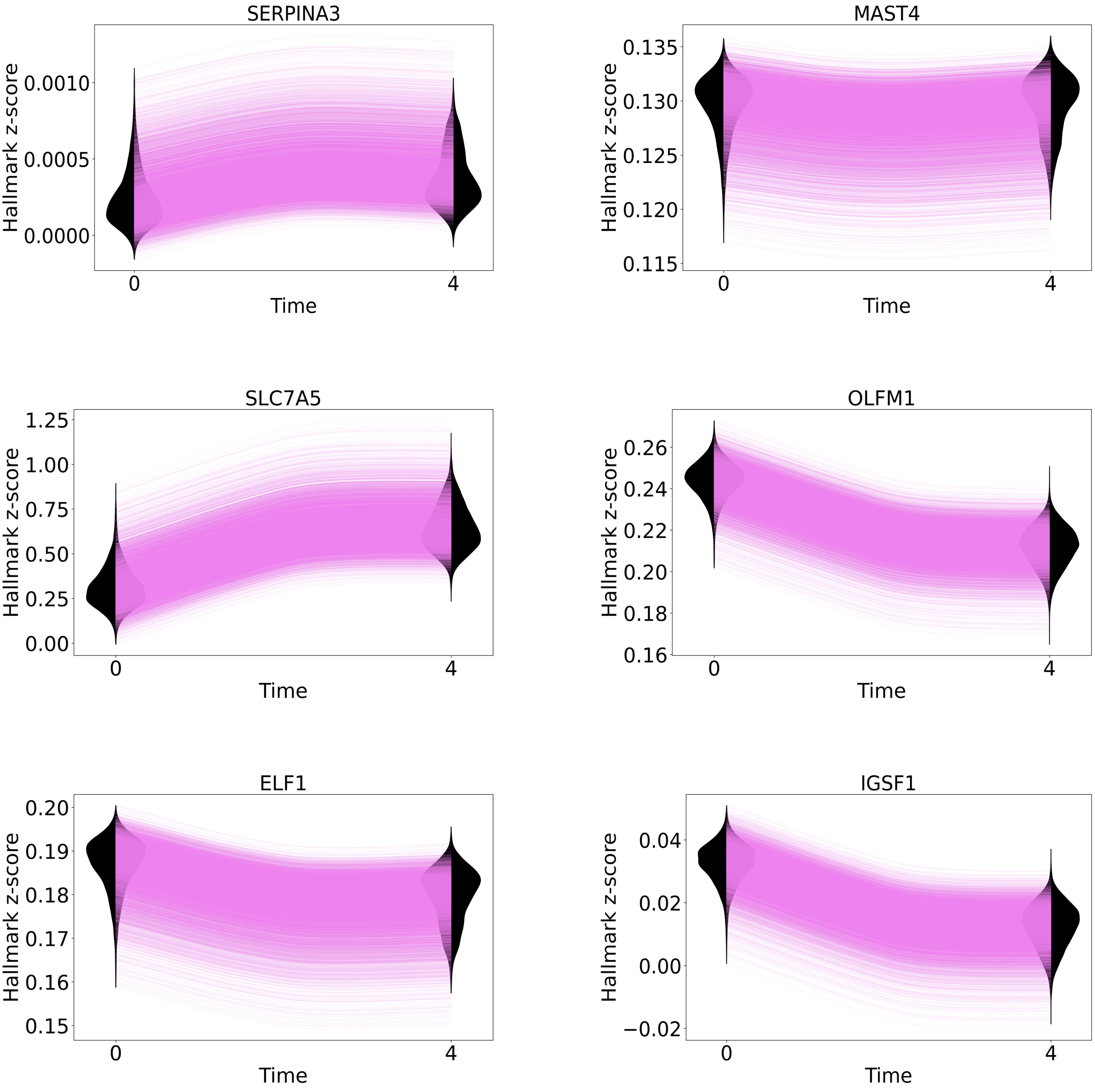

SERPINA3
MAST4
SLC7A5
OLFM1
ELF1
IGSF1
Hallmark z-score
Time
0
4
0.0000
0.0005
0.0010
0.115
0.120
0.125
0.130
0.135
0.00
0.25
0.50
0.75
1.00
1.25
0.16
0.18
0.20
0.22
0.24
0.26
0.15
0.16
0.17
0.18
0.19
0.20
−0.02
0.00
0.02
0.04

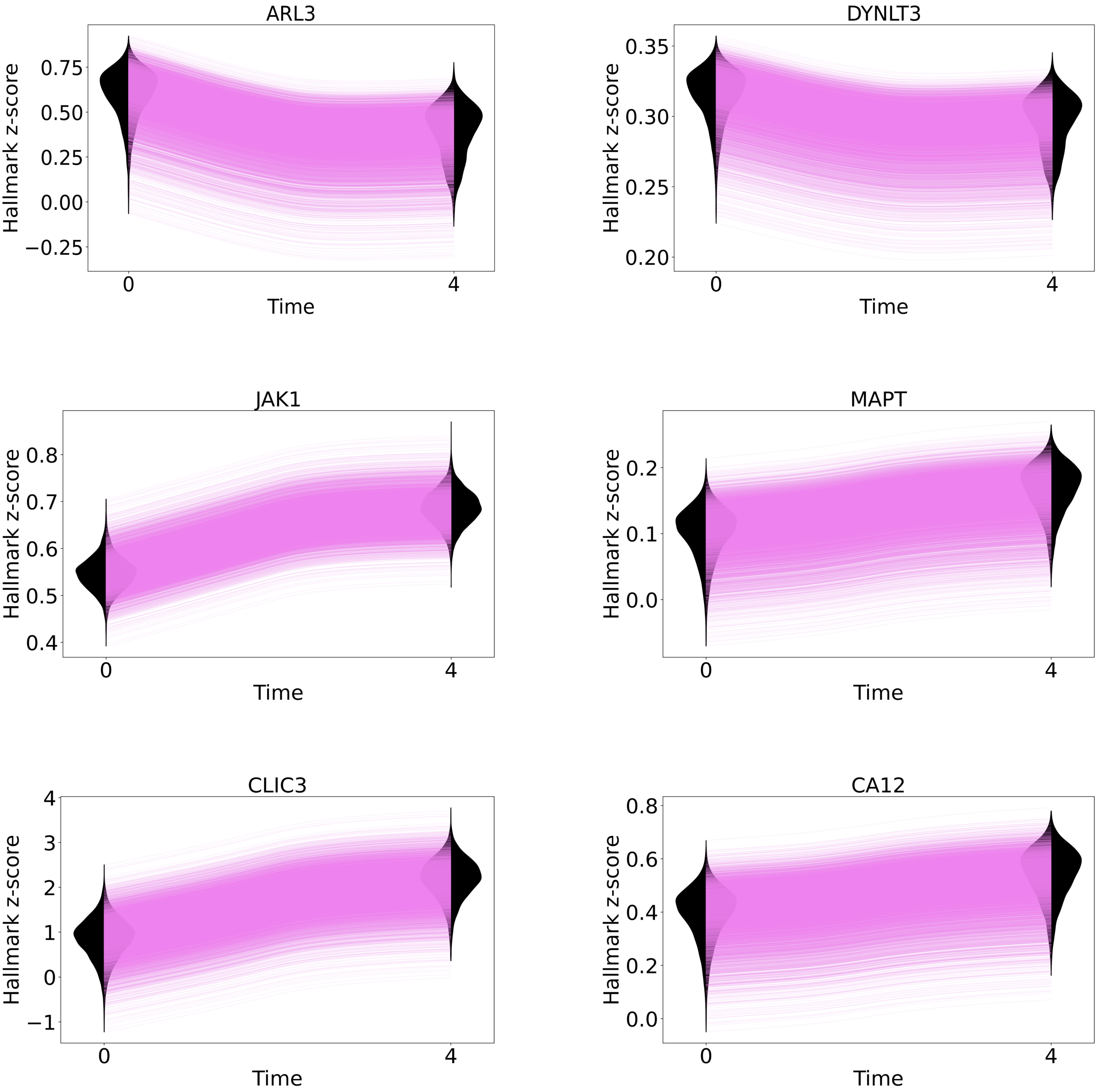
ARL3
DYNLT3
JAK1
MAPT
CLIC3
CA12
Hallmark z-score
Time
0
4

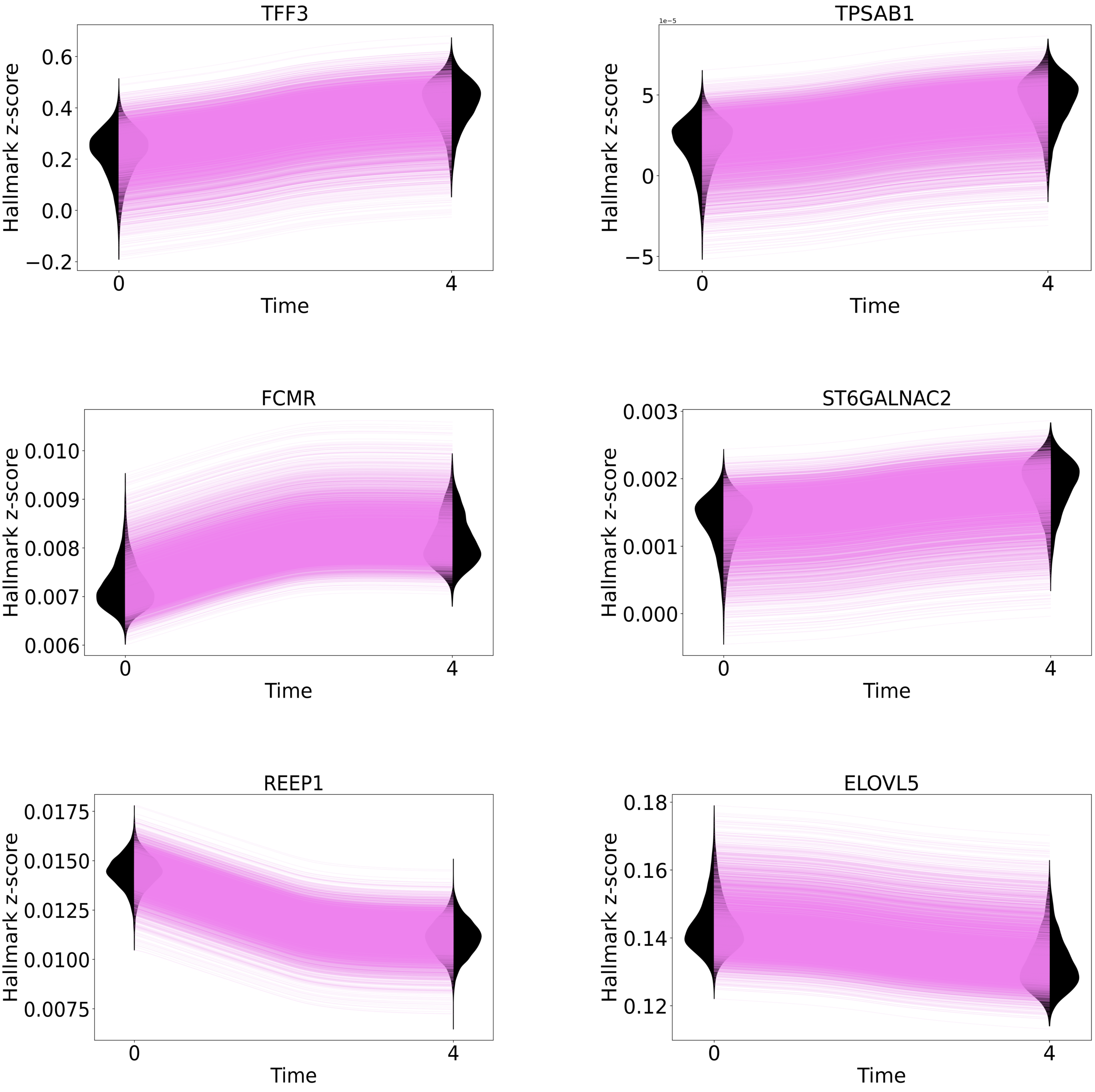

TFF3
Hallmark z-score
0.6
0.4
0.2
0.0
−0.2
0
4
Time
TPSAB1
1e−5
5
0
−5
FCMR
0.010
0.009
0.008
0.007
0.006
ST6GALNAC2
0.003
0.002
0.001
0.000
REEP1
0.0175
0.0150
0.0125
0.0100
0.0075
ELOVL5
0.18
0.16
0.14
0.12

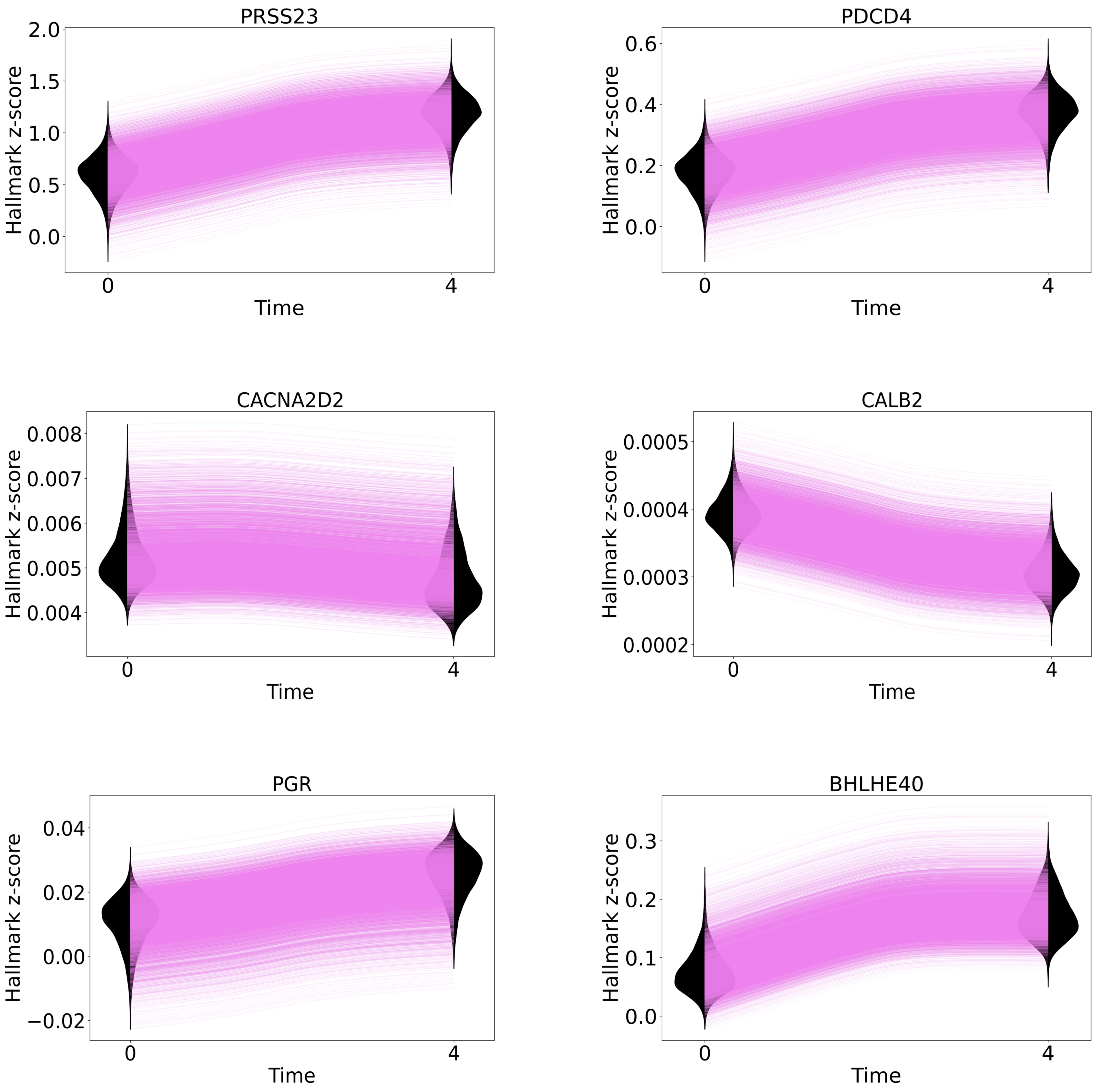
PRSS23
Hallmark z-score
2.0
1.5
1.0
0.5
0.0
0
4
Time
PDCD4
Hallmark z-score
0.6
0.4
0.2
0.0
0
4
Time
CACNA2D2
Hallmark z-score
0.008
0.007
0.006
0.005
0.004
0
4
Time
CALB2
Hallmark z-score
0.0005
0.0004
0.0003
0.0002
0
4
Time
PGR
Hallmark z-score
0.04
0.02
0.00
−0.02
0
4
Time
BHLHE40
Hallmark z-score
0.3
0.2
0.1
0.0
0
4
Time

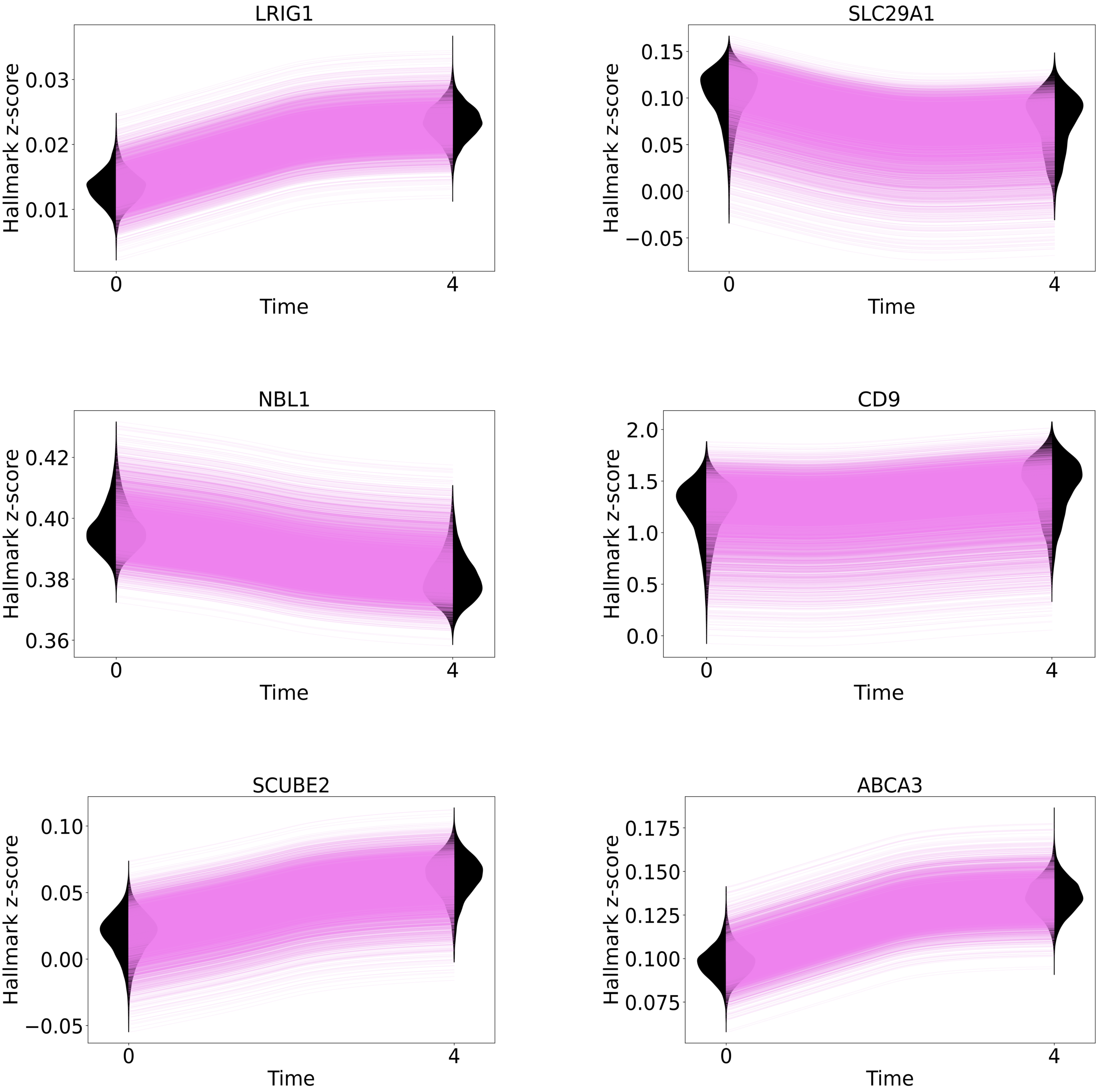

LRIG1
SLC29A1
NBL1
CD9
SCUBE2
ABCA3
Hallmark z-score
Time
0
4
0.03
0.02
0.01
0.15
0.10
0.05
0.00
−0.05
0.42
0.40
0.38
0.36
2.0
1.5
1.0
0.5
0.0
0.175
0.150
0.125
0.100
0.075

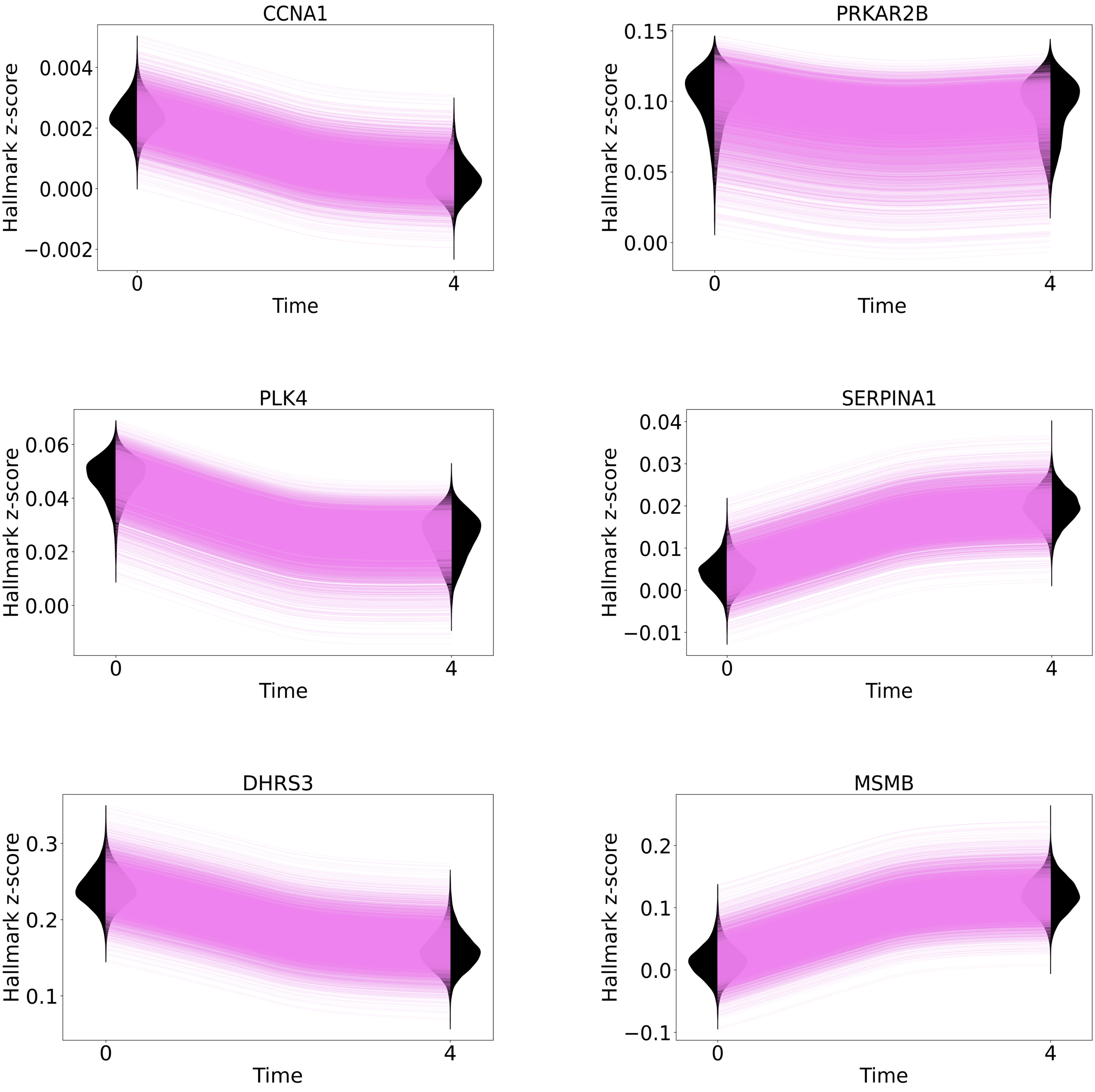

CCNA1
PRKAR2B
PLK4
SERPINA1
DHRS3
MSMB
Hallmark z-score
Time
0
4
0.004
0.002
0.000
−0.002
0.15
0.10
0.05
0.00
0.06
0.04
0.02
0.04
0.03
0.01
−0.01
0.3
0.2
0.1
0.0
−0.1

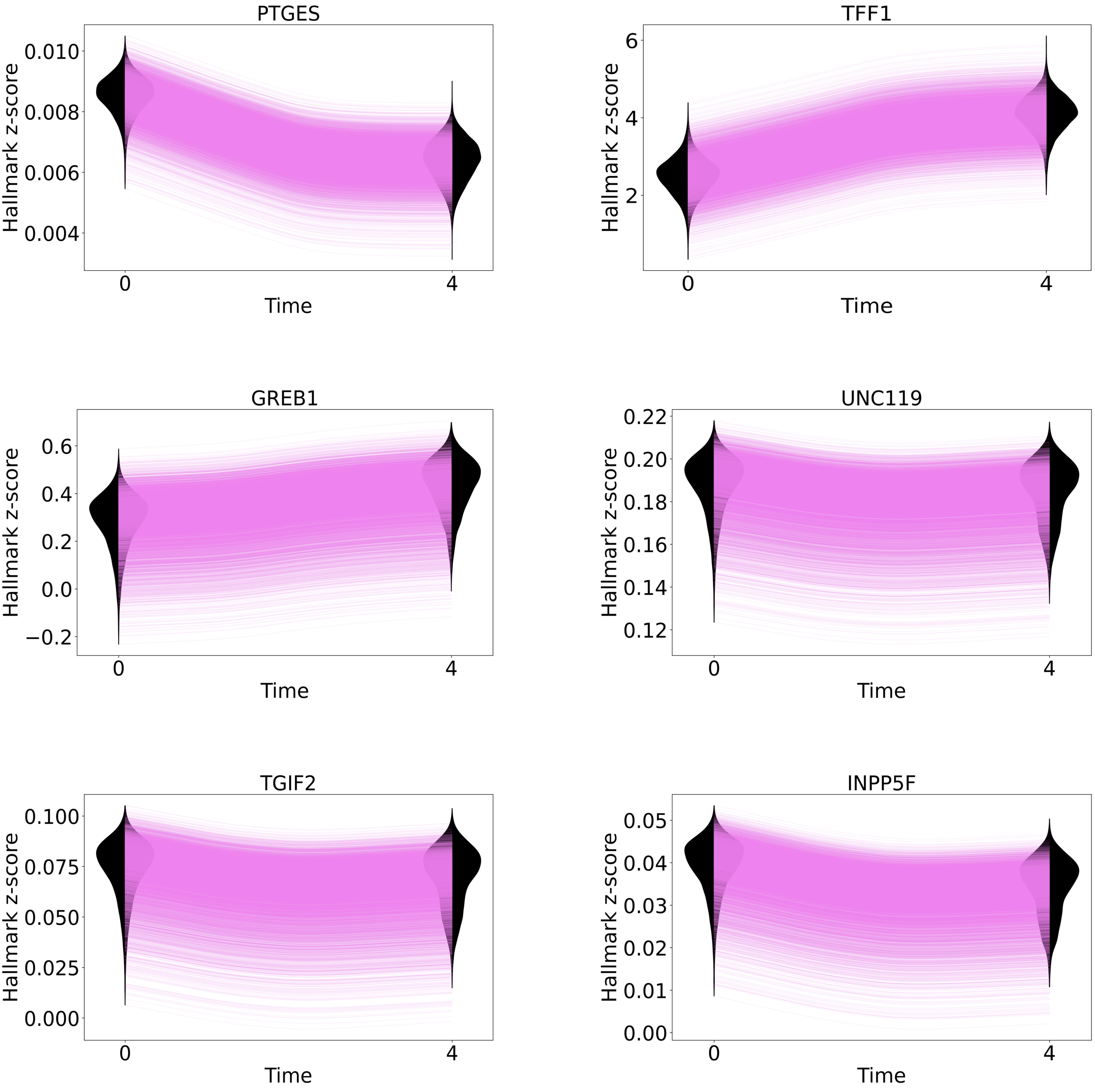
PTGES
TFF1
GREB1
UNC119
TGIF2
INPP5F
Hallmark z-score
Time
0
4
0.010
0.008
0.006
0.004
6
4
2
0.6
0.4
0.2
0.0
−0.2
0.22
0.20
0.18
0.16
0.14
0.12
0.100
0.075
0.050
0.025
0.000
0.05
0.04
0.03
0.02
0.01
0.00

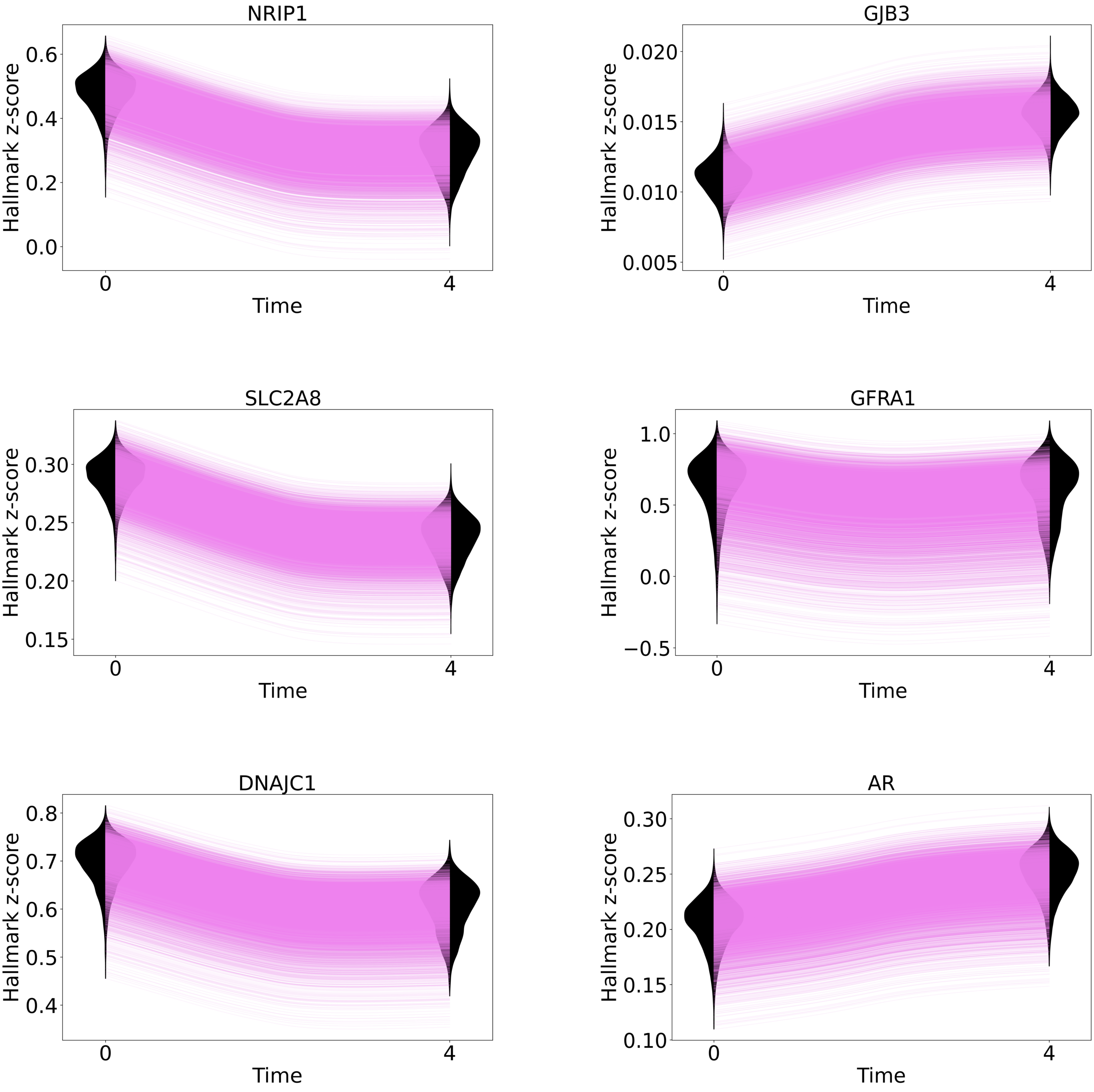
NRIP1
GJB3
SLC2A8
GFRA1
DNAJC1
AR
Hallmark z-score
Time
0
4

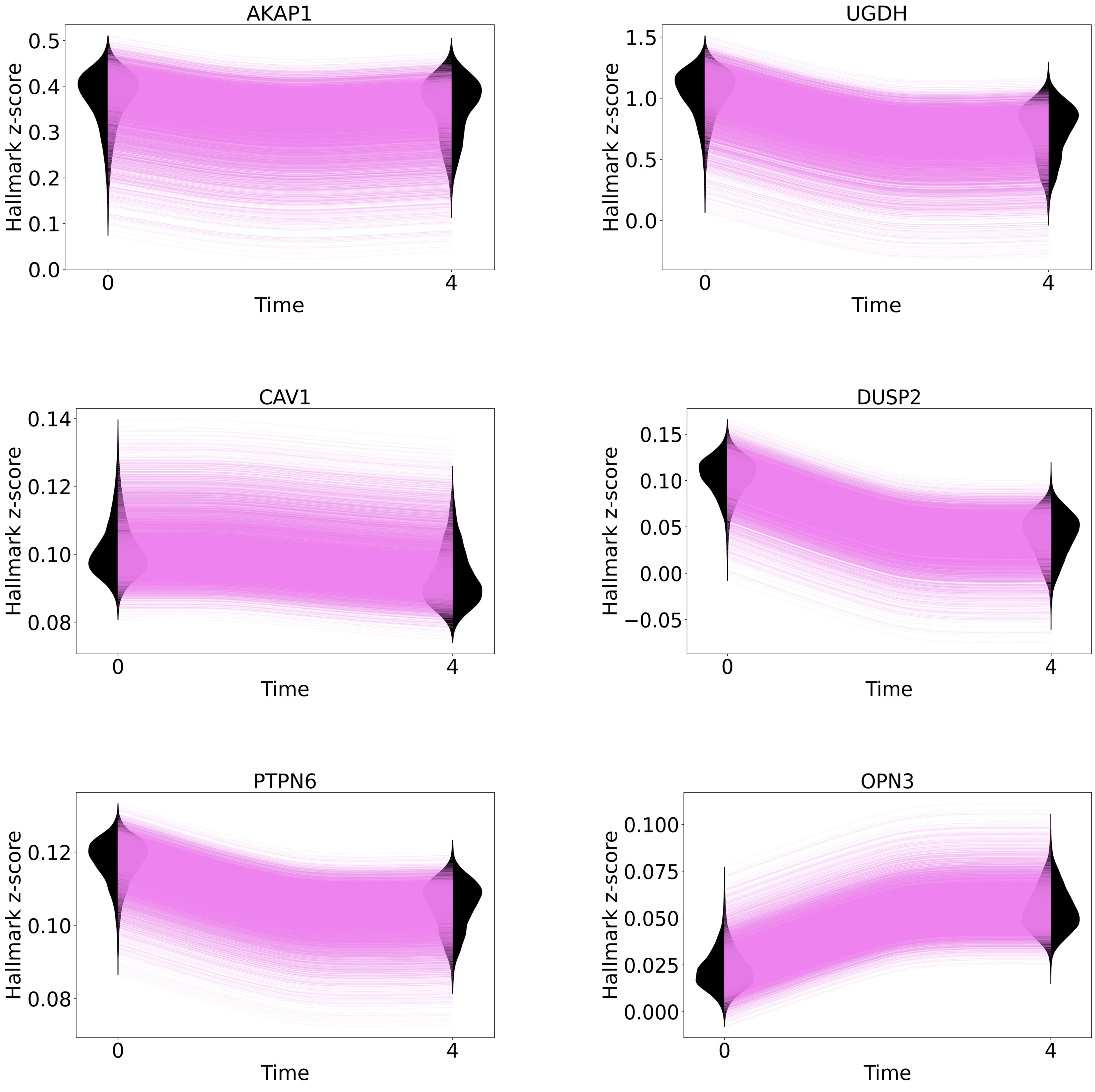
AKAP1
UGDH
CAV1
DUSP2
PTPN6
OPN3
Hallmark z-score
Time
0
4
0.0
0.1
0.2
0.3
0.4
0.5
1.5
1.0
0.5
0.0
0.14
0.12
0.10
0.08
0.15
0.10
0.05
0.00
−0.05
0.100
0.075
0.050
0.025
0.000

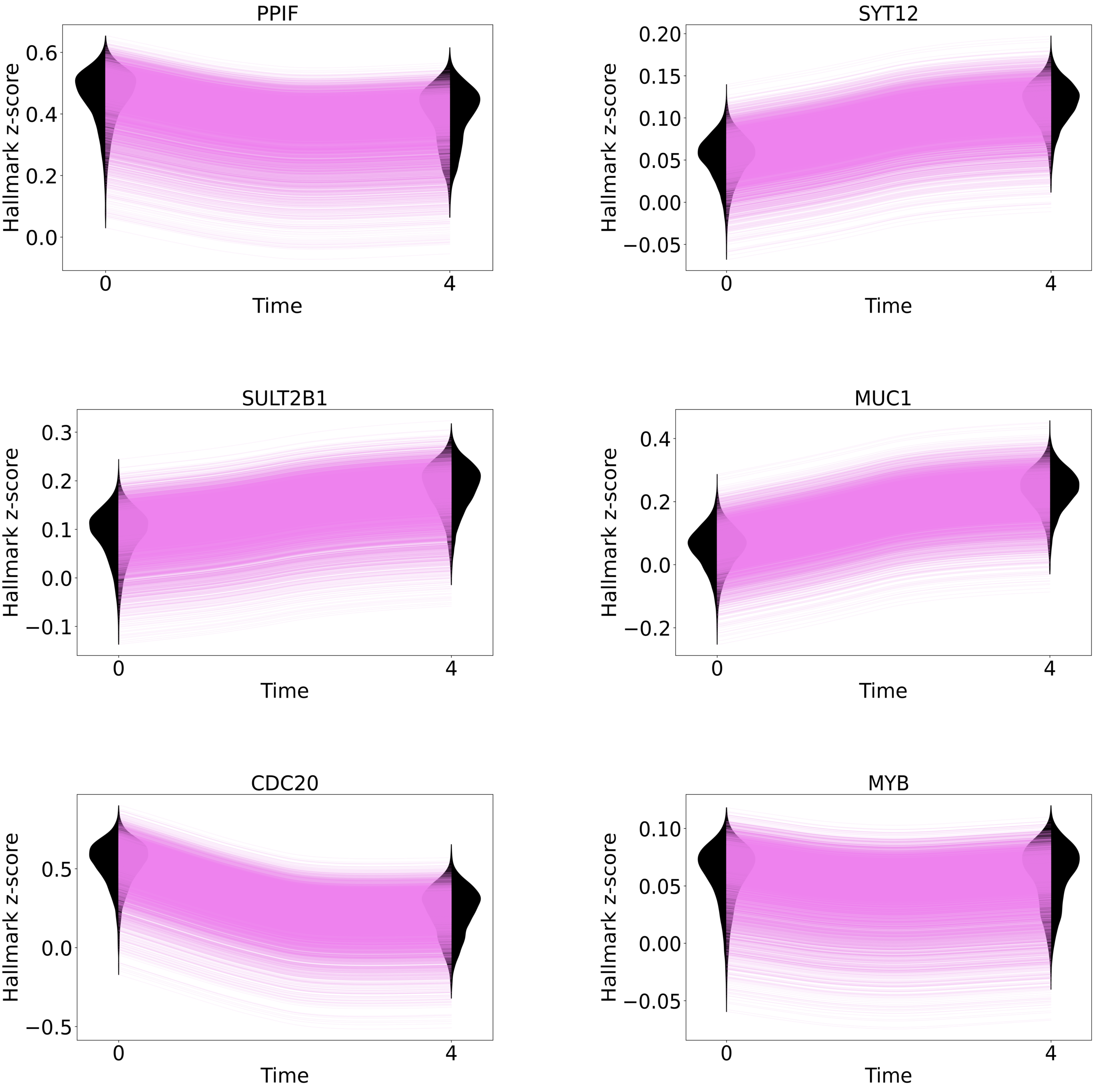

PPIF
SYT12
SULT2B1
MUC1
CDC20
MYB
Hallmark z-score
Time
0
4
0.6
0.4
0.2
0.0
0.20
0.15
0.10
0.05
0.00
−0.05
0.3
0.2
0.1
0.0
−0.1
0.4
0.2
0.0
−0.2
0.5
0.0
−0.5
0.10
0.05
0.00
−0.05

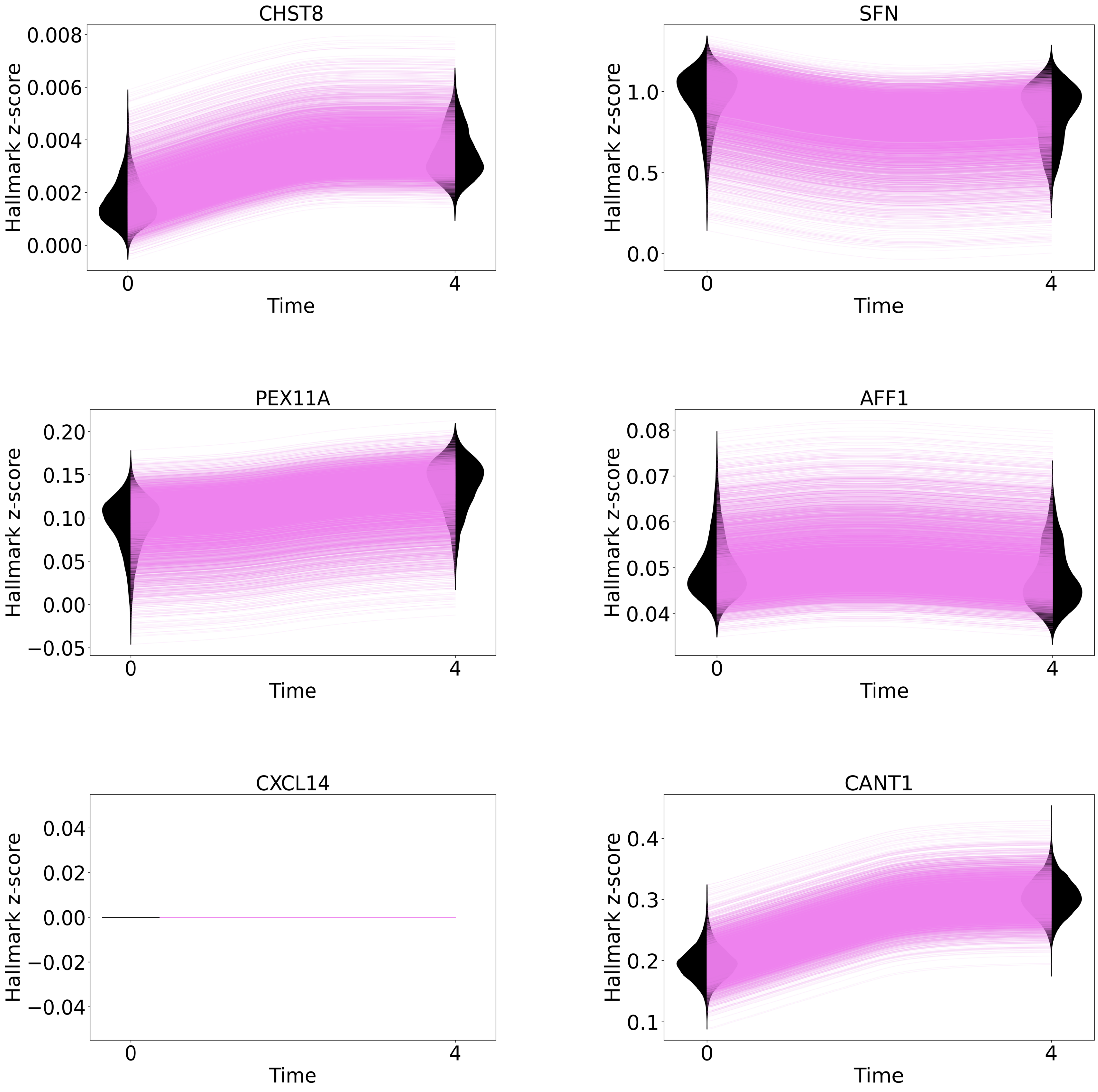
CHST8
0.008
0.006
0.004
0.002
0.000
Hallmark z-score
0
4
Time
SFN
1.0
0.5
0.0
Hallmark z-score
0
4
Time
PEX11A
0.20
0.15
0.10
0.05
0.00
−0.05
Hallmark z-score
0
4
Time
AFF1
0.08
0.07
0.06
0.05
0.04
Hallmark z-score
0
4
Time
CXCL14
0.04
0.02
0.00
−0.02
−0.04
Hallmark z-score
0
4
Time
CANT1
0.4
0.3
0.2
0.1
Hallmark z-score
0
4
Time

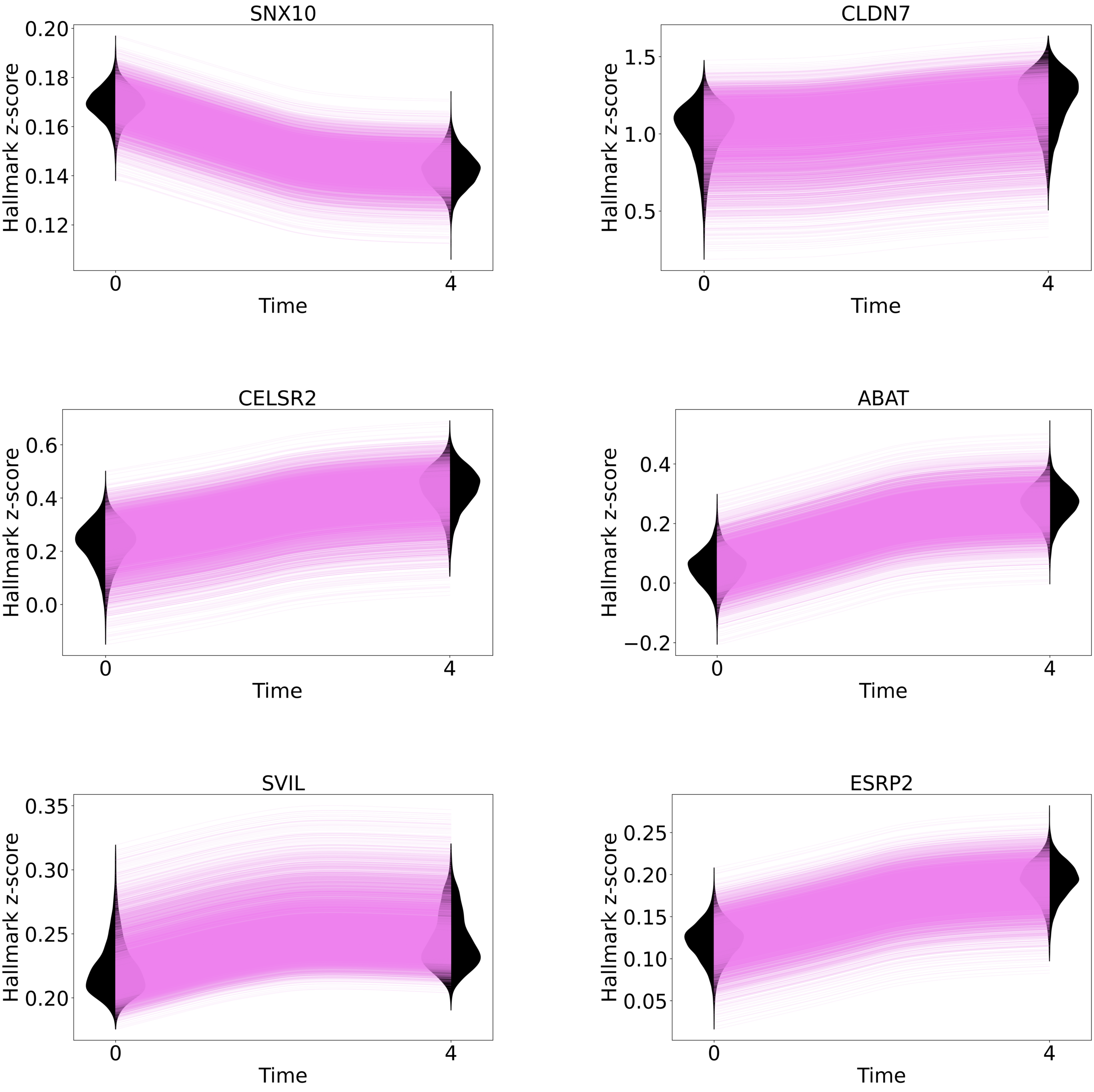

SNX10
CLDN7
CELSR2
ABAT
SVIL
ESRP2
Hallmark z-score
Time
0
4
0.20
0.18
0.16
0.14
0.12
1.5
1.0
0.5
0.6
0.4
0.2
0.0
−0.2
0.35
0.30
0.25
0.20
0.15
0.10
0.05

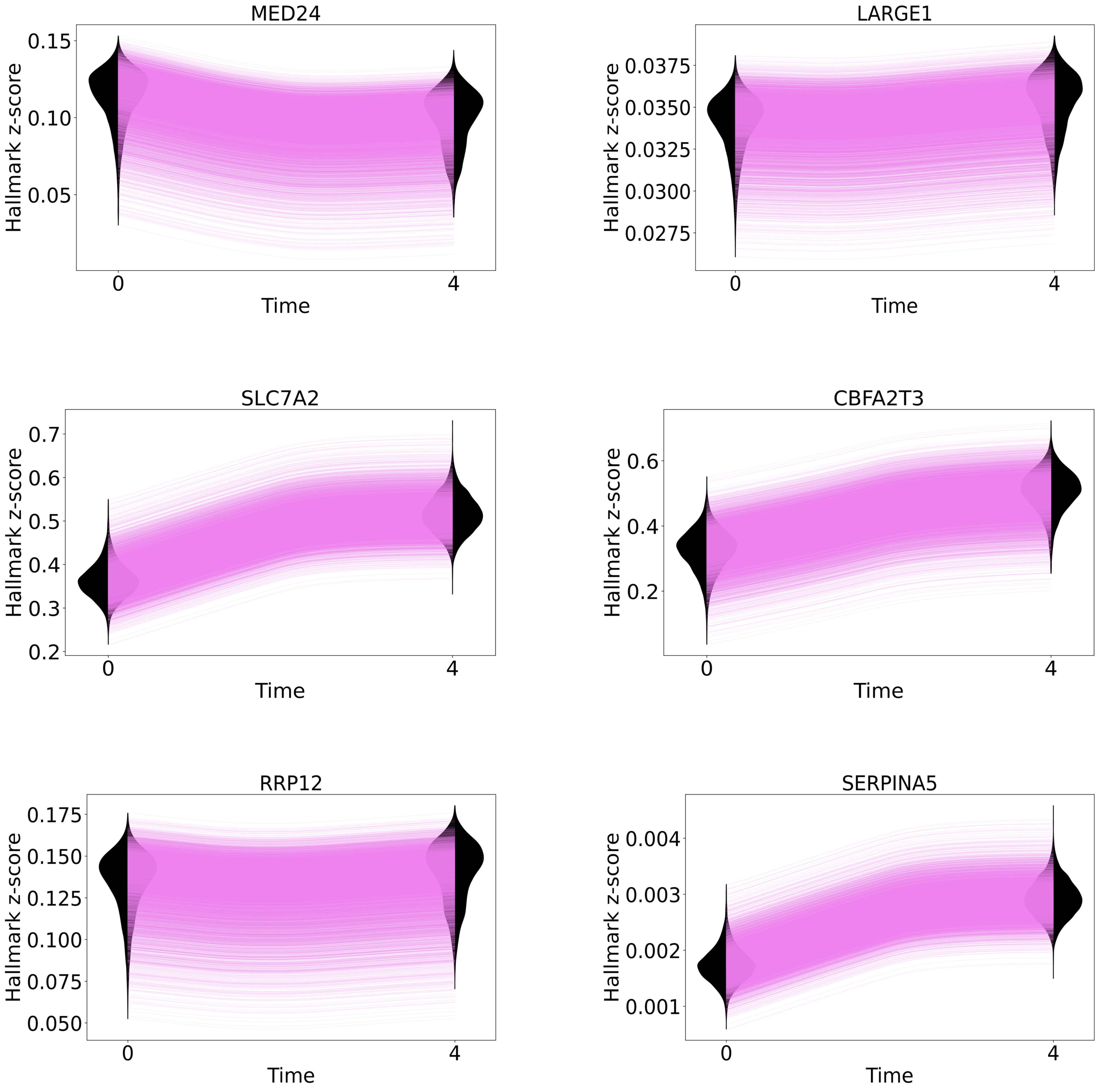
MED24
LARGE1
SLC7A2
CBFA2T3
RRP12
SERPINA5
Hallmark z-score
Time
0
4
0.15
0.10
0.05
0.0375
0.0350
0.0325
0.0300
0.0275
0.7
0.6
0.5
0.4
0.3
0.2
0.175
0.150
0.125
0.100
0.075
0.050
0.004
0.003
0.002
0.001

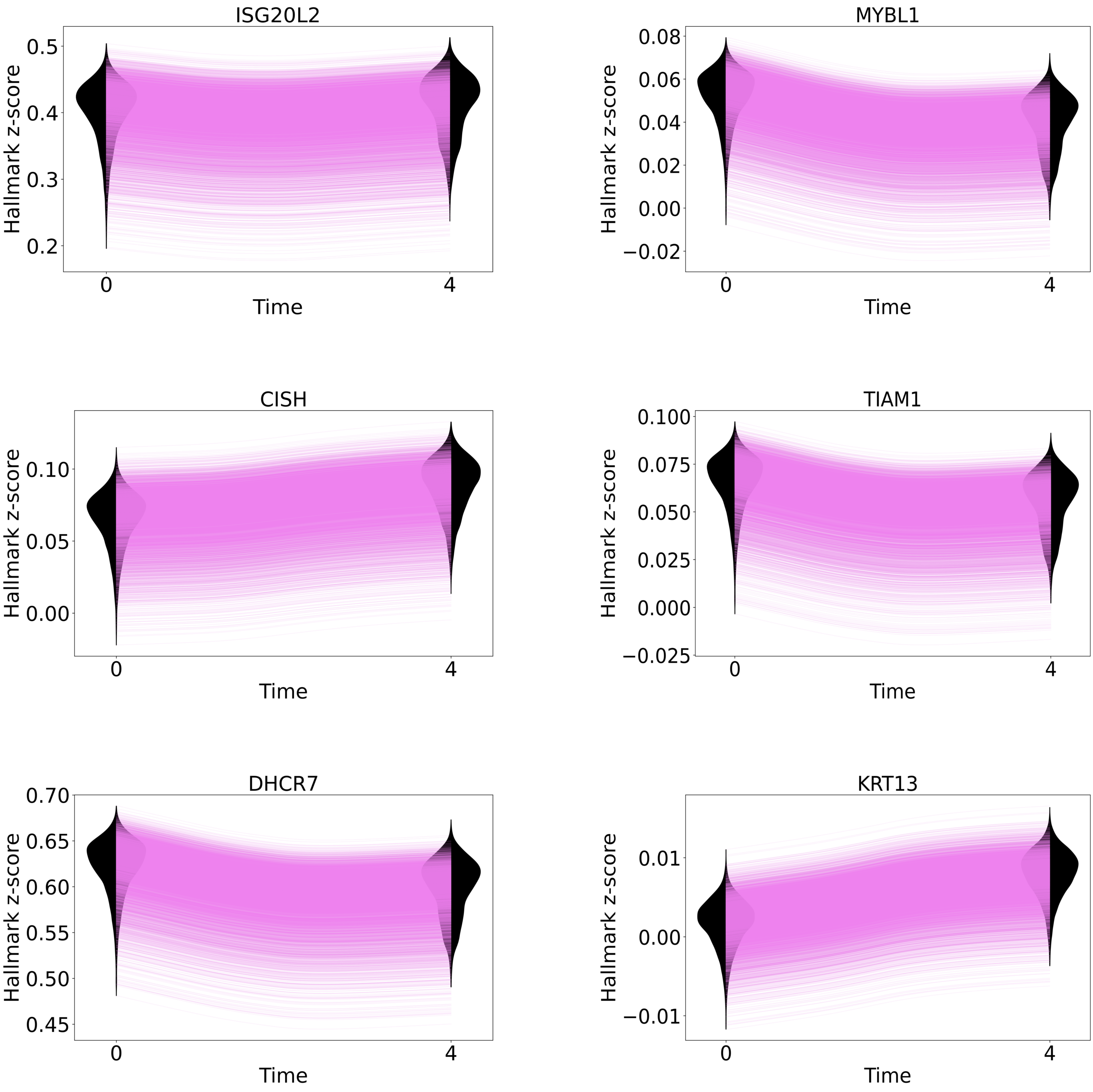

ISG20L2
MYBL1
CISH
TIAM1
DHCR7
KRT13
Hallmark z-score
Time
0
4
0.5
0.4
0.3
0.2
0.08
0.06
0.04
0.02
0.00
−0.02
0.10
0.05
0.00
0.100
0.075
0.050
0.025
0.000
−0.025
0.70
0.65
0.60
0.55
0.50
0.45
0.01
0.00
−0.01

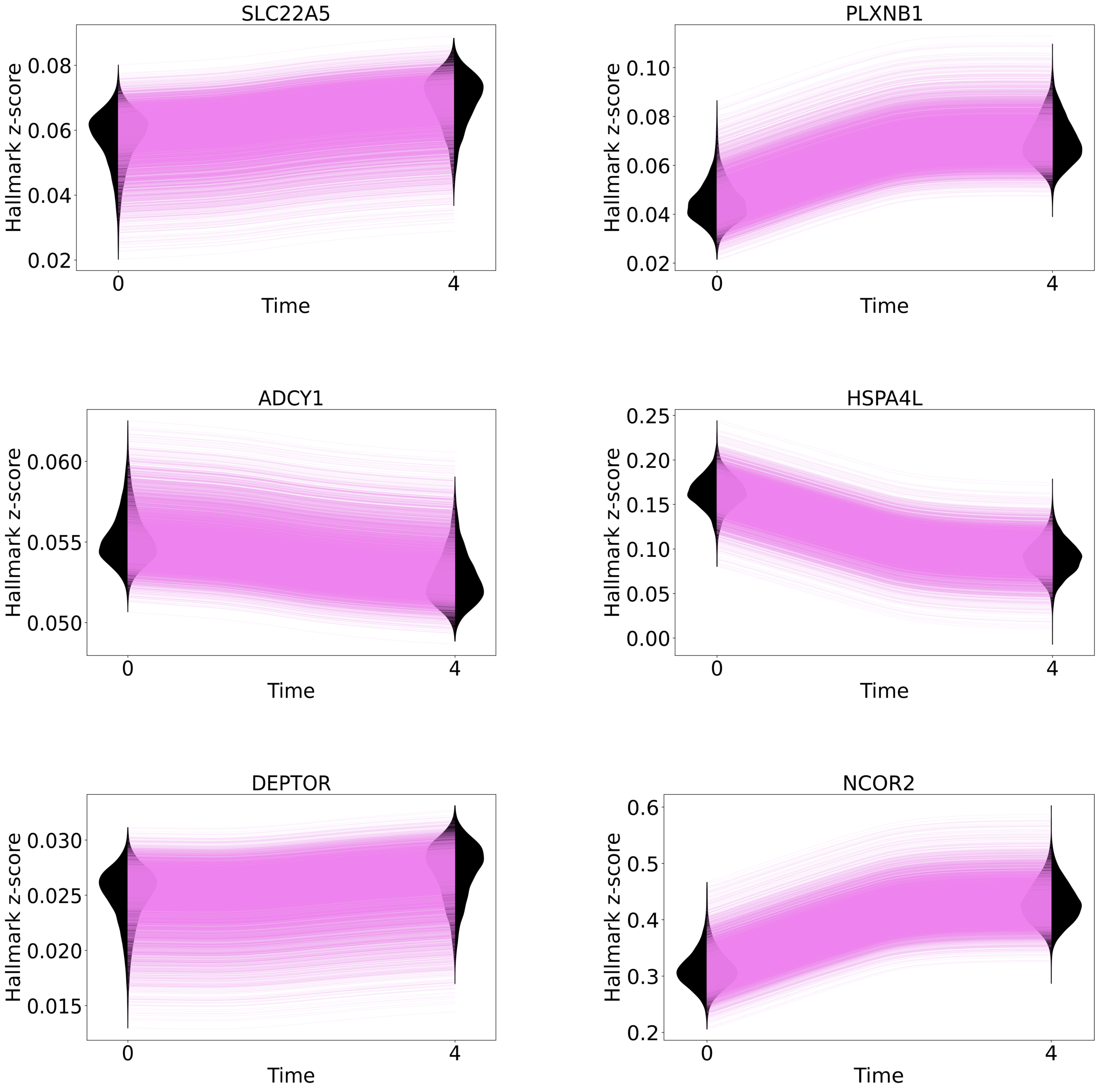

SLC22A5
PLXNB1
ADCY1
HSPA4L
DEPTOR
NCOR2
Hallmark z-score
Time
0
4
0.02
0.04
0.06
0.08
0.10
0.050
0.055
0.060
0.00
0.05
0.15
0.20
0.25
0.015
0.020
0.025
0.030
0.2
0.3
0.4
0.5
0.6

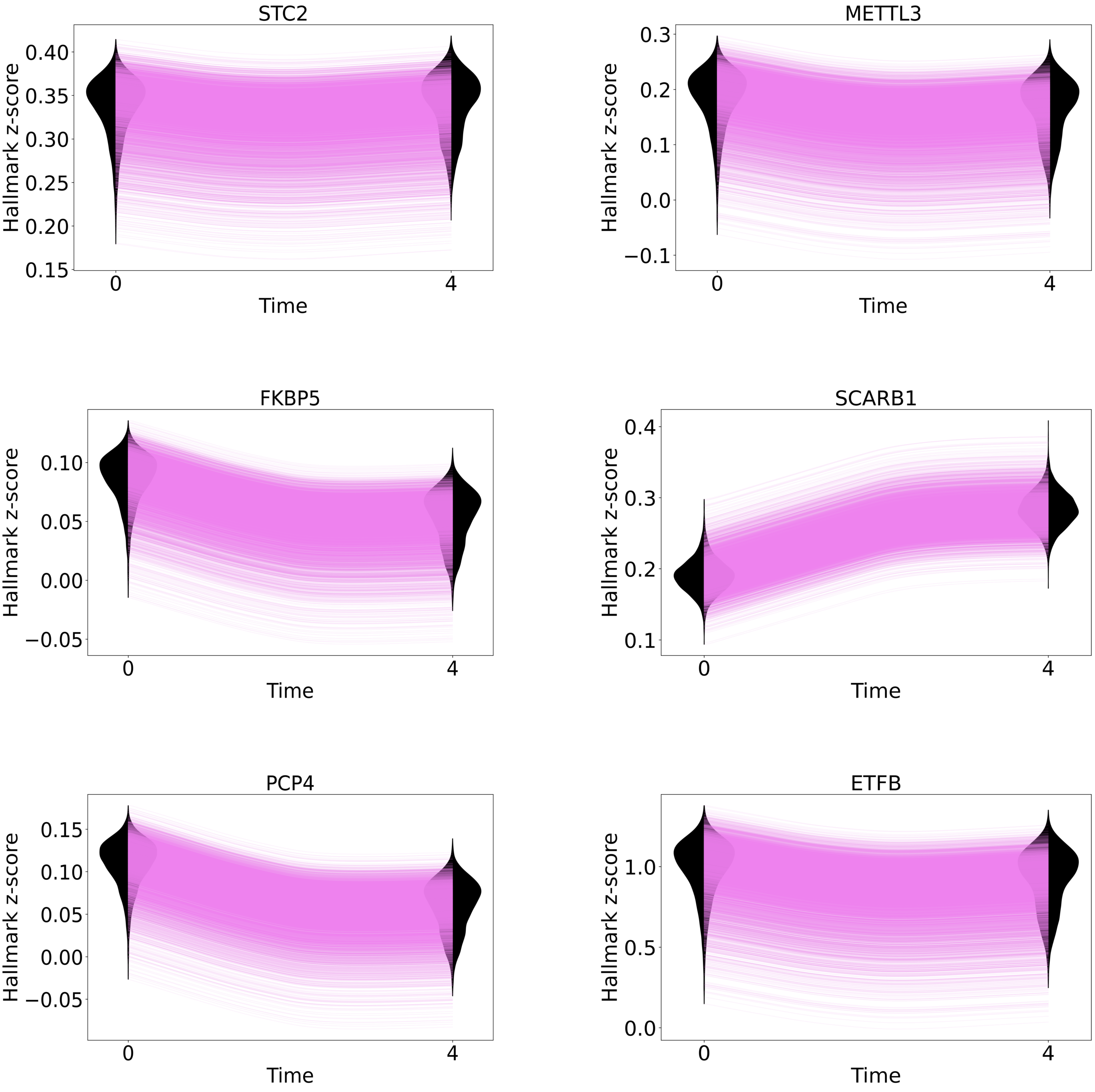

STC2
METTL3
FKBP5
SCARB1
PCP4
ETFB
Hallmark z-score
Time
0
4
0.40
0.35
0.30
0.25
0.20
0.15
0.3
0.2
0.1
0.0
−0.1
0.10
0.05
0.00
−0.05
0.4
0.15
1.0
0.5

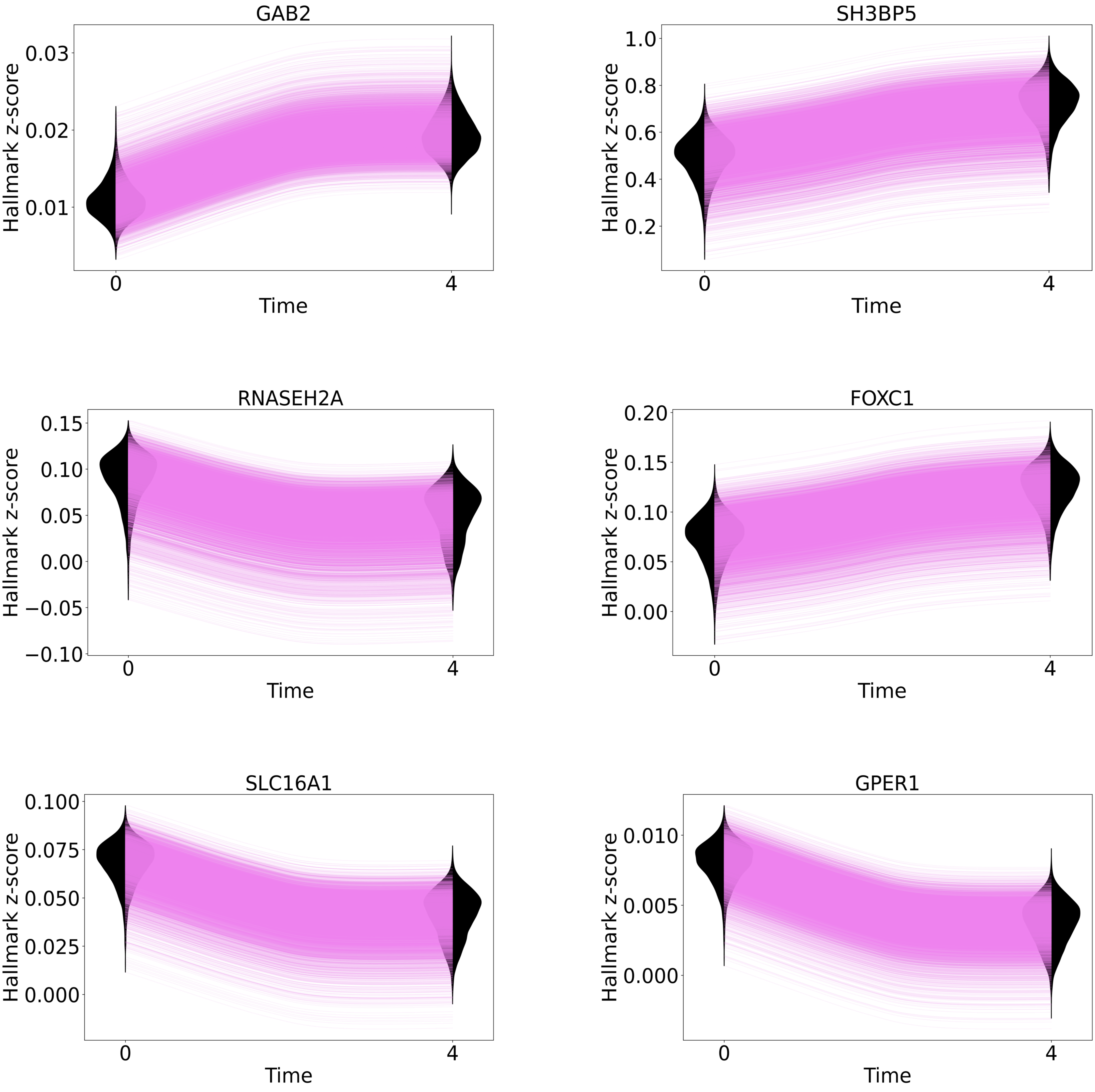
GAB2
SH3BP5
RNASEH2A
FOXC1
SLC16A1
GPER1
Hallmark z-score
Time
0
4
0.01
0.02
0.03
0.2
0.4
0.6
0.8
1.0
−0.10
−0.05
0.00
0.05
0.10
0.15
0.20
0.000
0.025
0.050
0.075
0.100
0.005
0.010

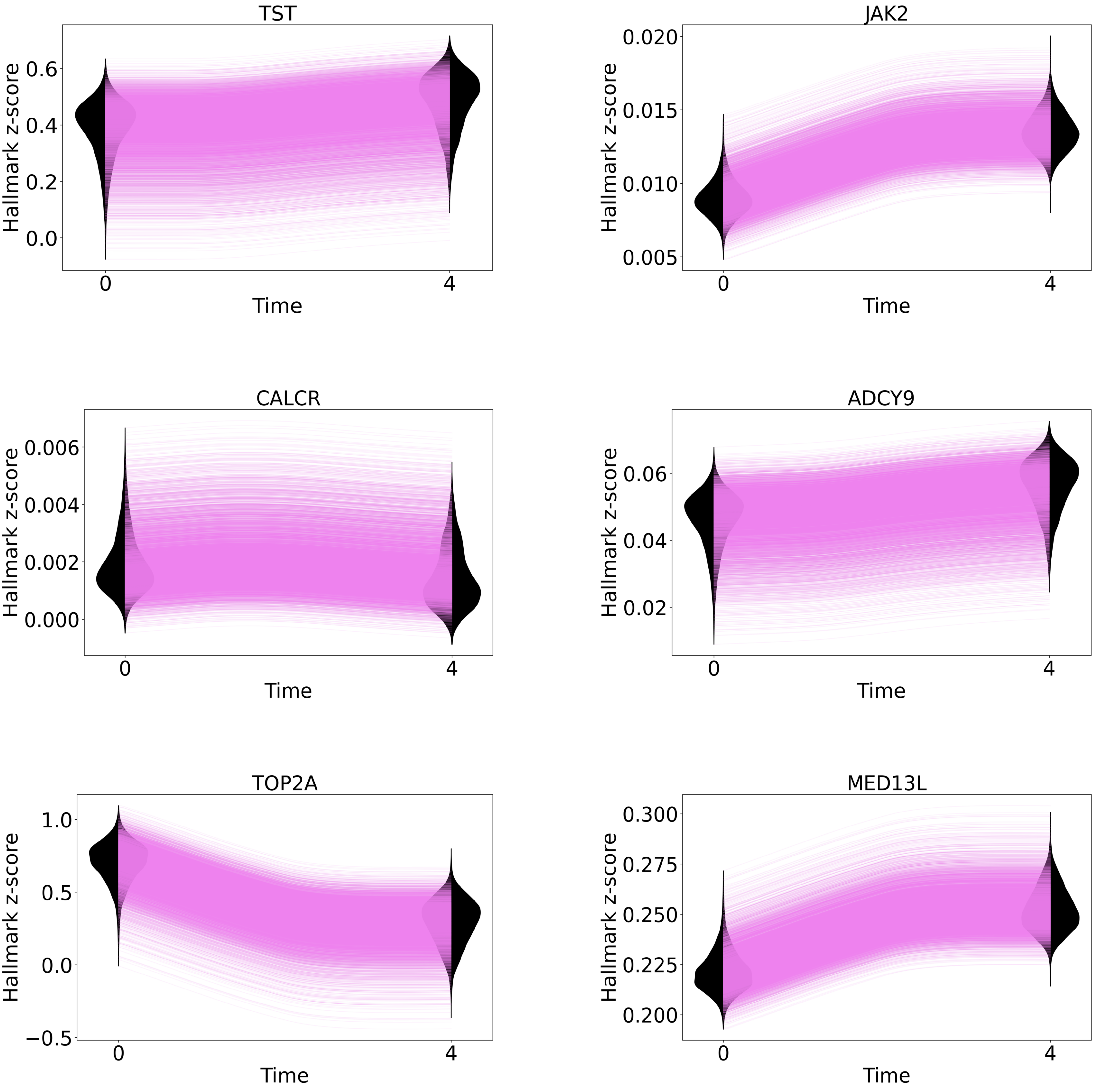

TST
JAK2
CALCR
ADCY9
TOP2A
MED13L
Hallmark z-score
Time
0
4
0.0
0.2
0.4
0.6
0.005
0.010
0.015
0.020
0.000
0.002
0.004
0.006
0.02
0.04
0.06
−0.5
0.0
0.5
1.0
0.200
0.225
0.250
0.275
0.300

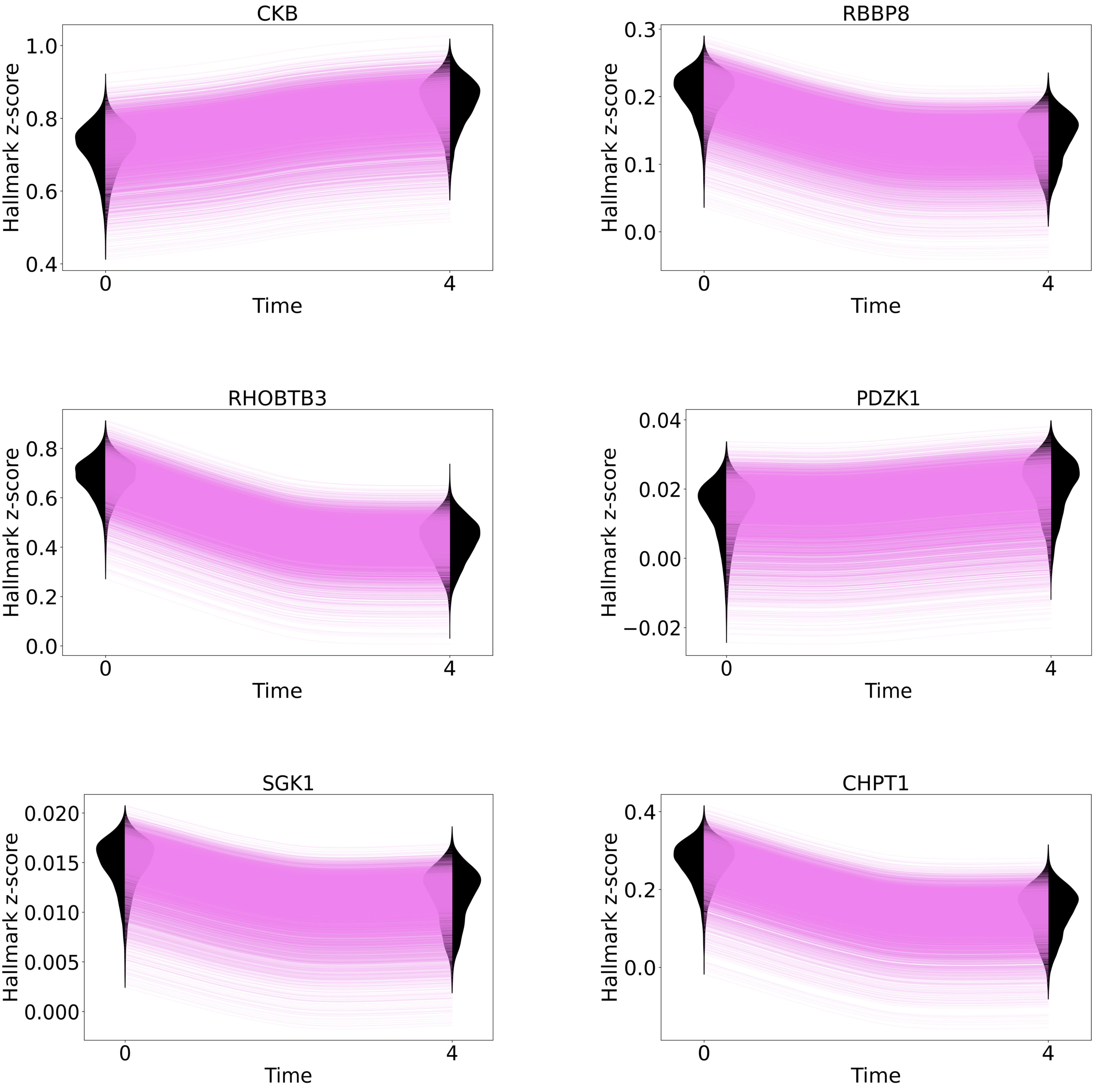

CKB
Hallmark z-score
1.0
0.8
0.6
0.4
0
4
Time
RBBP8
Hallmark z-score
0.3
0.2
0.1
0.0
0
4
Time
RHOBTB3
Hallmark z-score
0.8
0.6
0.4
0.2
0.0
0
4
Time
PDZK1
Hallmark z-score
0.04
0.02
0.00
−0.02
0
4
Time
SGK1
Hallmark z-score
0.020
0.015
0.010
0.005
0.000
0
4
Time
CHPT1
Hallmark z-score
0.4
0.2
0.0
0
4
Time

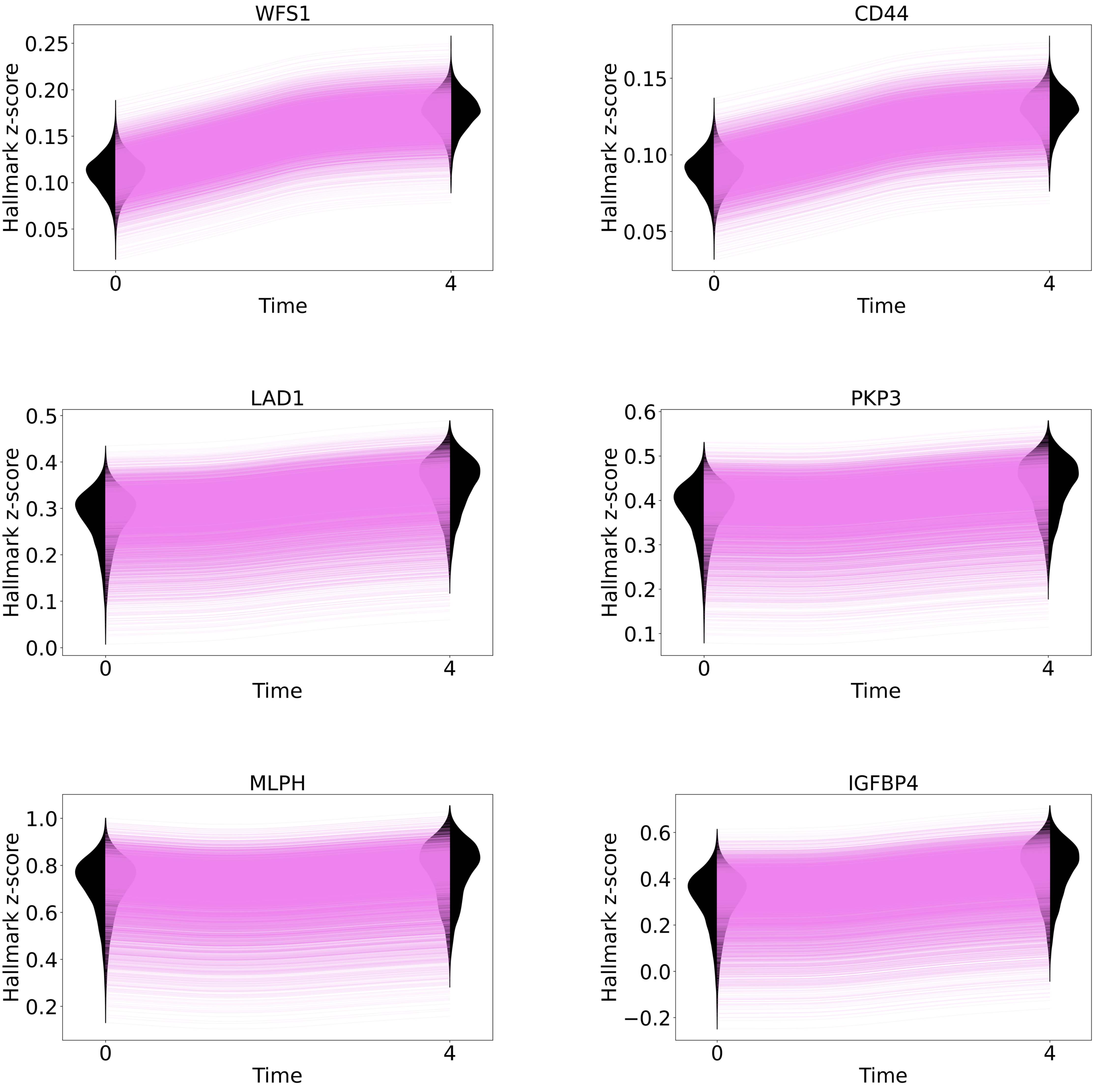

WFS1
CD44
LAD1
PKP3
MLPH
IGFBP4
Hallmark z-score
Time
0
4
0.25
0.20
0.15
0.10
0.05
0.5
0.4
0.3
0.2
0.1
0.0
0.6
1.0
0.8
−0.2

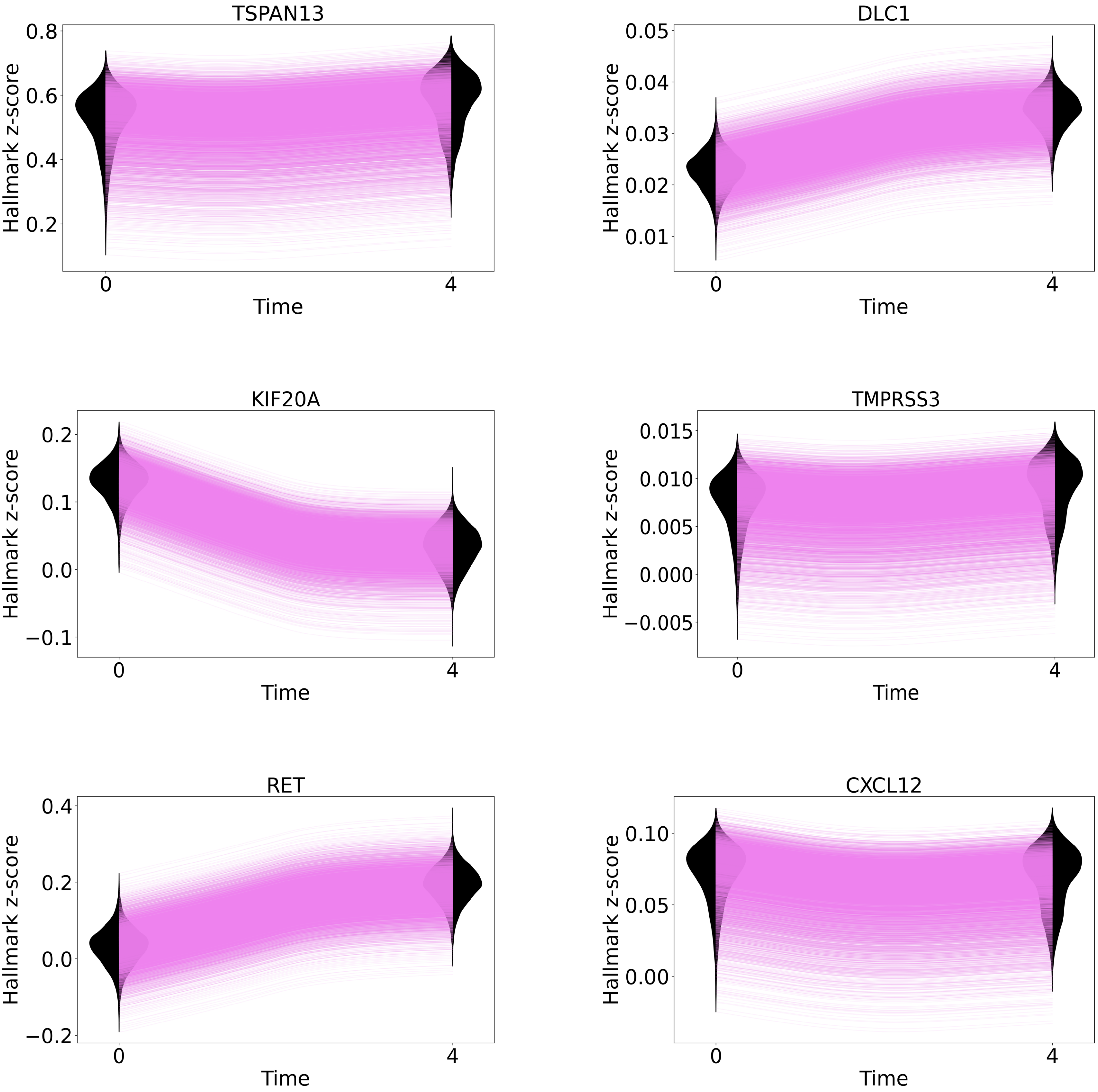

TSPAN13
DLC1
KIF20A
TMPRSS3
RET
CXCL12
Hallmark z-score
Time
0
4
0.2
0.4
0.6
0.8
0.01
0.02
0.03
0.04
0.05
−0.1
0.0
0.1
0.2
−0.005
0.000
0.005
0.010
0.015
−0.2
0.0
0.2
0.4
0.00
0.05
0.10

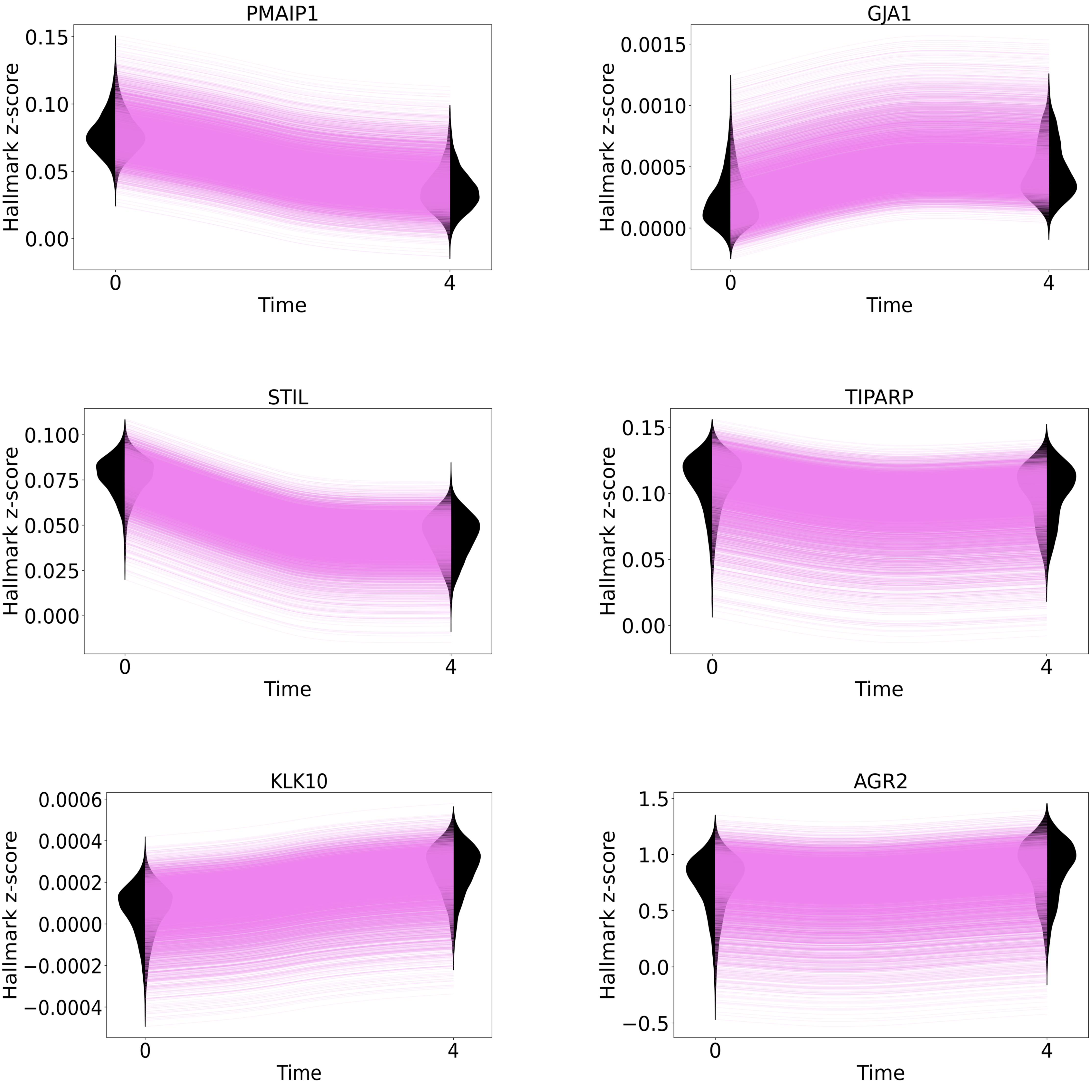
PMAIP1
GJA1
STIL
TIPARP
KLK10
AGR2
Hallmark z-score
Time
0
4
0.15
0.10
0.05
0.00
0.0015
0.0010
0.0005
0.0000
0.100
0.075
0.050
0.025
0.000
0.15
0.10
0.05
0.00
0.0006
0.0004
0.0002
0.0000
−0.0002
−0.0004
1.5
1.0
0.5
0.0
−0.5

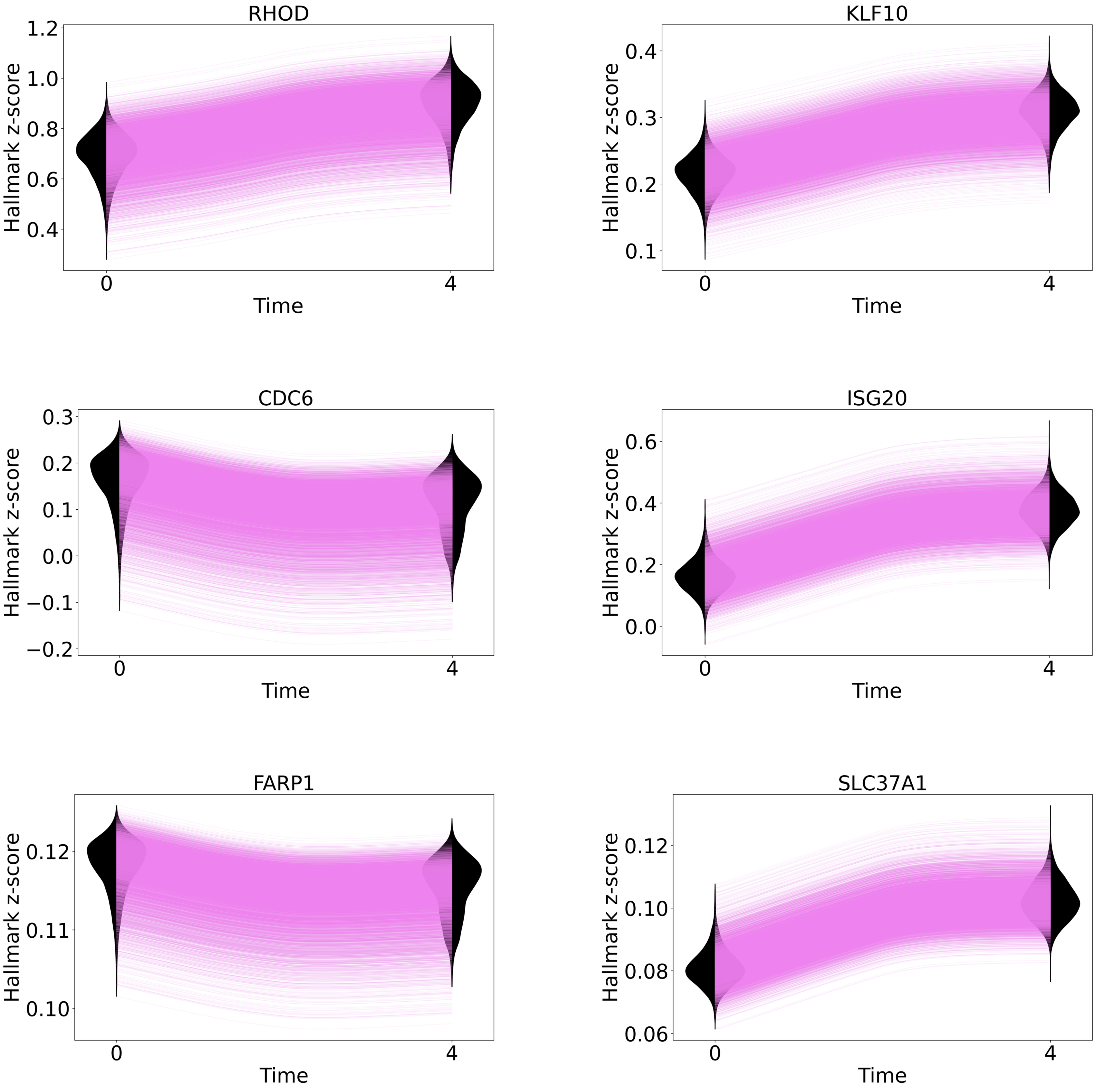
RHOD
KLF10
CDC6
ISG20
FARP1
SLC37A1
Hallmark z-score
Time
0
4
1.2
1.0
0.8
0.6
0.4
0.4
0.3
0.2
0.1
0.3
0.2
0.1
0.0
−0.1
−0.2
0.6
0.4
0.2
0.0
0.12
0.11
0.10
0.12
0.10
0.08
0.06

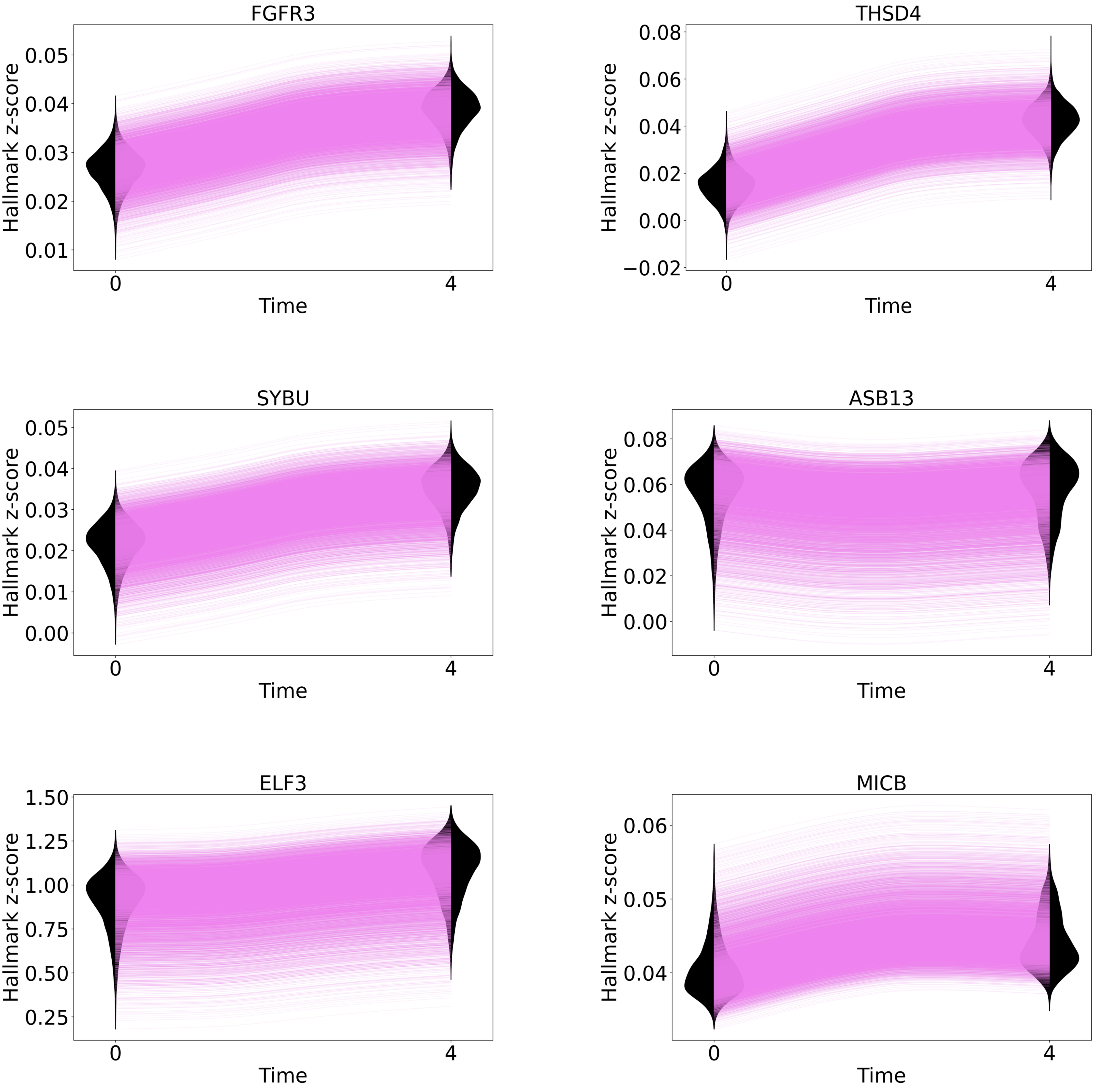
FGFR3
THSD4
SYBU
ASB13
ELF3
MICB
Hallmark z-score
Time
0
4
0.05
0.04
0.03
0.02
0.01
0.08
0.06
0.00
−0.02
1.50
1.25
1.00
0.75
0.50
0.25

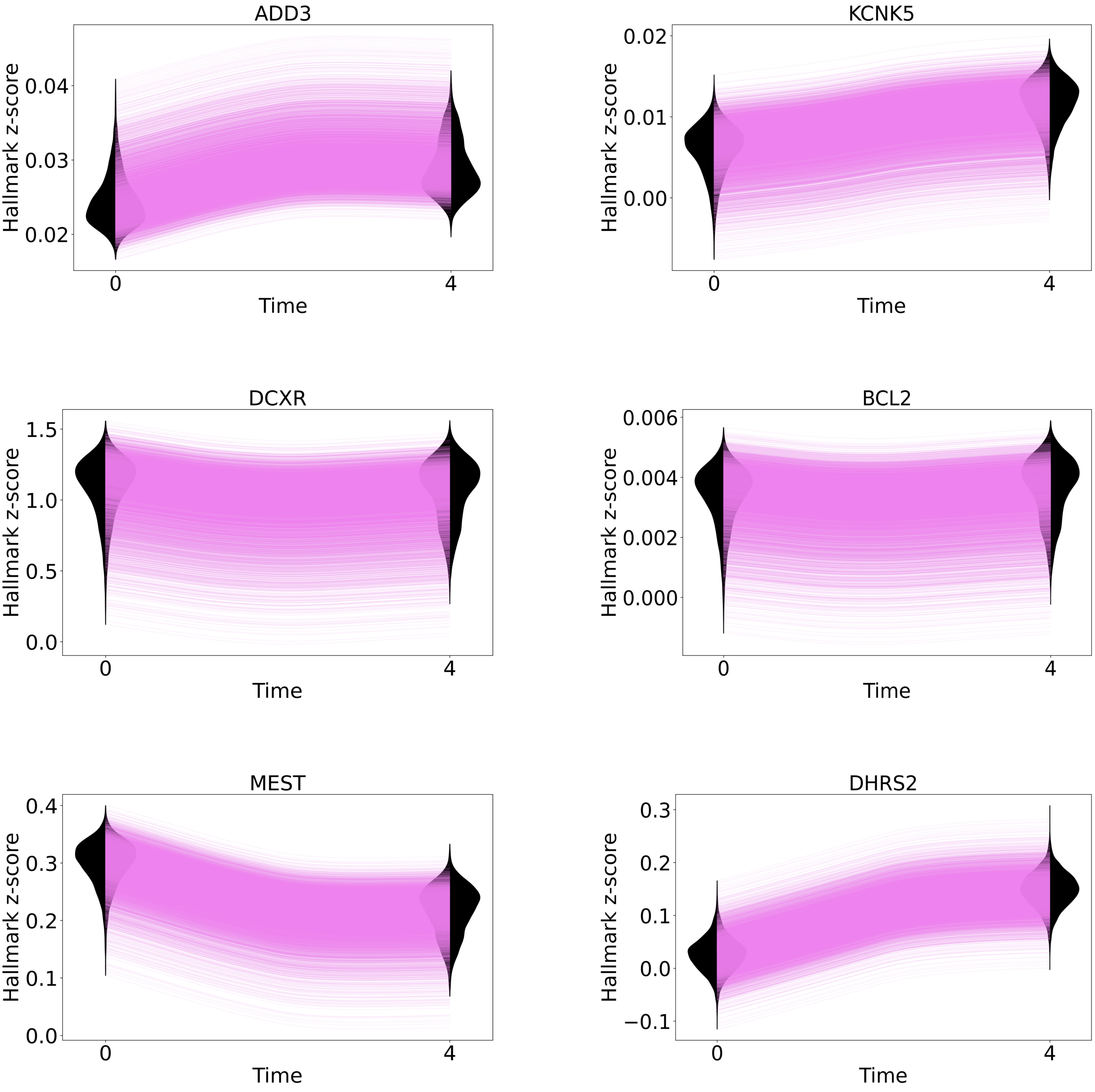
ADD3
KCNK5
DCXR
BCL2
MEST
DHRS2
Hallmark z-score
Time
0
4
0.02
0.03
0.04
0.00
0.01
0.0
0.5
1.0
1.5
0.000
0.002
0.004
0.006
0.1
0.2
0.3
0.4
−0.1

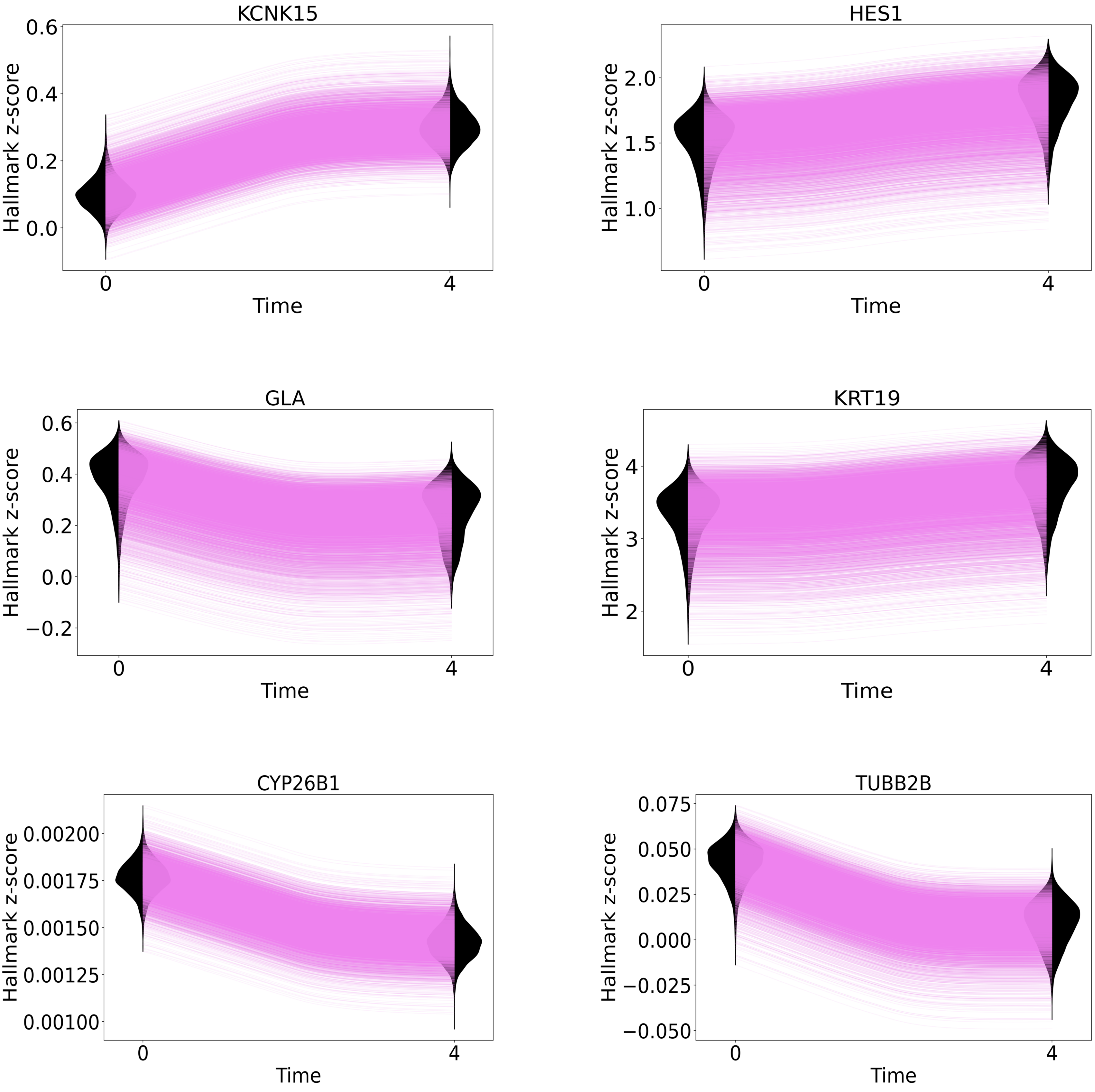

KCNK15
HES1
GLA
KRT19
CYP26B1
TUBB2B
Hallmark z-score
Time
0
4
0.6
0.4
0.2
0.0
2.0
1.5
1.0
−0.2
4
3
2
0.00200
0.00175
0.00150
0.00125
0.00100
0.075
0.050
0.025
0.000
−0.025
−0.050

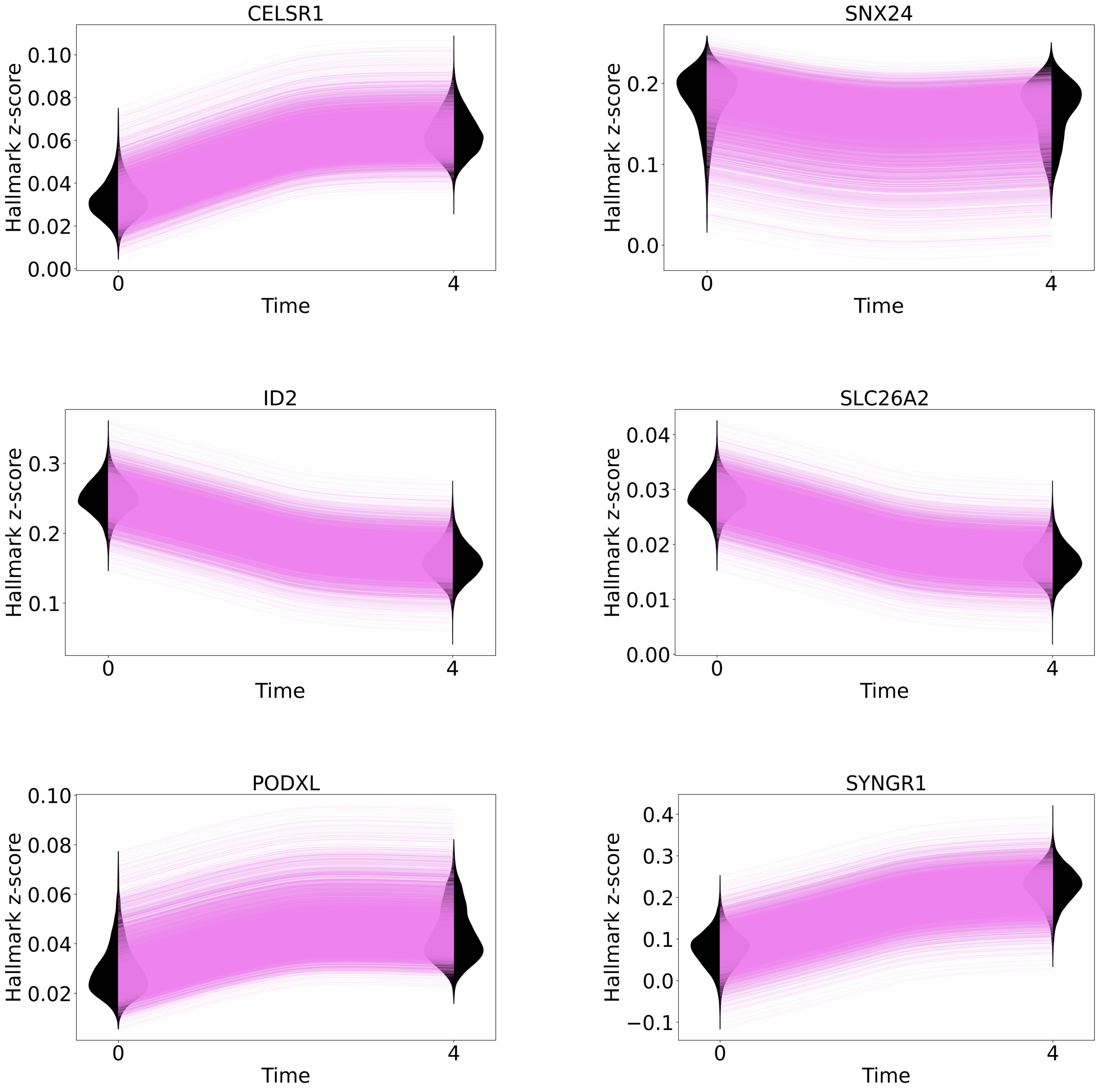

CELSR1
SNX24
ID2
SLC26A2
PODXL
SYNGR1
Hallmark z-score
Time
0
4
0.00
0.02
0.04
0.06
0.08
0.10
0.0
0.1
0.2
0.3
0.01
0.03
−0.1
0.4

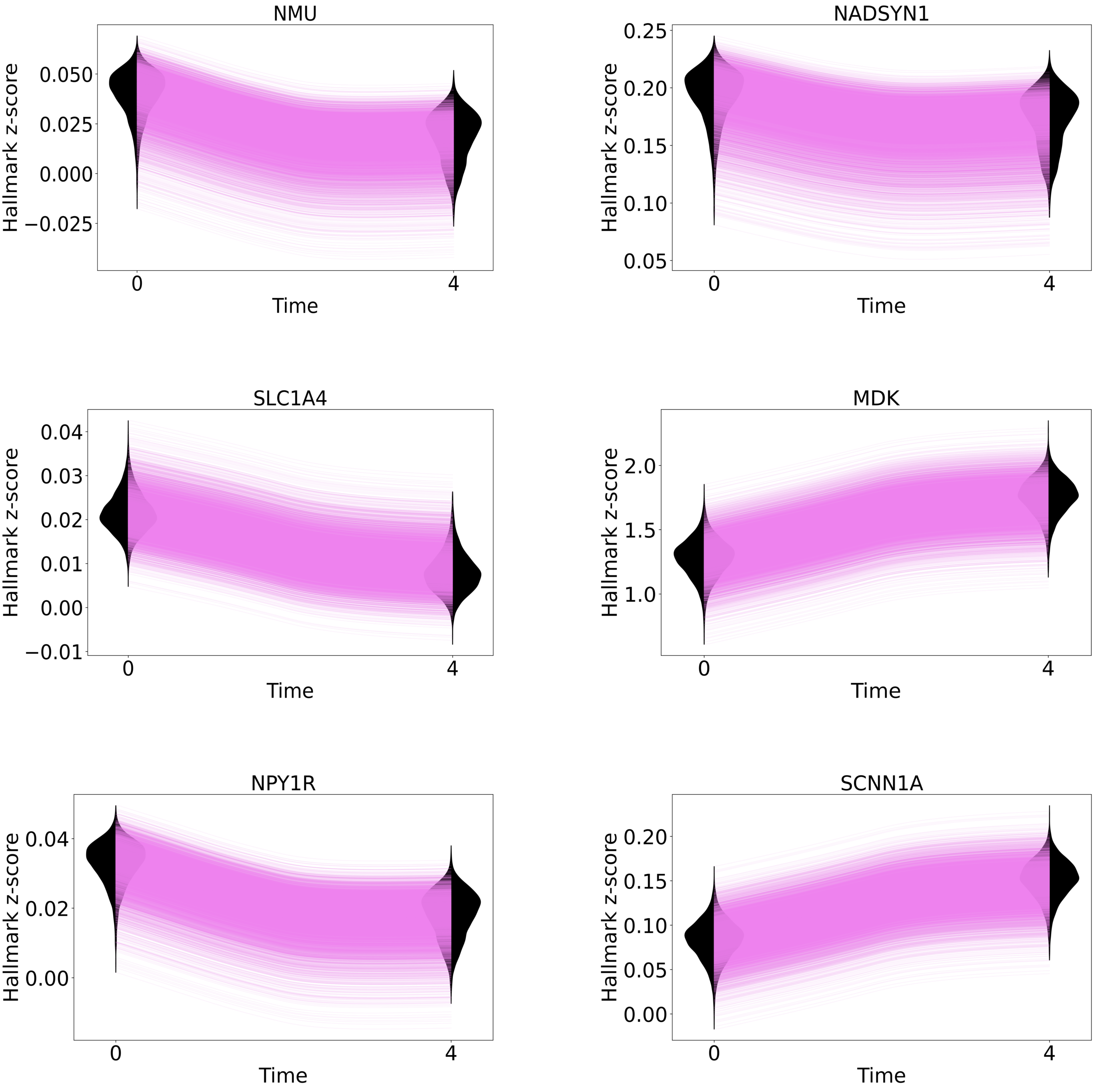
NMU
Hallmark z-score
0.050
0.025
0.000
−0.025
0
4
Time
NADSYN1
Hallmark z-score
0.25
0.20
0.15
0.10
0.05
0
4
Time
SLC1A4
Hallmark z-score
0.04
0.03
0.02
0.01
0.00
−0.01
0
4
Time
MDK
Hallmark z-score
2.0
1.5
1.0
0
4
Time
NPY1R
Hallmark z-score
0.04
0.02
0.00
0
4
Time
SCNN1A
Hallmark z-score
0.20
0.15
0.10
0.05
0.00
0
4
Time

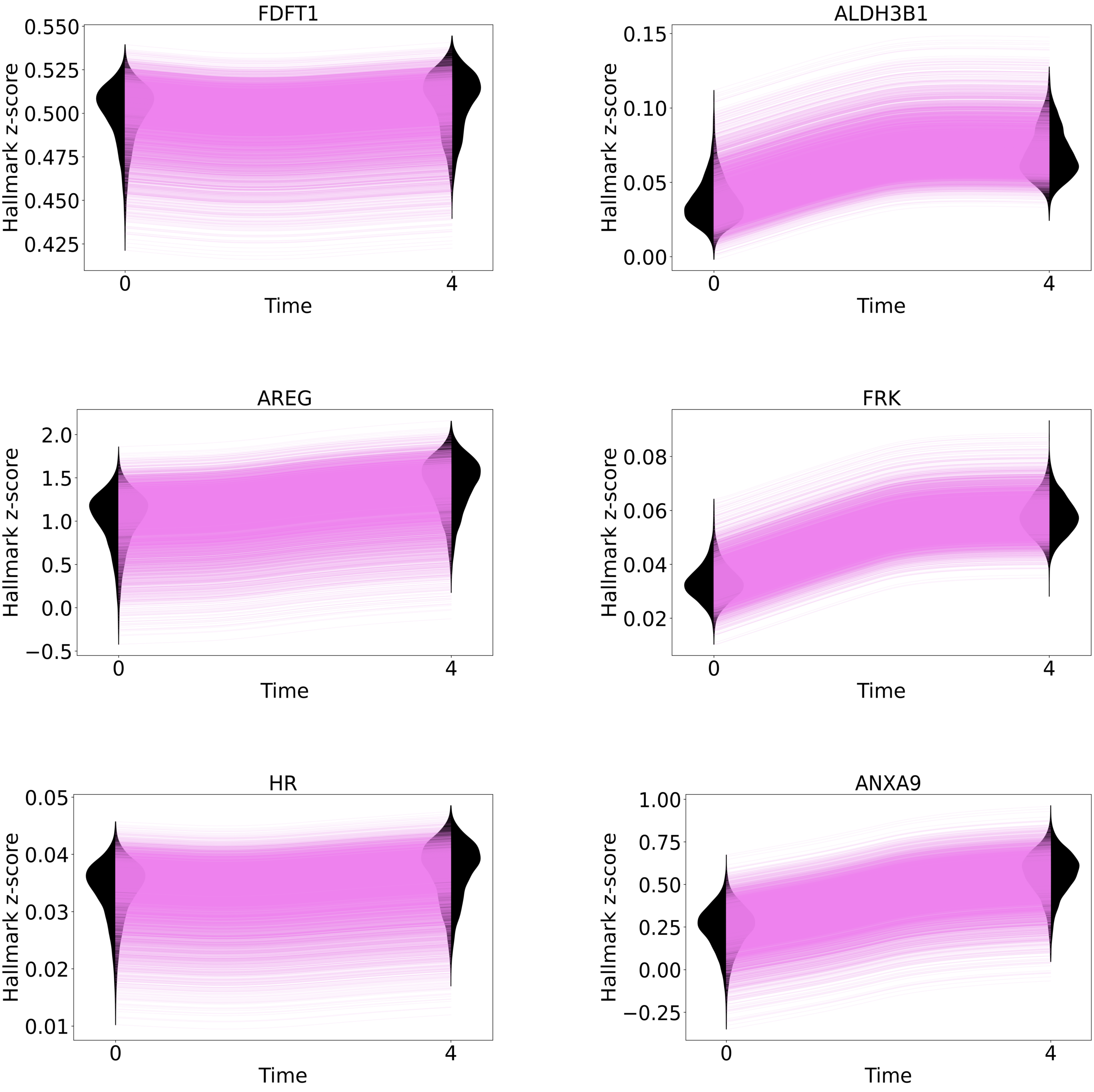
FDFT1
ALDH3B1
AREG
FRK
HR
ANXA9
Hallmark z-score
Time
0
4
0.550
0.525
0.500
0.475
0.450
0.425
0.15
0.10
0.05
0.00
2.0
1.5
1.0
0.5
0.0
−0.5
0.08
0.06
0.04
0.02
0.05
0.04
0.03
0.02
0.01
1.00
0.75
0.50
0.25
0.00
−0.25

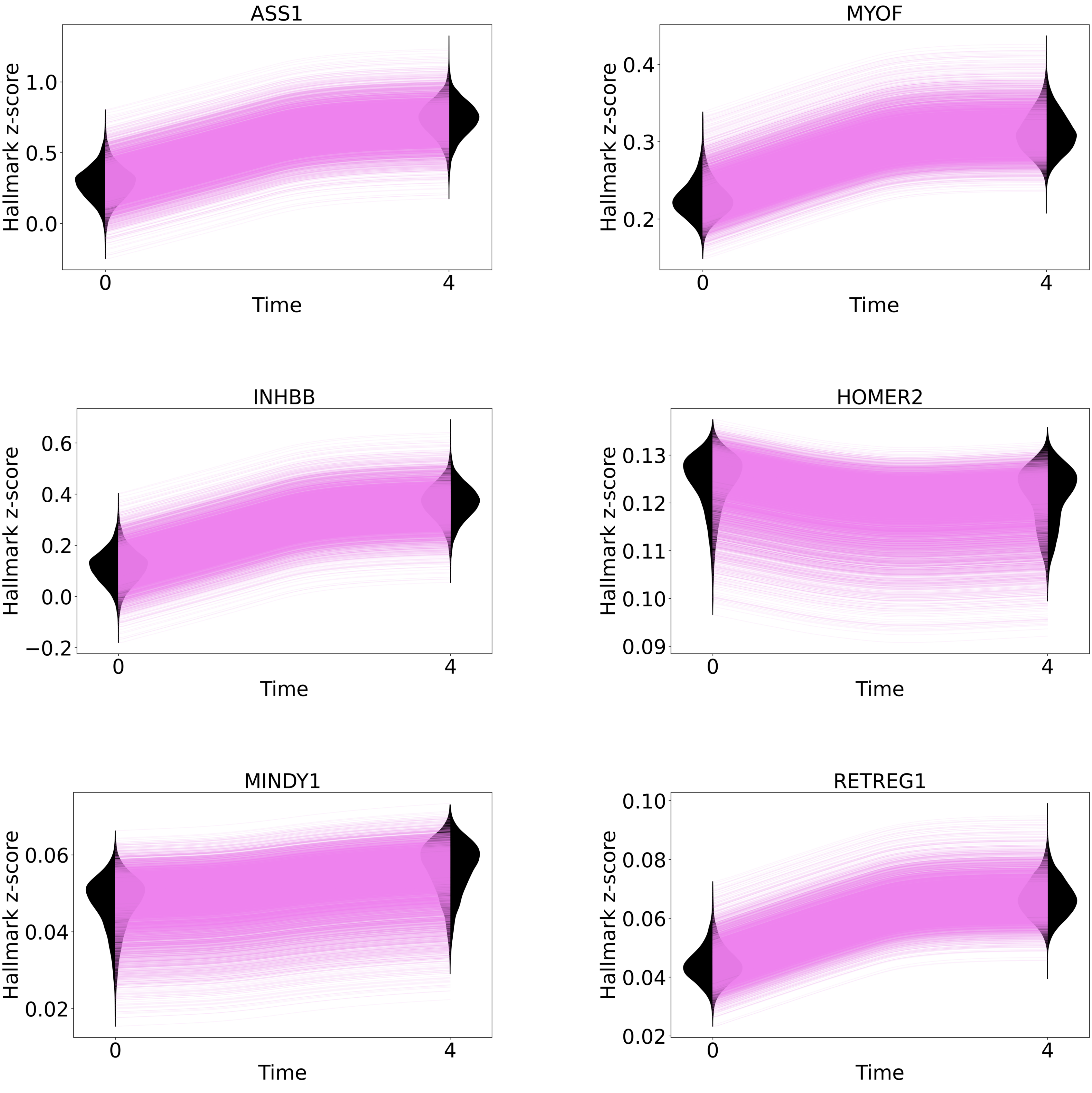
ASS1
MYOF
INHBB
HOMER2
MINDY1
RETREG1
Hallmark z-score
Time
0
4
1.0
0.5
0.0
0.4
0.3
0.2
0.6
−0.2
0.13
0.12
0.11
0.10
0.09
0.06
0.04
0.02
0.10
0.08

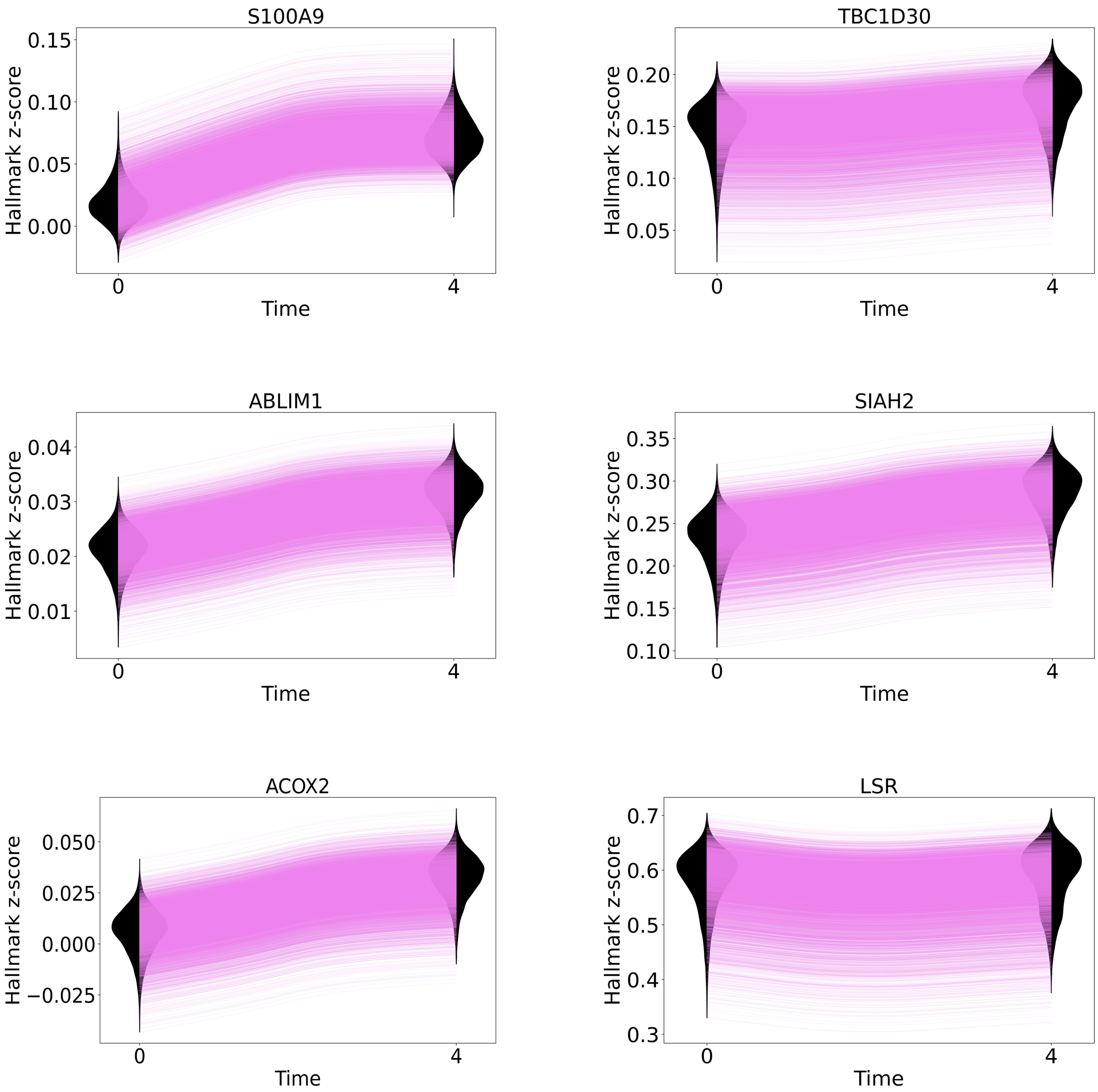
S100A9
TBC1D30
ABLIM1
SIAH2
ACOX2
LSR
Hallmark z-score
Time
0
4
0.15
0.10
0.05
0.00
0.20
0.04
0.03
0.02
0.01
0.35
0.30
0.25
0.050
0.025
0.000
−0.025
0.7
0.6
0.5
0.4
0.3

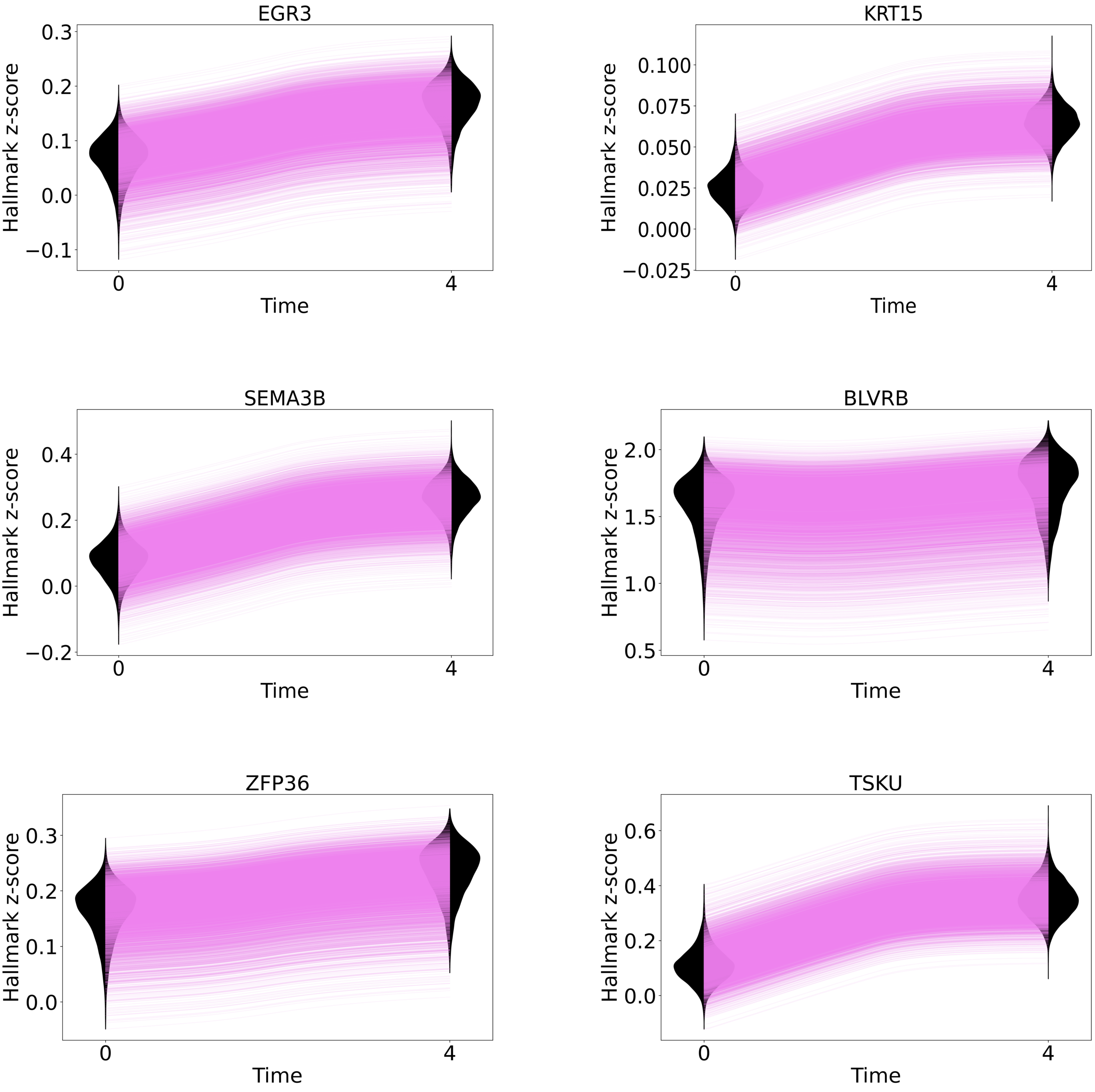

EGR3
KRT15
SEMA3B
BLVRB
ZFP36
TSKU
Hallmark z-score
Time
0
4
0.3
0.2
0.1
0.0
−0.1
0.100
0.075
0.050
0.025
0.000
−0.025
0.4
−0.2
2.0
1.5
1.0
0.5
0.6

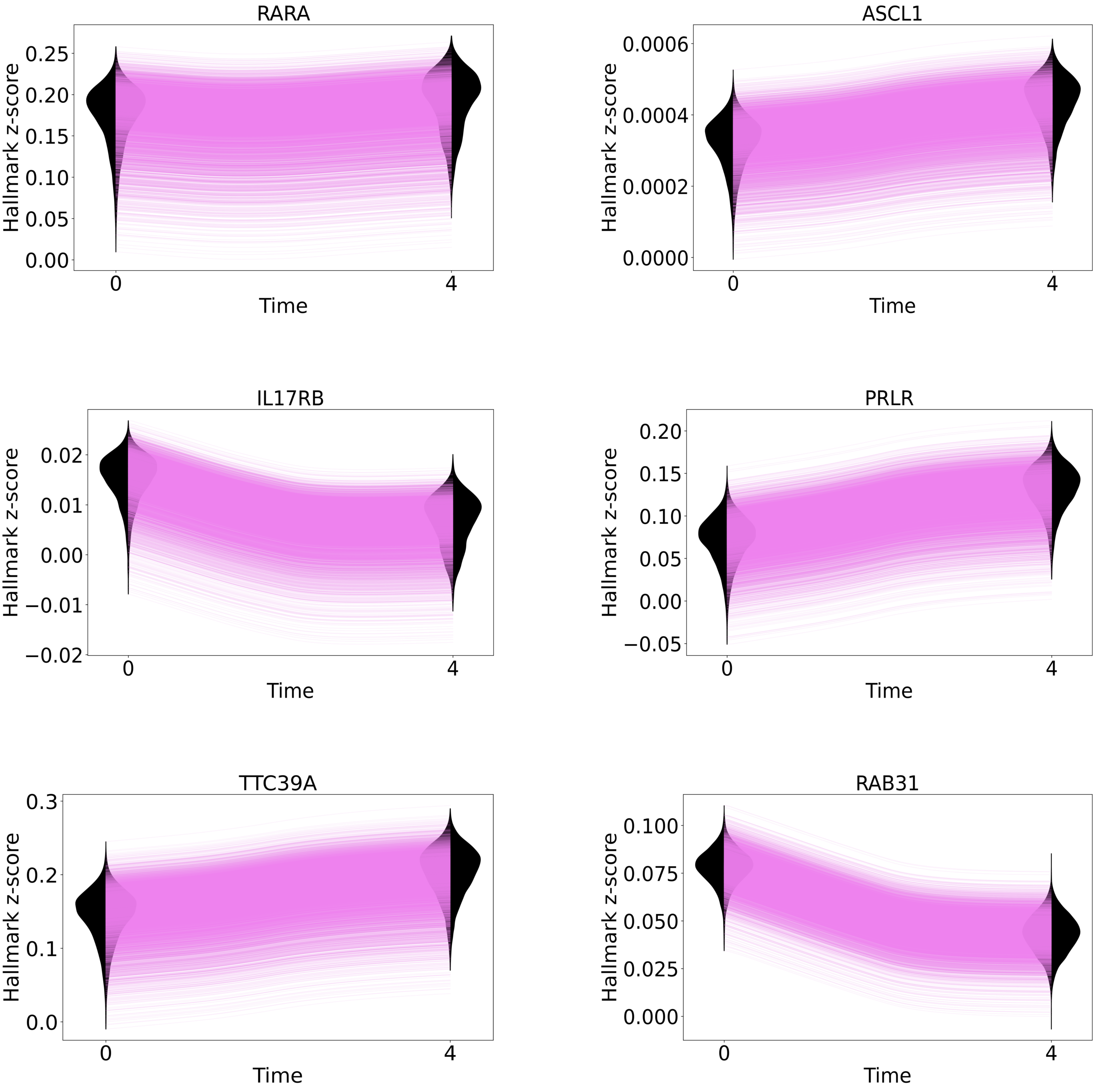
RARA
ASCL1
IL17RB
PRLR
TTC39A
RAB31
Hallmark z-score
Time
0
4
0.00
0.05
0.10
0.15
0.20
0.25
0.0000
0.0002
0.0004
0.0006
−0.02
−0.01
0.00
0.01
0.02
−0.05
0.00
0.05
0.10
0.15
0.20
0.0
0.1
0.2
0.3
0.000
0.025
0.050
0.075
0.100

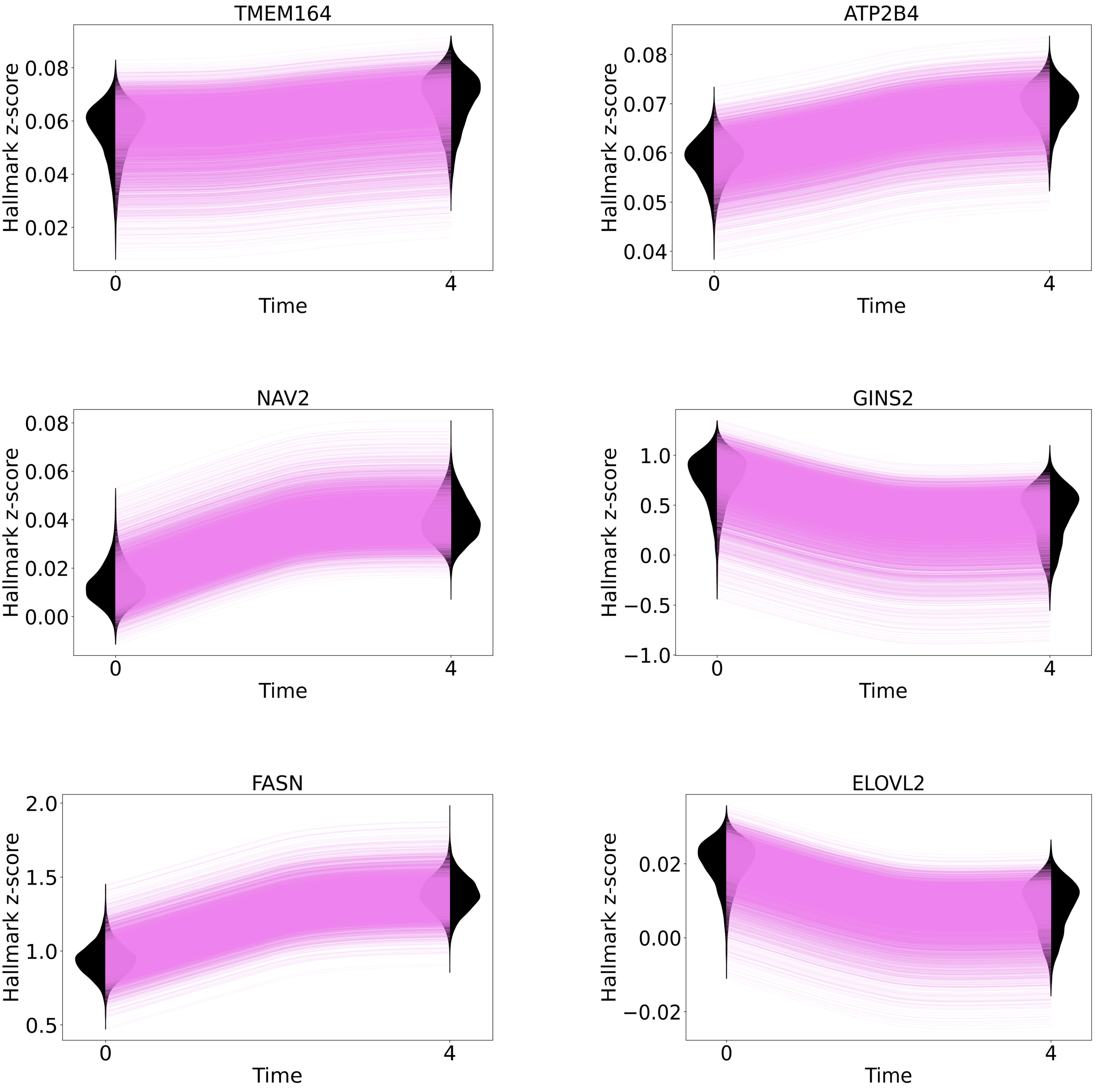
TMEM164
ATP2B4
NAV2
GINS2
FASN
ELOVL2
Hallmark z-score
Time
0
4

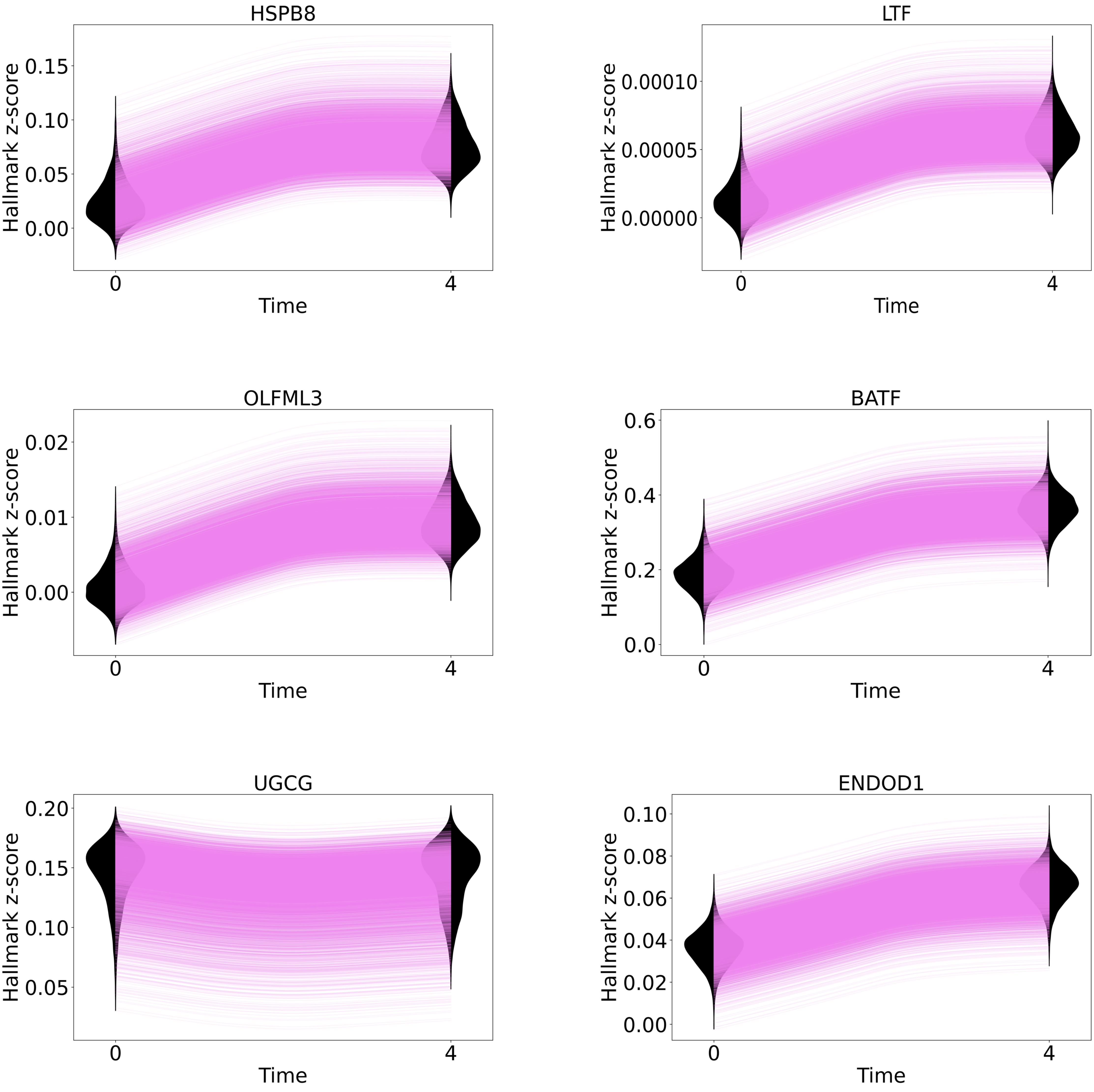
HSPB8
Hallmark z-score
0.15
0.10
0.05
0.00
0
4
Time
LTF
Hallmark z-score
0.00010
0.00005
0.00000
0
4
Time
OLFML3
Hallmark z-score
0.02
0.01
0.00
0
4
Time
BATF
Hallmark z-score
0.6
0.4
0.2
0.0
0
4
Time
UGCG
Hallmark z-score
0.20
0.15
0.10
0.05
0
4
Time
ENDOD1
Hallmark z-score
0.10
0.08
0.06
0.04
0.02
0.00
0
4
Time

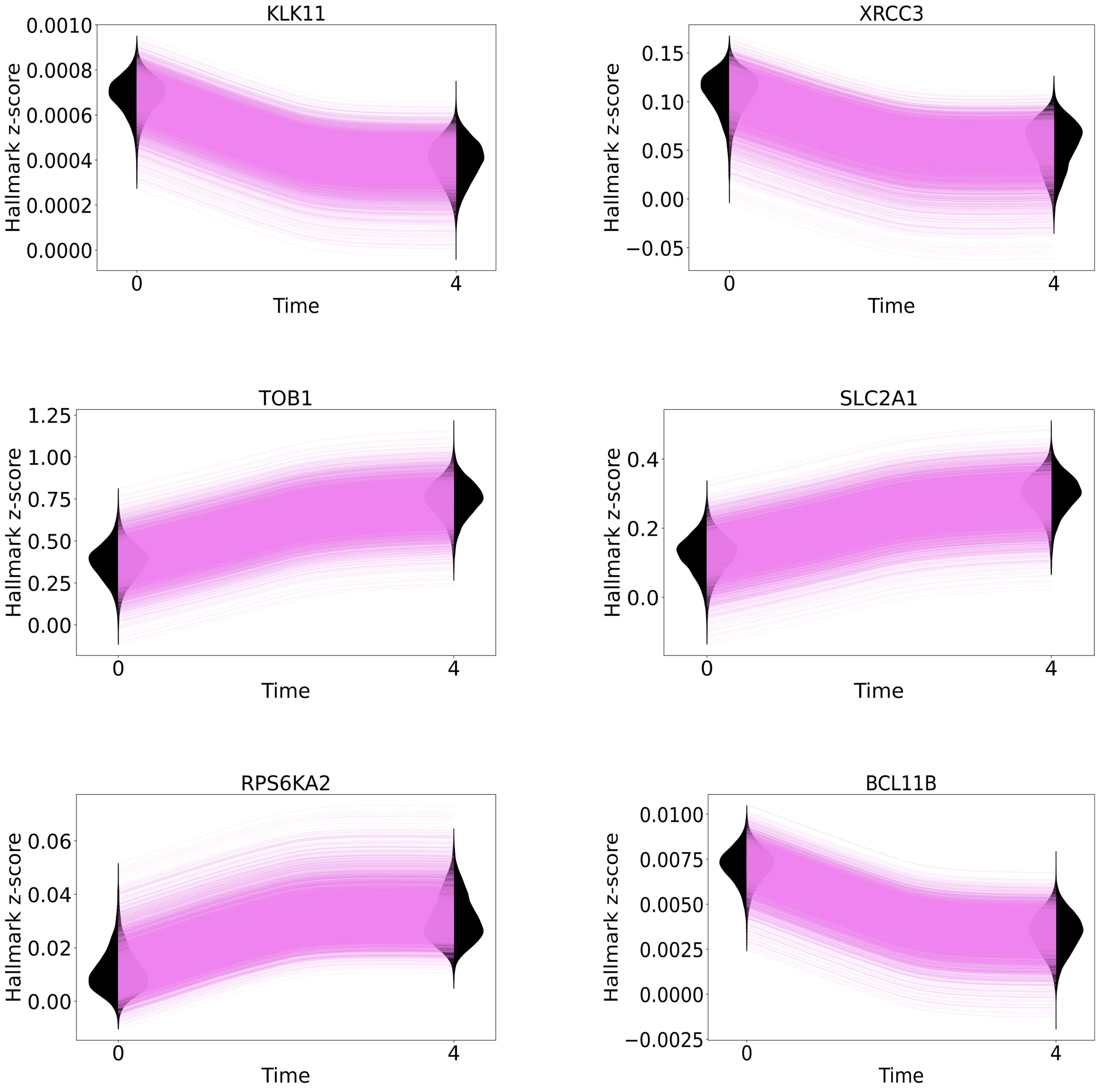
KLK11
XRCC3
TOB1
SLC2A1
RPS6KA2
BCL11B
Hallmark z-score
Time
0
4
0.0000
0.0002
0.0004
0.0006
0.0008
0.0010
−0.05
0.00
0.05
0.10
0.15
0.00
0.25
0.50
0.75
1.00
1.25
0.0
0.2
0.4
0.00
0.02
0.04
0.06
−0.0025
0.0000
0.0025
0.0050
0.0075
0.0100

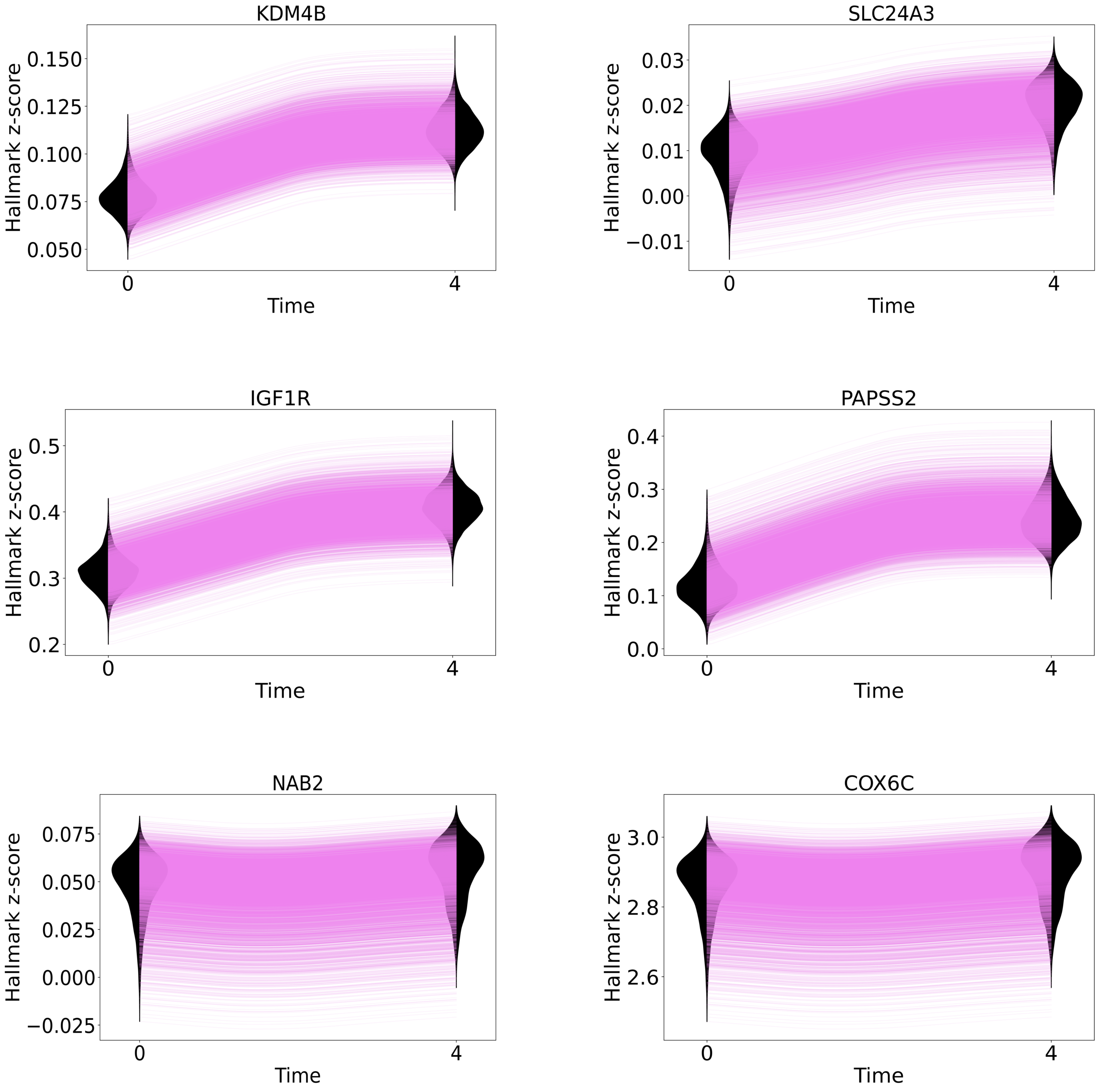
KDM4B
SLC24A3
IGF1R
PAPSS2
NAB2
COX6C
Hallmark z-score
Time
0
4
0.150
0.125
0.100
0.075
0.050
0.03
0.02
0.01
0.00
−0.01
0.5
0.4
0.3
0.2
0.1
0.0
0.075
0.050
0.025
0.000
−0.025
3.0
2.8
2.6

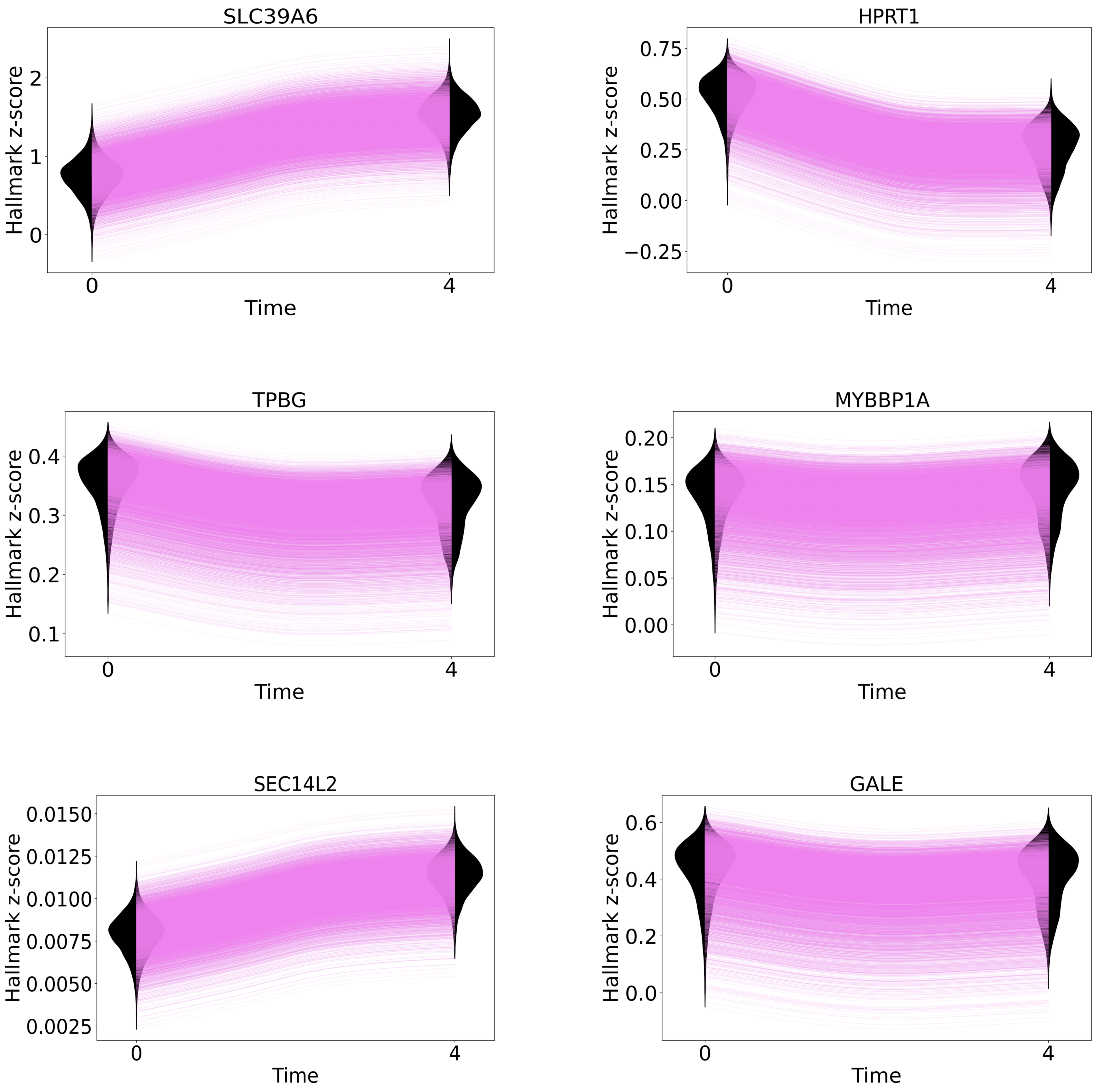

SLC39A6
HPRT1
TPBG
MYBBP1A
SEC14L2
GALE
Hallmark z-score
Time
0
4
2
1
0
0.75
0.50
0.25
0.00
−0.25
0.4
0.3
0.2
0.1
0.20
0.15
0.10
0.05
0.00
0.0150
0.0125
0.0100
0.0075
0.0050
0.0025
0.6
0.4
0.2
0.0

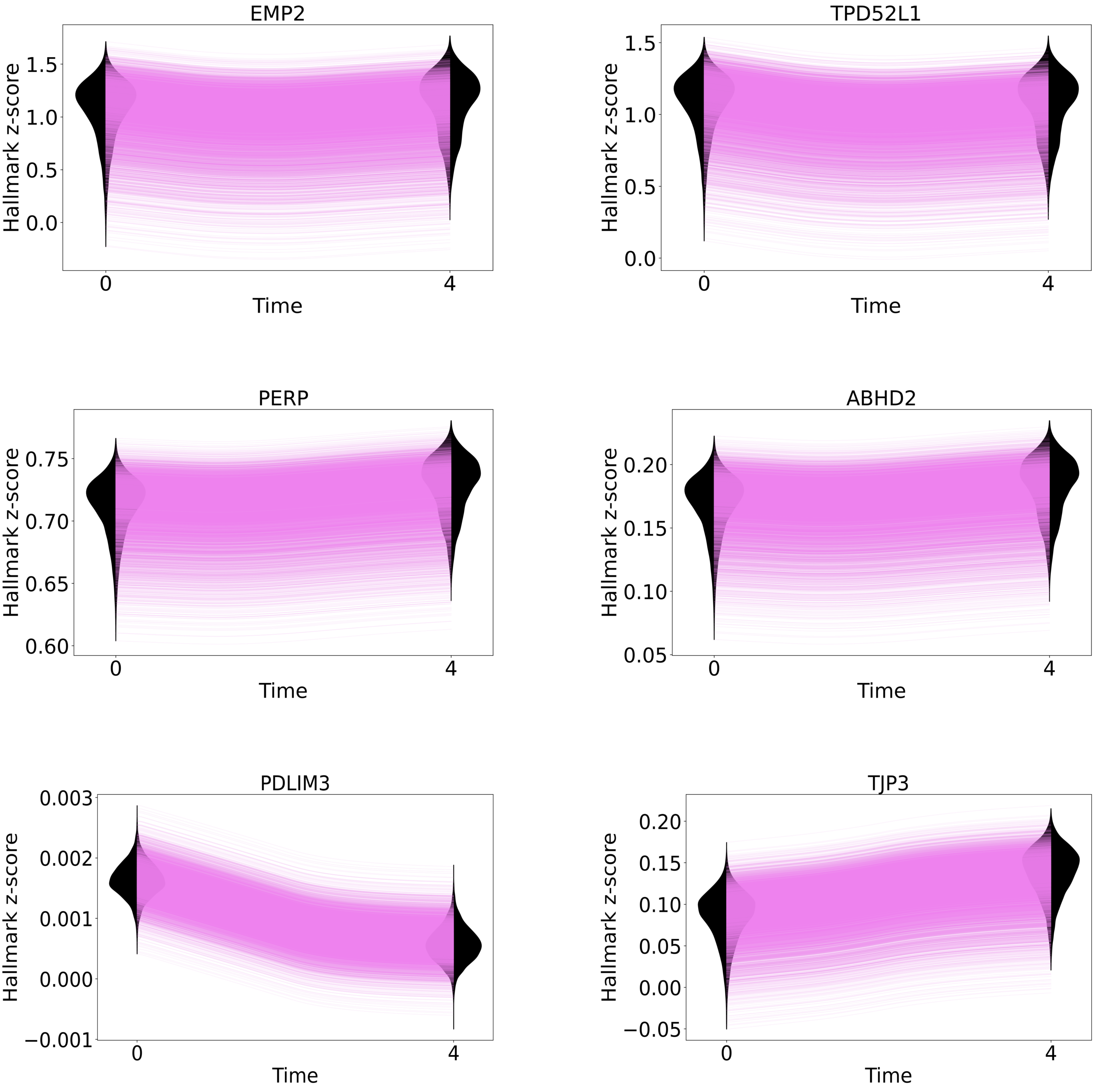

EMP2
TPD52L1
PERP
ABHD2
PDLIM3
TJP3
Hallmark z-score
Time
0
4

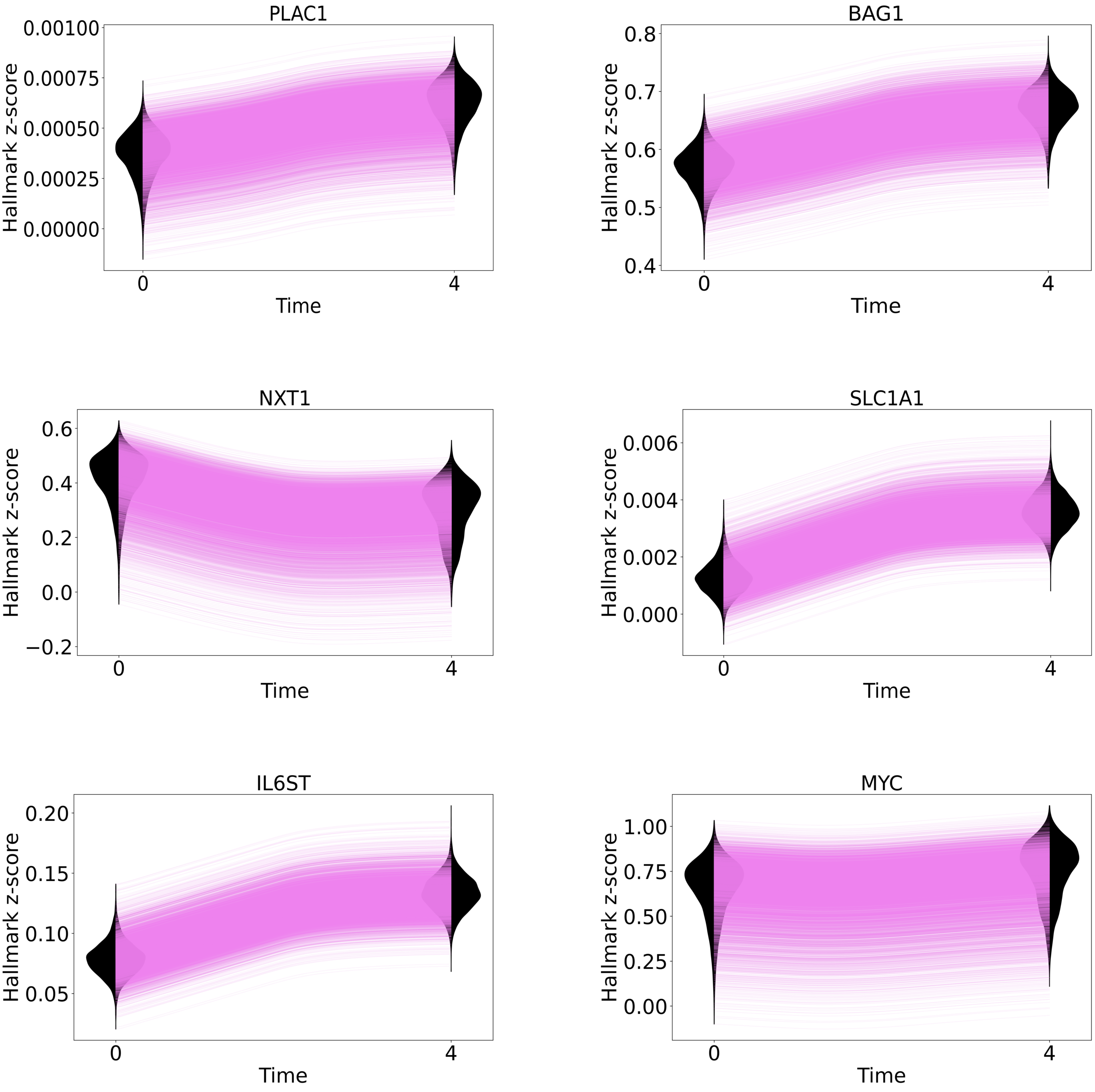

PLAC1
0.00100
0.00075
0.00050
0.00025
0.00000
Hallmark z-score
0
4
Time
BAG1
0.8
0.7
0.6
0.5
0.4
Hallmark z-score
0
4
Time
NXT1
0.6
0.4
0.2
0.0
−0.2
Hallmark z-score
0
4
Time
SLC1A1
0.006
0.004
0.002
0.000
Hallmark z-score
0
4
Time
IL6ST
0.20
0.15
0.10
0.05
Hallmark z-score
0
4
Time
MYC
1.00
0.75
0.50
0.25
0.00
Hallmark z-score
0
4
Time

Supplementary Figure 9: Single-cell, single-gene dynamics plotted as gene expression levels (y-axis) over time (x-axis), with each green curve representing an individual cell trajectory. The predicted trajectories are compared against real data distributions using violin plots (gray for test data, black for training data).
