# Supplementary material for: PROFET Predicts Continuous Gene Expression Dynamics from scRNA-seq Data to Elucidate Heterogeneity of Cancer Treatment Responses
Source: bioRxiv. 2025 Jul 3:2025.06.27.662030. Preprint. [Version 1] doi: 10.1101/2025.06.27.662030 (PMC12236938; doi:10.1101/2025.06.27.662030)
Supplement: Supplement 10 [file media-11.pdf]

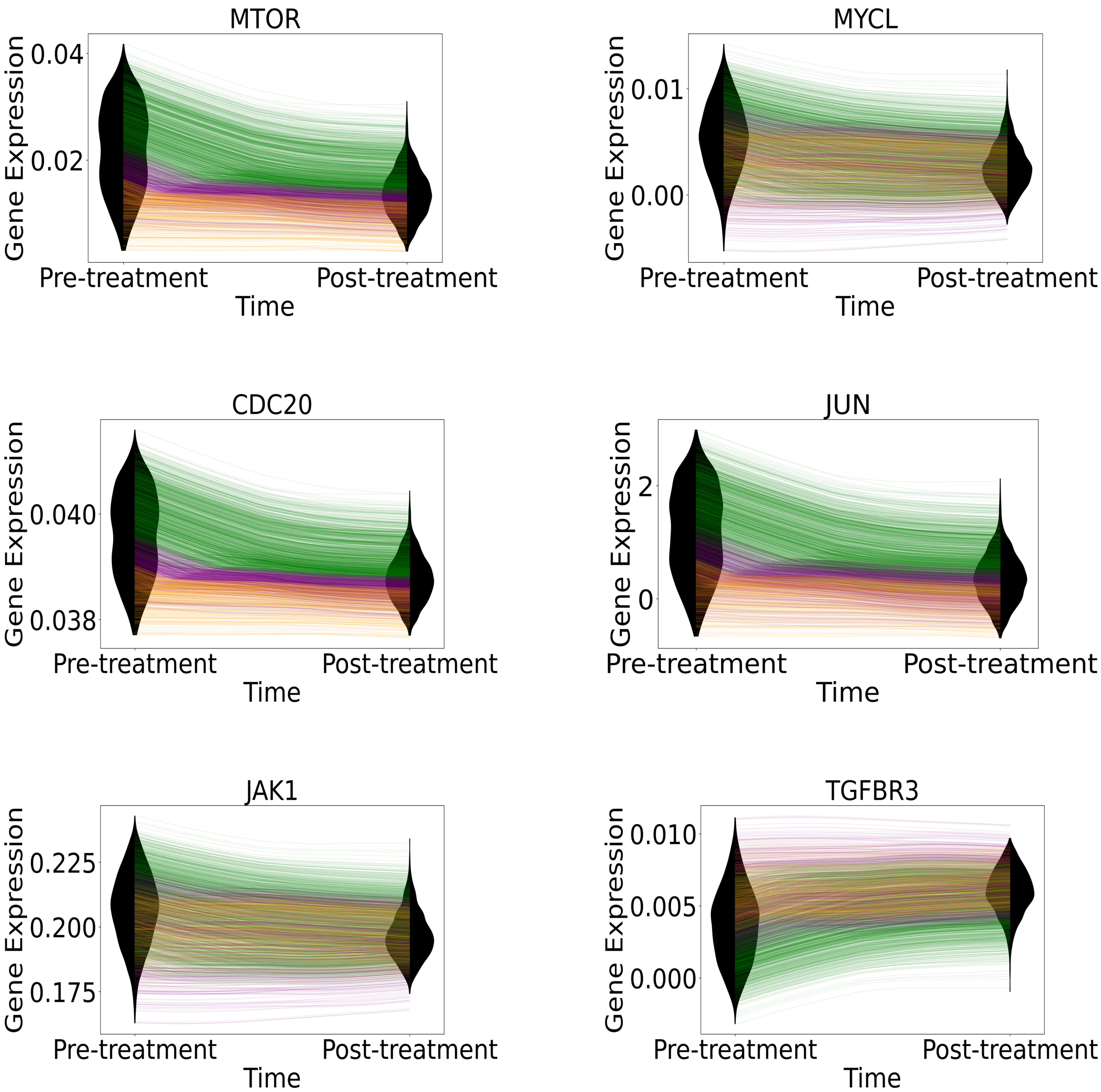
MTOR
MYCL
CDC20
JUN
JAK1
TGFBR3
Gene Expression
Time
Pre-treatment
Post-treatment
0.04
0.02
0.01
0.00
0.040
0.038
2
0
0.225
0.200
0.175
0.010
0.005
0.000

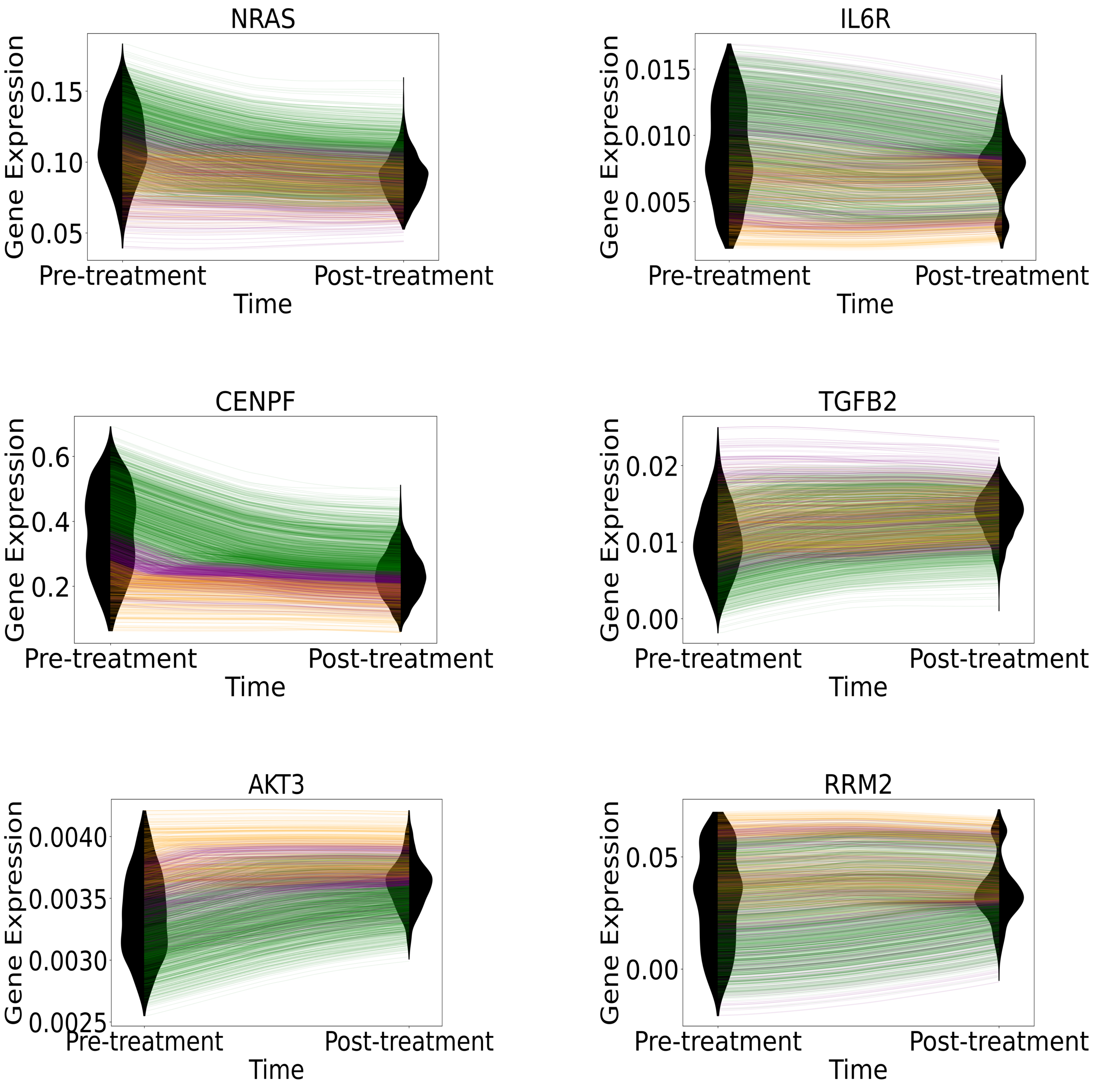
NRAS
Gene Expression
0.15
0.10
0.05
Pre-treatment
Post-treatment
Time
IL6R
Gene Expression
0.015
0.010
0.005
Pre-treatment
Post-treatment
Time
CENPF
Gene Expression
0.6
0.4
0.2
Pre-treatment
Post-treatment
Time
TGFB2
Gene Expression
0.02
0.01
0.00
Pre-treatment
Post-treatment
Time
AKT3
Gene Expression
0.0040
0.0035
0.0030
0.0025
Pre-treatment
Post-treatment
Time
RRM2
Gene Expression
0.05
0.00
Pre-treatment
Post-treatment
Time

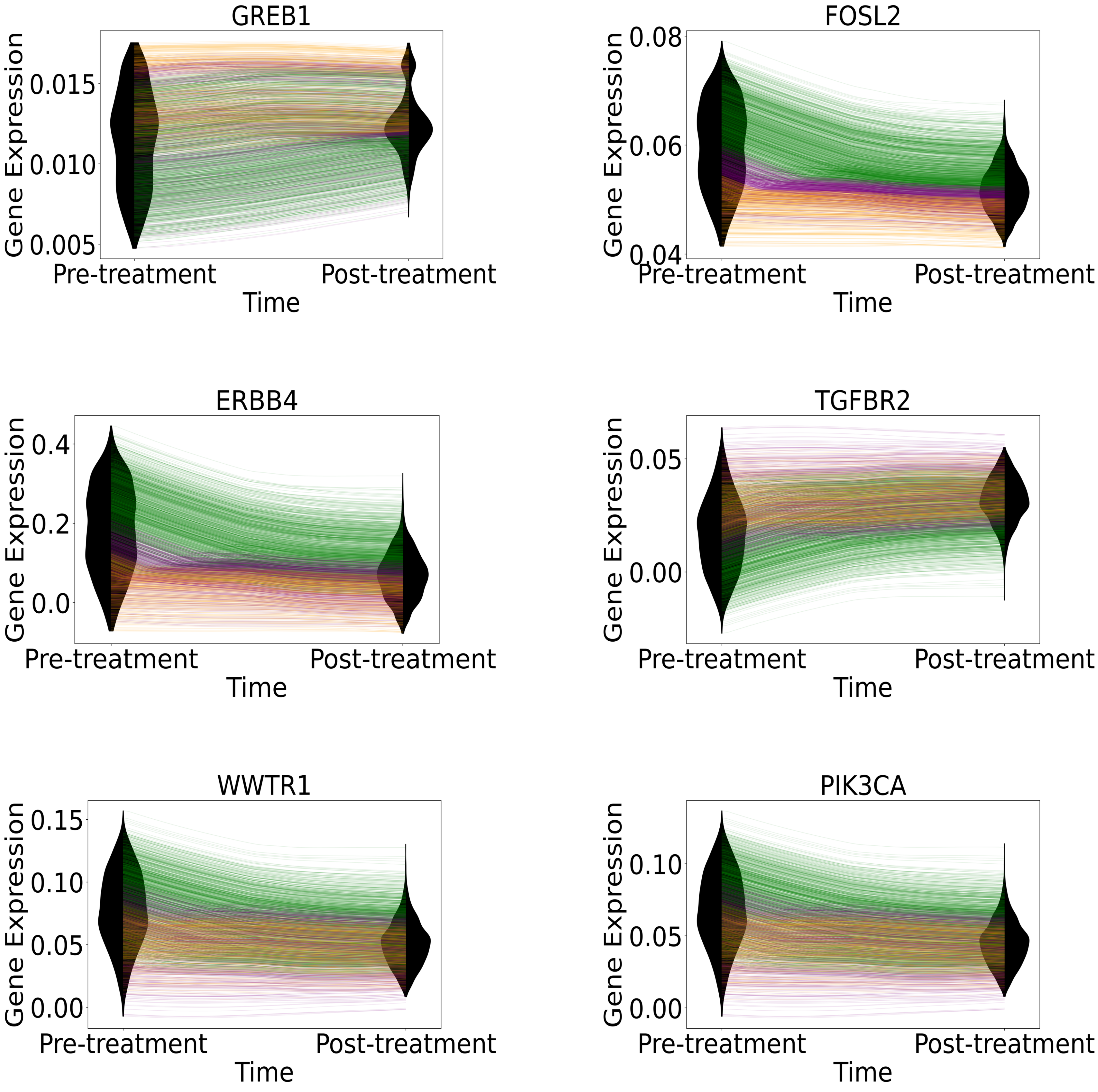

GREB1
Gene Expression
0.015
0.010
0.005
Pre-treatment
Post-treatment
Time
FOSL2
Gene Expression
0.08
0.06
0.04
Pre-treatment
Post-treatment
Time
ERBB4
Gene Expression
0.4
0.2
0.0
Pre-treatment
Post-treatment
Time
TGFBR2
Gene Expression
0.05
0.00
Pre-treatment
Post-treatment
Time
WWTR1
Gene Expression
0.15
0.10
0.05
0.00
Pre-treatment
Post-treatment
Time
PIK3CA
Gene Expression
0.10
0.05
0.00
Pre-treatment
Post-treatment
Time

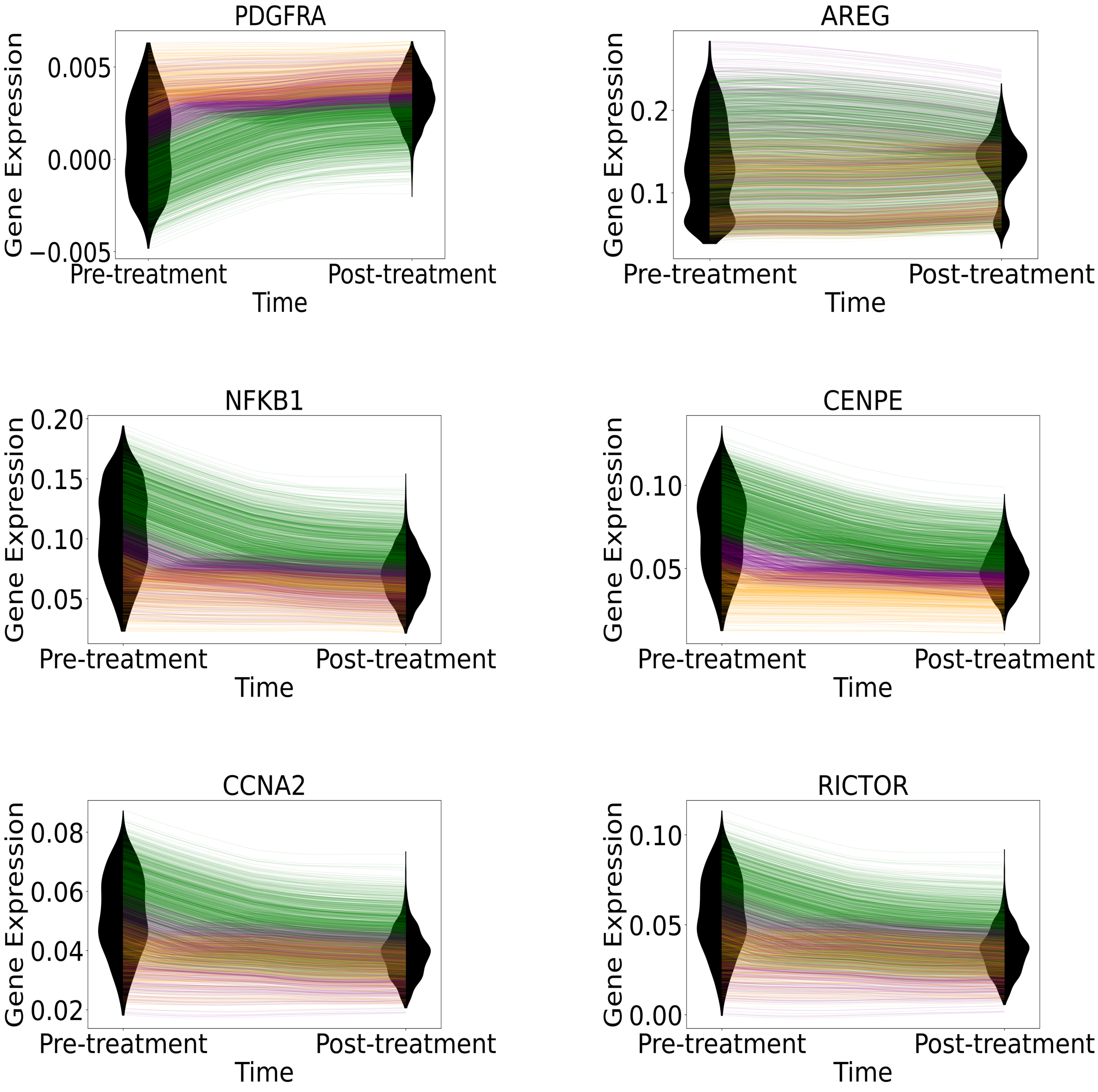
PDGFRA
AREG
NFKB1
CENPE
CCNA2
RICTOR
Gene Expression
Time
Pre-treatment
Post-treatment
0.005
0.000
−0.005
0.2
0.1
0.20
0.15
0.10
0.05
0.08
0.06
0.04
0.02
0.00

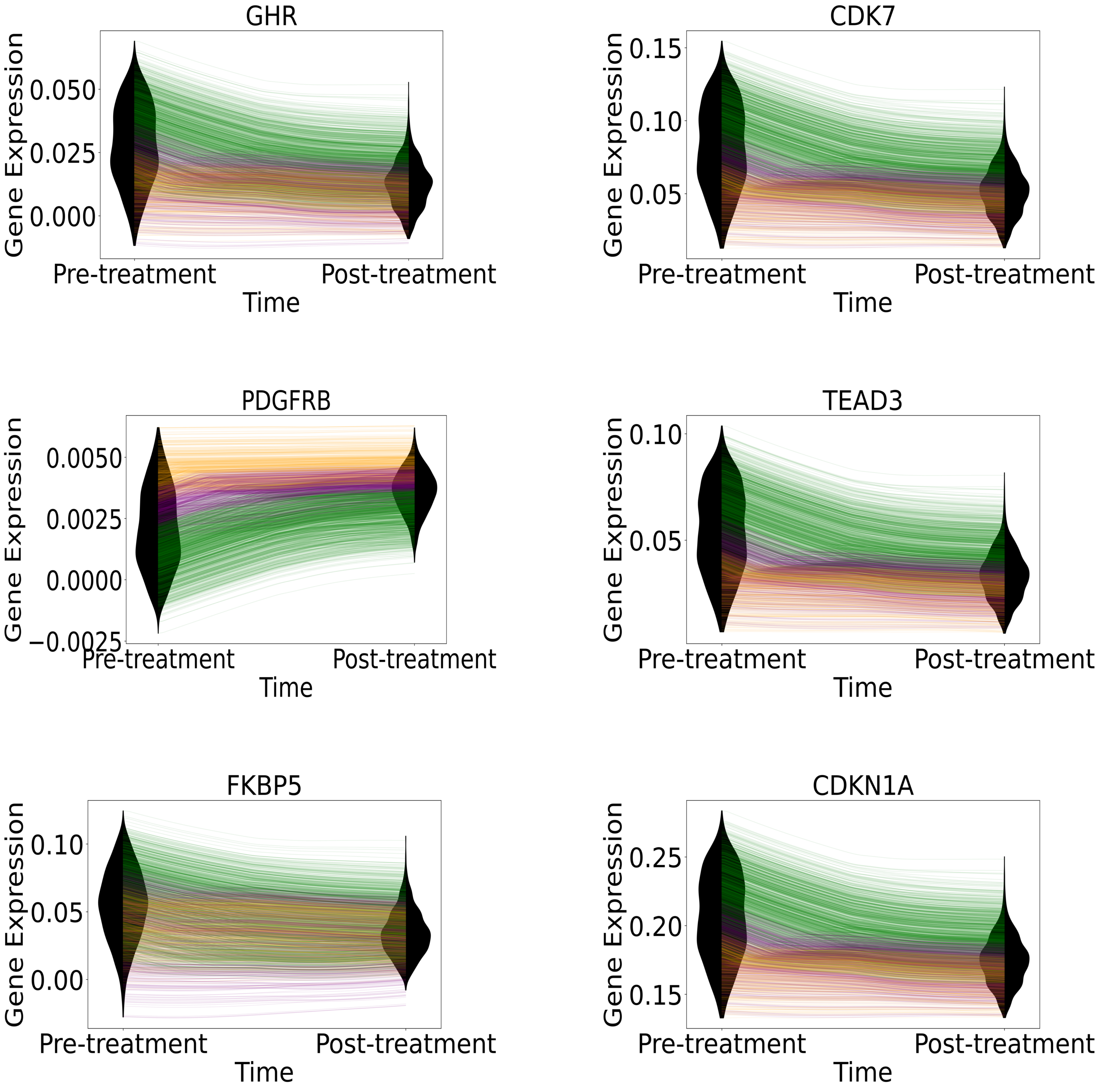
GHR
Gene Expression
0.050
0.025
0.000
Pre-treatment
Post-treatment
Time
CDK7
Gene Expression
0.15
0.10
0.05
Pre-treatment
Post-treatment
Time
PDGFRB
Gene Expression
0.0050
0.0025
0.0000
−0.0025
Pre-treatment
Post-treatment
Time
TEAD3
Gene Expression
0.10
0.05
Pre-treatment
Post-treatment
Time
FKBP5
Gene Expression
0.10
0.05
0.00
Pre-treatment
Post-treatment
Time
CDKN1A
Gene Expression
0.25
0.20
0.15
Pre-treatment
Post-treatment
Time

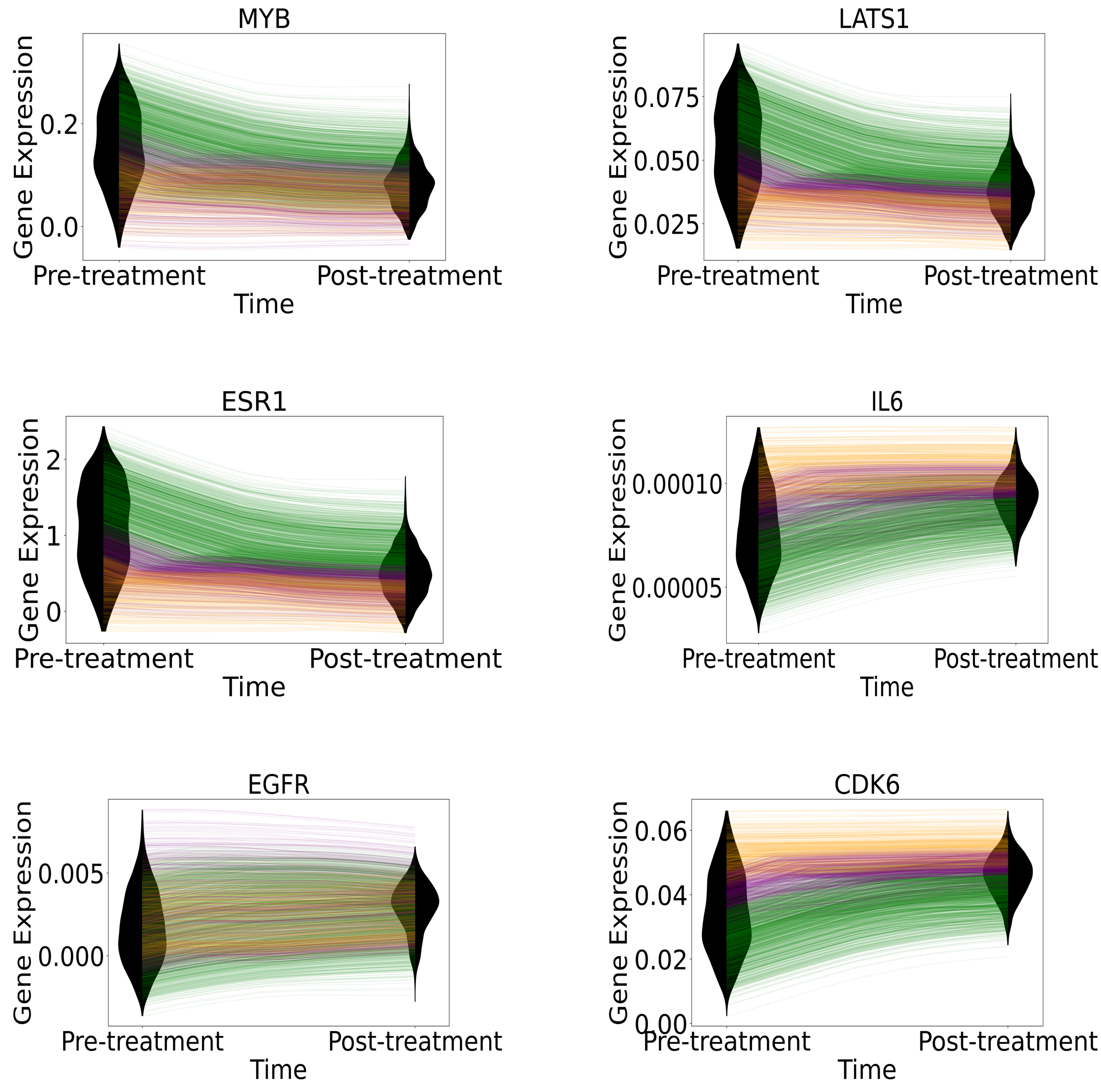
MYB
0.2
0.0
Gene Expression
Pre-treatment
Post-treatment
Time
LATS1
0.075
0.050
0.025
Gene Expression
Pre-treatment
Post-treatment
Time
ESR1
2
1
0
Gene Expression
Pre-treatment
Post-treatment
Time
IL6
0.00010
0.00005
Gene Expression
Pre-treatment
Post-treatment
Time
EGFR
0.005
0.000
Gene Expression
Pre-treatment
Post-treatment
Time
CDK6
0.06
0.04
0.02
0.00
Gene Expression
Pre-treatment
Post-treatment
Time

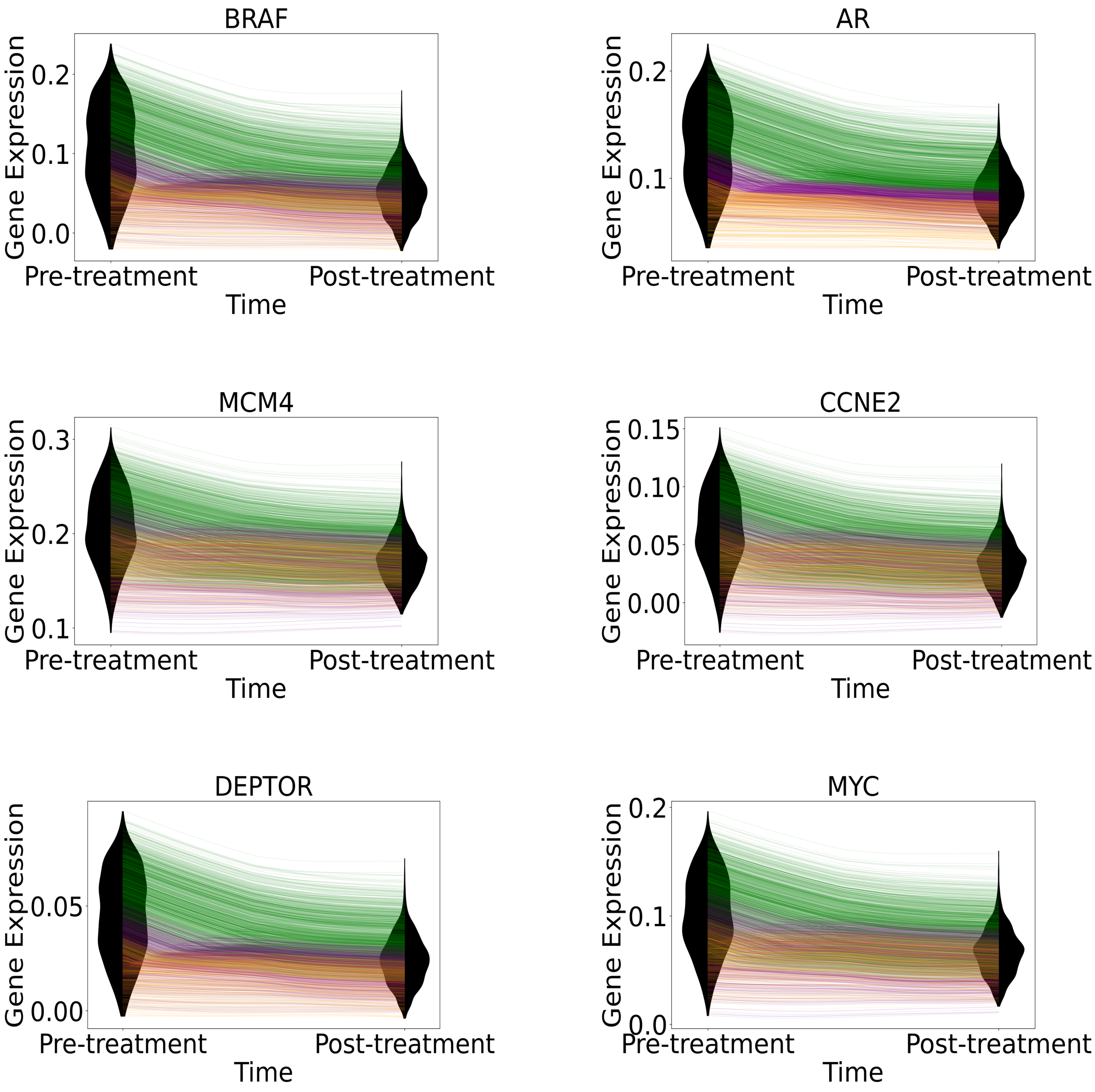
BRAF
AR
MCM4
CCNE2
DEPTOR
MYC
Gene Expression
Time
Pre-treatment
Post-treatment
0.0
0.1
0.2
0.3
0.00
0.05
0.10
0.15

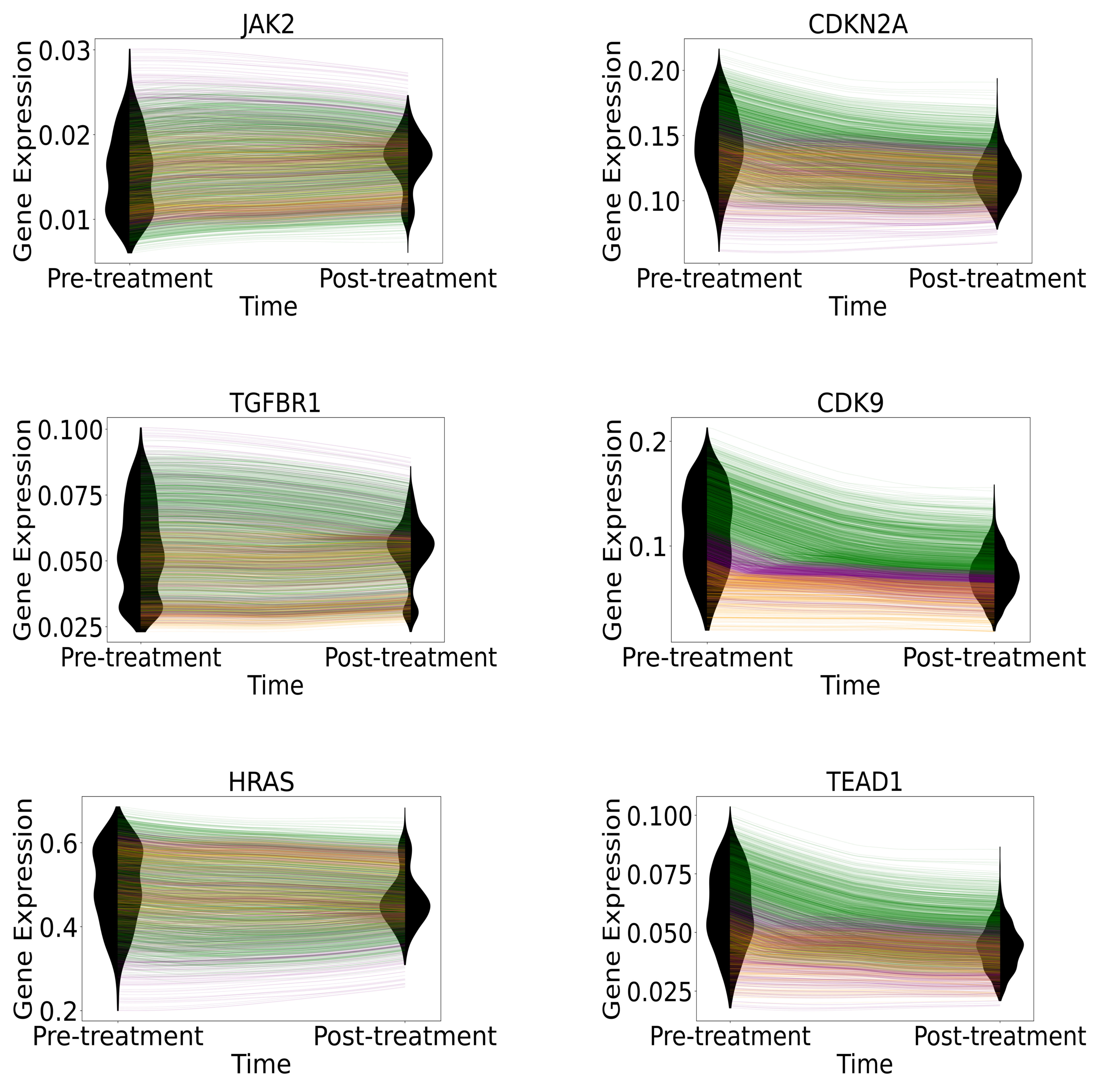
JAK2
Gene Expression
0.03
0.02
0.01
Pre-treatment
Post-treatment
Time
CDKN2A
0.20
0.15
0.10
TGFBR1
0.100
0.075
0.050
0.025
CDK9
0.2
0.1
HRAS
0.6
0.4
0.2
TEAD1
0.100
0.075
0.050
0.025

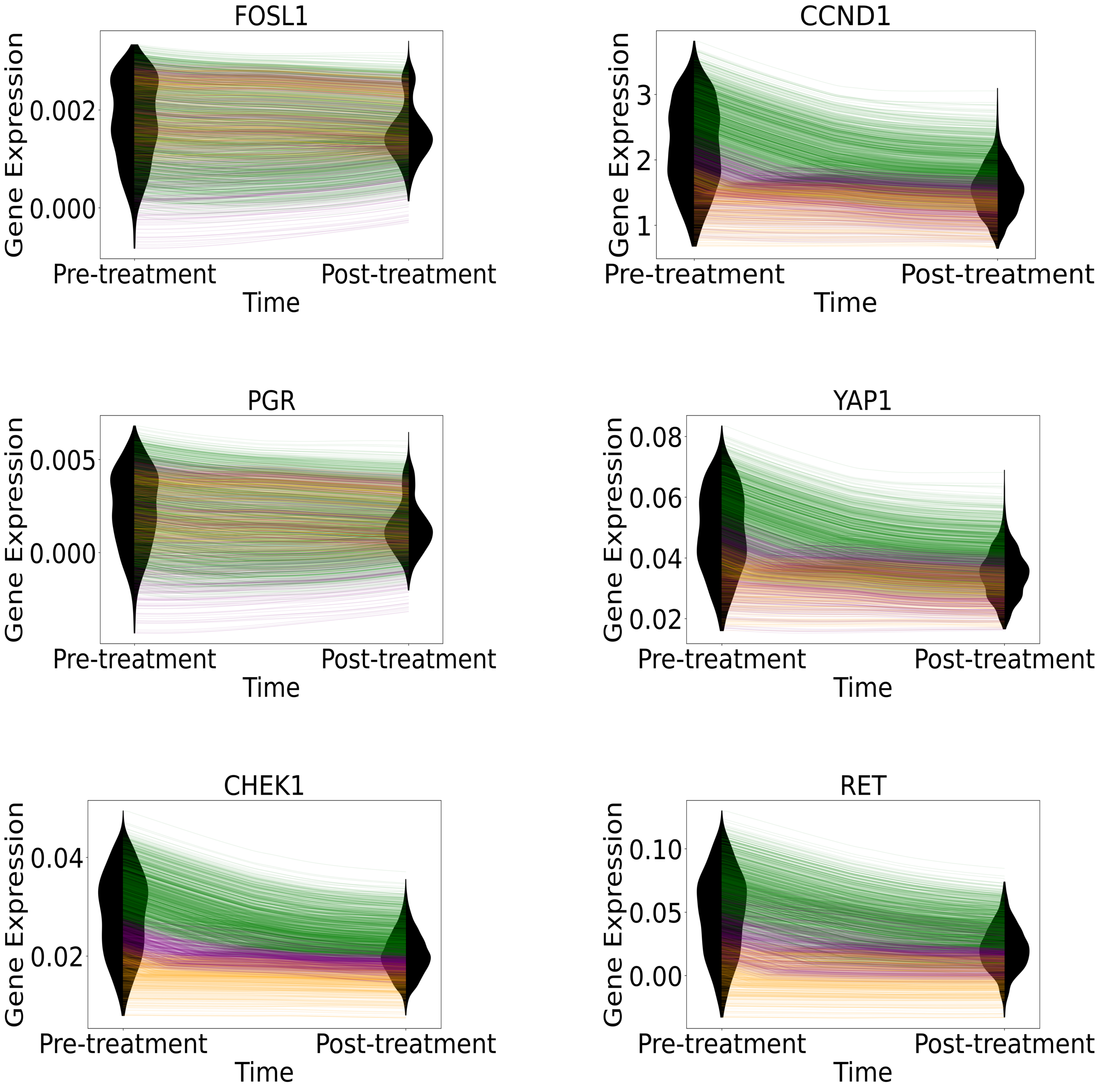
FOSL1
CCND1
PGR
YAP1
CHEK1
RET
Gene Expression
Pre-treatment
Post-treatment
Time
0.002
0.000
3
2
1
0.005
0.000
0.08
0.06
0.04
0.02
0.04
0.02
0.10
0.05
0.00

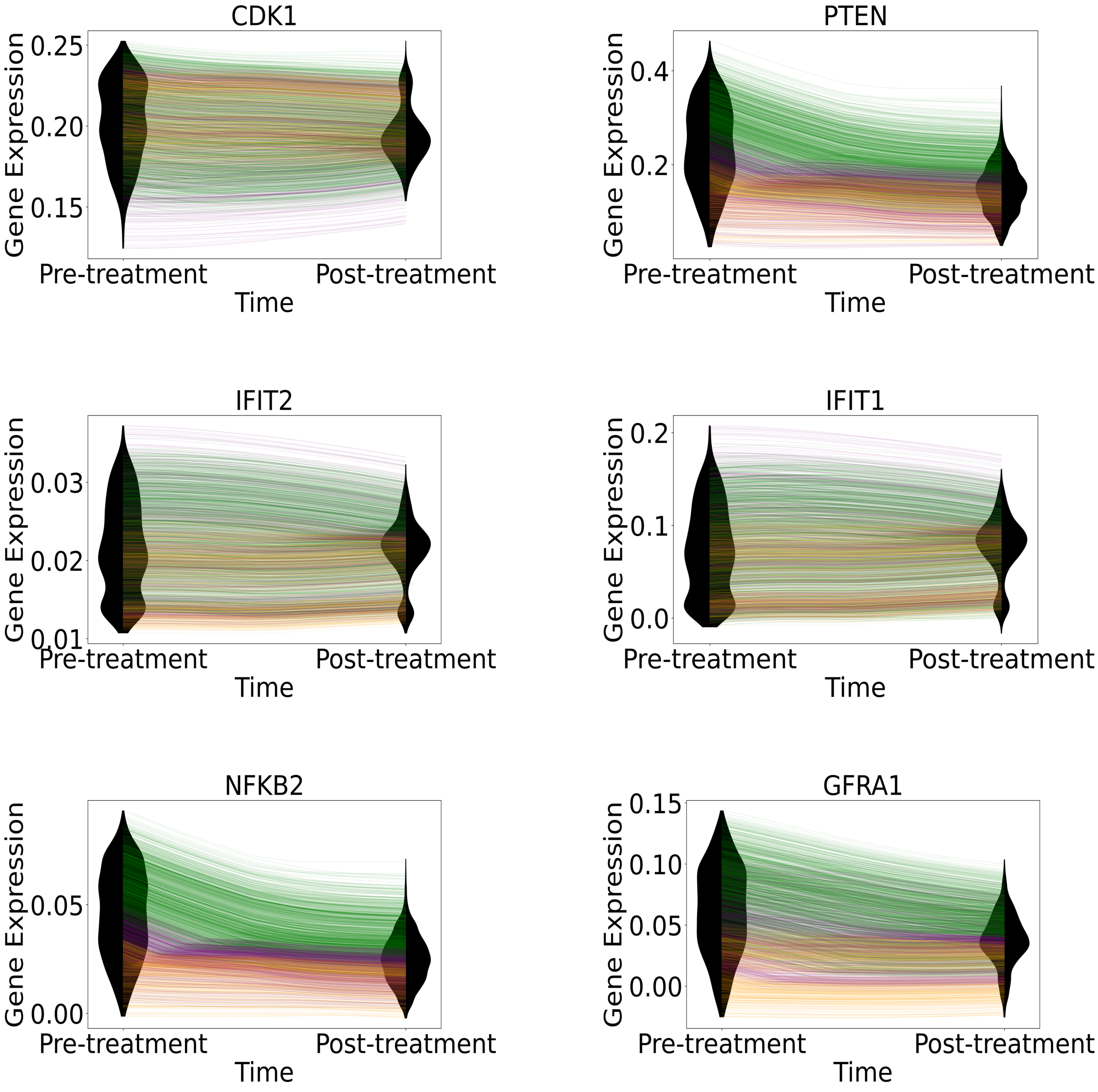
CDK1
PTEN
IFIT2
IFIT1
NFKB2
GFRA1
Gene Expression
Time
Pre-treatment
Post-treatment
0.25
0.20
0.15
0.4
0.2
0.03
0.02
0.01
0.2
0.1
0.0
0.05
0.00
0.15
0.10

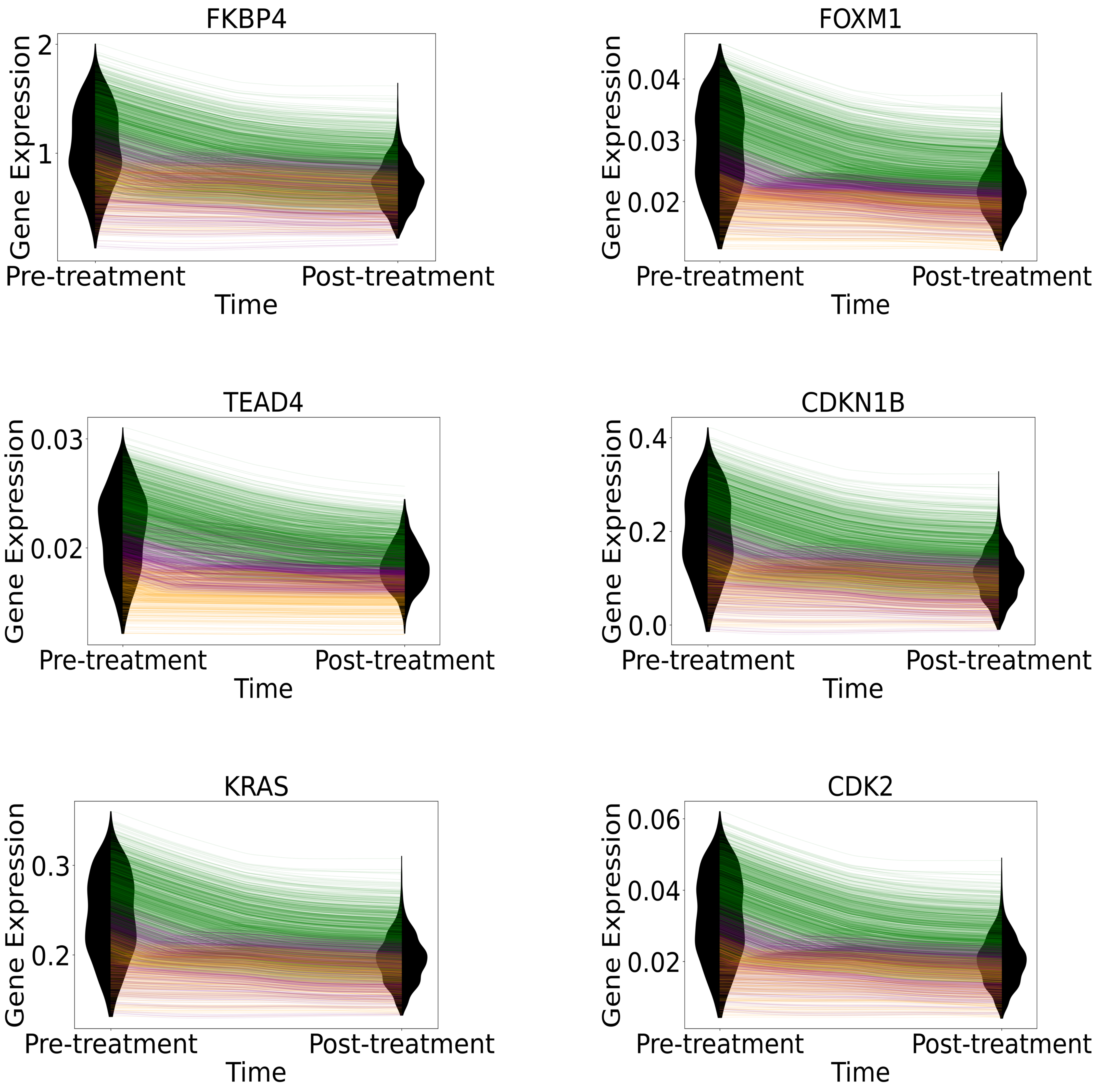

FKBP4
FOXM1
TEAD4
CDKN1B
KRAS
CDK2
Gene Expression
Time
Pre-treatment
Post-treatment

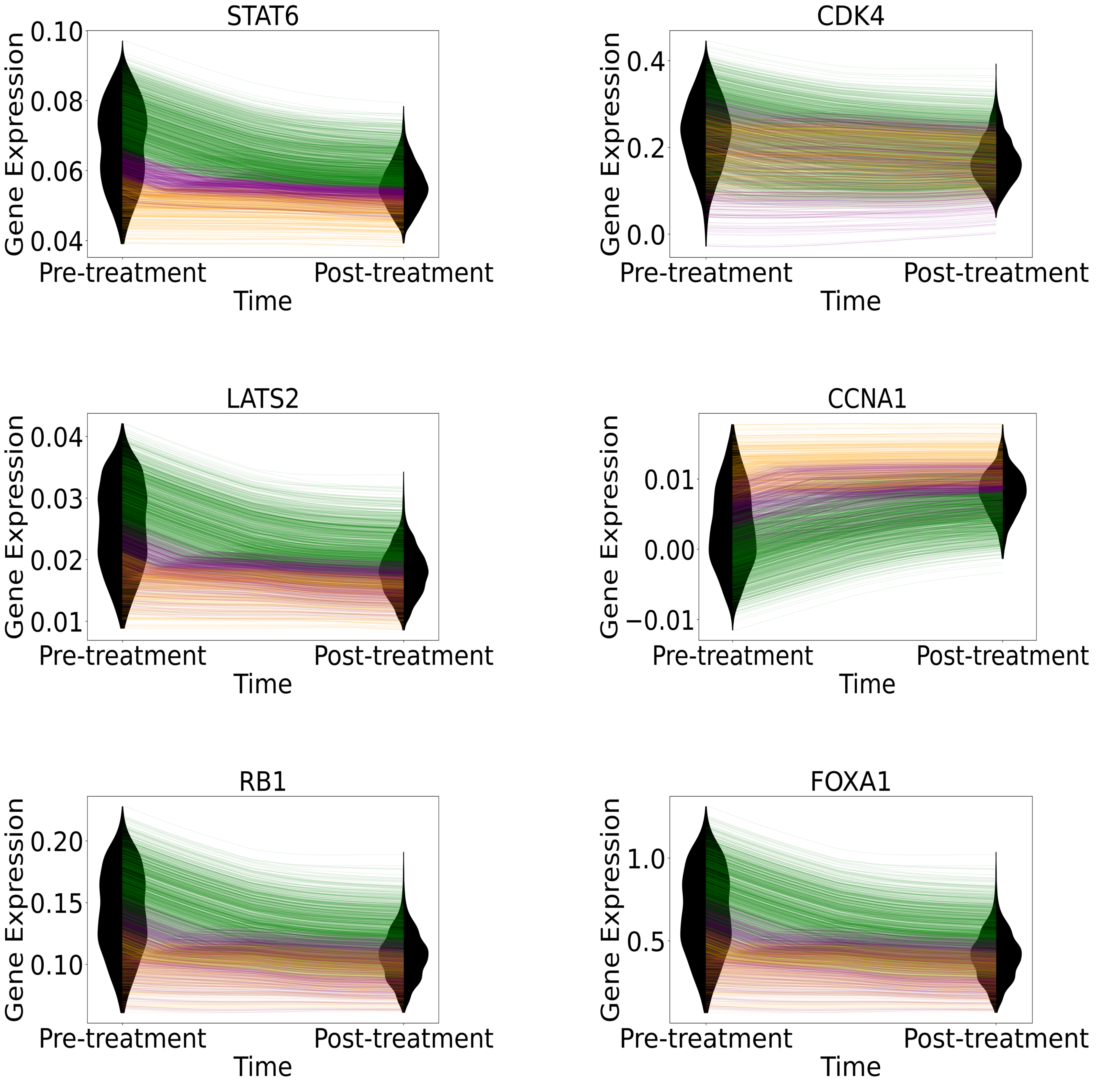
STAT6
0.10
0.08
0.06
0.04
Gene Expression
Pre-treatment
Post-treatment
Time
CDK4
0.4
0.2
0.0
Gene Expression
Pre-treatment
Post-treatment
Time
LATS2
0.04
0.03
0.02
0.01
Gene Expression
Pre-treatment
Post-treatment
Time
CCNA1
0.01
0.00
−0.01
Gene Expression
Pre-treatment
Post-treatment
Time
RB1
0.20
0.15
0.10
Gene Expression
Pre-treatment
Post-treatment
Time
FOXA1
1.0
0.5
Gene Expression
Pre-treatment
Post-treatment
Time

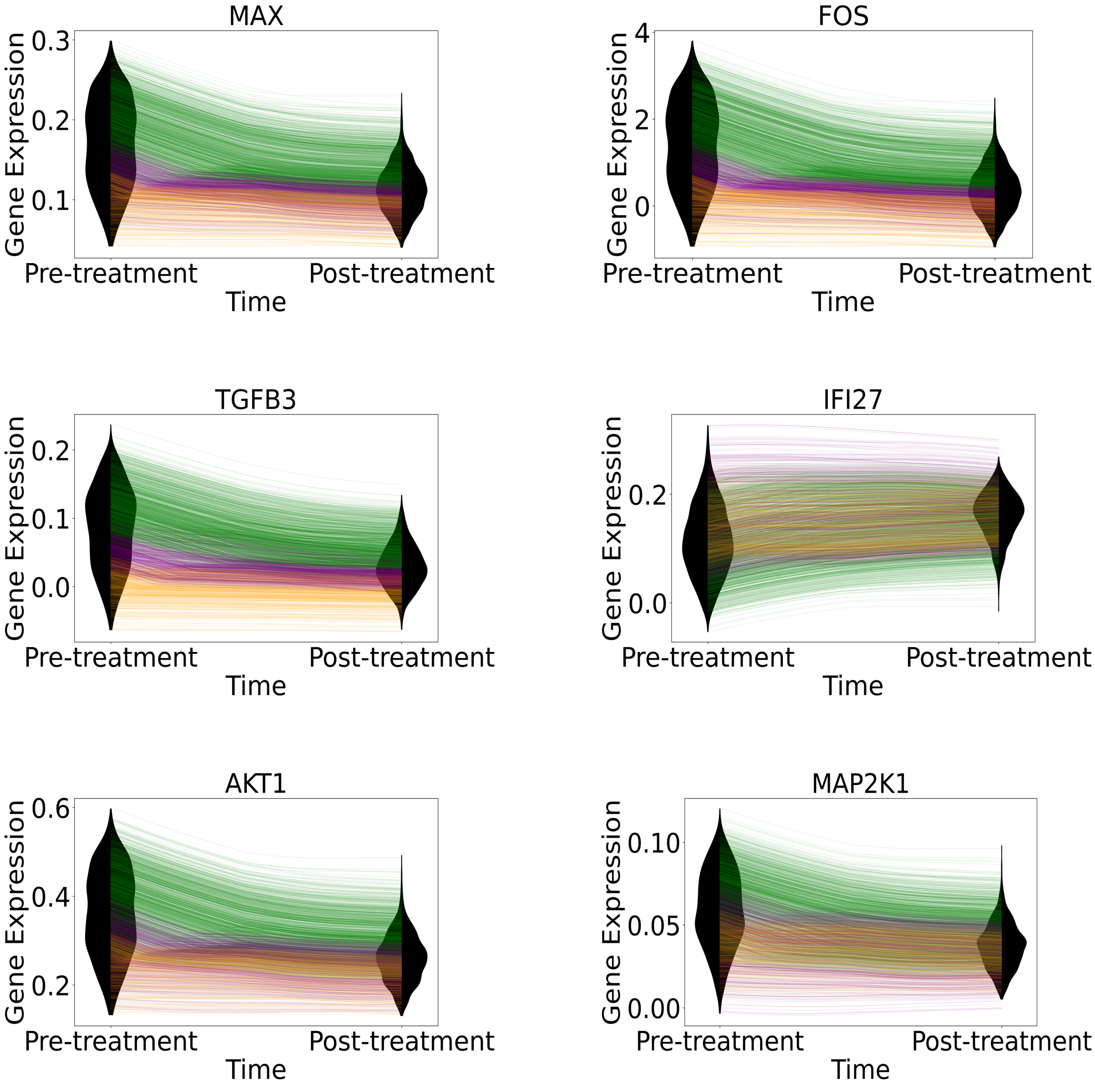

MAX
FOS
TGFB3
IFI27
AKT1
MAP2K1
Gene Expression
Pre-treatment
Post-treatment
Time
0.3
0.2
0.1
4
2
0
0.0
0.6
0.4
0.10
0.05
0.00

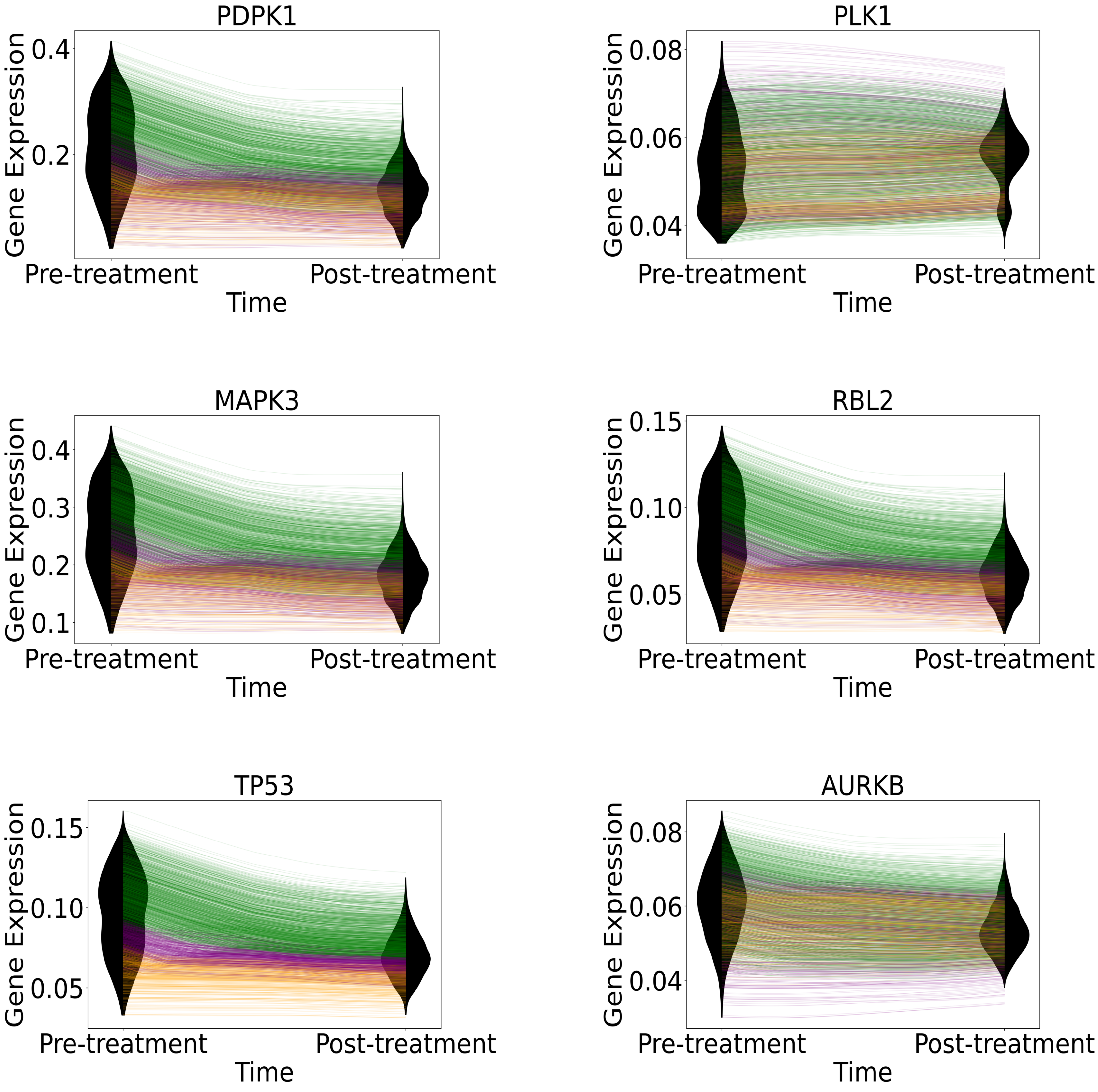
PDPK1
Gene Expression
0.4
0.2
Pre-treatment
Post-treatment
Time
PLK1
Gene Expression
0.08
0.06
0.04
Pre-treatment
Post-treatment
Time
MAPK3
Gene Expression
0.4
0.3
0.2
0.1
Pre-treatment
Post-treatment
Time
RBL2
Gene Expression
0.15
0.10
0.05
Pre-treatment
Post-treatment
Time
TP53
Gene Expression
0.15
0.10
0.05
Pre-treatment
Post-treatment
Time
AURKB
Gene Expression
0.08
0.06
0.04
Pre-treatment
Post-treatment
Time

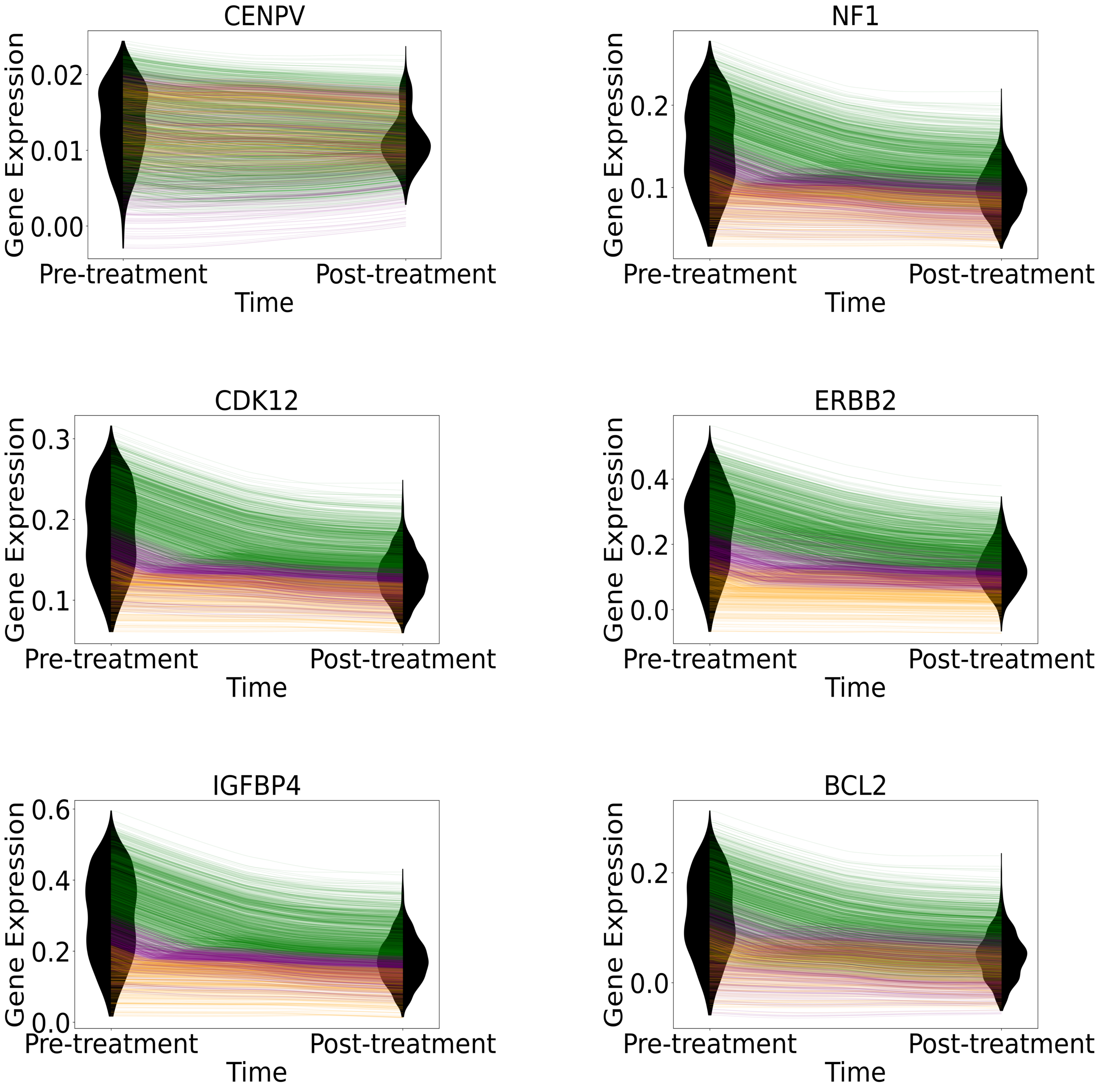
CENPV
Gene Expression
0.00
0.01
0.02
Pre-treatment
Post-treatment
Time
NF1
Gene Expression
0.1
0.2
Pre-treatment
Post-treatment
Time
CDK12
Gene Expression
0.1
0.2
0.3
Pre-treatment
Post-treatment
Time
ERBB2
Gene Expression
0.0
0.2
0.4
Pre-treatment
Post-treatment
Time
IGFBP4
Gene Expression
0.0
0.2
0.4
0.6
Pre-treatment
Post-treatment
Time
BCL2
Gene Expression
0.0
0.2
Pre-treatment
Post-treatment
Time

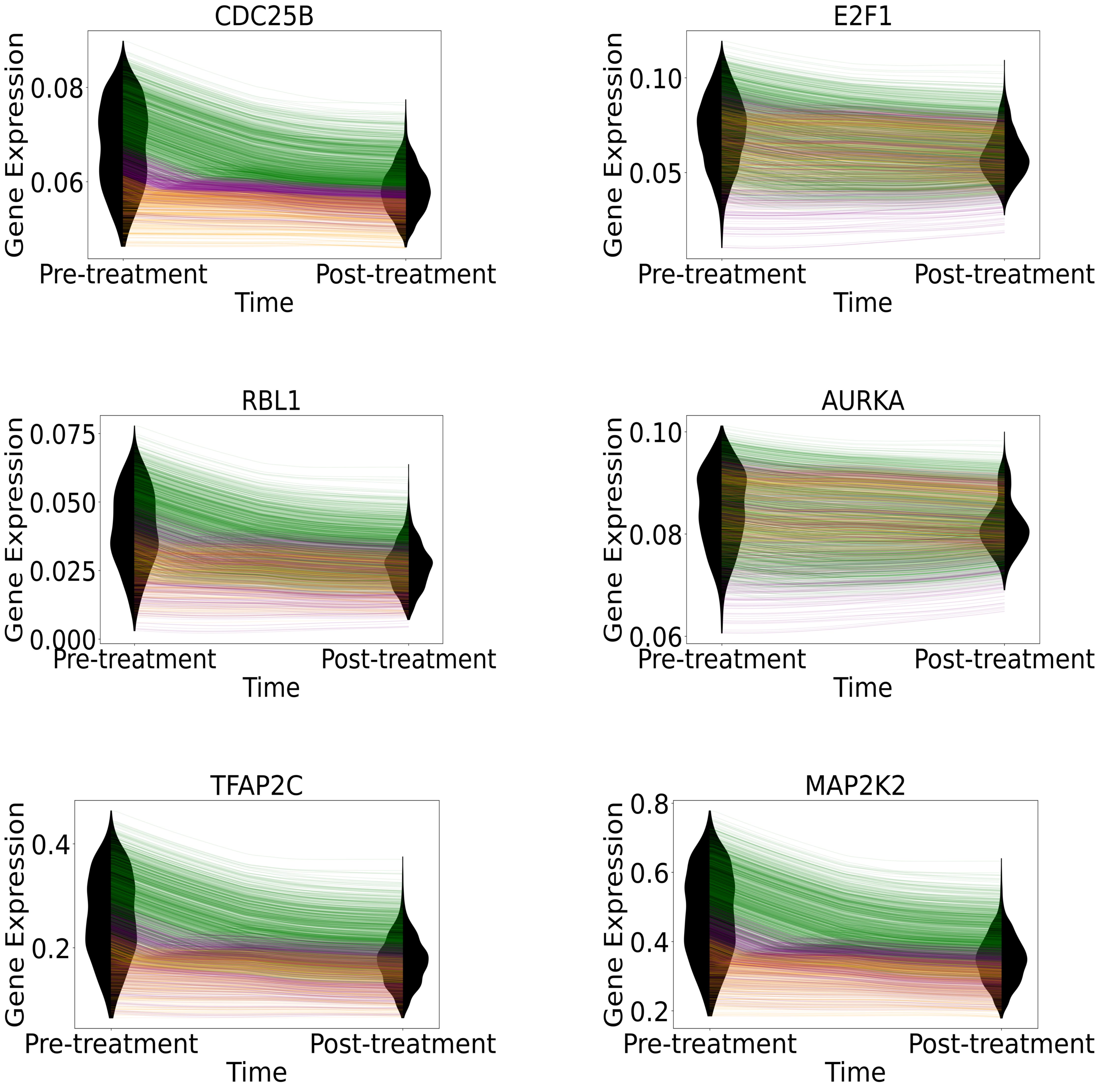

CDC25B
E2F1
RBL1
AURKA
TFAP2C
MAP2K2
Gene Expression
Time
Pre-treatment
Post-treatment
0.08
0.06
0.10
0.05
0.075
0.050
0.025
0.000
0.10
0.08
0.06
0.4
0.2
0.8
0.6
0.4
0.2

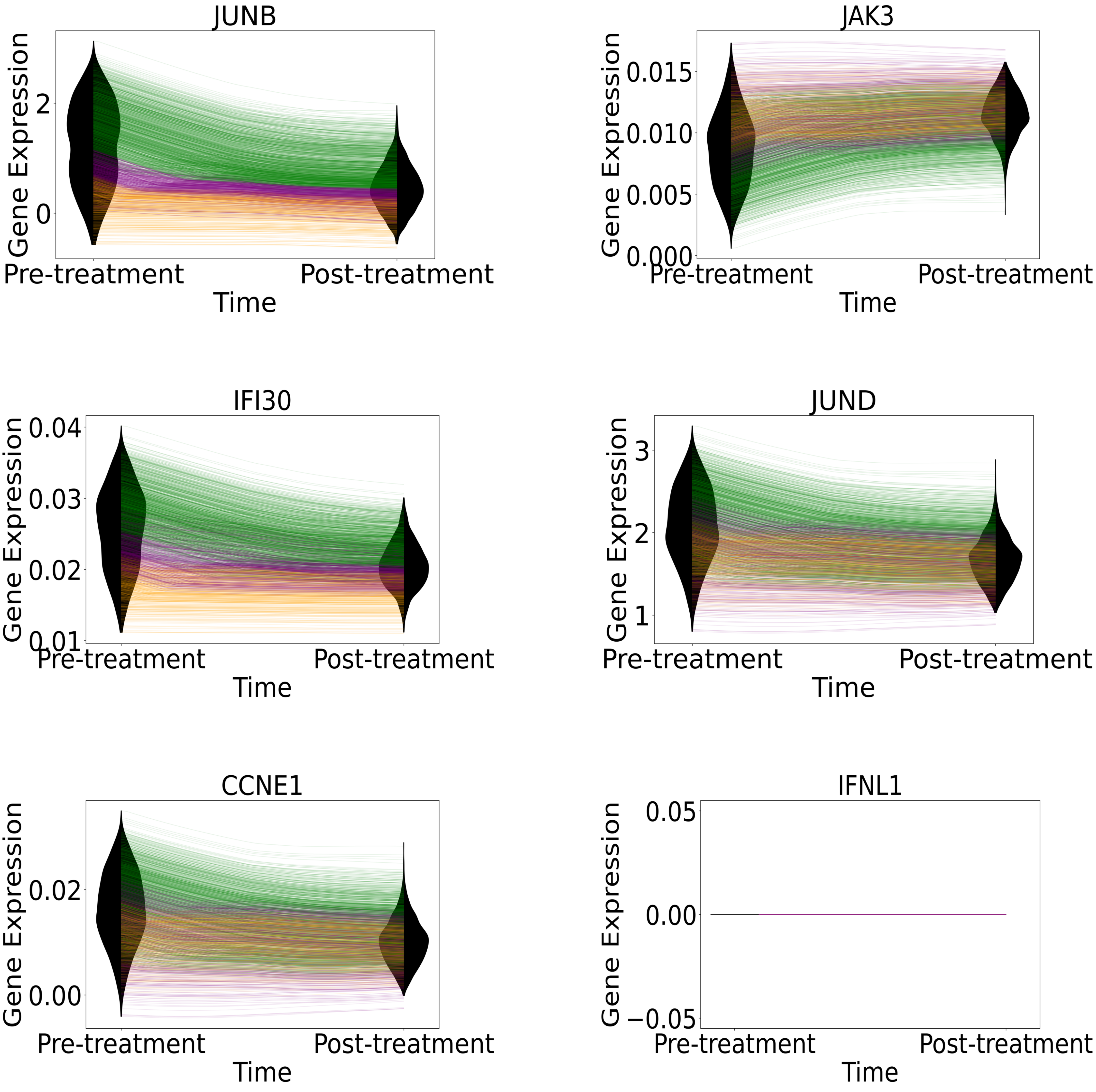
JUNB
Gene Expression
2
0
Pre-treatment
Post-treatment
Time
JAK3
Gene Expression
0.015
0.010
0.005
0.000
Pre-treatment
Post-treatment
Time
IFI30
Gene Expression
0.04
0.03
0.02
0.01
Pre-treatment
Post-treatment
Time
JUND
Gene Expression
3
2
1
Pre-treatment
Post-treatment
Time
CCNE1
Gene Expression
0.02
0.00
Pre-treatment
Post-treatment
Time
IFNL1
Gene Expression
0.05
0.00
−0.05
Pre-treatment
Post-treatment
Time

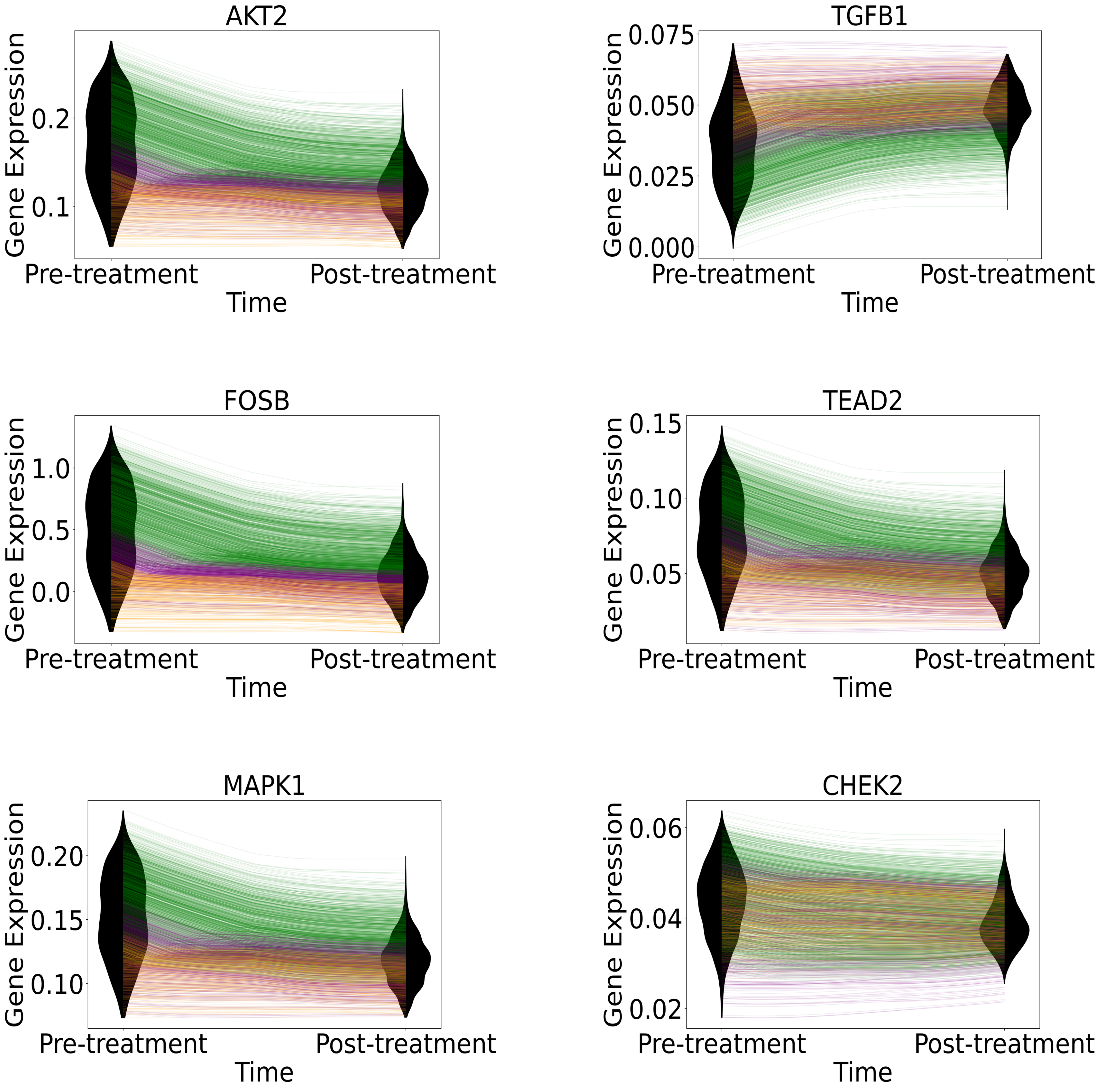

AKT2
TGFB1
FOSB
TEAD2
MAPK1
CHEK2
Gene Expression
Time
Pre-treatment
Post-treatment
0.1
0.2
0.000
0.025
0.050
0.075
0.0
0.5
1.0
0.05
0.10
0.15
0.20
0.02
0.04
0.06

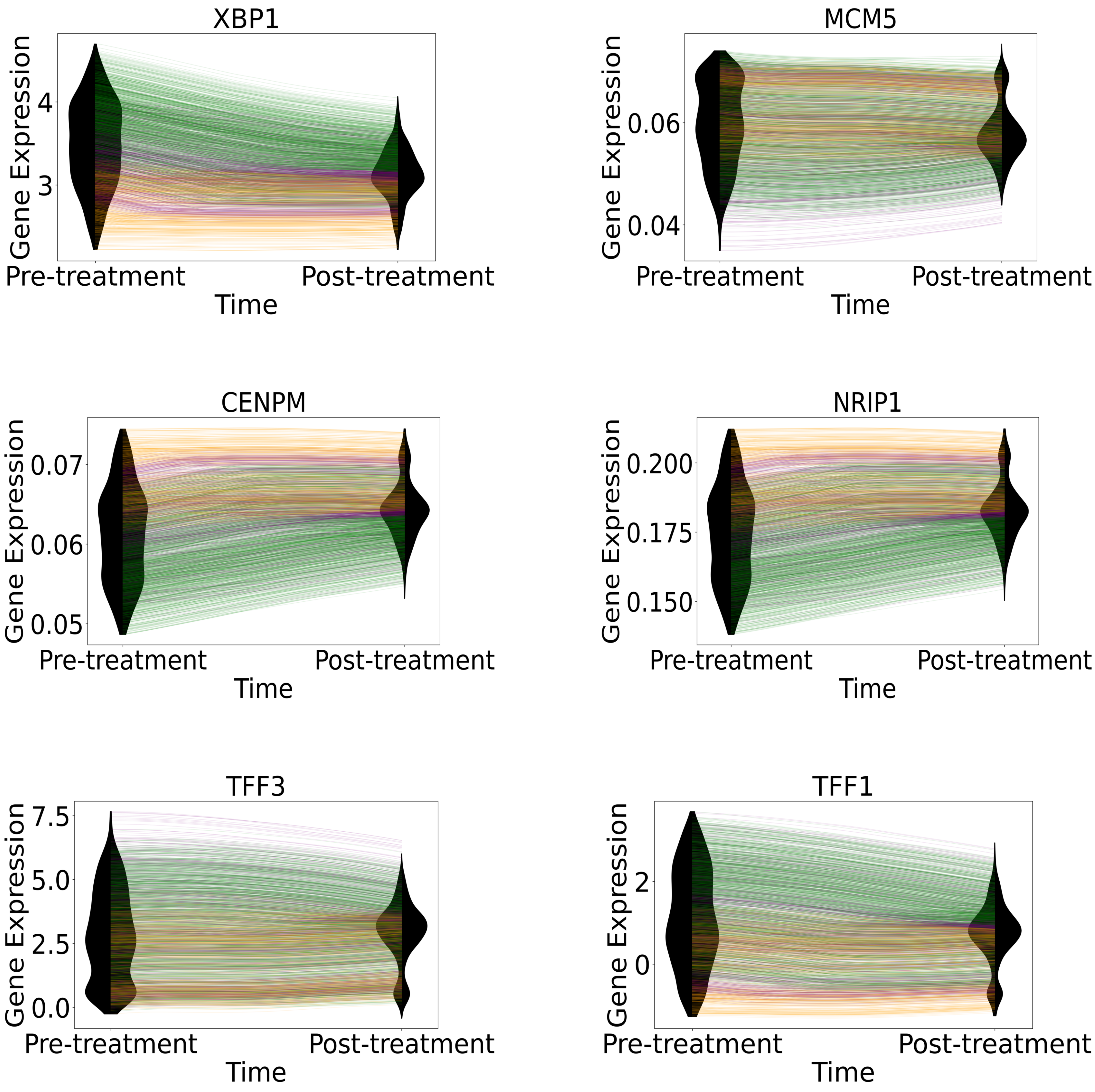

XBP1
Gene Expression
4
3
Pre-treatment
Post-treatment
Time
MCM5
Gene Expression
0.06
0.04
Pre-treatment
Post-treatment
Time
CENPM
Gene Expression
0.07
0.06
0.05
Pre-treatment
Post-treatment
Time
NRIP1
Gene Expression
0.200
0.175
0.150
Pre-treatment
Post-treatment
Time
TFF3
Gene Expression
7.5
5.0
2.5
0.0
Pre-treatment
Post-treatment
Time
TFF1
Gene Expression
2
0
Pre-treatment
Post-treatment
Time

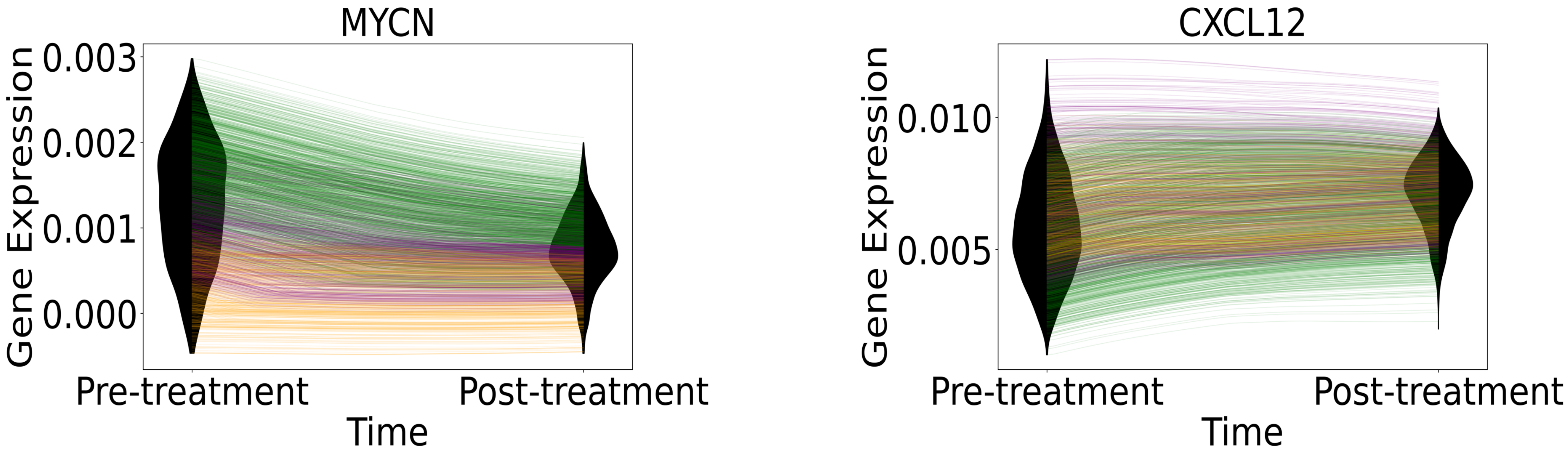

Supplementary Figure 10: Individual cells across three patient datasets. Violin plots show real expression data; colored lines represent predicted trajectories from three subgroups of cells defined by low, medium, and high phenotypic shift levels, as described in Figure 5.
