# Supplementary material for: PROFET Predicts Continuous Gene Expression Dynamics from scRNA-seq Data to Elucidate Heterogeneity of Cancer Treatment Responses
Source: bioRxiv. 2025 Jul 3:2025.06.27.662030. Preprint. [Version 1] doi: 10.1101/2025.06.27.662030 (PMC12236938; doi:10.1101/2025.06.27.662030)
Supplement: Supplement 11 [file media-13.pdf]

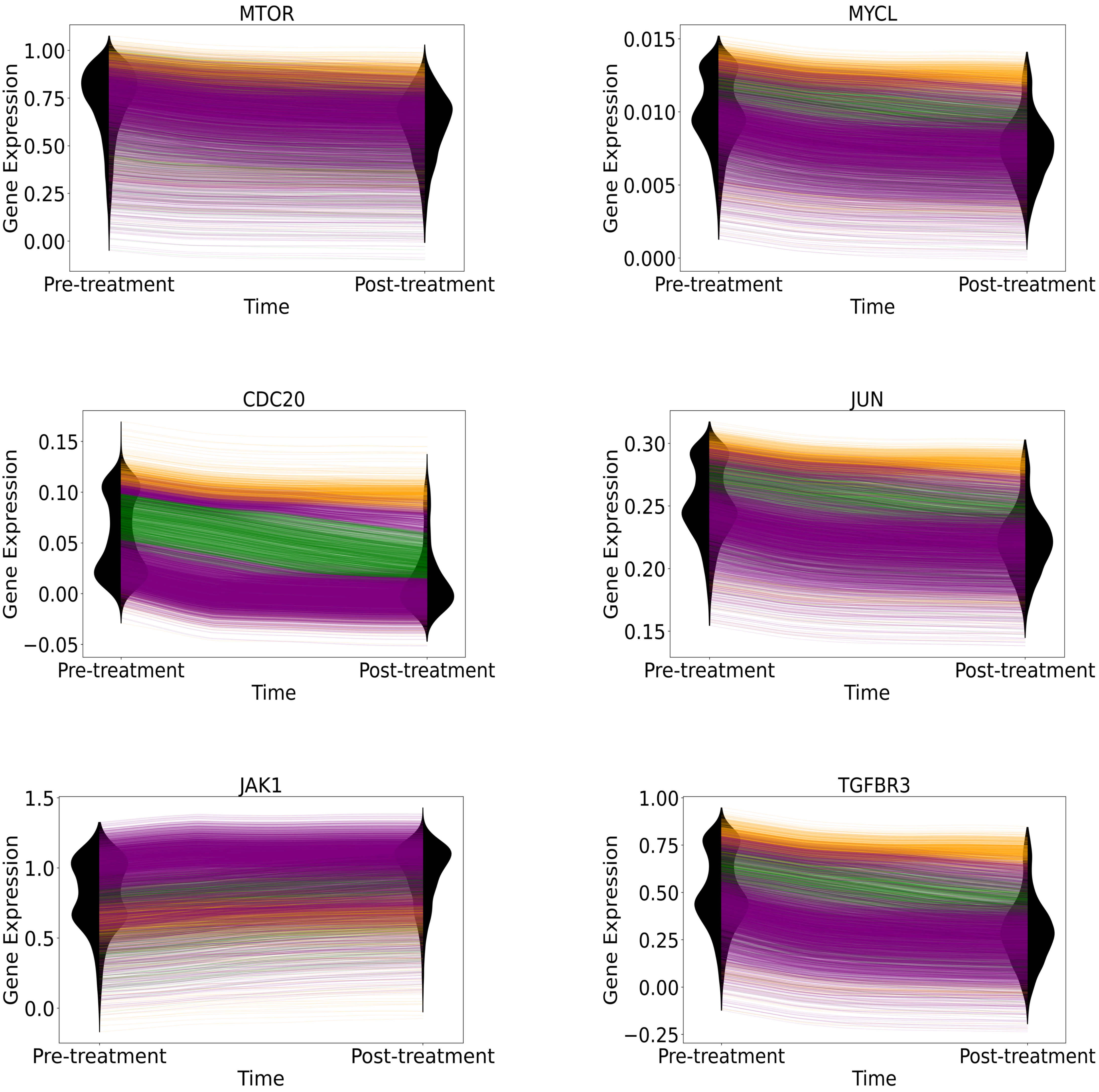
MTOR
Gene Expression
1.00
0.75
0.50
0.25
0.00
Pre-treatment
Post-treatment
Time
MYCL
Gene Expression
0.015
0.010
0.005
0.000
Pre-treatment
Post-treatment
Time
CDC20
Gene Expression
0.15
0.10
0.05
0.00
−0.05
Pre-treatment
Post-treatment
Time
JUN
Gene Expression
0.30
0.25
0.20
0.15
Pre-treatment
Post-treatment
Time
JAK1
Gene Expression
1.5
1.0
0.5
0.0
Pre-treatment
Post-treatment
Time
TGFBR3
Gene Expression
1.00
0.75
0.50
0.25
0.00
−0.25
Pre-treatment
Post-treatment
Time

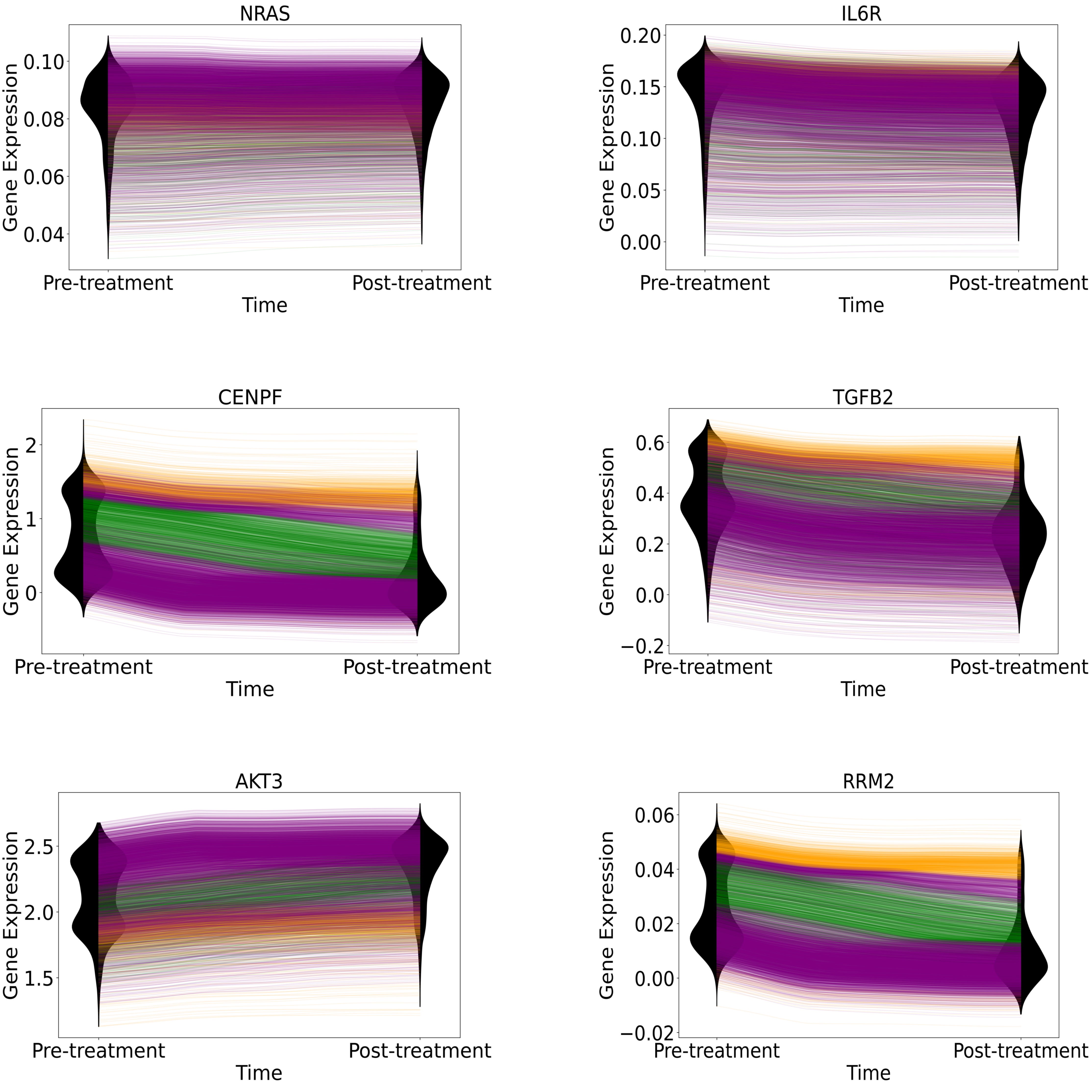
NRAS
Gene Expression
0.10
0.08
0.06
0.04
Pre-treatment
Post-treatment
Time
IL6R
Gene Expression
0.20
0.15
0.10
0.05
0.00
Pre-treatment
Post-treatment
Time
CENPF
Gene Expression
2
1
0
Pre-treatment
Post-treatment
Time
TGFB2
Gene Expression
0.6
0.4
0.2
0.0
−0.2
Pre-treatment
Post-treatment
Time
AKT3
Gene Expression
2.5
2.0
1.5
Pre-treatment
Post-treatment
Time
RRM2
Gene Expression
0.06
0.04
0.02
0.00
−0.02
Pre-treatment
Post-treatment
Time

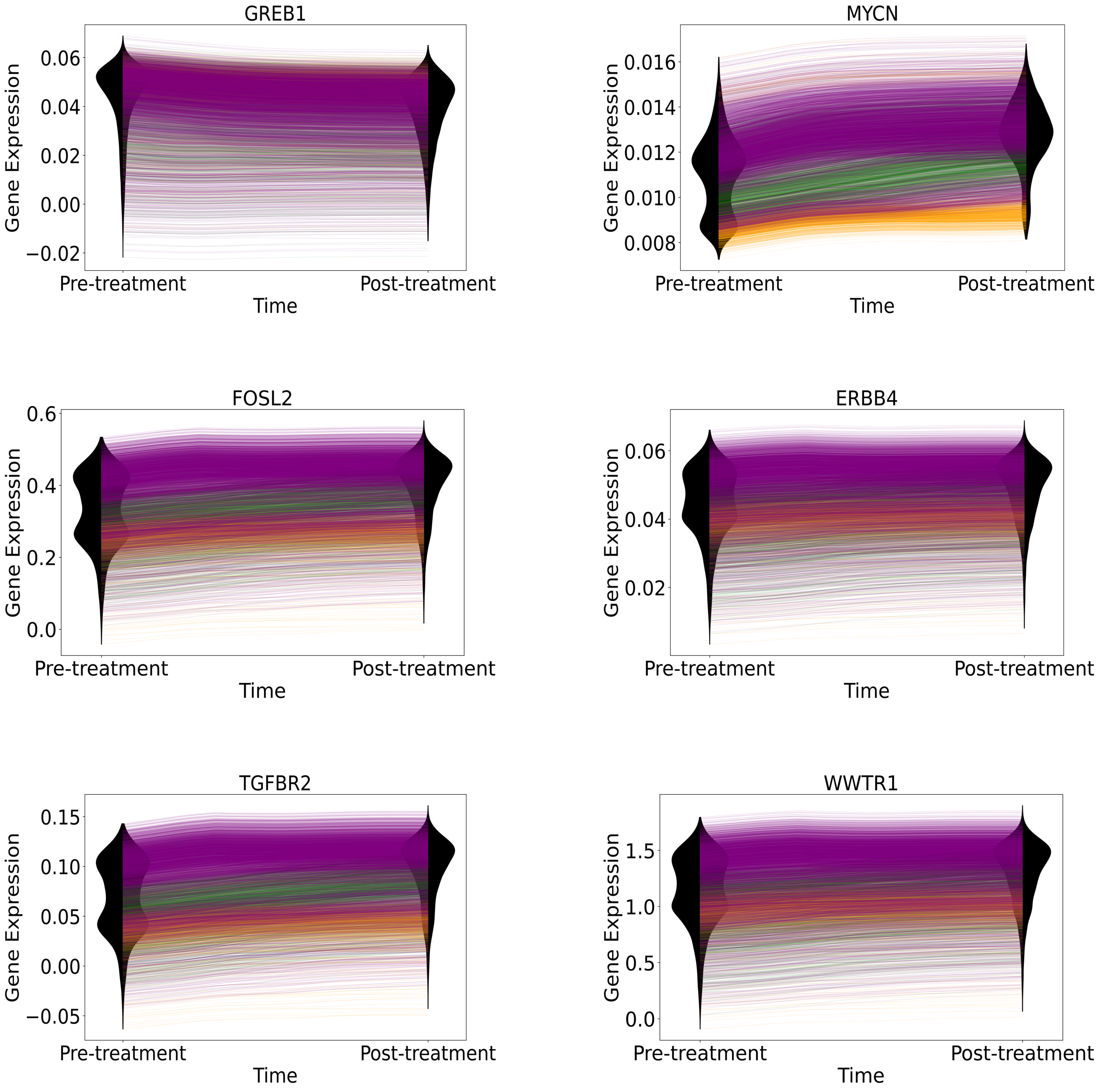

GREB1
MYCN
FOSL2
ERBB4
TGFBR2
WWTR1
Gene Expression
Time
Pre-treatment
Post-treatment
0.06
0.04
0.02
0.00
−0.02
0.016
0.014
0.012
0.010
0.008
0.6
0.4
0.2
0.0
0.15
0.10
0.05
−0.05
1.5
1.0
0.5

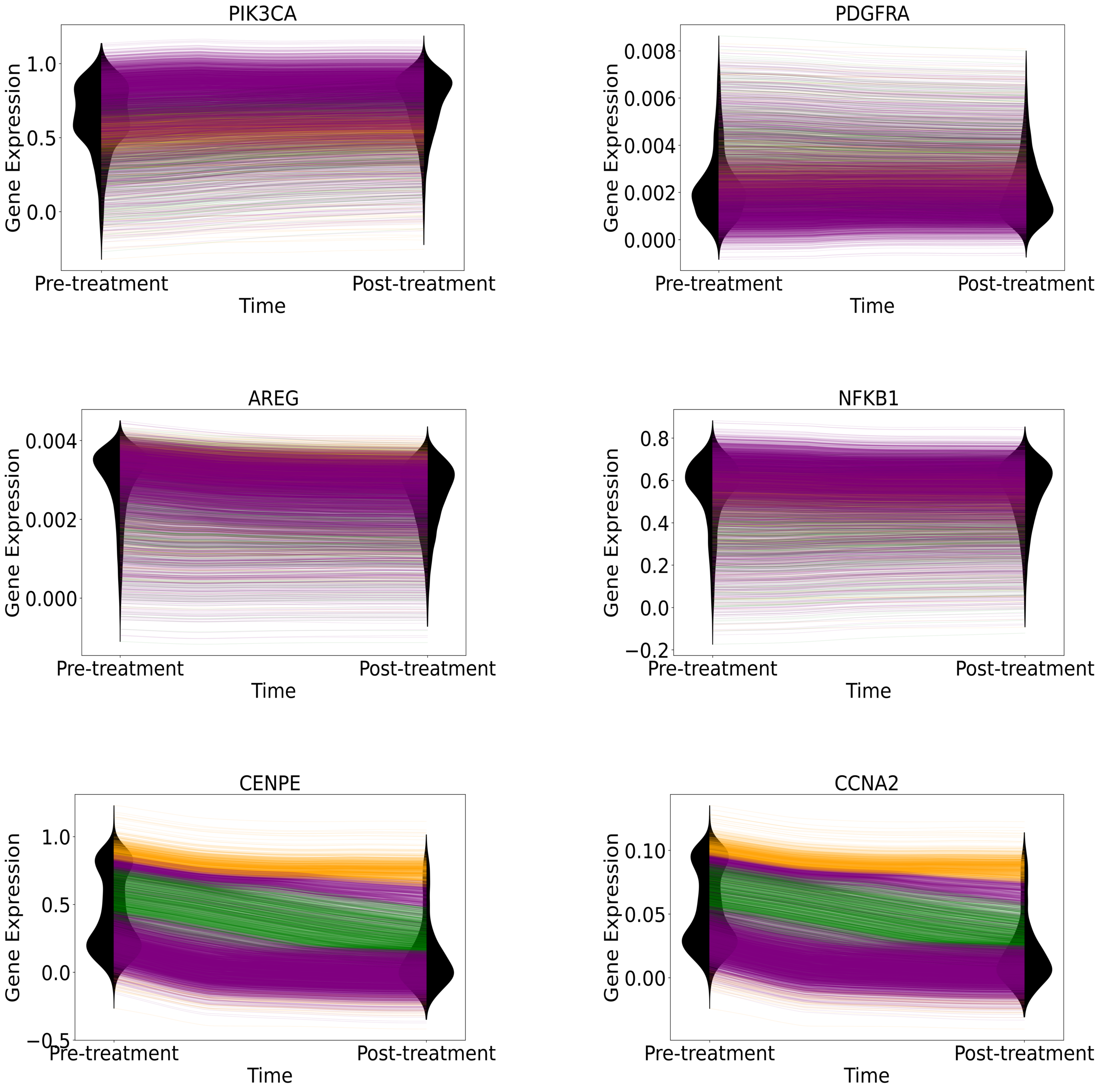

PIK3CA
PDGFRA
AREG
NFKB1
CENPE
CCNA2
Gene Expression
Time
Pre-treatment
Post-treatment
1.0
0.5
0.0
0.008
0.006
0.004
0.002
0.000
0.8
0.6
0.4
0.2
−0.2
−0.5
0.10
0.05
0.00

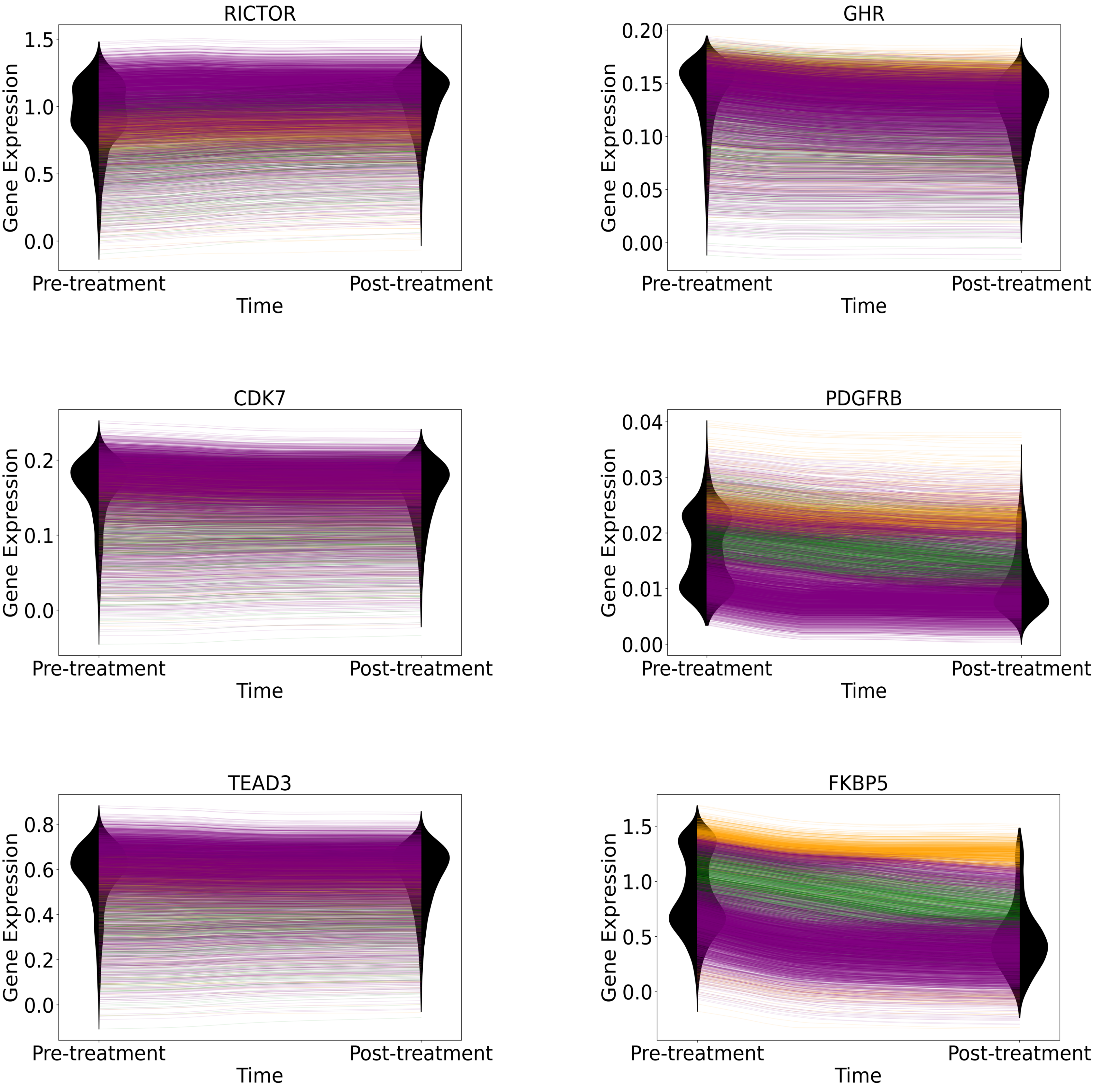

RICTOR
GHR
CDK7
PDGFRB
TEAD3
FKBP5
Gene Expression
Time
Pre-treatment
Post-treatment

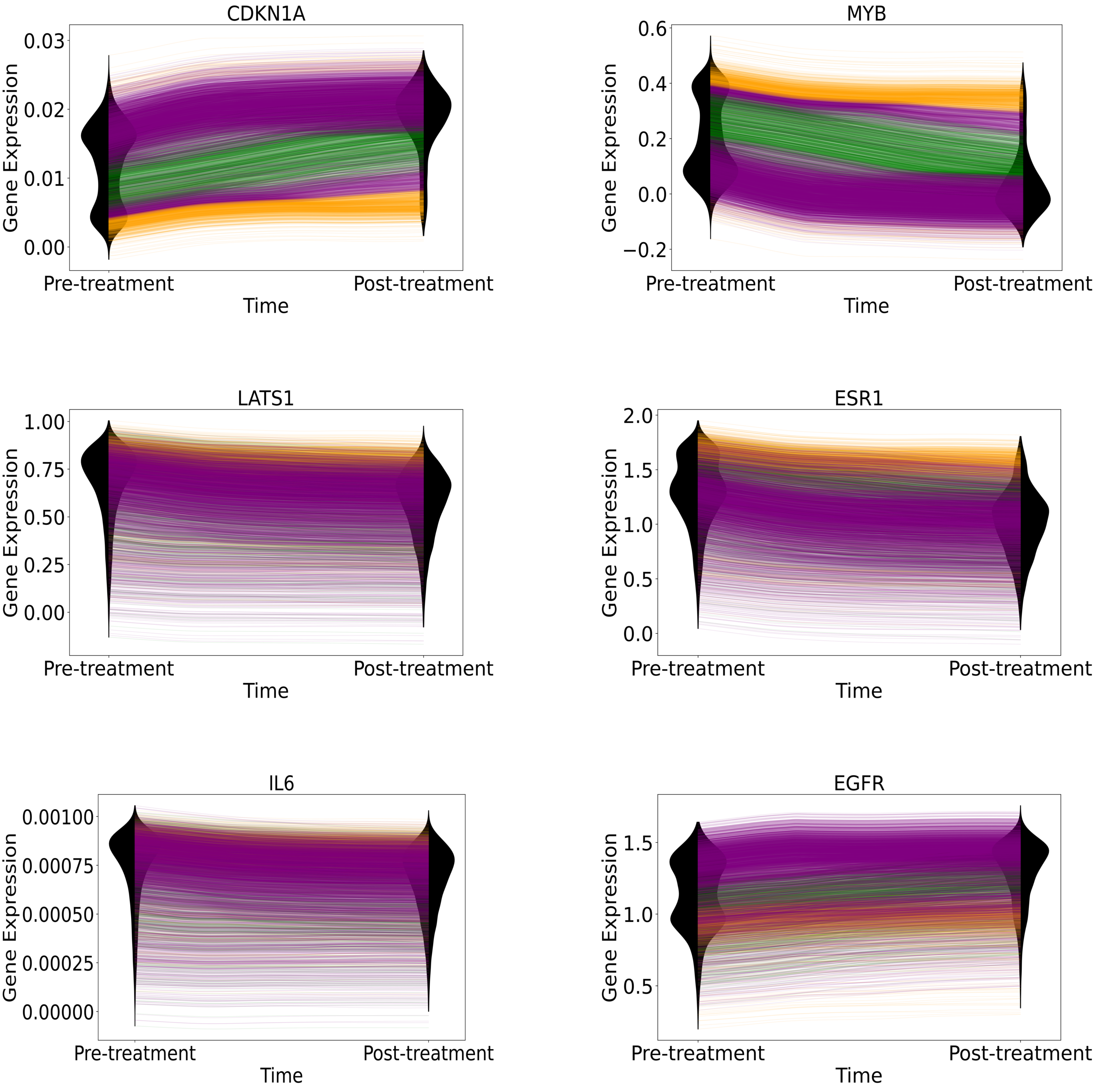

CDKN1A
MYB
LATS1
ESR1
IL6
EGFR
Gene Expression
Time
Pre-treatment
Post-treatment

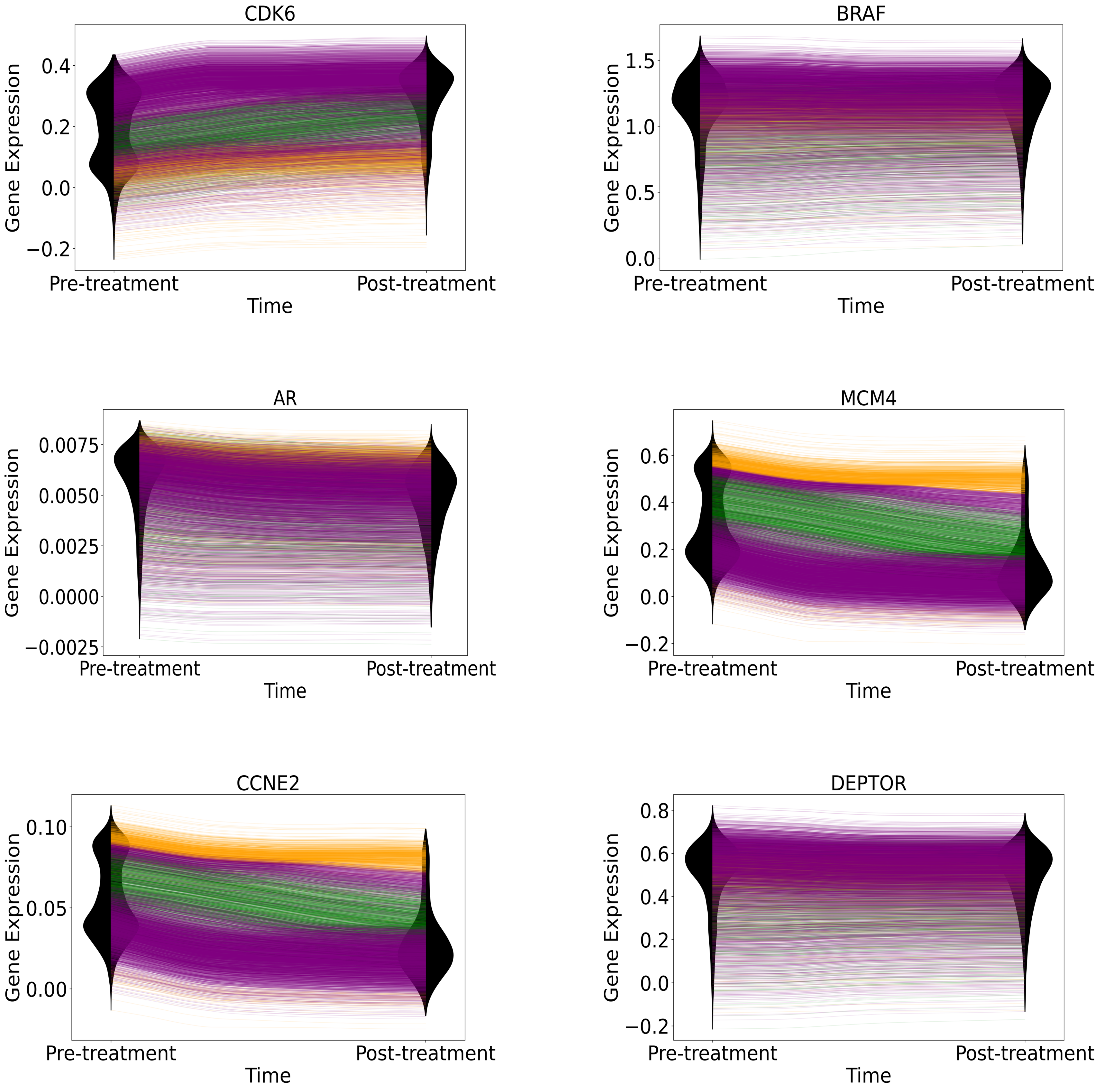

CDK6
BRAF
AR
MCM4
CCNE2
DEPTOR
Gene Expression
Time
Pre-treatment
Post-treatment

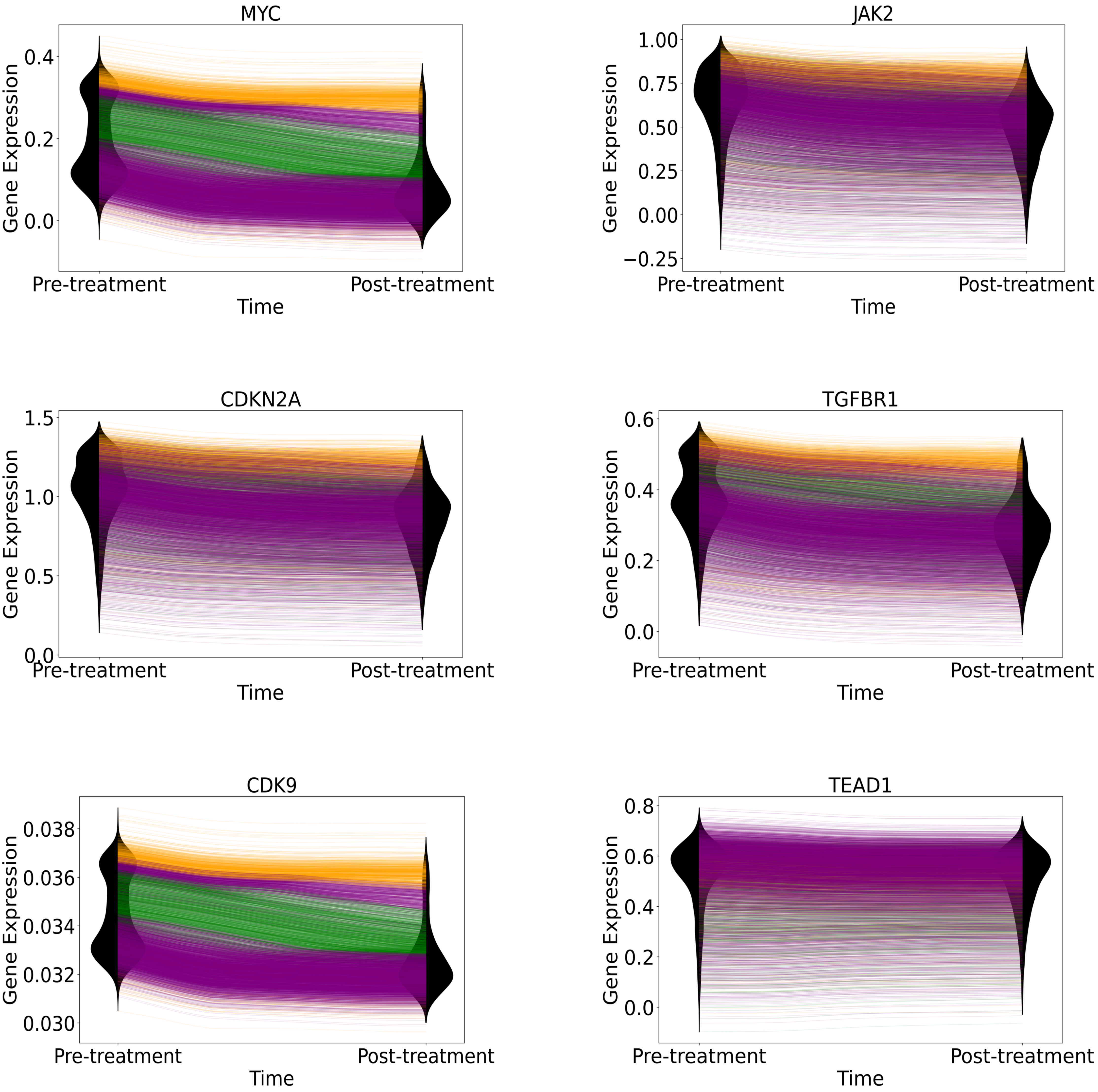

MYC
JAK2
CDKN2A
TGFBR1
CDK9
TEAD1
Gene Expression
Time
Pre-treatment
Post-treatment
0.4
0.2
0.0
1.00
0.75
0.50
0.25
0.00
−0.25
1.5
1.0
0.5
0.0
0.6
0.4
0.2
0.0
0.038
0.036
0.034
0.032
0.030
0.8
0.6
0.4
0.2
0.0

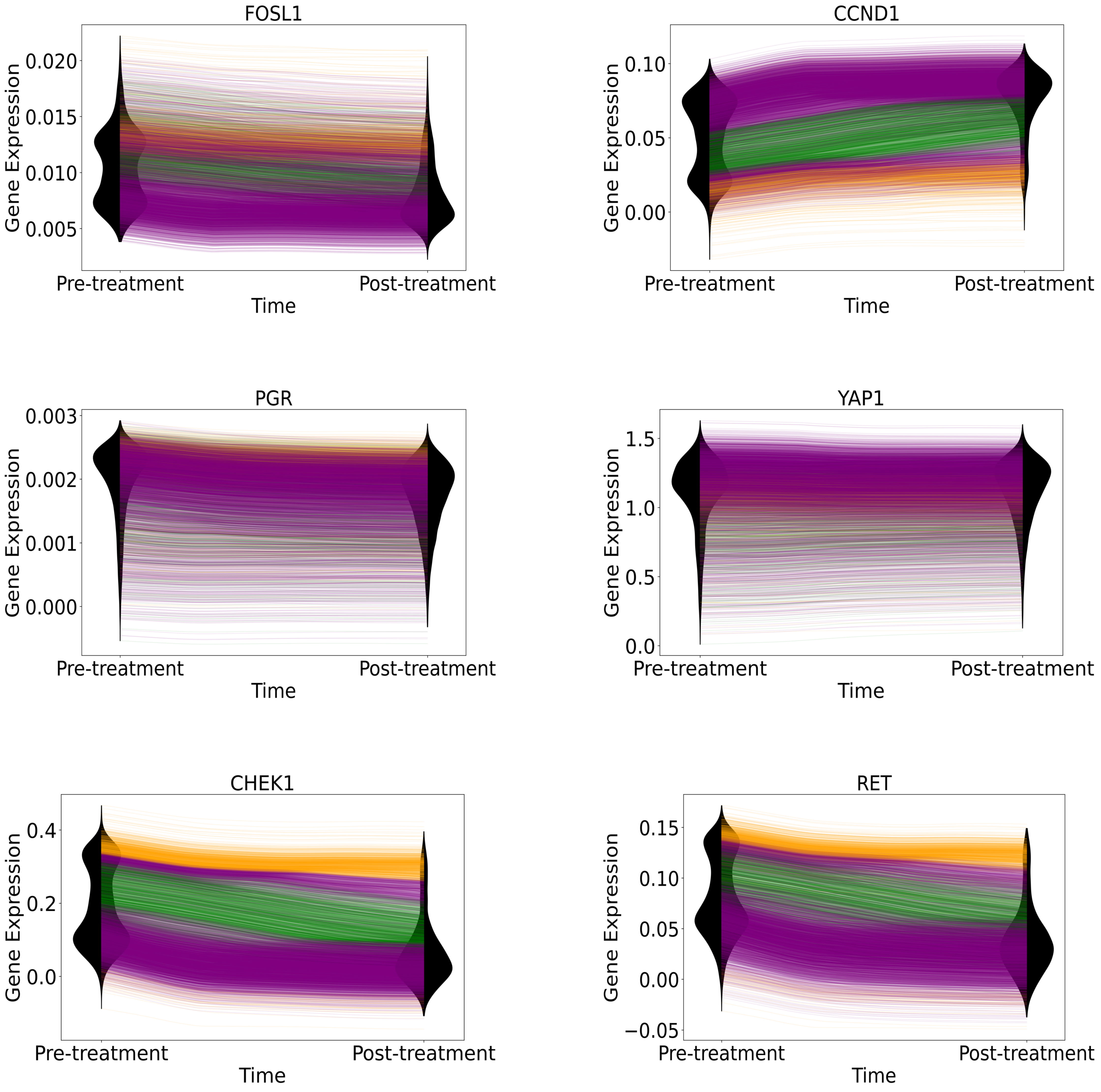

FOSL1
Gene Expression
0.020
0.015
0.010
0.005
Pre-treatment
Post-treatment
Time
CCND1
Gene Expression
0.10
0.05
0.00
Pre-treatment
Post-treatment
Time
PGR
Gene Expression
0.003
0.002
0.001
0.000
Pre-treatment
Post-treatment
Time
YAP1
Gene Expression
1.5
1.0
0.5
0.0
Pre-treatment
Post-treatment
Time
CHEK1
Gene Expression
0.4
0.2
0.0
Pre-treatment
Post-treatment
Time
RET
Gene Expression
0.15
0.10
0.05
0.00
−0.05
Pre-treatment
Post-treatment
Time

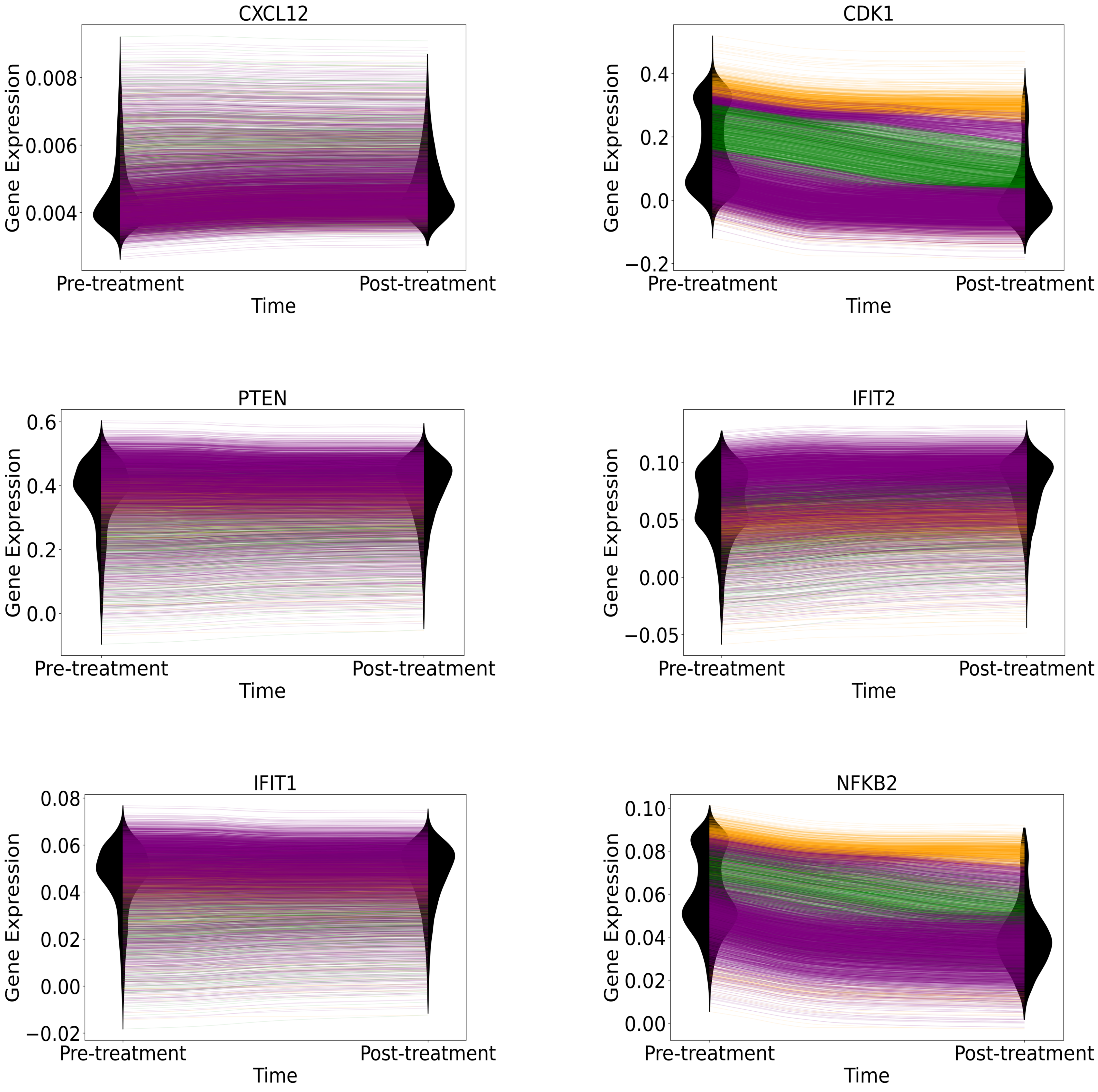
CXCL12
Gene Expression
0.008
0.006
0.004
Pre-treatment
Post-treatment
Time
CDK1
Gene Expression
0.4
0.2
0.0
−0.2
Pre-treatment
Post-treatment
Time
PTEN
Gene Expression
0.6
0.4
0.2
0.0
Pre-treatment
Post-treatment
Time
IFIT2
Gene Expression
0.10
0.05
0.00
−0.05
Pre-treatment
Post-treatment
Time
IFIT1
Gene Expression
0.08
0.06
0.04
0.02
0.00
−0.02
Pre-treatment
Post-treatment
Time
NFKB2
Gene Expression
0.10
0.08
0.06
0.04
0.02
0.00
Pre-treatment
Post-treatment
Time

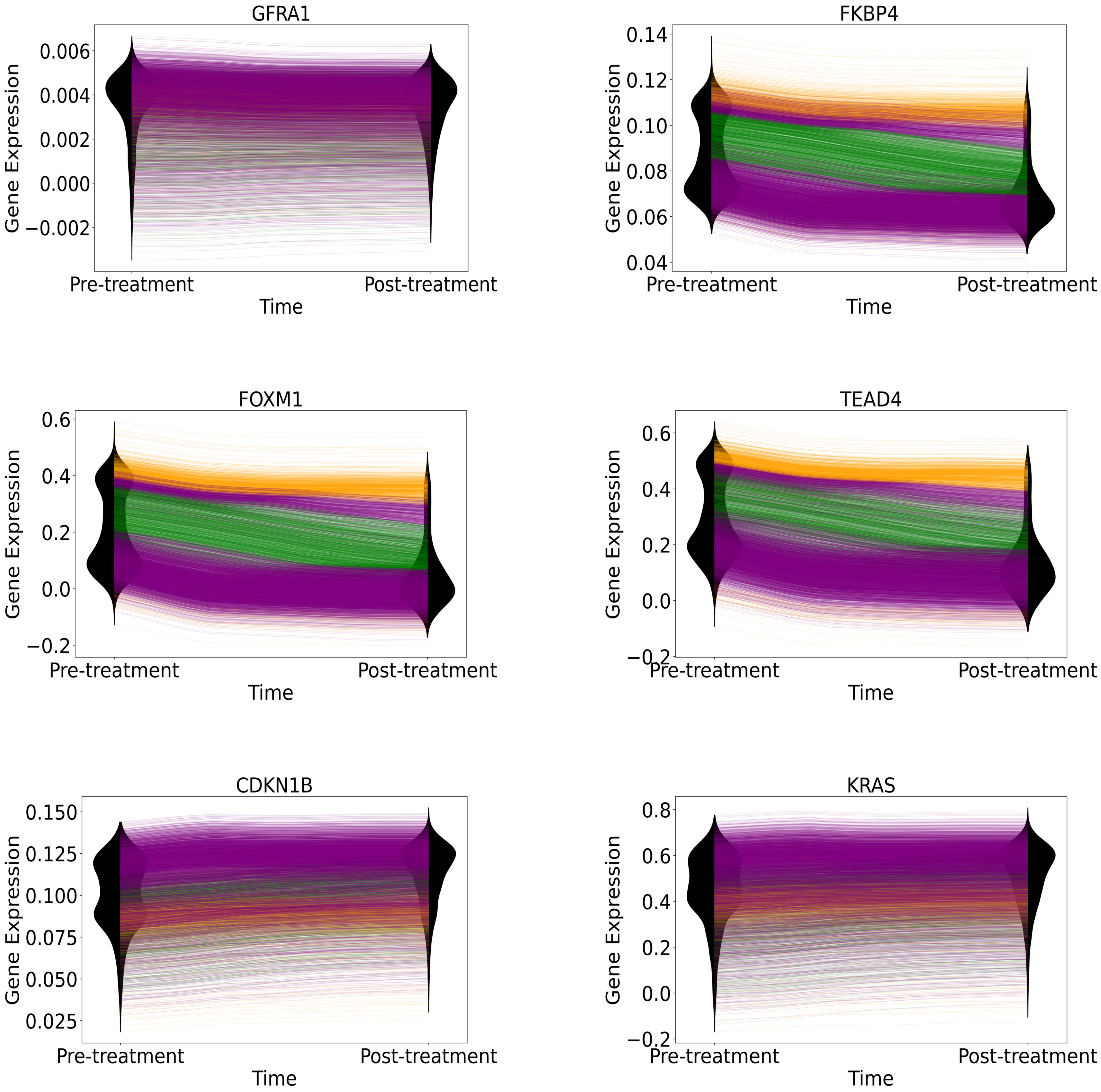

GFRA1
FKBP4
FOXM1
TEAD4
CDKN1B
KRAS
Gene Expression
Time
Pre-treatment
Post-treatment
0.006
0.004
0.002
0.000
−0.002
0.14
0.12
0.10
0.08
0.06
0.04
0.6
0.4
0.2
0.0
−0.2
0.150
0.125
0.100
0.075
0.050
0.025
0.8

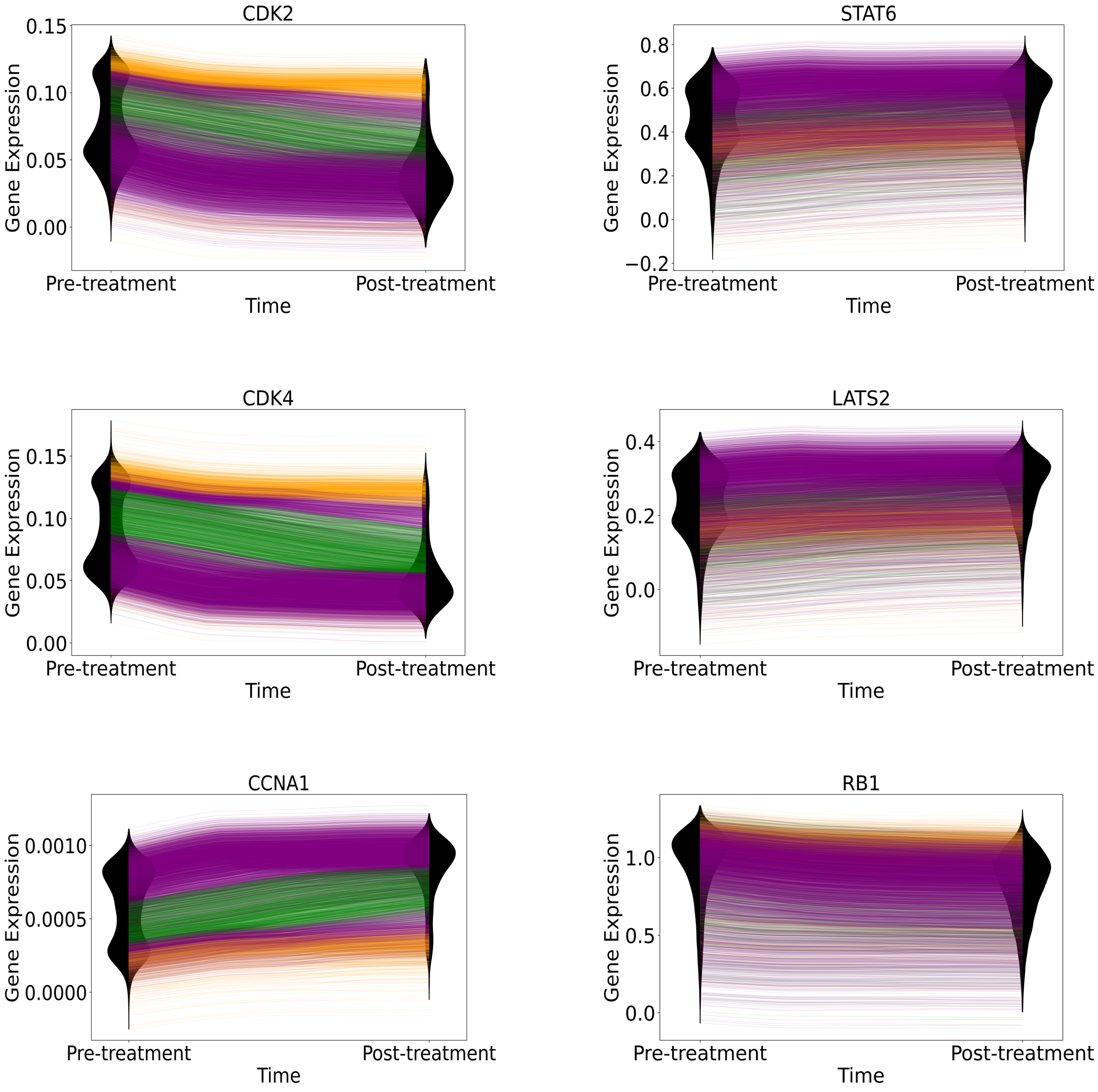
CDK2
Gene Expression
0.15
0.10
0.05
0.00
Pre-treatment
Post-treatment
Time
STAT6
Gene Expression
0.8
0.6
0.4
0.2
0.0
−0.2
Pre-treatment
Post-treatment
Time
CDK4
Gene Expression
0.15
0.10
0.05
0.00
Pre-treatment
Post-treatment
Time
LATS2
Gene Expression
0.4
0.2
0.0
Pre-treatment
Post-treatment
Time
CCNA1
Gene Expression
0.0010
0.0005
0.0000
Pre-treatment
Post-treatment
Time
RB1
Gene Expression
1.0
0.5
0.0
Pre-treatment
Post-treatment
Time

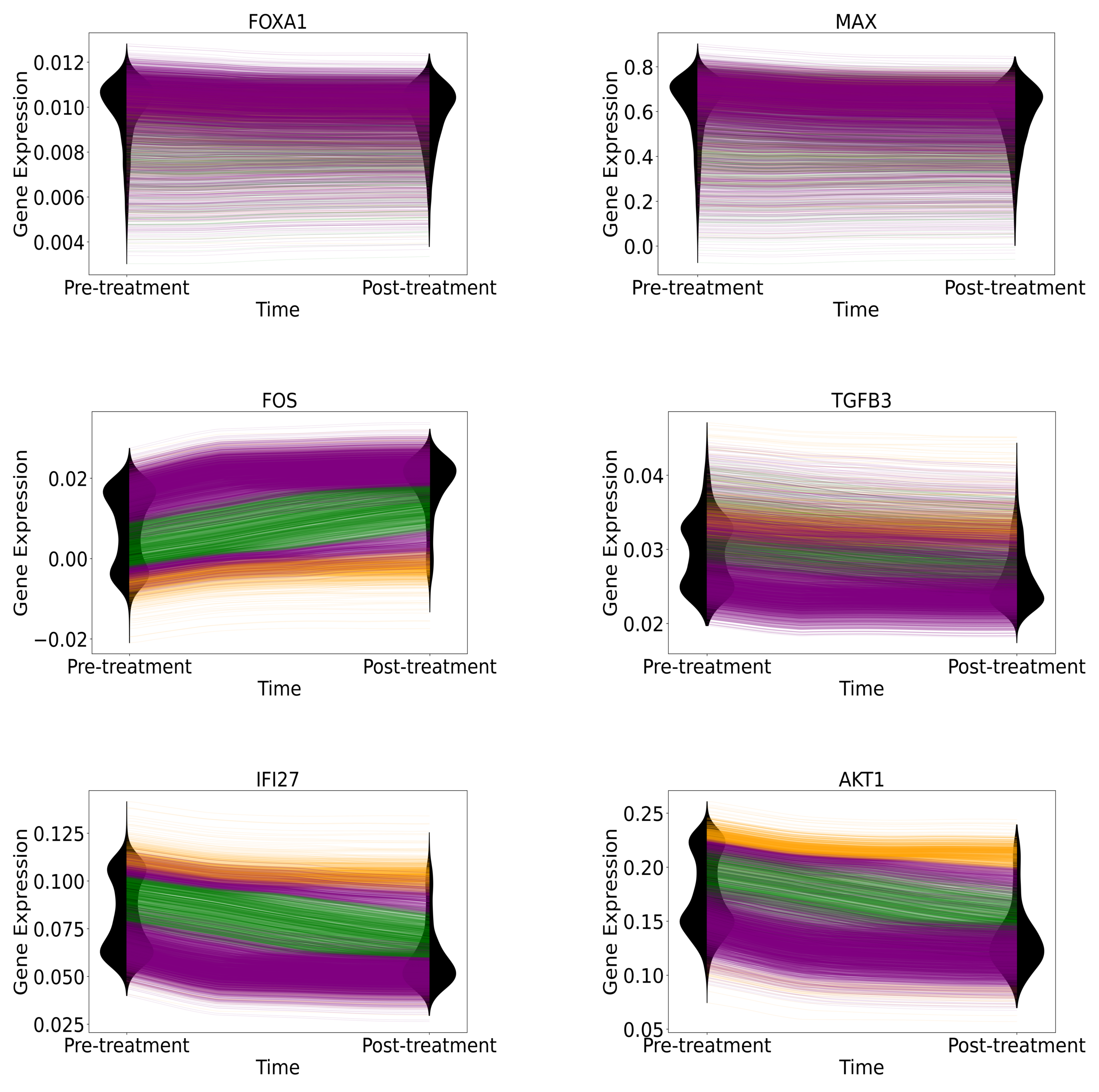

FOXA1
Gene Expression
0.012
0.010
0.008
0.006
0.004
Pre-treatment
Post-treatment
Time
MAX
Gene Expression
0.8
0.6
0.4
0.2
0.0
Pre-treatment
Post-treatment
Time
FOS
Gene Expression
0.02
0.00
−0.02
Pre-treatment
Post-treatment
Time
TGFB3
Gene Expression
0.04
0.03
0.02
Pre-treatment
Post-treatment
Time
IFI27
Gene Expression
0.125
0.100
0.075
0.050
0.025
Pre-treatment
Post-treatment
Time
AKT1
Gene Expression
0.25
0.20
0.15
0.10
0.05
Pre-treatment
Post-treatment
Time

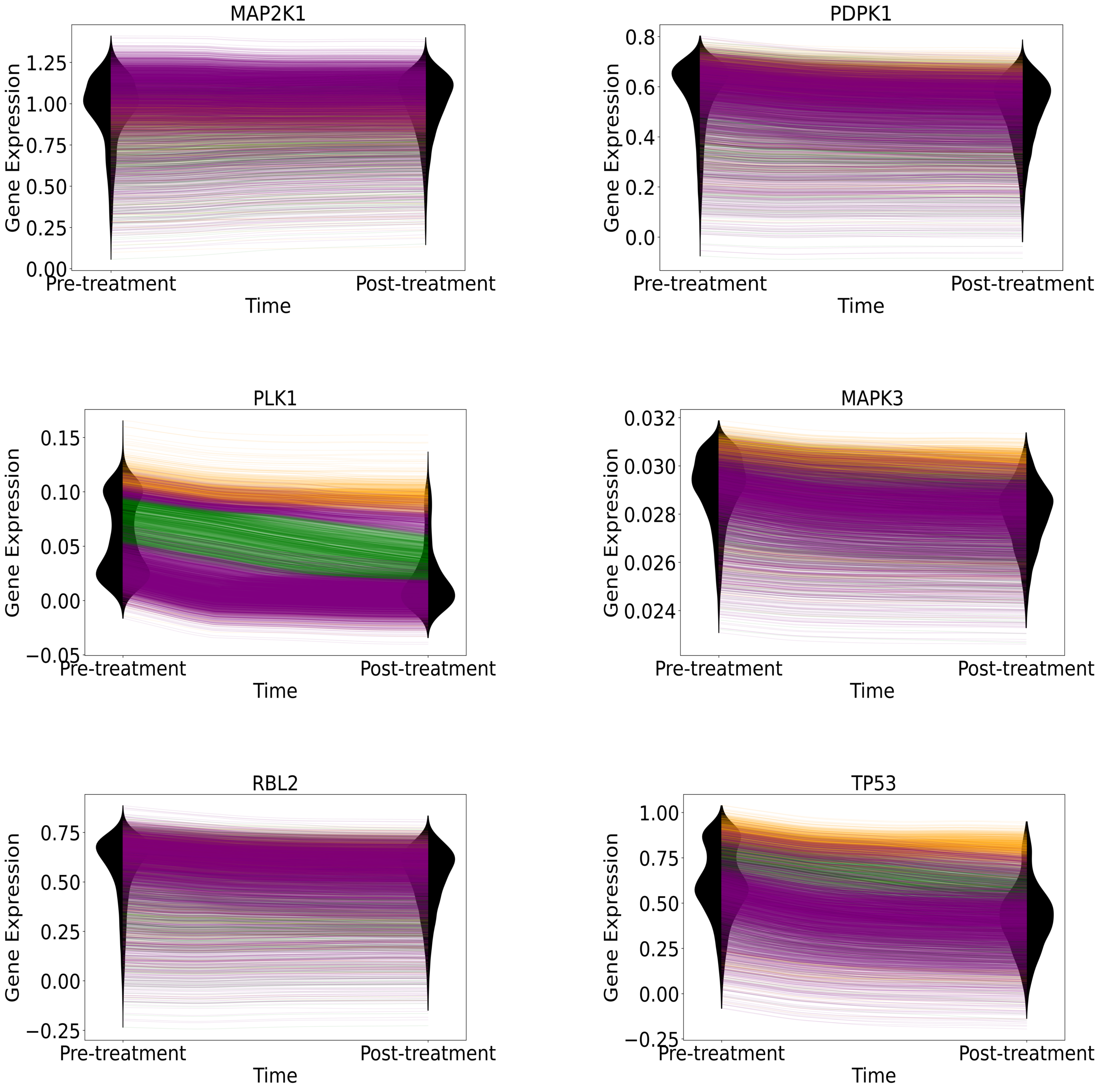

MAP2K1
PDPK1
PLK1
MAPK3
RBL2
TP53
Gene Expression
Time
Pre-treatment
Post-treatment
0.00
0.25
0.50
0.75
1.00
1.25
0.0
0.2
0.4
0.6
0.8
−0.05
0.05
0.10
0.15
0.024
0.026
0.028
0.030
0.032
−0.25

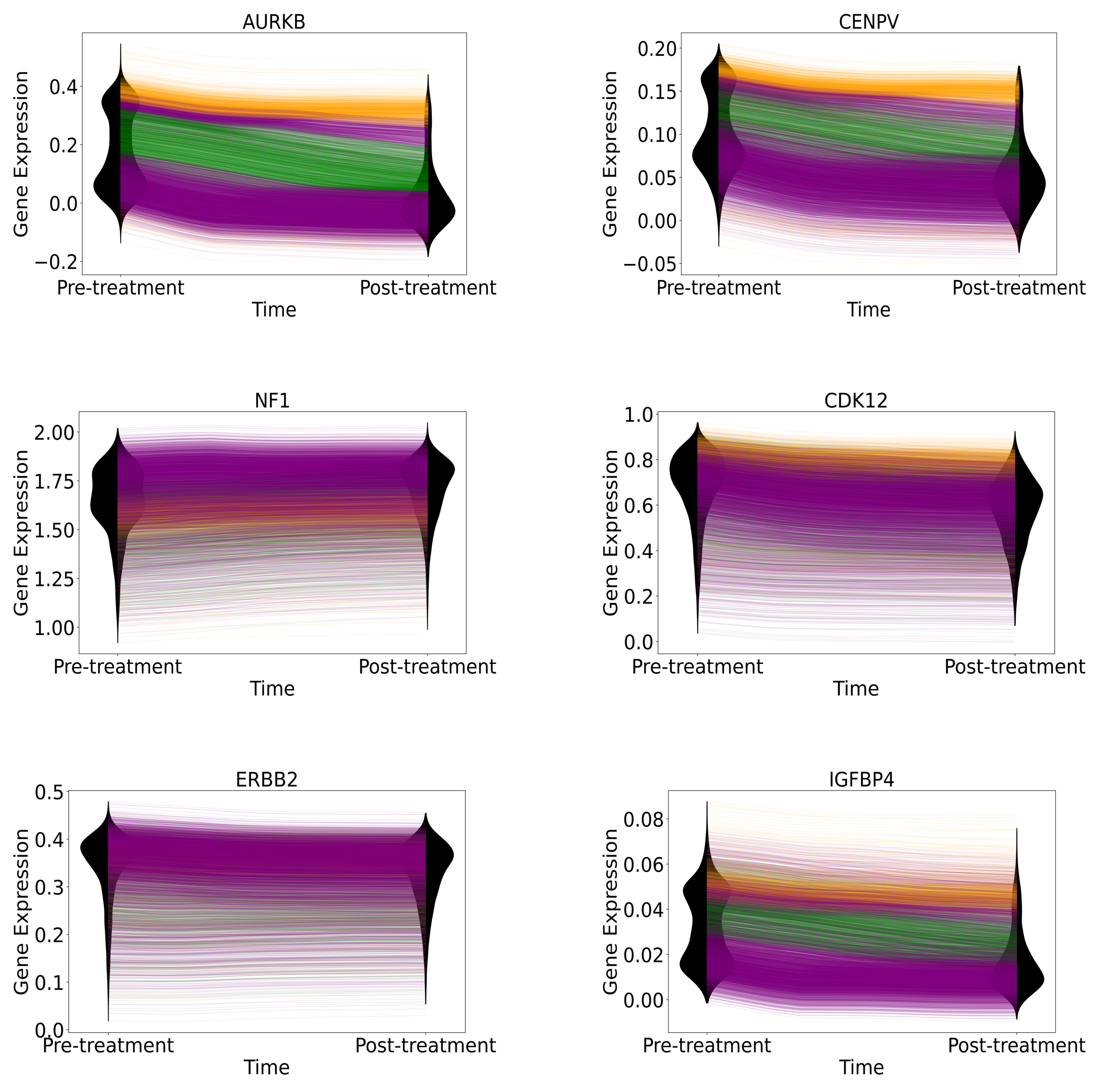
AURKB
CENPV
NF1
CDK12
ERBB2
IGFBP4
Gene Expression
Time
Pre-treatment
Post-treatment
0.4
0.2
0.0
−0.2
0.20
0.15
0.10
0.05
0.00
−0.05
2.00
1.75
1.50
1.25
1.00
1.0
0.8
0.6
0.4
0.2
0.0
0.5
0.4
0.3
0.2
0.1
0.0
0.08
0.06
0.04
0.02
0.00

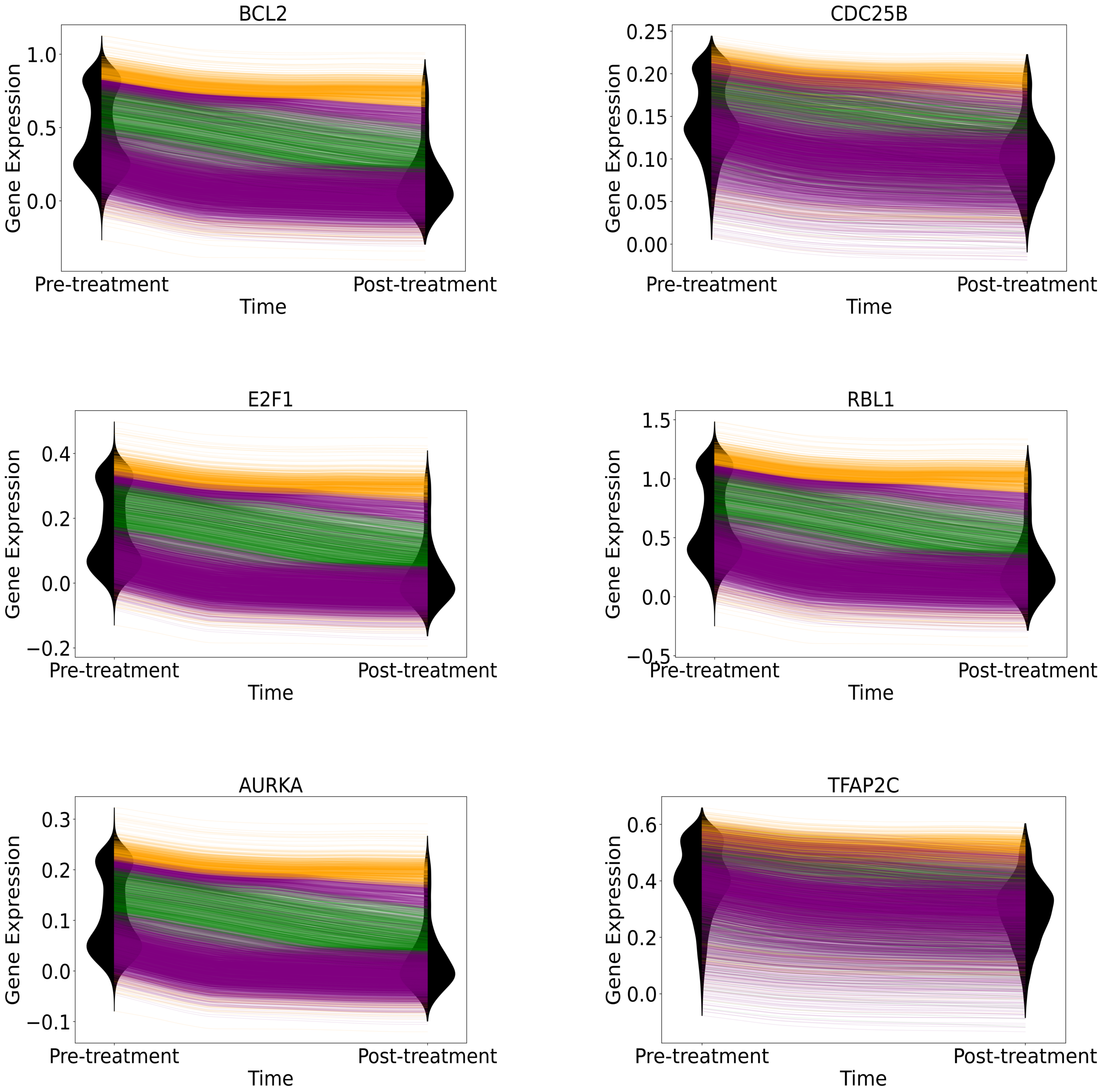
BCL2
CDC25B
E2F1
RBL1
AURKA
TFAP2C
Gene Expression
Time
Pre-treatment
Post-treatment

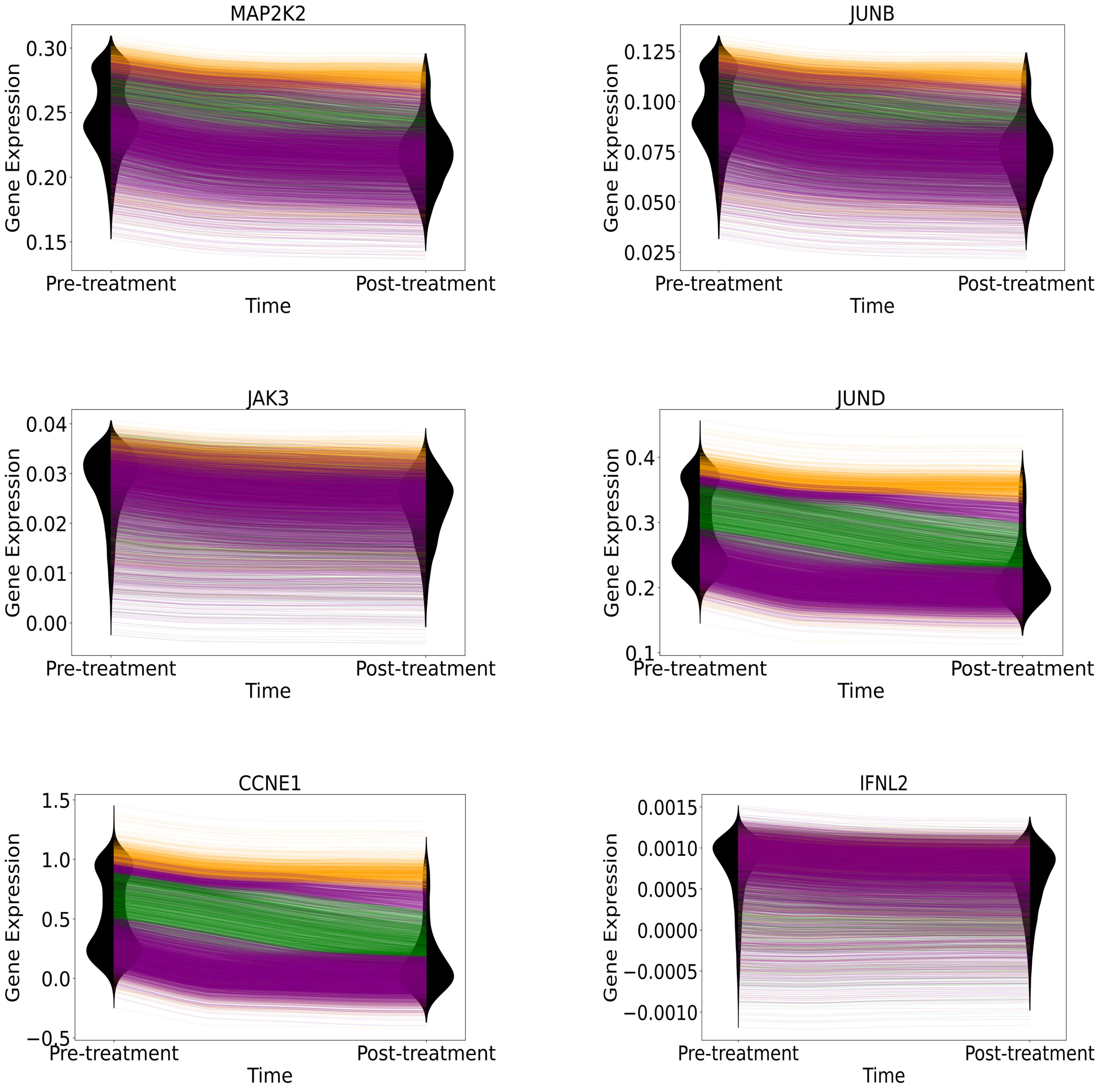
MAP2K2
JUNB
JAK3
JUND
CCNE1
IFNL2
Gene Expression
Time
Pre-treatment
Post-treatment
0.30
0.25
0.20
0.15
0.125
0.100
0.075
0.050
0.025
0.04
0.03
0.02
0.01
0.00
0.4
0.3
0.2
0.1
1.5
1.0
0.5
0.0
−0.5
0.0015
0.0010
0.0005
0.0000
−0.0005
−0.0010

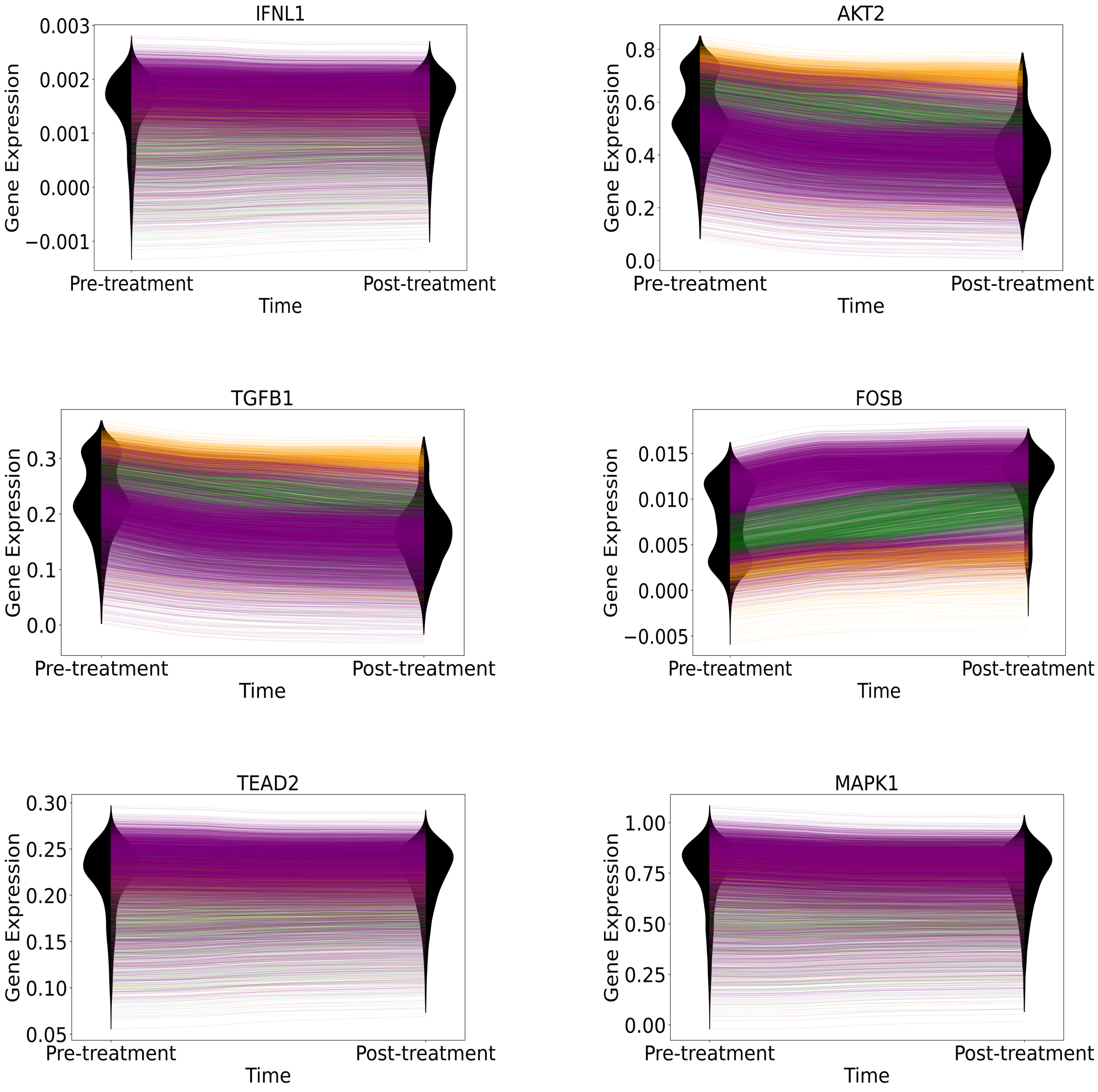
IFNL1
AKT2
TGFB1
FOSB
TEAD2
MAPK1
Gene Expression
Time
Pre-treatment
Post-treatment

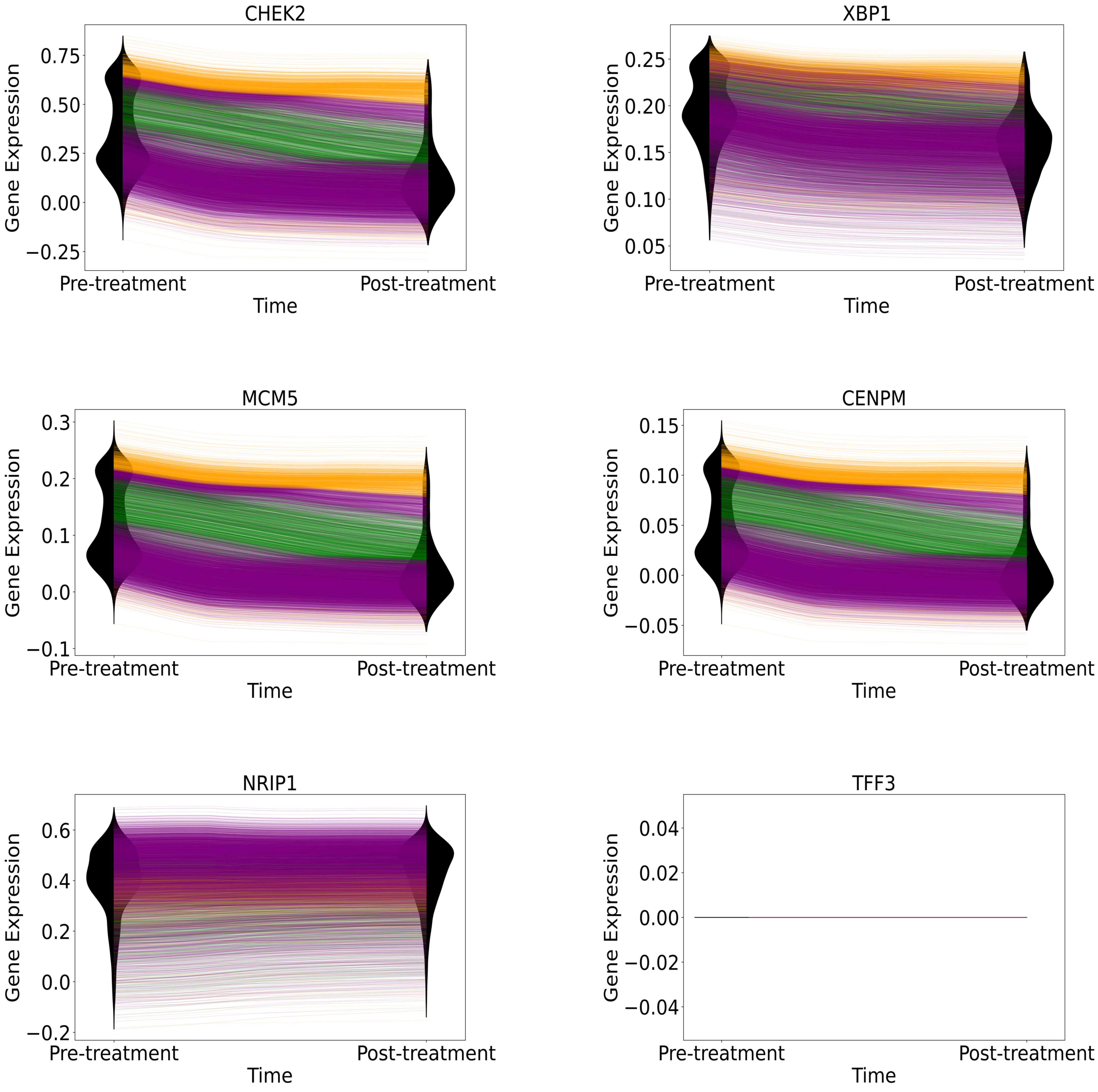

CHEK2
XBP1
MCM5
CENPM
NRIP1
TFF3
Gene Expression
Time
Pre-treatment
Post-treatment
0.75
0.50
0.25
0.00
−0.25
0.25
0.20
0.15
0.10
0.05
0.3
0.2
0.1
0.0
−0.1
0.15
0.10
0.05
0.00
−0.05
0.6
0.4
0.2
0.0
−0.2
0.04
0.02
0.00
−0.02
−0.04

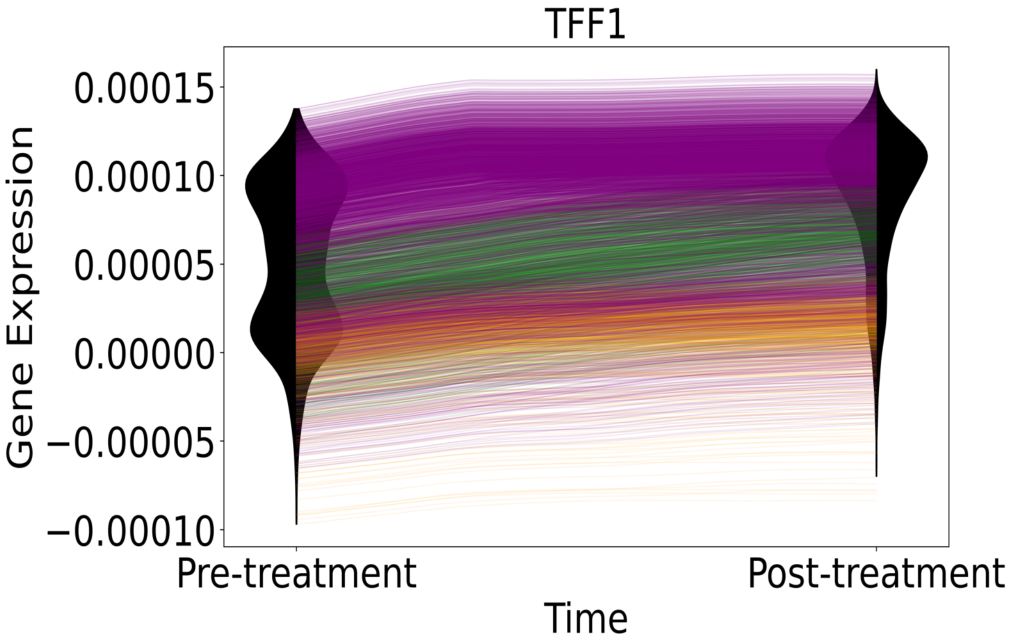

Supplementary Figure 12: Individual cells across three patient datasets. Violin plots show real expression data; colored lines represent predicted trajectories from three subgroups of cells defined by low, medium, and high phenotypic shift levels, as described in Figure 5.
