# Supplementary material for: PROFET Predicts Continuous Gene Expression Dynamics from scRNA-seq Data to Elucidate Heterogeneity of Cancer Treatment Responses
Source: bioRxiv. 2025 Jul 3:2025.06.27.662030. Preprint. [Version 1] doi: 10.1101/2025.06.27.662030 (PMC12236938; doi:10.1101/2025.06.27.662030)
Supplement: Supplement 12 [file media-14.pdf]

A

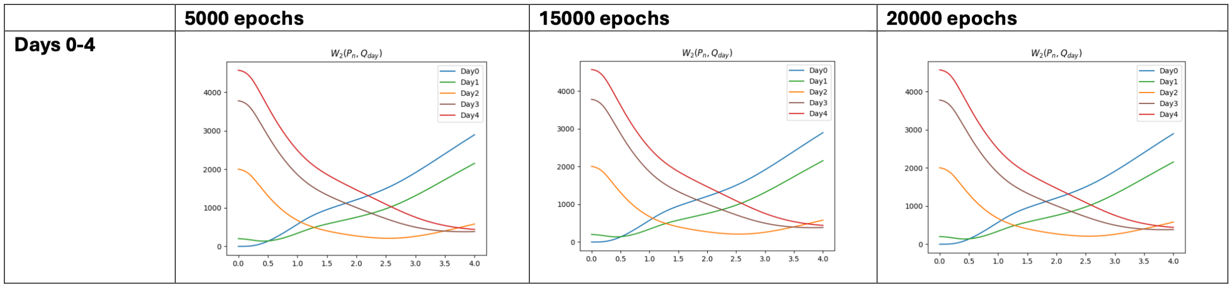

B

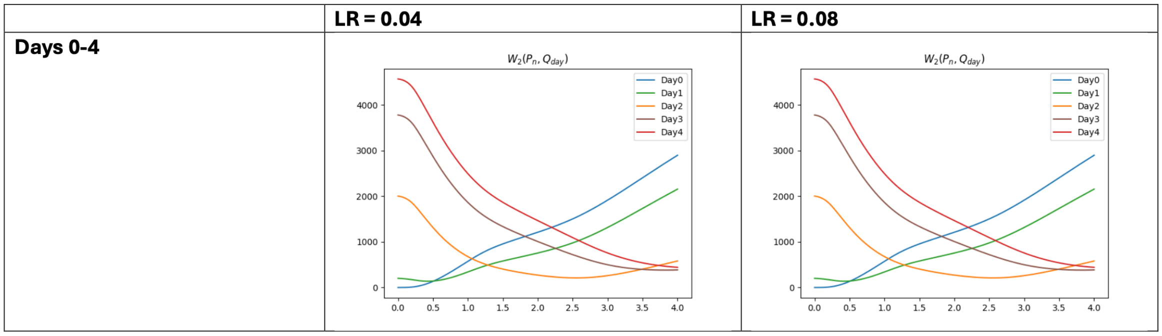

C

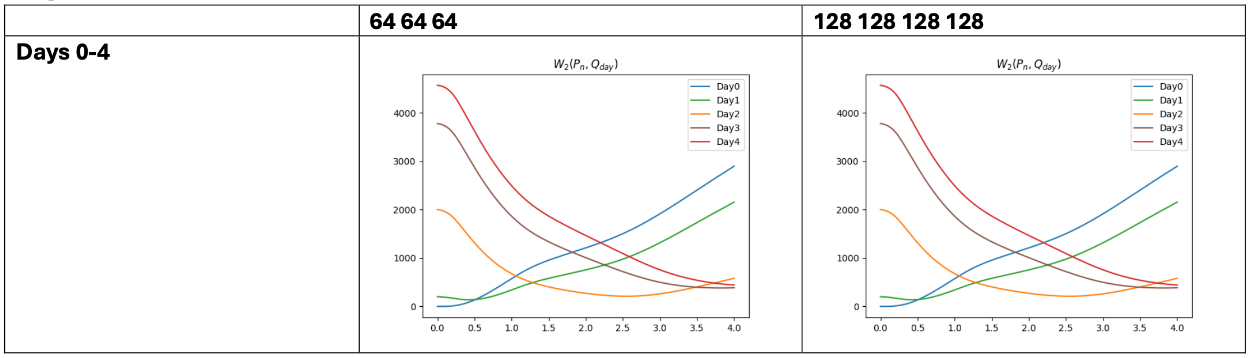

D

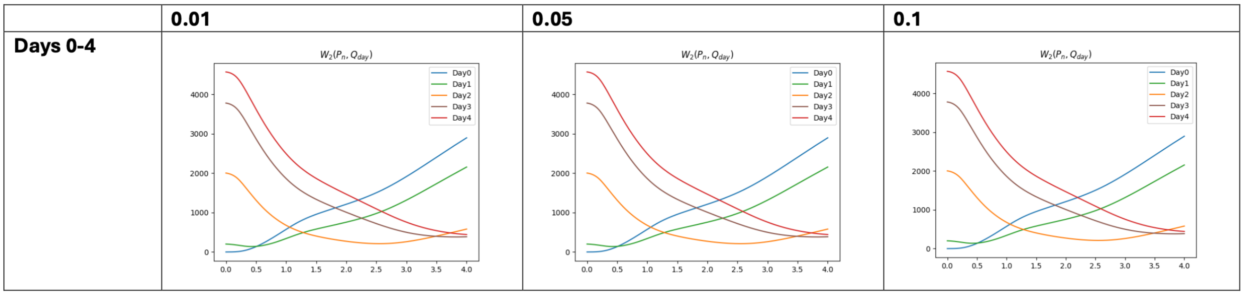

Supplementary Figure 13: Effect of the number of training epochs on the Wasserstein distance between predicted trajectories and real data over time. Lower distances indicate better alignment between predicted and observed trajectories.
