# Supplementary material for: PROFET Predicts Continuous Gene Expression Dynamics from scRNA-seq Data to Elucidate Heterogeneity of Cancer Treatment Responses
Source: bioRxiv. 2025 Jul 3:2025.06.27.662030. Preprint. [Version 1] doi: 10.1101/2025.06.27.662030 (PMC12236938; doi:10.1101/2025.06.27.662030)
Supplement: Supplement 13 [file media-15.pdf]

mESC permutation test for time 1 and time 2 distribution predictions

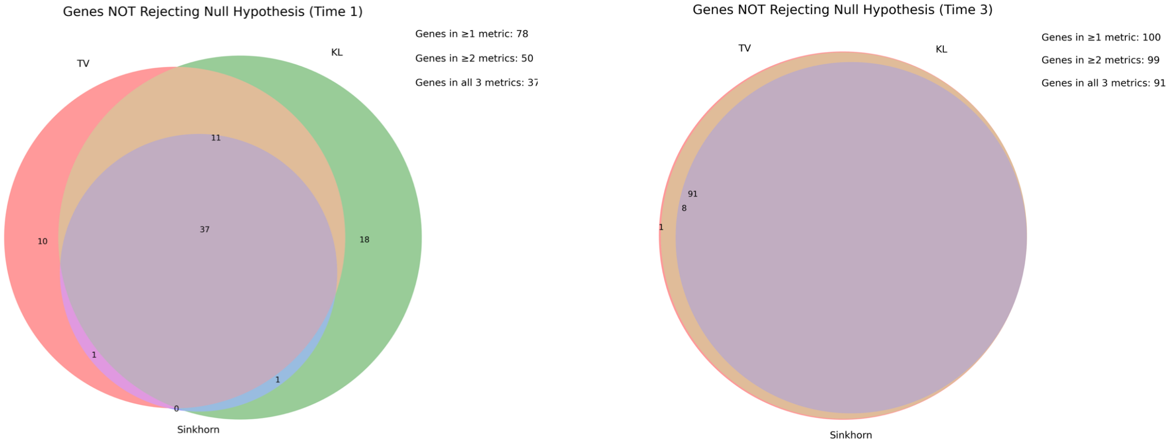

At least one metric (TV, KL, or Sinkhorn) showing No AFF1, BCL3, BHLHE40, BMP2, CARHSP1, CREB3, DDIT3, DNMT3A, DNMT3B, DNMT3L, EGR1, ELF3, EPAS1, ERF, ESRRB, ETV4, ETV5, FOXD3, FOXH1, GLI1, GLI2, HMGA1, HMGA2, HNRNPK, HOPX, ID2, ID3, JARID2, JUN, KDM5B, KLF10, KLF2, KLF3, KLF4, KLF6, L3MBTL3, MSC, MSX2, MTF2, MYBL2, NANOG, NR0B1, PARP1, PEG3, PHB, POU4F2, POU5F1, PURB, RARG, RBPJ, REST, RHOX6, RUNX1, SIX1, SMAD7, SNAI1, SOX2, TBX3, TCF15, TCF7L2, TET1, TET2, TFCP2L1, TFEB, TGIF1, TRP53, UTF1, ZBTB10, ZBTB44, ZC3H7A, ZFHX3, ZFP296, ZFP42, ZFP428, ZFP462, ZFP532, ZFP57, ZFP710

At least two metrics (TV, KL, or Sinkhorn) showing No
BCL3, BHLHE40, CARHSP1, CREB3, DDIT3, EPAS1, ERF, ESRRB, ETV4, ETV5, FOXH1, HMGA1, HNRNPK, HOPX, ID2, JARID2, JUN, KDM5B, KLF10, KLF3, KLF4, KLF6, L3MBTL3, MSC, MSX2, MTF2, MYBL2, PARP1, PEG3, PHB, POU5F1, PURB, RBPJ, REST, RHOX6, SNAI1, TCF15, TCF7L2, TET1, TET2, TFCP2L1, TRP53, UTF1, ZBTB44, ZC3H7A, ZFHX3, ZFP296, ZFP42, ZFP532, ZFP710

All three metrics (TV, KL, and Sinkhorn) showing No
BCL3, CARHSP1, CREB3, DDIT3, ERF, ETV4, HMGA1, HNRNPK, ID2, JARID2, JUN, KLF10, KLF3, KLF6, L3MBTL3, MSC, MSX2, MTF2, MYBL2, PARP1, PEG3, POU5F1, PURB, RBPJ, REST, RHOX6, SNAI1, TCF15, TCF7L2, TET2, TFCP2L1, TRP53, UTF1, ZBTB44, ZFHX3, ZFP296, ZFP532

EMT permutation test for time 1 and time 2 distribution predictions

At least one metric (TV, KL, or Sinkhorn) showing No ANKRD22, AP1M2, AP1M2.1, BSPRY, C1orf116, C1orf172, CDH1, CDH3, CDS1, CLDN4, CLDN7, CRB3, DSP, ELMO3, ENPP5, EPB41L5, EPCAM, EPHA1, EPN3, EPPK1, ERBB3, ESRP1, EVPL, F11R, FN1, FXYD3, GALNT3, GPR110, GPR56, GRHL1, INADL, ITGB6, KLC3, KRTCAP3, MAL2, MAPK13, MMP2, MPP7, MPZL2, MUC1, PRR5, PRSS22, PRSS8, RAB25, RBPMS, S100A14, SCNN1A, SERINC2, SH3YL1, SHROOM3, SPINT2, SSH3, ST14, STAP2, TACSTD2, TC2N, TJP3, TMC4, TMEM125, TMEM45B, TNFRSF21, TSKU

At least two metrics (TV, KL, or Sinkhorn) showing No
DSP, ENPP5, EPB41L5, KRTCAP3, MMP2, RAB25, SERINC2, TMEM45B

All three metrics (TV, KL, and Sinkhorn) showing No
RAB25

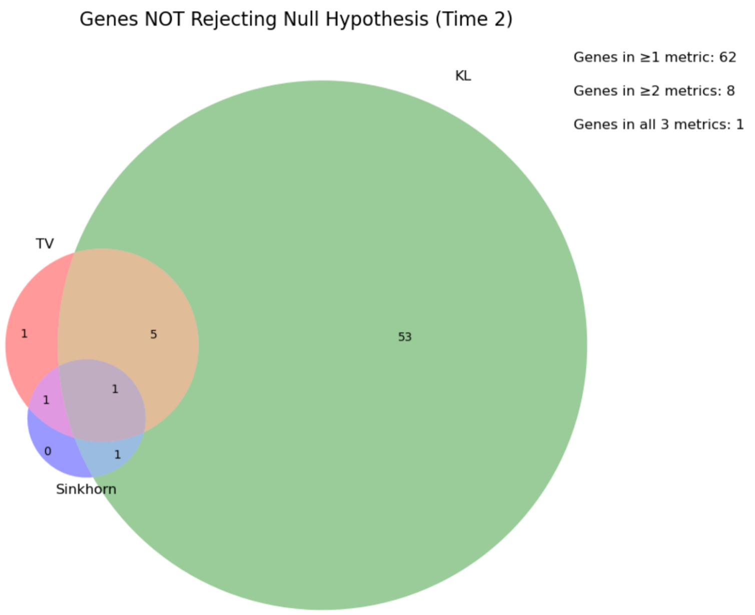

Supplementary Figure 14: Venn diagram and the list of genes which has not statistically difference through permutation tests under using metrics of KL, TV and Sinkhorm.Venn diagram and gene lists showing overlap among genes with no statistically significant differences based on permutation tests using KL divergence, total variation (TV), and Sinkhorn distance metrics.
