# Supplementary material for: PROFET Predicts Continuous Gene Expression Dynamics from scRNA-seq Data to Elucidate Heterogeneity of Cancer Treatment Responses
Source: bioRxiv. 2025 Jul 3:2025.06.27.662030. Preprint. [Version 1] doi: 10.1101/2025.06.27.662030 (PMC12236938; doi:10.1101/2025.06.27.662030)
Supplement: Supplement 14 [file media-16.pdf]

A

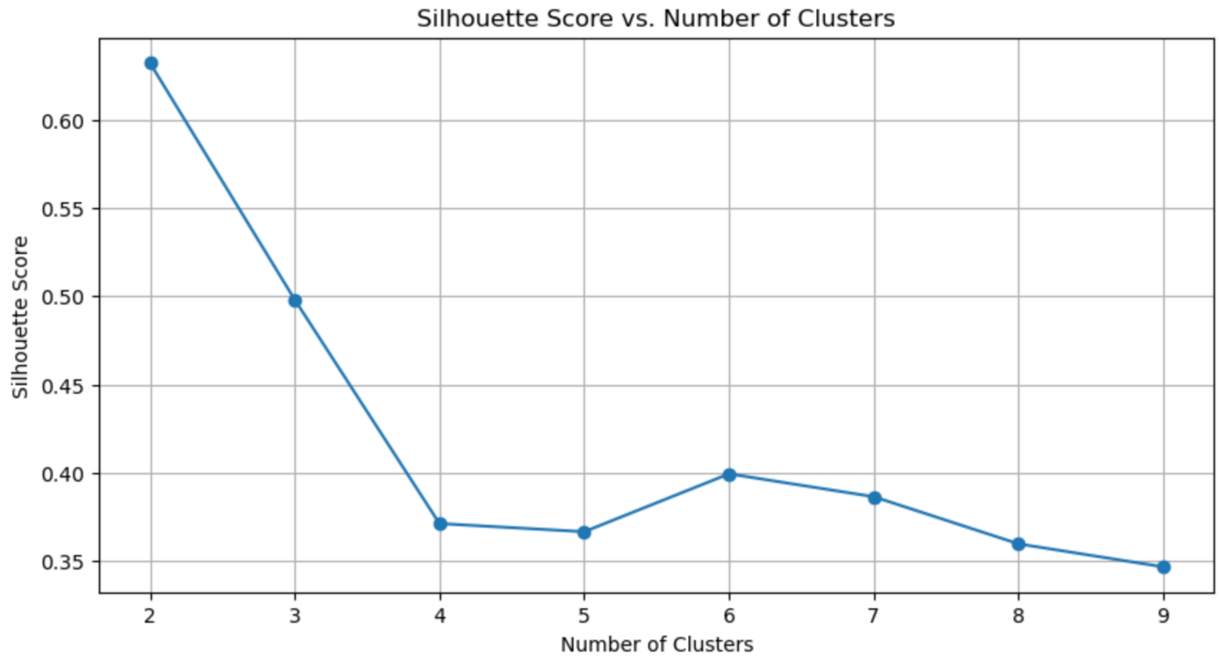

B

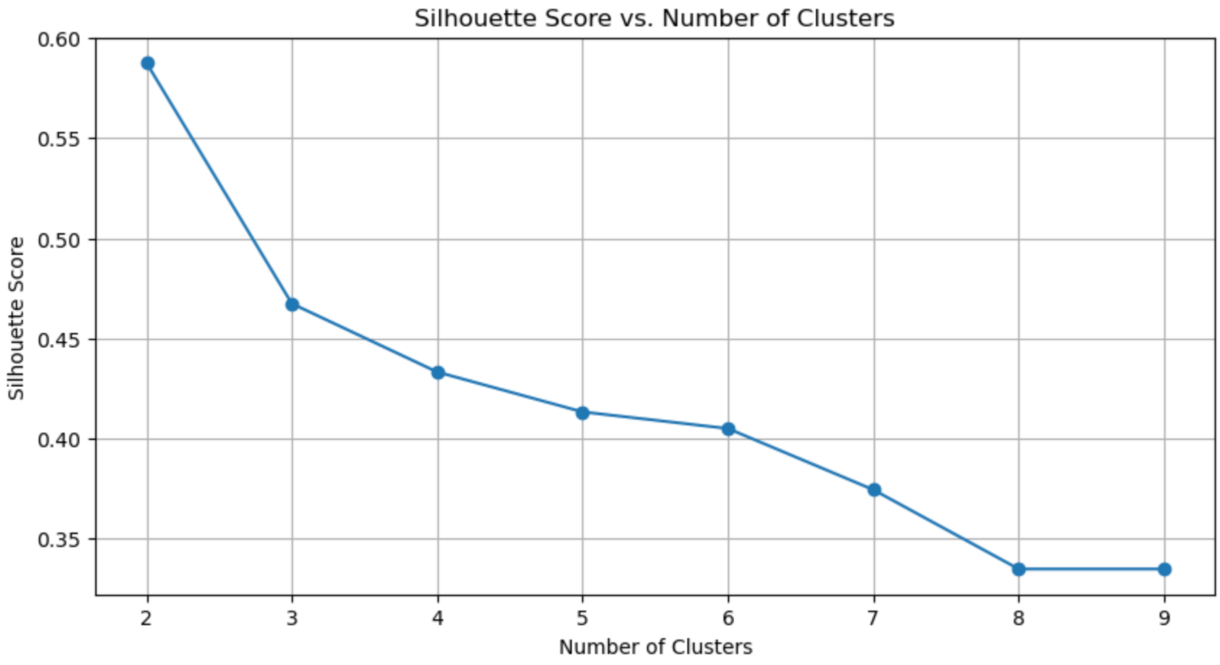

Supplementary Figure 15: Silhouette scores from clustering analysis across different numbers of clusters for (A) time point 4 of the mESC dataset and (B) day 0 of the EMT dataset.
