# Supplementary material for: PROFET Predicts Continuous Gene Expression Dynamics from scRNA-seq Data to Elucidate Heterogeneity of Cancer Treatment Responses
Source: bioRxiv. 2025 Jul 3:2025.06.27.662030. Preprint. [Version 1] doi: 10.1101/2025.06.27.662030 (PMC12236938; doi:10.1101/2025.06.27.662030)
Supplement: Supplement 15 [file media-17.pdf]

A

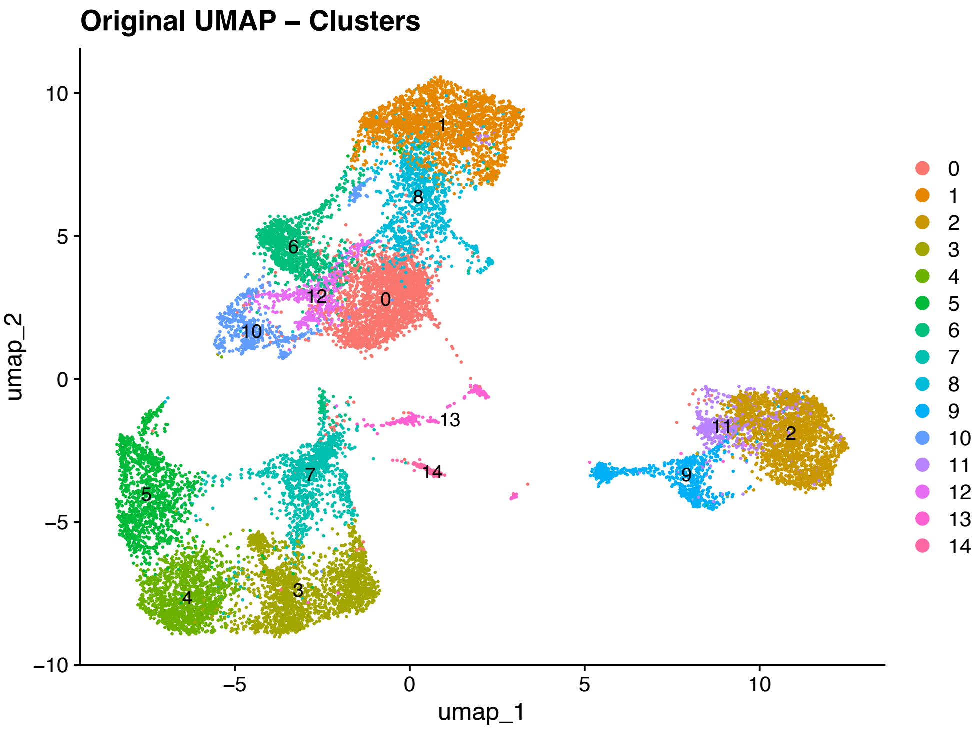

Original UMAP – Clusters
umap_2
umap_1
10
5
0
−5
−10
0
1
2
3
4
5
6
7
8
9
10
11
12
13
14

B

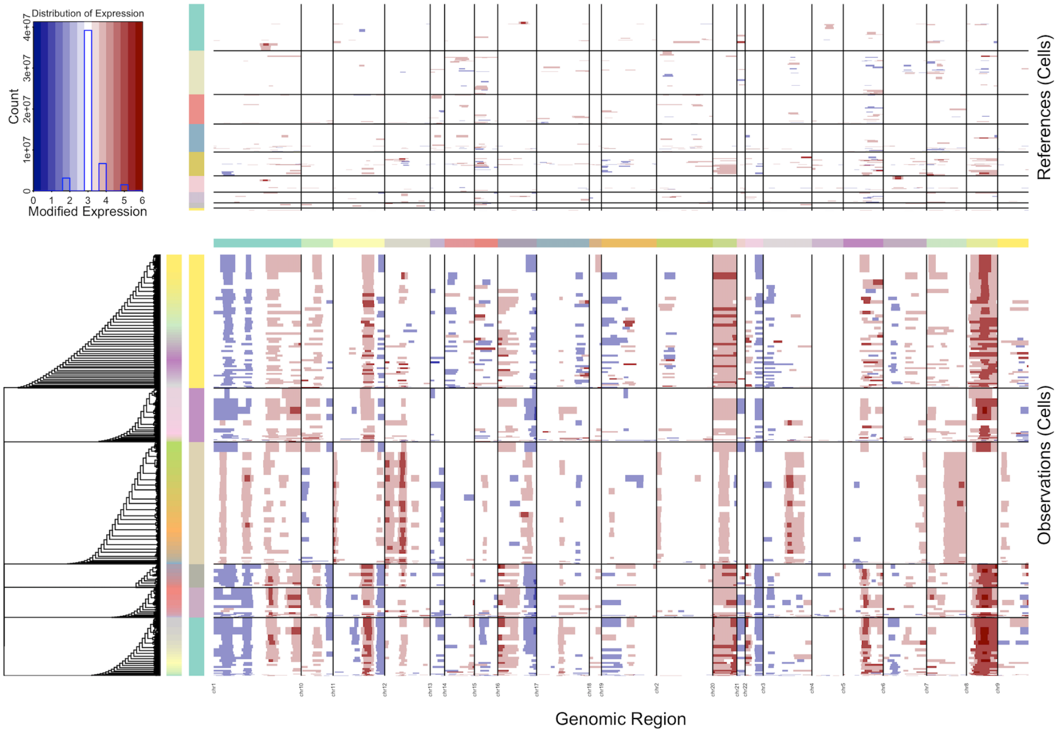

Distribution of Expression
Count
Modified Expression
References (Cells)
Observations (Cells)
Genomic Region

C

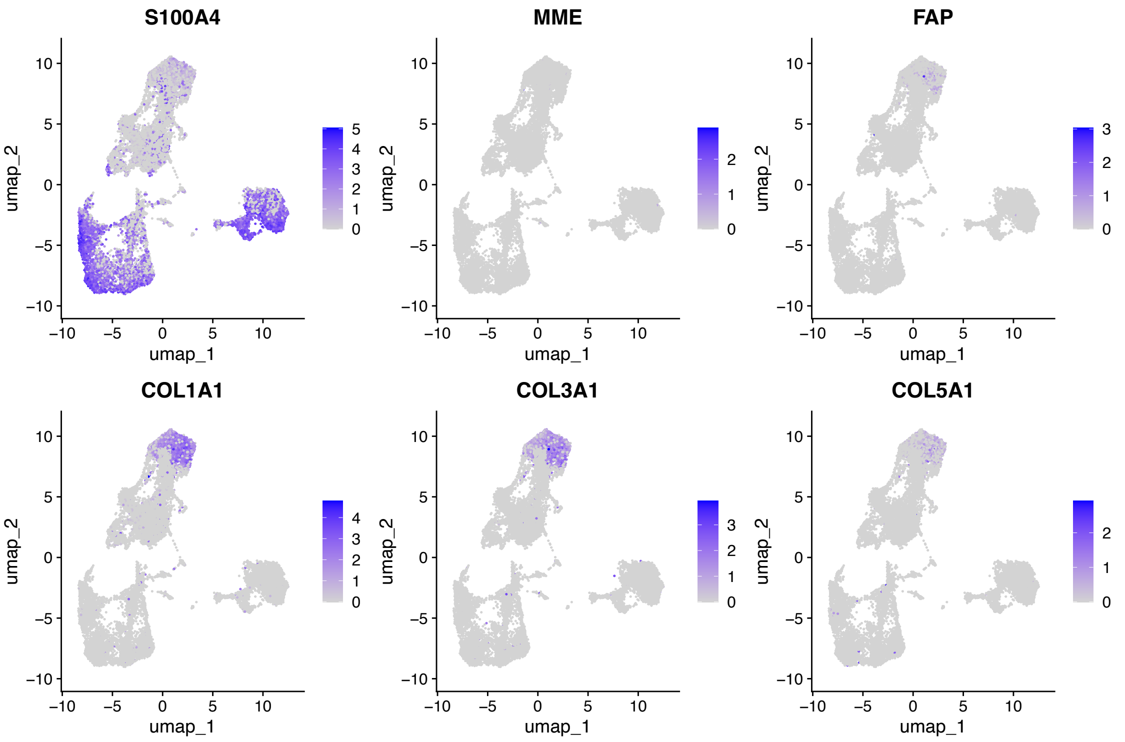
S100A4
MME
FAP
COL1A1
COL3A1
COL5A1
umap_1
umap_2

D

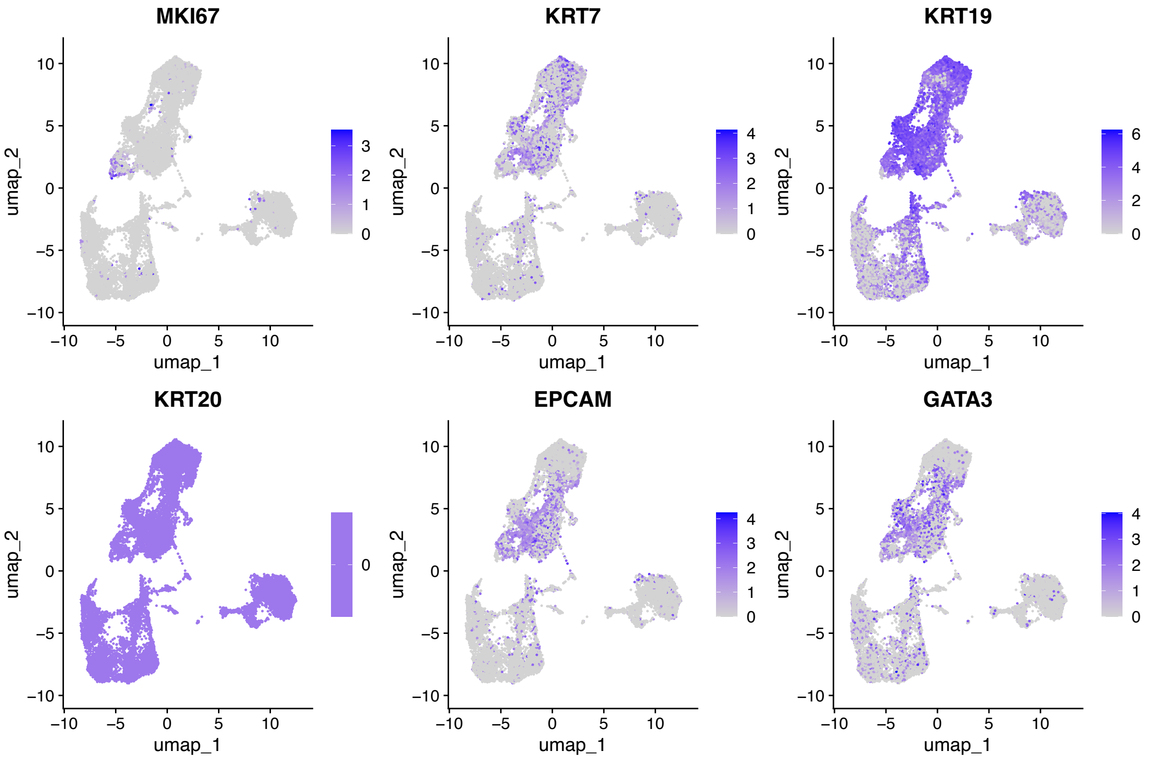
MKI67
KRT7
KRT19
KRT20
EPCAM
GATA3
umap_1
umap_2

Supplementary Figure 16: (A) Clustering results of single-cell data.
(B) inferCNV analysis for identifying large-scale chromosomal copy number variations.
(C) Gene expression heatmap for fibroblast marker genes.
(D) Gene expression heatmap for epithelial marker genes.
